# Use of ESI-FTICR-MS to Characterize Dissolved Organic Matter in Headwater Streams Draining Forest-Dominated and Pasture-Dominated Watersheds

**DOI:** 10.1371/journal.pone.0145639

**Published:** 2015-12-29

**Authors:** YueHan Lu, Xiaping Li, Rajaa Mesfioui, James E. Bauer, R. M. Chambers, Elizabeth A. Canuel, Patrick G. Hatcher

**Affiliations:** 1 Department of Geological Sciences, University of Alabama, Tuscaloosa, Alabama, United States of America; 2 Department of Chemistry and Biochemistry, Old Dominion University, Norfolk, Virginia, United States of America; 3 Aquatic Biogeochemistry Laboratory, Department of Evolution, Ecology and Organismal Biology, Ohio State University, Columbus, Ohio, United States of America; 4 Department of Biology, College of William and Mary, Williamsburg, Virginia, United States of America; 5 Department of Physical Sciences, Virginia Institute of Marine Sciences, Gloucester Point, Virginia, United States of America; Pacific Northwest National Laboratory, UNITED STATES

## Abstract

Electrospray ionization Fourier transform ion cyclotron resonance mass spectrometry (ESI-FTICR-MS) has proven to be a powerful technique revealing complexity and diversity of natural DOM molecules, but its application to DOM analysis in grazing-impacted agricultural systems remains scarce. In the present study, we presented a case study of using ESI-FTICR-MS in analyzing DOM from four headwater streams draining forest- or pasture-dominated watersheds in Virginia, USA. In all samples, most formulas were CHO compounds (71.8–87.9%), with other molecular series (CHOS, CHON, CHONS, and CHOP (N, S)) accounting for only minor fractions. All samples were dominated by molecules falling in the lignin-like region (H/C = 0.7–1.5, O/C = 0.1–0.67), suggesting the predominance of allochthonous, terrestrial plant-derived DOM. Relative to the two pasture streams, DOM formulas in the two forest streams were more similar, based on Jaccard similarity coefficients and nonmetric multidimensional scaling calculated from Bray-Curtis distance. Formulas from the pasture streams were characterized by lower proportions of aromatic formulas and lower unsaturation, suggesting that the allochthonous versus autochthonous contributions of organic matter to streams were modified by pasture land use. The number of condensed aromatic structures (CAS) was higher for the forest streams, which is possibly due to the controlled burning in the forest-dominated watersheds and suggests that black carbon was mobilized from soils to streams. During 15-day biodegradation experiments, DOM from the two pasture streams was altered to a greater extent than DOM from the forest streams, with formulas with H/C and O/C ranges similar to protein (H/C = 1.5–2.2, O/C = 0.3–0.67), lipid (H/C = 1.5–2.0, O/C = 0–0.3), and unsaturated hydrocarbon (H/C = 0.7–1.5, O/C = 0–0.1) being the most bioreactive groups. Aromatic compound formulas including CAS were preferentially removed during combined light+bacterial incubations, supporting the contention that black carbon is labile to light alterations. Collectively, our data demonstrate that headwater DOM composition contains integrative information on watershed sources and processes, and the application of ESI-FTICR-MS technique offers additional insights into compound composition and reactivity unrevealed by fluorescence and stable carbon isotopic measurements.

## Introduction

Dissolved organic matter (DOM) in streams and rivers is derived from both watershed and aquatic contributions, containing information integrating various biological sources and ecological processes. As a complex mixture containing a multitude of components with varied composition and reactivity, DOM plays a pivotal role in a variety of biogeochemical processes within aquatic environments, including altering light regime, providing energy and substrate to heterotrophic food webs, and influencing the forms of metal pollutants. Historically, the chemical characteristics of natural DOM have been analyzed mostly through bulk methods, including element compositions (particularly DOC:DON molecular ratios), stable carbon isotopes of DOC (δ^13^C-DOC), and optical properties which can generate a series of source and reactivity indices based on fluorescence and absorption [1–4]. These techniques capture DOM as a whole but can be biased from averaging DOM constituents of various characteristics. Using such methods, molecular-level understanding of DOM remains limited because low-resolution instrumental approaches cannot separate and identify complex molecules in natural DOM.

Fourier transform ion cyclotron resonance mass spectrometry (FTICR-MS) analysis is increasingly recognized over the past decade as a powerful instrumental approach for characterizing DOM at the molecular level [5]. FTICR-MS provides unparalleled resolution for identification of ionized organic compounds, and it can be coupled with atmospheric pressure electrospray ionization (ESI) technique to ionize water-soluble, hydrophilic molecules with no or negligible fragmentation [6–8]. Unsurprisingly, ESI-FTICR-MS has been more frequently used to obtain more detailed and accurate compositional information on natural DOM. For example, Sleighter and Hatcher [9], using ESI-FTICR-MS, successfully resolved thousands of DOM components along a river-estuary-coastal ocean transect in lower Chesapeake Bay. Through comparing FTICR-MS molecular families to fluorescence components derived from excitation emission matrix-parallel factor analysis (EEM-PARAFAC) in 22 freshwater samples, Stubbins and colleagues [8] show that fluorescence components represented less than half of the total number of formulas identified using FTICR-MS, furthering demonstrating the need to apply this technique to various systems for acquiring more robust information about the diversity of natural DOM compounds.

A few recent studies have applied FTICR-MS to characterizing DOM in soils and natural waters across a range of ecosystems [10–11]. To date, few FTICR-MS studies have been focused on human-impacted watersheds, especially considering high spatiotemporal variability observed across geographic regions and ecosystem types. In particular, detailed, molecular-level chemical characterizations of DOM exported from grazing-impacted systems remain scarce, although grazed lands account for around 45% of non-Federal rural lands in US. In the present case study, we employed ESI-FTICR-MS to compare the composition of DOM from temperate headwater streams draining watersheds dominated by forest and pasture land use. Additionally, we conducted laboratory microbial and photochemical incubations to assess how DOM composition influences the biodegradability and photodegradability. The study sites included four temperate streams of similar lithological and meteorological variables, and samples were collected at base flow. This sampling strategy minimizes DOM variations related to geological, climatic, and hydrological differences but highlights those more due to watershed land use differences.

## Materials and Methods

### Sample collection, filtration and incubation

**Fig 1:**
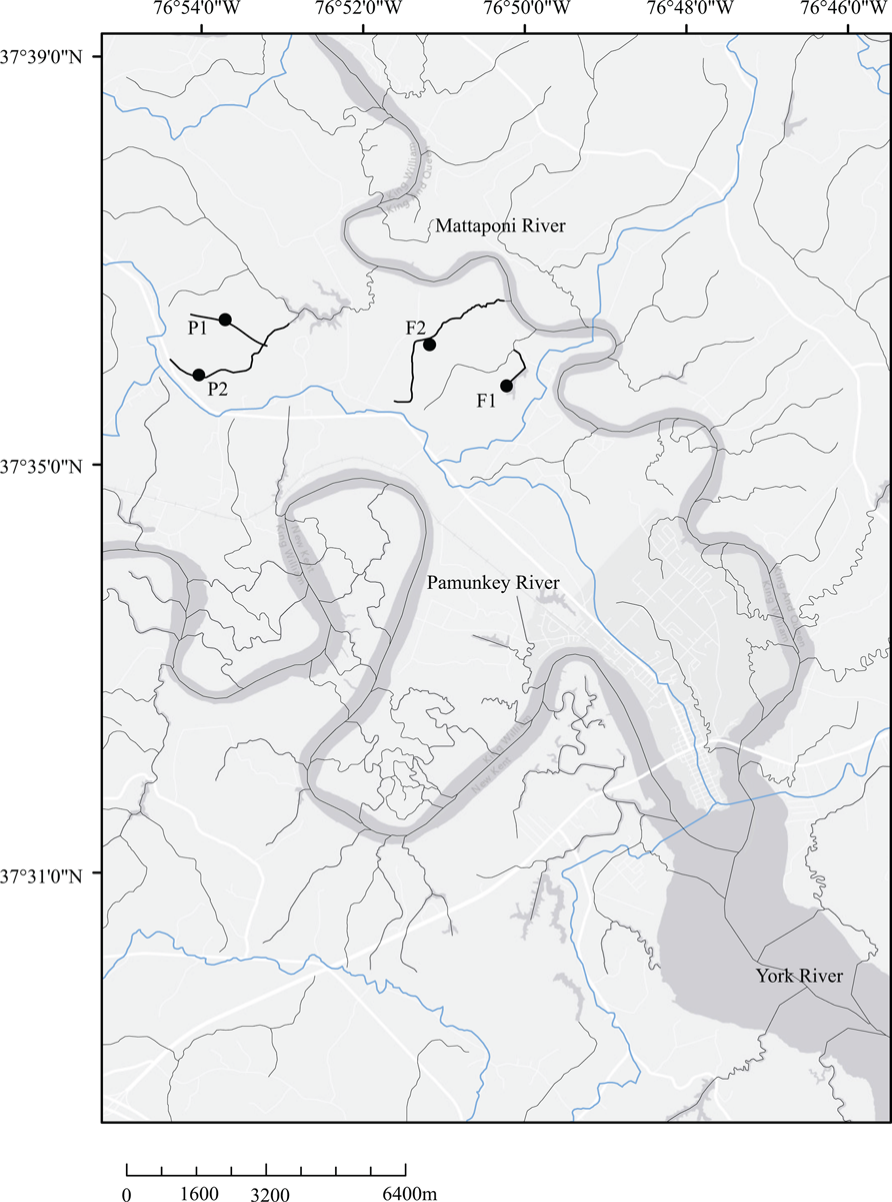
Map showing the study region and our sampling sites. Sampling streams are indicated by heavy black lines, and samples sites are indicated by solid black circles. Other streams in this area are denoted by gray lines, and the light blues lines are 12-digit HUC watershed boundaries.

Our study site included four headwater streams located 1–6 km apart, within the watershed of Mattaponi River, a tributary of the York River discharging to the Chesapeake Bay, Virginia, USA (Fig 1). Two of the streams (F1, F2) were situated within watersheds dominated by oak-pine forest (hereafter referred to as “forest streams”); the other two streams, P1 and P2, drained pasture-dominated watersheds (hereafter referred to as “pasture streams”), where pastures were rotated annually between warm-season grasses (May–October) and cool-season grasses (November–April) under the management of a local cattle farm. The study was carried out on private land, and the owners gave permission to conduct this study on the sites.

All streams were sampled during base flow conditions in November, 2009. During the sampling, a suite of *in situ* parameters was measured (Table 1). Stream water samples were collected in 20 l acid-cleaned polycarbonate carboys (soaked in 10% acid for > 24 h and rinsed extensively with Nanopure water) using a Masterflex® E/S™ portable sampler (Cole-Parmer) equipped with acid-cleaned silicone tubing. After collection, samples were stored on ice in the dark for up to 6 hours until being filtered in the lab. Living and non-living particulate materials were removed by filtering water through GF/F glass fiber filters (nominal pore size 0.7 μm, pre-combusted at 500°C for 5 h) [3]. Laboratory incubations were started immediately after the filtration process. All incubations lasted 15 days at 22±2°C, including two treatment types: 1) bacteria-only treatment (i.e., 0.7 μm filtrate under dark conditions) for all the samples, and 2) combined light+bacteria treatment (i.e., 0.7 μm filtrate under light conditions) only for samples from the pasture streams. Combined light+bacteria incubations were performed in 500 ml quartz flasks on a rotating light table, with the spectra of the light source having similar characteristics to that of natural sunlight for UV wavelengths between 295 and 365 nm. The irradiance was approximately one third of seasonally averaged daily solar irradiance in shallow water at 40°N [12], and thus the amount of UV exposure during the 15 days of 24 h light incubations was similar to 10 days of 12 h daylight at the sampling sites. Dark incubations were conducted in one-liter amber borosilicate glass bottles, which were covered by dark bags to further prevent light penetration. Throughout the incubations, subsamples were collected at the beginning and end of the incubations (T_0_ and T_15_, respectively) as well as four to six intermediate time points for the evaluation of DOC concentrations [3]. ESI-FTICR-MS analyses were performed on T_0_ and T_15_ samples.

**Table 1:** Watershed land use, environmental parameters, and water parameters measured for the streams sampled in this study*.

Sampling site	Watershed land use composition	Water temperature (°C)	Specific conductivity (μS)	pH	Dissolved oxygen (mg/L)	DOC concentration (μM)	Water column chlorophyll-a (μg/L)	Nitrate (mg/L)	Ammonium (mg/L)	Sulfate (mg/L)
F1	100% forest	12.6	158.9	5	3.0	539	0.01	b.d.	b.d.	37.9
F2	100% forest	12.6	56.9	5	7.8	562	0.05	b.d.	b.d.	8.8
P1	70% pasture, 30% forest	12.6	210.4	6–7	7.9	675	0.14	2.29	2.49	14.4
P2	61% pasture, 39% forest	16.5	73.4	5	5.8	206	0.48	b.d.	b.d.	1.1

*b.d. = below detection; nitrate, ammonium, and sulfate were measured by using a Dionex ion chromatography.

### ESI-FTICR-MS analysis

ESI-FTICR-MS analysis was performed at the College of Science Major Instrumentation Cluster (COSMIC) Lab, Old Dominion University (Virginia, USA). The C_18_ extraction method was used to concentrate DOM, which selectively retains non-polar, low-molecular-weight DOM (<700Da) [13]. Solid phase C_18_ extraction disks (3M, Empore™, 47 mm diameter) were activated by LC-MS grade water and methanol before use. Sample waters were acidified to pH = 2 before passing through C_18_ disks under vacuum. Water and methanol (both LC-MS grade) were used sequentially to elute materials retained on the C_18_ disks. The eluates were collected, diluted with LC-MS grade water to a ratio of 50:50 (v/v) methanol:water for each sample. Ammonium hydroxide was then added to raise the sample pH to 8 in order to increase ionization efficiency [9].

All samples were continuously infused into an Apollo II ESI ion source of a Bruker Daltonics 12 Tesla Apex Qe FTICR-MS, using a syringe pump providing an infusion rate of 120μL/h. A solution of 0.1% ammonium hydroxide in 50:50 (v/v) LC-MS grade methanol:water was analyzed between each sample, serving as a blank to check for sample cross-contamination [14]. The samples and blanks were analyzed in negative ion mode, and electrospray voltages were made effective for each sample. Ions accumulated in a hexapole for 1.0 s before traveling to the ion cyclotron resonance cell. 300 or 350 transients were applied, yielding 4 mega word time-domain data for a total run time of 30 or 35 minutes, respectively. The summed free induction decay signal was zero-filled once and Sine-Bell apodized prior to fast Fourier transformation and magnitude calculation using the Bruker Daltonics Data Analysis software.

### Formula assignments and data visualization and interpretation

All masses were internally calibrated following the calibration method described by Sleighter et al. [15], using fatty acid naturally present within our samples and other homologous peak series identified by Kendrick mass defect (KMD) analysis, which spanned the entire mass range of 200–700 m/z. Following the recommendation for data reproducibility [16], m/z values with signal to noise ratios ≥ 4 were considered for formula assignment. A molecular formula calculator developed at the FTICR-MS Facility at the National High Magnetic Field Laboratory of Florida State University (Molecular Formula Calc v.1.0 NHMFL, 1998) was used to generate empirical formulas. The range of the number of different atoms for each formula was set as 1–50 for carbon, 2–100 for hydrogen, 0–30 for oxygen, 0–6 for nitrogen, and 0–2 for sulfur.

Data processing followed the method described in detail by Sleighter and Hatcher [9] and Stubbins et al. [14]. Briefly, we selected chloride-free peaks whose measured mass and exact mass of empirical formulas were within ±1 ppm, and then eliminated those empirical formulas that did not comply with the basic bonding criteria of organic compounds[7, 14, 17]: 1) O/C ≤ 1.2; 2) 0.35 ≤ H/C ≤ 2.25; 3) N/C ≤ 0.5; 4) S/C ≤ 0.2; 5) nitrogen rule (i.e., odd mass weight containing even-electron N ions, while even mass weight containing odd-electron N ions); and 6) Double Bond Equivalent (DBE) value being an integer ≥ 0. The DBE is defined as:

DBE=(2+2×C−H+N+P)÷2 (1)

where C, H, N and P represent the number of carbon, hydrogen, nitrogen, and phosphorous atoms in molecules, respectively. When multiple assignments were possible for a single m/z value, the formula was assigned based on homologous series [15, 18–19].

**Fig 2:**
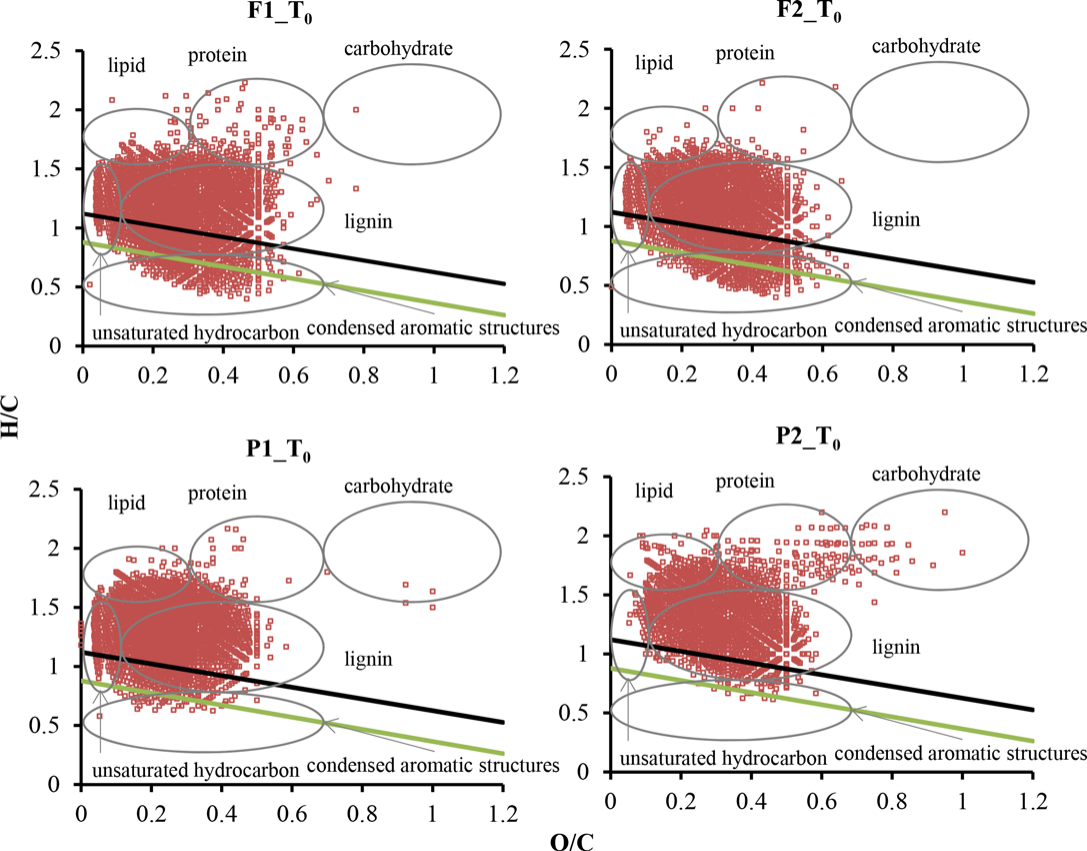
Van Krevelen diagrams of DOM at T_0_. The light green solid line denotes the regression line through the minimum H/C and O/C values for formulas with AI_mod_ = 0.5 (i.e., the majority of aromatic molecules are plotted below the line) and the black solid line denotes the regression line for formulas with AI_mod_ = 0.67 (i.e., the majority of CAS molecules are below the line).

Van Krevelen (VK) analysis classifies the DOM molecules into different biochemical classes based on their H/C and O/C atomic ratios [20–21]. Following the classification described by Hockaday et al. and Ohno et al. [22–24], VK diagrams can be classified into six regions: 1) lipid-like region (H/C = 1.5–2.0, O/C = 0–0.3); 2) protein-like region (H/C = 1.5–2.2, O/C = 0.3–0.67); 3) lignin-like region (H/C = 0.7–1.5, O/C = 0.1–0.67); 4) carbohydrate-like region (H/C = 1.5–2.4, O/C = 0.67–1.2); 5) unsaturated hydrocarbon-like region (H/C = 0.7–1.5, O/C = 0–0.1); and 6) condensed aromatic ring structure (CAS) region (H/C = 0.2–0.7,O/C = 0–0.67) (Fig 2).

Additionally, several indices were used to classify DOM molecules with respect to their possible function groups. The modified aromaticity index (AI_mod_), which assumes half of Os participating in a double bond, was used to identify aromatic formulas (AI_mod_ between 0.5 and 0.67) and CAS (AI_mod_ > 0.67, a more conservative way to define CAS than VK classification as described above) [25]:

$$AI_{\text{mod}}=\frac{1+C-0.5O-S-0.5H}{C-0.5O-S-N-P}$$ (2)

Formulas with AI_mod_ < 0.5 were classified as aliphatic and olefinic compounds such as alkanes, alkenes, alkanoic acids, alenoic acids, alkanals, alkenals, and terpens [26].

### Statistical analysis

Jaccard similarity coefficients, which have been shown to be a powerful approach to compare FTICR-MS formulas across samples [27], were calculated to analyze formula similarity between different samples based on compound presence/absence, where coefficient = 1 indicates that two samples share the same formulas while coefficient = 0 indicates two samples having no formulas in common. Additionally, nonmetric multidimensional scaling (NMDS) was used to discern samples’ similarities and dissimilarities. The imputing matrix was Bray-Curtis distance calculated according to compounds’ presence/absence. This method has also been shown efficient in grouping samples in FTICR-MS studies [11, 28]. Reliability analysis was performed prior to NMDS (Cronbach’s Alpha = 0.7; the two samples from the combined light+bacteria incubations were removed to increase the value of Cronbach’s Alpha), and three-dimension solution was used (Kruskal’ stress = 0.01).

## Results and Discussion

### DOM formulas in T_0_ samples

**Fig 3:**
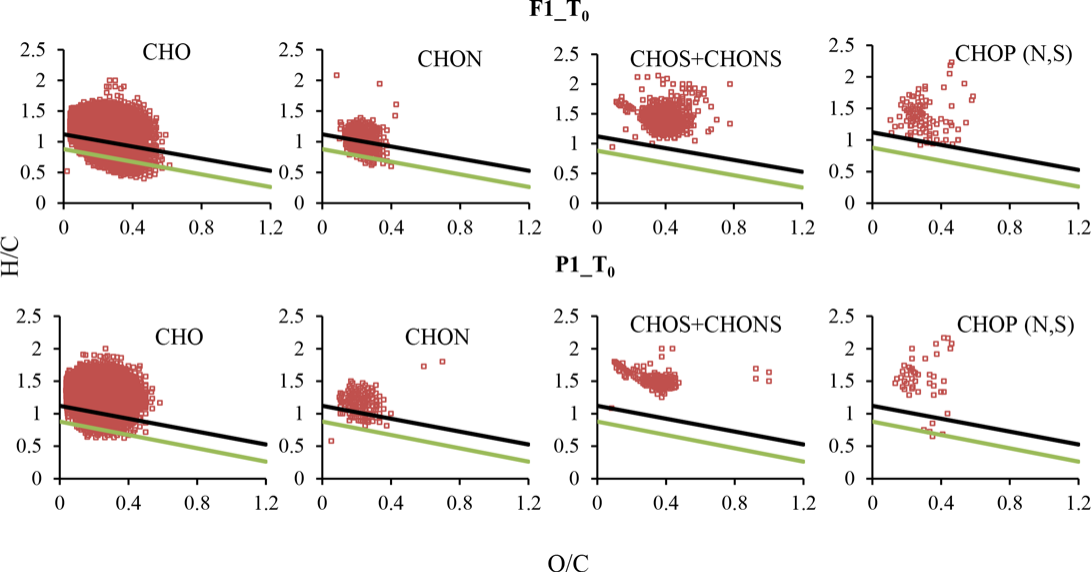
Van Krevelen diagrams for molecular series in F1 and P1 at T_0_. The light green solid line denotes the regression line through the minimum H/C and O/C values for formulas with AI_mod_ = 0.5 (i.e., the majority of aromatic molecules are plotted below the line) and the black solid line denotes the regression line for formulas with AI_mod_ = 0.67 (i.e., the majority of CAS molecules are below the line).

For T_0_ samples, 1936–3083 compound peaks were assigned with molecular formulas, accounting for 64.5–70.8% of total peaks detected (Table 2). In all samples, CHO series dominated the formulas (71.8–87.9%), and other series made up minor fractions: CHOS (3.0–14.0%), CHON (2.9–9.2%), CHONS (0.2–2.0%), and CHOP (N,S) (1.4–6.3%) (Fig 3, S1 Appendix). The dominance of CHO molecular series has also been observed in other streams and rivers [11, 28]. The majority of compounds were aliphatic or olefinic (Table 2, Fig 2). Based on H/C and O/C ratios, 69.2% to 79.0% of total formulas can be classified as lignin (S3 Appendix), although these numbers were perhaps overestimated because many formulas fell in the aliphatic and olefinic region based on AI_mod_ (Fig 2), which is a more conservative method to identify aromatic molecules. The large abundance of lignin-like formulas agrees with the previous EEM-PARAFAC data on these samples showing the dominance of terrestrially-derived, humic and fulvic-like compounds in DOM [3], i.e., C10 and SQ1 peaks in the 13-component model [29]. Lignin is found in the cell wall of vascular plants and is a major residual component of decomposed terrestrial plants [30–32]. Our observation supports the view that allochthonous inputs of OM serve as the primary source of food and energy to food webs in low-order streams [4, 33–34].

**Table 2:** FTICR-MS parameters of the forest and pasture stream DOM samples at T_0_ and T_15_.

Sample and Incubation Type	Time Point	Degradable DOC (%)*	#peaks detected^$^	#peaks with assigned formulas	Formulas in the CAS region	AI_mod_ between 0.5 and 0.67	AI_mod_ >0.67	(DBE)_*m*_ ^$^	(O/C)_*m*_ ^*$*^	(H/C)_*m*_ ^*$*^	Refractory formulas
F1 Bacteria-only	T_0_	13.2	4357	3083	206 (6.7%)	767 (24.9%)	146 (4.7%)	11.01	0.30	1.17	85.7%
	T_15_		4313	3063	201 (6.6%)	616 (20.1%)	140 (4.6%)	11.17	0.30	1.17	
F2 Bacteria-only	T_0_	15.0	3704	2576	251 (9.7%)	686 (26.6%)	149 (5.78%)	11.65	0.31	1.13	79.0%
	T_15_		3681	2576	208 (8.1%)	463 (18.0%)	122 (4.74%)	10.94	0.34	1.14	
P1 Bacteria-only	T_0_	1.9	3878	2502	22 (0.9%)	291 (11.6%)	15 (0.60%)	9.74	0.26	1.30	32.0%
	T_15_		1978	1386	5 (0.4%)	36 (2.60%)	6 (0.43%)	8.40	0.37	1.34	
P2 Bacteria-only	T_0_	23.9	2746	1936	11 (0.6%)	146 (7.54%)	6 (0.31%)	8.79	0.31	1.34	69.7%
	T_15_		3403	2449	110 (4.5%)	339 (13.8%)	43 (1.76%)	9.91	0.34	1.23	
P1 Light + Bacteria	T_0_	15.9	3878	2502	22 (0.9%)	291 (11.6%)	15 (0.60%)	9.74	0.26	1.30	26.9%
	T_15_		1007	692	0 (0%)	5 (0.72%)	0 (0%)	7.61	0.30	1.39	
P2 Light + Bacteria	T_0_	50.0	2746	1936	11 (0.6%)	146 (7.54%)	6 (0.31%)	8.79	0.32	1.34	63.1%
	T_15_		2999	1916	0 (0%)	36 (1.88%)	0 (0%)	8.54	0.37	1.35	

* data from [3]

^$^ m/z in the range of 200–700; s/n ≥ 4; ratios (O/C)_*m*_, (H/C)_*m*_, and DBE_*m*_ were mean values from all peaks with assigned molecular formulas.

Aromatic compounds including CAS were concentrated in the CHO and CHON molecular series, while S and P-containing compounds were mostly olefinic or aliphatic (Fig 3). For CHON group, many formulas were not plotted in the protein-like region (i.e., H/C = 1.5–2.2, O/C = 0.3–0.67). A large majority (85–98%) of sulfur-containing formulas had O/S values ≥4, perhaps suggesting that these formulas represented organosulfates. Comparing the distribution of molecular series across the T_0_ samples, the only noticeable pattern was that F1 had a higher proportion and larger number of S-containing compounds than other samples (16% in F1 *vs*. 3.4–7.1% in other three samples; also see S1 Appendix), which could be due to a much higher sulfate concentration in this stream (37.9 mg/L in F1 *vs*. 1.1–14.4 mg/L in other streams, Table 1) that facilitated the formation of organosulfates.

**Fig 4:**
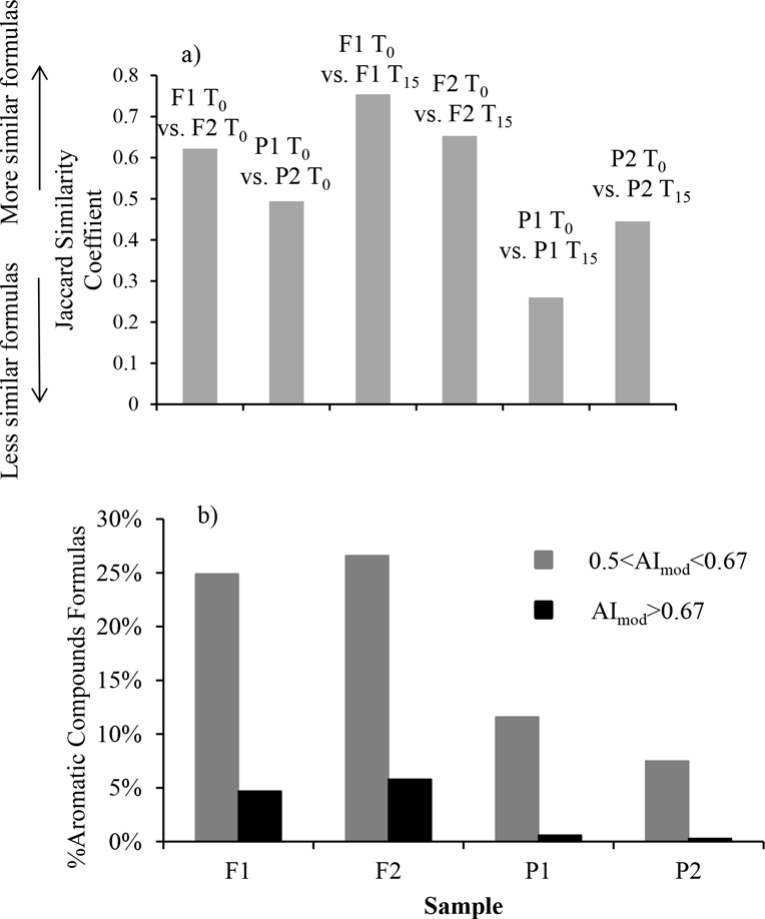
a) Jaccard similarity coefficients of DOM formulas between different samples or incubation time points; b) Relative abundance (%total formulas) of aromatic formulas in T_0_ samples.

**Fig 5:**
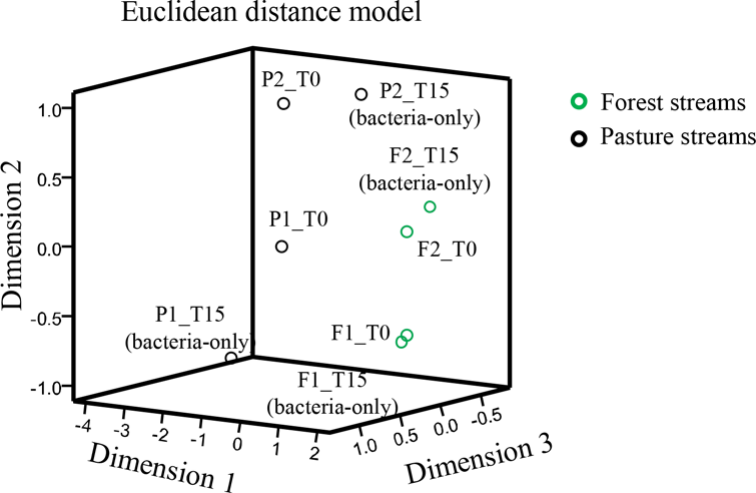
Nonmetric multidimensional scaling (NMDS) plot based on Bray-Curtis distances among the samples.

Based on the Jaccard similarity coefficients of DOM formulas, the two forest stream samples (F1 and F2) were more similar than the two pasture stream samples (P1 and P2) (Fig 4). This observation was also echoed by the NMDS based on Bray-Curtis distance (Fig 5), where samples from the forest streams were plotted tightly into a small region, relative to more widespread samples from the pasture streams. DOM at T_0_ from the pasture streams was characterized by lower mean DBE values (11.01 and 11.65 for the forest streams *vs*. 9.74 and 8.79 for the pasture streams), higher H/C values (1.17 and 1.13 for the forest streams *vs*. 1.30 and 1.34 for the pasture streams), and comparable O/C values (Table 2), indicating greater saturation for the formulas in the pasture streams. These patterns are in agreement with the results from VK classification and AI_mod_ analysis, showing DOM at T_0_ from the pasture streams had lower numbers and percentages of aromatic formulas (Table 2, Fig 4). In addition to greater contributions of high-molecular-weight, aromatic compounds derived from land plants in the forest streams, these characteristics may be also related to light penetration, which was greater to the pasture streams due to removal of riparian trees in some sections of the pasture streams (*personal observation*). Light preferentially alters and remineralizes aromatic carbons with high DBE values [2, 14, 35].

FTICR-MS data cannot be used directly to derive information about compound abundance because of varied ionization efficiencies across compounds. Nevertheless, we found that the relative abundance of formulas in our samples largely agrees with the EEM-PARAFAC data from a companion study by Lu and colleagues [3]. EEM offers a relatively low resolution in compound separation and identification, but it is viewed as a technique capable of offering DOM compositional information in a quantitative manner [36, 37]. The higher percentages of aromatic formulas in F1 and F2 than in P1 and P2 (Table 2, Fig 4B) agree with the EEM data showing that DOM from the forest streams had higher percent contributions of terrestrial fulvic and humic-like compounds than DOM from the pasture streams. Additionally, the EEM data showed that the percentages of protein-like DOM were lower in the forest streams, which was interpreted as evidence supporting that human activities likely increased autochthonous contributions of DOM. Similar patterns have been noted in many EEM studies of DOM in human-impacted streams and rivers [1, 2, 38]. However, this information cannot be derived from the FTICR-MS data. Although the relative abundance of formulas falling in the protein region (i.e., H/C = 1.5–2.2, O/C = 0.3–0.67) was higher in the pasture streams than in the forest streams (S3 Appendix), a large majority of these compounds did not contain N and cannot be classified as protein (Fig 3, S1 Appendix). Roth et al. [11] also reported that N-containing formulas were relatively lacking in surface water in comparison to soil water in a study employing the FTICR-MS technique, and explained this observation as a result of active microbial processing of N-containing compounds. Several reasons could account for the divergent observations from FTICR-MS and EEM measurements: 1) low retention of protein compounds by C_18_ filters, which was used for solid phase extraction prior to FTICR-MS analysis; and 2) possible overestimates of protein contribution by EEM measurements, on the basis that low molecular weight, N-free compounds such as gallic acid could also contribute to protein fluorescence peaks [39]. Divergent observations from FTICR-MS and EEM characterizations have also been recently noted by Stubbins et al. [14], who found that of the FTICR-MS formulas correlating to the classical protein-fluorescence peak in EEM-PARAFAC, only 31% contained N.

More CAS formulas were found in the forest streams than in the pasture streams (Table 2, Fig 2), based on both VK classification and AI_mod_ index. Black carbon, or charcoal, is an important source of molecules with CAS [22, 40–43], and they are produced by incomplete combustion of biomass and fossil fuels [44]. Therefore, the black carbon in DOM form the forest streams may be a result of controlled fire regularly used in the forested watersheds to suppress fuel buildup. This observation supports a recent contention that black carbon can be mobilized through dissolution and subsequently discharged to adjacent open water [43, 45].

### Bacterial alterations to DOM compound formulas

In T_15_ compounds after the bacteria only-incubations, the relative abundance of molecular series exhibited distributions similar to T_0_ samples, while no consistent pattern was observed among the samples with respect to changes in the relative abundance of molecular series (S1 Appendix). CHO series were still the most abundant group (73.7–84.6%) and the majority CHOS compounds (84.5–98.3%) had O/S ≥ 4, indicating the presence of organosulfates. Furthermore, no consistent changes were observed in the relative proportions of aromatic compounds based on the AI_mod_ index analysis, despite that aromatic compounds were commonly viewed as being more refractory to bacterial degradation [46]. F1, F2, and P1 showed a decrease in %aromatic formulas, while P2 showed an increase in %aromatic formulas (Table 2).

Two compound groups were evaluated by comparing DOM prior to and after the bacteria-only incubations: 1) biorefractory group that did not change during the incubations (i.e., present at T_0_ and T_15_); 2) bioreactive group that existed only at T_0_ because they were remineralized or altered over the course of the incubations. Overall, DOM from the pasture streams had lower proportions of biorefractory formulas but higher proportions of bioreactive formulas than DOM from the forest streams (Table 2). This pattern agrees with the results from Jaccard similarity coefficient and NMDS analysis (Figs 4 and 5). The Jaccard similarity coefficients between T_0_ and T_15_ samples were greater for DOM from the forest streams than from the pasture streams (Fig 4), and T_0_ versus T_15_ samples were separated by smaller distances for the forest streams than for the pasture streams in the NMDS plot (Fig 5). Furthermore, the number of formulas was reduced by less than 1% for the two forest samples but changed by more than 26% for the two pasture samples (Table 2). These observations all show that the molecular compositions of the two pasture streams were altered to a greater extent than the two forest streams during the bacteria-only, dark incubations, with the caveat that P1 was different from the other three samples by demonstrating a large decrease in the number of compounds after the incubation. This decrease may be related more to higher nutrient concentrations in this sample (Table 1) than to DOM composition itself in stimulating microbial activity.

**Fig 6:**
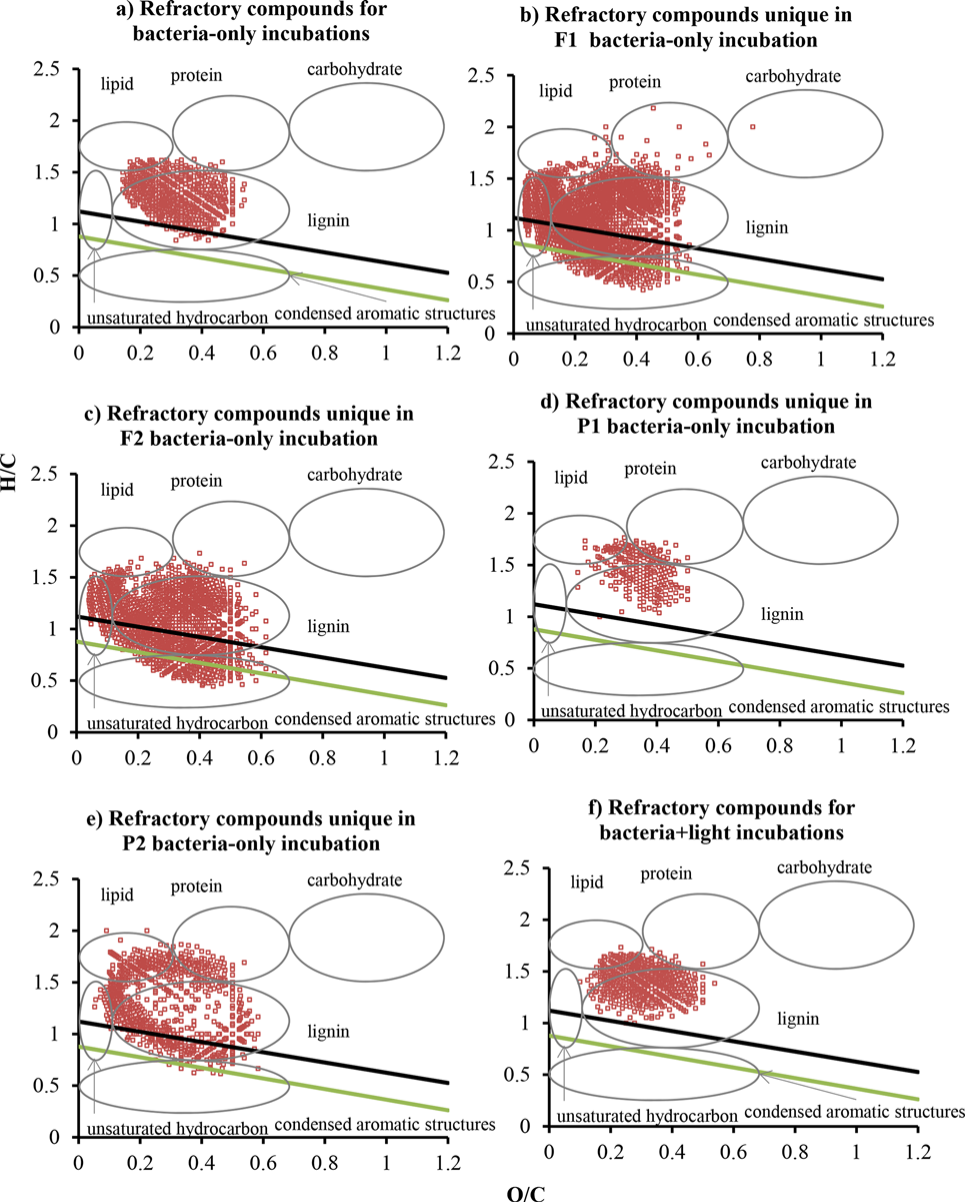
Van Krevelen diagrams of refractory compounds a) ubiquitous for all samples in bacteria-only incubations; b) unique in F1 bacteria-only incubation; c) unique in F2 bacteria-only incubation; d) unique in P1 bacteria-only incubation; e) unique in P1 bacteria-only incubation; and f) ubiquitous for all samples in combined bacteria+light incubations. The light green solid line denotes the regression line through the minimum H/C and O/C values for formulas with AI_mod_ = 0.5 (i.e., the majority of aromatic molecules are plotted below the line) and the black solid line denotes the regression line for formulas with AI_mod_ = 0.67 (i.e., the majority of CAS molecules are below the line).

**Fig 7:**
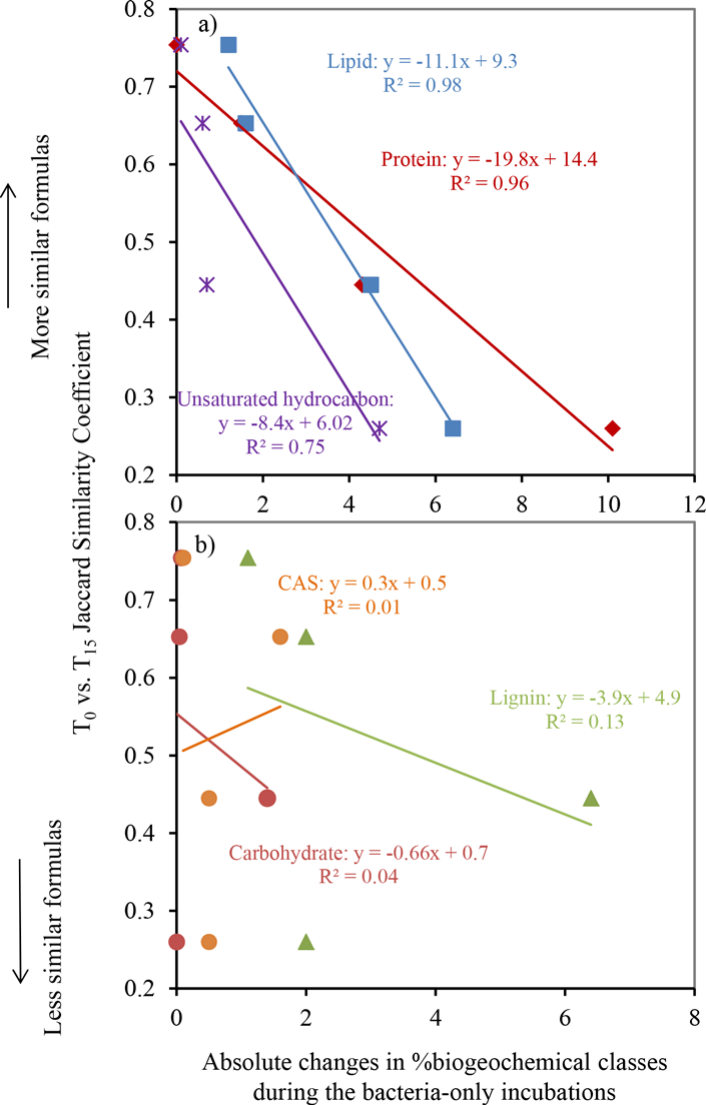
Correlations between Jaccard similarity coefficients and absolute changes in the proportions of the five Krevelen classified groups in the bacteria-only incubations. Solid lines represent linear trend lines.

There were 567 compounds refractory to bacteria-only incubations (i.e., present in T_0_ and T_15_ of all four samples, Fig 6A). These refractory compounds had H/C values ranging between 0.8 and 1.6 and O/C between 0.1 and 0.5, comparable to the ranges reported for formulas ubiquitously present across a range of ecosystems (river, forest, grassland, and bog), which were around 0.9–1.5 for H/C and 0.2–0.7 for O/C [11]. Biorefractory compounds unique to each sample varied from 235 and 2084, and they displayed larger ranges in both H/C and O/C ratios (Fig 6). Interestingly, the changes in the proportions of compounds falling in the protein-like and lipid-like regions showed the best correlation with the Jaccard similarity coefficients between T_0_ versus T_15_ samples for the bacteria-only incubations (Fig 7), indicating that these compounds were primarily responsible for the changes in DOM molecules under the influences of bacterial processing. This is consistent with overall lower proportions of formulas in lipid-like and protein-like regions in biorefractory group (8.0±5.2% for lipid-like and 6.8±3.7% for protein-like formulas) than in bioreactive group (12.4±3.6% for lipid-like and 12.3±7.9% for protein-like formulas). Although the lability of proteins and lipids to microbial utilization has been widely reported in aquatic environments and soil extracts [47–50], the formulas identified by VK classification are not necessarily proteins or lipids. Instead, our observation argues for the lability of compounds with elemental makeup similar to proteins or lipids. Similarly, the H/C and O/C ranges of those resistant, ubiquitous compounds in various ecosystems reported by Roth et al. [11] did not include protein-like or lipid-like regions in VK diagrams. Furthermore, although a number of studies have noted a positive correlation between %biodegradable DOC versus %protein fluorescence [51–54], this correlation was not found in our study streams [3]. The FTICR-MS data here may provide an explanation—other formulas with high H/C ratios (>1.5) including those in lipid-like and unsaturated hydrocarbon-like regions also exhibited a positive correlation with the Jaccard similarity coefficients, indicating that they were also bioreactive and can play an important part in determining DOM biodegradability (Fig 7). This observation agrees with the previous finding that DOM with high H/C values was a labile source for bacteria, on the basis that H/C was a positive predictor of bacterial production in the Ogeechee River system [55]. Formulas in the carbohydrate-like region, however, did not show changes corresponding to changes in DOM formula similarity. This observation, rather than suggesting the refractory nature of carbohydrate-like compounds, is more likely a result of poor representation of carbohydrates in FT-ICRMS data related to their inefficient ionization in negative-mode ESI [14] and/or the selective retention of non-polar compounds through C_18_ filters [13].

### Combined light + bacteria alterations to DOM compound formulas

The two pasture stream samples were incubated under combined light and bacteria incubations. In both samples, the proportions of aromatic formulas and DBE decreased, and O/C values increased (Table 2). These characteristics are typical for light-induced DOM alterations, where the addition of oxygen to DOM during photochemical oxidation may be a result of ionization yielding hydrated electrons and organic radicals that both can react with molecular oxygen [35]. P1 and P2 shared 480 refractory compounds (present at both T_0_ and T_15_ of combined light+bacteria incubations) which had H/C values varying from 1 to 1.7 and O/C values from 0.1 to 0.5 and comprised mostly aliphatic compounds (Fig 6). Relative to biorefractory compounds, they had higher values of H/C and lower values of AI_mod_. Correspondingly, the relative percentages and absolute numbers of formulas falling in the region for unsaturated hydrocarbons were higher in reactive formulas (present only in T_0_ of the combined incubations) than in refractory formulas—7.1±0.6% of reactive formulas versus 0.5±0.9% of refractory formulas. These observations confirmed the sensitivity of unsaturated bonds (e.g., C = C, benzene rings, conjugate systems) to light alterations [14, 56–58]. Similar observations have been made for riverine and estuarine waters. For example, Kujawinski et al. [57] irradiated DOM samples from the Suwannee river with light of wavelengths >305 and >360 nm and found that DOM formulas with high DBE and low oxygen content were generally destroyed, resulting in lower unsaturation and higher oxygen content in residual DOM. Notably, all CAS formulas were removed after the combined bacteria+light incubations (Table 2), supporting the recent notion that black carbon mobilized from soil to water can be further altered by light [14, 22, 42, 45], as opposed to the conventional view that black carbon was inert in biogeochemical cycles due to the abundant content of aromatic and graphitic C [59–61]. In our experiments, the decomposition occurred within days, consistent with the previous finding that all black carbon–like formulas disappeared after 57 d photochemical incubations of Congo River DOM [14].

### DOM similarity and diversity

The degree of alterations in DOM molecules indicated by the Jaccard similarity coefficient or Bray-Curtis distance did not correlate to %degradable DOC (Pearson r≤0.03, *P*≥0.94), indicating that heterotrophic microbes may be actively transforming DOM formulas without removing them through remineralization. This is not surprising as the similarity measures assess only compositional information (compound presence/absence) that does not always correspond to DOC quantity. Therefore, when interpreting DOM reactivity data from previous literature, one should keep in mind that degradable DOC, a widely used proxy to estimate DOM lability to microbes within lotic ecosystems [3, 62], may not always be an accurate proxy indicative of the actual roles of DOM in supporting microbial food webs. Likewise, Herlemann and colleagues [63] showed a decoupling between DOC concentration and DOM molecular composition, i.e., 4–10% DOC loss versus no significant changes in DOM molecular composition during 28-day incubation experiments.

**Fig 8:**
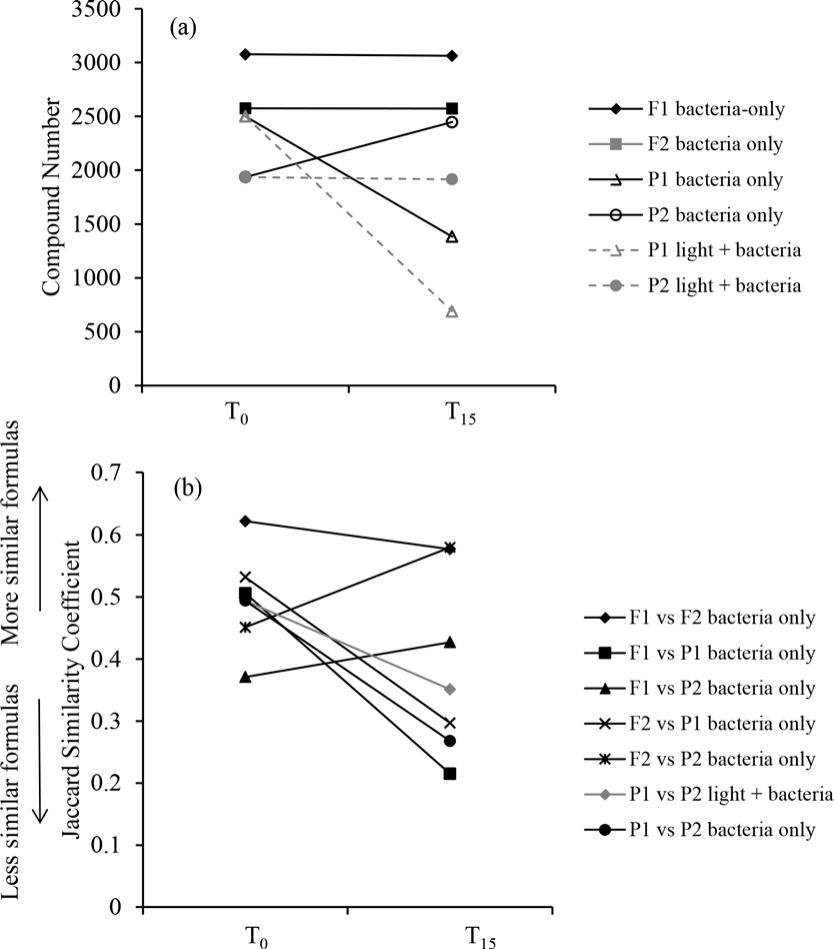
Comparing DOM diversity prior to versus after the incubations through a) compound number, and b) DOM formula similarities indicated by Jaccard similarity coefficients between T_0_ vs. T_15_ samples.

We combined the number and similarity of DOM molecules to indicate DOM diversity. After the incubation experiments, the number of formulas decreased in all but one sample, but the majority samples showed decreases in similarity coefficients (Fig 8). In agreement with this pattern, T_15_ samples spread more widely than T_0_ samples in the NMDS plot (Fig 5), indicating that light and/or bacterial processing led to more dissimilar DOM formulas. This is in contrast to the previous notion that DOM compounds become more uniform with increasing degrees of processing by light and bacteria, which was based primarily on the observation that photochemical and microbial processing of DOM tends to leave behind compounds with similar optical properties and carbon isotopic compositions across various systems (e.g., stream, river, and coastal waters) and sample types (e.g., surface water and soil solutions) [3, 35, 48]. Our observations based on the FTICR-MS data remain to be verified and are unable to lead to generalizations on across-system patterns, largely due to the non-quantitative nature of this technique as well as the small sample size, which is a general feature for FTICR-MS studies owing to arduous efforts and expensive instruments involved. Nevertheless, these observations demonstrate the need to compare and synthesize high-resolution molecular data with isotopic and optical measurements to yield more reliable understanding on molecular transformations in lotic ecosystems.

## Conclusions

Through the use of ESI-FTICR-MS, we characterized DOM from headwater streams draining pasture-dominated and forest-dominated watersheds, considering both molecular composition and molecular transformation under the influence of microbial and photochemical processes. In all the samples, the majority of the formulas fell within the lignin-like region, supporting the dominance of allochthonous DOM in headwaters. Formulas in the two forest streams were characterized by higher unsaturation and greater aromaticity than those in the two pasture streams, suggesting larger contributions of terrestrial plant-derived DOM in the forest streams. Larger numbers and percentages of CAS formulas were observed in the forest streams, indicating that soil black carbon resulting from controlled fires within the forest-dominated watersheds can be dissolved and discharged to open water. Bacterial processing altered DOM from the pasture streams to a greater extent than DOM from the forest streams, where formulas falling in the H/C and O/C ranges for protein-like, lipid-like, and unsaturated hydrocarbon-like regions were primarily responsible for DOM molecular alterations. During the combined light + bacteria incubations, more unsaturated compounds were preferentially degraded, and all CAS molecules were removed, supporting the liability of black carbon to photochemical alterations. Based on compound number and formula similarities, light and bacterial processing appeared to increase DOM formula diversity after the 15 d experiments. Overall, the use of FTICR-MS technique provided additional information unrevealed by previous measurements of DOC concentration, δ^13^C-DOC, and fluorescence properties, demonstrating the importance of incorporating this sophisticated technique to identify qualitative changes in the sources and fates of terrestrially derived DOM in aquatic systems. However, interpreting FTICR-MS data from the perspective of extrapolating compound-level information to whole-DOM compositional information has to rely closely on other more quantitative measures such as fluorescence characterizations.

## Supporting Information

S1 Appendix The abundance and elemental ratio of various molecular series in each sample. (DOCX)

S2 Appendix Van Krevelen diagrams of refractory compounds unique for P1 and P2 in bacteria+light incubations (PDF)

S3 Appendix FTICR-MS parameters of the forest and pasture stream DOM samples at T_0_ and T_15_. (DOCX)

S4 Appendix Parameters for peaks with assigned formulas in T_0_ samples. (DOCX)

S5 Appendix Parameters for peaks with assigned formulas in T_15_ samples (sample number continued from S4 Appendix). (DOCX)
