# Supplementary material for: Use of ESI-FTICR-MS to Characterize Dissolved Organic Matter in Headwater Streams Draining Forest-Dominated and Pasture-Dominated Watersheds
Source: PLoS One. 2015 Dec 29;10(12):e0145639. doi: 10.1371/journal.pone.0145639 (PMC4694922; doi:10.1371/journal.pone.0145639)
Supplement: S1 Appendix — (DOCX) [file pone.0145639.s001.docx]

S1 Appendix I: The abundance and elemental ratio of various molecular series in each sample.

| Sample | Elemental composition | Number of formulas | Percentages of formulas | O/C (mean±SD*) | H/C (mean±SD*) |
| --- | --- | --- | --- | --- | --- |
| F1 Bacteria-only, T_0_ | CHO | 2214 | 71.8 | 0.28±0.12 | 1.12±0.28 |
|  | CHON | 284 | 9.2 | 024±0.06 | 1.03±0.19 |
|  | CHOS+CHONS | 493 | 16.0 | 0.40±0.10 | 1.44±0.19 |
|  | CHOP+CHONP+CHOSP | 92 | 3.0 | 0.30±0.09 | 1.32±0.24 |
| F1 Bacteria-only, T_15_ | CHO | 2258 | 73.7 | 0.28±0.12 | 1.12±0.28 |
|  | CHON | 270 | 8.8 | 0.24±0.06 | 1.03±0.17 |
|  | CHOS+CHONS | 449 | 14.7 | 0.41±0.09 | 1.43±0.19 |
|  | CHOP+CHONP+CHOSP | 86 | 2.8 | 0.29±0.07 | 1.38±0.24 |
| F2 Bacteria-only, T_0_ | CHO | 2265 | 87.9 | 0.30±0.12 | 1.10±0.29 |
|  | CHON | 92 | 3.6 | 0.25±0.06 | 1.08±0.17 |
|  | CHOS+CHONS | 184 | 7.1 | 0.38±0.07 | 1.42±0.12 |
|  | CHOP+CHONP+CHOSP | 37 | 1.4 | 0.35±0.13 | 1.51±0.29 |
| F2 Bacteria-only, T_15_ | CHO | 2126 | 82.5 | 0.33±0.13 | 1.12±0.28 |
|  | CHON | 200 | 7.8 | 0.30±0.07 | 1.11±0.16 |
|  | CHOS+CHONS | 227 | 8.8 | 0.45±0.10 | 1.39±0.19 |
|  | CHOP+CHONP+CHOSP | 23 | 0.9 | 0.36±0.11 | 1.41±0.30 |
| P1 Bacteria-only, T_0_ | CHO | 2175 | 86.8 | 0.26±0.11 | 1.29±0.25 |
|  | CHON | 141 | 5.6 | 0.23±0.08 | 1.15±0.18 |
|  | CHOS+CHONS | 141 | 5.6 | 0.33±0.15 | 1.54±0.14 |
|  | CHOP+CHONP+CHOSP | 45 | 1.8 | 0.29±0.09 | 1.51±0.37 |
| P1 Bacteria-only, T_15_ | CHO | 1058 | 76.3 | 0.36±0.13 | 1.30±0.22 |
|  | CHON | 61 | 4.4 | 0.42±0.14 | 1.33±0.21 |
|  | CHOS+CHONS | 11 | 0.8 | 0.29±0.17 | 1.50±0.32 |
|  | CHOP+CHONP+CHOSP | 256 | 18.5 | 0.39±0.09 | 1.53±0.15 |
| P2 Bacteria-only, T_0_ | CHO | 1692 | 87.4 | 0.31±0.11 | 1.30±0.27 |
|  | CHON | 56 | 2.9 | 0.30±0.10 | 1.16±0.21 |
|  | CHOS+CHONS | 65 | 3.4 | 0.31±0.14 | 1.60±0.16 |
|  | CHOP+CHONP+CHOSP | 123 | 6.3 | 0.54±0.18 | 1.80±0.14 |
| P2 Bacteria-only, T_15_ | CHO | 2072 | 84.6 | 0.34±0.12 | 1.22±0.30 |
|  | CHON | 210 | 8.6 | 0.32±0.05 | 1.14±0.13 |
|  | CHOS+CHONS | 151 | 6.2 | 0.32±0.11 | 1.52±0.15 |
|  | CHOP+CHONP+CHOSP | 16 | 0.7 | 0.31±0.17 | 1.66±0.19 |
| P1 Light + Bacteria, T_0_ | CHO | 685 | 99.0 | 0.30±0.11 | 1.39±0.17 |
|  | CHON | 1 | 0.1 | 0.29 | 1.21 |
|  | CHOS+CHONS | 5 | 0.7 | 0.16±0.08 | 1.53±0.53 |
|  | CHOP+CHONP+CHOSP | 1 | 0.1 | 0.67 | 1.93 |
| P2 Light + Bacteria, T_0_ | CHO | 1499 | 78.2 | 0.37±0.12 | 1.32±0.23 |
|  | CHON | 264 | 13.8 | 0.39±0.07 | 1.30±0.17 |
|  | CHOS+CHONS | 107 | 5.6 | 0.41±0.15 | 1.68±0.26 |
|  | CHOP+CHONP+CHOSP | 46 | 2.4 | 0.39±0.23 | 1.73±0.20 |

*SD=standard deviation
