# Supplementary material for: Use of ESI-FTICR-MS to Characterize Dissolved Organic Matter in Headwater Streams Draining Forest-Dominated and Pasture-Dominated Watersheds
Source: PLoS One. 2015 Dec 29;10(12):e0145639. doi: 10.1371/journal.pone.0145639 (PMC4694922; doi:10.1371/journal.pone.0145639)

S2 Appendix II: Van Krevelen diagrams of refractory compounds in bacteria+light incubations unique for P1 and P2.

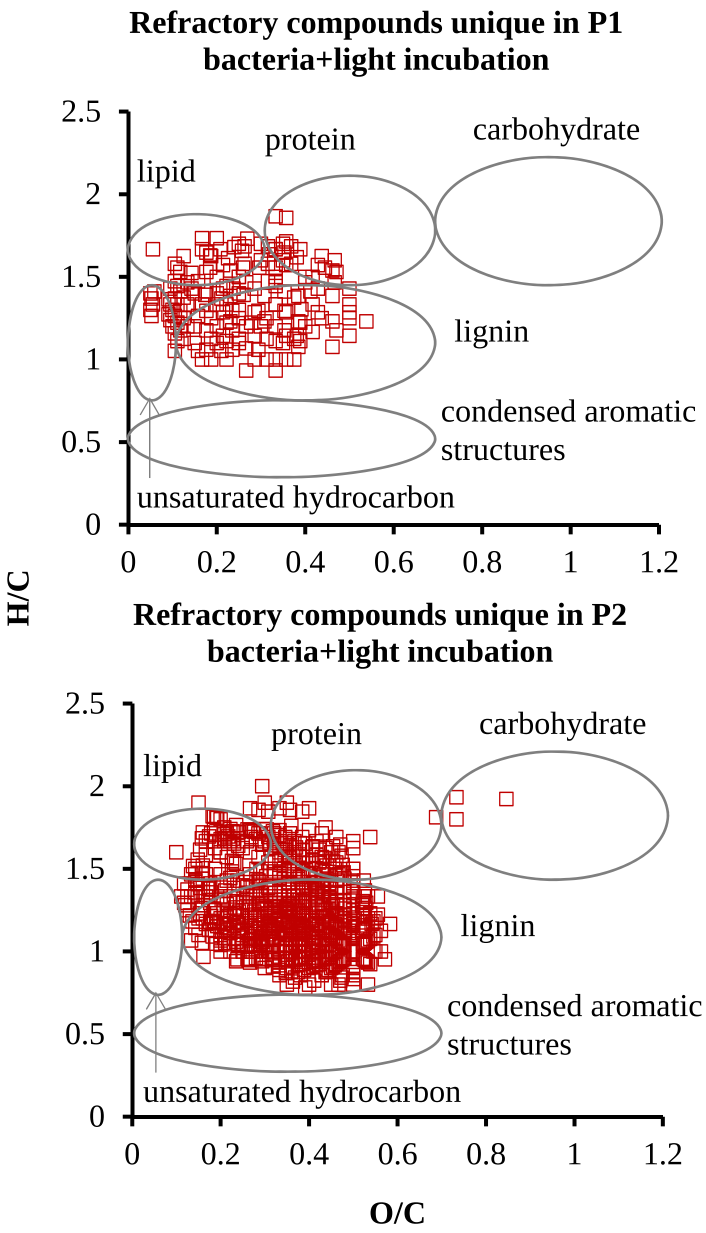

Supplement: S2 Appendix — (PDF) [file pone.0145639.s002.pdf]
