# Supplementary material for: Use of ESI-FTICR-MS to Characterize Dissolved Organic Matter in Headwater Streams Draining Forest-Dominated and Pasture-Dominated Watersheds
Source: PLoS One. 2015 Dec 29;10(12):e0145639. doi: 10.1371/journal.pone.0145639 (PMC4694922; doi:10.1371/journal.pone.0145639)
Supplement: S3 Appendix — (DOCX) [file pone.0145639.s003.docx]

S3 Appendix III: FTICR-MS parameters of the forest and pasture stream DOM samples at T_0_ and T_15_^$^.

| Sample | Formulas in the lipid region^*^ | Formulas in the protein region^*^ | Formulas in the lignin region^*^ | Formulas in the carbohydrate region^*^ | Formulas in the unsaturated hydrocarbon region^*^ |
| --- | --- | --- | --- | --- | --- |
| F1_T_0_ | 190 | 220 | 2400 | 1 | 150 |
|  | 6.2% | 7.1% | 77.8% | 0.03% | 4.9% |
| F2_T_0_ | 143 | 108 | 2034 | 0 | 105 |
|  | 5.6% | 4.2% | 79.0% | 0% | 4.1% |
| P1_T_0_ | 405 | 223 | 1793 | 5 | 149 |
|  | 16.2% | 8.9% | 71.7% | 0.2% | 6.0% |
| P2_T_0_ | 308 | 284 | 1340 | 29 | 34 |
|  | 15.9% | 14.7% | 69.2% | 1.5% | 1.8% |
| F1_T_15_ | 154 | 218 | 2420 | 4 | 152 |
| (bacteria-only) | 5.0% | 7.1% | 79.0% | 0.1% | 5.0% |
| F2_T_15_ | 102 | 148 | 2086 | 1 | 90 |
| (bacteria-only) | 4.0% | 5.7% | 81.0% | 0.04% | 3.5% |
| P1_T_15_ | 136 | 264 | 1020 | 3 | 18 |
| (bacteria-only) | 9.8% | 19.0% | 73.6% | 0.2% | 1.3% |
| P2_T_15_ | 279 | 254 | 1848 | 2 | 27 |
| (bacteria-only) | 11.4% | 10.4% | 75.5% | 0.1% | 1.1% |
| P1_T_15_ | 117 | 98 | 495 | 0 | 15 |
| (light+bacteria) | 16.9% | 14.2% | 71.5% | 0% | 2.2% |
| P2_T_15_ | 202 | 307 | 1452 | 14 | 2 |
| (light+bacteria) | 10.5% | 16.0% | 75.8% | 0.7% | 0.1% |

^$^m/z in the range of 200 – 700; s/n ≥ 4

*this column includes the number of formulas and their percentages relative to total count of peaks with assigned formulas; different regions are defined based on elemental ratios: lipid-like region (H/C = 1.5–2.0, O/C = 0–0.3), protein-like region (H/C = 1.5–2.2, O/C =0.3–0.67), lignin-like region (H/C = 0.7–1.5, O/C=0.1–0.67), carbohydrate-like region (H/C = 1.5–2.4, O/C = 0.67–1.2), and unsaturated hydrocarbon-like region (H/C = 0.7–1.5, O/C = 0–0.1). Note that formulas with delimiting values may be assigned in more than one region.
