# Supplementary material for: Use of ESI-FTICR-MS to Characterize Dissolved Organic Matter in Headwater Streams Draining Forest-Dominated and Pasture-Dominated Watersheds
Source: PLoS One. 2015 Dec 29;10(12):e0145639. doi: 10.1371/journal.pone.0145639 (PMC4694922; doi:10.1371/journal.pone.0145639)
Supplement: S4 Appendix — (DOCX) [file pone.0145639.s004.docx]

**S4 Appendix IV: Parameters for peaks with assigned formulas in T_0_ samples.**

1. Sample: F1_T_0_

| Experimental mass | Exact mass | Peak height | C | H | N | O | S | P |
| --- | --- | --- | --- | --- | --- | --- | --- | --- |
| 217.086995 | 217.087018 | 1366095 | 13 | 14 | 0 | 3 | 0 | 0 |
| 217.123402 | 217.123403 | 2495600 | 14 | 18 | 0 | 2 | 0 | 0 |
| 219.102633 | 219.102668 | 1787897 | 13 | 16 | 0 | 3 | 0 | 0 |
| 219.139071 | 219.139053 | 1552682 | 14 | 20 | 0 | 2 | 0 | 0 |
| 221.081947 | 221.081932 | 1327714 | 12 | 14 | 0 | 4 | 0 | 0 |
| 221.118309 | 221.118318 | 1264932 | 13 | 18 | 0 | 3 | 0 | 0 |
| 223.097556 | 223.097583 | 1580190 | 12 | 16 | 0 | 4 | 0 | 0 |
| 225.076819 | 225.076847 | 1899608 | 11 | 14 | 0 | 5 | 0 | 0 |
| 225.128357 | 225.128489 | 1256475 | 16 | 18 | 0 | 1 | 0 | 0 |
| 227.107814 | 227.107753 | 1687812 | 15 | 16 | 0 | 2 | 0 | 0 |
| 227.144172 | 227.144139 | 1557126 | 16 | 20 | 0 | 1 | 0 | 0 |
| 229.087026 | 229.087018 | 1752251 | 14 | 14 | 0 | 3 | 0 | 0 |
| 229.123415 | 229.123403 | 2437214 | 15 | 18 | 0 | 2 | 0 | 0 |
| 231.102674 | 231.102668 | 2419586 | 14 | 16 | 0 | 3 | 0 | 0 |
| 231.139038 | 231.139053 | 2268645 | 15 | 20 | 0 | 2 | 0 | 0 |
| 233.081981 | 233.081932 | 2442291 | 13 | 14 | 0 | 4 | 0 | 0 |
| 233.118321 | 233.118318 | 3412663 | 14 | 18 | 0 | 3 | 0 | 0 |
| 233.154701 | 233.154703 | 2372506 | 15 | 22 | 0 | 2 | 0 | 0 |
| 235.061273 | 235.061197 | 1428560 | 12 | 12 | 0 | 5 | 0 | 0 |
| 235.097611 | 235.097583 | 3505140 | 13 | 16 | 0 | 4 | 0 | 0 |
| 235.133989 | 235.133968 | 3465144 | 14 | 20 | 0 | 3 | 0 | 0 |
| 237.076817 | 237.076847 | 2249629 | 12 | 14 | 0 | 5 | 0 | 0 |
| 237.113293 | 237.113233 | 2438337 | 13 | 18 | 0 | 4 | 0 | 0 |
| 239.071397 | 239.071368 | 1380978 | 15 | 12 | 0 | 3 | 0 | 0 |
| 239.092457 | 239.092497 | 2946836 | 12 | 16 | 0 | 5 | 0 | 0 |
| 239.10766 | 239.107753 | 1901686 | 16 | 16 | 0 | 2 | 0 | 0 |
| 239.128907 | 239.128883 | 1675032 | 13 | 20 | 0 | 4 | 0 | 0 |
| 239.144056 | 239.144139 | 2373530 | 17 | 20 | 0 | 1 | 0 | 0 |
| 241.0506 | 241.050632 | 1313520 | 14 | 10 | 0 | 4 | 0 | 0 |
| 241.087022 | 241.087018 | 2699764 | 15 | 14 | 0 | 3 | 0 | 0 |
| 241.123403 | 241.123403 | 4203512 | 16 | 18 | 0 | 2 | 0 | 0 |
| 241.159791 | 241.159789 | 3223676 | 17 | 22 | 0 | 1 | 0 | 0 |
| 243.06629 | 243.066282 | 1956507 | 14 | 12 | 0 | 4 | 0 | 0 |
| 243.102691 | 243.102668 | 3524576 | 15 | 16 | 0 | 3 | 0 | 0 |
| 243.139081 | 243.139053 | 5458852 | 16 | 20 | 0 | 2 | 0 | 0 |
| 243.175471 | 243.175439 | 2203048 | 17 | 24 | 0 | 1 | 0 | 0 |
| 245.045593 | 245.045547 | 2132939 | 13 | 10 | 0 | 5 | 0 | 0 |
| 245.081971 | 245.081932 | 3656976 | 14 | 14 | 0 | 4 | 0 | 0 |
| 245.118305 | 245.118318 | 7837972 | 15 | 18 | 0 | 3 | 0 | 0 |
| 245.154746 | 245.154703 | 6348953 | 16 | 22 | 0 | 2 | 0 | 0 |
| 245.191114 | 245.191089 | 1759517 | 17 | 26 | 0 | 1 | 0 | 0 |
| 247.061224 | 247.061197 | 1964040 | 13 | 12 | 0 | 5 | 0 | 0 |
| 247.097568 | 247.097583 | 5245965 | 14 | 16 | 0 | 4 | 0 | 0 |
| 247.133987 | 247.133968 | 6253713 | 15 | 20 | 0 | 3 | 0 | 0 |
| 247.170375 | 247.170354 | 2606870 | 16 | 24 | 0 | 2 | 0 | 0 |
| 249.04042 | 249.040462 | 1394407 | 12 | 10 | 0 | 6 | 0 | 0 |
| 249.076877 | 249.076847 | 3840396 | 13 | 14 | 0 | 5 | 0 | 0 |
| 249.113264 | 249.113233 | 6513681 | 14 | 18 | 0 | 4 | 0 | 0 |
| 249.128446 | 249.128489 | 1237747 | 18 | 18 | 0 | 1 | 0 | 0 |
| 249.149666 | 249.149618 | 4830614 | 15 | 22 | 0 | 3 | 0 | 0 |
| 251.056125 | 251.056112 | 1245202 | 12 | 12 | 0 | 6 | 0 | 0 |
| 251.092552 | 251.092497 | 4505239 | 13 | 16 | 0 | 5 | 0 | 0 |
| 251.107755 | 251.107753 | 2050329 | 17 | 16 | 0 | 2 | 0 | 0 |
| 251.128945 | 251.128883 | 4661404 | 14 | 20 | 0 | 4 | 0 | 0 |
| 251.144203 | 251.144139 | 2216030 | 18 | 20 | 0 | 1 | 0 | 0 |
| 251.165259 | 251.165268 | 1528825 | 15 | 24 | 0 | 3 | 0 | 0 |
| 253.0506 | 253.050632 | 1411072 | 15 | 10 | 0 | 4 | 0 | 0 |
| 253.071792 | 253.071762 | 2042083 | 12 | 14 | 0 | 6 | 0 | 0 |
| 253.087061 | 253.087018 | 3085349 | 16 | 14 | 0 | 3 | 0 | 0 |
| 253.108207 | 253.108147 | 4271528 | 13 | 18 | 0 | 5 | 0 | 0 |
| 253.123422 | 253.123403 | 5303402 | 17 | 18 | 0 | 2 | 0 | 0 |
| 253.144546 | 253.144533 | 2480941 | 14 | 22 | 0 | 4 | 0 | 0 |
| 253.159847 | 253.159789 | 3989039 | 18 | 22 | 0 | 1 | 0 | 0 |
| 255.066299 | 255.066282 | 3426999 | 15 | 12 | 0 | 4 | 0 | 0 |
| 255.087516 | 255.087412 | 1477946 | 12 | 16 | 0 | 6 | 0 | 0 |
| 255.102678 | 255.102668 | 6577084 | 16 | 16 | 0 | 3 | 0 | 0 |
| 255.123912 | 255.123797 | 1387839 | 13 | 20 | 0 | 5 | 0 | 0 |
| 255.139116 | 255.139053 | 10048833 | 17 | 20 | 0 | 2 | 0 | 0 |
| 255.175459 | 255.175439 | 4012422 | 18 | 24 | 0 | 1 | 0 | 0 |
| 257.045522 | 257.045547 | 2603853 | 14 | 10 | 0 | 5 | 0 | 0 |
| 257.081943 | 257.081932 | 5864402 | 15 | 14 | 0 | 4 | 0 | 0 |
| 257.118359 | 257.118318 | 13183575 | 16 | 18 | 0 | 3 | 0 | 0 |
| 257.154719 | 257.154703 | 12813916 | 17 | 22 | 0 | 2 | 0 | 0 |
| 257.19118 | 257.191089 | 2775202 | 18 | 26 | 0 | 1 | 0 | 0 |
| 259.024856 | 259.024812 | 1740967 | 13 | 8 | 0 | 6 | 0 | 0 |
| 259.061234 | 259.061197 | 3441068 | 14 | 12 | 0 | 5 | 0 | 0 |
| 259.097633 | 259.097583 | 8618610 | 15 | 16 | 0 | 4 | 0 | 0 |
| 259.133989 | 259.133968 | 13969783 | 16 | 20 | 0 | 3 | 0 | 0 |
| 259.170413 | 259.170354 | 8936828 | 17 | 24 | 0 | 2 | 0 | 0 |
| 259.206733 | 259.206739 | 1337185 | 18 | 28 | 0 | 1 | 0 | 0 |
| 261.040544 | 261.040462 | 2065002 | 13 | 10 | 0 | 6 | 0 | 0 |
| 261.076832 | 261.076847 | 5426703 | 14 | 14 | 0 | 5 | 0 | 0 |
| 261.113283 | 261.113233 | 12517268 | 15 | 18 | 0 | 4 | 0 | 0 |
| 261.149625 | 261.149618 | 12469018 | 16 | 22 | 0 | 3 | 0 | 0 |
| 261.18606 | 261.186004 | 4038943 | 17 | 26 | 0 | 2 | 0 | 0 |
| 263.056084 | 263.056112 | 2422319 | 13 | 12 | 0 | 6 | 0 | 0 |
| 263.071261 | 263.071368 | 1379441 | 17 | 12 | 0 | 3 | 0 | 0 |
| 263.092526 | 263.092497 | 6023989 | 14 | 16 | 0 | 5 | 0 | 0 |
| 263.107685 | 263.107753 | 2175031 | 18 | 16 | 0 | 2 | 0 | 0 |
| 263.128937 | 263.128883 | 11982010 | 15 | 20 | 0 | 4 | 0 | 0 |
| 263.144191 | 263.144139 | 2452412 | 19 | 20 | 0 | 1 | 0 | 0 |
| 263.165231 | 263.165268 | 6430271 | 16 | 24 | 0 | 3 | 0 | 0 |
| 263.201633 | 263.201654 | 1261317 | 17 | 28 | 0 | 2 | 0 | 0 |
| 265.050758 | 265.050632 | 1990612 | 16 | 10 | 0 | 4 | 0 | 0 |
| 265.071804 | 265.071762 | 3173847 | 13 | 14 | 0 | 6 | 0 | 0 |
| 265.08702 | 265.087018 | 3804249 | 17 | 14 | 0 | 3 | 0 | 0 |
| 265.108199 | 265.108147 | 9174236 | 14 | 18 | 0 | 5 | 0 | 0 |
| 265.123388 | 265.123403 | 6527710 | 18 | 18 | 0 | 2 | 0 | 0 |
| 265.144515 | 265.144533 | 10636769 | 15 | 22 | 0 | 4 | 0 | 0 |
| 265.159799 | 265.159789 | 4456164 | 19 | 22 | 0 | 1 | 0 | 0 |
| 265.180963 | 265.180918 | 2153959 | 16 | 26 | 0 | 3 | 0 | 0 |
| 267.066296 | 267.066282 | 2893628 | 16 | 12 | 0 | 4 | 0 | 0 |
| 267.087366 | 267.087412 | 3476607 | 13 | 16 | 0 | 6 | 0 | 0 |
| 267.102709 | 267.102668 | 7995394 | 17 | 16 | 0 | 3 | 0 | 0 |
| 267.12381 | 267.123797 | 7305989 | 14 | 20 | 0 | 5 | 0 | 0 |
| 267.13907 | 267.139053 | 11838983 | 18 | 20 | 0 | 2 | 0 | 0 |
| 267.160209 | 267.160183 | 4235466 | 15 | 24 | 0 | 4 | 0 | 0 |
| 267.175446 | 267.175439 | 5614988 | 19 | 24 | 0 | 1 | 0 | 0 |
| 269.04548 | 269.045547 | 3481184 | 15 | 10 | 0 | 5 | 0 | 0 |
| 269.081915 | 269.081932 | 7799333 | 16 | 14 | 0 | 4 | 0 | 0 |
| 269.093297 | 269.093166 | 1493095 | 15 | 14 | 2 | 3 | 0 | 0 |
| 269.103076 | 269.103062 | 3261352 | 13 | 18 | 0 | 6 | 0 | 0 |
| 269.118352 | 269.118318 | 17369642 | 17 | 18 | 0 | 3 | 0 | 0 |
| 269.139511 | 269.139447 | 3924142 | 14 | 22 | 0 | 5 | 0 | 0 |
| 269.154729 | 269.154703 | 20042800 | 18 | 22 | 0 | 2 | 0 | 0 |
| 269.17598 | 269.175833 | 1461235 | 15 | 26 | 0 | 4 | 0 | 0 |
| 269.191079 | 269.191089 | 6891830 | 19 | 26 | 0 | 1 | 0 | 0 |
| 270.053447 | 270.053683 | 1624757 | 11 | 14 | 1 | 5 | 0 | 1 |
| 270.077189 | 270.077181 | 1547704 | 15 | 13 | 1 | 4 | 0 | 0 |
| 270.126277 | 270.126454 | 1751743 | 13 | 22 | 1 | 3 | 0 | 1 |
| 271.024799 | 271.024812 | 2340036 | 14 | 8 | 0 | 6 | 0 | 0 |
| 271.061202 | 271.061197 | 5526985 | 15 | 12 | 0 | 5 | 0 | 0 |
| 271.097599 | 271.097583 | 13778766 | 16 | 16 | 0 | 4 | 0 | 0 |
| 271.108836 | 271.108816 | 1731792 | 15 | 16 | 2 | 3 | 0 | 0 |
| 271.118753 | 271.118712 | 1525393 | 13 | 20 | 0 | 6 | 0 | 0 |
| 271.133971 | 271.133968 | 25317204 | 17 | 20 | 0 | 3 | 0 | 0 |
| 271.170385 | 271.170354 | 19556184 | 18 | 24 | 0 | 2 | 0 | 0 |
| 271.206765 | 271.206739 | 3644510 | 19 | 28 | 0 | 1 | 0 | 0 |
| 272.092919 | 272.092832 | 1374433 | 15 | 15 | 1 | 4 | 0 | 0 |
| 272.129269 | 272.129217 | 1549158 | 16 | 19 | 1 | 3 | 0 | 0 |
| 273.040538 | 273.040462 | 4769388 | 14 | 10 | 0 | 6 | 0 | 0 |
| 273.076914 | 273.076847 | 8351089 | 15 | 14 | 0 | 5 | 0 | 0 |
| 273.113259 | 273.113233 | 20171382 | 16 | 18 | 0 | 4 | 0 | 0 |
| 273.149643 | 273.149618 | 27549308 | 17 | 22 | 0 | 3 | 0 | 0 |
| 273.186004 | 273.186004 | 12417153 | 18 | 26 | 0 | 2 | 0 | 0 |
| 273.207173 | 273.207133 | 1700484 | 15 | 30 | 0 | 4 | 0 | 0 |
| 274.108545 | 274.108482 | 1866376 | 15 | 17 | 1 | 4 | 0 | 0 |
| 274.145015 | 274.144867 | 1215694 | 16 | 21 | 1 | 3 | 0 | 0 |
| 275.019731 | 275.019726 | 1617485 | 13 | 8 | 0 | 7 | 0 | 0 |
| 275.056077 | 275.056112 | 4719123 | 14 | 12 | 0 | 6 | 0 | 0 |
| 275.092493 | 275.092497 | 12169880 | 15 | 16 | 0 | 5 | 0 | 0 |
| 275.107832 | 275.107753 | 2215514 | 19 | 16 | 0 | 2 | 0 | 0 |
| 275.12891 | 275.128883 | 22832030 | 16 | 20 | 0 | 4 | 0 | 0 |
| 275.14417 | 275.144139 | 2397471 | 20 | 20 | 0 | 1 | 0 | 0 |
| 275.165333 | 275.165268 | 18299298 | 17 | 24 | 0 | 3 | 0 | 0 |
| 275.201691 | 275.201654 | 4048872 | 18 | 28 | 0 | 2 | 0 | 0 |
| 276.087783 | 276.087746 | 1230537 | 14 | 15 | 1 | 5 | 0 | 0 |
| 277.035343 | 277.035376 | 1984754 | 13 | 10 | 0 | 7 | 0 | 0 |
| 277.050774 | 277.050632 | 1246260 | 17 | 10 | 0 | 4 | 0 | 0 |
| 277.071749 | 277.071762 | 5690551 | 14 | 14 | 0 | 6 | 0 | 0 |
| 277.087053 | 277.087018 | 3183801 | 18 | 14 | 0 | 3 | 0 | 0 |
| 277.108132 | 277.108147 | 14131388 | 15 | 18 | 0 | 5 | 0 | 0 |
| 277.123398 | 277.123403 | 5175487 | 19 | 18 | 0 | 2 | 0 | 0 |
| 277.159815 | 277.159789 | 4509380 | 20 | 22 | 0 | 1 | 0 | 0 |
| 277.180964 | 277.180918 | 9627591 | 17 | 26 | 0 | 3 | 0 | 0 |
| 279.030008 | 279.029897 | 1343952 | 16 | 8 | 0 | 5 | 0 | 0 |
| 279.051054 | 279.051026 | 2183123 | 13 | 12 | 0 | 7 | 0 | 0 |
| 279.066304 | 279.066282 | 3785686 | 17 | 12 | 0 | 4 | 0 | 0 |
| 279.087388 | 279.087412 | 6616793 | 14 | 16 | 0 | 6 | 0 | 0 |
| 279.102676 | 279.102668 | 7782619 | 18 | 16 | 0 | 3 | 0 | 0 |
| 279.123791 | 279.123797 | 17647070 | 15 | 20 | 0 | 5 | 0 | 0 |
| 279.139084 | 279.139053 | 11599072 | 19 | 20 | 0 | 2 | 0 | 0 |
| 279.160187 | 279.160183 | 11132643 | 16 | 24 | 0 | 4 | 0 | 0 |
| 279.17548 | 279.175439 | 6943205 | 20 | 24 | 0 | 1 | 0 | 0 |
| 279.196518 | 279.196568 | 2449512 | 17 | 28 | 0 | 3 | 0 | 0 |
| 280.074155 | 280.074418 | 1778853 | 13 | 16 | 1 | 4 | 0 | 1 |
| 280.09798 | 280.097917 | 1403016 | 17 | 15 | 1 | 3 | 0 | 0 |
| 280.110549 | 280.110804 | 2446570 | 14 | 20 | 1 | 3 | 0 | 1 |
| 280.131756 | 280.131933 | 1241965 | 11 | 24 | 1 | 5 | 0 | 1 |
| 281.045566 | 281.045547 | 3344814 | 16 | 10 | 0 | 5 | 0 | 0 |
| 281.066688 | 281.066676 | 1641009 | 13 | 14 | 0 | 7 | 0 | 0 |
| 281.081962 | 281.081932 | 7736820 | 17 | 14 | 0 | 4 | 0 | 0 |
| 281.093094 | 281.093166 | 1329141 | 16 | 14 | 2 | 3 | 0 | 0 |
| 281.103082 | 281.103062 | 7725814 | 14 | 18 | 0 | 6 | 0 | 0 |
| 281.118381 | 281.118318 | 17419512 | 18 | 18 | 0 | 3 | 0 | 0 |
| 281.139452 | 281.139447 | 14007291 | 15 | 22 | 0 | 5 | 0 | 0 |
| 281.154721 | 281.154703 | 21393150 | 19 | 22 | 0 | 2 | 0 | 0 |
| 281.175862 | 281.175833 | 3648769 | 16 | 26 | 0 | 4 | 0 | 0 |
| 281.191086 | 281.191089 | 7990787 | 20 | 26 | 0 | 1 | 0 | 0 |
| 282.077223 | 282.077181 | 1528831 | 16 | 13 | 1 | 4 | 0 | 0 |
| 282.11357 | 282.113567 | 1814020 | 17 | 17 | 1 | 3 | 0 | 0 |
| 283.024775 | 283.024812 | 1950371 | 15 | 8 | 0 | 6 | 0 | 0 |
| 283.061227 | 283.061197 | 6673416 | 16 | 12 | 0 | 5 | 0 | 0 |
| 283.082329 | 283.082326 | 1621371 | 13 | 16 | 0 | 7 | 0 | 0 |
| 283.097612 | 283.097583 | 14363661 | 17 | 16 | 0 | 4 | 0 | 0 |
| 283.108715 | 283.108816 | 2141326 | 16 | 16 | 2 | 3 | 0 | 0 |
| 283.118694 | 283.118712 | 5361936 | 14 | 20 | 0 | 6 | 0 | 0 |
| 283.133999 | 283.133968 | 28122130 | 18 | 20 | 0 | 3 | 0 | 0 |
| 283.155089 | 283.155097 | 6320405 | 15 | 24 | 0 | 5 | 0 | 0 |
| 283.170379 | 283.170354 | 25649174 | 19 | 24 | 0 | 2 | 0 | 0 |
| 283.206756 | 283.206739 | 5015324 | 20 | 28 | 0 | 1 | 0 | 0 |
| 284.056377 | 284.056446 | 1454993 | 15 | 11 | 1 | 5 | 0 | 0 |
| 284.092791 | 284.092832 | 2253270 | 16 | 15 | 1 | 4 | 0 | 0 |
| 284.105478 | 284.105718 | 2472792 | 13 | 20 | 1 | 4 | 0 | 1 |
| 284.129106 | 284.129217 | 2004635 | 17 | 19 | 1 | 3 | 0 | 0 |
| 284.141849 | 284.142104 | 2061213 | 14 | 24 | 1 | 3 | 0 | 1 |
| 284.165525 | 284.165603 | 1404832 | 18 | 23 | 1 | 2 | 0 | 0 |
| 285.040457 | 285.040462 | 7203352 | 15 | 10 | 0 | 6 | 0 | 0 |
| 285.076827 | 285.076847 | 11534108 | 16 | 14 | 0 | 5 | 0 | 0 |
| 285.088157 | 285.08808 | 1820510 | 15 | 14 | 2 | 4 | 0 | 0 |
| 285.097879 | 285.097976 | 1907871 | 13 | 18 | 0 | 7 | 0 | 0 |
| 285.113251 | 285.113233 | 26328354 | 17 | 18 | 0 | 4 | 0 | 0 |
| 285.124621 | 285.124466 | 1564675 | 16 | 18 | 2 | 3 | 0 | 0 |
| 285.134325 | 285.134362 | 2555428 | 14 | 22 | 0 | 6 | 0 | 0 |
| 285.149617 | 285.149618 | 41589032 | 18 | 22 | 0 | 3 | 0 | 0 |
| 285.170672 | 285.170747 | 2124905 | 15 | 26 | 0 | 5 | 0 | 0 |
| 285.186014 | 285.186004 | 22243116 | 19 | 26 | 0 | 2 | 0 | 0 |
| 285.222372 | 285.222389 | 2497840 | 20 | 30 | 0 | 1 | 0 | 0 |
| 286.048351 | 286.048597 | 1497248 | 11 | 14 | 1 | 6 | 0 | 1 |
| 286.072207 | 286.072096 | 1555747 | 15 | 13 | 1 | 5 | 0 | 0 |
| 286.108422 | 286.108482 | 2135400 | 16 | 17 | 1 | 4 | 0 | 0 |
| 286.144874 | 286.144867 | 1818285 | 17 | 21 | 1 | 3 | 0 | 0 |
| 287.019762 | 287.019726 | 2801825 | 14 | 8 | 0 | 7 | 0 | 0 |
| 287.056149 | 287.056112 | 6840358 | 15 | 12 | 0 | 6 | 0 | 0 |
| 287.092517 | 287.092497 | 14768171 | 16 | 16 | 0 | 5 | 0 | 0 |
| 287.103686 | 287.103731 | 2402605 | 15 | 16 | 2 | 4 | 0 | 0 |
| 287.107739 | 287.107753 | 1587629 | 20 | 16 | 0 | 2 | 0 | 0 |
| 287.128906 | 287.128883 | 33664560 | 17 | 20 | 0 | 4 | 0 | 0 |
| 287.144162 | 287.144139 | 1926450 | 21 | 20 | 0 | 1 | 0 | 0 |
| 287.150131 | 287.150012 | 1561011 | 14 | 24 | 0 | 6 | 0 | 0 |
| 287.16528 | 287.165268 | 35734068 | 18 | 24 | 0 | 3 | 0 | 0 |
| 287.186393 | 287.186398 | 1236024 | 15 | 28 | 0 | 5 | 0 | 0 |
| 287.201667 | 287.201654 | 10529850 | 19 | 28 | 0 | 2 | 0 | 0 |
| 288.087852 | 288.087746 | 1471599 | 15 | 15 | 1 | 5 | 0 | 0 |
| 288.124077 | 288.124132 | 1839923 | 16 | 19 | 1 | 4 | 0 | 0 |
| 288.136746 | 288.137018 | 1398197 | 13 | 24 | 1 | 4 | 0 | 1 |
| 289.035375 | 289.035376 | 3927083 | 14 | 10 | 0 | 7 | 0 | 0 |
| 289.07176 | 289.071762 | 7654959 | 15 | 14 | 0 | 6 | 0 | 0 |
| 289.087007 | 289.087018 | 2582961 | 19 | 14 | 0 | 3 | 0 | 0 |
| 289.108137 | 289.108147 | 19039028 | 16 | 18 | 0 | 5 | 0 | 0 |
| 289.123423 | 289.123403 | 3716534 | 20 | 18 | 0 | 2 | 0 | 0 |
| 289.144516 | 289.144533 | 33096504 | 17 | 22 | 0 | 4 | 0 | 0 |
| 289.159795 | 289.159789 | 2716219 | 21 | 22 | 0 | 1 | 0 | 0 |
| 289.180912 | 289.180918 | 19605310 | 18 | 26 | 0 | 3 | 0 | 0 |
| 289.217325 | 289.217304 | 3227842 | 19 | 30 | 0 | 2 | 0 | 0 |
| 290.103395 | 290.103396 | 1648821 | 15 | 17 | 1 | 5 | 0 | 0 |
| 290.115993 | 290.116283 | 1539638 | 12 | 22 | 1 | 5 | 0 | 1 |
| 290.139854 | 290.139782 | 1422713 | 16 | 21 | 1 | 4 | 0 | 0 |
| 291.014645 | 291.014641 | 1346089 | 13 | 8 | 0 | 8 | 0 | 0 |
| 291.050965 | 291.051026 | 3370414 | 14 | 12 | 0 | 7 | 0 | 0 |
| 291.066308 | 291.066282 | 2920496 | 18 | 12 | 0 | 4 | 0 | 0 |
| 291.087392 | 291.087412 | 9350194 | 15 | 16 | 0 | 6 | 0 | 0 |
| 291.102709 | 291.102668 | 6700597 | 19 | 16 | 0 | 3 | 0 | 0 |
| 291.12379 | 291.123797 | 23452726 | 16 | 20 | 0 | 5 | 0 | 0 |
| 291.139075 | 291.139053 | 9587769 | 20 | 20 | 0 | 2 | 0 | 0 |
| 291.160173 | 291.160183 | 26871868 | 17 | 24 | 0 | 4 | 0 | 0 |
| 291.175485 | 291.175439 | 5786430 | 21 | 24 | 0 | 1 | 0 | 0 |
| 291.196541 | 291.196568 | 9761344 | 18 | 28 | 0 | 3 | 0 | 0 |
| 293.045548 | 293.045547 | 3427369 | 17 | 10 | 0 | 5 | 0 | 0 |
| 293.066631 | 293.066676 | 3059756 | 14 | 14 | 0 | 7 | 0 | 0 |
| 293.081972 | 293.081932 | 6335534 | 18 | 14 | 0 | 4 | 0 | 0 |
| 293.093302 | 293.093166 | 1386799 | 17 | 14 | 2 | 3 | 0 | 0 |
| 293.103049 | 293.103062 | 10497840 | 15 | 18 | 0 | 6 | 0 | 0 |
| 293.11833 | 293.118318 | 14274354 | 19 | 18 | 0 | 3 | 0 | 0 |
| 293.139433 | 293.139447 | 19691828 | 16 | 22 | 0 | 5 | 0 | 0 |
| 293.154728 | 293.154703 | 16525622 | 20 | 22 | 0 | 2 | 0 | 0 |
| 293.191108 | 293.191089 | 7333947 | 21 | 26 | 0 | 1 | 0 | 0 |
| 293.212208 | 293.212218 | 2774718 | 18 | 30 | 0 | 3 | 0 | 0 |
| 294.077221 | 294.077181 | 1475113 | 17 | 13 | 1 | 4 | 0 | 0 |
| 294.089805 | 294.090068 | 2136170 | 14 | 18 | 1 | 4 | 0 | 1 |
| 294.113574 | 294.113567 | 1577037 | 18 | 17 | 1 | 3 | 0 | 0 |
| 294.126189 | 294.126454 | 2725807 | 15 | 22 | 1 | 3 | 0 | 1 |
| 295.024775 | 295.024812 | 2277917 | 16 | 8 | 0 | 6 | 0 | 0 |
| 295.061228 | 295.061197 | 6175265 | 17 | 12 | 0 | 5 | 0 | 0 |
| 295.082337 | 295.082326 | 2482724 | 14 | 16 | 0 | 7 | 0 | 0 |
| 295.097589 | 295.097583 | 13305382 | 18 | 16 | 0 | 4 | 0 | 0 |
| 295.108814 | 295.108816 | 1864103 | 17 | 16 | 2 | 3 | 0 | 0 |
| 295.118713 | 295.118712 | 12165672 | 15 | 20 | 0 | 6 | 0 | 0 |
| 295.133962 | 295.133968 | 25567786 | 19 | 20 | 0 | 3 | 0 | 0 |
| 295.1551 | 295.155097 | 11242541 | 16 | 24 | 0 | 5 | 0 | 0 |
| 295.170342 | 295.170354 | 23825966 | 20 | 24 | 0 | 2 | 0 | 0 |
| 295.191497 | 295.191483 | 2790193 | 17 | 28 | 0 | 4 | 0 | 0 |
| 295.206744 | 295.206739 | 7325747 | 21 | 28 | 0 | 1 | 0 | 0 |
| 296.032665 | 296.032947 | 1388759 | 12 | 12 | 1 | 6 | 0 | 1 |
| 296.092863 | 296.092832 | 1864222 | 17 | 15 | 1 | 4 | 0 | 0 |
| 296.1055 | 296.105718 | 2690592 | 14 | 20 | 1 | 4 | 0 | 1 |
| 296.129179 | 296.129217 | 2259427 | 18 | 19 | 1 | 3 | 0 | 0 |
| 296.141834 | 296.142104 | 2350756 | 15 | 24 | 1 | 3 | 0 | 1 |
| 296.16567 | 296.165603 | 1384487 | 19 | 23 | 1 | 2 | 0 | 0 |
| 297.00414 | 297.004076 | 1426572 | 15 | 6 | 0 | 7 | 0 | 0 |
| 297.040437 | 297.040462 | 5491984 | 16 | 10 | 0 | 6 | 0 | 0 |
| 297.076805 | 297.076847 | 11046164 | 17 | 14 | 0 | 5 | 0 | 0 |
| 297.088181 | 297.08808 | 1974806 | 16 | 14 | 2 | 4 | 0 | 0 |
| 297.098072 | 297.097976 | 3123863 | 14 | 18 | 0 | 7 | 0 | 0 |
| 297.113201 | 297.113233 | 26913560 | 18 | 18 | 0 | 4 | 0 | 0 |
| 297.116521 | 297.116603 | 1494553 | 15 | 22 | 0 | 4 | 1 | 0 |
| 297.124359 | 297.124466 | 1853466 | 17 | 18 | 2 | 3 | 0 | 0 |
| 297.134394 | 297.134362 | 11072283 | 15 | 22 | 0 | 6 | 0 | 0 |
| 297.149591 | 297.149618 | 43749148 | 19 | 22 | 0 | 3 | 0 | 0 |
| 297.170784 | 297.170747 | 5009440 | 16 | 26 | 0 | 5 | 0 | 0 |
| 297.185987 | 297.186004 | 27100962 | 20 | 26 | 0 | 2 | 0 | 0 |
| 297.222384 | 297.222389 | 4264230 | 21 | 30 | 0 | 1 | 0 | 0 |
| 298.035673 | 298.035711 | 1301638 | 15 | 9 | 1 | 6 | 0 | 0 |
| 298.048375 | 298.048597 | 1728328 | 12 | 14 | 1 | 6 | 0 | 1 |
| 298.072125 | 298.072096 | 2177162 | 16 | 13 | 1 | 5 | 0 | 0 |
| 298.108427 | 298.108482 | 2385039 | 17 | 17 | 1 | 4 | 0 | 0 |
| 298.144844 | 298.144867 | 2593299 | 18 | 21 | 1 | 3 | 0 | 0 |
| 298.181316 | 298.181253 | 1439511 | 19 | 25 | 1 | 2 | 0 | 0 |
| 299.01974 | 299.019726 | 3004922 | 15 | 8 | 0 | 7 | 0 | 0 |
| 299.056108 | 299.056112 | 8392702 | 16 | 12 | 0 | 6 | 0 | 0 |
| 299.092478 | 299.092497 | 17332226 | 17 | 16 | 0 | 5 | 0 | 0 |
| 299.103726 | 299.103731 | 2696324 | 16 | 16 | 2 | 4 | 0 | 0 |
| 299.113622 | 299.113627 | 2610949 | 14 | 20 | 0 | 7 | 0 | 0 |
| 299.128888 | 299.128883 | 37998600 | 18 | 20 | 0 | 4 | 0 | 0 |
| 299.132192 | 299.132253 | 1478535 | 15 | 24 | 0 | 4 | 1 | 0 |
| 299.140047 | 299.140116 | 1382280 | 17 | 20 | 2 | 3 | 0 | 0 |
| 299.150058 | 299.150012 | 4513289 | 15 | 24 | 0 | 6 | 0 | 0 |
| 299.16528 | 299.165268 | 45690892 | 19 | 24 | 0 | 3 | 0 | 0 |
| 299.186368 | 299.186398 | 2107661 | 16 | 28 | 0 | 5 | 0 | 0 |
| 299.201673 | 299.201654 | 18553870 | 20 | 28 | 0 | 2 | 0 | 0 |
| 299.238112 | 299.238039 | 1561987 | 21 | 32 | 0 | 1 | 0 | 0 |
| 300.087695 | 300.087746 | 1827574 | 16 | 15 | 1 | 5 | 0 | 0 |
| 300.124081 | 300.124132 | 2667898 | 17 | 19 | 1 | 4 | 0 | 0 |
| 300.160471 | 300.160517 | 1911678 | 18 | 23 | 1 | 3 | 0 | 0 |
| 301.035372 | 301.035376 | 6474723 | 15 | 10 | 0 | 7 | 0 | 0 |
| 301.071806 | 301.071762 | 8936679 | 16 | 14 | 0 | 6 | 0 | 0 |
| 301.087007 | 301.087018 | 2723177 | 20 | 14 | 0 | 3 | 0 | 0 |
| 301.108172 | 301.108147 | 20837612 | 17 | 18 | 0 | 5 | 0 | 0 |
| 301.119417 | 301.119381 | 1934445 | 16 | 18 | 2 | 4 | 0 | 0 |
| 301.123402 | 301.123403 | 2493677 | 21 | 18 | 0 | 2 | 0 | 0 |
| 301.144542 | 301.144533 | 41214192 | 18 | 22 | 0 | 4 | 0 | 0 |
| 301.147813 | 301.147904 | 1303280 | 15 | 26 | 0 | 4 | 1 | 0 |
| 301.15995 | 301.159789 | 1541745 | 22 | 22 | 0 | 1 | 0 | 0 |
| 301.180924 | 301.180918 | 31297780 | 19 | 26 | 0 | 3 | 0 | 0 |
| 301.217293 | 301.217304 | 7047928 | 20 | 30 | 0 | 2 | 0 | 0 |
| 302.103459 | 302.103396 | 1588061 | 16 | 17 | 1 | 5 | 0 | 0 |
| 302.139767 | 302.139782 | 1917409 | 17 | 21 | 1 | 4 | 0 | 0 |
| 303.014594 | 303.014641 | 1286148 | 14 | 8 | 0 | 8 | 0 | 0 |
| 303.051013 | 303.051026 | 5326280 | 15 | 12 | 0 | 7 | 0 | 0 |
| 303.066289 | 303.066282 | 2604745 | 19 | 12 | 0 | 4 | 0 | 0 |
| 303.087402 | 303.087412 | 11098060 | 16 | 16 | 0 | 6 | 0 | 0 |
| 303.102712 | 303.102668 | 3983821 | 20 | 16 | 0 | 3 | 0 | 0 |
| 303.123776 | 303.123797 | 26823120 | 17 | 20 | 0 | 5 | 0 | 0 |
| 303.139099 | 303.139053 | 5666002 | 21 | 20 | 0 | 2 | 0 | 0 |
| 303.16016 | 303.160183 | 37437908 | 18 | 24 | 0 | 4 | 0 | 0 |
| 303.175431 | 303.175439 | 4346838 | 22 | 24 | 0 | 1 | 0 | 0 |
| 303.196547 | 303.196568 | 15661016 | 19 | 28 | 0 | 3 | 0 | 0 |
| 304.097996 | 304.097917 | 1360829 | 19 | 15 | 1 | 3 | 0 | 0 |
| 304.119028 | 304.119046 | 1818175 | 16 | 19 | 1 | 5 | 0 | 0 |
| 304.155446 | 304.155432 | 1737923 | 17 | 23 | 1 | 4 | 0 | 0 |
| 305.030273 | 305.030291 | 2132388 | 14 | 10 | 0 | 8 | 0 | 0 |
| 305.045616 | 305.045547 | 2456869 | 18 | 10 | 0 | 5 | 0 | 0 |
| 305.066656 | 305.066676 | 4925096 | 15 | 14 | 0 | 7 | 0 | 0 |
| 305.081964 | 305.081932 | 5258025 | 19 | 14 | 0 | 4 | 0 | 0 |
| 305.103042 | 305.103062 | 12289708 | 16 | 18 | 0 | 6 | 0 | 0 |
| 305.114228 | 305.114295 | 1302445 | 15 | 18 | 2 | 5 | 0 | 0 |
| 305.118327 | 305.118318 | 10447533 | 20 | 18 | 0 | 3 | 0 | 0 |
| 305.139445 | 305.139447 | 29303472 | 17 | 22 | 0 | 5 | 0 | 0 |
| 305.154692 | 305.154703 | 13055153 | 21 | 22 | 0 | 2 | 0 | 0 |
| 305.175867 | 305.175833 | 24674996 | 18 | 26 | 0 | 4 | 0 | 0 |
| 305.191058 | 305.191089 | 7073973 | 22 | 26 | 0 | 1 | 0 | 0 |
| 306.077153 | 306.077181 | 1420502 | 18 | 13 | 1 | 4 | 0 | 0 |
| 306.113554 | 306.113567 | 1668250 | 19 | 17 | 1 | 3 | 0 | 0 |
| 306.149928 | 306.149952 | 1308446 | 20 | 21 | 1 | 2 | 0 | 0 |
| 307.024919 | 307.024812 | 1823613 | 17 | 8 | 0 | 6 | 0 | 0 |
| 307.046012 | 307.045941 | 1548095 | 14 | 12 | 0 | 8 | 0 | 0 |
| 307.061196 | 307.061197 | 4922753 | 18 | 12 | 0 | 5 | 0 | 0 |
| 307.082312 | 307.082326 | 4685955 | 15 | 16 | 0 | 7 | 0 | 0 |
| 307.097563 | 307.097583 | 10697605 | 19 | 16 | 0 | 4 | 0 | 0 |
| 307.108906 | 307.108816 | 1392006 | 18 | 16 | 2 | 3 | 0 | 0 |
| 307.118702 | 307.118712 | 14722951 | 16 | 20 | 0 | 6 | 0 | 0 |
| 307.13395 | 307.133968 | 19013512 | 20 | 20 | 0 | 3 | 0 | 0 |
| 307.155121 | 307.155097 | 22601610 | 17 | 24 | 0 | 5 | 0 | 0 |
| 307.170333 | 307.170354 | 20688780 | 21 | 24 | 0 | 2 | 0 | 0 |
| 307.191474 | 307.191483 | 10146703 | 18 | 28 | 0 | 4 | 0 | 0 |
| 307.206759 | 307.206739 | 7782033 | 22 | 28 | 0 | 1 | 0 | 0 |
| 308.056538 | 308.056446 | 1251884 | 17 | 11 | 1 | 5 | 0 | 0 |
| 308.092845 | 308.092832 | 2020976 | 18 | 15 | 1 | 4 | 0 | 0 |
| 308.129356 | 308.129217 | 1837300 | 19 | 19 | 1 | 3 | 0 | 0 |
| 308.141827 | 308.142104 | 2519605 | 16 | 24 | 1 | 3 | 0 | 1 |
| 309.040465 | 309.040462 | 4556629 | 17 | 10 | 0 | 6 | 0 | 0 |
| 309.061585 | 309.061591 | 1404247 | 14 | 14 | 0 | 8 | 0 | 0 |
| 309.076803 | 309.076847 | 8920152 | 18 | 14 | 0 | 5 | 0 | 0 |
| 309.088 | 309.08808 | 1580378 | 17 | 14 | 2 | 4 | 0 | 0 |
| 309.097975 | 309.097976 | 4987739 | 15 | 18 | 0 | 7 | 0 | 0 |
| 309.113218 | 309.113233 | 19498076 | 19 | 18 | 0 | 4 | 0 | 0 |
| 309.124353 | 309.124466 | 1421917 | 18 | 18 | 2 | 3 | 0 | 0 |
| 309.134385 | 309.134362 | 14643806 | 16 | 22 | 0 | 6 | 0 | 0 |
| 309.14961 | 309.149618 | 30872672 | 20 | 22 | 0 | 3 | 0 | 0 |
| 309.152983 | 309.152989 | 1362720 | 17 | 26 | 0 | 3 | 1 | 0 |
| 309.170763 | 309.170747 | 11775074 | 17 | 26 | 0 | 5 | 0 | 0 |
| 309.174143 | 309.174118 | 3422563 | 14 | 30 | 0 | 5 | 1 | 0 |
| 309.186016 | 309.186004 | 24257636 | 21 | 26 | 0 | 2 | 0 | 0 |
| 309.2224 | 309.222389 | 5637736 | 22 | 30 | 0 | 1 | 0 | 0 |
| 310.072091 | 310.072096 | 1904577 | 17 | 13 | 1 | 5 | 0 | 0 |
| 310.108456 | 310.108482 | 2700357 | 18 | 17 | 1 | 4 | 0 | 0 |
| 310.144848 | 310.144867 | 2269001 | 19 | 21 | 1 | 3 | 0 | 0 |
| 311.019784 | 311.019726 | 2569828 | 16 | 8 | 0 | 7 | 0 | 0 |
| 311.056088 | 311.056112 | 8013096 | 17 | 12 | 0 | 6 | 0 | 0 |
| 311.09247 | 311.092497 | 15656236 | 18 | 16 | 0 | 5 | 0 | 0 |
| 311.095953 | 311.095868 | 1319212 | 15 | 20 | 0 | 5 | 1 | 0 |
| 311.103744 | 311.103731 | 2869677 | 17 | 16 | 2 | 4 | 0 | 0 |
| 311.113645 | 311.113627 | 6047534 | 15 | 20 | 0 | 7 | 0 | 0 |
| 311.128853 | 311.128883 | 33588528 | 19 | 20 | 0 | 4 | 0 | 0 |
| 311.15004 | 311.150012 | 8665906 | 16 | 24 | 0 | 6 | 0 | 0 |
| 311.163162 | 311.162899 | 1625395 | 13 | 29 | 0 | 6 | 0 | 1 |
| 311.165281 | 311.165268 | 41329972 | 20 | 24 | 0 | 3 | 0 | 0 |
| 311.186373 | 311.186398 | 3639222 | 17 | 28 | 0 | 5 | 0 | 0 |
| 311.201678 | 311.201654 | 20431160 | 21 | 28 | 0 | 2 | 0 | 0 |
| 311.238058 | 311.238039 | 3046715 | 22 | 32 | 0 | 1 | 0 | 0 |
| 312.051288 | 312.051361 | 1554319 | 16 | 11 | 1 | 6 | 0 | 0 |
| 312.063953 | 312.064247 | 2122896 | 13 | 16 | 1 | 6 | 0 | 1 |
| 312.087757 | 312.087746 | 1970739 | 17 | 15 | 1 | 5 | 0 | 0 |
| 312.124152 | 312.124132 | 2622230 | 18 | 19 | 1 | 4 | 0 | 0 |
| 312.136736 | 312.137018 | 2312984 | 15 | 24 | 1 | 4 | 0 | 1 |
| 312.16051 | 312.160517 | 2134490 | 19 | 23 | 1 | 3 | 0 | 0 |
| 313.035393 | 313.035376 | 5440500 | 16 | 10 | 0 | 7 | 0 | 0 |
| 313.071725 | 313.071762 | 11211255 | 17 | 14 | 0 | 6 | 0 | 0 |
| 313.083069 | 313.082995 | 1893497 | 16 | 14 | 2 | 5 | 0 | 0 |
| 313.087031 | 313.087018 | 1498361 | 21 | 14 | 0 | 3 | 0 | 0 |
| 313.108121 | 313.108147 | 23820796 | 18 | 18 | 0 | 5 | 0 | 0 |
| 313.11148 | 313.111518 | 3095676 | 15 | 22 | 0 | 5 | 1 | 0 |
| 313.119324 | 313.119381 | 2455548 | 17 | 18 | 2 | 4 | 0 | 0 |
| 313.123431 | 313.123403 | 1252029 | 22 | 18 | 0 | 2 | 0 | 0 |
| 313.129336 | 313.129277 | 4405246 | 15 | 22 | 0 | 7 | 0 | 0 |
| 313.1445 | 313.144533 | 46386688 | 19 | 22 | 0 | 4 | 0 | 0 |
| 313.165649 | 313.165662 | 3355137 | 16 | 26 | 0 | 6 | 0 | 0 |
| 313.180905 | 313.180918 | 41543172 | 20 | 26 | 0 | 3 | 0 | 0 |
| 313.217287 | 313.217304 | 12008454 | 21 | 30 | 0 | 2 | 0 | 0 |
| 313.253753 | 313.253689 | 1440394 | 22 | 34 | 0 | 1 | 0 | 0 |
| 314.067024 | 314.067011 | 1593309 | 16 | 13 | 1 | 6 | 0 | 0 |
| 314.103368 | 314.103396 | 1959200 | 17 | 17 | 1 | 5 | 0 | 0 |
| 314.139764 | 314.139782 | 2636900 | 18 | 21 | 1 | 4 | 0 | 0 |
| 314.152366 | 314.152668 | 2143589 | 15 | 26 | 1 | 4 | 0 | 1 |
| 314.176118 | 314.176167 | 2101480 | 19 | 25 | 1 | 3 | 0 | 0 |
| 315.014668 | 315.014641 | 2019708 | 15 | 8 | 0 | 8 | 0 | 0 |
| 315.051008 | 315.051026 | 7459264 | 16 | 12 | 0 | 7 | 0 | 0 |
| 315.066271 | 315.066282 | 1855553 | 20 | 12 | 0 | 4 | 0 | 0 |
| 315.087427 | 315.087412 | 11972803 | 17 | 16 | 0 | 6 | 0 | 0 |
| 315.090771 | 315.090783 | 1754308 | 14 | 20 | 0 | 6 | 1 | 0 |
| 315.09865 | 315.098645 | 2075333 | 16 | 16 | 2 | 5 | 0 | 0 |
| 315.102629 | 315.102668 | 2929605 | 21 | 16 | 0 | 3 | 0 | 0 |
| 315.123807 | 315.123797 | 29826760 | 18 | 20 | 0 | 5 | 0 | 0 |
| 315.127218 | 315.127168 | 4648904 | 15 | 24 | 0 | 5 | 1 | 0 |
| 315.139082 | 315.139053 | 3137993 | 22 | 20 | 0 | 2 | 0 | 0 |
| 315.144886 | 315.144927 | 1579465 | 15 | 24 | 0 | 7 | 0 | 0 |
| 315.16018 | 315.160183 | 44302028 | 19 | 24 | 0 | 4 | 0 | 0 |
| 315.175484 | 315.175439 | 2507724 | 23 | 24 | 0 | 1 | 0 | 0 |
| 315.19655 | 315.196568 | 26085070 | 20 | 28 | 0 | 3 | 0 | 0 |
| 315.232942 | 315.232954 | 3841490 | 21 | 32 | 0 | 2 | 0 | 0 |
| 316.082657 | 316.082661 | 1350055 | 16 | 15 | 1 | 6 | 0 | 0 |
| 316.095263 | 316.095547 | 1341032 | 13 | 20 | 1 | 6 | 0 | 1 |
| 316.119003 | 316.119046 | 2304043 | 17 | 19 | 1 | 5 | 0 | 0 |
| 316.155456 | 316.155432 | 2106542 | 18 | 23 | 1 | 4 | 0 | 0 |
| 317.030274 | 317.030291 | 2426885 | 15 | 10 | 0 | 8 | 0 | 0 |
| 317.066642 | 317.066676 | 6555528 | 16 | 14 | 0 | 7 | 0 | 0 |
| 317.081949 | 317.081932 | 3904394 | 20 | 14 | 0 | 4 | 0 | 0 |
| 317.103019 | 317.103062 | 13257612 | 17 | 18 | 0 | 6 | 0 | 0 |
| 317.106329 | 317.106433 | 1570700 | 14 | 22 | 0 | 6 | 1 | 0 |
| 317.118324 | 317.118318 | 5187726 | 21 | 18 | 0 | 3 | 0 | 0 |
| 317.139448 | 317.139447 | 31766416 | 18 | 22 | 0 | 5 | 0 | 0 |
| 317.142794 | 317.142818 | 3500688 | 15 | 26 | 0 | 5 | 1 | 0 |
| 317.154689 | 317.154703 | 6946705 | 22 | 22 | 0 | 2 | 0 | 0 |
| 317.175851 | 317.175833 | 31688594 | 19 | 26 | 0 | 4 | 0 | 0 |
| 317.191069 | 317.191089 | 4629909 | 23 | 26 | 0 | 1 | 0 | 0 |
| 317.21224 | 317.212218 | 10137495 | 20 | 30 | 0 | 3 | 0 | 0 |
| 318.089757 | 318.090068 | 1450413 | 16 | 18 | 1 | 4 | 0 | 1 |
| 318.134614 | 318.134696 | 1639281 | 17 | 21 | 1 | 5 | 0 | 0 |
| 318.171055 | 318.171082 | 1526005 | 18 | 25 | 1 | 4 | 0 | 0 |
| 319.025005 | 319.024812 | 1282760 | 18 | 8 | 0 | 6 | 0 | 0 |
| 319.045939 | 319.045941 | 3299914 | 15 | 12 | 0 | 8 | 0 | 0 |
| 319.06122 | 319.061197 | 4140875 | 19 | 12 | 0 | 5 | 0 | 0 |
| 319.082377 | 319.082326 | 5599566 | 16 | 16 | 0 | 7 | 0 | 0 |
| 319.097585 | 319.097583 | 7090767 | 20 | 16 | 0 | 4 | 0 | 0 |
| 319.118727 | 319.118712 | 16852048 | 17 | 20 | 0 | 6 | 0 | 0 |
| 319.133962 | 319.133968 | 12557394 | 21 | 20 | 0 | 3 | 0 | 0 |
| 319.155092 | 319.155097 | 29712468 | 18 | 24 | 0 | 5 | 0 | 0 |
| 319.170364 | 319.170354 | 13131862 | 22 | 24 | 0 | 2 | 0 | 0 |
| 319.191453 | 319.191483 | 18089048 | 19 | 28 | 0 | 4 | 0 | 0 |
| 319.206762 | 319.206739 | 5779546 | 23 | 28 | 0 | 1 | 0 | 0 |
| 320.069158 | 320.069333 | 1671341 | 15 | 16 | 1 | 5 | 0 | 1 |
| 320.092842 | 320.092832 | 1797039 | 19 | 15 | 1 | 4 | 0 | 0 |
| 320.105461 | 320.105718 | 2532144 | 16 | 20 | 1 | 4 | 0 | 1 |
| 320.113915 | 320.113961 | 1323953 | 16 | 19 | 1 | 6 | 0 | 0 |
| 320.129149 | 320.129217 | 1509811 | 20 | 19 | 1 | 3 | 0 | 0 |
| 320.150274 | 320.150346 | 1347253 | 17 | 23 | 1 | 5 | 0 | 0 |
| 321.040449 | 321.040462 | 4316170 | 18 | 10 | 0 | 6 | 0 | 0 |
| 321.061609 | 321.061591 | 2529164 | 15 | 14 | 0 | 8 | 0 | 0 |
| 321.076839 | 321.076847 | 7894030 | 19 | 14 | 0 | 5 | 0 | 0 |
| 321.08802 | 321.08808 | 1384846 | 18 | 14 | 2 | 4 | 0 | 0 |
| 321.098013 | 321.097976 | 6235920 | 16 | 18 | 0 | 7 | 0 | 0 |
| 321.113247 | 321.113233 | 14181649 | 20 | 18 | 0 | 4 | 0 | 0 |
| 321.124382 | 321.124466 | 1687826 | 19 | 18 | 2 | 3 | 0 | 0 |
| 321.134349 | 321.134362 | 19321106 | 17 | 22 | 0 | 6 | 0 | 0 |
| 321.149654 | 321.149618 | 23227668 | 21 | 22 | 0 | 3 | 0 | 0 |
| 321.170709 | 321.170747 | 22972694 | 18 | 26 | 0 | 5 | 0 | 0 |
| 321.18601 | 321.186004 | 20222232 | 22 | 26 | 0 | 2 | 0 | 0 |
| 321.222344 | 321.222389 | 6292763 | 23 | 30 | 0 | 1 | 0 | 0 |
| 322.04829 | 322.048597 | 1737066 | 14 | 14 | 1 | 6 | 0 | 1 |
| 322.072123 | 322.072096 | 1652012 | 18 | 13 | 1 | 5 | 0 | 0 |
| 322.084673 | 322.084983 | 2226029 | 15 | 18 | 1 | 5 | 0 | 1 |
| 322.108497 | 322.108482 | 1896911 | 19 | 17 | 1 | 4 | 0 | 0 |
| 322.121081 | 322.121368 | 2846065 | 16 | 22 | 1 | 4 | 0 | 1 |
| 322.144812 | 322.144867 | 2325107 | 20 | 21 | 1 | 3 | 0 | 0 |
| 323.019797 | 323.019726 | 2077765 | 17 | 8 | 0 | 7 | 0 | 0 |
| 323.056132 | 323.056112 | 6725833 | 18 | 12 | 0 | 6 | 0 | 0 |
| 323.077244 | 323.077241 | 2247115 | 15 | 16 | 0 | 8 | 0 | 0 |
| 323.092452 | 323.092497 | 12901836 | 19 | 16 | 0 | 5 | 0 | 0 |
| 323.103761 | 323.103731 | 1780685 | 18 | 16 | 2 | 4 | 0 | 0 |
| 323.113615 | 323.113627 | 7681486 | 16 | 20 | 0 | 7 | 0 | 0 |
| 323.128878 | 323.128883 | 24539600 | 20 | 20 | 0 | 4 | 0 | 0 |
| 323.149988 | 323.150012 | 18037202 | 17 | 24 | 0 | 6 | 0 | 0 |
| 323.165293 | 323.165268 | 34533844 | 21 | 24 | 0 | 3 | 0 | 0 |
| 323.168673 | 323.168639 | 1801427 | 18 | 28 | 0 | 3 | 1 | 0 |
| 323.186367 | 323.186398 | 10951125 | 18 | 28 | 0 | 5 | 0 | 0 |
| 323.201623 | 323.201654 | 22062550 | 22 | 28 | 0 | 2 | 0 | 0 |
| 323.22275 | 323.222783 | 2228569 | 19 | 32 | 0 | 4 | 0 | 0 |
| 323.238062 | 323.238039 | 4403930 | 23 | 32 | 0 | 1 | 0 | 0 |
| 324.05124 | 324.051361 | 1483942 | 17 | 11 | 1 | 6 | 0 | 0 |
| 324.064017 | 324.064247 | 1759783 | 14 | 16 | 1 | 6 | 0 | 1 |
| 324.087696 | 324.087746 | 2429482 | 18 | 15 | 1 | 5 | 0 | 0 |
| 324.100353 | 324.100633 | 2295467 | 15 | 20 | 1 | 5 | 0 | 1 |
| 324.124213 | 324.124132 | 2627885 | 19 | 19 | 1 | 4 | 0 | 0 |
| 324.136721 | 324.137018 | 3255086 | 16 | 24 | 1 | 4 | 0 | 1 |
| 324.160547 | 324.160517 | 1952880 | 20 | 23 | 1 | 3 | 0 | 0 |
| 324.17316 | 324.173404 | 1543218 | 17 | 28 | 1 | 3 | 0 | 1 |
| 325.035368 | 325.035376 | 4652674 | 17 | 10 | 0 | 7 | 0 | 0 |
| 325.071758 | 325.071762 | 9988229 | 18 | 14 | 0 | 6 | 0 | 0 |
| 325.083018 | 325.082995 | 1647366 | 17 | 14 | 2 | 5 | 0 | 0 |
| 325.0929 | 325.092891 | 1855111 | 15 | 18 | 0 | 8 | 0 | 0 |
| 325.108151 | 325.108147 | 18395784 | 19 | 18 | 0 | 5 | 0 | 0 |
| 325.119421 | 325.119381 | 2134666 | 18 | 18 | 2 | 4 | 0 | 0 |
| 325.129277 | 325.129277 | 7723659 | 16 | 22 | 0 | 7 | 0 | 0 |
| 325.144518 | 325.144533 | 35416716 | 20 | 22 | 0 | 4 | 0 | 0 |
| 325.14784 | 325.147904 | 1579020 | 17 | 26 | 0 | 4 | 1 | 0 |
| 325.165672 | 325.165662 | 8878734 | 17 | 26 | 0 | 6 | 0 | 0 |
| 325.180931 | 325.180918 | 36407952 | 21 | 26 | 0 | 3 | 0 | 0 |
| 325.217341 | 325.217304 | 16382611 | 22 | 30 | 0 | 2 | 0 | 0 |
| 325.253668 | 325.253689 | 1973014 | 23 | 34 | 0 | 1 | 0 | 0 |
| 326.066931 | 326.067011 | 1523041 | 17 | 13 | 1 | 6 | 0 | 0 |
| 326.079635 | 326.079897 | 1832674 | 14 | 18 | 1 | 6 | 0 | 1 |
| 326.103366 | 326.103396 | 2636772 | 18 | 17 | 1 | 5 | 0 | 0 |
| 326.139704 | 326.139782 | 2746600 | 19 | 21 | 1 | 4 | 0 | 0 |
| 326.17626 | 326.176167 | 1832299 | 20 | 25 | 1 | 3 | 0 | 0 |
| 327.014631 | 327.014641 | 2107064 | 16 | 8 | 0 | 8 | 0 | 0 |
| 327.05106 | 327.051026 | 6214716 | 17 | 12 | 0 | 7 | 0 | 0 |
| 327.087405 | 327.087412 | 12442431 | 18 | 16 | 0 | 6 | 0 | 0 |
| 327.090761 | 327.090783 | 2545471 | 15 | 20 | 0 | 6 | 1 | 0 |
| 327.098558 | 327.098645 | 2015168 | 17 | 16 | 2 | 5 | 0 | 0 |
| 327.10257 | 327.102668 | 1824960 | 22 | 16 | 0 | 3 | 0 | 0 |
| 327.108466 | 327.108541 | 1854913 | 15 | 20 | 0 | 8 | 0 | 0 |
| 327.123779 | 327.123797 | 27827010 | 19 | 20 | 0 | 5 | 0 | 0 |
| 327.127102 | 327.127168 | 1328194 | 16 | 24 | 0 | 5 | 1 | 0 |
| 327.135003 | 327.135031 | 1471939 | 18 | 20 | 2 | 4 | 0 | 0 |
| 327.139044 | 327.139053 | 1790531 | 23 | 20 | 0 | 2 | 0 | 0 |
| 327.144947 | 327.144927 | 4157764 | 16 | 24 | 0 | 7 | 0 | 0 |
| 327.160207 | 327.160183 | 42285892 | 20 | 24 | 0 | 4 | 0 | 0 |
| 327.181265 | 327.181312 | 2396103 | 17 | 28 | 0 | 6 | 0 | 0 |
| 327.196572 | 327.196568 | 29256520 | 21 | 28 | 0 | 3 | 0 | 0 |
| 327.232948 | 327.232954 | 7857996 | 22 | 32 | 0 | 2 | 0 | 0 |
| 328.119075 | 328.119046 | 2462493 | 18 | 19 | 1 | 5 | 0 | 0 |
| 328.122445 | 328.122417 | 1974557 | 15 | 23 | 1 | 5 | 1 | 0 |
| 328.131704 | 328.131933 | 1784862 | 15 | 24 | 1 | 5 | 0 | 1 |
| 328.155374 | 328.155432 | 2742944 | 19 | 23 | 1 | 4 | 0 | 0 |
| 329.030255 | 329.030291 | 3651055 | 16 | 10 | 0 | 8 | 0 | 0 |
| 329.066663 | 329.066676 | 7326195 | 17 | 14 | 0 | 7 | 0 | 0 |
| 329.081984 | 329.081932 | 2699508 | 21 | 14 | 0 | 4 | 0 | 0 |
| 329.103058 | 329.103062 | 15894518 | 18 | 18 | 0 | 6 | 0 | 0 |
| 329.106374 | 329.106433 | 5229046 | 15 | 22 | 0 | 6 | 1 | 0 |
| 329.11423 | 329.114295 | 1763959 | 17 | 18 | 2 | 5 | 0 | 0 |
| 329.118326 | 329.118318 | 3795959 | 22 | 18 | 0 | 3 | 0 | 0 |
| 329.139471 | 329.139447 | 34548728 | 19 | 22 | 0 | 5 | 0 | 0 |
| 329.154731 | 329.154703 | 4124410 | 23 | 22 | 0 | 2 | 0 | 0 |
| 329.160604 | 329.160577 | 1434875 | 16 | 26 | 0 | 7 | 0 | 0 |
| 329.175833 | 329.175833 | 38677500 | 20 | 26 | 0 | 4 | 0 | 0 |
| 329.191114 | 329.191089 | 2459134 | 24 | 26 | 0 | 1 | 0 | 0 |
| 329.212197 | 329.212218 | 16019456 | 21 | 30 | 0 | 3 | 0 | 0 |
| 329.248587 | 329.248604 | 3482115 | 22 | 34 | 0 | 2 | 0 | 0 |
| 330.126138 | 330.126454 | 1298002 | 18 | 22 | 1 | 3 | 0 | 1 |
| 330.134756 | 330.134696 | 2296147 | 18 | 21 | 1 | 5 | 0 | 0 |
| 330.171022 | 330.171082 | 1994710 | 19 | 25 | 1 | 4 | 0 | 0 |
| 331.045933 | 331.045941 | 3321252 | 16 | 12 | 0 | 8 | 0 | 0 |
| 331.061179 | 331.061197 | 3030437 | 20 | 12 | 0 | 5 | 0 | 0 |
| 331.082288 | 331.082326 | 9162919 | 17 | 16 | 0 | 7 | 0 | 0 |
| 331.085679 | 331.085697 | 1345448 | 14 | 20 | 0 | 7 | 1 | 0 |
| 331.097581 | 331.097583 | 5092009 | 21 | 16 | 0 | 4 | 0 | 0 |
| 331.101017 | 331.100953 | 1403945 | 18 | 20 | 0 | 4 | 1 | 0 |
| 331.118683 | 331.118712 | 19085482 | 18 | 20 | 0 | 6 | 0 | 0 |
| 331.121994 | 331.122083 | 6672043 | 15 | 24 | 0 | 6 | 1 | 0 |
| 331.133956 | 331.133968 | 8155308 | 22 | 20 | 0 | 3 | 0 | 0 |
| 331.155117 | 331.155097 | 36293804 | 19 | 24 | 0 | 5 | 0 | 0 |
| 331.170334 | 331.170354 | 8323759 | 23 | 24 | 0 | 2 | 0 | 0 |
| 331.19151 | 331.191483 | 29660336 | 20 | 28 | 0 | 4 | 0 | 0 |
| 331.206744 | 331.206739 | 3881138 | 24 | 28 | 0 | 1 | 0 | 0 |
| 331.227847 | 331.227868 | 6222516 | 21 | 32 | 0 | 3 | 0 | 0 |
| 332.069016 | 332.069333 | 1626367 | 16 | 16 | 1 | 5 | 0 | 1 |
| 332.092705 | 332.092832 | 1468673 | 20 | 15 | 1 | 4 | 0 | 0 |
| 332.12926 | 332.129217 | 1559428 | 21 | 19 | 1 | 3 | 0 | 0 |
| 332.150343 | 332.150346 | 1848966 | 18 | 23 | 1 | 5 | 0 | 0 |
| 333.04045 | 333.040462 | 3350741 | 19 | 10 | 0 | 6 | 0 | 0 |
| 333.061521 | 333.061591 | 5263703 | 16 | 14 | 0 | 8 | 0 | 0 |
| 333.076862 | 333.076847 | 5405528 | 20 | 14 | 0 | 5 | 0 | 0 |
| 333.097994 | 333.097976 | 8115034 | 17 | 18 | 0 | 7 | 0 | 0 |
| 333.113179 | 333.113233 | 9146715 | 21 | 18 | 0 | 4 | 0 | 0 |
| 333.134369 | 333.134362 | 21800286 | 18 | 22 | 0 | 6 | 0 | 0 |
| 333.137809 | 333.137733 | 3697758 | 15 | 26 | 0 | 6 | 1 | 0 |
| 333.149623 | 333.149618 | 14592350 | 22 | 22 | 0 | 3 | 0 | 0 |
| 333.170718 | 333.170747 | 30565728 | 19 | 26 | 0 | 5 | 0 | 0 |
| 333.186016 | 333.186004 | 13936994 | 23 | 26 | 0 | 2 | 0 | 0 |
| 333.207141 | 333.207133 | 16564580 | 20 | 30 | 0 | 4 | 0 | 0 |
| 333.222358 | 333.222389 | 4606565 | 24 | 30 | 0 | 1 | 0 | 0 |
| 333.243543 | 333.243519 | 1378151 | 21 | 34 | 0 | 3 | 0 | 0 |
| 334.071983 | 334.072096 | 1526320 | 19 | 13 | 1 | 5 | 0 | 0 |
| 334.084784 | 334.084983 | 1748273 | 16 | 18 | 1 | 5 | 0 | 1 |
| 334.108546 | 334.108482 | 1383219 | 20 | 17 | 1 | 4 | 0 | 0 |
| 334.121038 | 334.121368 | 2343988 | 17 | 22 | 1 | 4 | 0 | 1 |
| 334.129756 | 334.129611 | 1341877 | 17 | 21 | 1 | 6 | 0 | 0 |
| 334.144806 | 334.144867 | 1703350 | 21 | 21 | 1 | 3 | 0 | 0 |
| 335.019673 | 335.019726 | 1695491 | 18 | 8 | 0 | 7 | 0 | 0 |
| 335.056119 | 335.056112 | 5629446 | 19 | 12 | 0 | 6 | 0 | 0 |
| 335.077185 | 335.077241 | 3033096 | 16 | 16 | 0 | 8 | 0 | 0 |
| 335.092461 | 335.092497 | 9143305 | 20 | 16 | 0 | 5 | 0 | 0 |
| 335.103857 | 335.103731 | 1304906 | 19 | 16 | 2 | 4 | 0 | 0 |
| 335.113651 | 335.113627 | 8514571 | 17 | 20 | 0 | 7 | 0 | 0 |
| 335.128872 | 335.128883 | 16712204 | 21 | 20 | 0 | 4 | 0 | 0 |
| 335.140038 | 335.140116 | 1362061 | 20 | 20 | 2 | 3 | 0 | 0 |
| 335.149984 | 335.150012 | 23389710 | 18 | 24 | 0 | 6 | 0 | 0 |
| 335.165292 | 335.165268 | 24476176 | 22 | 24 | 0 | 3 | 0 | 0 |
| 335.186421 | 335.186398 | 18696722 | 19 | 28 | 0 | 5 | 0 | 0 |
| 335.201616 | 335.201654 | 18245138 | 23 | 28 | 0 | 2 | 0 | 0 |
| 335.226274 | 335.226154 | 1601173 | 17 | 36 | 0 | 4 | 1 | 0 |
| 335.238023 | 335.238039 | 4284438 | 24 | 32 | 0 | 1 | 0 | 0 |
| 336.087652 | 336.087746 | 1613535 | 19 | 15 | 1 | 5 | 0 | 0 |
| 336.1003 | 336.100633 | 1999585 | 16 | 20 | 1 | 5 | 0 | 1 |
| 336.124136 | 336.124132 | 1886179 | 20 | 19 | 1 | 4 | 0 | 0 |
| 336.136759 | 336.137018 | 2958692 | 17 | 24 | 1 | 4 | 0 | 1 |
| 336.160522 | 336.160517 | 1668838 | 21 | 23 | 1 | 3 | 0 | 0 |
| 336.173215 | 336.173404 | 1305639 | 18 | 28 | 1 | 3 | 0 | 1 |
| 337.035393 | 337.035376 | 3810738 | 18 | 10 | 0 | 7 | 0 | 0 |
| 337.071792 | 337.071762 | 8232117 | 19 | 14 | 0 | 6 | 0 | 0 |
| 337.092896 | 337.092891 | 2705975 | 16 | 18 | 0 | 8 | 0 | 0 |
| 337.108122 | 337.108147 | 13334200 | 20 | 18 | 0 | 5 | 0 | 0 |
| 337.119476 | 337.119381 | 1625465 | 19 | 18 | 2 | 4 | 0 | 0 |
| 337.129317 | 337.129277 | 11495098 | 17 | 22 | 0 | 7 | 0 | 0 |
| 337.144522 | 337.144533 | 26404538 | 21 | 22 | 0 | 4 | 0 | 0 |
| 337.155739 | 337.155766 | 1382844 | 20 | 22 | 2 | 3 | 0 | 0 |
| 337.165652 | 337.165662 | 16462525 | 18 | 26 | 0 | 6 | 0 | 0 |
| 337.18093 | 337.180918 | 31694526 | 22 | 26 | 0 | 3 | 0 | 0 |
| 337.217277 | 337.217304 | 16834242 | 23 | 30 | 0 | 2 | 0 | 0 |
| 337.253721 | 337.253689 | 2438468 | 24 | 34 | 0 | 1 | 0 | 0 |
| 338.043198 | 338.043512 | 1351432 | 14 | 14 | 1 | 7 | 0 | 1 |
| 338.067024 | 338.067011 | 1521803 | 18 | 13 | 1 | 6 | 0 | 0 |
| 338.103389 | 338.103396 | 2344718 | 19 | 17 | 1 | 5 | 0 | 0 |
| 338.116024 | 338.116283 | 1721551 | 16 | 22 | 1 | 5 | 0 | 1 |
| 338.139711 | 338.139782 | 2429713 | 20 | 21 | 1 | 4 | 0 | 0 |
| 338.152433 | 338.152668 | 2243474 | 17 | 26 | 1 | 4 | 0 | 1 |
| 338.176195 | 338.176167 | 1933588 | 21 | 25 | 1 | 3 | 0 | 0 |
| 339.014619 | 339.014641 | 1568412 | 17 | 8 | 0 | 8 | 0 | 0 |
| 339.051008 | 339.051026 | 5700959 | 18 | 12 | 0 | 7 | 0 | 0 |
| 339.087369 | 339.087412 | 10756450 | 19 | 16 | 0 | 6 | 0 | 0 |
| 339.090743 | 339.090783 | 1287394 | 16 | 20 | 0 | 6 | 1 | 0 |
| 339.0987 | 339.098645 | 1615075 | 18 | 16 | 2 | 5 | 0 | 0 |
| 339.108561 | 339.108541 | 2958948 | 16 | 20 | 0 | 8 | 0 | 0 |
| 339.123834 | 339.123797 | 20200294 | 20 | 20 | 0 | 5 | 0 | 0 |
| 339.135194 | 339.135031 | 1642662 | 19 | 20 | 2 | 4 | 0 | 0 |
| 339.144883 | 339.144927 | 9467751 | 17 | 24 | 0 | 7 | 0 | 0 |
| 339.160159 | 339.160183 | 36962152 | 21 | 24 | 0 | 4 | 0 | 0 |
| 339.196565 | 339.196568 | 30822252 | 22 | 28 | 0 | 3 | 0 | 0 |
| 339.232972 | 339.232954 | 12254063 | 23 | 32 | 0 | 2 | 0 | 0 |
| 340.082648 | 340.082661 | 1509943 | 18 | 15 | 1 | 6 | 0 | 0 |
| 340.095405 | 340.095547 | 1638712 | 15 | 20 | 1 | 6 | 0 | 1 |
| 340.118961 | 340.119046 | 2499002 | 19 | 19 | 1 | 5 | 0 | 0 |
| 340.155419 | 340.155432 | 2586557 | 20 | 23 | 1 | 4 | 0 | 0 |
| 341.030338 | 341.030291 | 3468552 | 17 | 10 | 0 | 8 | 0 | 0 |
| 341.066651 | 341.066676 | 7327243 | 18 | 14 | 0 | 7 | 0 | 0 |
| 341.0703 | 341.070047 | 1329163 | 15 | 18 | 0 | 7 | 1 | 0 |
| 341.081979 | 341.081932 | 1284236 | 22 | 14 | 0 | 4 | 0 | 0 |
| 341.103032 | 341.103062 | 15623182 | 19 | 18 | 0 | 6 | 0 | 0 |
| 341.106399 | 341.106433 | 1667406 | 16 | 22 | 0 | 6 | 1 | 0 |
| 341.114289 | 341.114295 | 2267151 | 18 | 18 | 2 | 5 | 0 | 0 |
| 341.118213 | 341.118318 | 2359823 | 23 | 18 | 0 | 3 | 0 | 0 |
| 341.124216 | 341.124191 | 2684432 | 16 | 22 | 0 | 8 | 0 | 0 |
| 341.139454 | 341.139447 | 30343186 | 20 | 22 | 0 | 5 | 0 | 0 |
| 341.154727 | 341.154703 | 2317971 | 24 | 22 | 0 | 2 | 0 | 0 |
| 341.160541 | 341.160577 | 4141587 | 17 | 26 | 0 | 7 | 0 | 0 |
| 341.175815 | 341.175833 | 39583764 | 21 | 26 | 0 | 4 | 0 | 0 |
| 341.191225 | 341.191089 | 1302550 | 25 | 26 | 0 | 1 | 0 | 0 |
| 341.196797 | 341.196962 | 1738390 | 18 | 30 | 0 | 6 | 0 | 0 |
| 341.212226 | 341.212218 | 22880280 | 22 | 30 | 0 | 3 | 0 | 0 |
| 341.24856 | 341.248604 | 5475867 | 23 | 34 | 0 | 2 | 0 | 0 |
| 342.098362 | 342.098311 | 1861091 | 18 | 17 | 1 | 6 | 0 | 0 |
| 342.134799 | 342.134696 | 2415846 | 19 | 21 | 1 | 5 | 0 | 0 |
| 342.147325 | 342.147583 | 1522919 | 16 | 26 | 1 | 5 | 0 | 1 |
| 342.170969 | 342.171082 | 2010217 | 20 | 25 | 1 | 4 | 0 | 0 |
| 343.045934 | 343.045941 | 4562611 | 17 | 12 | 0 | 8 | 0 | 0 |
| 343.06114 | 343.061197 | 2261684 | 21 | 12 | 0 | 5 | 0 | 0 |
| 343.082292 | 343.082326 | 8550070 | 18 | 16 | 0 | 7 | 0 | 0 |
| 343.085787 | 343.085697 | 2284278 | 15 | 20 | 0 | 7 | 1 | 0 |
| 343.097637 | 343.097583 | 3272375 | 22 | 16 | 0 | 4 | 0 | 0 |
| 343.118674 | 343.118712 | 22300856 | 19 | 20 | 0 | 6 | 0 | 0 |
| 343.121946 | 343.122083 | 2041273 | 16 | 24 | 0 | 6 | 1 | 0 |
| 343.133939 | 343.133968 | 4855994 | 23 | 20 | 0 | 3 | 0 | 0 |
| 343.13985 | 343.139841 | 1680059 | 16 | 24 | 0 | 8 | 0 | 0 |
| 343.155117 | 343.155097 | 34631868 | 20 | 24 | 0 | 5 | 0 | 0 |
| 343.17029 | 343.170354 | 4888765 | 24 | 24 | 0 | 2 | 0 | 0 |
| 343.176144 | 343.176227 | 1420094 | 17 | 28 | 0 | 7 | 0 | 0 |
| 343.191462 | 343.191483 | 31201470 | 21 | 28 | 0 | 4 | 0 | 0 |
| 343.206725 | 343.206739 | 2553536 | 25 | 28 | 0 | 1 | 0 | 0 |
| 343.227833 | 343.227868 | 11350210 | 22 | 32 | 0 | 3 | 0 | 0 |
| 343.248901 | 343.248998 | 2512324 | 19 | 36 | 0 | 5 | 0 | 0 |
| 343.264475 | 343.264254 | 1538117 | 23 | 36 | 0 | 2 | 0 | 0 |
| 344.105461 | 344.105718 | 1287948 | 18 | 20 | 1 | 4 | 0 | 1 |
| 344.11401 | 344.113961 | 1475405 | 18 | 19 | 1 | 6 | 0 | 0 |
| 344.117401 | 344.117332 | 1502989 | 15 | 23 | 1 | 6 | 1 | 0 |
| 344.150377 | 344.150346 | 1842000 | 19 | 23 | 1 | 5 | 0 | 0 |
| 344.186766 | 344.186732 | 1618067 | 20 | 27 | 1 | 4 | 0 | 0 |
| 345.025097 | 345.025205 | 1365593 | 16 | 10 | 0 | 9 | 0 | 0 |
| 345.040328 | 345.040462 | 1594971 | 20 | 10 | 0 | 6 | 0 | 0 |
| 345.061624 | 345.061591 | 4671836 | 17 | 14 | 0 | 8 | 0 | 0 |
| 345.076943 | 345.076847 | 3813726 | 21 | 14 | 0 | 5 | 0 | 0 |
| 345.09797 | 345.097976 | 8534367 | 18 | 18 | 0 | 7 | 0 | 0 |
| 345.101305 | 345.101347 | 4348000 | 15 | 22 | 0 | 7 | 1 | 0 |
| 345.113232 | 345.113233 | 6879585 | 22 | 18 | 0 | 4 | 0 | 0 |
| 345.124405 | 345.124466 | 1337314 | 21 | 18 | 2 | 3 | 0 | 0 |
| 345.134372 | 345.134362 | 24908130 | 19 | 22 | 0 | 6 | 0 | 0 |
| 345.137704 | 345.137733 | 1660515 | 16 | 26 | 0 | 6 | 1 | 0 |
| 345.149622 | 345.149618 | 8834916 | 23 | 22 | 0 | 3 | 0 | 0 |
| 345.170741 | 345.170747 | 32940390 | 20 | 26 | 0 | 5 | 0 | 0 |
| 345.186004 | 345.186004 | 8271207 | 24 | 26 | 0 | 2 | 0 | 0 |
| 345.207114 | 345.207133 | 16872808 | 21 | 30 | 0 | 4 | 0 | 0 |
| 345.222434 | 345.222389 | 3153002 | 25 | 30 | 0 | 1 | 0 | 0 |
| 345.2435 | 345.243519 | 4372332 | 22 | 34 | 0 | 3 | 0 | 0 |
| 346.072106 | 346.072096 | 1326833 | 20 | 13 | 1 | 5 | 0 | 0 |
| 346.108531 | 346.108482 | 1674676 | 21 | 17 | 1 | 4 | 0 | 0 |
| 346.121142 | 346.121368 | 1926453 | 18 | 22 | 1 | 4 | 0 | 1 |
| 346.129576 | 346.129611 | 1796534 | 18 | 21 | 1 | 6 | 0 | 0 |
| 346.144887 | 346.144867 | 1418935 | 22 | 21 | 1 | 3 | 0 | 0 |
| 346.157449 | 346.157754 | 1398968 | 19 | 26 | 1 | 3 | 0 | 1 |
| 346.165918 | 346.165996 | 1742137 | 19 | 25 | 1 | 5 | 0 | 0 |
| 347.040762 | 347.040856 | 1646850 | 16 | 12 | 0 | 9 | 0 | 0 |
| 347.056099 | 347.056112 | 4088067 | 20 | 12 | 0 | 6 | 0 | 0 |
| 347.077256 | 347.077241 | 4230661 | 17 | 16 | 0 | 8 | 0 | 0 |
| 347.092512 | 347.092497 | 6636294 | 21 | 16 | 0 | 5 | 0 | 0 |
| 347.113588 | 347.113627 | 10727944 | 18 | 20 | 0 | 7 | 0 | 0 |
| 347.116998 | 347.116997 | 3291528 | 15 | 24 | 0 | 7 | 1 | 0 |
| 347.128857 | 347.128883 | 10462217 | 22 | 20 | 0 | 4 | 0 | 0 |
| 347.150034 | 347.150012 | 23387660 | 19 | 24 | 0 | 6 | 0 | 0 |
| 347.153332 | 347.153383 | 1406283 | 16 | 28 | 0 | 6 | 1 | 0 |
| 347.165236 | 347.165268 | 15455756 | 23 | 24 | 0 | 3 | 0 | 0 |
| 347.186375 | 347.186398 | 25049614 | 20 | 28 | 0 | 5 | 0 | 0 |
| 347.201683 | 347.201654 | 11620879 | 24 | 28 | 0 | 2 | 0 | 0 |
| 347.222785 | 347.222783 | 7289361 | 21 | 32 | 0 | 4 | 0 | 0 |
| 347.237996 | 347.238039 | 3466515 | 25 | 32 | 0 | 1 | 0 | 0 |
| 348.063948 | 348.064247 | 1648727 | 16 | 16 | 1 | 6 | 0 | 1 |
| 348.087829 | 348.087746 | 1822681 | 20 | 15 | 1 | 5 | 0 | 0 |
| 348.100319 | 348.100633 | 1860058 | 17 | 20 | 1 | 5 | 0 | 1 |
| 348.124309 | 348.124132 | 1695964 | 21 | 19 | 1 | 4 | 0 | 0 |
| 348.136712 | 348.137018 | 2360669 | 18 | 24 | 1 | 4 | 0 | 1 |
| 348.160495 | 348.160517 | 1352415 | 22 | 23 | 1 | 3 | 0 | 0 |
| 349.035363 | 349.035376 | 3092904 | 19 | 10 | 0 | 7 | 0 | 0 |
| 349.071771 | 349.071762 | 6368427 | 20 | 14 | 0 | 6 | 0 | 0 |
| 349.092863 | 349.092891 | 3319725 | 17 | 18 | 0 | 8 | 0 | 0 |
| 349.108108 | 349.108147 | 10312366 | 21 | 18 | 0 | 5 | 0 | 0 |
| 349.11926 | 349.119381 | 2033071 | 20 | 18 | 2 | 4 | 0 | 0 |
| 349.129281 | 349.129277 | 12149424 | 18 | 22 | 0 | 7 | 0 | 0 |
| 349.13263 | 349.132647 | 1897136 | 15 | 26 | 0 | 7 | 1 | 0 |
| 349.144552 | 349.144533 | 17989298 | 22 | 22 | 0 | 4 | 0 | 0 |
| 349.165627 | 349.165662 | 23483060 | 19 | 26 | 0 | 6 | 0 | 0 |
| 349.180913 | 349.180918 | 22241972 | 23 | 26 | 0 | 3 | 0 | 0 |
| 349.217267 | 349.217304 | 13303479 | 24 | 30 | 0 | 2 | 0 | 0 |
| 349.238525 | 349.238433 | 2300601 | 21 | 34 | 0 | 4 | 0 | 0 |
| 349.253741 | 349.253689 | 2959290 | 25 | 34 | 0 | 1 | 0 | 0 |
| 350.043179 | 350.043512 | 1318268 | 15 | 14 | 1 | 7 | 0 | 1 |
| 350.103256 | 350.103396 | 1589889 | 20 | 17 | 1 | 5 | 0 | 0 |
| 350.13988 | 350.139782 | 1656260 | 21 | 21 | 1 | 4 | 0 | 0 |
| 350.152441 | 350.152668 | 2159749 | 18 | 26 | 1 | 4 | 0 | 1 |
| 350.176154 | 350.176167 | 1294855 | 22 | 25 | 1 | 3 | 0 | 0 |
| 351.014577 | 351.014641 | 1754445 | 18 | 8 | 0 | 8 | 0 | 0 |
| 351.050954 | 351.051026 | 5257552 | 19 | 12 | 0 | 7 | 0 | 0 |
| 351.087439 | 351.087412 | 9740115 | 20 | 16 | 0 | 6 | 0 | 0 |
| 351.098723 | 351.098645 | 1394900 | 19 | 16 | 2 | 5 | 0 | 0 |
| 351.10853 | 351.108541 | 3764821 | 17 | 20 | 0 | 8 | 0 | 0 |
| 351.12377 | 351.123797 | 15729494 | 21 | 20 | 0 | 5 | 0 | 0 |
| 351.135146 | 351.135031 | 1968087 | 20 | 20 | 2 | 4 | 0 | 0 |
| 351.1449 | 351.144927 | 14653271 | 18 | 24 | 0 | 7 | 0 | 0 |
| 351.160212 | 351.160183 | 27960152 | 22 | 24 | 0 | 4 | 0 | 0 |
| 351.181308 | 351.181312 | 15266650 | 19 | 28 | 0 | 6 | 0 | 0 |
| 351.196555 | 351.196568 | 27355996 | 23 | 28 | 0 | 3 | 0 | 0 |
| 351.22111 | 351.221069 | 1358174 | 17 | 36 | 0 | 5 | 1 | 0 |
| 351.23298 | 351.232954 | 12363615 | 24 | 32 | 0 | 2 | 0 | 0 |
| 351.269388 | 351.269339 | 1947490 | 25 | 36 | 0 | 1 | 0 | 0 |
| 352.082633 | 352.082661 | 1634085 | 19 | 15 | 1 | 6 | 0 | 0 |
| 352.095202 | 352.095547 | 1455334 | 16 | 20 | 1 | 6 | 0 | 1 |
| 352.119167 | 352.119046 | 2344616 | 20 | 19 | 1 | 5 | 0 | 0 |
| 352.131707 | 352.131933 | 1412073 | 17 | 24 | 1 | 5 | 0 | 1 |
| 352.155465 | 352.155432 | 2465707 | 21 | 23 | 1 | 4 | 0 | 0 |
| 352.167991 | 352.168318 | 1759340 | 18 | 28 | 1 | 4 | 0 | 1 |
| 352.191858 | 352.191817 | 2260270 | 22 | 27 | 1 | 3 | 0 | 0 |
| 353.030273 | 353.030291 | 3503604 | 18 | 10 | 0 | 8 | 0 | 0 |
| 353.066644 | 353.066676 | 6722039 | 19 | 14 | 0 | 7 | 0 | 0 |
| 353.08209 | 353.081932 | 1321336 | 23 | 14 | 0 | 4 | 0 | 0 |
| 353.103045 | 353.103062 | 11516922 | 20 | 18 | 0 | 6 | 0 | 0 |
| 353.114185 | 353.114295 | 2040315 | 19 | 18 | 2 | 5 | 0 | 0 |
| 353.124134 | 353.124191 | 5425660 | 17 | 22 | 0 | 8 | 0 | 0 |
| 353.139456 | 353.139447 | 23131132 | 21 | 22 | 0 | 5 | 0 | 0 |
| 353.142859 | 353.142818 | 1358973 | 18 | 26 | 0 | 5 | 1 | 0 |
| 353.154952 | 353.154703 | 1733118 | 25 | 22 | 0 | 2 | 0 | 0 |
| 353.160571 | 353.160577 | 10418174 | 18 | 26 | 0 | 7 | 0 | 0 |
| 353.175803 | 353.175833 | 34548736 | 22 | 26 | 0 | 4 | 0 | 0 |
| 353.179143 | 353.179204 | 2716928 | 19 | 30 | 0 | 4 | 1 | 0 |
| 353.212244 | 353.212218 | 25015298 | 23 | 30 | 0 | 3 | 0 | 0 |
| 353.248548 | 353.248604 | 7631366 | 24 | 34 | 0 | 2 | 0 | 0 |
| 354.09824 | 354.098311 | 1715404 | 19 | 17 | 1 | 6 | 0 | 0 |
| 354.134682 | 354.134696 | 2469711 | 20 | 21 | 1 | 5 | 0 | 0 |
| 354.147279 | 354.147583 | 1406768 | 17 | 26 | 1 | 5 | 0 | 1 |
| 354.171032 | 354.171082 | 1902930 | 21 | 25 | 1 | 4 | 0 | 0 |
| 355.009425 | 355.009555 | 1294872 | 17 | 8 | 0 | 9 | 0 | 0 |
| 355.045927 | 355.045941 | 4206747 | 18 | 12 | 0 | 8 | 0 | 0 |
| 355.082333 | 355.082326 | 8655005 | 19 | 16 | 0 | 7 | 0 | 0 |
| 355.086009 | 355.085697 | 1458590 | 16 | 20 | 0 | 7 | 1 | 0 |
| 355.097386 | 355.097583 | 1856927 | 23 | 16 | 0 | 4 | 0 | 0 |
| 355.11871 | 355.118712 | 15326368 | 20 | 20 | 0 | 6 | 0 | 0 |
| 355.122052 | 355.122083 | 1832225 | 17 | 24 | 0 | 6 | 1 | 0 |
| 355.130138 | 355.129945 | 1695393 | 19 | 20 | 2 | 5 | 0 | 0 |
| 355.133866 | 355.133968 | 2361762 | 24 | 20 | 0 | 3 | 0 | 0 |
| 355.139829 | 355.139841 | 2918946 | 17 | 24 | 0 | 8 | 0 | 0 |
| 355.155102 | 355.155097 | 30524580 | 21 | 24 | 0 | 5 | 0 | 0 |
| 355.158349 | 355.158468 | 1370276 | 18 | 28 | 0 | 5 | 1 | 0 |
| 355.166512 | 355.166331 | 1320356 | 20 | 24 | 2 | 4 | 0 | 0 |
| 355.170367 | 355.170354 | 2371493 | 25 | 24 | 0 | 2 | 0 | 0 |
| 355.17622 | 355.176227 | 4539045 | 18 | 28 | 0 | 7 | 0 | 0 |
| 355.19148 | 355.191483 | 33559720 | 22 | 28 | 0 | 4 | 0 | 0 |
| 355.206681 | 355.206739 | 1554216 | 26 | 28 | 0 | 1 | 0 | 0 |
| 355.22786 | 355.227868 | 17340586 | 23 | 32 | 0 | 3 | 0 | 0 |
| 355.249128 | 355.248998 | 1593451 | 20 | 36 | 0 | 5 | 0 | 0 |
| 355.264284 | 355.264254 | 3088045 | 24 | 36 | 0 | 2 | 0 | 0 |
| 356.113908 | 356.113961 | 1802995 | 19 | 19 | 1 | 6 | 0 | 0 |
| 356.150301 | 356.150346 | 2062326 | 20 | 23 | 1 | 5 | 0 | 0 |
| 356.18674 | 356.186732 | 1806201 | 21 | 27 | 1 | 4 | 0 | 0 |
| 357.061533 | 357.061591 | 4322369 | 18 | 14 | 0 | 8 | 0 | 0 |
| 357.076772 | 357.076847 | 2373699 | 22 | 14 | 0 | 5 | 0 | 0 |
| 357.097976 | 357.097976 | 9507140 | 19 | 18 | 0 | 7 | 0 | 0 |
| 357.101313 | 357.101347 | 2243653 | 16 | 22 | 0 | 7 | 1 | 0 |
| 357.113202 | 357.113233 | 4444998 | 23 | 18 | 0 | 4 | 0 | 0 |
| 357.134314 | 357.134362 | 21128520 | 20 | 22 | 0 | 6 | 0 | 0 |
| 357.13773 | 357.137733 | 2340168 | 17 | 26 | 0 | 6 | 1 | 0 |
| 357.149609 | 357.149618 | 5253705 | 24 | 22 | 0 | 3 | 0 | 0 |
| 357.170757 | 357.170747 | 33031498 | 21 | 26 | 0 | 5 | 0 | 0 |
| 357.185951 | 357.186004 | 4698956 | 25 | 26 | 0 | 2 | 0 | 0 |
| 357.207096 | 357.207133 | 23549262 | 22 | 30 | 0 | 4 | 0 | 0 |
| 357.222422 | 357.222389 | 2248783 | 26 | 30 | 0 | 1 | 0 | 0 |
| 357.243537 | 357.243519 | 8814416 | 23 | 34 | 0 | 3 | 0 | 0 |
| 357.264555 | 357.264648 | 2389842 | 20 | 38 | 0 | 5 | 0 | 0 |
| 358.129739 | 358.129611 | 1564570 | 19 | 21 | 1 | 6 | 0 | 0 |
| 358.166028 | 358.165996 | 2265757 | 20 | 25 | 1 | 5 | 0 | 0 |
| 359.040743 | 359.040856 | 1620837 | 17 | 12 | 0 | 9 | 0 | 0 |
| 359.056023 | 359.056112 | 2848998 | 21 | 12 | 0 | 6 | 0 | 0 |
| 359.077277 | 359.077241 | 4577256 | 18 | 16 | 0 | 8 | 0 | 0 |
| 359.080686 | 359.080612 | 2063592 | 15 | 20 | 0 | 8 | 1 | 0 |
| 359.092467 | 359.092497 | 5049833 | 22 | 16 | 0 | 5 | 0 | 0 |
| 359.1136 | 359.113627 | 11361771 | 19 | 20 | 0 | 7 | 0 | 0 |
| 359.116963 | 359.116997 | 2249707 | 16 | 24 | 0 | 7 | 1 | 0 |
| 359.128862 | 359.128883 | 7176941 | 23 | 20 | 0 | 4 | 0 | 0 |
| 359.149985 | 359.150012 | 25507310 | 20 | 24 | 0 | 6 | 0 | 0 |
| 359.153369 | 359.153383 | 1351406 | 17 | 28 | 0 | 6 | 1 | 0 |
| 359.165232 | 359.165268 | 9207279 | 24 | 24 | 0 | 3 | 0 | 0 |
| 359.186414 | 359.186398 | 26650098 | 21 | 28 | 0 | 5 | 0 | 0 |
| 359.201682 | 359.201654 | 8300531 | 25 | 28 | 0 | 2 | 0 | 0 |
| 359.222726 | 359.222783 | 12586484 | 22 | 32 | 0 | 4 | 0 | 0 |
| 359.238053 | 359.238039 | 2716405 | 26 | 32 | 0 | 1 | 0 | 0 |
| 359.259182 | 359.259169 | 2573815 | 23 | 36 | 0 | 3 | 0 | 0 |
| 360.064066 | 360.064247 | 1418042 | 17 | 16 | 1 | 6 | 0 | 1 |
| 360.087698 | 360.087746 | 1459004 | 21 | 15 | 1 | 5 | 0 | 0 |
| 360.100332 | 360.100633 | 1514045 | 18 | 20 | 1 | 5 | 0 | 1 |
| 360.124148 | 360.124132 | 1448511 | 22 | 19 | 1 | 4 | 0 | 0 |
| 360.145276 | 360.145261 | 2043201 | 19 | 23 | 1 | 6 | 0 | 0 |
| 360.239207 | 360.239161 | 2087880 | 18 | 35 | 1 | 6 | 0 | 0 |
| 361.035367 | 361.035376 | 2949770 | 20 | 10 | 0 | 7 | 0 | 0 |
| 361.056496 | 361.056506 | 2364300 | 17 | 14 | 0 | 9 | 0 | 0 |
| 361.071718 | 361.071762 | 4931981 | 21 | 14 | 0 | 6 | 0 | 0 |
| 361.092856 | 361.092891 | 5420175 | 18 | 18 | 0 | 8 | 0 | 0 |
| 361.096255 | 361.096262 | 2587023 | 15 | 22 | 0 | 8 | 1 | 0 |
| 361.108144 | 361.108147 | 7574673 | 22 | 18 | 0 | 5 | 0 | 0 |
| 361.119425 | 361.119381 | 1802769 | 21 | 18 | 2 | 4 | 0 | 0 |
| 361.129281 | 361.129277 | 14028434 | 19 | 22 | 0 | 7 | 0 | 0 |
| 361.132901 | 361.132647 | 1907730 | 16 | 26 | 0 | 7 | 1 | 0 |
| 361.144487 | 361.144533 | 12296851 | 23 | 22 | 0 | 4 | 0 | 0 |
| 361.165625 | 361.165662 | 25220758 | 20 | 26 | 0 | 6 | 0 | 0 |
| 361.180934 | 361.180918 | 14323350 | 24 | 26 | 0 | 3 | 0 | 0 |
| 361.202016 | 361.202048 | 17495704 | 21 | 30 | 0 | 5 | 0 | 0 |
| 361.217259 | 361.217304 | 10117785 | 25 | 30 | 0 | 2 | 0 | 0 |
| 361.238455 | 361.238433 | 4866203 | 22 | 34 | 0 | 4 | 0 | 0 |
| 361.253702 | 361.253689 | 2768924 | 26 | 34 | 0 | 1 | 0 | 0 |
| 362.066873 | 362.067011 | 1571808 | 20 | 13 | 1 | 6 | 0 | 0 |
| 362.103566 | 362.103396 | 1580259 | 21 | 17 | 1 | 5 | 0 | 0 |
| 362.116001 | 362.116283 | 1924836 | 18 | 22 | 1 | 5 | 0 | 1 |
| 362.139945 | 362.139782 | 1539302 | 22 | 21 | 1 | 4 | 0 | 0 |
| 362.152522 | 362.152668 | 1665511 | 19 | 26 | 1 | 4 | 0 | 1 |
| 363.014717 | 363.014641 | 1514671 | 19 | 8 | 0 | 8 | 0 | 0 |
| 363.050996 | 363.051026 | 4722226 | 20 | 12 | 0 | 7 | 0 | 0 |
| 363.072191 | 363.072156 | 1572595 | 17 | 16 | 0 | 9 | 0 | 0 |
| 363.087421 | 363.087412 | 7334197 | 21 | 16 | 0 | 6 | 0 | 0 |
| 363.108507 | 363.108541 | 5154615 | 18 | 20 | 0 | 8 | 0 | 0 |
| 363.111833 | 363.111912 | 1767991 | 15 | 24 | 0 | 8 | 1 | 0 |
| 363.123746 | 363.123797 | 11220792 | 22 | 20 | 0 | 5 | 0 | 0 |
| 363.13499 | 363.135031 | 1446457 | 21 | 20 | 2 | 4 | 0 | 0 |
| 363.144908 | 363.144927 | 15779641 | 19 | 24 | 0 | 7 | 0 | 0 |
| 363.160179 | 363.160183 | 19254074 | 23 | 24 | 0 | 4 | 0 | 0 |
| 363.181261 | 363.181312 | 20594492 | 20 | 28 | 0 | 6 | 0 | 0 |
| 363.196544 | 363.196568 | 20475710 | 24 | 28 | 0 | 3 | 0 | 0 |
| 363.217717 | 363.217698 | 7871296 | 21 | 32 | 0 | 5 | 0 | 0 |
| 363.232947 | 363.232954 | 10494785 | 25 | 32 | 0 | 2 | 0 | 0 |
| 363.254019 | 363.254083 | 1424450 | 22 | 36 | 0 | 4 | 0 | 0 |
| 363.269239 | 363.269339 | 2221892 | 26 | 36 | 0 | 1 | 0 | 0 |
| 364.082668 | 364.082661 | 2125191 | 20 | 15 | 1 | 6 | 0 | 0 |
| 364.119124 | 364.119046 | 2046858 | 21 | 19 | 1 | 5 | 0 | 0 |
| 364.131806 | 364.131933 | 1838604 | 18 | 24 | 1 | 5 | 0 | 1 |
| 364.155321 | 364.155432 | 2420110 | 22 | 23 | 1 | 4 | 0 | 0 |
| 364.168017 | 364.168318 | 1582351 | 19 | 28 | 1 | 4 | 0 | 1 |
| 364.191857 | 364.191817 | 1481105 | 23 | 27 | 1 | 3 | 0 | 0 |
| 365.030169 | 365.030291 | 2805206 | 19 | 10 | 0 | 8 | 0 | 0 |
| 365.066743 | 365.066676 | 6173658 | 20 | 14 | 0 | 7 | 0 | 0 |
| 365.087743 | 365.087806 | 2411483 | 17 | 18 | 0 | 9 | 0 | 0 |
| 365.103046 | 365.103062 | 9524188 | 21 | 18 | 0 | 6 | 0 | 0 |
| 365.114262 | 365.114295 | 1437533 | 20 | 18 | 2 | 5 | 0 | 0 |
| 365.124216 | 365.124191 | 6813662 | 18 | 22 | 0 | 8 | 0 | 0 |
| 365.139478 | 365.139447 | 18121696 | 22 | 22 | 0 | 5 | 0 | 0 |
| 365.160565 | 365.160577 | 15769569 | 19 | 26 | 0 | 7 | 0 | 0 |
| 365.175798 | 365.175833 | 25718754 | 23 | 26 | 0 | 4 | 0 | 0 |
| 365.196937 | 365.196962 | 13240292 | 20 | 30 | 0 | 6 | 0 | 0 |
| 365.212247 | 365.212218 | 21958630 | 24 | 30 | 0 | 3 | 0 | 0 |
| 365.233327 | 365.233348 | 2640743 | 21 | 34 | 0 | 5 | 0 | 0 |
| 365.248521 | 365.248604 | 8128489 | 25 | 34 | 0 | 2 | 0 | 0 |
| 365.284743 | 365.284989 | 1322604 | 26 | 38 | 0 | 1 | 0 | 0 |
| 366.098243 | 366.098311 | 1661743 | 20 | 17 | 1 | 6 | 0 | 0 |
| 366.11084 | 366.111198 | 1487153 | 17 | 22 | 1 | 6 | 0 | 1 |
| 366.134738 | 366.134696 | 2084147 | 21 | 21 | 1 | 5 | 0 | 0 |
| 366.147543 | 366.147583 | 1482164 | 18 | 26 | 1 | 5 | 0 | 1 |
| 366.171104 | 366.171082 | 1886774 | 22 | 25 | 1 | 4 | 0 | 0 |
| 367.009628 | 367.009555 | 1387516 | 18 | 8 | 0 | 9 | 0 | 0 |
| 367.045927 | 367.045941 | 3952511 | 19 | 12 | 0 | 8 | 0 | 0 |
| 367.082398 | 367.082326 | 7201922 | 20 | 16 | 0 | 7 | 0 | 0 |
| 367.085642 | 367.085697 | 1617410 | 17 | 20 | 0 | 7 | 1 | 0 |
| 367.103349 | 367.103456 | 1620868 | 17 | 20 | 0 | 9 | 0 | 0 |
| 367.118642 | 367.118712 | 12012677 | 21 | 20 | 0 | 6 | 0 | 0 |
| 367.121908 | 367.122083 | 1434757 | 18 | 24 | 0 | 6 | 1 | 0 |
| 367.129815 | 367.129945 | 1610758 | 20 | 20 | 2 | 5 | 0 | 0 |
| 367.139829 | 367.139841 | 7003271 | 18 | 24 | 0 | 8 | 0 | 0 |
| 367.155127 | 367.155097 | 23861384 | 22 | 24 | 0 | 5 | 0 | 0 |
| 367.158284 | 367.158468 | 1318152 | 19 | 28 | 0 | 5 | 1 | 0 |
| 367.170464 | 367.170354 | 1666313 | 26 | 24 | 0 | 2 | 0 | 0 |
| 367.176178 | 367.176227 | 9933962 | 19 | 28 | 0 | 7 | 0 | 0 |
| 367.191434 | 367.191483 | 29755530 | 23 | 28 | 0 | 4 | 0 | 0 |
| 367.194634 | 367.194854 | 1343627 | 20 | 32 | 0 | 4 | 1 | 0 |
| 367.227887 | 367.227868 | 18550926 | 24 | 32 | 0 | 3 | 0 | 0 |
| 367.231282 | 367.231239 | 2681614 | 21 | 36 | 0 | 3 | 1 | 0 |
| 367.264226 | 367.264254 | 4580497 | 25 | 36 | 0 | 2 | 0 | 0 |
| 368.127997 | 368.128185 | 1421273 | 18 | 20 | 5 | 2 | 0 | 1 |
| 368.131873 | 368.131936 | 1468890 | 13 | 27 | 3 | 5 | 2 | 0 |
| 368.138703 | 368.138461 | 1573594 | 14 | 27 | 1 | 8 | 1 | 0 |
| 368.150366 | 368.150346 | 2080091 | 21 | 23 | 1 | 5 | 0 | 0 |
| 368.162879 | 368.163233 | 1366236 | 18 | 28 | 1 | 5 | 0 | 1 |
| 368.1868 | 368.186732 | 1759838 | 22 | 27 | 1 | 4 | 0 | 0 |
| 369.025113 | 369.025205 | 2029349 | 18 | 10 | 0 | 9 | 0 | 0 |
| 369.061576 | 369.061591 | 4438568 | 19 | 14 | 0 | 8 | 0 | 0 |
| 369.076856 | 369.076847 | 1772585 | 23 | 14 | 0 | 5 | 0 | 0 |
| 369.097953 | 369.097976 | 8369451 | 20 | 18 | 0 | 7 | 0 | 0 |
| 369.101228 | 369.101347 | 2088491 | 17 | 22 | 0 | 7 | 1 | 0 |
| 369.113254 | 369.113233 | 2349996 | 24 | 18 | 0 | 4 | 0 | 0 |
| 369.118991 | 369.119106 | 1423149 | 17 | 22 | 0 | 9 | 0 | 0 |
| 369.137754 | 369.137733 | 2657070 | 18 | 26 | 0 | 6 | 1 | 0 |
| 369.149706 | 369.149618 | 2844207 | 25 | 22 | 0 | 3 | 0 | 0 |
| 369.155467 | 369.155491 | 4059824 | 18 | 26 | 0 | 8 | 0 | 0 |
| 369.170705 | 369.170747 | 29301040 | 22 | 26 | 0 | 5 | 0 | 0 |
| 369.17407 | 369.174118 | 1451889 | 19 | 30 | 0 | 5 | 1 | 0 |
| 369.185931 | 369.186004 | 2896946 | 26 | 26 | 0 | 2 | 0 | 0 |
| 369.191797 | 369.191877 | 3703091 | 19 | 30 | 0 | 7 | 0 | 0 |
| 369.207136 | 369.207133 | 26655028 | 23 | 30 | 0 | 4 | 0 | 0 |
| 369.222741 | 369.222389 | 1308981 | 27 | 30 | 0 | 1 | 0 | 0 |
| 369.2435 | 369.243519 | 11450679 | 24 | 34 | 0 | 3 | 0 | 0 |
| 369.279899 | 369.279904 | 2569018 | 25 | 38 | 0 | 2 | 0 | 0 |
| 370.129622 | 370.129611 | 1624066 | 20 | 21 | 1 | 6 | 0 | 0 |
| 370.166146 | 370.165996 | 1885061 | 21 | 25 | 1 | 5 | 0 | 0 |
| 370.202335 | 370.202382 | 1372168 | 22 | 29 | 1 | 4 | 0 | 0 |
| 371.040918 | 371.040856 | 2122575 | 18 | 12 | 0 | 9 | 0 | 0 |
| 371.056043 | 371.056112 | 2141520 | 22 | 12 | 0 | 6 | 0 | 0 |
| 371.077277 | 371.077241 | 4891090 | 19 | 16 | 0 | 8 | 0 | 0 |
| 371.08068 | 371.080612 | 1378706 | 16 | 20 | 0 | 8 | 1 | 0 |
| 371.092503 | 371.092497 | 3409875 | 23 | 16 | 0 | 5 | 0 | 0 |
| 371.113564 | 371.113627 | 10096085 | 20 | 20 | 0 | 7 | 0 | 0 |
| 371.117009 | 371.116997 | 3395797 | 17 | 24 | 0 | 7 | 1 | 0 |
| 371.128821 | 371.128883 | 3875542 | 24 | 20 | 0 | 4 | 0 | 0 |
| 371.15003 | 371.150012 | 21998040 | 21 | 24 | 0 | 6 | 0 | 0 |
| 371.165288 | 371.165268 | 5655514 | 25 | 24 | 0 | 3 | 0 | 0 |
| 371.171214 | 371.171141 | 1629658 | 18 | 28 | 0 | 8 | 0 | 0 |
| 371.18635 | 371.186398 | 28142044 | 22 | 28 | 0 | 5 | 0 | 0 |
| 371.201689 | 371.201654 | 4370141 | 26 | 28 | 0 | 2 | 0 | 0 |
| 371.222801 | 371.222783 | 18483678 | 23 | 32 | 0 | 4 | 0 | 0 |
| 371.238035 | 371.238039 | 1672415 | 27 | 32 | 0 | 1 | 0 | 0 |
| 371.25916 | 371.259169 | 5400033 | 24 | 36 | 0 | 3 | 0 | 0 |
| 372.108893 | 372.108876 | 1342249 | 19 | 19 | 1 | 7 | 0 | 0 |
| 372.145168 | 372.145261 | 1788972 | 20 | 23 | 1 | 6 | 0 | 0 |
| 372.181568 | 372.181647 | 1831343 | 21 | 27 | 1 | 5 | 0 | 0 |
| 373.035445 | 373.035376 | 1708152 | 21 | 10 | 0 | 7 | 0 | 0 |
| 373.056515 | 373.056506 | 2459258 | 18 | 14 | 0 | 9 | 0 | 0 |
| 373.071729 | 373.071762 | 3930747 | 22 | 14 | 0 | 6 | 0 | 0 |
| 373.092795 | 373.092891 | 5365885 | 19 | 18 | 0 | 8 | 0 | 0 |
| 373.096153 | 373.096262 | 1889149 | 16 | 22 | 0 | 8 | 1 | 0 |
| 373.108185 | 373.108147 | 5135230 | 23 | 18 | 0 | 5 | 0 | 0 |
| 373.129308 | 373.129277 | 13789824 | 20 | 22 | 0 | 7 | 0 | 0 |
| 373.132667 | 373.132647 | 2772864 | 17 | 26 | 0 | 7 | 1 | 0 |
| 373.144521 | 373.144533 | 7320705 | 24 | 22 | 0 | 4 | 0 | 0 |
| 373.165632 | 373.165662 | 22206084 | 21 | 26 | 0 | 6 | 0 | 0 |
| 373.18087 | 373.180918 | 9285252 | 25 | 26 | 0 | 3 | 0 | 0 |
| 373.202089 | 373.202048 | 20781702 | 22 | 30 | 0 | 5 | 0 | 0 |
| 373.21728 | 373.217304 | 6090376 | 26 | 30 | 0 | 2 | 0 | 0 |
| 373.23841 | 373.238433 | 9275017 | 23 | 34 | 0 | 4 | 0 | 0 |
| 373.253923 | 373.253689 | 1568395 | 27 | 34 | 0 | 1 | 0 | 0 |
| 373.259562 | 373.259562 | 4797323 | 20 | 38 | 0 | 6 | 0 | 0 |
| 373.274753 | 373.274819 | 1605644 | 24 | 38 | 0 | 3 | 0 | 0 |
| 374.066812 | 374.067011 | 1556432 | 21 | 13 | 1 | 6 | 0 | 0 |
| 374.103458 | 374.103396 | 1523539 | 22 | 17 | 1 | 5 | 0 | 0 |
| 374.139971 | 374.139782 | 1339734 | 23 | 21 | 1 | 4 | 0 | 0 |
| 374.152445 | 374.152668 | 1371223 | 20 | 26 | 1 | 4 | 0 | 1 |
| 374.160933 | 374.160911 | 1481560 | 20 | 25 | 1 | 6 | 0 | 0 |
| 375.05104 | 375.051026 | 3895332 | 21 | 12 | 0 | 7 | 0 | 0 |
| 375.072068 | 375.072156 | 2123302 | 18 | 16 | 0 | 9 | 0 | 0 |
| 375.087385 | 375.087412 | 5656871 | 22 | 16 | 0 | 6 | 0 | 0 |
| 375.108524 | 375.108541 | 5674793 | 19 | 20 | 0 | 8 | 0 | 0 |
| 375.111999 | 375.111912 | 1485417 | 16 | 24 | 0 | 8 | 1 | 0 |
| 375.12379 | 375.123797 | 8922922 | 23 | 20 | 0 | 5 | 0 | 0 |
| 375.144917 | 375.144927 | 15044396 | 20 | 24 | 0 | 7 | 0 | 0 |
| 375.148262 | 375.148297 | 1644076 | 17 | 28 | 0 | 7 | 1 | 0 |
| 375.16014 | 375.160183 | 12106541 | 24 | 24 | 0 | 4 | 0 | 0 |
| 375.181278 | 375.181312 | 20228912 | 21 | 28 | 0 | 6 | 0 | 0 |
| 375.196595 | 375.196568 | 13825841 | 25 | 28 | 0 | 3 | 0 | 0 |
| 375.217701 | 375.217698 | 12242738 | 22 | 32 | 0 | 5 | 0 | 0 |
| 375.232917 | 375.232954 | 8112948 | 26 | 32 | 0 | 2 | 0 | 0 |
| 375.253974 | 375.254083 | 3143990 | 23 | 36 | 0 | 4 | 0 | 0 |
| 375.269305 | 375.269339 | 1669559 | 27 | 36 | 0 | 1 | 0 | 0 |
| 375.275207 | 375.275213 | 3863607 | 20 | 40 | 0 | 6 | 0 | 0 |
| 376.058862 | 376.059162 | 1628795 | 17 | 16 | 1 | 7 | 0 | 1 |
| 376.118966 | 376.119046 | 1887232 | 22 | 19 | 1 | 5 | 0 | 0 |
| 376.130127 | 376.13028 | 1394945 | 21 | 19 | 3 | 4 | 0 | 0 |
| 377.030319 | 377.030291 | 2511822 | 20 | 10 | 0 | 8 | 0 | 0 |
| 377.066658 | 377.066676 | 5256146 | 21 | 14 | 0 | 7 | 0 | 0 |
| 377.087826 | 377.087806 | 2295763 | 18 | 18 | 0 | 9 | 0 | 0 |
| 377.103072 | 377.103062 | 8125909 | 22 | 18 | 0 | 6 | 0 | 0 |
| 377.114513 | 377.114295 | 1343702 | 21 | 18 | 2 | 5 | 0 | 0 |
| 377.12417 | 377.124191 | 7646167 | 19 | 22 | 0 | 8 | 0 | 0 |
| 377.127626 | 377.127562 | 1423959 | 16 | 26 | 0 | 8 | 1 | 0 |
| 377.139391 | 377.139447 | 12185560 | 23 | 22 | 0 | 5 | 0 | 0 |
| 377.16061 | 377.160577 | 17221594 | 20 | 26 | 0 | 7 | 0 | 0 |
| 377.175838 | 377.175833 | 19102682 | 24 | 26 | 0 | 4 | 0 | 0 |
| 377.196922 | 377.196962 | 14528477 | 21 | 30 | 0 | 6 | 0 | 0 |
| 377.212159 | 377.212218 | 17265630 | 25 | 30 | 0 | 3 | 0 | 0 |
| 377.233333 | 377.233348 | 5373920 | 22 | 34 | 0 | 5 | 0 | 0 |
| 377.248583 | 377.248604 | 7299553 | 26 | 34 | 0 | 2 | 0 | 0 |
| 378.098188 | 378.098311 | 1609643 | 21 | 17 | 1 | 6 | 0 | 0 |
| 378.134565 | 378.134696 | 2154542 | 22 | 21 | 1 | 5 | 0 | 0 |
| 378.147456 | 378.147583 | 1551535 | 19 | 26 | 1 | 5 | 0 | 1 |
| 378.171108 | 378.171082 | 2326065 | 23 | 25 | 1 | 4 | 0 | 0 |
| 379.045996 | 379.045941 | 3888765 | 20 | 12 | 0 | 8 | 0 | 0 |
| 379.082337 | 379.082326 | 7540865 | 21 | 16 | 0 | 7 | 0 | 0 |
| 379.103411 | 379.103456 | 2567042 | 18 | 20 | 0 | 9 | 0 | 0 |
| 379.118637 | 379.118712 | 9733252 | 22 | 20 | 0 | 6 | 0 | 0 |
| 379.122141 | 379.122083 | 1743492 | 19 | 24 | 0 | 6 | 1 | 0 |
| 379.139812 | 379.139841 | 9300101 | 19 | 24 | 0 | 8 | 0 | 0 |
| 379.155097 | 379.155097 | 17899654 | 23 | 24 | 0 | 5 | 0 | 0 |
| 379.17624 | 379.176227 | 14035081 | 20 | 28 | 0 | 7 | 0 | 0 |
| 379.191439 | 379.191483 | 23538826 | 24 | 28 | 0 | 4 | 0 | 0 |
| 379.212595 | 379.212612 | 7082636 | 21 | 32 | 0 | 6 | 0 | 0 |
| 379.227837 | 379.227868 | 16792718 | 25 | 32 | 0 | 3 | 0 | 0 |
| 379.231342 | 379.231239 | 1349197 | 22 | 36 | 0 | 3 | 1 | 0 |
| 379.24906 | 379.248998 | 1683727 | 22 | 36 | 0 | 5 | 0 | 0 |
| 379.264185 | 379.264254 | 6088336 | 26 | 36 | 0 | 2 | 0 | 0 |
| 380.113904 | 380.113961 | 1440922 | 21 | 19 | 1 | 6 | 0 | 0 |
| 380.150299 | 380.150346 | 1671646 | 22 | 23 | 1 | 5 | 0 | 0 |
| 380.162864 | 380.163233 | 1359327 | 19 | 28 | 1 | 5 | 0 | 1 |
| 380.186766 | 380.186732 | 1574497 | 23 | 27 | 1 | 4 | 0 | 0 |
| 381.025149 | 381.025205 | 2340139 | 19 | 10 | 0 | 9 | 0 | 0 |
| 381.061501 | 381.061591 | 4758574 | 20 | 14 | 0 | 8 | 0 | 0 |
| 381.076955 | 381.076847 | 1399727 | 24 | 14 | 0 | 5 | 0 | 0 |
| 381.097963 | 381.097976 | 7735601 | 21 | 18 | 0 | 7 | 0 | 0 |
| 381.101366 | 381.101347 | 1996465 | 18 | 22 | 0 | 7 | 1 | 0 |
| 381.113248 | 381.113233 | 1549362 | 25 | 18 | 0 | 4 | 0 | 0 |
| 381.119115 | 381.119106 | 2848307 | 18 | 22 | 0 | 9 | 0 | 0 |
| 381.134315 | 381.134362 | 13664564 | 22 | 22 | 0 | 6 | 0 | 0 |
| 381.137711 | 381.137733 | 2035252 | 19 | 26 | 0 | 6 | 1 | 0 |
| 381.149723 | 381.149618 | 1659829 | 26 | 22 | 0 | 3 | 0 | 0 |
| 381.15542 | 381.155491 | 7674166 | 19 | 26 | 0 | 8 | 0 | 0 |
| 381.170759 | 381.170747 | 24255800 | 23 | 26 | 0 | 5 | 0 | 0 |
| 381.186017 | 381.186004 | 1354169 | 27 | 26 | 0 | 2 | 0 | 0 |
| 381.191862 | 381.191877 | 8682809 | 20 | 30 | 0 | 7 | 0 | 0 |
| 381.207096 | 381.207133 | 24210746 | 24 | 30 | 0 | 4 | 0 | 0 |
| 381.210251 | 381.210504 | 1365819 | 21 | 34 | 0 | 4 | 1 | 0 |
| 381.228293 | 381.228262 | 2699580 | 21 | 34 | 0 | 6 | 0 | 0 |
| 381.243505 | 381.243519 | 12907838 | 25 | 34 | 0 | 3 | 0 | 0 |
| 381.279914 | 381.279904 | 3757889 | 26 | 38 | 0 | 2 | 0 | 0 |
| 382.093453 | 382.093225 | 1458953 | 20 | 17 | 1 | 7 | 0 | 0 |
| 382.12951 | 382.129611 | 1719436 | 21 | 21 | 1 | 6 | 0 | 0 |
| 382.166096 | 382.165996 | 2115215 | 22 | 25 | 1 | 5 | 0 | 0 |
| 382.202383 | 382.202382 | 1653906 | 23 | 29 | 1 | 4 | 0 | 0 |
| 383.040783 | 383.040856 | 2438749 | 19 | 12 | 0 | 9 | 0 | 0 |
| 383.055969 | 383.056112 | 1423326 | 23 | 12 | 0 | 6 | 0 | 0 |
| 383.077252 | 383.077241 | 5655776 | 20 | 16 | 0 | 8 | 0 | 0 |
| 383.092391 | 383.092497 | 2218849 | 24 | 16 | 0 | 5 | 0 | 0 |
| 383.113588 | 383.113627 | 8482275 | 21 | 20 | 0 | 7 | 0 | 0 |
| 383.116892 | 383.116997 | 2494691 | 18 | 24 | 0 | 7 | 1 | 0 |
| 383.124895 | 383.12486 | 1398820 | 20 | 20 | 2 | 6 | 0 | 0 |
| 383.128714 | 383.128883 | 3075556 | 25 | 20 | 0 | 4 | 0 | 0 |
| 383.134655 | 383.134756 | 2312677 | 18 | 24 | 0 | 9 | 0 | 0 |
| 383.150049 | 383.150012 | 18730470 | 22 | 24 | 0 | 6 | 0 | 0 |
| 383.153278 | 383.153383 | 2686439 | 19 | 28 | 0 | 6 | 1 | 0 |
| 383.165314 | 383.165268 | 2858984 | 26 | 24 | 0 | 3 | 0 | 0 |
| 383.171192 | 383.171141 | 4272616 | 19 | 28 | 0 | 8 | 0 | 0 |
| 383.186361 | 383.186398 | 26279402 | 23 | 28 | 0 | 5 | 0 | 0 |
| 383.201494 | 383.201654 | 2106347 | 27 | 28 | 0 | 2 | 0 | 0 |
| 383.222755 | 383.222783 | 21159404 | 24 | 32 | 0 | 4 | 0 | 0 |
| 383.259117 | 383.259169 | 7504368 | 25 | 36 | 0 | 3 | 0 | 0 |
| 383.295525 | 383.295554 | 1782131 | 26 | 40 | 0 | 2 | 0 | 0 |
| 384.145404 | 384.145261 | 2134079 | 21 | 23 | 1 | 6 | 0 | 0 |
| 384.181578 | 384.181647 | 2325058 | 22 | 27 | 1 | 5 | 0 | 0 |
| 385.056479 | 385.056506 | 2525840 | 19 | 14 | 0 | 9 | 0 | 0 |
| 385.071799 | 385.071762 | 2392722 | 23 | 14 | 0 | 6 | 0 | 0 |
| 385.092884 | 385.092891 | 5753748 | 20 | 18 | 0 | 8 | 0 | 0 |
| 385.096222 | 385.096262 | 2080916 | 17 | 22 | 0 | 8 | 1 | 0 |
| 385.108047 | 385.108147 | 3841685 | 24 | 18 | 0 | 5 | 0 | 0 |
| 385.129289 | 385.129277 | 11469463 | 21 | 22 | 0 | 7 | 0 | 0 |
| 385.132639 | 385.132647 | 3577751 | 18 | 26 | 0 | 7 | 1 | 0 |
| 385.144531 | 385.144533 | 5192856 | 25 | 22 | 0 | 4 | 0 | 0 |
| 385.165659 | 385.165662 | 22150810 | 22 | 26 | 0 | 6 | 0 | 0 |
| 385.180906 | 385.180918 | 5342108 | 26 | 26 | 0 | 3 | 0 | 0 |
| 385.186621 | 385.186791 | 1515548 | 19 | 30 | 0 | 8 | 0 | 0 |
| 385.202035 | 385.202048 | 24848030 | 23 | 30 | 0 | 5 | 0 | 0 |
| 385.217292 | 385.217304 | 3884191 | 27 | 30 | 0 | 2 | 0 | 0 |
| 385.238427 | 385.238433 | 14599841 | 24 | 34 | 0 | 4 | 0 | 0 |
| 385.274802 | 385.274819 | 3486628 | 25 | 38 | 0 | 3 | 0 | 0 |
| 386.13958 | 386.139782 | 1417842 | 24 | 21 | 1 | 4 | 0 | 0 |
| 386.161054 | 386.160911 | 1926132 | 21 | 25 | 1 | 6 | 0 | 0 |
| 386.197172 | 386.197297 | 1642231 | 22 | 29 | 1 | 5 | 0 | 0 |
| 387.051003 | 387.051026 | 3031876 | 22 | 12 | 0 | 7 | 0 | 0 |
| 387.072081 | 387.072156 | 3046982 | 19 | 16 | 0 | 9 | 0 | 0 |
| 387.08736 | 387.087412 | 4161352 | 23 | 16 | 0 | 6 | 0 | 0 |
| 387.108606 | 387.108541 | 6732618 | 20 | 20 | 0 | 8 | 0 | 0 |
| 387.11195 | 387.111912 | 2904394 | 17 | 24 | 0 | 8 | 1 | 0 |
| 387.123748 | 387.123797 | 5527883 | 24 | 20 | 0 | 5 | 0 | 0 |
| 387.144871 | 387.144927 | 14615373 | 21 | 24 | 0 | 7 | 0 | 0 |
| 387.148222 | 387.148297 | 2814797 | 18 | 28 | 0 | 7 | 1 | 0 |
| 387.160114 | 387.160183 | 7801166 | 25 | 24 | 0 | 4 | 0 | 0 |
| 387.181328 | 387.181312 | 21257040 | 22 | 28 | 0 | 6 | 0 | 0 |
| 387.196625 | 387.196568 | 8060242 | 26 | 28 | 0 | 3 | 0 | 0 |
| 387.217668 | 387.217698 | 17243988 | 23 | 32 | 0 | 5 | 0 | 0 |
| 387.232887 | 387.232954 | 5065045 | 27 | 32 | 0 | 2 | 0 | 0 |
| 387.254079 | 387.254083 | 6985559 | 24 | 36 | 0 | 4 | 0 | 0 |
| 388.082495 | 388.082661 | 1390242 | 22 | 15 | 1 | 6 | 0 | 0 |
| 388.119179 | 388.119046 | 1535654 | 23 | 19 | 1 | 5 | 0 | 0 |
| 388.155423 | 388.155432 | 1861929 | 24 | 23 | 1 | 4 | 0 | 0 |
| 388.176597 | 388.176561 | 1431979 | 21 | 27 | 1 | 6 | 0 | 0 |
| 389.030163 | 389.030291 | 2147321 | 21 | 10 | 0 | 8 | 0 | 0 |
| 389.066675 | 389.066676 | 3971324 | 22 | 14 | 0 | 7 | 0 | 0 |
| 389.087738 | 389.087806 | 2529534 | 19 | 18 | 0 | 9 | 0 | 0 |
| 389.103121 | 389.103062 | 5889792 | 23 | 18 | 0 | 6 | 0 | 0 |
| 389.124113 | 389.124191 | 7269378 | 20 | 22 | 0 | 8 | 0 | 0 |
| 389.127466 | 389.127562 | 2357506 | 17 | 26 | 0 | 8 | 1 | 0 |
| 389.139452 | 389.139447 | 8306179 | 24 | 22 | 0 | 5 | 0 | 0 |
| 389.160607 | 389.160577 | 16640005 | 21 | 26 | 0 | 7 | 0 | 0 |
| 389.164001 | 389.163948 | 1843973 | 18 | 30 | 0 | 7 | 1 | 0 |
| 389.175858 | 389.175833 | 12157958 | 25 | 26 | 0 | 4 | 0 | 0 |
| 389.196934 | 389.196962 | 17778696 | 22 | 30 | 0 | 6 | 0 | 0 |
| 389.212183 | 389.212218 | 11021834 | 26 | 30 | 0 | 3 | 0 | 0 |
| 389.233361 | 389.233348 | 9404428 | 23 | 34 | 0 | 5 | 0 | 0 |
| 389.248661 | 389.248604 | 5841421 | 27 | 34 | 0 | 2 | 0 | 0 |
| 389.254389 | 389.254477 | 1397390 | 20 | 38 | 0 | 7 | 0 | 0 |
| 389.269723 | 389.269733 | 2570511 | 24 | 38 | 0 | 4 | 0 | 0 |
| 390.098353 | 390.098311 | 1841243 | 22 | 17 | 1 | 6 | 0 | 0 |
| 390.134691 | 390.134696 | 1429471 | 23 | 21 | 1 | 5 | 0 | 0 |
| 390.182186 | 390.182315 | 1423459 | 23 | 25 | 3 | 3 | 0 | 0 |
| 391.045935 | 391.045941 | 3451571 | 21 | 12 | 0 | 8 | 0 | 0 |
| 391.082294 | 391.082326 | 5450935 | 22 | 16 | 0 | 7 | 0 | 0 |
| 391.103363 | 391.103456 | 2344761 | 19 | 20 | 0 | 9 | 0 | 0 |
| 391.118735 | 391.118712 | 7287994 | 23 | 20 | 0 | 6 | 0 | 0 |
| 391.139879 | 391.139841 | 8816828 | 20 | 24 | 0 | 8 | 0 | 0 |
| 391.142987 | 391.143212 | 1543100 | 17 | 28 | 0 | 8 | 1 | 0 |
| 391.155118 | 391.155097 | 12288189 | 24 | 24 | 0 | 5 | 0 | 0 |
| 391.176242 | 391.176227 | 15626431 | 21 | 28 | 0 | 7 | 0 | 0 |
| 391.19151 | 391.191483 | 16299201 | 25 | 28 | 0 | 4 | 0 | 0 |
| 391.212604 | 391.212612 | 11519171 | 22 | 32 | 0 | 6 | 0 | 0 |
| 391.227817 | 391.227868 | 13833412 | 26 | 32 | 0 | 3 | 0 | 0 |
| 391.249038 | 391.248998 | 3228230 | 23 | 36 | 0 | 5 | 0 | 0 |
| 391.264312 | 391.264254 | 4999880 | 27 | 36 | 0 | 2 | 0 | 0 |
| 392.11401 | 392.113961 | 1399191 | 22 | 19 | 1 | 6 | 0 | 0 |
| 392.1504 | 392.150346 | 1907226 | 23 | 23 | 1 | 5 | 0 | 0 |
| 392.186705 | 392.186732 | 1471902 | 24 | 27 | 1 | 4 | 0 | 0 |
| 393.02511 | 393.025205 | 2108524 | 20 | 10 | 0 | 9 | 0 | 0 |
| 393.06162 | 393.061591 | 4773232 | 21 | 14 | 0 | 8 | 0 | 0 |
| 393.09798 | 393.097976 | 6225267 | 22 | 18 | 0 | 7 | 0 | 0 |
| 393.101279 | 393.101347 | 1994611 | 19 | 22 | 0 | 7 | 1 | 0 |
| 393.11935 | 393.119106 | 3333493 | 19 | 22 | 0 | 9 | 0 | 0 |
| 393.134445 | 393.134362 | 10889591 | 23 | 22 | 0 | 6 | 0 | 0 |
| 393.137834 | 393.137733 | 1836535 | 20 | 26 | 0 | 6 | 1 | 0 |
| 393.145509 | 393.145595 | 1505400 | 22 | 22 | 2 | 5 | 0 | 0 |
| 393.155536 | 393.155491 | 11167097 | 20 | 26 | 0 | 8 | 0 | 0 |
| 393.170704 | 393.170747 | 17504634 | 24 | 26 | 0 | 5 | 0 | 0 |
| 393.191851 | 393.191877 | 10936700 | 21 | 30 | 0 | 7 | 0 | 0 |
| 393.207158 | 393.207133 | 19816830 | 25 | 30 | 0 | 4 | 0 | 0 |
| 393.228307 | 393.228262 | 4847744 | 22 | 34 | 0 | 6 | 0 | 0 |
| 393.243543 | 393.243519 | 12431233 | 26 | 34 | 0 | 3 | 0 | 0 |
| 393.246758 | 393.246889 | 2249345 | 23 | 38 | 0 | 3 | 1 | 0 |
| 393.279766 | 393.279904 | 4391300 | 27 | 38 | 0 | 2 | 0 | 0 |
| 394.093117 | 394.093225 | 1614289 | 21 | 17 | 1 | 7 | 0 | 0 |
| 394.12959 | 394.129611 | 1836245 | 22 | 21 | 1 | 6 | 0 | 0 |
| 394.165833 | 394.165996 | 1328344 | 23 | 25 | 1 | 5 | 0 | 0 |
| 394.2025 | 394.202382 | 1695708 | 24 | 29 | 1 | 4 | 0 | 0 |
| 395.040851 | 395.040856 | 2611755 | 20 | 12 | 0 | 9 | 0 | 0 |
| 395.077259 | 395.077241 | 4860975 | 21 | 16 | 0 | 8 | 0 | 0 |
| 395.092388 | 395.092497 | 1607472 | 25 | 16 | 0 | 5 | 0 | 0 |
| 395.11366 | 395.113627 | 10459698 | 22 | 20 | 0 | 7 | 0 | 0 |
| 395.1171 | 395.116997 | 2658355 | 19 | 24 | 0 | 7 | 1 | 0 |
| 395.129114 | 395.128883 | 1665588 | 26 | 20 | 0 | 4 | 0 | 0 |
| 395.134712 | 395.134756 | 4116788 | 19 | 24 | 0 | 9 | 0 | 0 |
| 395.149935 | 395.150012 | 14367286 | 23 | 24 | 0 | 6 | 0 | 0 |
| 395.153516 | 395.153383 | 1842742 | 20 | 28 | 0 | 6 | 1 | 0 |
| 395.165314 | 395.165268 | 2102327 | 27 | 24 | 0 | 3 | 0 | 0 |
| 395.171068 | 395.171141 | 8747576 | 20 | 28 | 0 | 8 | 0 | 0 |
| 395.186419 | 395.186398 | 22680122 | 24 | 28 | 0 | 5 | 0 | 0 |
| 395.201622 | 395.201654 | 1438715 | 28 | 28 | 0 | 2 | 0 | 0 |
| 395.207512 | 395.207527 | 5018171 | 21 | 32 | 0 | 7 | 0 | 0 |
| 395.222785 | 395.222783 | 18787900 | 25 | 32 | 0 | 4 | 0 | 0 |
| 395.226352 | 395.226154 | 2476861 | 22 | 36 | 0 | 4 | 1 | 0 |
| 395.243692 | 395.243912 | 1620543 | 22 | 36 | 0 | 6 | 0 | 0 |
| 395.259045 | 395.259169 | 9289280 | 26 | 36 | 0 | 3 | 0 | 0 |
| 395.295503 | 395.295554 | 2716996 | 27 | 40 | 0 | 2 | 0 | 0 |
| 396.109029 | 396.108876 | 1469842 | 21 | 19 | 1 | 7 | 0 | 0 |
| 396.145153 | 396.145261 | 1531541 | 22 | 23 | 1 | 6 | 0 | 0 |
| 396.181528 | 396.181647 | 1485337 | 23 | 27 | 1 | 5 | 0 | 0 |
| 397.056555 | 397.056506 | 2994669 | 20 | 14 | 0 | 9 | 0 | 0 |
| 397.071681 | 397.071762 | 2015982 | 24 | 14 | 0 | 6 | 0 | 0 |
| 397.092822 | 397.092891 | 5076209 | 21 | 18 | 0 | 8 | 0 | 0 |
| 397.096133 | 397.096262 | 2125041 | 18 | 22 | 0 | 8 | 1 | 0 |
| 397.108144 | 397.108147 | 2197874 | 25 | 18 | 0 | 5 | 0 | 0 |
| 397.12932 | 397.129277 | 10882292 | 22 | 22 | 0 | 7 | 0 | 0 |
| 397.132741 | 397.132647 | 3800564 | 19 | 26 | 0 | 7 | 1 | 0 |
| 397.144554 | 397.144533 | 2504950 | 26 | 22 | 0 | 4 | 0 | 0 |
| 397.150325 | 397.150406 | 2648054 | 19 | 26 | 0 | 9 | 0 | 0 |
| 397.165642 | 397.165662 | 18561784 | 23 | 26 | 0 | 6 | 0 | 0 |
| 397.169231 | 397.169033 | 2285048 | 20 | 30 | 0 | 6 | 1 | 0 |
| 397.180918 | 397.180918 | 2500409 | 27 | 26 | 0 | 3 | 0 | 0 |
| 397.186844 | 397.186791 | 4273658 | 20 | 30 | 0 | 8 | 0 | 0 |
| 397.202116 | 397.202048 | 24716028 | 24 | 30 | 0 | 5 | 0 | 0 |
| 397.217479 | 397.217304 | 1781501 | 28 | 30 | 0 | 2 | 0 | 0 |
| 397.223281 | 397.223177 | 2246653 | 21 | 34 | 0 | 7 | 0 | 0 |
| 397.238432 | 397.238433 | 15329023 | 25 | 34 | 0 | 4 | 0 | 0 |
| 397.274725 | 397.274819 | 5192962 | 26 | 38 | 0 | 3 | 0 | 0 |
| 398.124699 | 398.124526 | 1490389 | 21 | 21 | 1 | 7 | 0 | 0 |
| 398.161016 | 398.160911 | 1664728 | 22 | 25 | 1 | 6 | 0 | 0 |
| 398.19733 | 398.197297 | 1616476 | 23 | 29 | 1 | 5 | 0 | 0 |
| 399.050872 | 399.051026 | 1629359 | 23 | 12 | 0 | 7 | 0 | 0 |
| 399.072219 | 399.072156 | 2906418 | 20 | 16 | 0 | 9 | 0 | 0 |
| 399.087514 | 399.087412 | 3372723 | 24 | 16 | 0 | 6 | 0 | 0 |
| 399.108497 | 399.108541 | 5904821 | 21 | 20 | 0 | 8 | 0 | 0 |
| 399.111796 | 399.111912 | 2823349 | 18 | 24 | 0 | 8 | 1 | 0 |
| 399.123843 | 399.123797 | 3607479 | 25 | 20 | 0 | 5 | 0 | 0 |
| 399.144916 | 399.144927 | 12474809 | 22 | 24 | 0 | 7 | 0 | 0 |
| 399.148297 | 399.148297 | 4804537 | 19 | 28 | 0 | 7 | 1 | 0 |
| 399.160242 | 399.160183 | 4807098 | 26 | 24 | 0 | 4 | 0 | 0 |
| 399.166346 | 399.166056 | 1521595 | 19 | 28 | 0 | 9 | 0 | 0 |
| 399.181323 | 399.181312 | 21521340 | 23 | 28 | 0 | 6 | 0 | 0 |
| 399.184901 | 399.184683 | 2483645 | 20 | 32 | 0 | 6 | 1 | 0 |
| 399.196412 | 399.196568 | 4557246 | 27 | 28 | 0 | 3 | 0 | 0 |
| 399.217628 | 399.217698 | 21066688 | 24 | 32 | 0 | 5 | 0 | 0 |
| 399.232869 | 399.232954 | 2627521 | 28 | 32 | 0 | 2 | 0 | 0 |
| 399.254153 | 399.254083 | 9696707 | 25 | 36 | 0 | 4 | 0 | 0 |
| 399.290379 | 399.290469 | 1599303 | 26 | 40 | 0 | 3 | 0 | 0 |
| 400.176688 | 400.176561 | 1543198 | 22 | 27 | 1 | 6 | 0 | 0 |
| 401.051447 | 401.05142 | 1438581 | 19 | 14 | 0 | 10 | 0 | 0 |
| 401.066654 | 401.066676 | 3461239 | 23 | 14 | 0 | 7 | 0 | 0 |
| 401.087728 | 401.087806 | 3783289 | 20 | 18 | 0 | 9 | 0 | 0 |
| 401.091015 | 401.091177 | 1531193 | 17 | 22 | 0 | 9 | 1 | 0 |
| 401.102975 | 401.103062 | 4866171 | 24 | 18 | 0 | 6 | 0 | 0 |
| 401.124201 | 401.124191 | 7161469 | 21 | 22 | 0 | 8 | 0 | 0 |
| 401.127507 | 401.127562 | 3941245 | 18 | 26 | 0 | 8 | 1 | 0 |
| 401.13957 | 401.139447 | 6025854 | 25 | 22 | 0 | 5 | 0 | 0 |
| 401.160608 | 401.160577 | 15316096 | 22 | 26 | 0 | 7 | 0 | 0 |
| 401.163859 | 401.163948 | 3542657 | 19 | 30 | 0 | 7 | 1 | 0 |
| 401.175755 | 401.175833 | 7466626 | 26 | 26 | 0 | 4 | 0 | 0 |
| 401.196902 | 401.196962 | 19623044 | 23 | 30 | 0 | 6 | 0 | 0 |
| 401.212222 | 401.212218 | 6905989 | 27 | 30 | 0 | 3 | 0 | 0 |
| 401.233335 | 401.233348 | 14845063 | 24 | 34 | 0 | 5 | 0 | 0 |
| 401.248607 | 401.248604 | 3107593 | 28 | 34 | 0 | 2 | 0 | 0 |
| 401.269713 | 401.269733 | 4586763 | 25 | 38 | 0 | 4 | 0 | 0 |
| 402.145833 | 402.14593 | 1583971 | 23 | 21 | 3 | 4 | 0 | 0 |
| 402.171075 | 402.171082 | 1484517 | 25 | 25 | 1 | 4 | 0 | 0 |
| 402.192074 | 402.192211 | 1452519 | 22 | 29 | 1 | 6 | 0 | 0 |
| 403.045938 | 403.045941 | 3383358 | 22 | 12 | 0 | 8 | 0 | 0 |
| 403.066986 | 403.06707 | 1662016 | 19 | 16 | 0 | 10 | 0 | 0 |
| 403.082487 | 403.082326 | 4385089 | 23 | 16 | 0 | 7 | 0 | 0 |
| 403.10343 | 403.103456 | 3375427 | 20 | 20 | 0 | 9 | 0 | 0 |
| 403.106778 | 403.106827 | 2150724 | 17 | 24 | 0 | 9 | 1 | 0 |
| 403.118581 | 403.118712 | 6048069 | 24 | 20 | 0 | 6 | 0 | 0 |
| 403.139848 | 403.139841 | 9006407 | 21 | 24 | 0 | 8 | 0 | 0 |
| 403.143159 | 403.143212 | 3137095 | 18 | 28 | 0 | 8 | 1 | 0 |
| 403.155153 | 403.155097 | 8669769 | 25 | 24 | 0 | 5 | 0 | 0 |
| 403.176193 | 403.176227 | 16887114 | 22 | 28 | 0 | 7 | 0 | 0 |
| 403.179802 | 403.179598 | 1412139 | 19 | 32 | 0 | 7 | 1 | 0 |
| 403.191451 | 403.191483 | 11954508 | 26 | 28 | 0 | 4 | 0 | 0 |
| 403.212604 | 403.212612 | 15617358 | 23 | 32 | 0 | 6 | 0 | 0 |
| 403.227867 | 403.227868 | 9099088 | 27 | 32 | 0 | 3 | 0 | 0 |
| 403.248972 | 403.248998 | 7658322 | 24 | 36 | 0 | 5 | 0 | 0 |
| 403.264347 | 403.264254 | 4201812 | 28 | 36 | 0 | 2 | 0 | 0 |
| 403.28531 | 403.285383 | 1717846 | 25 | 40 | 0 | 4 | 0 | 0 |
| 404.113915 | 404.113961 | 1680810 | 23 | 19 | 1 | 6 | 0 | 0 |
| 404.150318 | 404.150346 | 1546670 | 24 | 23 | 1 | 5 | 0 | 0 |
| 405.024982 | 405.025205 | 1554951 | 21 | 10 | 0 | 9 | 0 | 0 |
| 405.061465 | 405.061591 | 4451851 | 22 | 14 | 0 | 8 | 0 | 0 |
| 405.098089 | 405.097976 | 5847055 | 23 | 18 | 0 | 7 | 0 | 0 |
| 405.119019 | 405.119106 | 3864593 | 20 | 22 | 0 | 9 | 0 | 0 |
| 405.134334 | 405.134362 | 8307731 | 24 | 22 | 0 | 6 | 0 | 0 |
| 405.155478 | 405.155491 | 11303445 | 21 | 26 | 0 | 8 | 0 | 0 |
| 405.159008 | 405.158862 | 1529749 | 18 | 30 | 0 | 8 | 1 | 0 |
| 405.170752 | 405.170747 | 13614614 | 25 | 26 | 0 | 5 | 0 | 0 |
| 405.19187 | 405.191877 | 15734296 | 22 | 30 | 0 | 7 | 0 | 0 |
| 405.207163 | 405.207133 | 15872538 | 26 | 30 | 0 | 4 | 0 | 0 |
| 405.22828 | 405.228262 | 9690140 | 23 | 34 | 0 | 6 | 0 | 0 |
| 405.24346 | 405.243519 | 10053150 | 27 | 34 | 0 | 3 | 0 | 0 |
| 405.280003 | 405.279904 | 3637026 | 28 | 38 | 0 | 2 | 0 | 0 |
| 406.129766 | 406.129611 | 1948025 | 23 | 21 | 1 | 6 | 0 | 0 |
| 406.166045 | 406.165996 | 1435709 | 24 | 25 | 1 | 5 | 0 | 0 |
| 407.040929 | 407.040856 | 2326488 | 21 | 12 | 0 | 9 | 0 | 0 |
| 407.07731 | 407.077241 | 4058332 | 22 | 16 | 0 | 8 | 0 | 0 |
| 407.098566 | 407.09837 | 1540318 | 19 | 20 | 0 | 10 | 0 | 0 |
| 407.113605 | 407.113627 | 6719712 | 23 | 20 | 0 | 7 | 0 | 0 |
| 407.117182 | 407.116997 | 1672928 | 20 | 24 | 0 | 7 | 1 | 0 |
| 407.134742 | 407.134756 | 5048034 | 20 | 24 | 0 | 9 | 0 | 0 |
| 407.150038 | 407.150012 | 11045604 | 24 | 24 | 0 | 6 | 0 | 0 |
| 407.153112 | 407.153383 | 1409380 | 21 | 28 | 0 | 6 | 1 | 0 |
| 407.171134 | 407.171141 | 10931430 | 21 | 28 | 0 | 8 | 0 | 0 |
| 407.186452 | 407.186398 | 17133288 | 25 | 28 | 0 | 5 | 0 | 0 |
| 407.207468 | 407.207527 | 9889002 | 22 | 32 | 0 | 7 | 0 | 0 |
| 407.222724 | 407.222783 | 17535724 | 26 | 32 | 0 | 4 | 0 | 0 |
| 407.243892 | 407.243912 | 3493870 | 23 | 36 | 0 | 6 | 0 | 0 |
| 407.259217 | 407.259169 | 9196783 | 27 | 36 | 0 | 3 | 0 | 0 |
| 407.26233 | 407.262539 | 1973231 | 24 | 40 | 0 | 3 | 1 | 0 |
| 407.295554 | 407.295554 | 3415795 | 28 | 40 | 0 | 2 | 0 | 0 |
| 408.145246 | 408.145261 | 1537356 | 23 | 23 | 1 | 6 | 0 | 0 |
| 408.181829 | 408.181647 | 1458256 | 24 | 27 | 1 | 5 | 0 | 0 |
| 409.056461 | 409.056506 | 3466157 | 21 | 14 | 0 | 9 | 0 | 0 |
| 409.092938 | 409.092891 | 5132849 | 22 | 18 | 0 | 8 | 0 | 0 |
| 409.096367 | 409.096262 | 1864545 | 19 | 22 | 0 | 8 | 1 | 0 |
| 409.108296 | 409.108147 | 1810354 | 26 | 18 | 0 | 5 | 0 | 0 |
| 409.114029 | 409.11402 | 1730099 | 19 | 22 | 0 | 10 | 0 | 0 |
| 409.129307 | 409.129277 | 7862197 | 23 | 22 | 0 | 7 | 0 | 0 |
| 409.132737 | 409.132647 | 3095989 | 20 | 26 | 0 | 7 | 1 | 0 |
| 409.144657 | 409.144533 | 1849270 | 27 | 22 | 0 | 4 | 0 | 0 |
| 409.150385 | 409.150406 | 5131191 | 20 | 26 | 0 | 9 | 0 | 0 |
| 409.165656 | 409.165662 | 14756792 | 24 | 26 | 0 | 6 | 0 | 0 |
| 409.169144 | 409.169033 | 1439161 | 21 | 30 | 0 | 6 | 1 | 0 |
| 409.180813 | 409.180918 | 1901242 | 28 | 26 | 0 | 3 | 0 | 0 |
| 409.202097 | 409.202048 | 19689404 | 25 | 30 | 0 | 5 | 0 | 0 |
| 409.223164 | 409.223177 | 3580350 | 22 | 34 | 0 | 7 | 0 | 0 |
| 409.238377 | 409.238433 | 16090048 | 26 | 34 | 0 | 4 | 0 | 0 |
| 409.274771 | 409.274819 | 6974916 | 27 | 38 | 0 | 3 | 0 | 0 |
| 409.311085 | 409.311204 | 2040776 | 28 | 42 | 0 | 2 | 0 | 0 |
| 410.160801 | 410.160911 | 1867811 | 23 | 25 | 1 | 6 | 0 | 0 |
| 411.035814 | 411.03577 | 1517697 | 20 | 12 | 0 | 10 | 0 | 0 |
| 411.050977 | 411.051026 | 1489026 | 24 | 12 | 0 | 7 | 0 | 0 |
| 411.072132 | 411.072156 | 3385733 | 21 | 16 | 0 | 9 | 0 | 0 |
| 411.087322 | 411.087412 | 2177926 | 25 | 16 | 0 | 6 | 0 | 0 |
| 411.108554 | 411.108541 | 5486729 | 22 | 20 | 0 | 8 | 0 | 0 |
| 411.111902 | 411.111912 | 3198601 | 19 | 24 | 0 | 8 | 1 | 0 |
| 411.12371 | 411.123797 | 3031690 | 26 | 20 | 0 | 5 | 0 | 0 |
| 411.129801 | 411.129671 | 2007179 | 19 | 24 | 0 | 10 | 0 | 0 |
| 411.144986 | 411.144927 | 10891916 | 23 | 24 | 0 | 7 | 0 | 0 |
| 411.148335 | 411.148297 | 3972237 | 20 | 28 | 0 | 7 | 1 | 0 |
| 411.160121 | 411.160183 | 2710158 | 27 | 24 | 0 | 4 | 0 | 0 |
| 411.165943 | 411.166056 | 3365263 | 20 | 28 | 0 | 9 | 0 | 0 |
| 411.181321 | 411.181312 | 18951312 | 24 | 28 | 0 | 6 | 0 | 0 |
| 411.196675 | 411.196568 | 2693394 | 28 | 28 | 0 | 3 | 0 | 0 |
| 411.202442 | 411.202442 | 3356307 | 21 | 32 | 0 | 8 | 0 | 0 |
| 411.217665 | 411.217698 | 20121748 | 25 | 32 | 0 | 5 | 0 | 0 |
| 411.233176 | 411.232954 | 1887382 | 29 | 32 | 0 | 2 | 0 | 0 |
| 411.254123 | 411.254083 | 12186776 | 26 | 36 | 0 | 4 | 0 | 0 |
| 411.290614 | 411.290469 | 4026524 | 27 | 40 | 0 | 3 | 0 | 0 |
| 412.176416 | 412.176561 | 1538045 | 23 | 27 | 1 | 6 | 0 | 0 |
| 412.213099 | 412.212947 | 1376001 | 24 | 31 | 1 | 5 | 0 | 0 |
| 413.066783 | 413.066676 | 2731102 | 24 | 14 | 0 | 7 | 0 | 0 |
| 413.087821 | 413.087806 | 3282720 | 21 | 18 | 0 | 9 | 0 | 0 |
| 413.091325 | 413.091177 | 1383009 | 18 | 22 | 0 | 9 | 1 | 0 |
| 413.103039 | 413.103062 | 3297634 | 25 | 18 | 0 | 6 | 0 | 0 |
| 413.124155 | 413.124191 | 6402661 | 22 | 22 | 0 | 8 | 0 | 0 |
| 413.12756 | 413.127562 | 4302181 | 19 | 26 | 0 | 8 | 1 | 0 |
| 413.139541 | 413.139447 | 3736678 | 26 | 22 | 0 | 5 | 0 | 0 |
| 413.160568 | 413.160577 | 13742440 | 23 | 26 | 0 | 7 | 0 | 0 |
| 413.163961 | 413.163948 | 4863849 | 20 | 30 | 0 | 7 | 1 | 0 |
| 413.175816 | 413.175833 | 5160554 | 27 | 26 | 0 | 4 | 0 | 0 |
| 413.181648 | 413.181706 | 1466859 | 20 | 30 | 0 | 9 | 0 | 0 |
| 413.196954 | 413.196962 | 19899756 | 24 | 30 | 0 | 6 | 0 | 0 |
| 413.21226 | 413.212218 | 4746094 | 28 | 30 | 0 | 3 | 0 | 0 |
| 413.233367 | 413.233348 | 16305520 | 25 | 34 | 0 | 5 | 0 | 0 |
| 413.248846 | 413.248604 | 1580658 | 29 | 34 | 0 | 2 | 0 | 0 |
| 413.269726 | 413.269733 | 7276405 | 26 | 38 | 0 | 4 | 0 | 0 |
| 413.306038 | 413.306119 | 1750392 | 27 | 42 | 0 | 3 | 0 | 0 |
| 415.045923 | 415.045941 | 2443066 | 23 | 12 | 0 | 8 | 0 | 0 |
| 415.067167 | 415.06707 | 2200124 | 20 | 16 | 0 | 10 | 0 | 0 |
| 415.08233 | 415.082326 | 3839038 | 24 | 16 | 0 | 7 | 0 | 0 |
| 415.103401 | 415.103456 | 3429696 | 21 | 20 | 0 | 9 | 0 | 0 |
| 415.106992 | 415.106827 | 2281792 | 18 | 24 | 0 | 9 | 1 | 0 |
| 415.118678 | 415.118712 | 4660290 | 25 | 20 | 0 | 6 | 0 | 0 |
| 415.139912 | 415.139841 | 8823876 | 22 | 24 | 0 | 8 | 0 | 0 |
| 415.143227 | 415.143212 | 4572997 | 19 | 28 | 0 | 8 | 1 | 0 |
| 415.155151 | 415.155097 | 6073926 | 26 | 24 | 0 | 5 | 0 | 0 |
| 415.176239 | 415.176227 | 16548424 | 23 | 28 | 0 | 7 | 0 | 0 |
| 415.179591 | 415.179598 | 3760713 | 20 | 32 | 0 | 7 | 1 | 0 |
| 415.191555 | 415.191483 | 7245386 | 27 | 28 | 0 | 4 | 0 | 0 |
| 415.212591 | 415.212612 | 18269772 | 24 | 32 | 0 | 6 | 0 | 0 |
| 415.227939 | 415.227868 | 5098062 | 28 | 32 | 0 | 3 | 0 | 0 |
| 415.249049 | 415.248998 | 11097680 | 25 | 36 | 0 | 5 | 0 | 0 |
| 415.264427 | 415.264254 | 1845074 | 29 | 36 | 0 | 2 | 0 | 0 |
| 415.285421 | 415.285383 | 3403348 | 26 | 40 | 0 | 4 | 0 | 0 |
| 416.113882 | 416.113961 | 1517362 | 24 | 19 | 1 | 6 | 0 | 0 |
| 416.171302 | 416.171476 | 1518520 | 22 | 27 | 1 | 7 | 0 | 0 |
| 417.0617 | 417.061591 | 3724061 | 23 | 14 | 0 | 8 | 0 | 0 |
| 417.082719 | 417.08272 | 2001695 | 20 | 18 | 0 | 10 | 0 | 0 |
| 417.098083 | 417.097976 | 4496417 | 24 | 18 | 0 | 7 | 0 | 0 |
| 417.119144 | 417.119106 | 4377892 | 21 | 22 | 0 | 9 | 0 | 0 |
| 417.122627 | 417.122477 | 2631204 | 18 | 26 | 0 | 9 | 1 | 0 |
| 417.134341 | 417.134362 | 6415142 | 25 | 22 | 0 | 6 | 0 | 0 |
| 417.155532 | 417.155491 | 10368296 | 22 | 26 | 0 | 8 | 0 | 0 |
| 417.158923 | 417.158862 | 2766632 | 19 | 30 | 0 | 8 | 1 | 0 |
| 417.170759 | 417.170747 | 8451881 | 26 | 26 | 0 | 5 | 0 | 0 |
| 417.191897 | 417.191877 | 15742764 | 23 | 30 | 0 | 7 | 0 | 0 |
| 417.195355 | 417.195248 | 1619244 | 20 | 34 | 0 | 7 | 1 | 0 |
| 417.207167 | 417.207133 | 9426734 | 27 | 30 | 0 | 4 | 0 | 0 |
| 417.228279 | 417.228262 | 14853936 | 24 | 34 | 0 | 6 | 0 | 0 |
| 417.243573 | 417.243519 | 7020338 | 28 | 34 | 0 | 3 | 0 | 0 |
| 417.264604 | 417.264648 | 5875508 | 25 | 38 | 0 | 5 | 0 | 0 |
| 417.280097 | 417.279904 | 2001462 | 29 | 38 | 0 | 2 | 0 | 0 |
| 418.12937 | 418.129611 | 1362839 | 24 | 21 | 1 | 6 | 0 | 0 |
| 419.040816 | 419.040856 | 2347905 | 22 | 12 | 0 | 9 | 0 | 0 |
| 419.077372 | 419.077241 | 4343301 | 23 | 16 | 0 | 8 | 0 | 0 |
| 419.098503 | 419.09837 | 1641223 | 20 | 20 | 0 | 10 | 0 | 0 |
| 419.101427 | 419.101361 | 1359880 | 19 | 21 | 2 | 7 | 0 | 1 |
| 419.113626 | 419.113627 | 5598473 | 24 | 20 | 0 | 7 | 0 | 0 |
| 419.116838 | 419.116997 | 1382537 | 21 | 24 | 0 | 7 | 1 | 0 |
| 419.134672 | 419.134756 | 5139212 | 21 | 24 | 0 | 9 | 0 | 0 |
| 419.138239 | 419.138127 | 2184972 | 18 | 28 | 0 | 9 | 1 | 0 |
| 419.149992 | 419.150012 | 8207373 | 25 | 24 | 0 | 6 | 0 | 0 |
| 419.153213 | 419.153383 | 1597198 | 22 | 28 | 0 | 6 | 1 | 0 |
| 419.171178 | 419.171141 | 13031440 | 22 | 28 | 0 | 8 | 0 | 0 |
| 419.174768 | 419.174512 | 1575056 | 19 | 32 | 0 | 8 | 1 | 0 |
| 419.186417 | 419.186398 | 13272081 | 26 | 28 | 0 | 5 | 0 | 0 |
| 419.207595 | 419.207527 | 13575188 | 23 | 32 | 0 | 7 | 0 | 0 |
| 419.222832 | 419.222783 | 13657110 | 27 | 32 | 0 | 4 | 0 | 0 |
| 419.243842 | 419.243912 | 7547416 | 24 | 36 | 0 | 6 | 0 | 0 |
| 419.259198 | 419.259169 | 7599130 | 28 | 36 | 0 | 3 | 0 | 0 |
| 419.28022 | 419.280298 | 2280988 | 25 | 40 | 0 | 5 | 0 | 0 |
| 419.295643 | 419.295554 | 2090270 | 29 | 40 | 0 | 2 | 0 | 0 |
| 420.10876 | 420.108876 | 1428093 | 23 | 19 | 1 | 7 | 0 | 0 |
| 420.145125 | 420.145261 | 1817985 | 24 | 23 | 1 | 6 | 0 | 0 |
| 420.181708 | 420.181647 | 1615878 | 25 | 27 | 1 | 5 | 0 | 0 |
| 421.056471 | 421.056506 | 3217133 | 22 | 14 | 0 | 9 | 0 | 0 |
| 421.093022 | 421.092891 | 4838129 | 23 | 18 | 0 | 8 | 0 | 0 |
| 421.114092 | 421.11402 | 1662195 | 20 | 22 | 0 | 10 | 0 | 0 |
| 421.129309 | 421.129277 | 6713589 | 24 | 22 | 0 | 7 | 0 | 0 |
| 421.132573 | 421.132647 | 1886709 | 21 | 26 | 0 | 7 | 1 | 0 |
| 421.150477 | 421.150406 | 5769464 | 21 | 26 | 0 | 9 | 0 | 0 |
| 421.165696 | 421.165662 | 11636985 | 25 | 26 | 0 | 6 | 0 | 0 |
| 421.186863 | 421.186791 | 10648316 | 22 | 30 | 0 | 8 | 0 | 0 |
| 421.202069 | 421.202048 | 16136446 | 26 | 30 | 0 | 5 | 0 | 0 |
| 421.223149 | 421.223177 | 7811328 | 23 | 34 | 0 | 7 | 0 | 0 |
| 421.238492 | 421.238433 | 13749506 | 27 | 34 | 0 | 4 | 0 | 0 |
| 421.259486 | 421.259562 | 3190533 | 24 | 38 | 0 | 6 | 0 | 0 |
| 421.274845 | 421.274819 | 6536966 | 28 | 38 | 0 | 3 | 0 | 0 |
| 421.311474 | 421.311204 | 1561099 | 29 | 42 | 0 | 2 | 0 | 0 |
| 422.124501 | 422.124526 | 1526891 | 23 | 21 | 1 | 7 | 0 | 0 |
| 423.035943 | 423.03577 | 1529112 | 21 | 12 | 0 | 10 | 0 | 0 |
| 423.072231 | 423.072156 | 3752157 | 22 | 16 | 0 | 9 | 0 | 0 |
| 423.108454 | 423.108541 | 5602785 | 23 | 20 | 0 | 8 | 0 | 0 |
| 423.112009 | 423.111912 | 2268898 | 20 | 24 | 0 | 8 | 1 | 0 |
| 423.123828 | 423.123797 | 1702627 | 27 | 20 | 0 | 5 | 0 | 0 |
| 423.129789 | 423.129671 | 2374884 | 20 | 24 | 0 | 10 | 0 | 0 |
| 423.144969 | 423.144927 | 9189861 | 24 | 24 | 0 | 7 | 0 | 0 |
| 423.148531 | 423.148297 | 2484966 | 21 | 28 | 0 | 7 | 1 | 0 |
| 423.160192 | 423.160183 | 1630439 | 28 | 24 | 0 | 4 | 0 | 0 |
| 423.166153 | 423.166056 | 5878248 | 21 | 28 | 0 | 9 | 0 | 0 |
| 423.181298 | 423.181312 | 14225386 | 25 | 28 | 0 | 6 | 0 | 0 |
| 423.196495 | 423.196568 | 1409260 | 29 | 28 | 0 | 3 | 0 | 0 |
| 423.202533 | 423.202442 | 5482989 | 22 | 32 | 0 | 8 | 0 | 0 |
| 423.217697 | 423.217698 | 18193902 | 26 | 32 | 0 | 5 | 0 | 0 |
| 423.23886 | 423.238827 | 3071601 | 23 | 36 | 0 | 7 | 0 | 0 |
| 423.254112 | 423.254083 | 13754867 | 27 | 36 | 0 | 4 | 0 | 0 |
| 423.257533 | 423.257454 | 3321843 | 24 | 40 | 0 | 4 | 1 | 0 |
| 423.290433 | 423.290469 | 5862391 | 28 | 40 | 0 | 3 | 0 | 0 |
| 424.176782 | 424.176561 | 1567586 | 24 | 27 | 1 | 6 | 0 | 0 |
| 424.212974 | 424.212947 | 1498087 | 25 | 31 | 1 | 5 | 0 | 0 |
| 425.051636 | 425.05142 | 1459149 | 21 | 14 | 0 | 10 | 0 | 0 |
| 425.066735 | 425.066676 | 2133199 | 25 | 14 | 0 | 7 | 0 | 0 |
| 425.087875 | 425.087806 | 3883729 | 22 | 18 | 0 | 9 | 0 | 0 |
| 425.091295 | 425.091177 | 1680721 | 19 | 22 | 0 | 9 | 1 | 0 |
| 425.103218 | 425.103062 | 2515923 | 26 | 18 | 0 | 6 | 0 | 0 |
| 425.124205 | 425.124191 | 5937366 | 23 | 22 | 0 | 8 | 0 | 0 |
| 425.127445 | 425.127562 | 3438550 | 20 | 26 | 0 | 8 | 1 | 0 |
| 425.139527 | 425.139447 | 2314711 | 27 | 22 | 0 | 5 | 0 | 0 |
| 425.145472 | 425.145321 | 1862872 | 20 | 26 | 0 | 10 | 0 | 0 |
| 425.160589 | 425.160577 | 11196122 | 24 | 26 | 0 | 7 | 0 | 0 |
| 425.163923 | 425.163948 | 3503834 | 21 | 30 | 0 | 7 | 1 | 0 |
| 425.175941 | 425.175833 | 3089628 | 28 | 26 | 0 | 4 | 0 | 0 |
| 425.181636 | 425.181706 | 3713757 | 21 | 30 | 0 | 9 | 0 | 0 |
| 425.196992 | 425.196962 | 18257630 | 25 | 30 | 0 | 6 | 0 | 0 |
| 425.212246 | 425.212218 | 2636512 | 29 | 30 | 0 | 3 | 0 | 0 |
| 425.218222 | 425.218092 | 2144097 | 22 | 34 | 0 | 8 | 0 | 0 |
| 425.233342 | 425.233348 | 17364706 | 26 | 34 | 0 | 5 | 0 | 0 |
| 425.24868 | 425.248604 | 1876965 | 30 | 34 | 0 | 2 | 0 | 0 |
| 425.269664 | 425.269733 | 8873191 | 27 | 38 | 0 | 4 | 0 | 0 |
| 425.306254 | 425.306119 | 3342572 | 28 | 42 | 0 | 3 | 0 | 0 |
| 426.19225 | 426.192211 | 1750616 | 24 | 29 | 1 | 6 | 0 | 0 |
| 427.04607 | 427.045941 | 1368770 | 24 | 12 | 0 | 8 | 0 | 0 |
| 427.0671 | 427.06707 | 2277444 | 21 | 16 | 0 | 10 | 0 | 0 |
| 427.08252 | 427.082326 | 2777286 | 25 | 16 | 0 | 7 | 0 | 0 |
| 427.10356 | 427.103456 | 3906377 | 22 | 20 | 0 | 9 | 0 | 0 |
| 427.106943 | 427.106827 | 2739657 | 19 | 24 | 0 | 9 | 1 | 0 |
| 427.118875 | 427.118712 | 3255499 | 26 | 20 | 0 | 6 | 0 | 0 |
| 427.139869 | 427.139841 | 7738829 | 23 | 24 | 0 | 8 | 0 | 0 |
| 427.143369 | 427.143212 | 5436366 | 20 | 28 | 0 | 8 | 1 | 0 |
| 427.155225 | 427.155097 | 4095439 | 27 | 24 | 0 | 5 | 0 | 0 |
| 427.176263 | 427.176227 | 15388626 | 24 | 28 | 0 | 7 | 0 | 0 |
| 427.179571 | 427.179598 | 2725330 | 21 | 32 | 0 | 7 | 1 | 0 |
| 427.191444 | 427.191483 | 4581844 | 28 | 28 | 0 | 4 | 0 | 0 |
| 427.212632 | 427.212612 | 19211222 | 25 | 32 | 0 | 6 | 0 | 0 |
| 427.227909 | 427.227868 | 3456216 | 29 | 32 | 0 | 3 | 0 | 0 |
| 427.249044 | 427.248998 | 13075419 | 26 | 36 | 0 | 5 | 0 | 0 |
| 427.285521 | 427.285383 | 5338592 | 27 | 40 | 0 | 4 | 0 | 0 |
| 427.321551 | 427.321769 | 1572452 | 28 | 44 | 0 | 3 | 0 | 0 |
| 429.061435 | 429.061591 | 2430012 | 24 | 14 | 0 | 8 | 0 | 0 |
| 429.082717 | 429.08272 | 2319551 | 21 | 18 | 0 | 10 | 0 | 0 |
| 429.09779 | 429.097976 | 3925441 | 25 | 18 | 0 | 7 | 0 | 0 |
| 429.11912 | 429.119106 | 5210820 | 22 | 22 | 0 | 9 | 0 | 0 |
| 429.122573 | 429.122477 | 3975108 | 19 | 26 | 0 | 9 | 1 | 0 |
| 429.134421 | 429.134362 | 4477382 | 26 | 22 | 0 | 6 | 0 | 0 |
| 429.155532 | 429.155491 | 9557704 | 23 | 26 | 0 | 8 | 0 | 0 |
| 429.158812 | 429.158482 | 5363913 | 22 | 27 | 2 | 5 | 0 | 1 |
| 429.170818 | 429.170747 | 5528778 | 27 | 26 | 0 | 5 | 0 | 0 |
| 429.191862 | 429.191877 | 15859917 | 24 | 30 | 0 | 7 | 0 | 0 |
| 429.207171 | 429.207133 | 6075599 | 28 | 30 | 0 | 4 | 0 | 0 |
| 429.228283 | 429.228262 | 16414929 | 25 | 34 | 0 | 6 | 0 | 0 |
| 429.243491 | 429.243519 | 3839699 | 29 | 34 | 0 | 3 | 0 | 0 |
| 429.264661 | 429.264648 | 8302806 | 26 | 38 | 0 | 5 | 0 | 0 |
| 429.301114 | 429.301033 | 2500442 | 27 | 42 | 0 | 4 | 0 | 0 |
| 431.04107 | 431.040856 | 2273205 | 23 | 12 | 0 | 9 | 0 | 0 |
| 431.077267 | 431.077241 | 4222906 | 24 | 16 | 0 | 8 | 0 | 0 |
| 431.09838 | 431.09837 | 3051196 | 21 | 20 | 0 | 10 | 0 | 0 |
| 431.113711 | 431.113627 | 5029310 | 25 | 20 | 0 | 7 | 0 | 0 |
| 431.134811 | 431.134756 | 5564353 | 22 | 24 | 0 | 9 | 0 | 0 |
| 431.138096 | 431.138127 | 3824321 | 19 | 28 | 0 | 9 | 1 | 0 |
| 431.150085 | 431.150012 | 6546371 | 26 | 24 | 0 | 6 | 0 | 0 |
| 431.171126 | 431.171141 | 11473349 | 23 | 28 | 0 | 8 | 0 | 0 |
| 431.174644 | 431.174512 | 2924998 | 20 | 32 | 0 | 8 | 1 | 0 |
| 431.186414 | 431.186398 | 8116167 | 27 | 28 | 0 | 5 | 0 | 0 |
| 431.207543 | 431.207527 | 16262602 | 24 | 32 | 0 | 7 | 0 | 0 |
| 431.222821 | 431.222783 | 9002444 | 28 | 32 | 0 | 4 | 0 | 0 |
| 431.243932 | 431.243912 | 12447182 | 25 | 36 | 0 | 6 | 0 | 0 |
| 431.259053 | 431.259169 | 3709392 | 29 | 36 | 0 | 3 | 0 | 0 |
| 431.280313 | 431.280298 | 4026323 | 26 | 40 | 0 | 5 | 0 | 0 |
| 432.156219 | 432.156494 | 1750082 | 24 | 23 | 3 | 5 | 0 | 0 |
| 433.056316 | 433.056506 | 3153588 | 23 | 14 | 0 | 9 | 0 | 0 |
| 433.092776 | 433.092891 | 4118713 | 24 | 18 | 0 | 8 | 0 | 0 |
| 433.113783 | 433.11402 | 2282171 | 21 | 22 | 0 | 10 | 0 | 0 |
| 433.117585 | 433.117391 | 1474492 | 18 | 26 | 0 | 10 | 1 | 0 |
| 433.129366 | 433.129277 | 6292157 | 25 | 22 | 0 | 7 | 0 | 0 |
| 433.132875 | 433.132647 | 1518782 | 22 | 26 | 0 | 7 | 1 | 0 |
| 433.150463 | 433.150406 | 7123136 | 22 | 26 | 0 | 9 | 0 | 0 |
| 433.153968 | 433.153777 | 2205120 | 19 | 30 | 0 | 9 | 1 | 0 |
| 433.165712 | 433.165662 | 8393922 | 26 | 26 | 0 | 6 | 0 | 0 |
| 433.186822 | 433.186791 | 12805828 | 23 | 30 | 0 | 8 | 0 | 0 |
| 433.202053 | 433.202048 | 11402950 | 27 | 30 | 0 | 5 | 0 | 0 |
| 433.22314 | 433.223177 | 12326601 | 24 | 34 | 0 | 7 | 0 | 0 |
| 433.238427 | 433.238433 | 9769675 | 28 | 34 | 0 | 4 | 0 | 0 |
| 433.259598 | 433.259562 | 6888654 | 25 | 38 | 0 | 6 | 0 | 0 |
| 433.274815 | 433.274819 | 4259024 | 29 | 38 | 0 | 3 | 0 | 0 |
| 433.295884 | 433.295948 | 1595090 | 26 | 42 | 0 | 5 | 0 | 0 |
| 434.124297 | 434.124526 | 1502012 | 24 | 21 | 1 | 7 | 0 | 0 |
| 434.172287 | 434.172144 | 1584962 | 24 | 25 | 3 | 5 | 0 | 0 |
| 435.035706 | 435.03577 | 1618608 | 22 | 12 | 0 | 10 | 0 | 0 |
| 435.072278 | 435.072156 | 3806644 | 23 | 16 | 0 | 9 | 0 | 0 |
| 435.108401 | 435.108541 | 5039545 | 24 | 20 | 0 | 8 | 0 | 0 |
| 435.111933 | 435.111912 | 1915833 | 21 | 24 | 0 | 8 | 1 | 0 |
| 435.129818 | 435.129671 | 2732220 | 21 | 24 | 0 | 10 | 0 | 0 |
| 435.144884 | 435.144927 | 7427518 | 25 | 24 | 0 | 7 | 0 | 0 |
| 435.148288 | 435.148297 | 1851454 | 22 | 28 | 0 | 7 | 1 | 0 |
| 435.160375 | 435.160183 | 1429440 | 29 | 24 | 0 | 4 | 0 | 0 |
| 435.166148 | 435.166056 | 7652289 | 22 | 28 | 0 | 9 | 0 | 0 |
| 435.181358 | 435.181312 | 12197826 | 26 | 28 | 0 | 6 | 0 | 0 |
| 435.202541 | 435.202442 | 9798597 | 23 | 32 | 0 | 8 | 0 | 0 |
| 435.217747 | 435.217698 | 13913031 | 27 | 32 | 0 | 5 | 0 | 0 |
| 435.238912 | 435.238827 | 6715338 | 24 | 36 | 0 | 7 | 0 | 0 |
| 435.254116 | 435.254083 | 10276812 | 28 | 36 | 0 | 4 | 0 | 0 |
| 435.275095 | 435.275213 | 3097550 | 25 | 40 | 0 | 6 | 0 | 0 |
| 435.290465 | 435.290469 | 3841488 | 29 | 40 | 0 | 3 | 0 | 0 |
| 435.294147 | 435.29384 | 1571281 | 26 | 44 | 0 | 3 | 1 | 0 |
| 436.176791 | 436.176561 | 1576513 | 25 | 27 | 1 | 6 | 0 | 0 |
| 437.051289 | 437.05142 | 2126385 | 22 | 14 | 0 | 10 | 0 | 0 |
| 437.087855 | 437.087806 | 3578550 | 23 | 18 | 0 | 9 | 0 | 0 |
| 437.091201 | 437.091177 | 2117878 | 20 | 22 | 0 | 9 | 1 | 0 |
| 437.103324 | 437.103062 | 1437624 | 27 | 18 | 0 | 6 | 0 | 0 |
| 437.124224 | 437.124191 | 5938363 | 24 | 22 | 0 | 8 | 0 | 0 |
| 437.127422 | 437.127562 | 2661563 | 21 | 26 | 0 | 8 | 1 | 0 |
| 437.139778 | 437.139447 | 2112957 | 28 | 22 | 0 | 5 | 0 | 0 |
| 437.145393 | 437.145321 | 3218109 | 21 | 26 | 0 | 10 | 0 | 0 |
| 437.160554 | 437.160577 | 9858239 | 25 | 26 | 0 | 7 | 0 | 0 |
| 437.164126 | 437.163948 | 3433664 | 22 | 30 | 0 | 7 | 1 | 0 |
| 437.175942 | 437.175833 | 1574209 | 29 | 26 | 0 | 4 | 0 | 0 |
| 437.181744 | 437.181706 | 5422274 | 22 | 30 | 0 | 9 | 0 | 0 |
| 437.196963 | 437.196962 | 15459524 | 26 | 30 | 0 | 6 | 0 | 0 |
| 437.212312 | 437.212218 | 1764678 | 30 | 30 | 0 | 3 | 0 | 0 |
| 437.218019 | 437.218092 | 5680583 | 23 | 34 | 0 | 8 | 0 | 0 |
| 437.233443 | 437.233348 | 16446664 | 27 | 34 | 0 | 5 | 0 | 0 |
| 437.254423 | 437.254477 | 2730571 | 24 | 38 | 0 | 7 | 0 | 0 |
| 437.269721 | 437.269733 | 10413261 | 28 | 38 | 0 | 4 | 0 | 0 |
| 437.273393 | 437.273104 | 1552846 | 25 | 42 | 0 | 4 | 1 | 0 |
| 437.306247 | 437.306119 | 3053778 | 29 | 42 | 0 | 3 | 0 | 0 |
| 438.156059 | 438.155826 | 1831230 | 24 | 25 | 1 | 7 | 0 | 0 |
| 438.192082 | 438.192211 | 1521987 | 25 | 29 | 1 | 6 | 0 | 0 |
| 439.066938 | 439.06707 | 2562227 | 22 | 16 | 0 | 10 | 0 | 0 |
| 439.082347 | 439.082326 | 2043829 | 26 | 16 | 0 | 7 | 0 | 0 |
| 439.103403 | 439.103456 | 3712824 | 23 | 20 | 0 | 9 | 0 | 0 |
| 439.107051 | 439.106827 | 2846136 | 20 | 24 | 0 | 9 | 1 | 0 |
| 439.118513 | 439.118712 | 2437562 | 27 | 20 | 0 | 6 | 0 | 0 |
| 439.139887 | 439.139841 | 6820284 | 24 | 24 | 0 | 8 | 0 | 0 |
| 439.143188 | 439.143212 | 4548285 | 21 | 28 | 0 | 8 | 1 | 0 |
| 439.15519 | 439.155097 | 3158974 | 28 | 24 | 0 | 5 | 0 | 0 |
| 439.160837 | 439.160971 | 2023871 | 21 | 28 | 0 | 10 | 0 | 0 |
| 439.176273 | 439.176227 | 13157825 | 25 | 28 | 0 | 7 | 0 | 0 |
| 439.179697 | 439.179598 | 3353537 | 22 | 32 | 0 | 7 | 1 | 0 |
| 439.191586 | 439.191483 | 2971075 | 29 | 28 | 0 | 4 | 0 | 0 |
| 439.197416 | 439.197356 | 3257796 | 22 | 32 | 0 | 9 | 0 | 0 |
| 439.212673 | 439.212612 | 17540550 | 26 | 32 | 0 | 6 | 0 | 0 |
| 439.227843 | 439.227868 | 1932520 | 30 | 32 | 0 | 3 | 0 | 0 |
| 439.233954 | 439.233742 | 1897864 | 23 | 36 | 0 | 8 | 0 | 0 |
| 439.248903 | 439.248998 | 14801354 | 27 | 36 | 0 | 5 | 0 | 0 |
| 439.285413 | 439.285383 | 8827855 | 28 | 40 | 0 | 4 | 0 | 0 |
| 439.321911 | 439.321769 | 3092436 | 29 | 44 | 0 | 3 | 0 | 0 |
| 440.207946 | 440.207861 | 1465925 | 25 | 31 | 1 | 6 | 0 | 0 |
| 441.061424 | 441.061591 | 1962418 | 25 | 14 | 0 | 8 | 0 | 0 |
| 441.082876 | 441.08272 | 2422709 | 22 | 18 | 0 | 10 | 0 | 0 |
| 441.086195 | 441.086091 | 1428405 | 19 | 22 | 0 | 10 | 1 | 0 |
| 441.098177 | 441.097976 | 2997431 | 26 | 18 | 0 | 7 | 0 | 0 |
| 441.119153 | 441.119106 | 4323514 | 23 | 22 | 0 | 9 | 0 | 0 |
| 441.122657 | 441.122477 | 3893946 | 20 | 26 | 0 | 9 | 1 | 0 |
| 441.134385 | 441.134362 | 3490235 | 27 | 22 | 0 | 6 | 0 | 0 |
| 441.155532 | 441.155491 | 9738430 | 24 | 26 | 0 | 8 | 0 | 0 |
| 441.158996 | 441.158862 | 5115583 | 21 | 30 | 0 | 8 | 1 | 0 |
| 441.170761 | 441.170747 | 3827136 | 28 | 26 | 0 | 5 | 0 | 0 |
| 441.176322 | 441.176621 | 1477825 | 21 | 30 | 0 | 10 | 0 | 0 |
| 441.191896 | 441.191877 | 15285955 | 25 | 30 | 0 | 7 | 0 | 0 |
| 441.195461 | 441.195248 | 2331843 | 22 | 34 | 0 | 7 | 1 | 0 |
| 441.207258 | 441.207133 | 3673797 | 29 | 30 | 0 | 4 | 0 | 0 |
| 441.228222 | 441.228262 | 16672455 | 26 | 34 | 0 | 6 | 0 | 0 |
| 441.243728 | 441.243519 | 1968841 | 30 | 34 | 0 | 3 | 0 | 0 |
| 441.264701 | 441.264648 | 11354828 | 27 | 38 | 0 | 5 | 0 | 0 |
| 441.301157 | 441.301033 | 5561553 | 28 | 42 | 0 | 4 | 0 | 0 |
| 442.187239 | 442.187126 | 1462082 | 24 | 29 | 1 | 7 | 0 | 0 |
| 443.041004 | 443.040856 | 1591215 | 24 | 12 | 0 | 9 | 0 | 0 |
| 443.077264 | 443.077241 | 3236787 | 25 | 16 | 0 | 8 | 0 | 0 |
| 443.098274 | 443.09837 | 2806710 | 22 | 20 | 0 | 10 | 0 | 0 |
| 443.101565 | 443.101361 | 1941942 | 21 | 21 | 2 | 7 | 0 | 1 |
| 443.113537 | 443.113627 | 3943352 | 26 | 20 | 0 | 7 | 0 | 0 |
| 443.13485 | 443.134756 | 5354427 | 23 | 24 | 0 | 9 | 0 | 0 |
| 443.138193 | 443.138127 | 5236411 | 20 | 28 | 0 | 9 | 1 | 0 |
| 443.15009 | 443.150012 | 5113277 | 27 | 24 | 0 | 6 | 0 | 0 |
| 443.171173 | 443.171141 | 11240383 | 24 | 28 | 0 | 8 | 0 | 0 |
| 443.174564 | 443.174512 | 3822784 | 21 | 32 | 0 | 8 | 1 | 0 |
| 443.186393 | 443.186398 | 5961153 | 28 | 28 | 0 | 5 | 0 | 0 |
| 443.207508 | 443.207527 | 16368580 | 25 | 32 | 0 | 7 | 0 | 0 |
| 443.222775 | 443.222783 | 4994758 | 29 | 32 | 0 | 4 | 0 | 0 |
| 443.244004 | 443.243912 | 13065160 | 26 | 36 | 0 | 6 | 0 | 0 |
| 443.259219 | 443.259169 | 2465994 | 30 | 36 | 0 | 3 | 0 | 0 |
| 443.280296 | 443.280298 | 7579085 | 27 | 40 | 0 | 5 | 0 | 0 |
| 443.316917 | 443.316683 | 2292690 | 28 | 44 | 0 | 4 | 0 | 0 |
| 444.181795 | 444.181647 | 1431360 | 27 | 27 | 1 | 5 | 0 | 0 |
| 445.056424 | 445.056506 | 2587311 | 24 | 14 | 0 | 9 | 0 | 0 |
| 445.092975 | 445.092891 | 3848371 | 25 | 18 | 0 | 8 | 0 | 0 |
| 445.113993 | 445.11402 | 2635446 | 22 | 22 | 0 | 10 | 0 | 0 |
| 445.11735 | 445.117011 | 2414006 | 21 | 23 | 2 | 7 | 0 | 1 |
| 445.129308 | 445.129277 | 4861368 | 26 | 22 | 0 | 7 | 0 | 0 |
| 445.150429 | 445.150406 | 6234811 | 23 | 26 | 0 | 9 | 0 | 0 |
| 445.153969 | 445.153777 | 4331707 | 20 | 30 | 0 | 9 | 1 | 0 |
| 445.165565 | 445.165662 | 5812669 | 27 | 26 | 0 | 6 | 0 | 0 |
| 445.18687 | 445.186791 | 13177023 | 24 | 30 | 0 | 8 | 0 | 0 |
| 445.202113 | 445.202048 | 7944385 | 28 | 30 | 0 | 5 | 0 | 0 |
| 445.223224 | 445.223177 | 16474308 | 25 | 34 | 0 | 7 | 0 | 0 |
| 445.238398 | 445.238433 | 6131398 | 29 | 34 | 0 | 4 | 0 | 0 |
| 445.25957 | 445.259562 | 9309384 | 26 | 38 | 0 | 6 | 0 | 0 |
| 445.274769 | 445.274819 | 2173514 | 30 | 38 | 0 | 3 | 0 | 0 |
| 446.172494 | 446.172144 | 1452092 | 25 | 25 | 3 | 5 | 0 | 0 |
| 447.035746 | 447.03577 | 1473576 | 23 | 12 | 0 | 10 | 0 | 0 |
| 447.072244 | 447.072156 | 3455405 | 24 | 16 | 0 | 9 | 0 | 0 |
| 447.093315 | 447.093285 | 2894000 | 21 | 20 | 0 | 11 | 0 | 0 |
| 447.108618 | 447.108541 | 5332402 | 25 | 20 | 0 | 8 | 0 | 0 |
| 447.111755 | 447.111912 | 1438386 | 22 | 24 | 0 | 8 | 1 | 0 |
| 447.129802 | 447.129671 | 3003060 | 22 | 24 | 0 | 10 | 0 | 0 |
| 447.133159 | 447.133041 | 1972277 | 19 | 28 | 0 | 10 | 1 | 0 |
| 447.14502 | 447.144927 | 6418870 | 26 | 24 | 0 | 7 | 0 | 0 |
| 447.148286 | 447.148297 | 1634999 | 23 | 28 | 0 | 7 | 1 | 0 |
| 447.166159 | 447.166056 | 8169913 | 23 | 28 | 0 | 9 | 0 | 0 |
| 447.169554 | 447.169427 | 2716345 | 20 | 32 | 0 | 9 | 1 | 0 |
| 447.181377 | 447.181312 | 8222139 | 27 | 28 | 0 | 6 | 0 | 0 |
| 447.20248 | 447.202442 | 13590973 | 24 | 32 | 0 | 8 | 0 | 0 |
| 447.217714 | 447.217698 | 9636287 | 28 | 32 | 0 | 5 | 0 | 0 |
| 447.238874 | 447.238827 | 12533186 | 25 | 36 | 0 | 7 | 0 | 0 |
| 447.253974 | 447.254083 | 6517188 | 29 | 36 | 0 | 4 | 0 | 0 |
| 447.275229 | 447.275213 | 5086663 | 26 | 40 | 0 | 6 | 0 | 0 |
| 447.290628 | 447.290469 | 2168265 | 30 | 40 | 0 | 3 | 0 | 0 |
| 449.051469 | 449.05142 | 1781157 | 23 | 14 | 0 | 10 | 0 | 0 |
| 449.087635 | 449.087806 | 3517865 | 24 | 18 | 0 | 9 | 0 | 0 |
| 449.109144 | 449.108935 | 1412524 | 21 | 22 | 0 | 11 | 0 | 0 |
| 449.124157 | 449.124191 | 4769966 | 25 | 22 | 0 | 8 | 0 | 0 |
| 449.127621 | 449.127562 | 1879406 | 22 | 26 | 0 | 8 | 1 | 0 |
| 449.139507 | 449.139447 | 1415344 | 29 | 22 | 0 | 5 | 0 | 0 |
| 449.145294 | 449.145321 | 3529649 | 22 | 26 | 0 | 10 | 0 | 0 |
| 449.160532 | 449.160577 | 7873715 | 26 | 26 | 0 | 7 | 0 | 0 |
| 449.163609 | 449.163948 | 2037683 | 23 | 30 | 0 | 7 | 1 | 0 |
| 449.181647 | 449.181706 | 8076981 | 23 | 30 | 0 | 9 | 0 | 0 |
| 449.196988 | 449.196962 | 10745015 | 27 | 30 | 0 | 6 | 0 | 0 |
| 449.218051 | 449.218092 | 9969337 | 24 | 34 | 0 | 8 | 0 | 0 |
| 449.233328 | 449.233348 | 12070587 | 28 | 34 | 0 | 5 | 0 | 0 |
| 449.254578 | 449.254477 | 6569662 | 25 | 38 | 0 | 7 | 0 | 0 |
| 449.269826 | 449.269733 | 6577856 | 29 | 38 | 0 | 4 | 0 | 0 |
| 449.29103 | 449.290863 | 1794243 | 26 | 42 | 0 | 6 | 0 | 0 |
| 449.306061 | 449.306119 | 1589188 | 30 | 42 | 0 | 3 | 0 | 0 |
| 449.309348 | 449.30949 | 1637061 | 27 | 46 | 0 | 3 | 1 | 0 |
| 450.167114 | 450.167059 | 1460015 | 24 | 25 | 3 | 6 | 0 | 0 |
| 450.192328 | 450.192211 | 1397298 | 26 | 29 | 1 | 6 | 0 | 0 |
| 451.06697 | 451.06707 | 2541727 | 23 | 16 | 0 | 10 | 0 | 0 |
| 451.082339 | 451.082326 | 1395360 | 27 | 16 | 0 | 7 | 0 | 0 |
| 451.10344 | 451.103456 | 4192163 | 24 | 20 | 0 | 9 | 0 | 0 |
| 451.10641 | 451.106827 | 1400867 | 21 | 24 | 0 | 9 | 1 | 0 |
| 451.118764 | 451.118712 | 1896229 | 28 | 20 | 0 | 6 | 0 | 0 |
| 451.139884 | 451.139841 | 6152616 | 25 | 24 | 0 | 8 | 0 | 0 |
| 451.143289 | 451.143212 | 3333032 | 22 | 28 | 0 | 8 | 1 | 0 |
| 451.155202 | 451.155097 | 2075817 | 29 | 24 | 0 | 5 | 0 | 0 |
| 451.16103 | 451.160971 | 3582890 | 22 | 28 | 0 | 10 | 0 | 0 |
| 451.176285 | 451.176227 | 9415596 | 26 | 28 | 0 | 7 | 0 | 0 |
| 451.179847 | 451.179598 | 2463916 | 23 | 32 | 0 | 7 | 1 | 0 |
| 451.191553 | 451.191483 | 2251182 | 30 | 28 | 0 | 4 | 0 | 0 |
| 451.197312 | 451.197356 | 5628335 | 23 | 32 | 0 | 9 | 0 | 0 |
| 451.212594 | 451.212612 | 14227376 | 27 | 32 | 0 | 6 | 0 | 0 |
| 451.233811 | 451.233742 | 5091251 | 24 | 36 | 0 | 8 | 0 | 0 |
| 451.249037 | 451.248998 | 13087669 | 28 | 36 | 0 | 5 | 0 | 0 |
| 451.270068 | 451.270127 | 2414520 | 25 | 40 | 0 | 7 | 0 | 0 |
| 451.285336 | 451.285383 | 6566330 | 29 | 40 | 0 | 4 | 0 | 0 |
| 452.13523 | 452.13509 | 1418018 | 24 | 23 | 1 | 8 | 0 | 0 |
| 452.171324 | 452.171476 | 1841190 | 25 | 27 | 1 | 7 | 0 | 0 |
| 453.061486 | 453.061591 | 1764754 | 26 | 14 | 0 | 8 | 0 | 0 |
| 453.082451 | 453.08272 | 2258581 | 23 | 18 | 0 | 10 | 0 | 0 |
| 453.09804 | 453.097976 | 1927703 | 27 | 18 | 0 | 7 | 0 | 0 |
| 453.119034 | 453.119106 | 4230682 | 24 | 22 | 0 | 9 | 0 | 0 |
| 453.122472 | 453.122477 | 3176602 | 21 | 26 | 0 | 9 | 1 | 0 |
| 453.13425 | 453.134362 | 2209307 | 28 | 22 | 0 | 6 | 0 | 0 |
| 453.155542 | 453.155491 | 7992478 | 25 | 26 | 0 | 8 | 0 | 0 |
| 453.158865 | 453.158862 | 5589150 | 22 | 30 | 0 | 8 | 1 | 0 |
| 453.170721 | 453.170747 | 2451616 | 29 | 26 | 0 | 5 | 0 | 0 |
| 453.176895 | 453.176621 | 2532001 | 22 | 30 | 0 | 10 | 0 | 0 |
| 453.191966 | 453.191877 | 13702306 | 26 | 30 | 0 | 7 | 0 | 0 |
| 453.195335 | 453.195248 | 2811299 | 23 | 34 | 0 | 7 | 1 | 0 |
| 453.20731 | 453.207133 | 2092708 | 30 | 30 | 0 | 4 | 0 | 0 |
| 453.212987 | 453.213006 | 2888101 | 23 | 34 | 0 | 9 | 0 | 0 |
| 453.228256 | 453.228262 | 15462055 | 27 | 34 | 0 | 6 | 0 | 0 |
| 453.243404 | 453.243519 | 1497161 | 31 | 34 | 0 | 3 | 0 | 0 |
| 453.249381 | 453.249392 | 2146985 | 24 | 38 | 0 | 8 | 0 | 0 |
| 453.264652 | 453.264648 | 12137643 | 28 | 38 | 0 | 5 | 0 | 0 |
| 453.301061 | 453.301033 | 6214320 | 29 | 42 | 0 | 4 | 0 | 0 |
| 453.3921 | 453.392248 | 1402299 | 24 | 50 | 6 | 2 | 0 | 0 |
| 455.077248 | 455.077241 | 2411973 | 26 | 16 | 0 | 8 | 0 | 0 |
| 455.098183 | 455.09837 | 2563208 | 23 | 20 | 0 | 10 | 0 | 0 |
| 455.101662 | 455.101361 | 1666184 | 22 | 21 | 2 | 7 | 0 | 1 |
| 455.113901 | 455.113627 | 2846602 | 27 | 20 | 0 | 7 | 0 | 0 |
| 455.134786 | 455.134756 | 4953484 | 24 | 24 | 0 | 9 | 0 | 0 |
| 455.138088 | 455.138127 | 5095309 | 21 | 28 | 0 | 9 | 1 | 0 |
| 455.150094 | 455.150012 | 3134094 | 28 | 24 | 0 | 6 | 0 | 0 |
| 455.171154 | 455.171141 | 9748368 | 25 | 28 | 0 | 8 | 0 | 0 |
| 455.174545 | 455.174512 | 5664657 | 22 | 32 | 0 | 8 | 1 | 0 |
| 455.186474 | 455.186398 | 3790738 | 29 | 28 | 0 | 5 | 0 | 0 |
| 455.19243 | 455.192271 | 1582483 | 22 | 32 | 0 | 10 | 0 | 0 |
| 455.207606 | 455.207527 | 15648149 | 26 | 32 | 0 | 7 | 0 | 0 |
| 455.211182 | 455.210898 | 1964181 | 23 | 36 | 0 | 7 | 1 | 0 |
| 455.222923 | 455.222783 | 2949527 | 30 | 32 | 0 | 4 | 0 | 0 |
| 455.243955 | 455.243912 | 16579993 | 27 | 36 | 0 | 6 | 0 | 0 |
| 455.259241 | 455.259169 | 1740955 | 31 | 36 | 0 | 3 | 0 | 0 |
| 455.28031 | 455.280298 | 10055070 | 28 | 40 | 0 | 5 | 0 | 0 |
| 455.316788 | 455.316683 | 6083490 | 29 | 44 | 0 | 4 | 0 | 0 |
| 455.353167 | 455.353069 | 2148518 | 30 | 48 | 0 | 3 | 0 | 0 |
| 457.056565 | 457.056506 | 2213488 | 25 | 14 | 0 | 9 | 0 | 0 |
| 457.077731 | 457.077635 | 1735538 | 22 | 18 | 0 | 11 | 0 | 0 |
| 457.092832 | 457.092891 | 3154548 | 26 | 18 | 0 | 8 | 0 | 0 |
| 457.114328 | 457.11402 | 2866807 | 23 | 22 | 0 | 10 | 0 | 0 |
| 457.117576 | 457.117391 | 2844279 | 20 | 26 | 0 | 10 | 1 | 0 |
| 457.150373 | 457.150406 | 5886843 | 24 | 26 | 0 | 9 | 0 | 0 |
| 457.153864 | 457.153777 | 5299067 | 21 | 30 | 0 | 9 | 1 | 0 |
| 457.165751 | 457.165662 | 4063229 | 28 | 26 | 0 | 6 | 0 | 0 |
| 457.186834 | 457.186791 | 12768895 | 25 | 30 | 0 | 8 | 0 | 0 |
| 457.190368 | 457.190162 | 3885696 | 22 | 34 | 0 | 8 | 1 | 0 |
| 457.202134 | 457.202048 | 5011585 | 29 | 30 | 0 | 5 | 0 | 0 |
| 457.223203 | 457.223177 | 15425155 | 26 | 34 | 0 | 7 | 0 | 0 |
| 457.238494 | 457.238433 | 3868805 | 30 | 34 | 0 | 4 | 0 | 0 |
| 457.259584 | 457.259562 | 12999304 | 27 | 38 | 0 | 6 | 0 | 0 |
| 457.274968 | 457.274819 | 1808137 | 31 | 38 | 0 | 3 | 0 | 0 |
| 457.296049 | 457.295948 | 6928012 | 28 | 42 | 0 | 5 | 0 | 0 |
| 457.332493 | 457.332334 | 2463120 | 29 | 46 | 0 | 4 | 0 | 0 |
| 459.072044 | 459.072156 | 2608602 | 25 | 16 | 0 | 9 | 0 | 0 |
| 459.093495 | 459.093285 | 1946972 | 22 | 20 | 0 | 11 | 0 | 0 |
| 459.10864 | 459.108541 | 3713886 | 26 | 20 | 0 | 8 | 0 | 0 |
| 459.129855 | 459.129671 | 3367265 | 23 | 24 | 0 | 10 | 0 | 0 |
| 459.13308 | 459.133041 | 3754849 | 20 | 28 | 0 | 10 | 1 | 0 |
| 459.144913 | 459.144927 | 5170530 | 27 | 24 | 0 | 7 | 0 | 0 |
| 459.166159 | 459.166056 | 8412005 | 24 | 28 | 0 | 9 | 0 | 0 |
| 459.16946 | 459.169427 | 3809893 | 21 | 32 | 0 | 9 | 1 | 0 |
| 459.181354 | 459.181312 | 6180199 | 28 | 28 | 0 | 6 | 0 | 0 |
| 459.202399 | 459.202442 | 13541225 | 25 | 32 | 0 | 8 | 0 | 0 |
| 459.217706 | 459.217698 | 6244203 | 29 | 32 | 0 | 5 | 0 | 0 |
| 459.238876 | 459.238827 | 13368173 | 26 | 36 | 0 | 7 | 0 | 0 |
| 459.254226 | 459.254083 | 3797871 | 30 | 36 | 0 | 4 | 0 | 0 |
| 459.275252 | 459.275213 | 8566641 | 27 | 40 | 0 | 6 | 0 | 0 |
| 459.311707 | 459.311598 | 2903669 | 28 | 44 | 0 | 5 | 0 | 0 |
| 460.176731 | 460.176561 | 1460696 | 27 | 27 | 1 | 6 | 0 | 0 |
| 461.051451 | 461.05142 | 1940539 | 24 | 14 | 0 | 10 | 0 | 0 |
| 461.087834 | 461.087806 | 3062207 | 25 | 18 | 0 | 9 | 0 | 0 |
| 461.109247 | 461.108935 | 1712961 | 22 | 22 | 0 | 11 | 0 | 0 |
| 461.124165 | 461.124191 | 4635203 | 26 | 22 | 0 | 8 | 0 | 0 |
| 461.145357 | 461.145321 | 3823941 | 23 | 26 | 0 | 10 | 0 | 0 |
| 461.148828 | 461.148691 | 2724422 | 20 | 30 | 0 | 10 | 1 | 0 |
| 461.160606 | 461.160577 | 6594119 | 27 | 26 | 0 | 7 | 0 | 0 |
| 461.181792 | 461.181706 | 9801801 | 24 | 30 | 0 | 9 | 0 | 0 |
| 461.185038 | 461.185077 | 2231114 | 21 | 34 | 0 | 9 | 1 | 0 |
| 461.197011 | 461.196962 | 8232011 | 28 | 30 | 0 | 6 | 0 | 0 |
| 461.218136 | 461.218092 | 13954125 | 25 | 34 | 0 | 8 | 0 | 0 |
| 461.233327 | 461.233348 | 7495759 | 29 | 34 | 0 | 5 | 0 | 0 |
| 461.25444 | 461.254477 | 8288338 | 26 | 38 | 0 | 7 | 0 | 0 |
| 461.269483 | 461.269733 | 3474003 | 30 | 38 | 0 | 4 | 0 | 0 |
| 461.290981 | 461.290863 | 4102230 | 27 | 42 | 0 | 6 | 0 | 0 |
| 462.156109 | 462.155826 | 1388214 | 26 | 25 | 1 | 7 | 0 | 0 |
| 463.067219 | 463.06707 | 2646298 | 24 | 16 | 0 | 10 | 0 | 0 |
| 463.10347 | 463.103456 | 4090910 | 25 | 20 | 0 | 9 | 0 | 0 |
| 463.106955 | 463.106827 | 1762590 | 22 | 24 | 0 | 9 | 1 | 0 |
| 463.124206 | 463.124585 | 1616416 | 22 | 24 | 0 | 11 | 0 | 0 |
| 463.139917 | 463.139841 | 4552482 | 26 | 24 | 0 | 8 | 0 | 0 |
| 463.143317 | 463.143212 | 2069794 | 23 | 28 | 0 | 8 | 1 | 0 |
| 463.161069 | 463.160971 | 4733732 | 23 | 28 | 0 | 10 | 0 | 0 |
| 463.164217 | 463.164341 | 1942821 | 20 | 32 | 0 | 10 | 1 | 0 |
| 463.176302 | 463.176227 | 8053030 | 27 | 28 | 0 | 7 | 0 | 0 |
| 463.179625 | 463.179598 | 1730470 | 24 | 32 | 0 | 7 | 1 | 0 |
| 463.197421 | 463.197356 | 9039144 | 24 | 32 | 0 | 9 | 0 | 0 |
| 463.212657 | 463.212612 | 10006826 | 28 | 32 | 0 | 6 | 0 | 0 |
| 463.233792 | 463.233742 | 10114348 | 25 | 36 | 0 | 8 | 0 | 0 |
| 463.249015 | 463.248998 | 8444206 | 29 | 36 | 0 | 5 | 0 | 0 |
| 463.270187 | 463.270127 | 4384048 | 26 | 40 | 0 | 7 | 0 | 0 |
| 463.285445 | 463.285383 | 3278642 | 30 | 40 | 0 | 4 | 0 | 0 |
| 463.306659 | 463.306513 | 1567284 | 27 | 44 | 0 | 6 | 0 | 0 |
| 465.082881 | 465.08272 | 2934771 | 24 | 18 | 0 | 10 | 0 | 0 |
| 465.098127 | 465.097976 | 1485813 | 28 | 18 | 0 | 7 | 0 | 0 |
| 465.118878 | 465.119106 | 4606455 | 25 | 22 | 0 | 9 | 0 | 0 |
| 465.122625 | 465.122477 | 2531319 | 22 | 26 | 0 | 9 | 1 | 0 |
| 465.134461 | 465.134362 | 1624825 | 29 | 22 | 0 | 6 | 0 | 0 |
| 465.140151 | 465.140235 | 2224633 | 22 | 26 | 0 | 11 | 0 | 0 |
| 465.155505 | 465.155491 | 7395835 | 26 | 26 | 0 | 8 | 0 | 0 |
| 465.158842 | 465.158862 | 3539707 | 23 | 30 | 0 | 8 | 1 | 0 |
| 465.170837 | 465.170747 | 2200061 | 30 | 26 | 0 | 5 | 0 | 0 |
| 465.176686 | 465.176621 | 4119805 | 23 | 30 | 0 | 10 | 0 | 0 |
| 465.191839 | 465.191877 | 10064895 | 27 | 30 | 0 | 7 | 0 | 0 |
| 465.195187 | 465.195248 | 2867199 | 24 | 34 | 0 | 7 | 1 | 0 |
| 465.207209 | 465.207133 | 2088192 | 31 | 30 | 0 | 4 | 0 | 0 |
| 465.213015 | 465.213006 | 6702081 | 24 | 34 | 0 | 9 | 0 | 0 |
| 465.228268 | 465.228262 | 12635650 | 28 | 34 | 0 | 6 | 0 | 0 |
| 465.249374 | 465.249392 | 5041669 | 25 | 38 | 0 | 8 | 0 | 0 |
| 465.264727 | 465.264648 | 9106950 | 29 | 38 | 0 | 5 | 0 | 0 |
| 465.286006 | 465.285777 | 1768969 | 26 | 42 | 0 | 7 | 0 | 0 |
| 465.301025 | 465.301033 | 2863114 | 30 | 42 | 0 | 4 | 0 | 0 |
| 467.040978 | 467.040856 | 1488576 | 26 | 12 | 0 | 9 | 0 | 0 |
| 467.062232 | 467.061985 | 1469122 | 23 | 16 | 0 | 11 | 0 | 0 |
| 467.076933 | 467.077241 | 1858371 | 27 | 16 | 0 | 8 | 0 | 0 |
| 467.098376 | 467.09837 | 3609797 | 24 | 20 | 0 | 10 | 0 | 0 |
| 467.101937 | 467.101741 | 1956038 | 21 | 24 | 0 | 10 | 1 | 0 |
| 467.113576 | 467.113627 | 1986887 | 28 | 20 | 0 | 7 | 0 | 0 |
| 467.134569 | 467.134756 | 4819401 | 25 | 24 | 0 | 9 | 0 | 0 |
| 467.138185 | 467.138127 | 4461258 | 22 | 28 | 0 | 9 | 1 | 0 |
| 467.150043 | 467.150012 | 2171339 | 29 | 24 | 0 | 6 | 0 | 0 |
| 467.155964 | 467.155885 | 1680587 | 22 | 28 | 0 | 11 | 0 | 0 |
| 467.171136 | 467.171141 | 8066765 | 26 | 28 | 0 | 8 | 0 | 0 |
| 467.174563 | 467.174512 | 4485453 | 23 | 32 | 0 | 8 | 1 | 0 |
| 467.186517 | 467.186398 | 2578383 | 30 | 28 | 0 | 5 | 0 | 0 |
| 467.192406 | 467.192271 | 2865359 | 23 | 32 | 0 | 10 | 0 | 0 |
| 467.207576 | 467.207527 | 12764881 | 27 | 32 | 0 | 7 | 0 | 0 |
| 467.211051 | 467.210898 | 2689745 | 24 | 36 | 0 | 7 | 1 | 0 |
| 467.222885 | 467.222783 | 2206418 | 31 | 32 | 0 | 4 | 0 | 0 |
| 467.228756 | 467.228656 | 3497171 | 24 | 36 | 0 | 9 | 0 | 0 |
| 467.243937 | 467.243912 | 13946580 | 28 | 36 | 0 | 6 | 0 | 0 |
| 467.265107 | 467.265042 | 1600247 | 25 | 40 | 0 | 8 | 0 | 0 |
| 467.280361 | 467.280298 | 8331992 | 29 | 40 | 0 | 5 | 0 | 0 |
| 467.316712 | 467.316683 | 2281948 | 30 | 44 | 0 | 4 | 0 | 0 |
| 469.056614 | 469.056506 | 1499275 | 26 | 14 | 0 | 9 | 0 | 0 |
| 469.077681 | 469.077635 | 1753997 | 23 | 18 | 0 | 11 | 0 | 0 |
| 469.092988 | 469.092891 | 2590350 | 27 | 18 | 0 | 8 | 0 | 0 |
| 469.114161 | 469.11402 | 2911633 | 24 | 22 | 0 | 10 | 0 | 0 |
| 469.117518 | 469.117391 | 2373905 | 21 | 26 | 0 | 10 | 1 | 0 |
| 469.129043 | 469.129277 | 2848402 | 28 | 22 | 0 | 7 | 0 | 0 |
| 469.150389 | 469.150406 | 5767060 | 25 | 26 | 0 | 9 | 0 | 0 |
| 469.153925 | 469.153777 | 6333333 | 22 | 30 | 0 | 9 | 1 | 0 |
| 469.165426 | 469.165662 | 2548502 | 29 | 26 | 0 | 6 | 0 | 0 |
| 469.186802 | 469.186791 | 11407256 | 26 | 30 | 0 | 8 | 0 | 0 |
| 469.190194 | 469.190162 | 4875160 | 23 | 34 | 0 | 8 | 1 | 0 |
| 469.201963 | 469.202048 | 3401113 | 30 | 30 | 0 | 5 | 0 | 0 |
| 469.223201 | 469.223177 | 15629211 | 27 | 34 | 0 | 7 | 0 | 0 |
| 469.238641 | 469.238433 | 2387357 | 31 | 34 | 0 | 4 | 0 | 0 |
| 469.244485 | 469.244306 | 1452957 | 24 | 38 | 0 | 9 | 0 | 0 |
| 469.25957 | 469.259562 | 13769631 | 28 | 38 | 0 | 6 | 0 | 0 |
| 469.295917 | 469.295948 | 8041379 | 29 | 42 | 0 | 5 | 0 | 0 |
| 469.332398 | 469.332334 | 2224294 | 30 | 46 | 0 | 4 | 0 | 0 |
| 470.182018 | 470.18204 | 1415673 | 25 | 29 | 1 | 8 | 0 | 0 |
| 471.07217 | 471.072156 | 2744398 | 26 | 16 | 0 | 9 | 0 | 0 |
| 471.092959 | 471.093285 | 1654224 | 23 | 20 | 0 | 11 | 0 | 0 |
| 471.108549 | 471.108541 | 3278418 | 27 | 20 | 0 | 8 | 0 | 0 |
| 471.12965 | 471.129671 | 3577684 | 24 | 24 | 0 | 10 | 0 | 0 |
| 471.133137 | 471.133041 | 3983444 | 21 | 28 | 0 | 10 | 1 | 0 |
| 471.145066 | 471.144927 | 3611733 | 28 | 24 | 0 | 7 | 0 | 0 |
| 471.165882 | 471.166056 | 7287895 | 25 | 28 | 0 | 9 | 0 | 0 |
| 471.169474 | 471.169427 | 5842008 | 22 | 32 | 0 | 9 | 1 | 0 |
| 471.181178 | 471.181312 | 3719129 | 29 | 28 | 0 | 6 | 0 | 0 |
| 471.202488 | 471.202442 | 13003867 | 26 | 32 | 0 | 8 | 0 | 0 |
| 471.205832 | 471.205812 | 3108699 | 23 | 36 | 0 | 8 | 1 | 0 |
| 471.217832 | 471.217698 | 3710172 | 30 | 32 | 0 | 5 | 0 | 0 |
| 471.238859 | 471.238827 | 15043678 | 27 | 36 | 0 | 7 | 0 | 0 |
| 471.254154 | 471.254083 | 2743904 | 31 | 36 | 0 | 4 | 0 | 0 |
| 471.275224 | 471.275213 | 10625122 | 28 | 40 | 0 | 6 | 0 | 0 |
| 471.311582 | 471.311598 | 8780901 | 29 | 44 | 0 | 5 | 0 | 0 |
| 471.348118 | 471.347984 | 1979496 | 30 | 48 | 0 | 4 | 0 | 0 |
| 473.051615 | 473.05142 | 1719046 | 25 | 14 | 0 | 10 | 0 | 0 |
| 473.087711 | 473.087806 | 3288074 | 26 | 18 | 0 | 9 | 0 | 0 |
| 473.109032 | 473.108935 | 1840396 | 23 | 22 | 0 | 11 | 0 | 0 |
| 473.112452 | 473.112306 | 1639948 | 20 | 26 | 0 | 11 | 1 | 0 |
| 473.124296 | 473.124191 | 4534541 | 27 | 22 | 0 | 8 | 0 | 0 |
| 473.145411 | 473.145321 | 4514063 | 24 | 26 | 0 | 10 | 0 | 0 |
| 473.14877 | 473.148691 | 3875087 | 21 | 30 | 0 | 10 | 1 | 0 |
| 473.160696 | 473.160577 | 4492048 | 28 | 26 | 0 | 7 | 0 | 0 |
| 473.181744 | 473.181706 | 8798994 | 25 | 30 | 0 | 9 | 0 | 0 |
| 473.185084 | 473.185077 | 3658514 | 22 | 34 | 0 | 9 | 1 | 0 |
| 473.197014 | 473.196962 | 5314324 | 29 | 30 | 0 | 6 | 0 | 0 |
| 473.218138 | 473.218092 | 13991189 | 26 | 34 | 0 | 8 | 0 | 0 |
| 473.233416 | 473.233348 | 5001495 | 30 | 34 | 0 | 5 | 0 | 0 |
| 473.254575 | 473.254477 | 12668185 | 27 | 38 | 0 | 7 | 0 | 0 |
| 473.26963 | 473.269733 | 2573338 | 31 | 38 | 0 | 4 | 0 | 0 |
| 473.29098 | 473.290863 | 7167772 | 28 | 42 | 0 | 6 | 0 | 0 |
| 473.327385 | 473.327248 | 3335967 | 29 | 46 | 0 | 5 | 0 | 0 |
| 475.067051 | 475.06707 | 2572729 | 25 | 16 | 0 | 10 | 0 | 0 |
| 475.10362 | 475.103456 | 3334588 | 26 | 20 | 0 | 9 | 0 | 0 |
| 475.124296 | 475.124585 | 1732286 | 23 | 24 | 0 | 11 | 0 | 0 |
| 475.128397 | 475.127956 | 1610174 | 20 | 28 | 0 | 11 | 1 | 0 |
| 475.139926 | 475.139841 | 4815295 | 27 | 24 | 0 | 8 | 0 | 0 |
| 475.143156 | 475.143212 | 1624511 | 24 | 28 | 0 | 8 | 1 | 0 |
| 475.160948 | 475.160971 | 5865921 | 24 | 28 | 0 | 10 | 0 | 0 |
| 475.16405 | 475.164341 | 2187969 | 21 | 32 | 0 | 10 | 1 | 0 |
| 475.176361 | 475.176227 | 6181315 | 28 | 28 | 0 | 7 | 0 | 0 |
| 475.197402 | 475.197356 | 10536388 | 25 | 32 | 0 | 9 | 0 | 0 |
| 475.201177 | 475.200727 | 1446084 | 22 | 36 | 0 | 9 | 1 | 0 |
| 475.212625 | 475.212612 | 6676934 | 29 | 32 | 0 | 6 | 0 | 0 |
| 475.233713 | 475.233742 | 12204487 | 26 | 36 | 0 | 8 | 0 | 0 |
| 475.249069 | 475.248998 | 4581833 | 30 | 36 | 0 | 5 | 0 | 0 |
| 475.270219 | 475.270127 | 8311243 | 27 | 40 | 0 | 7 | 0 | 0 |
| 475.284907 | 475.284731 | 1694412 | 23 | 44 | 2 | 6 | 1 | 0 |
| 475.306592 | 475.306513 | 3350222 | 28 | 44 | 0 | 6 | 0 | 0 |
| 477.046437 | 477.046335 | 1931871 | 24 | 14 | 0 | 11 | 0 | 0 |
| 477.082675 | 477.08272 | 2714722 | 25 | 18 | 0 | 10 | 0 | 0 |
| 477.11913 | 477.119106 | 4454501 | 26 | 22 | 0 | 9 | 0 | 0 |
| 477.122701 | 477.122477 | 1985894 | 23 | 26 | 0 | 9 | 1 | 0 |
| 477.134756 | 477.134362 | 1424230 | 30 | 22 | 0 | 6 | 0 | 0 |
| 477.140118 | 477.140235 | 2137703 | 23 | 26 | 0 | 11 | 0 | 0 |
| 477.155516 | 477.155491 | 5643880 | 27 | 26 | 0 | 8 | 0 | 0 |
| 477.158637 | 477.158862 | 2044104 | 24 | 30 | 0 | 8 | 1 | 0 |
| 477.176603 | 477.176621 | 5743722 | 24 | 30 | 0 | 10 | 0 | 0 |
| 477.191901 | 477.191877 | 7321707 | 28 | 30 | 0 | 7 | 0 | 0 |
| 477.213035 | 477.213006 | 11031149 | 25 | 34 | 0 | 9 | 0 | 0 |
| 477.228317 | 477.228262 | 8988270 | 29 | 34 | 0 | 6 | 0 | 0 |
| 477.249398 | 477.249392 | 8050288 | 26 | 38 | 0 | 8 | 0 | 0 |
| 477.26479 | 477.264648 | 5500529 | 30 | 38 | 0 | 5 | 0 | 0 |
| 477.301108 | 477.301033 | 2073972 | 31 | 42 | 0 | 4 | 0 | 0 |
| 477.322018 | 477.322163 | 1425142 | 28 | 46 | 0 | 6 | 0 | 0 |
| 479.061956 | 479.061985 | 1711615 | 24 | 16 | 0 | 11 | 0 | 0 |
| 479.077329 | 479.077241 | 1597952 | 28 | 16 | 0 | 8 | 0 | 0 |
| 479.098288 | 479.09837 | 2982658 | 25 | 20 | 0 | 10 | 0 | 0 |
| 479.101969 | 479.101741 | 1737730 | 22 | 24 | 0 | 10 | 1 | 0 |
| 479.113173 | 479.113627 | 1692419 | 29 | 20 | 0 | 7 | 0 | 0 |
| 479.134773 | 479.134756 | 4206597 | 26 | 24 | 0 | 9 | 0 | 0 |
| 479.13816 | 479.138127 | 2608645 | 23 | 28 | 0 | 9 | 1 | 0 |
| 479.15026 | 479.150012 | 1802246 | 30 | 24 | 0 | 6 | 0 | 0 |
| 479.155717 | 479.155885 | 2449414 | 23 | 28 | 0 | 11 | 0 | 0 |
| 479.171094 | 479.171141 | 6841096 | 27 | 28 | 0 | 8 | 0 | 0 |
| 479.174504 | 479.174512 | 3432200 | 24 | 32 | 0 | 8 | 1 | 0 |
| 479.186703 | 479.186398 | 1486217 | 31 | 28 | 0 | 5 | 0 | 0 |
| 479.192332 | 479.192271 | 5313801 | 24 | 32 | 0 | 10 | 0 | 0 |
| 479.207556 | 479.207527 | 9336074 | 28 | 32 | 0 | 7 | 0 | 0 |
| 479.210915 | 479.210898 | 1964554 | 25 | 36 | 0 | 7 | 1 | 0 |
| 479.222715 | 479.222783 | 1722315 | 32 | 32 | 0 | 4 | 0 | 0 |
| 479.228597 | 479.228656 | 6712076 | 25 | 36 | 0 | 9 | 0 | 0 |
| 479.243886 | 479.243912 | 10226445 | 29 | 36 | 0 | 6 | 0 | 0 |
| 479.265029 | 479.265042 | 3677711 | 26 | 40 | 0 | 8 | 0 | 0 |
| 479.280342 | 479.280298 | 4571408 | 30 | 40 | 0 | 5 | 0 | 0 |
| 479.301699 | 479.301427 | 1415185 | 27 | 44 | 0 | 7 | 0 | 0 |
| 479.319973 | 479.320054 | 2041619 | 28 | 48 | 0 | 4 | 1 | 0 |
| 481.077652 | 481.077635 | 1616788 | 24 | 18 | 0 | 11 | 0 | 0 |
| 481.092961 | 481.092891 | 1953685 | 28 | 18 | 0 | 8 | 0 | 0 |
| 481.114 | 481.11402 | 2811671 | 25 | 22 | 0 | 10 | 0 | 0 |
| 481.117458 | 481.117391 | 2264215 | 22 | 26 | 0 | 10 | 1 | 0 |
| 481.129366 | 481.129277 | 2261400 | 29 | 22 | 0 | 7 | 0 | 0 |
| 481.150422 | 481.150406 | 7882650 | 26 | 26 | 0 | 9 | 0 | 0 |
| 481.153885 | 481.153777 | 4354714 | 23 | 30 | 0 | 9 | 1 | 0 |
| 481.165824 | 481.165662 | 2360219 | 30 | 26 | 0 | 6 | 0 | 0 |
| 481.171241 | 481.171535 | 1966235 | 23 | 30 | 0 | 11 | 0 | 0 |
| 481.186806 | 481.186791 | 9373596 | 27 | 30 | 0 | 8 | 0 | 0 |
| 481.19027 | 481.190162 | 4883357 | 24 | 34 | 0 | 8 | 1 | 0 |
| 481.202139 | 481.202048 | 5278621 | 31 | 30 | 0 | 5 | 0 | 0 |
| 481.208025 | 481.207921 | 3359134 | 24 | 34 | 0 | 10 | 0 | 0 |
| 481.223206 | 481.223177 | 12588959 | 28 | 34 | 0 | 7 | 0 | 0 |
| 481.226646 | 481.226548 | 2049951 | 25 | 38 | 0 | 7 | 1 | 0 |
| 481.238321 | 481.238433 | 1898400 | 32 | 34 | 0 | 4 | 0 | 0 |
| 481.244148 | 481.244306 | 3046432 | 25 | 38 | 0 | 9 | 0 | 0 |
| 481.259615 | 481.259562 | 10536865 | 29 | 38 | 0 | 6 | 0 | 0 |
| 481.280724 | 481.280692 | 1398947 | 26 | 42 | 0 | 8 | 0 | 0 |
| 481.29592 | 481.295948 | 4370596 | 30 | 42 | 0 | 5 | 0 | 0 |
| 481.335747 | 481.335704 | 1438887 | 28 | 50 | 0 | 4 | 1 | 0 |
| 483.072164 | 483.072156 | 1999646 | 27 | 16 | 0 | 9 | 0 | 0 |
| 483.092944 | 483.093285 | 2101023 | 24 | 20 | 0 | 11 | 0 | 0 |
| 483.108559 | 483.108541 | 2435872 | 28 | 20 | 0 | 8 | 0 | 0 |
| 483.129779 | 483.129671 | 3767841 | 25 | 24 | 0 | 10 | 0 | 0 |
| 483.133055 | 483.133041 | 3943970 | 22 | 28 | 0 | 10 | 1 | 0 |
| 483.144953 | 483.144927 | 2963490 | 29 | 24 | 0 | 7 | 0 | 0 |
| 483.166086 | 483.166056 | 9109540 | 26 | 28 | 0 | 9 | 0 | 0 |
| 483.169547 | 483.169427 | 6144036 | 23 | 32 | 0 | 9 | 1 | 0 |
| 483.181296 | 483.181312 | 2841125 | 30 | 28 | 0 | 6 | 0 | 0 |
| 483.202468 | 483.202442 | 12011558 | 27 | 32 | 0 | 8 | 0 | 0 |
| 483.205906 | 483.205812 | 4108070 | 24 | 36 | 0 | 8 | 1 | 0 |
| 483.217882 | 483.217698 | 2902055 | 31 | 32 | 0 | 5 | 0 | 0 |
| 483.223724 | 483.223571 | 1542440 | 24 | 36 | 0 | 10 | 0 | 0 |
| 483.238859 | 483.238827 | 13671464 | 28 | 36 | 0 | 7 | 0 | 0 |
| 483.253832 | 483.254083 | 2155306 | 32 | 36 | 0 | 4 | 0 | 0 |
| 483.275263 | 483.275213 | 10965035 | 29 | 40 | 0 | 6 | 0 | 0 |
| 483.311609 | 483.311598 | 4643373 | 30 | 44 | 0 | 5 | 0 | 0 |
| 485.087913 | 485.087806 | 2610333 | 27 | 18 | 0 | 9 | 0 | 0 |
| 485.109037 | 485.108935 | 2288542 | 24 | 22 | 0 | 11 | 0 | 0 |
| 485.112007 | 485.112306 | 1500830 | 21 | 26 | 0 | 11 | 1 | 0 |
| 485.12408 | 485.124191 | 3222431 | 28 | 22 | 0 | 8 | 0 | 0 |
| 485.145401 | 485.145321 | 3759264 | 25 | 26 | 0 | 10 | 0 | 0 |
| 485.14867 | 485.148691 | 5501601 | 22 | 30 | 0 | 10 | 1 | 0 |
| 485.160414 | 485.160577 | 3973793 | 29 | 26 | 0 | 7 | 0 | 0 |
| 485.181708 | 485.181706 | 7895715 | 26 | 30 | 0 | 9 | 0 | 0 |
| 485.185134 | 485.185077 | 5502627 | 23 | 34 | 0 | 9 | 1 | 0 |
| 485.196998 | 485.196962 | 3623076 | 30 | 30 | 0 | 6 | 0 | 0 |
| 485.218173 | 485.218092 | 13098149 | 27 | 34 | 0 | 8 | 0 | 0 |
| 485.221714 | 485.221462 | 2607781 | 24 | 38 | 0 | 8 | 1 | 0 |
| 485.233339 | 485.233348 | 3223718 | 31 | 34 | 0 | 5 | 0 | 0 |
| 485.254505 | 485.254477 | 14252199 | 28 | 38 | 0 | 7 | 0 | 0 |
| 485.269764 | 485.269733 | 2444712 | 32 | 38 | 0 | 4 | 0 | 0 |
| 485.279137 | 485.278977 | 2671784 | 22 | 46 | 0 | 9 | 1 | 0 |
| 485.290945 | 485.290863 | 10924201 | 29 | 42 | 0 | 6 | 0 | 0 |
| 485.327305 | 485.327248 | 6876331 | 30 | 46 | 0 | 5 | 0 | 0 |
| 487.066931 | 487.06707 | 2260238 | 26 | 16 | 0 | 10 | 0 | 0 |
| 487.103482 | 487.103456 | 3394832 | 27 | 20 | 0 | 9 | 0 | 0 |
| 487.124718 | 487.124585 | 2458898 | 24 | 24 | 0 | 11 | 0 | 0 |
| 487.128013 | 487.127956 | 2458386 | 21 | 28 | 0 | 11 | 1 | 0 |
| 487.139753 | 487.139841 | 3806994 | 28 | 24 | 0 | 8 | 0 | 0 |
| 487.161119 | 487.160971 | 4914964 | 25 | 28 | 0 | 10 | 0 | 0 |
| 487.164391 | 487.164341 | 4910868 | 22 | 32 | 0 | 10 | 1 | 0 |
| 487.176161 | 487.176227 | 4380436 | 29 | 28 | 0 | 7 | 0 | 0 |
| 487.197437 | 487.197356 | 10149141 | 26 | 32 | 0 | 9 | 0 | 0 |
| 487.200705 | 487.200727 | 2998038 | 23 | 36 | 0 | 9 | 1 | 0 |
| 487.212694 | 487.212612 | 4487702 | 30 | 32 | 0 | 6 | 0 | 0 |
| 487.233727 | 487.233742 | 13979927 | 27 | 36 | 0 | 8 | 0 | 0 |
| 487.248932 | 487.248998 | 3525400 | 31 | 36 | 0 | 5 | 0 | 0 |
| 487.270168 | 487.270127 | 11704601 | 28 | 40 | 0 | 7 | 0 | 0 |
| 487.285258 | 487.285383 | 2108826 | 32 | 40 | 0 | 4 | 0 | 0 |
| 487.306525 | 487.306513 | 8832283 | 29 | 44 | 0 | 6 | 0 | 0 |
| 487.342903 | 487.342898 | 9095453 | 30 | 48 | 0 | 5 | 0 | 0 |
| 489.082784 | 489.08272 | 2567542 | 26 | 18 | 0 | 10 | 0 | 0 |
| 489.119129 | 489.119106 | 3756408 | 27 | 22 | 0 | 9 | 0 | 0 |
| 489.140246 | 489.140235 | 2416761 | 24 | 26 | 0 | 11 | 0 | 0 |
| 489.143719 | 489.143606 | 2231161 | 21 | 30 | 0 | 11 | 1 | 0 |
| 489.15554 | 489.155491 | 4436857 | 28 | 26 | 0 | 8 | 0 | 0 |
| 489.176657 | 489.176621 | 6373754 | 25 | 30 | 0 | 10 | 0 | 0 |
| 489.180112 | 489.179992 | 2284922 | 22 | 34 | 0 | 10 | 1 | 0 |
| 489.191983 | 489.191877 | 5326715 | 29 | 30 | 0 | 7 | 0 | 0 |
| 489.213027 | 489.213006 | 11098492 | 26 | 34 | 0 | 9 | 0 | 0 |
| 489.22819 | 489.228262 | 5520765 | 30 | 34 | 0 | 6 | 0 | 0 |
| 489.24944 | 489.249392 | 12423550 | 27 | 38 | 0 | 8 | 0 | 0 |
| 489.264749 | 489.264648 | 3510142 | 31 | 38 | 0 | 5 | 0 | 0 |
| 489.285794 | 489.285777 | 7679360 | 28 | 42 | 0 | 7 | 0 | 0 |
| 489.301169 | 489.301033 | 1688960 | 32 | 42 | 0 | 4 | 0 | 0 |
| 489.322241 | 489.322163 | 5411201 | 29 | 46 | 0 | 6 | 0 | 0 |
| 491.061962 | 491.061985 | 1876943 | 25 | 16 | 0 | 11 | 0 | 0 |
| 491.09854 | 491.09837 | 2906064 | 26 | 20 | 0 | 10 | 0 | 0 |
| 491.113685 | 491.113627 | 1523665 | 30 | 20 | 0 | 7 | 0 | 0 |
| 491.134825 | 491.134756 | 4239826 | 27 | 24 | 0 | 9 | 0 | 0 |
| 491.13821 | 491.138127 | 1761746 | 24 | 28 | 0 | 9 | 1 | 0 |
| 491.150464 | 491.150012 | 1467347 | 31 | 24 | 0 | 6 | 0 | 0 |
| 491.156005 | 491.155885 | 2835411 | 24 | 28 | 0 | 11 | 0 | 0 |
| 491.159497 | 491.159256 | 1443795 | 21 | 32 | 0 | 11 | 1 | 0 |
| 491.171128 | 491.171141 | 5386708 | 28 | 28 | 0 | 8 | 0 | 0 |
| 491.174295 | 491.174512 | 2286804 | 25 | 32 | 0 | 8 | 1 | 0 |
| 491.192347 | 491.192271 | 7215060 | 25 | 32 | 0 | 10 | 0 | 0 |
| 491.207535 | 491.207527 | 7449045 | 29 | 32 | 0 | 7 | 0 | 0 |
| 491.211083 | 491.210898 | 2086869 | 26 | 36 | 0 | 7 | 1 | 0 |
| 491.228689 | 491.228656 | 10073558 | 26 | 36 | 0 | 9 | 0 | 0 |
| 491.243853 | 491.243912 | 5973975 | 30 | 36 | 0 | 6 | 0 | 0 |
| 491.265185 | 491.265042 | 7308759 | 27 | 40 | 0 | 8 | 0 | 0 |
| 491.280242 | 491.280298 | 3624408 | 31 | 40 | 0 | 5 | 0 | 0 |
| 491.301375 | 491.301427 | 3561433 | 28 | 44 | 0 | 7 | 0 | 0 |
| 492.166546 | 492.16639 | 1532923 | 27 | 27 | 1 | 8 | 0 | 0 |
| 493.077365 | 493.077635 | 1816093 | 25 | 18 | 0 | 11 | 0 | 0 |
| 493.113956 | 493.11402 | 2853791 | 26 | 22 | 0 | 10 | 0 | 0 |
| 493.116849 | 493.117011 | 1417247 | 25 | 23 | 2 | 7 | 0 | 1 |
| 493.129421 | 493.129277 | 1507103 | 30 | 22 | 0 | 7 | 0 | 0 |
| 493.150443 | 493.150406 | 4565024 | 27 | 26 | 0 | 9 | 0 | 0 |
| 493.153513 | 493.153777 | 3472416 | 24 | 30 | 0 | 9 | 1 | 0 |
| 493.16609 | 493.165662 | 1434144 | 31 | 26 | 0 | 6 | 0 | 0 |
| 493.171502 | 493.171535 | 2699809 | 24 | 30 | 0 | 11 | 0 | 0 |
| 493.18679 | 493.186791 | 7319073 | 28 | 30 | 0 | 8 | 0 | 0 |
| 493.190267 | 493.190162 | 2875425 | 25 | 34 | 0 | 8 | 1 | 0 |
| 493.201991 | 493.202048 | 1854882 | 32 | 30 | 0 | 5 | 0 | 0 |
| 493.207808 | 493.207921 | 6369826 | 25 | 34 | 0 | 10 | 0 | 0 |
| 493.22325 | 493.223177 | 8669730 | 29 | 34 | 0 | 7 | 0 | 0 |
| 493.244303 | 493.244306 | 5685283 | 26 | 38 | 0 | 9 | 0 | 0 |
| 493.259569 | 493.259562 | 5903908 | 30 | 38 | 0 | 6 | 0 | 0 |
| 493.295723 | 493.295948 | 2859557 | 31 | 42 | 0 | 5 | 0 | 0 |
| 493.3354 | 493.335704 | 1580070 | 29 | 50 | 0 | 4 | 1 | 0 |
| 495.093318 | 495.093285 | 1938272 | 25 | 20 | 0 | 11 | 0 | 0 |
| 495.108422 | 495.108541 | 2105696 | 29 | 20 | 0 | 8 | 0 | 0 |
| 495.129405 | 495.129671 | 3208033 | 26 | 24 | 0 | 10 | 0 | 0 |
| 495.13307 | 495.133041 | 2926689 | 23 | 28 | 0 | 10 | 1 | 0 |
| 495.145019 | 495.144927 | 1966689 | 30 | 24 | 0 | 7 | 0 | 0 |
| 495.16602 | 495.166056 | 5470818 | 27 | 28 | 0 | 9 | 0 | 0 |
| 495.16948 | 495.169427 | 4862050 | 24 | 32 | 0 | 9 | 1 | 0 |
| 495.180963 | 495.181312 | 1503874 | 31 | 28 | 0 | 6 | 0 | 0 |
| 495.187183 | 495.187185 | 2588515 | 24 | 32 | 0 | 11 | 0 | 0 |
| 495.202465 | 495.202442 | 9211491 | 28 | 32 | 0 | 8 | 0 | 0 |
| 495.205835 | 495.205812 | 3501667 | 25 | 36 | 0 | 8 | 1 | 0 |
| 495.217254 | 495.217698 | 1504099 | 32 | 32 | 0 | 5 | 0 | 0 |
| 495.223573 | 495.223571 | 3666532 | 25 | 36 | 0 | 10 | 0 | 0 |
| 495.238843 | 495.238827 | 10638948 | 29 | 36 | 0 | 7 | 0 | 0 |
| 495.254038 | 495.254083 | 1758565 | 33 | 36 | 0 | 4 | 0 | 0 |
| 495.259897 | 495.259956 | 2766949 | 26 | 40 | 0 | 9 | 0 | 0 |
| 495.27512 | 495.275213 | 5705317 | 30 | 40 | 0 | 6 | 0 | 0 |
| 495.31168 | 495.311598 | 2225254 | 31 | 44 | 0 | 5 | 0 | 0 |
| 497.088009 | 497.087806 | 1729302 | 28 | 18 | 0 | 9 | 0 | 0 |
| 497.108995 | 497.108935 | 2216343 | 25 | 22 | 0 | 11 | 0 | 0 |
| 497.112218 | 497.112306 | 1542807 | 22 | 26 | 0 | 11 | 1 | 0 |
| 497.124178 | 497.124191 | 2333335 | 29 | 22 | 0 | 8 | 0 | 0 |
| 497.145386 | 497.145321 | 3987608 | 26 | 26 | 0 | 10 | 0 | 0 |
| 497.148588 | 497.148691 | 4241816 | 23 | 30 | 0 | 10 | 1 | 0 |
| 497.160627 | 497.160577 | 2877592 | 30 | 26 | 0 | 7 | 0 | 0 |
| 497.18173 | 497.181706 | 6957209 | 27 | 30 | 0 | 9 | 0 | 0 |
| 497.185107 | 497.185077 | 6267545 | 24 | 34 | 0 | 9 | 1 | 0 |
| 497.196907 | 497.196962 | 2601369 | 31 | 30 | 0 | 6 | 0 | 0 |
| 497.218099 | 497.218092 | 10630809 | 28 | 34 | 0 | 8 | 0 | 0 |
| 497.221624 | 497.221462 | 3151513 | 25 | 38 | 0 | 8 | 1 | 0 |
| 497.233464 | 497.233348 | 2752666 | 32 | 34 | 0 | 5 | 0 | 0 |
| 497.254505 | 497.254477 | 11339418 | 29 | 38 | 0 | 7 | 0 | 0 |
| 497.290955 | 497.290863 | 6238875 | 30 | 42 | 0 | 6 | 0 | 0 |
| 499.067095 | 499.06707 | 1480001 | 27 | 16 | 0 | 10 | 0 | 0 |
| 499.103368 | 499.103456 | 2701506 | 28 | 20 | 0 | 9 | 0 | 0 |
| 499.124369 | 499.124585 | 2752451 | 25 | 24 | 0 | 11 | 0 | 0 |
| 499.127979 | 499.127956 | 2636227 | 22 | 28 | 0 | 11 | 1 | 0 |
| 499.13976 | 499.139841 | 2618819 | 29 | 24 | 0 | 8 | 0 | 0 |
| 499.160931 | 499.160971 | 5653699 | 26 | 28 | 0 | 10 | 0 | 0 |
| 499.164458 | 499.164341 | 5637827 | 23 | 32 | 0 | 10 | 1 | 0 |
| 499.176279 | 499.176227 | 3309763 | 30 | 28 | 0 | 7 | 0 | 0 |
| 499.197364 | 499.197356 | 8855236 | 27 | 32 | 0 | 9 | 0 | 0 |
| 499.200804 | 499.200727 | 5059780 | 24 | 36 | 0 | 9 | 1 | 0 |
| 499.212696 | 499.212612 | 3735748 | 31 | 32 | 0 | 6 | 0 | 0 |
| 499.233741 | 499.233742 | 12575428 | 28 | 36 | 0 | 8 | 0 | 0 |
| 499.237361 | 499.237112 | 2125509 | 25 | 40 | 0 | 8 | 1 | 0 |
| 499.24905 | 499.248998 | 2565829 | 32 | 36 | 0 | 5 | 0 | 0 |
| 499.270069 | 499.270127 | 10799813 | 29 | 40 | 0 | 7 | 0 | 0 |
| 499.285371 | 499.285383 | 1806277 | 33 | 40 | 0 | 4 | 0 | 0 |
| 499.306558 | 499.306513 | 7582406 | 30 | 44 | 0 | 6 | 0 | 0 |
| 501.082833 | 501.08272 | 1924323 | 27 | 18 | 0 | 10 | 0 | 0 |
| 501.119202 | 501.119106 | 3285731 | 28 | 22 | 0 | 9 | 0 | 0 |
| 501.140123 | 501.140235 | 2782948 | 25 | 26 | 0 | 11 | 0 | 0 |
| 501.143728 | 501.143606 | 3212004 | 22 | 30 | 0 | 11 | 1 | 0 |
| 501.155431 | 501.155491 | 3782372 | 29 | 26 | 0 | 8 | 0 | 0 |
| 501.176639 | 501.176621 | 5774564 | 26 | 30 | 0 | 10 | 0 | 0 |
| 501.179977 | 501.179992 | 4594404 | 23 | 34 | 0 | 10 | 1 | 0 |
| 501.191968 | 501.191877 | 4305124 | 30 | 30 | 0 | 7 | 0 | 0 |
| 501.213022 | 501.213006 | 11277028 | 27 | 34 | 0 | 9 | 0 | 0 |
| 501.216604 | 501.216377 | 3014373 | 24 | 38 | 0 | 9 | 1 | 0 |
| 501.228152 | 501.228262 | 3755621 | 31 | 34 | 0 | 6 | 0 | 0 |
| 501.249371 | 501.249392 | 13258469 | 28 | 38 | 0 | 8 | 0 | 0 |
| 501.264551 | 501.264648 | 2989797 | 32 | 38 | 0 | 5 | 0 | 0 |
| 501.285803 | 501.285777 | 10844901 | 29 | 42 | 0 | 7 | 0 | 0 |
| 501.322047 | 501.322163 | 7576806 | 30 | 46 | 0 | 6 | 0 | 0 |
| 503.061768 | 503.061985 | 1415418 | 26 | 16 | 0 | 11 | 0 | 0 |
| 503.098361 | 503.09837 | 2579706 | 27 | 20 | 0 | 10 | 0 | 0 |
| 503.119637 | 503.1195 | 1840890 | 24 | 24 | 0 | 12 | 0 | 0 |
| 503.134585 | 503.134756 | 3507450 | 28 | 24 | 0 | 9 | 0 | 0 |
| 503.156112 | 503.155885 | 3257594 | 25 | 28 | 0 | 11 | 0 | 0 |
| 503.159387 | 503.159256 | 2888954 | 22 | 32 | 0 | 11 | 1 | 0 |
| 503.171159 | 503.171141 | 4487419 | 29 | 28 | 0 | 8 | 0 | 0 |
| 503.192285 | 503.192271 | 7824123 | 26 | 32 | 0 | 10 | 0 | 0 |
| 503.195503 | 503.195642 | 3191547 | 23 | 36 | 0 | 10 | 1 | 0 |
| 503.207467 | 503.207527 | 4764155 | 30 | 32 | 0 | 7 | 0 | 0 |
| 503.228639 | 503.228656 | 11590395 | 27 | 36 | 0 | 9 | 0 | 0 |
| 503.243914 | 503.243912 | 4333819 | 31 | 36 | 0 | 6 | 0 | 0 |
| 503.265017 | 503.265042 | 11304699 | 28 | 40 | 0 | 8 | 0 | 0 |
| 503.280327 | 503.280298 | 2292476 | 32 | 40 | 0 | 5 | 0 | 0 |
| 503.301316 | 503.301427 | 6915836 | 29 | 44 | 0 | 7 | 0 | 0 |
| 503.337835 | 503.337813 | 7669756 | 30 | 48 | 0 | 6 | 0 | 0 |
| 505.077752 | 505.077635 | 1543687 | 26 | 18 | 0 | 11 | 0 | 0 |
| 505.113907 | 505.11402 | 3625735 | 27 | 22 | 0 | 10 | 0 | 0 |
| 505.150337 | 505.150406 | 3898887 | 28 | 26 | 0 | 9 | 0 | 0 |
| 505.153682 | 505.153777 | 2040455 | 25 | 30 | 0 | 9 | 1 | 0 |
| 505.1653 | 505.165662 | 1490695 | 32 | 26 | 0 | 6 | 0 | 0 |
| 505.171516 | 505.171535 | 3715847 | 25 | 30 | 0 | 11 | 0 | 0 |
| 505.186762 | 505.186791 | 5419784 | 29 | 30 | 0 | 8 | 0 | 0 |
| 505.190132 | 505.190162 | 1915399 | 26 | 34 | 0 | 8 | 1 | 0 |
| 505.207956 | 505.207921 | 7888648 | 26 | 34 | 0 | 10 | 0 | 0 |
| 505.223264 | 505.223177 | 6505224 | 30 | 34 | 0 | 7 | 0 | 0 |
| 505.226257 | 505.226548 | 1743112 | 27 | 38 | 0 | 7 | 1 | 0 |
| 505.24434 | 505.244306 | 9698056 | 27 | 38 | 0 | 9 | 0 | 0 |
| 505.259508 | 505.259562 | 3922184 | 31 | 38 | 0 | 6 | 0 | 0 |
| 505.280716 | 505.280692 | 6650120 | 28 | 42 | 0 | 8 | 0 | 0 |
| 505.295972 | 505.295948 | 1901832 | 32 | 42 | 0 | 5 | 0 | 0 |
| 505.317125 | 505.317077 | 2978568 | 29 | 46 | 0 | 7 | 0 | 0 |
| 507.093146 | 507.093285 | 2349579 | 26 | 20 | 0 | 11 | 0 | 0 |
| 507.108769 | 507.108541 | 1441547 | 30 | 20 | 0 | 8 | 0 | 0 |
| 507.129586 | 507.129671 | 2907019 | 27 | 24 | 0 | 10 | 0 | 0 |
| 507.13319 | 507.133041 | 2387723 | 24 | 28 | 0 | 10 | 1 | 0 |
| 507.145261 | 507.144927 | 1652747 | 31 | 24 | 0 | 7 | 0 | 0 |
| 507.166019 | 507.166056 | 4895499 | 28 | 28 | 0 | 9 | 0 | 0 |
| 507.168854 | 507.169047 | 2914571 | 27 | 29 | 2 | 6 | 0 | 1 |
| 507.186995 | 507.187185 | 3900683 | 25 | 32 | 0 | 11 | 0 | 0 |
| 507.202513 | 507.202442 | 6911755 | 29 | 32 | 0 | 8 | 0 | 0 |
| 507.2058 | 507.205812 | 2486027 | 26 | 36 | 0 | 8 | 1 | 0 |
| 507.223525 | 507.223571 | 5626635 | 26 | 36 | 0 | 10 | 0 | 0 |
| 507.23885 | 507.238827 | 6281995 | 30 | 36 | 0 | 7 | 0 | 0 |
| 507.259948 | 507.259956 | 5712651 | 27 | 40 | 0 | 9 | 0 | 0 |
| 507.27531 | 507.275213 | 4171531 | 31 | 40 | 0 | 6 | 0 | 0 |
| 507.29639 | 507.296342 | 2911243 | 28 | 44 | 0 | 8 | 0 | 0 |
| 507.311272 | 507.311598 | 1649163 | 32 | 44 | 0 | 5 | 0 | 0 |
| 507.332653 | 507.332727 | 1437707 | 29 | 48 | 0 | 7 | 0 | 0 |
| 508.172651 | 508.172538 | 1521930 | 26 | 27 | 3 | 8 | 0 | 0 |
| 509.088133 | 509.087806 | 1613830 | 29 | 18 | 0 | 9 | 0 | 0 |
| 509.108693 | 509.108935 | 3033350 | 26 | 22 | 0 | 11 | 0 | 0 |
| 509.124103 | 509.124191 | 1946374 | 30 | 22 | 0 | 8 | 0 | 0 |
| 509.1453 | 509.145321 | 3408646 | 27 | 26 | 0 | 10 | 0 | 0 |
| 509.148814 | 509.148691 | 3831558 | 24 | 30 | 0 | 10 | 1 | 0 |
| 509.160627 | 509.160577 | 2036998 | 31 | 26 | 0 | 7 | 0 | 0 |
| 509.181729 | 509.181706 | 5779206 | 28 | 30 | 0 | 9 | 0 | 0 |
| 509.185035 | 509.185077 | 4718342 | 25 | 34 | 0 | 9 | 1 | 0 |
| 509.196896 | 509.196962 | 1695238 | 32 | 30 | 0 | 6 | 0 | 0 |
| 509.202934 | 509.202835 | 2969350 | 25 | 34 | 0 | 11 | 0 | 0 |
| 509.217985 | 509.218092 | 7546630 | 29 | 34 | 0 | 8 | 0 | 0 |
| 509.221598 | 509.221462 | 2968838 | 26 | 38 | 0 | 8 | 1 | 0 |
| 509.233259 | 509.233348 | 1409670 | 33 | 34 | 0 | 5 | 0 | 0 |
| 509.239163 | 509.239221 | 3157766 | 26 | 38 | 0 | 10 | 0 | 0 |
| 509.254386 | 509.254477 | 6941446 | 30 | 38 | 0 | 7 | 0 | 0 |
| 509.269844 | 509.269733 | 1912581 | 34 | 38 | 0 | 4 | 0 | 0 |
| 509.27575 | 509.275606 | 1845893 | 27 | 42 | 0 | 9 | 0 | 0 |
| 509.290845 | 509.290863 | 3388677 | 31 | 42 | 0 | 6 | 0 | 0 |
| 511.103208 | 511.103456 | 2259193 | 29 | 20 | 0 | 9 | 0 | 0 |
| 511.124601 | 511.124585 | 2552057 | 26 | 24 | 0 | 11 | 0 | 0 |
| 511.12775 | 511.127956 | 2435833 | 23 | 28 | 0 | 11 | 1 | 0 |
| 511.139771 | 511.139841 | 2767609 | 30 | 24 | 0 | 8 | 0 | 0 |
| 511.16094 | 511.160971 | 4419321 | 27 | 28 | 0 | 10 | 0 | 0 |
| 511.164394 | 511.164341 | 5139704 | 24 | 32 | 0 | 10 | 1 | 0 |
| 511.175801 | 511.176227 | 1967096 | 31 | 28 | 0 | 7 | 0 | 0 |
| 511.197331 | 511.197356 | 7375608 | 28 | 32 | 0 | 9 | 0 | 0 |
| 511.200777 | 511.200727 | 5273848 | 25 | 36 | 0 | 9 | 1 | 0 |
| 511.212219 | 511.212612 | 2418680 | 32 | 32 | 0 | 6 | 0 | 0 |
| 511.21807 | 511.218486 | 1546232 | 25 | 36 | 0 | 11 | 0 | 0 |
| 511.233755 | 511.233742 | 9640696 | 29 | 36 | 0 | 8 | 0 | 0 |
| 511.237188 | 511.237112 | 2446072 | 26 | 40 | 0 | 8 | 1 | 0 |
| 511.249012 | 511.248998 | 2065144 | 33 | 36 | 0 | 5 | 0 | 0 |
| 511.254329 | 511.253839 | 1673975 | 20 | 41 | 4 | 9 | 0 | 1 |
| 511.270119 | 511.270127 | 7248632 | 30 | 40 | 0 | 7 | 0 | 0 |
| 511.285323 | 511.285383 | 1429239 | 34 | 40 | 0 | 4 | 0 | 0 |
| 511.306516 | 511.306513 | 3065079 | 31 | 44 | 0 | 6 | 0 | 0 |
| 513.082687 | 513.08272 | 1840868 | 28 | 18 | 0 | 10 | 0 | 0 |
| 513.103421 | 513.10385 | 1529060 | 25 | 22 | 0 | 12 | 0 | 0 |
| 513.107113 | 513.107221 | 1710307 | 22 | 26 | 0 | 12 | 1 | 0 |
| 513.118941 | 513.119106 | 2484963 | 29 | 22 | 0 | 9 | 0 | 0 |
| 513.139855 | 513.140235 | 2138595 | 26 | 26 | 0 | 11 | 0 | 0 |
| 513.143592 | 513.143606 | 3231459 | 23 | 30 | 0 | 11 | 1 | 0 |
| 513.155543 | 513.155491 | 2432483 | 30 | 26 | 0 | 8 | 0 | 0 |
| 513.176584 | 513.176621 | 5351139 | 27 | 30 | 0 | 10 | 0 | 0 |
| 513.179988 | 513.179992 | 6696675 | 24 | 34 | 0 | 10 | 1 | 0 |
| 513.191685 | 513.191877 | 2884578 | 31 | 30 | 0 | 7 | 0 | 0 |
| 513.21295 | 513.213006 | 9167586 | 28 | 34 | 0 | 9 | 0 | 0 |
| 513.216524 | 513.216377 | 3890402 | 25 | 38 | 0 | 9 | 1 | 0 |
| 513.228279 | 513.228262 | 3167970 | 32 | 34 | 0 | 6 | 0 | 0 |
| 513.24942 | 513.249392 | 11246306 | 29 | 38 | 0 | 8 | 0 | 0 |
| 513.264765 | 513.264648 | 1897185 | 33 | 38 | 0 | 5 | 0 | 0 |
| 513.285802 | 513.285777 | 7752417 | 30 | 42 | 0 | 7 | 0 | 0 |
| 513.322318 | 513.322163 | 1989857 | 31 | 46 | 0 | 6 | 0 | 0 |
| 515.098368 | 515.09837 | 2381511 | 28 | 20 | 0 | 10 | 0 | 0 |
| 515.134569 | 515.134756 | 2795974 | 29 | 24 | 0 | 9 | 0 | 0 |
| 515.155928 | 515.155885 | 2913990 | 26 | 28 | 0 | 11 | 0 | 0 |
| 515.159293 | 515.159256 | 3623622 | 23 | 32 | 0 | 11 | 1 | 0 |
| 515.171262 | 515.171141 | 2804934 | 30 | 28 | 0 | 8 | 0 | 0 |
| 515.192232 | 515.192271 | 6427333 | 27 | 32 | 0 | 10 | 0 | 0 |
| 515.195542 | 515.195642 | 4129477 | 24 | 36 | 0 | 10 | 1 | 0 |
| 515.207589 | 515.207527 | 3355333 | 31 | 32 | 0 | 7 | 0 | 0 |
| 515.228626 | 515.228656 | 11159236 | 28 | 36 | 0 | 9 | 0 | 0 |
| 515.232017 | 515.232027 | 2024773 | 25 | 40 | 0 | 9 | 1 | 0 |
| 515.243968 | 515.243912 | 3159236 | 32 | 36 | 0 | 6 | 0 | 0 |
| 515.265044 | 515.265042 | 12174020 | 29 | 40 | 0 | 8 | 0 | 0 |
| 515.268532 | 515.268413 | 2944196 | 26 | 44 | 0 | 8 | 1 | 0 |
| 515.280429 | 515.280298 | 2035396 | 33 | 40 | 0 | 5 | 0 | 0 |
| 515.301427 | 515.301427 | 8295108 | 30 | 44 | 0 | 7 | 0 | 0 |
| 517.077742 | 517.077635 | 1513123 | 27 | 18 | 0 | 11 | 0 | 0 |
| 517.114022 | 517.11402 | 2658466 | 28 | 22 | 0 | 10 | 0 | 0 |
| 517.138434 | 517.138521 | 1607330 | 22 | 30 | 0 | 12 | 1 | 0 |
| 517.150462 | 517.150406 | 2988194 | 29 | 26 | 0 | 9 | 0 | 0 |
| 517.171521 | 517.171535 | 3885473 | 26 | 30 | 0 | 11 | 0 | 0 |
| 517.175138 | 517.174906 | 2629281 | 23 | 34 | 0 | 11 | 1 | 0 |
| 517.186755 | 517.186791 | 4002977 | 30 | 30 | 0 | 8 | 0 | 0 |
| 517.20793 | 517.207921 | 8181409 | 27 | 34 | 0 | 10 | 0 | 0 |
| 517.211428 | 517.211292 | 2299552 | 24 | 38 | 0 | 10 | 1 | 0 |
| 517.22307 | 517.223177 | 4108448 | 31 | 34 | 0 | 7 | 0 | 0 |
| 517.24436 | 517.244306 | 11635360 | 28 | 38 | 0 | 9 | 0 | 0 |
| 517.259301 | 517.259562 | 3396512 | 32 | 38 | 0 | 6 | 0 | 0 |
| 517.280625 | 517.280692 | 9084063 | 29 | 42 | 0 | 8 | 0 | 0 |
| 517.284008 | 517.284063 | 14975647 | 26 | 46 | 0 | 8 | 1 | 0 |
| 517.295979 | 517.295948 | 1844895 | 33 | 42 | 0 | 5 | 0 | 0 |
| 517.317049 | 517.317077 | 9725598 | 30 | 46 | 0 | 7 | 0 | 0 |
| 519.092817 | 519.093285 | 1996408 | 27 | 20 | 0 | 11 | 0 | 0 |
| 519.12956 | 519.129671 | 2397687 | 28 | 24 | 0 | 10 | 0 | 0 |
| 519.150798 | 519.1508 | 1968503 | 25 | 28 | 0 | 12 | 0 | 0 |
| 519.165885 | 519.166056 | 3466358 | 29 | 28 | 0 | 9 | 0 | 0 |
| 519.169306 | 519.169427 | 2178422 | 26 | 32 | 0 | 9 | 1 | 0 |
| 519.187033 | 519.187185 | 4268022 | 26 | 32 | 0 | 11 | 0 | 0 |
| 519.202254 | 519.202442 | 4469110 | 30 | 32 | 0 | 8 | 0 | 0 |
| 519.20595 | 519.205812 | 2197621 | 27 | 36 | 0 | 8 | 1 | 0 |
| 519.223601 | 519.223571 | 8615541 | 27 | 36 | 0 | 10 | 0 | 0 |
| 519.238837 | 519.238827 | 3933813 | 31 | 36 | 0 | 7 | 0 | 0 |
| 519.241829 | 519.242198 | 1434741 | 28 | 40 | 0 | 7 | 1 | 0 |
| 519.259907 | 519.259956 | 9127540 | 28 | 40 | 0 | 9 | 0 | 0 |
| 519.27522 | 519.275213 | 3443828 | 32 | 40 | 0 | 6 | 0 | 0 |
| 519.296238 | 519.296342 | 6094452 | 29 | 44 | 0 | 8 | 0 | 0 |
| 519.311139 | 519.311598 | 1463923 | 33 | 44 | 0 | 5 | 0 | 0 |
| 519.332713 | 519.332727 | 5856883 | 30 | 48 | 0 | 7 | 0 | 0 |
| 521.072443 | 521.07255 | 1539656 | 26 | 18 | 0 | 12 | 0 | 0 |
| 521.108823 | 521.108935 | 2069063 | 27 | 22 | 0 | 11 | 0 | 0 |
| 521.124067 | 521.124191 | 1471558 | 31 | 22 | 0 | 8 | 0 | 0 |
| 521.144997 | 521.145321 | 3485254 | 28 | 26 | 0 | 10 | 0 | 0 |
| 521.148761 | 521.148691 | 2164038 | 25 | 30 | 0 | 10 | 1 | 0 |
| 521.165891 | 521.165418 | 1984325 | 19 | 31 | 4 | 11 | 0 | 1 |
| 521.181549 | 521.181706 | 4053573 | 29 | 30 | 0 | 9 | 0 | 0 |
| 521.185067 | 521.185077 | 2943557 | 26 | 34 | 0 | 9 | 1 | 0 |
| 521.202771 | 521.202835 | 4832836 | 26 | 34 | 0 | 11 | 0 | 0 |
| 521.218066 | 521.218092 | 5054532 | 30 | 34 | 0 | 8 | 0 | 0 |
| 521.221 | 521.221462 | 2738500 | 27 | 38 | 0 | 8 | 1 | 0 |
| 521.23923 | 521.239221 | 6369859 | 27 | 38 | 0 | 10 | 0 | 0 |
| 521.254455 | 521.254477 | 4785731 | 31 | 38 | 0 | 7 | 0 | 0 |
| 521.275559 | 521.275606 | 4983874 | 28 | 42 | 0 | 9 | 0 | 0 |
| 521.290914 | 521.290863 | 3012674 | 32 | 42 | 0 | 6 | 0 | 0 |
| 521.311957 | 521.311992 | 2626113 | 29 | 46 | 0 | 8 | 0 | 0 |
| 523.087561 | 523.087548 | 1660816 | 18 | 24 | 2 | 14 | 1 | 0 |
| 523.103291 | 523.103456 | 1773584 | 30 | 20 | 0 | 9 | 0 | 0 |
| 523.124656 | 523.124585 | 2936335 | 27 | 24 | 0 | 11 | 0 | 0 |
| 523.128098 | 523.127956 | 1549839 | 24 | 28 | 0 | 11 | 1 | 0 |
| 523.139844 | 523.139841 | 1474575 | 31 | 24 | 0 | 8 | 0 | 0 |
| 523.160859 | 523.160971 | 3749902 | 28 | 28 | 0 | 10 | 0 | 0 |
| 523.164269 | 523.164341 | 3857422 | 25 | 32 | 0 | 10 | 1 | 0 |
| 523.176179 | 523.176227 | 1965838 | 32 | 28 | 0 | 7 | 0 | 0 |
| 523.182268 | 523.1821 | 1617933 | 25 | 32 | 0 | 12 | 0 | 0 |
| 523.197483 | 523.197356 | 5275149 | 29 | 32 | 0 | 9 | 0 | 0 |
| 523.200802 | 523.200727 | 4193293 | 26 | 36 | 0 | 9 | 1 | 0 |
| 523.212748 | 523.212612 | 1538061 | 33 | 32 | 0 | 6 | 0 | 0 |
| 523.218555 | 523.218486 | 3243020 | 26 | 36 | 0 | 11 | 0 | 0 |
| 523.233641 | 523.233742 | 6097420 | 30 | 36 | 0 | 8 | 0 | 0 |
| 523.2372 | 523.237112 | 2633996 | 27 | 40 | 0 | 8 | 1 | 0 |
| 523.248983 | 523.248998 | 1599244 | 34 | 36 | 0 | 5 | 0 | 0 |
| 523.254814 | 523.254871 | 3206155 | 27 | 40 | 0 | 10 | 0 | 0 |
| 523.269989 | 523.270127 | 4781067 | 31 | 40 | 0 | 7 | 0 | 0 |
| 523.291312 | 523.291257 | 2440714 | 28 | 44 | 0 | 9 | 0 | 0 |
| 523.306758 | 523.306513 | 1753610 | 32 | 44 | 0 | 6 | 0 | 0 |
| 525.082104 | 525.082068 | 1474516 | 21 | 22 | 2 | 12 | 1 | 0 |
| 525.103597 | 525.10385 | 1614547 | 26 | 22 | 0 | 12 | 0 | 0 |
| 525.118837 | 525.119106 | 1763795 | 30 | 22 | 0 | 9 | 0 | 0 |
| 525.139976 | 525.140235 | 2689746 | 27 | 26 | 0 | 11 | 0 | 0 |
| 525.143783 | 525.143606 | 2319570 | 24 | 30 | 0 | 11 | 1 | 0 |
| 525.155465 | 525.155491 | 1720914 | 31 | 26 | 0 | 8 | 0 | 0 |
| 525.176569 | 525.176621 | 4337617 | 28 | 30 | 0 | 10 | 0 | 0 |
| 525.180023 | 525.179992 | 4802513 | 25 | 34 | 0 | 10 | 1 | 0 |
| 525.191854 | 525.191877 | 2200529 | 32 | 30 | 0 | 7 | 0 | 0 |
| 525.213013 | 525.213006 | 6732240 | 29 | 34 | 0 | 9 | 0 | 0 |
| 525.216345 | 525.216377 | 3948496 | 26 | 38 | 0 | 9 | 1 | 0 |
| 525.228236 | 525.228262 | 2159055 | 33 | 34 | 0 | 6 | 0 | 0 |
| 525.23411 | 525.234136 | 1584079 | 26 | 38 | 0 | 11 | 0 | 0 |
| 525.24937 | 525.249392 | 6949327 | 30 | 38 | 0 | 8 | 0 | 0 |
| 525.270331 | 525.270521 | 1692878 | 27 | 42 | 0 | 10 | 0 | 0 |
| 525.28571 | 525.285777 | 3794638 | 31 | 42 | 0 | 7 | 0 | 0 |
| 525.322069 | 525.322163 | 1739212 | 32 | 46 | 0 | 6 | 0 | 0 |
| 527.098508 | 527.09837 | 1630098 | 29 | 20 | 0 | 10 | 0 | 0 |
| 527.119235 | 527.1195 | 1389585 | 26 | 24 | 0 | 12 | 0 | 0 |
| 527.122874 | 527.122871 | 1411857 | 23 | 28 | 0 | 12 | 1 | 0 |
| 527.134728 | 527.134756 | 2060689 | 30 | 24 | 0 | 9 | 0 | 0 |
| 527.156051 | 527.155885 | 2960272 | 27 | 28 | 0 | 11 | 0 | 0 |
| 527.159271 | 527.159256 | 3540880 | 24 | 32 | 0 | 11 | 1 | 0 |
| 527.171123 | 527.171141 | 2056848 | 31 | 28 | 0 | 8 | 0 | 0 |
| 527.192282 | 527.192271 | 5084047 | 28 | 32 | 0 | 10 | 0 | 0 |
| 527.195591 | 527.195642 | 4891023 | 25 | 36 | 0 | 10 | 1 | 0 |
| 527.207273 | 527.207527 | 2396174 | 32 | 32 | 0 | 7 | 0 | 0 |
| 527.228584 | 527.228656 | 8029582 | 29 | 36 | 0 | 9 | 0 | 0 |
| 527.232043 | 527.232027 | 2815374 | 26 | 40 | 0 | 9 | 1 | 0 |
| 527.243925 | 527.243912 | 2353037 | 33 | 36 | 0 | 6 | 0 | 0 |
| 527.26503 | 527.265042 | 7786893 | 30 | 40 | 0 | 8 | 0 | 0 |
| 527.280192 | 527.280298 | 1610124 | 34 | 40 | 0 | 5 | 0 | 0 |
| 527.301442 | 527.301427 | 3254667 | 31 | 44 | 0 | 7 | 0 | 0 |
| 527.337736 | 527.337813 | 1403530 | 32 | 48 | 0 | 6 | 0 | 0 |
| 529.077783 | 529.077635 | 1586509 | 28 | 18 | 0 | 11 | 0 | 0 |
| 529.113892 | 529.11402 | 1853515 | 29 | 22 | 0 | 10 | 0 | 0 |
| 529.134899 | 529.13515 | 1575499 | 26 | 26 | 0 | 12 | 0 | 0 |
| 529.138549 | 529.138521 | 1467466 | 23 | 30 | 0 | 12 | 1 | 0 |
| 529.150208 | 529.150406 | 2588490 | 30 | 26 | 0 | 9 | 0 | 0 |
| 529.171586 | 529.171535 | 3615561 | 27 | 30 | 0 | 11 | 0 | 0 |
| 529.174935 | 529.174906 | 3488585 | 24 | 34 | 0 | 11 | 1 | 0 |
| 529.186176 | 529.18614 | 2308425 | 23 | 34 | 2 | 10 | 1 | 0 |
| 529.207854 | 529.207921 | 7230792 | 28 | 34 | 0 | 10 | 0 | 0 |
| 529.211317 | 529.211292 | 3428680 | 25 | 38 | 0 | 10 | 1 | 0 |
| 529.223081 | 529.223177 | 2982983 | 32 | 34 | 0 | 7 | 0 | 0 |
| 529.244258 | 529.244306 | 9674055 | 29 | 38 | 0 | 9 | 0 | 0 |
| 529.259346 | 529.259562 | 2262854 | 33 | 38 | 0 | 6 | 0 | 0 |
| 529.28055 | 529.280692 | 8158533 | 30 | 42 | 0 | 8 | 0 | 0 |
| 529.295929 | 529.295948 | 1635397 | 34 | 42 | 0 | 5 | 0 | 0 |
| 529.304687 | 529.304812 | 1507908 | 26 | 47 | 2 | 7 | 0 | 1 |
| 529.316985 | 529.317077 | 2659652 | 31 | 46 | 0 | 7 | 0 | 0 |
| 531.092728 | 531.092633 | 1828610 | 20 | 24 | 2 | 13 | 1 | 0 |
| 531.129559 | 531.129671 | 2817792 | 29 | 24 | 0 | 10 | 0 | 0 |
| 531.150397 | 531.1508 | 1604224 | 26 | 28 | 0 | 12 | 0 | 0 |
| 531.154356 | 531.154171 | 1611775 | 23 | 32 | 0 | 12 | 1 | 0 |
| 531.166104 | 531.166056 | 2838783 | 30 | 28 | 0 | 9 | 0 | 0 |
| 531.169345 | 531.169427 | 1874943 | 27 | 32 | 0 | 9 | 1 | 0 |
| 531.187076 | 531.187185 | 4567806 | 27 | 32 | 0 | 11 | 0 | 0 |
| 531.190566 | 531.190556 | 3096318 | 24 | 36 | 0 | 11 | 1 | 0 |
| 531.202351 | 531.202442 | 3433726 | 31 | 32 | 0 | 8 | 0 | 0 |
| 531.205828 | 531.205812 | 1675518 | 28 | 36 | 0 | 8 | 1 | 0 |
| 531.223565 | 531.223571 | 8258813 | 28 | 36 | 0 | 10 | 0 | 0 |
| 531.238818 | 531.238827 | 3250428 | 32 | 36 | 0 | 7 | 0 | 0 |
| 531.259988 | 531.259956 | 9681147 | 29 | 40 | 0 | 9 | 0 | 0 |
| 531.275044 | 531.275213 | 2906875 | 33 | 40 | 0 | 6 | 0 | 0 |
| 531.296307 | 531.296342 | 8106234 | 30 | 44 | 0 | 8 | 0 | 0 |
| 531.332362 | 531.332727 | 1862265 | 31 | 48 | 0 | 7 | 0 | 0 |
| 533.108589 | 533.108935 | 1867443 | 28 | 22 | 0 | 11 | 0 | 0 |
| 533.145367 | 533.145321 | 2628274 | 29 | 26 | 0 | 10 | 0 | 0 |
| 533.148849 | 533.148691 | 1957042 | 26 | 30 | 0 | 10 | 1 | 0 |
| 533.166455 | 533.16645 | 2022833 | 26 | 30 | 0 | 12 | 0 | 0 |
| 533.18158 | 533.181706 | 2694832 | 30 | 30 | 0 | 9 | 0 | 0 |
| 533.184529 | 533.184697 | 2185648 | 29 | 31 | 2 | 6 | 0 | 1 |
| 533.202852 | 533.202835 | 5164208 | 27 | 34 | 0 | 11 | 0 | 0 |
| 533.218159 | 533.218092 | 3651759 | 31 | 34 | 0 | 8 | 0 | 0 |
| 533.22152 | 533.221462 | 2317999 | 28 | 38 | 0 | 8 | 1 | 0 |
| 533.239126 | 533.239221 | 7955118 | 28 | 38 | 0 | 10 | 0 | 0 |
| 533.254254 | 533.254477 | 3368621 | 32 | 38 | 0 | 7 | 0 | 0 |
| 533.275532 | 533.275606 | 7766189 | 29 | 42 | 0 | 9 | 0 | 0 |
| 533.290663 | 533.290863 | 2431660 | 33 | 42 | 0 | 6 | 0 | 0 |
| 533.311929 | 533.311992 | 6557867 | 30 | 46 | 0 | 8 | 0 | 0 |
| 535.124513 | 535.124585 | 1994593 | 28 | 24 | 0 | 11 | 0 | 0 |
| 535.160942 | 535.160971 | 2990688 | 29 | 28 | 0 | 10 | 0 | 0 |
| 535.164497 | 535.164341 | 2578015 | 26 | 32 | 0 | 10 | 1 | 0 |
| 535.181986 | 535.1821 | 2618975 | 26 | 32 | 0 | 12 | 0 | 0 |
| 535.197428 | 535.197356 | 4183134 | 30 | 32 | 0 | 9 | 0 | 0 |
| 535.200755 | 535.200727 | 3860062 | 27 | 36 | 0 | 9 | 1 | 0 |
| 535.218539 | 535.218486 | 5006429 | 27 | 36 | 0 | 11 | 0 | 0 |
| 535.233598 | 535.233742 | 3961181 | 31 | 36 | 0 | 8 | 0 | 0 |
| 535.237177 | 535.237112 | 3515484 | 28 | 40 | 0 | 8 | 1 | 0 |
| 535.25473 | 535.254871 | 5664860 | 28 | 40 | 0 | 10 | 0 | 0 |
| 535.270135 | 535.270127 | 2935899 | 32 | 40 | 0 | 7 | 0 | 0 |
| 535.291192 | 535.291257 | 4191322 | 29 | 44 | 0 | 9 | 0 | 0 |
| 535.306377 | 535.306513 | 2007129 | 33 | 44 | 0 | 6 | 0 | 0 |
| 535.327597 | 535.327642 | 2076633 | 30 | 48 | 0 | 8 | 0 | 0 |
| 537.103995 | 537.10385 | 1389582 | 27 | 22 | 0 | 12 | 0 | 0 |
| 537.119209 | 537.119106 | 1721101 | 31 | 22 | 0 | 9 | 0 | 0 |
| 537.140311 | 537.140235 | 2527500 | 28 | 26 | 0 | 11 | 0 | 0 |
| 537.143536 | 537.143606 | 1794828 | 25 | 30 | 0 | 11 | 1 | 0 |
| 537.176627 | 537.176621 | 3349514 | 29 | 30 | 0 | 10 | 0 | 0 |
| 537.179923 | 537.179992 | 4278794 | 26 | 34 | 0 | 10 | 1 | 0 |
| 537.197746 | 537.19775 | 1840394 | 26 | 34 | 0 | 12 | 0 | 0 |
| 537.212689 | 537.213006 | 4438025 | 30 | 34 | 0 | 9 | 0 | 0 |
| 537.216284 | 537.216377 | 4357129 | 27 | 38 | 0 | 9 | 1 | 0 |
| 537.228346 | 537.228262 | 1400072 | 34 | 34 | 0 | 6 | 0 | 0 |
| 537.234226 | 537.234136 | 3298312 | 27 | 38 | 0 | 11 | 0 | 0 |
| 537.249294 | 537.249392 | 4503047 | 31 | 38 | 0 | 8 | 0 | 0 |
| 537.252624 | 537.252763 | 2158343 | 28 | 42 | 0 | 8 | 1 | 0 |
| 537.270287 | 537.270521 | 3304454 | 28 | 42 | 0 | 10 | 0 | 0 |
| 537.2858 | 537.285777 | 3098118 | 32 | 42 | 0 | 7 | 0 | 0 |
| 537.307098 | 537.306907 | 1678341 | 29 | 46 | 0 | 9 | 0 | 0 |
| 537.321912 | 537.322163 | 1684484 | 33 | 46 | 0 | 6 | 0 | 0 |
| 539.098539 | 539.09837 | 1396919 | 30 | 20 | 0 | 10 | 0 | 0 |
| 539.134674 | 539.134756 | 1649845 | 31 | 24 | 0 | 9 | 0 | 0 |
| 539.155836 | 539.155885 | 2568628 | 28 | 28 | 0 | 11 | 0 | 0 |
| 539.159132 | 539.159256 | 3193780 | 25 | 32 | 0 | 11 | 1 | 0 |
| 539.171295 | 539.171141 | 1529012 | 32 | 28 | 0 | 8 | 0 | 0 |
| 539.192104 | 539.192271 | 4281267 | 29 | 32 | 0 | 10 | 0 | 0 |
| 539.19566 | 539.195642 | 4272051 | 26 | 36 | 0 | 10 | 1 | 0 |
| 539.20764 | 539.207527 | 1672626 | 33 | 32 | 0 | 7 | 0 | 0 |
| 539.213299 | 539.2134 | 1436338 | 26 | 36 | 0 | 12 | 0 | 0 |
| 539.228703 | 539.228656 | 5027761 | 30 | 36 | 0 | 9 | 0 | 0 |
| 539.23195 | 539.232027 | 4026033 | 27 | 40 | 0 | 9 | 1 | 0 |
| 539.249845 | 539.249786 | 1510064 | 27 | 40 | 0 | 11 | 0 | 0 |
| 539.264921 | 539.265042 | 4524976 | 31 | 40 | 0 | 8 | 0 | 0 |
| 539.268432 | 539.268413 | 1772207 | 28 | 44 | 0 | 8 | 1 | 0 |
| 539.280233 | 539.280298 | 1559215 | 35 | 40 | 0 | 5 | 0 | 0 |
| 539.301178 | 539.301427 | 2740142 | 32 | 44 | 0 | 7 | 0 | 0 |
| 541.114027 | 541.11402 | 1746013 | 30 | 22 | 0 | 10 | 0 | 0 |
| 541.134768 | 541.13515 | 1925212 | 27 | 26 | 0 | 12 | 0 | 0 |
| 541.138298 | 541.138521 | 1679452 | 24 | 30 | 0 | 12 | 1 | 0 |
| 541.150443 | 541.150406 | 1728348 | 31 | 26 | 0 | 9 | 0 | 0 |
| 541.171584 | 541.171535 | 2735707 | 28 | 30 | 0 | 11 | 0 | 0 |
| 541.175044 | 541.174906 | 3844955 | 25 | 34 | 0 | 11 | 1 | 0 |
| 541.186648 | 541.186791 | 2036570 | 32 | 30 | 0 | 8 | 0 | 0 |
| 541.207817 | 541.207921 | 5193049 | 29 | 34 | 0 | 10 | 0 | 0 |
| 541.211235 | 541.211292 | 3593049 | 26 | 38 | 0 | 10 | 1 | 0 |
| 541.22312 | 541.223177 | 2143320 | 33 | 34 | 0 | 7 | 0 | 0 |
| 541.244184 | 541.244306 | 6357336 | 30 | 38 | 0 | 9 | 0 | 0 |
| 541.247842 | 541.247677 | 2372439 | 27 | 42 | 0 | 9 | 1 | 0 |
| 541.2596 | 541.259562 | 1531991 | 34 | 38 | 0 | 6 | 0 | 0 |
| 541.280547 | 541.280692 | 4170582 | 31 | 42 | 0 | 8 | 0 | 0 |
| 541.316992 | 541.317077 | 2145364 | 32 | 46 | 0 | 7 | 0 | 0 |
| 543.129246 | 543.129671 | 2025730 | 30 | 24 | 0 | 10 | 0 | 0 |
| 543.150512 | 543.1508 | 2358273 | 27 | 28 | 0 | 12 | 0 | 0 |
| 543.154074 | 543.154171 | 2091265 | 24 | 32 | 0 | 12 | 1 | 0 |
| 543.165644 | 543.166056 | 2046848 | 31 | 28 | 0 | 9 | 0 | 0 |
| 543.187124 | 543.187185 | 3891455 | 28 | 32 | 0 | 11 | 0 | 0 |
| 543.190605 | 543.190556 | 3993343 | 25 | 36 | 0 | 11 | 1 | 0 |
| 543.202569 | 543.202442 | 2582783 | 32 | 32 | 0 | 8 | 0 | 0 |
| 543.22335 | 543.223571 | 6263550 | 29 | 36 | 0 | 10 | 0 | 0 |
| 543.227099 | 543.226942 | 2389246 | 26 | 40 | 0 | 10 | 1 | 0 |
| 543.238737 | 543.238827 | 2154237 | 33 | 36 | 0 | 7 | 0 | 0 |
| 543.259946 | 543.259956 | 8046332 | 30 | 40 | 0 | 9 | 0 | 0 |
| 543.275223 | 543.275213 | 2405115 | 34 | 40 | 0 | 6 | 0 | 0 |
| 543.296379 | 543.296342 | 3807994 | 31 | 44 | 0 | 8 | 0 | 0 |
| 543.332762 | 543.332727 | 1802489 | 32 | 48 | 0 | 7 | 0 | 0 |
| 545.108723 | 545.108935 | 1637031 | 29 | 22 | 0 | 11 | 0 | 0 |
| 545.145175 | 545.145321 | 1786629 | 30 | 26 | 0 | 10 | 0 | 0 |
| 545.166429 | 545.16645 | 2023588 | 27 | 30 | 0 | 12 | 0 | 0 |
| 545.169825 | 545.169821 | 1914532 | 24 | 34 | 0 | 12 | 1 | 0 |
| 545.181534 | 545.181706 | 2769060 | 31 | 30 | 0 | 9 | 0 | 0 |
| 545.202698 | 545.202835 | 4542627 | 28 | 34 | 0 | 11 | 0 | 0 |
| 545.20615 | 545.206206 | 2652835 | 25 | 38 | 0 | 11 | 1 | 0 |
| 545.218054 | 545.218092 | 2616482 | 32 | 34 | 0 | 8 | 0 | 0 |
| 545.239134 | 545.239221 | 7440033 | 29 | 38 | 0 | 10 | 0 | 0 |
| 545.242902 | 545.242592 | 1677473 | 26 | 42 | 0 | 10 | 1 | 0 |
| 545.254188 | 545.254477 | 2692000 | 33 | 38 | 0 | 7 | 0 | 0 |
| 545.275534 | 545.275606 | 7878303 | 30 | 42 | 0 | 9 | 0 | 0 |
| 545.290754 | 545.290863 | 2451103 | 34 | 42 | 0 | 6 | 0 | 0 |
| 545.311641 | 545.311992 | 3285150 | 31 | 46 | 0 | 8 | 0 | 0 |
| 547.124098 | 547.124585 | 1745994 | 29 | 24 | 0 | 11 | 0 | 0 |
| 547.16009 | 547.160319 | 1888328 | 22 | 32 | 2 | 12 | 1 | 0 |
| 547.181632 | 547.1821 | 2427847 | 27 | 32 | 0 | 12 | 0 | 0 |
| 547.185284 | 547.185471 | 1735751 | 24 | 36 | 0 | 12 | 1 | 0 |
| 547.19712 | 547.197356 | 2692038 | 31 | 32 | 0 | 9 | 0 | 0 |
| 547.200805 | 547.200727 | 2339398 | 28 | 36 | 0 | 9 | 1 | 0 |
| 547.218402 | 547.218486 | 5255749 | 28 | 36 | 0 | 11 | 0 | 0 |
| 547.233616 | 547.233742 | 3227205 | 32 | 36 | 0 | 8 | 0 | 0 |
| 547.236966 | 547.237112 | 2002500 | 29 | 40 | 0 | 8 | 1 | 0 |
| 547.254811 | 547.254871 | 7424580 | 29 | 40 | 0 | 10 | 0 | 0 |
| 547.27028 | 547.270127 | 2572355 | 33 | 40 | 0 | 7 | 0 | 0 |
| 547.291087 | 547.291257 | 6227778 | 30 | 44 | 0 | 9 | 0 | 0 |
| 547.306139 | 547.306513 | 1426753 | 34 | 44 | 0 | 6 | 0 | 0 |
| 547.327695 | 547.327642 | 1957440 | 31 | 48 | 0 | 8 | 0 | 0 |
| 549.140298 | 549.140235 | 1994732 | 29 | 26 | 0 | 11 | 0 | 0 |
| 549.176253 | 549.176621 | 2438890 | 30 | 30 | 0 | 10 | 0 | 0 |
| 549.179806 | 549.179992 | 3128298 | 27 | 34 | 0 | 10 | 1 | 0 |
| 549.197603 | 549.19775 | 2732521 | 27 | 34 | 0 | 12 | 0 | 0 |
| 549.212917 | 549.213006 | 3065320 | 31 | 34 | 0 | 9 | 0 | 0 |
| 549.216365 | 549.216377 | 3598312 | 28 | 38 | 0 | 9 | 1 | 0 |
| 549.234034 | 549.234136 | 4606440 | 28 | 38 | 0 | 11 | 0 | 0 |
| 549.249289 | 549.249392 | 3282919 | 32 | 38 | 0 | 8 | 0 | 0 |
| 549.252692 | 549.252763 | 2764775 | 29 | 42 | 0 | 8 | 1 | 0 |
| 549.270492 | 549.270521 | 4963814 | 29 | 42 | 0 | 10 | 0 | 0 |
| 549.285666 | 549.285777 | 2014181 | 33 | 42 | 0 | 7 | 0 | 0 |
| 549.306694 | 549.306907 | 3359716 | 30 | 46 | 0 | 9 | 0 | 0 |
| 549.321753 | 549.322163 | 1553891 | 34 | 46 | 0 | 6 | 0 | 0 |
| 551.118793 | 551.118848 | 1513104 | 20 | 28 | 2 | 14 | 1 | 0 |
| 551.15552 | 551.155885 | 2243470 | 29 | 28 | 0 | 11 | 0 | 0 |
| 551.159288 | 551.159256 | 2508686 | 26 | 32 | 0 | 11 | 1 | 0 |
| 551.170908 | 551.171141 | 1439885 | 33 | 28 | 0 | 8 | 0 | 0 |
| 551.192168 | 551.192271 | 2869388 | 30 | 32 | 0 | 10 | 0 | 0 |
| 551.195609 | 551.195642 | 4927884 | 27 | 36 | 0 | 10 | 1 | 0 |
| 551.213373 | 551.2134 | 2921355 | 27 | 36 | 0 | 12 | 0 | 0 |
| 551.228558 | 551.228656 | 3671947 | 31 | 36 | 0 | 9 | 0 | 0 |
| 551.231986 | 551.232027 | 4436619 | 28 | 40 | 0 | 9 | 1 | 0 |
| 551.243752 | 551.243912 | 1599882 | 35 | 36 | 0 | 6 | 0 | 0 |
| 551.249762 | 551.249786 | 3024266 | 28 | 40 | 0 | 11 | 0 | 0 |
| 551.264683 | 551.265042 | 3297929 | 32 | 40 | 0 | 8 | 0 | 0 |
| 551.268327 | 551.268413 | 2493833 | 29 | 44 | 0 | 8 | 1 | 0 |
| 551.286117 | 551.286171 | 2427272 | 29 | 44 | 0 | 10 | 0 | 0 |
| 551.301387 | 551.301427 | 2202503 | 33 | 44 | 0 | 7 | 0 | 0 |
| 551.32254 | 551.322557 | 1845126 | 30 | 48 | 0 | 9 | 0 | 0 |
| 553.11395 | 553.11402 | 1689907 | 31 | 22 | 0 | 10 | 0 | 0 |
| 553.135268 | 553.13515 | 1736754 | 28 | 26 | 0 | 12 | 0 | 0 |
| 553.138179 | 553.138521 | 1539378 | 25 | 30 | 0 | 12 | 1 | 0 |
| 553.149661 | 553.149754 | 1484338 | 24 | 30 | 2 | 11 | 1 | 0 |
| 553.171381 | 553.171535 | 2587697 | 29 | 30 | 0 | 11 | 0 | 0 |
| 553.174716 | 553.174906 | 3236913 | 26 | 34 | 0 | 11 | 1 | 0 |
| 553.186816 | 553.186791 | 1537328 | 33 | 30 | 0 | 8 | 0 | 0 |
| 553.207525 | 553.207921 | 3206959 | 30 | 34 | 0 | 10 | 0 | 0 |
| 553.211071 | 553.211292 | 4643119 | 27 | 38 | 0 | 10 | 1 | 0 |
| 553.222107 | 553.222525 | 1640750 | 26 | 38 | 2 | 9 | 1 | 0 |
| 553.244193 | 553.244306 | 4146477 | 31 | 38 | 0 | 9 | 0 | 0 |
| 553.247694 | 553.247677 | 3219757 | 28 | 42 | 0 | 9 | 1 | 0 |
| 553.26493 | 553.265436 | 1552684 | 28 | 42 | 0 | 11 | 0 | 0 |
| 553.280509 | 553.280692 | 3470636 | 32 | 42 | 0 | 8 | 0 | 0 |
| 553.317138 | 553.317077 | 1788714 | 33 | 46 | 0 | 7 | 0 | 0 |
| 555.129715 | 555.129671 | 1445335 | 31 | 24 | 0 | 10 | 0 | 0 |
| 555.150742 | 555.1508 | 1920470 | 28 | 28 | 0 | 12 | 0 | 0 |
| 555.154135 | 555.154171 | 2096854 | 25 | 32 | 0 | 12 | 1 | 0 |
| 555.165964 | 555.166056 | 1938645 | 32 | 28 | 0 | 9 | 0 | 0 |
| 555.187108 | 555.187185 | 3032788 | 29 | 32 | 0 | 11 | 0 | 0 |
| 555.190519 | 555.190556 | 4187348 | 26 | 36 | 0 | 11 | 1 | 0 |
| 555.202555 | 555.202442 | 1411411 | 33 | 32 | 0 | 8 | 0 | 0 |
| 555.223516 | 555.223571 | 5094611 | 30 | 36 | 0 | 10 | 0 | 0 |
| 555.22689 | 555.226942 | 5228755 | 27 | 40 | 0 | 10 | 1 | 0 |
| 555.238534 | 555.238827 | 1772498 | 34 | 36 | 0 | 7 | 0 | 0 |
| 555.259797 | 555.259956 | 4514513 | 31 | 40 | 0 | 9 | 0 | 0 |
| 555.263251 | 555.263327 | 2124497 | 28 | 44 | 0 | 9 | 1 | 0 |
| 555.296146 | 555.296342 | 2846927 | 32 | 44 | 0 | 8 | 0 | 0 |
| 557.145172 | 557.145321 | 2547324 | 31 | 26 | 0 | 10 | 0 | 0 |
| 557.166165 | 557.16645 | 2227835 | 28 | 30 | 0 | 12 | 0 | 0 |
| 557.169559 | 557.169821 | 2156411 | 25 | 34 | 0 | 12 | 1 | 0 |
| 557.181726 | 557.181706 | 2045562 | 32 | 30 | 0 | 9 | 0 | 0 |
| 557.202834 | 557.202835 | 3571321 | 29 | 34 | 0 | 11 | 0 | 0 |
| 557.206049 | 557.206206 | 3087481 | 26 | 38 | 0 | 11 | 1 | 0 |
| 557.218207 | 557.218092 | 1491064 | 33 | 34 | 0 | 8 | 0 | 0 |
| 557.239036 | 557.239221 | 5537912 | 30 | 38 | 0 | 10 | 0 | 0 |
| 557.241928 | 557.242212 | 2277751 | 29 | 39 | 2 | 7 | 0 | 1 |
| 557.254285 | 557.254477 | 2007159 | 34 | 38 | 0 | 7 | 0 | 0 |
| 557.275596 | 557.275606 | 3363446 | 31 | 42 | 0 | 9 | 0 | 0 |
| 557.312042 | 557.311992 | 2616436 | 32 | 46 | 0 | 8 | 0 | 0 |
| 559.124777 | 559.124585 | 1616932 | 30 | 24 | 0 | 11 | 0 | 0 |
| 559.160811 | 559.160971 | 1898530 | 31 | 28 | 0 | 10 | 0 | 0 |
| 559.181834 | 559.1821 | 2404897 | 28 | 32 | 0 | 12 | 0 | 0 |
| 559.185525 | 559.185471 | 2090017 | 25 | 36 | 0 | 12 | 1 | 0 |
| 559.197255 | 559.197356 | 2603041 | 32 | 32 | 0 | 9 | 0 | 0 |
| 559.2184 | 559.218486 | 4944928 | 29 | 36 | 0 | 11 | 0 | 0 |
| 559.221904 | 559.221856 | 2336288 | 26 | 40 | 0 | 11 | 1 | 0 |
| 559.233782 | 559.233742 | 1869343 | 33 | 36 | 0 | 8 | 0 | 0 |
| 559.254705 | 559.254871 | 6532126 | 30 | 40 | 0 | 10 | 0 | 0 |
| 559.269389 | 559.269475 | 1752349 | 26 | 44 | 2 | 9 | 1 | 0 |
| 559.291071 | 559.291257 | 3613212 | 31 | 44 | 0 | 9 | 0 | 0 |
| 559.327973 | 559.327642 | 1511451 | 32 | 48 | 0 | 8 | 0 | 0 |
| 561.103112 | 561.103198 | 1445582 | 21 | 26 | 2 | 14 | 1 | 0 |
| 561.140341 | 561.140235 | 1678796 | 30 | 26 | 0 | 11 | 0 | 0 |
| 561.176659 | 561.176621 | 2051530 | 31 | 30 | 0 | 10 | 0 | 0 |
| 561.180218 | 561.179992 | 1982410 | 28 | 34 | 0 | 10 | 1 | 0 |
| 561.197748 | 561.19775 | 2966474 | 28 | 34 | 0 | 12 | 0 | 0 |
| 561.212993 | 561.213006 | 2444745 | 32 | 34 | 0 | 9 | 0 | 0 |
| 561.216234 | 561.216377 | 2386377 | 29 | 38 | 0 | 9 | 1 | 0 |
| 561.234009 | 561.234136 | 5384136 | 29 | 38 | 0 | 11 | 0 | 0 |
| 561.249319 | 561.249392 | 2300103 | 33 | 38 | 0 | 8 | 0 | 0 |
| 561.27042 | 561.270521 | 6264775 | 30 | 42 | 0 | 10 | 0 | 0 |
| 561.285699 | 561.285777 | 2047430 | 34 | 42 | 0 | 7 | 0 | 0 |
| 561.306504 | 561.306907 | 2821317 | 31 | 46 | 0 | 9 | 0 | 0 |
| 563.119442 | 563.1195 | 1597304 | 29 | 24 | 0 | 12 | 0 | 0 |
| 563.155939 | 563.155885 | 1702518 | 30 | 28 | 0 | 11 | 0 | 0 |
| 563.159142 | 563.159256 | 1745782 | 27 | 32 | 0 | 11 | 1 | 0 |
| 563.17732 | 563.177015 | 1420661 | 27 | 32 | 0 | 13 | 0 | 0 |
| 563.192098 | 563.192271 | 2419573 | 31 | 32 | 0 | 10 | 0 | 0 |
| 563.195494 | 563.195642 | 3114868 | 28 | 36 | 0 | 10 | 1 | 0 |
| 563.213487 | 563.2134 | 3199860 | 28 | 36 | 0 | 12 | 0 | 0 |
| 563.228414 | 563.228656 | 3124083 | 32 | 36 | 0 | 9 | 0 | 0 |
| 563.231878 | 563.232027 | 3332467 | 29 | 40 | 0 | 9 | 1 | 0 |
| 563.249686 | 563.249786 | 4875122 | 29 | 40 | 0 | 11 | 0 | 0 |
| 563.264973 | 563.265042 | 2705522 | 33 | 40 | 0 | 8 | 0 | 0 |
| 563.268476 | 563.268413 | 2126705 | 30 | 44 | 0 | 8 | 1 | 0 |
| 563.286015 | 563.286171 | 4762993 | 30 | 44 | 0 | 10 | 0 | 0 |
| 563.301216 | 563.301427 | 2042736 | 34 | 44 | 0 | 7 | 0 | 0 |
| 563.322478 | 563.322557 | 1788783 | 31 | 48 | 0 | 9 | 0 | 0 |
| 565.135168 | 565.13515 | 1639716 | 29 | 26 | 0 | 12 | 0 | 0 |
| 565.170896 | 565.170883 | 2310434 | 22 | 34 | 2 | 13 | 1 | 0 |
| 565.174769 | 565.174906 | 2720290 | 27 | 34 | 0 | 11 | 1 | 0 |
| 565.20779 | 565.207921 | 3006241 | 31 | 34 | 0 | 10 | 0 | 0 |
| 565.211268 | 565.211292 | 4840225 | 28 | 38 | 0 | 10 | 1 | 0 |
| 565.229008 | 565.22905 | 3047200 | 28 | 38 | 0 | 12 | 0 | 0 |
| 565.243911 | 565.244306 | 2914335 | 32 | 38 | 0 | 9 | 0 | 0 |
| 565.247753 | 565.247677 | 3839775 | 29 | 42 | 0 | 9 | 1 | 0 |
| 565.265348 | 565.265436 | 2834206 | 29 | 42 | 0 | 11 | 0 | 0 |
| 565.280722 | 565.280692 | 2443038 | 33 | 42 | 0 | 8 | 0 | 0 |
| 565.283948 | 565.284063 | 1883422 | 30 | 46 | 0 | 8 | 1 | 0 |
| 565.301726 | 565.301821 | 2253341 | 30 | 46 | 0 | 10 | 0 | 0 |
| 565.317033 | 565.317077 | 1665820 | 34 | 46 | 0 | 7 | 0 | 0 |
| 567.129863 | 567.129671 | 1537747 | 32 | 24 | 0 | 10 | 0 | 0 |
| 567.153408 | 567.153519 | 1910482 | 18 | 36 | 2 | 14 | 2 | 0 |
| 567.166066 | 567.166056 | 1868241 | 33 | 28 | 0 | 9 | 0 | 0 |
| 567.187027 | 567.187185 | 2618064 | 30 | 32 | 0 | 11 | 0 | 0 |
| 567.190595 | 567.190556 | 4117200 | 27 | 36 | 0 | 11 | 1 | 0 |
| 567.223555 | 567.223571 | 2751951 | 31 | 36 | 0 | 10 | 0 | 0 |
| 567.226871 | 567.226942 | 5452495 | 28 | 40 | 0 | 10 | 1 | 0 |
| 567.244699 | 567.2447 | 1743054 | 28 | 40 | 0 | 12 | 0 | 0 |
| 567.25995 | 567.259956 | 3284685 | 32 | 40 | 0 | 9 | 0 | 0 |
| 567.263379 | 567.263327 | 3659981 | 29 | 44 | 0 | 9 | 1 | 0 |
| 567.296152 | 567.296342 | 2738892 | 33 | 44 | 0 | 8 | 0 | 0 |
| 567.332686 | 567.332727 | 1497546 | 34 | 48 | 0 | 7 | 0 | 0 |
| 569.165304 | 569.165798 | 2039938 | 21 | 34 | 2 | 14 | 1 | 0 |
| 569.169654 | 569.169821 | 2528130 | 26 | 34 | 0 | 12 | 1 | 0 |
| 569.180989 | 569.181054 | 1448769 | 25 | 34 | 2 | 11 | 1 | 0 |
| 569.202911 | 569.202835 | 2869889 | 30 | 34 | 0 | 11 | 0 | 0 |
| 569.206098 | 569.206206 | 4145281 | 27 | 38 | 0 | 11 | 1 | 0 |
| 569.217841 | 569.218092 | 1491584 | 34 | 34 | 0 | 8 | 0 | 0 |
| 569.239217 | 569.239221 | 3569791 | 31 | 38 | 0 | 10 | 0 | 0 |
| 569.242511 | 569.242592 | 3478655 | 28 | 42 | 0 | 10 | 1 | 0 |
| 569.254659 | 569.254477 | 1623423 | 35 | 38 | 0 | 7 | 0 | 0 |
| 569.275397 | 569.275606 | 3682942 | 32 | 42 | 0 | 9 | 0 | 0 |
| 569.278944 | 569.278977 | 2011262 | 29 | 46 | 0 | 9 | 1 | 0 |
| 569.311682 | 569.311992 | 2303100 | 33 | 46 | 0 | 8 | 0 | 0 |
| 571.124502 | 571.124585 | 1393975 | 31 | 24 | 0 | 11 | 0 | 0 |
| 571.160871 | 571.160971 | 1477685 | 32 | 28 | 0 | 10 | 0 | 0 |
| 571.181981 | 571.1821 | 2453557 | 29 | 32 | 0 | 12 | 0 | 0 |
| 571.18539 | 571.185471 | 2647604 | 26 | 36 | 0 | 12 | 1 | 0 |
| 571.196859 | 571.197356 | 1552692 | 33 | 32 | 0 | 9 | 0 | 0 |
| 571.217732 | 571.217834 | 3059251 | 22 | 40 | 2 | 13 | 1 | 0 |
| 571.221721 | 571.221856 | 3255603 | 27 | 40 | 0 | 11 | 1 | 0 |
| 571.233494 | 571.233742 | 1956403 | 34 | 36 | 0 | 8 | 0 | 0 |
| 571.254744 | 571.254871 | 3197490 | 31 | 40 | 0 | 10 | 0 | 0 |
| 571.270015 | 571.270127 | 1705777 | 35 | 40 | 0 | 7 | 0 | 0 |
| 571.291057 | 571.291257 | 3488817 | 32 | 44 | 0 | 9 | 0 | 0 |
| 573.10392 | 573.10385 | 1531117 | 30 | 22 | 0 | 12 | 0 | 0 |
| 573.139651 | 573.139583 | 1937388 | 23 | 30 | 2 | 13 | 1 | 0 |
| 573.176519 | 573.176621 | 2173418 | 32 | 30 | 0 | 10 | 0 | 0 |
| 573.197209 | 573.19775 | 2210282 | 29 | 34 | 0 | 12 | 0 | 0 |
| 573.200865 | 573.201121 | 1549545 | 26 | 38 | 0 | 12 | 1 | 0 |
| 573.212839 | 573.213006 | 2172905 | 33 | 34 | 0 | 9 | 0 | 0 |
| 573.233896 | 573.234136 | 3986408 | 30 | 38 | 0 | 11 | 0 | 0 |
| 573.249438 | 573.249392 | 1990376 | 34 | 38 | 0 | 8 | 0 | 0 |
| 573.27025 | 573.270521 | 3511527 | 31 | 42 | 0 | 10 | 0 | 0 |
| 573.285677 | 573.285777 | 1629926 | 35 | 42 | 0 | 7 | 0 | 0 |
| 573.306266 | 573.305875 | 2186982 | 26 | 47 | 4 | 8 | 0 | 1 |
| 575.155928 | 575.155885 | 1557667 | 31 | 28 | 0 | 11 | 0 | 0 |
| 575.176829 | 575.177015 | 1745570 | 28 | 32 | 0 | 13 | 0 | 0 |
| 575.1924 | 575.192271 | 1848226 | 32 | 32 | 0 | 10 | 0 | 0 |
| 575.195725 | 575.195642 | 1709985 | 29 | 36 | 0 | 10 | 1 | 0 |
| 575.212785 | 575.212235 | 2874273 | 34 | 32 | 4 | 3 | 1 | 0 |
| 575.228342 | 575.228656 | 2671520 | 33 | 36 | 0 | 9 | 0 | 0 |
| 575.249744 | 575.249786 | 4504992 | 30 | 40 | 0 | 11 | 0 | 0 |
| 575.265055 | 575.265042 | 2252703 | 34 | 40 | 0 | 8 | 0 | 0 |
| 575.286151 | 575.286171 | 3351966 | 31 | 44 | 0 | 10 | 0 | 0 |
| 575.301239 | 575.301427 | 1655710 | 35 | 44 | 0 | 7 | 0 | 0 |
| 575.322482 | 575.322557 | 1417373 | 32 | 48 | 0 | 9 | 0 | 0 |
| 577.1351 | 577.13515 | 1682270 | 30 | 26 | 0 | 12 | 0 | 0 |
| 577.170928 | 577.170883 | 1625565 | 23 | 34 | 2 | 13 | 1 | 0 |
| 577.207755 | 577.207921 | 2031579 | 32 | 34 | 0 | 10 | 0 | 0 |
| 577.211115 | 577.211292 | 3289947 | 29 | 38 | 0 | 10 | 1 | 0 |
| 577.229037 | 577.22905 | 3223387 | 29 | 38 | 0 | 12 | 0 | 0 |
| 577.244035 | 577.244306 | 2158426 | 33 | 38 | 0 | 9 | 0 | 0 |
| 577.247572 | 577.247677 | 2645338 | 30 | 42 | 0 | 9 | 1 | 0 |
| 577.26507 | 577.265436 | 3999834 | 30 | 42 | 0 | 11 | 0 | 0 |
| 577.280554 | 577.280692 | 2277977 | 34 | 42 | 0 | 8 | 0 | 0 |
| 577.301639 | 577.301821 | 2167640 | 31 | 46 | 0 | 10 | 0 | 0 |
| 577.316934 | 577.317077 | 1604184 | 35 | 46 | 0 | 7 | 0 | 0 |
| 579.15387 | 579.154171 | 1553946 | 27 | 32 | 0 | 12 | 1 | 0 |
| 579.187064 | 579.187185 | 2108441 | 31 | 32 | 0 | 11 | 0 | 0 |
| 579.190407 | 579.190556 | 3577625 | 28 | 36 | 0 | 11 | 1 | 0 |
| 579.208367 | 579.208315 | 1468953 | 28 | 36 | 0 | 13 | 0 | 0 |
| 579.223413 | 579.223571 | 2692888 | 32 | 36 | 0 | 10 | 0 | 0 |
| 579.226846 | 579.226942 | 4484376 | 29 | 40 | 0 | 10 | 1 | 0 |
| 579.244423 | 579.2447 | 2167319 | 29 | 40 | 0 | 12 | 0 | 0 |
| 579.259943 | 579.259956 | 2988823 | 33 | 40 | 0 | 9 | 0 | 0 |
| 579.2632 | 579.263327 | 3351319 | 30 | 44 | 0 | 9 | 1 | 0 |
| 579.281015 | 579.281086 | 3361558 | 30 | 44 | 0 | 11 | 0 | 0 |
| 579.296259 | 579.296342 | 1975574 | 34 | 44 | 0 | 8 | 0 | 0 |
| 579.332489 | 579.332727 | 1431316 | 35 | 48 | 0 | 7 | 0 | 0 |
| 581.166342 | 581.16645 | 1535322 | 30 | 30 | 0 | 12 | 0 | 0 |
| 581.169894 | 581.169821 | 2156250 | 27 | 34 | 0 | 12 | 1 | 0 |
| 581.202872 | 581.202835 | 2149977 | 31 | 34 | 0 | 11 | 0 | 0 |
| 581.206103 | 581.206206 | 5091545 | 28 | 38 | 0 | 11 | 1 | 0 |
| 581.217541 | 581.218092 | 1384280 | 35 | 34 | 0 | 8 | 0 | 0 |
| 581.238167 | 581.238569 | 2218839 | 24 | 42 | 2 | 12 | 1 | 0 |
| 581.242506 | 581.242592 | 4690135 | 29 | 42 | 0 | 10 | 1 | 0 |
| 581.260339 | 581.26035 | 1697495 | 29 | 42 | 0 | 12 | 0 | 0 |
| 581.27553 | 581.275606 | 2164182 | 33 | 42 | 0 | 9 | 0 | 0 |
| 581.278754 | 581.278977 | 2817238 | 30 | 46 | 0 | 9 | 1 | 0 |
| 581.296745 | 581.296736 | 1462486 | 30 | 46 | 0 | 11 | 0 | 0 |
| 581.311613 | 581.311992 | 2057685 | 34 | 46 | 0 | 8 | 0 | 0 |
| 583.181769 | 583.1821 | 2055196 | 30 | 32 | 0 | 12 | 0 | 0 |
| 583.185316 | 583.185471 | 2921628 | 27 | 36 | 0 | 12 | 1 | 0 |
| 583.218482 | 583.218486 | 2274715 | 31 | 36 | 0 | 11 | 0 | 0 |
| 583.221646 | 583.221856 | 4551835 | 28 | 40 | 0 | 11 | 1 | 0 |
| 583.233814 | 583.233742 | 1387162 | 35 | 36 | 0 | 8 | 0 | 0 |
| 583.254467 | 583.254871 | 2931866 | 32 | 40 | 0 | 10 | 0 | 0 |
| 583.257937 | 583.258242 | 3890329 | 29 | 44 | 0 | 10 | 1 | 0 |
| 583.291146 | 583.291257 | 2357400 | 33 | 44 | 0 | 9 | 0 | 0 |
| 583.327031 | 583.32699 | 1566871 | 26 | 52 | 2 | 10 | 1 | 0 |
| 585.161428 | 585.161365 | 1678434 | 29 | 30 | 0 | 13 | 0 | 0 |
| 585.164506 | 585.164735 | 1470818 | 26 | 34 | 0 | 13 | 1 | 0 |
| 585.196999 | 585.197098 | 2101729 | 22 | 38 | 2 | 14 | 1 | 0 |
| 585.200975 | 585.201121 | 3354209 | 27 | 38 | 0 | 12 | 1 | 0 |
| 585.233893 | 585.234136 | 3113824 | 31 | 38 | 0 | 11 | 0 | 0 |
| 585.23747 | 585.237506 | 2855520 | 28 | 42 | 0 | 11 | 1 | 0 |
| 585.270259 | 585.270521 | 3431007 | 32 | 42 | 0 | 10 | 0 | 0 |
| 585.273904 | 585.273892 | 1899615 | 29 | 46 | 0 | 10 | 1 | 0 |
| 585.306814 | 585.306907 | 2087006 | 33 | 46 | 0 | 9 | 0 | 0 |
| 587.119509 | 587.1195 | 1378859 | 31 | 24 | 0 | 12 | 0 | 0 |
| 587.180421 | 587.180385 | 1420074 | 26 | 36 | 0 | 13 | 1 | 0 |
| 587.192166 | 587.192271 | 1552169 | 33 | 32 | 0 | 10 | 0 | 0 |
| 587.213252 | 587.2134 | 2290729 | 30 | 36 | 0 | 12 | 0 | 0 |
| 587.216736 | 587.216771 | 2476073 | 27 | 40 | 0 | 12 | 1 | 0 |
| 587.228753 | 587.228656 | 1777064 | 34 | 36 | 0 | 9 | 0 | 0 |
| 587.249303 | 587.249786 | 3108904 | 31 | 40 | 0 | 11 | 0 | 0 |
| 587.264961 | 587.265042 | 1550375 | 35 | 40 | 0 | 8 | 0 | 0 |
| 587.285365 | 587.285519 | 2538023 | 24 | 48 | 2 | 12 | 1 | 0 |
| 587.300818 | 587.300775 | 1393702 | 28 | 48 | 2 | 9 | 1 | 0 |
| 587.32228 | 587.322557 | 1771046 | 33 | 48 | 0 | 9 | 0 | 0 |
| 589.192151 | 589.192665 | 1633397 | 29 | 34 | 0 | 13 | 0 | 0 |
| 589.207361 | 589.207921 | 1572852 | 33 | 34 | 0 | 10 | 0 | 0 |
| 589.211347 | 589.211292 | 1515508 | 30 | 38 | 0 | 10 | 1 | 0 |
| 589.228639 | 589.22905 | 2703348 | 30 | 38 | 0 | 12 | 0 | 0 |
| 589.244081 | 589.244306 | 2166516 | 34 | 38 | 0 | 9 | 0 | 0 |
| 589.265435 | 589.265436 | 2930675 | 31 | 42 | 0 | 11 | 0 | 0 |
| 589.280667 | 589.280692 | 1464051 | 35 | 42 | 0 | 8 | 0 | 0 |
| 589.301706 | 589.301821 | 2162674 | 32 | 46 | 0 | 10 | 0 | 0 |
| 591.114313 | 591.114414 | 1594566 | 30 | 24 | 0 | 13 | 0 | 0 |
| 591.151027 | 591.1508 | 1456325 | 31 | 28 | 0 | 12 | 0 | 0 |
| 591.187052 | 591.187185 | 1872324 | 32 | 32 | 0 | 11 | 0 | 0 |
| 591.19006 | 591.190556 | 2283972 | 29 | 36 | 0 | 11 | 1 | 0 |
| 591.207778 | 591.208315 | 1562691 | 29 | 36 | 0 | 13 | 0 | 0 |
| 591.223136 | 591.223571 | 2129859 | 33 | 36 | 0 | 10 | 0 | 0 |
| 591.226897 | 591.226942 | 2454467 | 30 | 40 | 0 | 10 | 1 | 0 |
| 591.244506 | 591.2447 | 2951618 | 30 | 40 | 0 | 12 | 0 | 0 |
| 591.259534 | 591.259956 | 1991106 | 34 | 40 | 0 | 9 | 0 | 0 |
| 591.281003 | 591.281086 | 2530242 | 31 | 44 | 0 | 11 | 0 | 0 |
| 591.296294 | 591.296342 | 1578945 | 35 | 44 | 0 | 8 | 0 | 0 |
| 593.130008 | 593.130064 | 1428375 | 30 | 26 | 0 | 13 | 0 | 0 |
| 593.151085 | 593.151194 | 1492887 | 27 | 30 | 0 | 15 | 0 | 0 |
| 593.166366 | 593.16645 | 1934742 | 31 | 30 | 0 | 12 | 0 | 0 |
| 593.206033 | 593.206206 | 3537813 | 29 | 38 | 0 | 11 | 1 | 0 |
| 593.224045 | 593.223965 | 1530773 | 29 | 38 | 0 | 13 | 0 | 0 |
| 593.23839 | 593.238569 | 2149013 | 25 | 42 | 2 | 12 | 1 | 0 |
| 593.242372 | 593.242592 | 3649941 | 30 | 42 | 0 | 10 | 1 | 0 |
| 593.260169 | 593.26035 | 2440596 | 30 | 42 | 0 | 12 | 0 | 0 |
| 593.275708 | 593.275606 | 1988500 | 34 | 42 | 0 | 9 | 0 | 0 |
| 593.31185 | 593.311992 | 1667219 | 35 | 46 | 0 | 8 | 0 | 0 |
| 594.242167 | 594.241681 | 1348735 | 26 | 37 | 5 | 11 | 0 | 0 |
| 595.149856 | 595.150423 | 1345900 | 28 | 28 | 4 | 9 | 1 | 0 |
| 595.185414 | 595.185471 | 2817899 | 28 | 36 | 0 | 12 | 1 | 0 |
| 595.221699 | 595.221856 | 4271979 | 29 | 40 | 0 | 11 | 1 | 0 |
| 595.25461 | 595.254871 | 2333802 | 33 | 40 | 0 | 10 | 0 | 0 |
| 595.257954 | 595.258242 | 4443498 | 30 | 44 | 0 | 10 | 1 | 0 |
| 595.29088 | 595.291257 | 2671465 | 34 | 44 | 0 | 9 | 0 | 0 |
| 595.326866 | 595.32661 | 1552744 | 29 | 49 | 4 | 7 | 0 | 1 |
| 597.164755 | 597.164735 | 2232645 | 27 | 34 | 0 | 13 | 1 | 0 |
| 597.176289 | 597.176621 | 1659716 | 34 | 30 | 0 | 10 | 0 | 0 |
| 597.201038 | 597.201121 | 4128580 | 28 | 38 | 0 | 12 | 1 | 0 |
| 597.23741 | 597.237506 | 4648771 | 29 | 42 | 0 | 11 | 1 | 0 |
| 597.27051 | 597.270521 | 2414915 | 33 | 42 | 0 | 10 | 0 | 0 |
| 597.273729 | 597.273892 | 3010371 | 30 | 46 | 0 | 10 | 1 | 0 |
| 597.306285 | 597.305875 | 2113602 | 28 | 47 | 4 | 8 | 0 | 1 |
| 599.176688 | 599.177015 | 1591841 | 30 | 32 | 0 | 13 | 0 | 0 |
| 599.191942 | 599.192271 | 1496865 | 34 | 32 | 0 | 10 | 0 | 0 |
| 599.216644 | 599.216771 | 3897120 | 28 | 40 | 0 | 12 | 1 | 0 |
| 599.249546 | 599.249786 | 2852128 | 32 | 40 | 0 | 11 | 0 | 0 |
| 599.252842 | 599.253156 | 3687200 | 29 | 44 | 0 | 11 | 1 | 0 |
| 599.286028 | 599.286171 | 2950943 | 33 | 44 | 0 | 10 | 0 | 0 |
| 599.322229 | 599.322557 | 1746718 | 34 | 48 | 0 | 9 | 0 | 0 |
| 601.195739 | 601.196036 | 1941761 | 27 | 38 | 0 | 13 | 1 | 0 |
| 601.207526 | 601.207921 | 1597185 | 34 | 34 | 0 | 10 | 0 | 0 |
| 601.228788 | 601.22905 | 2044672 | 31 | 38 | 0 | 12 | 0 | 0 |
| 601.243951 | 601.244306 | 1612032 | 35 | 38 | 0 | 9 | 0 | 0 |
| 601.264438 | 601.264784 | 2549504 | 24 | 46 | 2 | 13 | 1 | 0 |
| 601.268714 | 601.268807 | 1657088 | 29 | 46 | 0 | 11 | 1 | 0 |
| 601.279823 | 601.28004 | 1512191 | 28 | 46 | 2 | 10 | 1 | 0 |
| 601.3013 | 601.301821 | 2125055 | 33 | 46 | 0 | 10 | 0 | 0 |
| 603.187312 | 603.187185 | 1384421 | 33 | 32 | 0 | 11 | 0 | 0 |
| 603.190619 | 603.190556 | 1550565 | 30 | 36 | 0 | 11 | 1 | 0 |
| 603.208148 | 603.208315 | 2022629 | 30 | 36 | 0 | 13 | 0 | 0 |
| 603.211907 | 603.211686 | 1354468 | 27 | 40 | 0 | 13 | 1 | 0 |
| 603.223236 | 603.223571 | 1672164 | 34 | 36 | 0 | 10 | 0 | 0 |
| 603.24462 | 603.2447 | 2517732 | 31 | 40 | 0 | 12 | 0 | 0 |
| 603.259895 | 603.259956 | 1670884 | 35 | 40 | 0 | 9 | 0 | 0 |
| 603.280831 | 603.281086 | 3151588 | 32 | 44 | 0 | 11 | 0 | 0 |
| 603.31721 | 603.317471 | 2238179 | 33 | 48 | 0 | 10 | 0 | 0 |
| 603.353704 | 603.353857 | 1480419 | 34 | 52 | 0 | 9 | 0 | 0 |
| 605.202885 | 605.202835 | 1907916 | 33 | 34 | 0 | 11 | 0 | 0 |
| 605.206214 | 605.206206 | 2248908 | 30 | 38 | 0 | 11 | 1 | 0 |
| 605.223481 | 605.223965 | 2356940 | 30 | 38 | 0 | 13 | 0 | 0 |
| 605.23911 | 605.239221 | 2036940 | 34 | 38 | 0 | 10 | 0 | 0 |
| 605.260185 | 605.26035 | 2414796 | 31 | 42 | 0 | 12 | 0 | 0 |
| 605.275567 | 605.275606 | 1930444 | 35 | 42 | 0 | 9 | 0 | 0 |
| 605.296408 | 605.296736 | 2029259 | 32 | 46 | 0 | 11 | 0 | 0 |
| 605.311717 | 605.311992 | 1528267 | 36 | 46 | 0 | 8 | 0 | 0 |
| 607.185299 | 607.185471 | 2185400 | 29 | 36 | 0 | 12 | 1 | 0 |
| 607.221607 | 607.221856 | 3118776 | 30 | 40 | 0 | 11 | 1 | 0 |
| 607.239294 | 607.239615 | 1686712 | 30 | 40 | 0 | 13 | 0 | 0 |
| 607.254792 | 607.254871 | 2301624 | 34 | 40 | 0 | 10 | 0 | 0 |
| 607.275707 | 607.276 | 1896632 | 31 | 44 | 0 | 12 | 0 | 0 |
| 607.290933 | 607.291257 | 2043831 | 35 | 44 | 0 | 9 | 0 | 0 |
| 607.312327 | 607.312386 | 1508279 | 32 | 48 | 0 | 11 | 0 | 0 |
| 608.294527 | 608.294585 | 1411759 | 29 | 47 | 5 | 5 | 2 | 0 |
| 609.146319 | 609.146108 | 1640617 | 27 | 30 | 0 | 16 | 0 | 0 |
| 609.197353 | 609.19775 | 2135208 | 32 | 34 | 0 | 12 | 0 | 0 |
| 609.200884 | 609.201121 | 3461288 | 29 | 38 | 0 | 12 | 1 | 0 |
| 609.2373 | 609.237506 | 4180648 | 30 | 42 | 0 | 11 | 1 | 0 |
| 609.254922 | 609.255265 | 1537448 | 30 | 42 | 0 | 13 | 0 | 0 |
| 609.270109 | 609.270521 | 2163368 | 34 | 42 | 0 | 10 | 0 | 0 |
| 609.306541 | 609.306907 | 1986727 | 35 | 46 | 0 | 9 | 0 | 0 |
| 611.179945 | 611.180385 | 2040092 | 28 | 36 | 0 | 13 | 1 | 0 |
| 611.216598 | 611.216771 | 3657372 | 29 | 40 | 0 | 12 | 1 | 0 |
| 611.25296 | 611.253156 | 4043420 | 30 | 44 | 0 | 11 | 1 | 0 |
| 611.286048 | 611.286171 | 2437788 | 34 | 44 | 0 | 10 | 0 | 0 |
| 611.322061 | 611.322557 | 1629340 | 35 | 48 | 0 | 9 | 0 | 0 |
| 613.195651 | 613.196036 | 2669205 | 28 | 38 | 0 | 13 | 1 | 0 |
| 613.227894 | 613.228398 | 2251924 | 24 | 42 | 2 | 14 | 1 | 0 |
| 613.232216 | 613.232421 | 3093140 | 29 | 42 | 0 | 12 | 1 | 0 |
| 613.265515 | 613.265436 | 2453140 | 33 | 42 | 0 | 11 | 0 | 0 |
| 613.268742 | 613.268807 | 2644116 | 30 | 46 | 0 | 11 | 1 | 0 |
| 613.280211 | 613.280692 | 1512340 | 37 | 42 | 0 | 8 | 0 | 0 |
| 613.301671 | 613.301821 | 1965716 | 34 | 46 | 0 | 10 | 0 | 0 |
| 613.337982 | 613.338207 | 1729172 | 35 | 50 | 0 | 9 | 0 | 0 |
| 615.207779 | 615.208315 | 1730961 | 31 | 36 | 0 | 13 | 0 | 0 |
| 615.211603 | 615.211686 | 2148497 | 28 | 40 | 0 | 13 | 1 | 0 |
| 615.223322 | 615.223571 | 1507473 | 35 | 36 | 0 | 10 | 0 | 0 |
| 615.24451 | 615.2447 | 2038929 | 32 | 40 | 0 | 12 | 0 | 0 |
| 615.259997 | 615.259956 | 1560465 | 36 | 40 | 0 | 9 | 0 | 0 |
| 615.280755 | 615.281086 | 2634385 | 33 | 44 | 0 | 11 | 0 | 0 |
| 615.317232 | 615.317471 | 2149265 | 34 | 48 | 0 | 10 | 0 | 0 |
| 617.202543 | 617.202835 | 1560210 | 34 | 34 | 0 | 11 | 0 | 0 |
| 617.223364 | 617.223965 | 1598354 | 31 | 38 | 0 | 13 | 0 | 0 |
| 617.23897 | 617.239221 | 1771922 | 35 | 38 | 0 | 10 | 0 | 0 |
| 617.260031 | 617.26035 | 2454162 | 32 | 42 | 0 | 12 | 0 | 0 |
| 617.275448 | 617.275606 | 1530514 | 36 | 42 | 0 | 9 | 0 | 0 |
| 617.296779 | 617.296736 | 2121874 | 33 | 46 | 0 | 11 | 0 | 0 |
| 617.31104 | 617.31134 | 1419666 | 29 | 50 | 2 | 10 | 1 | 0 |
| 619.185294 | 619.185471 | 1537176 | 30 | 36 | 0 | 12 | 1 | 0 |
| 619.218116 | 619.218486 | 1418392 | 34 | 36 | 0 | 11 | 0 | 0 |
| 619.239287 | 619.239615 | 1901464 | 31 | 40 | 0 | 13 | 0 | 0 |
| 619.254409 | 619.254871 | 1867160 | 35 | 40 | 0 | 10 | 0 | 0 |
| 619.275484 | 619.276 | 2345880 | 32 | 44 | 0 | 12 | 0 | 0 |
| 619.290588 | 619.290605 | 1511320 | 28 | 48 | 2 | 11 | 1 | 0 |
| 621.200945 | 621.201121 | 1859234 | 30 | 38 | 0 | 12 | 1 | 0 |
| 621.234041 | 621.234136 | 1744034 | 34 | 38 | 0 | 11 | 0 | 0 |
| 621.237481 | 621.237506 | 1591970 | 31 | 42 | 0 | 11 | 1 | 0 |
| 621.255145 | 621.255265 | 1787042 | 31 | 42 | 0 | 13 | 0 | 0 |
| 621.270352 | 621.270521 | 1969827 | 35 | 42 | 0 | 10 | 0 | 0 |
| 621.291147 | 621.29165 | 1800867 | 32 | 46 | 0 | 12 | 0 | 0 |
| 621.306501 | 621.306907 | 1606691 | 36 | 46 | 0 | 9 | 0 | 0 |
| 623.180196 | 623.180385 | 1672369 | 29 | 36 | 0 | 13 | 1 | 0 |
| 623.216477 | 623.216771 | 2642097 | 30 | 40 | 0 | 12 | 1 | 0 |
| 623.249574 | 623.249786 | 2347698 | 34 | 40 | 0 | 11 | 0 | 0 |
| 623.285864 | 623.286171 | 2146482 | 35 | 44 | 0 | 10 | 0 | 0 |
| 623.322451 | 623.322557 | 1808050 | 36 | 48 | 0 | 9 | 0 | 0 |
| 625.196017 | 625.196036 | 1965253 | 29 | 38 | 0 | 13 | 1 | 0 |
| 625.232194 | 625.232421 | 3773125 | 30 | 42 | 0 | 12 | 1 | 0 |
| 625.264899 | 625.265436 | 2028998 | 34 | 42 | 0 | 11 | 0 | 0 |
| 625.301694 | 625.301821 | 2588358 | 35 | 46 | 0 | 10 | 0 | 0 |
| 626.303582 | 626.304084 | 1329362 | 38 | 46 | 1 | 5 | 0 | 1 |
| 627.211397 | 627.211686 | 2243742 | 29 | 40 | 0 | 13 | 1 | 0 |
| 627.24424 | 627.2447 | 2269918 | 33 | 40 | 0 | 12 | 0 | 0 |
| 627.247574 | 627.248071 | 2657246 | 30 | 44 | 0 | 12 | 1 | 0 |
| 627.258948 | 627.258924 | 1606110 | 31 | 41 | 4 | 8 | 0 | 1 |
| 627.280743 | 627.281086 | 1879263 | 34 | 44 | 0 | 11 | 0 | 0 |
| 627.316853 | 627.317471 | 1568607 | 35 | 48 | 0 | 10 | 0 | 0 |
| 629.227339 | 629.227336 | 1852411 | 29 | 42 | 0 | 13 | 1 | 0 |
| 629.259989 | 629.26035 | 1959675 | 33 | 42 | 0 | 12 | 0 | 0 |
| 629.296063 | 629.296084 | 2070012 | 26 | 50 | 2 | 13 | 1 | 0 |
| 629.332087 | 629.332469 | 1328508 | 27 | 54 | 2 | 12 | 1 | 0 |
| 631.238383 | 631.238963 | 1482524 | 24 | 44 | 2 | 15 | 1 | 0 |
| 631.254451 | 631.254871 | 1611548 | 36 | 40 | 0 | 10 | 0 | 0 |
| 631.275816 | 631.276 | 2180381 | 33 | 44 | 0 | 12 | 0 | 0 |
| 631.290691 | 631.291257 | 1571101 | 37 | 44 | 0 | 9 | 0 | 0 |
| 631.311029 | 631.311157 | 1546013 | 38 | 50 | 0 | 4 | 0 | 2 |
| 633.16093 | 633.161365 | 1386303 | 33 | 30 | 0 | 13 | 0 | 0 |
| 633.197523 | 633.19775 | 1345343 | 34 | 34 | 0 | 12 | 0 | 0 |
| 633.233579 | 633.234136 | 1711680 | 35 | 38 | 0 | 11 | 0 | 0 |
| 633.254023 | 633.254613 | 1926464 | 24 | 46 | 2 | 15 | 1 | 0 |
| 633.270186 | 633.270521 | 1694529 | 36 | 42 | 0 | 10 | 0 | 0 |
| 633.306831 | 633.306907 | 1448769 | 37 | 46 | 0 | 9 | 0 | 0 |
| 633.32788 | 633.328036 | 1532482 | 34 | 50 | 0 | 11 | 0 | 0 |
| 635.212321 | 635.212748 | 1372519 | 26 | 40 | 2 | 14 | 1 | 0 |
| 635.249607 | 635.249786 | 2208104 | 35 | 40 | 0 | 11 | 0 | 0 |
| 635.252995 | 635.253156 | 1423720 | 32 | 44 | 0 | 11 | 1 | 0 |
| 635.27061 | 635.270915 | 1575528 | 32 | 44 | 0 | 13 | 0 | 0 |
| 635.285228 | 635.285519 | 2212712 | 28 | 48 | 2 | 12 | 1 | 0 |
| 635.322437 | 635.322557 | 1705321 | 37 | 48 | 0 | 9 | 0 | 0 |
| 637.195821 | 637.196036 | 1877904 | 30 | 38 | 0 | 13 | 1 | 0 |
| 637.228184 | 637.228398 | 1739409 | 26 | 42 | 2 | 14 | 1 | 0 |
| 637.26479 | 637.264784 | 2071954 | 27 | 46 | 2 | 13 | 1 | 0 |
| 637.28641 | 637.286565 | 1417362 | 32 | 46 | 0 | 13 | 0 | 0 |
| 637.301407 | 637.301821 | 1660819 | 36 | 46 | 0 | 10 | 0 | 0 |
| 637.337876 | 637.338207 | 1417619 | 37 | 50 | 0 | 9 | 0 | 0 |
| 639.211487 | 639.211686 | 2800573 | 30 | 40 | 0 | 13 | 1 | 0 |
| 639.243935 | 639.244048 | 2117565 | 26 | 44 | 2 | 14 | 1 | 0 |
| 639.247765 | 639.248071 | 1653438 | 31 | 44 | 0 | 12 | 1 | 0 |
| 639.280828 | 639.281086 | 2630590 | 35 | 44 | 0 | 11 | 0 | 0 |
| 639.317274 | 639.317471 | 2203583 | 36 | 48 | 0 | 10 | 0 | 0 |
| 641.190904 | 641.191089 | 1793002 | 50 | 26 | 0 | 1 | 0 | 0 |
| 641.227201 | 641.227336 | 3054571 | 30 | 42 | 0 | 13 | 1 | 0 |
| 641.260075 | 641.26035 | 2039788 | 34 | 42 | 0 | 12 | 0 | 0 |
| 641.296246 | 641.296736 | 2337260 | 35 | 46 | 0 | 11 | 0 | 0 |
| 641.333258 | 641.333121 | 1602797 | 36 | 50 | 0 | 10 | 0 | 0 |
| 642.299812 | 642.299195 | 1324036 | 28 | 45 | 5 | 12 | 0 | 0 |
| 643.202919 | 643.203229 | 1373977 | 32 | 36 | 0 | 14 | 0 | 0 |
| 643.242639 | 643.242986 | 1808666 | 30 | 44 | 0 | 13 | 1 | 0 |
| 643.27575 | 643.276 | 2372635 | 34 | 44 | 0 | 12 | 0 | 0 |
| 643.31194 | 643.312386 | 1889820 | 35 | 48 | 0 | 11 | 0 | 0 |
| 645.218289 | 645.218879 | 1553482 | 32 | 38 | 0 | 14 | 0 | 0 |
| 645.254823 | 645.255265 | 1907531 | 33 | 42 | 0 | 13 | 0 | 0 |
| 645.270247 | 645.270521 | 1355595 | 37 | 42 | 0 | 10 | 0 | 0 |
| 645.291553 | 645.29165 | 2028619 | 34 | 46 | 0 | 12 | 0 | 0 |
| 645.327705 | 645.328036 | 1707852 | 35 | 50 | 0 | 11 | 0 | 0 |
| 647.234318 | 647.23453 | 1456507 | 32 | 40 | 0 | 14 | 0 | 0 |
| 647.248893 | 647.249134 | 1735035 | 28 | 44 | 2 | 13 | 1 | 0 |
| 647.270578 | 647.270915 | 1646715 | 33 | 44 | 0 | 13 | 0 | 0 |
| 647.306483 | 647.306649 | 1631100 | 26 | 52 | 2 | 14 | 1 | 0 |
| 649.250027 | 649.25018 | 1676971 | 32 | 42 | 0 | 14 | 0 | 0 |
| 649.264711 | 649.264784 | 1600940 | 28 | 46 | 2 | 13 | 1 | 0 |
| 649.301249 | 649.301821 | 1506221 | 37 | 46 | 0 | 10 | 0 | 0 |
| 651.244509 | 651.2447 | 1348443 | 35 | 40 | 0 | 12 | 0 | 0 |
| 651.280644 | 651.281086 | 1674972 | 36 | 44 | 0 | 11 | 0 | 0 |
| 653.226885 | 653.227336 | 1760266 | 31 | 42 | 0 | 13 | 1 | 0 |
| 653.259932 | 653.26035 | 1971979 | 35 | 42 | 0 | 12 | 0 | 0 |
| 653.296136 | 653.296736 | 2368780 | 36 | 46 | 0 | 11 | 0 | 0 |
| 653.332651 | 653.333121 | 1561101 | 37 | 50 | 0 | 10 | 0 | 0 |
| 655.206085 | 655.2066 | 1637688 | 30 | 40 | 0 | 14 | 1 | 0 |
| 655.238959 | 655.238963 | 1657785 | 26 | 44 | 2 | 15 | 1 | 0 |
| 655.275488 | 655.276 | 2228281 | 35 | 44 | 0 | 12 | 0 | 0 |
| 655.312111 | 655.312386 | 1454906 | 36 | 48 | 0 | 11 | 0 | 0 |
| 655.348869 | 655.348771 | 1447227 | 37 | 52 | 0 | 10 | 0 | 0 |
| 657.22213 | 657.22225 | 1781093 | 30 | 42 | 0 | 14 | 1 | 0 |
| 657.255076 | 657.255265 | 1352037 | 34 | 42 | 0 | 13 | 0 | 0 |
| 657.290529 | 657.290999 | 1815654 | 27 | 50 | 2 | 14 | 1 | 0 |
| 657.327591 | 657.328036 | 1760615 | 36 | 50 | 0 | 11 | 0 | 0 |
| 659.234077 | 659.23453 | 1434511 | 33 | 40 | 0 | 14 | 0 | 0 |
| 659.249262 | 659.249786 | 1338511 | 37 | 40 | 0 | 11 | 0 | 0 |
| 659.270834 | 659.270915 | 1744272 | 34 | 44 | 0 | 13 | 0 | 0 |
| 659.305551 | 659.304931 | 1675153 | 28 | 53 | 0 | 15 | 0 | 1 |
| 661.249996 | 661.25018 | 1530295 | 33 | 42 | 0 | 14 | 0 | 0 |
| 661.264897 | 661.265436 | 1424696 | 37 | 42 | 0 | 11 | 0 | 0 |
| 661.286263 | 661.286565 | 1849016 | 34 | 46 | 0 | 13 | 0 | 0 |
| 661.301815 | 661.301821 | 1630648 | 38 | 46 | 0 | 10 | 0 | 0 |
| 661.322852 | 661.322951 | 1675193 | 35 | 50 | 0 | 12 | 0 | 0 |
| 663.280683 | 663.281086 | 1731549 | 37 | 44 | 0 | 11 | 0 | 0 |
| 663.301891 | 663.302215 | 1737693 | 34 | 48 | 0 | 13 | 0 | 0 |
| 663.317093 | 663.317471 | 1721053 | 38 | 48 | 0 | 10 | 0 | 0 |
| 665.227476 | 665.227336 | 1410045 | 32 | 42 | 0 | 13 | 1 | 0 |
| 665.260142 | 665.26035 | 1723902 | 36 | 42 | 0 | 12 | 0 | 0 |
| 665.296672 | 665.296736 | 1462526 | 37 | 46 | 0 | 11 | 0 | 0 |
| 665.332532 | 665.333121 | 1431935 | 38 | 50 | 0 | 10 | 0 | 0 |
| 667.242784 | 667.242986 | 1491739 | 32 | 44 | 0 | 13 | 1 | 0 |
| 667.274825 | 667.275348 | 1901276 | 28 | 48 | 2 | 14 | 1 | 0 |
| 669.222278 | 669.22225 | 1642036 | 31 | 42 | 0 | 14 | 1 | 0 |
| 669.233814 | 669.234136 | 1347636 | 38 | 38 | 0 | 11 | 0 | 0 |
| 669.254857 | 669.255265 | 1909813 | 35 | 42 | 0 | 13 | 0 | 0 |
| 669.290543 | 669.290999 | 1864757 | 28 | 50 | 2 | 14 | 1 | 0 |
| 669.32775 | 669.328036 | 1742390 | 37 | 50 | 0 | 11 | 0 | 0 |
| 671.201327 | 671.201515 | 1337417 | 30 | 40 | 0 | 15 | 1 | 0 |
| 671.270302 | 671.270915 | 1876298 | 35 | 44 | 0 | 13 | 0 | 0 |
| 671.306647 | 671.307301 | 1590858 | 36 | 48 | 0 | 12 | 0 | 0 |
| 671.343298 | 671.343686 | 1344586 | 37 | 52 | 0 | 11 | 0 | 0 |
| 673.228589 | 673.22905 | 1322585 | 37 | 38 | 0 | 12 | 0 | 0 |
| 673.249533 | 673.25018 | 1446873 | 34 | 42 | 0 | 14 | 0 | 0 |
| 673.285447 | 673.285913 | 1812057 | 27 | 50 | 2 | 15 | 1 | 0 |
| 673.321937 | 673.322299 | 1454554 | 28 | 54 | 2 | 14 | 1 | 0 |
| 675.301863 | 675.302215 | 1572452 | 35 | 48 | 0 | 13 | 0 | 0 |
| 675.31728 | 675.317471 | 1466980 | 39 | 48 | 0 | 10 | 0 | 0 |
| 677.223623 | 677.223965 | 1404520 | 36 | 38 | 0 | 13 | 0 | 0 |
| 677.281312 | 677.28148 | 1358184 | 34 | 46 | 0 | 14 | 0 | 0 |
| 677.295793 | 677.296084 | 1486696 | 30 | 50 | 2 | 13 | 1 | 0 |
| 677.332713 | 677.333121 | 1762920 | 39 | 50 | 0 | 10 | 0 | 0 |
| 679.239146 | 679.239615 | 1523558 | 36 | 40 | 0 | 13 | 0 | 0 |
| 679.275604 | 679.276 | 1945190 | 37 | 44 | 0 | 12 | 0 | 0 |
| 679.311919 | 679.312386 | 1907814 | 38 | 48 | 0 | 11 | 0 | 0 |
| 679.348606 | 679.348771 | 1551461 | 39 | 52 | 0 | 10 | 0 | 0 |
| 681.221801 | 681.22225 | 1397597 | 32 | 42 | 0 | 14 | 1 | 0 |
| 681.254226 | 681.254613 | 1796957 | 28 | 46 | 2 | 15 | 1 | 0 |
| 681.291175 | 681.29165 | 1604445 | 37 | 46 | 0 | 12 | 0 | 0 |
| 681.326775 | 681.327384 | 1783900 | 30 | 54 | 2 | 13 | 1 | 0 |
| 683.270193 | 683.270263 | 1682509 | 28 | 48 | 2 | 15 | 1 | 0 |
| 683.306806 | 683.307301 | 1497420 | 37 | 48 | 0 | 12 | 0 | 0 |
| 683.342861 | 683.343034 | 1769292 | 30 | 56 | 2 | 13 | 1 | 0 |
| 685.286124 | 685.286565 | 1544245 | 36 | 46 | 0 | 13 | 0 | 0 |
| 685.322654 | 685.322951 | 1436980 | 37 | 50 | 0 | 12 | 0 | 0 |
| 687.265357 | 687.26583 | 1808917 | 35 | 44 | 0 | 14 | 0 | 0 |
| 687.301815 | 687.302215 | 2077205 | 36 | 48 | 0 | 13 | 0 | 0 |
| 687.338637 | 687.338601 | 1482132 | 37 | 52 | 0 | 12 | 0 | 0 |
| 689.280655 | 689.280828 | 1665517 | 27 | 50 | 2 | 16 | 1 | 0 |
| 689.296693 | 689.296736 | 1467372 | 39 | 46 | 0 | 11 | 0 | 0 |
| 689.316654 | 689.317213 | 1791340 | 28 | 54 | 2 | 15 | 1 | 0 |
| 691.275721 | 691.276 | 1330364 | 38 | 44 | 0 | 12 | 0 | 0 |
| 693.291499 | 693.29165 | 1680257 | 38 | 46 | 0 | 12 | 0 | 0 |
| 693.328103 | 693.328036 | 1340031 | 39 | 50 | 0 | 11 | 0 | 0 |
| 695.238001 | 695.2379 | 1444671 | 33 | 44 | 0 | 14 | 1 | 0 |
| 695.270708 | 695.270915 | 1399358 | 37 | 44 | 0 | 13 | 0 | 0 |
| 695.306779 | 695.307301 | 1758780 | 38 | 48 | 0 | 12 | 0 | 0 |
| 697.286143 | 697.286565 | 2186481 | 37 | 46 | 0 | 13 | 0 | 0 |
| 697.321764 | 697.322299 | 1847023 | 30 | 54 | 2 | 14 | 1 | 0 |
| 699.263725 | 699.26346 | 1512094 | 29 | 49 | 0 | 17 | 0 | 1 |
| 699.301599 | 699.302215 | 1992860 | 37 | 48 | 0 | 13 | 0 | 0 |
| 699.336678 | 699.336098 | 1327258 | 42 | 52 | 0 | 7 | 1 | 0 |

2. Sample: F2_T_0_

| Experimental mass | Exact mass | Peak height | C | H | N | O | S | P |
| --- | --- | --- | --- | --- | --- | --- | --- | --- |
| 215.10774 | 215.107753 | 1084797 | 14 | 16 | 0 | 2 | 0 | 0 |
| 217.12344 | 217.123403 | 1645910 | 14 | 18 | 0 | 2 | 0 | 0 |
| 219.102686 | 219.102668 | 1503502 | 13 | 16 | 0 | 3 | 0 | 0 |
| 221.118149 | 221.118318 | 1306616 | 13 | 18 | 0 | 3 | 0 | 0 |
| 223.097593 | 223.097583 | 1500228 | 12 | 16 | 0 | 4 | 0 | 0 |
| 223.133983 | 223.133968 | 1104690 | 13 | 20 | 0 | 3 | 0 | 0 |
| 225.076853 | 225.076847 | 1337655 | 11 | 14 | 0 | 5 | 0 | 0 |
| 227.10775 | 227.107753 | 1493647 | 15 | 16 | 0 | 2 | 0 | 0 |
| 227.144182 | 227.144139 | 1413858 | 16 | 20 | 0 | 1 | 0 | 0 |
| 229.050642 | 229.050632 | 1182911 | 13 | 10 | 0 | 4 | 0 | 0 |
| 229.087017 | 229.087018 | 1918002 | 14 | 14 | 0 | 3 | 0 | 0 |
| 229.123399 | 229.123403 | 2210165 | 15 | 18 | 0 | 2 | 0 | 0 |
| 229.159812 | 229.159789 | 1199143 | 16 | 22 | 0 | 1 | 0 | 0 |
| 231.066313 | 231.066282 | 2162032 | 13 | 12 | 0 | 4 | 0 | 0 |
| 231.10269 | 231.102668 | 4016371 | 14 | 16 | 0 | 3 | 0 | 0 |
| 231.139056 | 231.139053 | 3776182 | 15 | 20 | 0 | 2 | 0 | 0 |
| 231.175451 | 231.175439 | 1301881 | 16 | 24 | 0 | 1 | 0 | 0 |
| 233.081943 | 233.081932 | 2822749 | 13 | 14 | 0 | 4 | 0 | 0 |
| 233.118361 | 233.118318 | 4138400 | 14 | 18 | 0 | 3 | 0 | 0 |
| 233.154727 | 233.154703 | 2846499 | 15 | 22 | 0 | 2 | 0 | 0 |
| 235.024867 | 235.024812 | 1240494 | 11 | 8 | 0 | 6 | 0 | 0 |
| 235.061228 | 235.061197 | 1560273 | 12 | 12 | 0 | 5 | 0 | 0 |
| 235.097612 | 235.097583 | 3887636 | 13 | 16 | 0 | 4 | 0 | 0 |
| 235.13401 | 235.133968 | 3116760 | 14 | 20 | 0 | 3 | 0 | 0 |
| 235.170387 | 235.170354 | 1261531 | 15 | 24 | 0 | 2 | 0 | 0 |
| 237.076886 | 237.076847 | 3318354 | 12 | 14 | 0 | 5 | 0 | 0 |
| 237.092144 | 237.092103 | 1170132 | 16 | 14 | 0 | 2 | 0 | 0 |
| 237.113263 | 237.113233 | 4362326 | 13 | 18 | 0 | 4 | 0 | 0 |
| 239.09253 | 239.092497 | 2921372 | 12 | 16 | 0 | 5 | 0 | 0 |
| 239.107757 | 239.107753 | 2143838 | 16 | 16 | 0 | 2 | 0 | 0 |
| 239.144163 | 239.144139 | 1952802 | 17 | 20 | 0 | 1 | 0 | 0 |
| 241.050661 | 241.050632 | 1892648 | 14 | 10 | 0 | 4 | 0 | 0 |
| 241.087045 | 241.087018 | 3414508 | 15 | 14 | 0 | 3 | 0 | 0 |
| 241.123422 | 241.123403 | 5757552 | 16 | 18 | 0 | 2 | 0 | 0 |
| 241.159812 | 241.159789 | 3322804 | 17 | 22 | 0 | 1 | 0 | 0 |
| 243.029929 | 243.029897 | 1625214 | 13 | 8 | 0 | 5 | 0 | 0 |
| 243.066298 | 243.066282 | 3570626 | 14 | 12 | 0 | 4 | 0 | 0 |
| 243.102692 | 243.102668 | 7136070 | 15 | 16 | 0 | 3 | 0 | 0 |
| 243.1391 | 243.139053 | 8976457 | 16 | 20 | 0 | 2 | 0 | 0 |
| 243.175448 | 243.175439 | 3447758 | 17 | 24 | 0 | 1 | 0 | 0 |
| 245.045587 | 245.045547 | 2381021 | 13 | 10 | 0 | 5 | 0 | 0 |
| 245.081965 | 245.081932 | 4858785 | 14 | 14 | 0 | 4 | 0 | 0 |
| 245.118329 | 245.118318 | 9722917 | 15 | 18 | 0 | 3 | 0 | 0 |
| 245.154736 | 245.154703 | 7861801 | 16 | 22 | 0 | 2 | 0 | 0 |
| 245.191113 | 245.191089 | 1265677 | 17 | 26 | 0 | 1 | 0 | 0 |
| 247.024837 | 247.024812 | 2501629 | 12 | 8 | 0 | 6 | 0 | 0 |
| 247.061233 | 247.061197 | 5004546 | 13 | 12 | 0 | 5 | 0 | 0 |
| 247.097608 | 247.097583 | 10881030 | 14 | 16 | 0 | 4 | 0 | 0 |
| 247.133979 | 247.133968 | 13254410 | 15 | 20 | 0 | 3 | 0 | 0 |
| 247.170388 | 247.170354 | 5389838 | 16 | 24 | 0 | 2 | 0 | 0 |
| 249.040515 | 249.040462 | 2095334 | 12 | 10 | 0 | 6 | 0 | 0 |
| 249.076856 | 249.076847 | 4500459 | 13 | 14 | 0 | 5 | 0 | 0 |
| 249.113243 | 249.113233 | 9041391 | 14 | 18 | 0 | 4 | 0 | 0 |
| 249.128536 | 249.128489 | 1302576 | 18 | 18 | 0 | 1 | 0 | 0 |
| 249.185979 | 249.186004 | 1638135 | 16 | 26 | 0 | 2 | 0 | 0 |
| 251.01969 | 251.019726 | 1120414 | 11 | 8 | 0 | 7 | 0 | 0 |
| 251.056143 | 251.056112 | 2774611 | 12 | 12 | 0 | 6 | 0 | 0 |
| 251.07139 | 251.071368 | 1961876 | 16 | 12 | 0 | 3 | 0 | 0 |
| 251.092504 | 251.092497 | 8586711 | 13 | 16 | 0 | 5 | 0 | 0 |
| 251.107785 | 251.107753 | 3638617 | 17 | 16 | 0 | 2 | 0 | 0 |
| 251.128916 | 251.128883 | 11020251 | 14 | 20 | 0 | 4 | 0 | 0 |
| 251.144159 | 251.144139 | 3290589 | 18 | 20 | 0 | 1 | 0 | 0 |
| 253.050638 | 253.050632 | 2566207 | 15 | 10 | 0 | 4 | 0 | 0 |
| 253.071759 | 253.071762 | 2838658 | 12 | 14 | 0 | 6 | 0 | 0 |
| 253.087051 | 253.087018 | 3442499 | 16 | 14 | 0 | 3 | 0 | 0 |
| 253.108177 | 253.108147 | 6018886 | 13 | 18 | 0 | 5 | 0 | 0 |
| 253.123426 | 253.123403 | 6655176 | 17 | 18 | 0 | 2 | 0 | 0 |
| 253.15981 | 253.159789 | 3980748 | 18 | 22 | 0 | 1 | 0 | 0 |
| 255.029911 | 255.029897 | 2445803 | 14 | 8 | 0 | 5 | 0 | 0 |
| 255.066318 | 255.066282 | 4848560 | 15 | 12 | 0 | 4 | 0 | 0 |
| 255.087443 | 255.087412 | 3461170 | 12 | 16 | 0 | 6 | 0 | 0 |
| 255.102687 | 255.102668 | 10199220 | 16 | 16 | 0 | 3 | 0 | 0 |
| 255.139082 | 255.139053 | 15686840 | 17 | 20 | 0 | 2 | 0 | 0 |
| 255.175453 | 255.175439 | 7382461 | 18 | 24 | 0 | 1 | 0 | 0 |
| 256.074172 | 256.074418 | 1137768 | 11 | 16 | 1 | 4 | 0 | 1 |
| 257.045582 | 257.045547 | 4016669 | 14 | 10 | 0 | 5 | 0 | 0 |
| 257.081942 | 257.081932 | 6944801 | 15 | 14 | 0 | 4 | 0 | 0 |
| 257.103062 | 257.103062 | 1643236 | 12 | 18 | 0 | 6 | 0 | 0 |
| 257.11835 | 257.118318 | 14030758 | 16 | 18 | 0 | 3 | 0 | 0 |
| 257.154714 | 257.154703 | 15930794 | 17 | 22 | 0 | 2 | 0 | 0 |
| 257.19111 | 257.191089 | 4190510 | 18 | 26 | 0 | 1 | 0 | 0 |
| 258.077243 | 258.077181 | 1151768 | 14 | 13 | 1 | 4 | 0 | 0 |
| 258.113593 | 258.113567 | 1237789 | 15 | 17 | 1 | 3 | 0 | 0 |
| 259.024797 | 259.024812 | 4029322 | 13 | 8 | 0 | 6 | 0 | 0 |
| 259.061223 | 259.061197 | 7619470 | 14 | 12 | 0 | 5 | 0 | 0 |
| 259.097607 | 259.097583 | 16355987 | 15 | 16 | 0 | 4 | 0 | 0 |
| 259.133982 | 259.133968 | 26106518 | 16 | 20 | 0 | 3 | 0 | 0 |
| 259.170381 | 259.170354 | 15599259 | 17 | 24 | 0 | 2 | 0 | 0 |
| 259.206656 | 259.206739 | 1491556 | 18 | 28 | 0 | 1 | 0 | 0 |
| 260.092892 | 260.092832 | 1300041 | 14 | 15 | 1 | 4 | 0 | 0 |
| 260.129288 | 260.129217 | 1126542 | 15 | 19 | 1 | 3 | 0 | 0 |
| 261.004155 | 261.004076 | 1563254 | 12 | 6 | 0 | 7 | 0 | 0 |
| 261.040465 | 261.040462 | 4337530 | 13 | 10 | 0 | 6 | 0 | 0 |
| 261.076859 | 261.076847 | 7590015 | 14 | 14 | 0 | 5 | 0 | 0 |
| 261.092125 | 261.092103 | 1203008 | 18 | 14 | 0 | 2 | 0 | 0 |
| 261.113259 | 261.113233 | 17792898 | 15 | 18 | 0 | 4 | 0 | 0 |
| 261.149621 | 261.149618 | 17358216 | 16 | 22 | 0 | 3 | 0 | 0 |
| 261.186019 | 261.186004 | 5286668 | 17 | 26 | 0 | 2 | 0 | 0 |
| 263.019764 | 263.019726 | 2916772 | 12 | 8 | 0 | 7 | 0 | 0 |
| 263.056134 | 263.056112 | 5239401 | 13 | 12 | 0 | 6 | 0 | 0 |
| 263.071419 | 263.071368 | 1998058 | 17 | 12 | 0 | 3 | 0 | 0 |
| 263.092517 | 263.092497 | 14104173 | 14 | 16 | 0 | 5 | 0 | 0 |
| 263.107768 | 263.107753 | 2856815 | 18 | 16 | 0 | 2 | 0 | 0 |
| 263.144137 | 263.144139 | 2934259 | 19 | 20 | 0 | 1 | 0 | 0 |
| 263.201625 | 263.201654 | 1392090 | 17 | 28 | 0 | 2 | 0 | 0 |
| 265.035359 | 265.035376 | 2431952 | 12 | 10 | 0 | 7 | 0 | 0 |
| 265.050589 | 265.050632 | 1683794 | 16 | 10 | 0 | 4 | 0 | 0 |
| 265.071752 | 265.071762 | 5521620 | 13 | 14 | 0 | 6 | 0 | 0 |
| 265.087018 | 265.087018 | 3249942 | 17 | 14 | 0 | 3 | 0 | 0 |
| 265.108157 | 265.108147 | 14362968 | 14 | 18 | 0 | 5 | 0 | 0 |
| 265.123417 | 265.123403 | 6480730 | 18 | 18 | 0 | 2 | 0 | 0 |
| 265.159811 | 265.159789 | 4789599 | 19 | 22 | 0 | 1 | 0 | 0 |
| 267.029854 | 267.029897 | 2031157 | 15 | 8 | 0 | 5 | 0 | 0 |
| 267.051041 | 267.051026 | 2041080 | 12 | 12 | 0 | 7 | 0 | 0 |
| 267.06629 | 267.066282 | 5153338 | 16 | 12 | 0 | 4 | 0 | 0 |
| 267.08743 | 267.087412 | 7320124 | 13 | 16 | 0 | 6 | 0 | 0 |
| 267.102695 | 267.102668 | 9968190 | 17 | 16 | 0 | 3 | 0 | 0 |
| 267.123814 | 267.123797 | 13542464 | 14 | 20 | 0 | 5 | 0 | 0 |
| 267.139079 | 267.139053 | 14861378 | 18 | 20 | 0 | 2 | 0 | 0 |
| 267.17546 | 267.175439 | 7603782 | 19 | 24 | 0 | 1 | 0 | 0 |
| 269.009218 | 269.009161 | 1325845 | 14 | 6 | 0 | 6 | 0 | 0 |
| 269.045555 | 269.045547 | 5535769 | 15 | 10 | 0 | 5 | 0 | 0 |
| 269.066714 | 269.066676 | 2019675 | 12 | 14 | 0 | 7 | 0 | 0 |
| 269.081949 | 269.081932 | 9110557 | 16 | 14 | 0 | 4 | 0 | 0 |
| 269.103076 | 269.103062 | 5344031 | 13 | 18 | 0 | 6 | 0 | 0 |
| 269.118336 | 269.118318 | 18269984 | 17 | 18 | 0 | 3 | 0 | 0 |
| 269.154712 | 269.154703 | 22372132 | 18 | 22 | 0 | 2 | 0 | 0 |
| 269.191084 | 269.191089 | 6703145 | 19 | 26 | 0 | 1 | 0 | 0 |
| 270.113529 | 270.113567 | 1197455 | 16 | 17 | 1 | 3 | 0 | 0 |
| 270.126232 | 270.126454 | 1204560 | 13 | 22 | 1 | 3 | 0 | 1 |
| 271.024839 | 271.024812 | 4455155 | 14 | 8 | 0 | 6 | 0 | 0 |
| 271.061218 | 271.061197 | 8061431 | 15 | 12 | 0 | 5 | 0 | 0 |
| 271.082327 | 271.082326 | 1643065 | 12 | 16 | 0 | 7 | 0 | 0 |
| 271.097595 | 271.097583 | 15141882 | 16 | 16 | 0 | 4 | 0 | 0 |
| 271.108852 | 271.108816 | 2132476 | 15 | 16 | 2 | 3 | 0 | 0 |
| 271.133977 | 271.133968 | 29027326 | 17 | 20 | 0 | 3 | 0 | 0 |
| 271.170375 | 271.170354 | 21828610 | 18 | 24 | 0 | 2 | 0 | 0 |
| 271.206773 | 271.206739 | 3408902 | 19 | 28 | 0 | 1 | 0 | 0 |
| 272.069063 | 272.069333 | 1776515 | 11 | 16 | 1 | 5 | 0 | 1 |
| 272.092847 | 272.092832 | 2153829 | 15 | 15 | 1 | 4 | 0 | 0 |
| 272.105458 | 272.105718 | 2116583 | 12 | 20 | 1 | 4 | 0 | 1 |
| 272.129219 | 272.129217 | 1884521 | 16 | 19 | 1 | 3 | 0 | 0 |
| 273.004103 | 273.004076 | 1935814 | 13 | 6 | 0 | 7 | 0 | 0 |
| 273.040482 | 273.040462 | 8241354 | 14 | 10 | 0 | 6 | 0 | 0 |
| 273.076865 | 273.076847 | 13018318 | 15 | 14 | 0 | 5 | 0 | 0 |
| 273.113241 | 273.113233 | 27526354 | 16 | 18 | 0 | 4 | 0 | 0 |
| 273.124489 | 273.124466 | 2862675 | 15 | 18 | 2 | 3 | 0 | 0 |
| 273.149627 | 273.149618 | 37641428 | 17 | 22 | 0 | 3 | 0 | 0 |
| 273.186013 | 273.186004 | 16038617 | 18 | 26 | 0 | 2 | 0 | 0 |
| 273.207142 | 273.207133 | 1784155 | 15 | 30 | 0 | 4 | 0 | 0 |
| 273.22239 | 273.222389 | 1524893 | 19 | 30 | 0 | 1 | 0 | 0 |
| 274.072098 | 274.072096 | 1734005 | 14 | 13 | 1 | 5 | 0 | 0 |
| 274.10843 | 274.108482 | 2025145 | 15 | 17 | 1 | 4 | 0 | 0 |
| 275.019746 | 275.019726 | 3319062 | 13 | 8 | 0 | 7 | 0 | 0 |
| 275.056134 | 275.056112 | 6675098 | 14 | 12 | 0 | 6 | 0 | 0 |
| 275.071384 | 275.071368 | 1227995 | 18 | 12 | 0 | 3 | 0 | 0 |
| 275.092503 | 275.092497 | 13602205 | 15 | 16 | 0 | 5 | 0 | 0 |
| 275.107775 | 275.107753 | 1443615 | 19 | 16 | 0 | 2 | 0 | 0 |
| 275.128899 | 275.128883 | 25642402 | 16 | 20 | 0 | 4 | 0 | 0 |
| 275.144089 | 275.144139 | 1653795 | 20 | 20 | 0 | 1 | 0 | 0 |
| 275.165297 | 275.165268 | 20029860 | 17 | 24 | 0 | 3 | 0 | 0 |
| 275.201684 | 275.201654 | 4376233 | 18 | 28 | 0 | 2 | 0 | 0 |
| 276.124133 | 276.124132 | 1266789 | 15 | 19 | 1 | 4 | 0 | 0 |
| 276.160353 | 276.160517 | 1170312 | 16 | 23 | 1 | 3 | 0 | 0 |
| 277.03541 | 277.035376 | 3816798 | 13 | 10 | 0 | 7 | 0 | 0 |
| 277.050678 | 277.050632 | 1728735 | 17 | 10 | 0 | 4 | 0 | 0 |
| 277.071778 | 277.071762 | 8670817 | 14 | 14 | 0 | 6 | 0 | 0 |
| 277.087031 | 277.087018 | 3152611 | 18 | 14 | 0 | 3 | 0 | 0 |
| 277.108157 | 277.108147 | 21708388 | 15 | 18 | 0 | 5 | 0 | 0 |
| 277.123422 | 277.123403 | 4902503 | 19 | 18 | 0 | 2 | 0 | 0 |
| 277.159784 | 277.159789 | 4116330 | 20 | 22 | 0 | 1 | 0 | 0 |
| 277.18094 | 277.180918 | 11135084 | 17 | 26 | 0 | 3 | 0 | 0 |
| 279.014605 | 279.014641 | 1409753 | 12 | 8 | 0 | 8 | 0 | 0 |
| 279.029892 | 279.029897 | 1740891 | 16 | 8 | 0 | 5 | 0 | 0 |
| 279.051035 | 279.051026 | 3274653 | 13 | 12 | 0 | 7 | 0 | 0 |
| 279.066277 | 279.066282 | 4459550 | 17 | 12 | 0 | 4 | 0 | 0 |
| 279.087418 | 279.087412 | 9861920 | 14 | 16 | 0 | 6 | 0 | 0 |
| 279.102674 | 279.102668 | 6479394 | 18 | 16 | 0 | 3 | 0 | 0 |
| 279.123788 | 279.123797 | 23894820 | 15 | 20 | 0 | 5 | 0 | 0 |
| 279.139059 | 279.139053 | 9728293 | 19 | 20 | 0 | 2 | 0 | 0 |
| 279.175461 | 279.175439 | 5971240 | 20 | 24 | 0 | 1 | 0 | 0 |
| 279.196545 | 279.196568 | 2769194 | 17 | 28 | 0 | 3 | 0 | 0 |
| 280.074178 | 280.074418 | 1428154 | 13 | 16 | 1 | 4 | 0 | 1 |
| 280.097885 | 280.097917 | 1139004 | 17 | 15 | 1 | 3 | 0 | 0 |
| 280.110542 | 280.110804 | 1301821 | 14 | 20 | 1 | 3 | 0 | 1 |
| 281.045545 | 281.045547 | 3522127 | 16 | 10 | 0 | 5 | 0 | 0 |
| 281.06669 | 281.066676 | 2574801 | 13 | 14 | 0 | 7 | 0 | 0 |
| 281.081954 | 281.081932 | 6015699 | 17 | 14 | 0 | 4 | 0 | 0 |
| 281.093267 | 281.093166 | 1281363 | 16 | 14 | 2 | 3 | 0 | 0 |
| 281.103051 | 281.103062 | 9021908 | 14 | 18 | 0 | 6 | 0 | 0 |
| 281.11835 | 281.118318 | 11777494 | 18 | 18 | 0 | 3 | 0 | 0 |
| 281.139456 | 281.139447 | 17062872 | 15 | 22 | 0 | 5 | 0 | 0 |
| 281.15472 | 281.154703 | 15502809 | 19 | 22 | 0 | 2 | 0 | 0 |
| 281.1911 | 281.191089 | 5962716 | 20 | 26 | 0 | 1 | 0 | 0 |
| 282.077093 | 282.077181 | 1213064 | 16 | 13 | 1 | 4 | 0 | 0 |
| 282.113582 | 282.113567 | 1409579 | 17 | 17 | 1 | 3 | 0 | 0 |
| 282.126199 | 282.126454 | 1537196 | 14 | 22 | 1 | 3 | 0 | 1 |
| 283.0248 | 283.024812 | 4523126 | 15 | 8 | 0 | 6 | 0 | 0 |
| 283.061206 | 283.061197 | 9068665 | 16 | 12 | 0 | 5 | 0 | 0 |
| 283.082291 | 283.082326 | 2880635 | 13 | 16 | 0 | 7 | 0 | 0 |
| 283.097601 | 283.097583 | 14682236 | 17 | 16 | 0 | 4 | 0 | 0 |
| 283.100465 | 283.100573 | 1392508 | 16 | 17 | 2 | 1 | 0 | 1 |
| 283.108827 | 283.108816 | 2672381 | 16 | 16 | 2 | 3 | 0 | 0 |
| 283.118703 | 283.118712 | 9636478 | 14 | 20 | 0 | 6 | 0 | 0 |
| 283.133988 | 283.133968 | 30183550 | 18 | 20 | 0 | 3 | 0 | 0 |
| 283.170369 | 283.170354 | 25653378 | 19 | 24 | 0 | 2 | 0 | 0 |
| 283.20675 | 283.206739 | 5928069 | 20 | 28 | 0 | 1 | 0 | 0 |
| 284.09274 | 284.092832 | 1653451 | 16 | 15 | 1 | 4 | 0 | 0 |
| 284.12921 | 284.129217 | 2189902 | 17 | 19 | 1 | 3 | 0 | 0 |
| 284.141852 | 284.142104 | 1790031 | 14 | 24 | 1 | 3 | 0 | 1 |
| 285.004081 | 285.004076 | 1299889 | 14 | 6 | 0 | 7 | 0 | 0 |
| 285.040459 | 285.040462 | 7488276 | 15 | 10 | 0 | 6 | 0 | 0 |
| 285.076834 | 285.076847 | 10823959 | 16 | 14 | 0 | 5 | 0 | 0 |
| 285.088053 | 285.08808 | 1272407 | 15 | 14 | 2 | 4 | 0 | 0 |
| 285.097971 | 285.097976 | 1889304 | 13 | 18 | 0 | 7 | 0 | 0 |
| 285.113237 | 285.113233 | 21524762 | 17 | 18 | 0 | 4 | 0 | 0 |
| 285.12447 | 285.124466 | 1914010 | 16 | 18 | 2 | 3 | 0 | 0 |
| 285.134345 | 285.134362 | 3761179 | 14 | 22 | 0 | 6 | 0 | 0 |
| 285.149614 | 285.149618 | 35729692 | 18 | 22 | 0 | 3 | 0 | 0 |
| 285.186008 | 285.186004 | 18867486 | 19 | 26 | 0 | 2 | 0 | 0 |
| 285.222411 | 285.222389 | 2307618 | 20 | 30 | 0 | 1 | 0 | 0 |
| 286.072084 | 286.072096 | 1130016 | 15 | 13 | 1 | 5 | 0 | 0 |
| 286.108507 | 286.108482 | 1796578 | 16 | 17 | 1 | 4 | 0 | 0 |
| 286.144883 | 286.144867 | 1262437 | 17 | 21 | 1 | 3 | 0 | 0 |
| 286.157482 | 286.157754 | 1416486 | 14 | 26 | 1 | 3 | 0 | 1 |
| 287.019756 | 287.019726 | 4729251 | 14 | 8 | 0 | 7 | 0 | 0 |
| 287.056136 | 287.056112 | 7857573 | 15 | 12 | 0 | 6 | 0 | 0 |
| 287.092511 | 287.092497 | 13674408 | 16 | 16 | 0 | 5 | 0 | 0 |
| 287.10376 | 287.103731 | 1676584 | 15 | 16 | 2 | 4 | 0 | 0 |
| 287.128896 | 287.128883 | 30347690 | 17 | 20 | 0 | 4 | 0 | 0 |
| 287.165279 | 287.165268 | 33624492 | 18 | 24 | 0 | 3 | 0 | 0 |
| 287.201669 | 287.201654 | 9293743 | 19 | 28 | 0 | 2 | 0 | 0 |
| 288.063971 | 288.064247 | 1578729 | 11 | 16 | 1 | 6 | 0 | 1 |
| 288.087828 | 288.087746 | 1659562 | 15 | 15 | 1 | 5 | 0 | 0 |
| 288.092708 | 288.092454 | 1229419 | 13 | 15 | 5 | 1 | 1 | 0 |
| 288.100346 | 288.100633 | 1659947 | 12 | 20 | 1 | 5 | 0 | 1 |
| 288.124117 | 288.124132 | 1692013 | 16 | 19 | 1 | 4 | 0 | 0 |
| 289.035394 | 289.035376 | 6093863 | 14 | 10 | 0 | 7 | 0 | 0 |
| 289.071761 | 289.071762 | 11008553 | 15 | 14 | 0 | 6 | 0 | 0 |
| 289.087061 | 289.087018 | 1825578 | 19 | 14 | 0 | 3 | 0 | 0 |
| 289.108156 | 289.108147 | 22551084 | 16 | 18 | 0 | 5 | 0 | 0 |
| 289.119387 | 289.119381 | 1866796 | 15 | 18 | 2 | 4 | 0 | 0 |
| 289.123422 | 289.123403 | 2941741 | 20 | 18 | 0 | 2 | 0 | 0 |
| 289.144531 | 289.144533 | 39154220 | 17 | 22 | 0 | 4 | 0 | 0 |
| 289.159821 | 289.159789 | 2490031 | 21 | 22 | 0 | 1 | 0 | 0 |
| 289.180923 | 289.180918 | 22528560 | 18 | 26 | 0 | 3 | 0 | 0 |
| 289.217302 | 289.217304 | 3674163 | 19 | 30 | 0 | 2 | 0 | 0 |
| 290.067043 | 290.067011 | 1247462 | 14 | 13 | 1 | 6 | 0 | 0 |
| 290.079634 | 290.079897 | 1599591 | 11 | 18 | 1 | 6 | 0 | 1 |
| 290.103409 | 290.103396 | 1399144 | 15 | 17 | 1 | 5 | 0 | 0 |
| 290.139832 | 290.139782 | 1397099 | 16 | 21 | 1 | 4 | 0 | 0 |
| 291.014603 | 291.014641 | 2269341 | 13 | 8 | 0 | 8 | 0 | 0 |
| 291.051015 | 291.051026 | 4480416 | 14 | 12 | 0 | 7 | 0 | 0 |
| 291.066283 | 291.066282 | 3118240 | 18 | 12 | 0 | 4 | 0 | 0 |
| 291.087414 | 291.087412 | 9197218 | 15 | 16 | 0 | 6 | 0 | 0 |
| 291.102672 | 291.102668 | 3609251 | 19 | 16 | 0 | 3 | 0 | 0 |
| 291.123797 | 291.123797 | 21294756 | 16 | 20 | 0 | 5 | 0 | 0 |
| 291.139069 | 291.139053 | 5393573 | 20 | 20 | 0 | 2 | 0 | 0 |
| 291.175454 | 291.175439 | 3755687 | 21 | 24 | 0 | 1 | 0 | 0 |
| 292.095294 | 292.095547 | 1179289 | 11 | 20 | 1 | 6 | 0 | 1 |
| 292.131692 | 292.131933 | 1330331 | 12 | 24 | 1 | 5 | 0 | 1 |
| 293.030296 | 293.030291 | 1791370 | 13 | 10 | 0 | 8 | 0 | 0 |
| 293.045568 | 293.045547 | 2560523 | 17 | 10 | 0 | 5 | 0 | 0 |
| 293.06671 | 293.066676 | 3284748 | 14 | 14 | 0 | 7 | 0 | 0 |
| 293.081935 | 293.081932 | 4349709 | 18 | 14 | 0 | 4 | 0 | 0 |
| 293.103065 | 293.103062 | 11788046 | 15 | 18 | 0 | 6 | 0 | 0 |
| 293.118357 | 293.118318 | 8099087 | 19 | 18 | 0 | 3 | 0 | 0 |
| 293.139446 | 293.139447 | 19369744 | 16 | 22 | 0 | 5 | 0 | 0 |
| 293.154713 | 293.154703 | 10507025 | 20 | 22 | 0 | 2 | 0 | 0 |
| 293.191091 | 293.191089 | 4765203 | 21 | 26 | 0 | 1 | 0 | 0 |
| 294.053458 | 294.053683 | 1334973 | 13 | 14 | 1 | 5 | 0 | 1 |
| 294.089812 | 294.090068 | 1269055 | 14 | 18 | 1 | 4 | 0 | 1 |
| 294.113519 | 294.113567 | 1142848 | 18 | 17 | 1 | 3 | 0 | 0 |
| 294.126215 | 294.126454 | 1145281 | 15 | 22 | 1 | 3 | 0 | 1 |
| 295.024839 | 295.024812 | 3341675 | 16 | 8 | 0 | 6 | 0 | 0 |
| 295.045894 | 295.045941 | 1558380 | 13 | 12 | 0 | 8 | 0 | 0 |
| 295.061212 | 295.061197 | 6572909 | 17 | 12 | 0 | 5 | 0 | 0 |
| 295.082347 | 295.082326 | 4388974 | 14 | 16 | 0 | 7 | 0 | 0 |
| 295.097595 | 295.097583 | 10562415 | 18 | 16 | 0 | 4 | 0 | 0 |
| 295.108925 | 295.108816 | 1438959 | 17 | 16 | 2 | 3 | 0 | 0 |
| 295.118722 | 295.118712 | 15007600 | 15 | 20 | 0 | 6 | 0 | 0 |
| 295.133971 | 295.133968 | 18874224 | 19 | 20 | 0 | 3 | 0 | 0 |
| 295.170358 | 295.170354 | 18632562 | 20 | 24 | 0 | 2 | 0 | 0 |
| 295.206733 | 295.206739 | 6130036 | 21 | 28 | 0 | 1 | 0 | 0 |
| 296.032663 | 296.032947 | 1242841 | 12 | 12 | 1 | 6 | 0 | 1 |
| 296.069063 | 296.069333 | 1623834 | 13 | 16 | 1 | 5 | 0 | 1 |
| 296.141824 | 296.142104 | 1852829 | 15 | 24 | 1 | 3 | 0 | 1 |
| 297.004148 | 297.004076 | 1167234 | 15 | 6 | 0 | 7 | 0 | 0 |
| 297.040442 | 297.040462 | 5641155 | 16 | 10 | 0 | 6 | 0 | 0 |
| 297.061607 | 297.061591 | 1238404 | 13 | 14 | 0 | 8 | 0 | 0 |
| 297.07684 | 297.076847 | 8857541 | 17 | 14 | 0 | 5 | 0 | 0 |
| 297.088068 | 297.08808 | 1564229 | 16 | 14 | 2 | 4 | 0 | 0 |
| 297.097989 | 297.097976 | 3925446 | 14 | 18 | 0 | 7 | 0 | 0 |
| 297.113214 | 297.113233 | 17474502 | 18 | 18 | 0 | 4 | 0 | 0 |
| 297.116596 | 297.116603 | 1215686 | 15 | 22 | 0 | 4 | 1 | 0 |
| 297.12446 | 297.124466 | 1204807 | 17 | 18 | 2 | 3 | 0 | 0 |
| 297.134385 | 297.134362 | 10776519 | 15 | 22 | 0 | 6 | 0 | 0 |
| 297.149606 | 297.149618 | 27679688 | 19 | 22 | 0 | 3 | 0 | 0 |
| 297.185999 | 297.186004 | 17697736 | 20 | 26 | 0 | 2 | 0 | 0 |
| 297.222368 | 297.222389 | 3516363 | 21 | 30 | 0 | 1 | 0 | 0 |
| 298.048307 | 298.048597 | 1349355 | 12 | 14 | 1 | 6 | 0 | 1 |
| 298.072075 | 298.072096 | 1281772 | 16 | 13 | 1 | 5 | 0 | 0 |
| 298.108487 | 298.108482 | 1198702 | 17 | 17 | 1 | 4 | 0 | 0 |
| 298.144854 | 298.144867 | 1247599 | 18 | 21 | 1 | 3 | 0 | 0 |
| 299.019749 | 299.019726 | 3017232 | 15 | 8 | 0 | 7 | 0 | 0 |
| 299.056125 | 299.056112 | 7625745 | 16 | 12 | 0 | 6 | 0 | 0 |
| 299.092491 | 299.092497 | 11334162 | 17 | 16 | 0 | 5 | 0 | 0 |
| 299.103753 | 299.103731 | 2359059 | 16 | 16 | 2 | 4 | 0 | 0 |
| 299.113657 | 299.113627 | 2457491 | 14 | 20 | 0 | 7 | 0 | 0 |
| 299.128883 | 299.128883 | 29037588 | 18 | 20 | 0 | 4 | 0 | 0 |
| 299.165279 | 299.165268 | 30260244 | 19 | 24 | 0 | 3 | 0 | 0 |
| 299.201667 | 299.201654 | 11719702 | 20 | 28 | 0 | 2 | 0 | 0 |
| 300.087721 | 300.087746 | 1323445 | 16 | 15 | 1 | 5 | 0 | 0 |
| 300.124131 | 300.124132 | 1574071 | 17 | 19 | 1 | 4 | 0 | 0 |
| 300.160581 | 300.160517 | 1311544 | 18 | 23 | 1 | 3 | 0 | 0 |
| 301.035378 | 301.035376 | 6952021 | 15 | 10 | 0 | 7 | 0 | 0 |
| 301.071775 | 301.071762 | 8575062 | 16 | 14 | 0 | 6 | 0 | 0 |
| 301.083041 | 301.082995 | 1449110 | 15 | 14 | 2 | 5 | 0 | 0 |
| 301.086972 | 301.087018 | 1449046 | 20 | 14 | 0 | 3 | 0 | 0 |
| 301.108161 | 301.108147 | 17975382 | 17 | 18 | 0 | 5 | 0 | 0 |
| 301.119392 | 301.119381 | 2710103 | 16 | 18 | 2 | 4 | 0 | 0 |
| 301.123315 | 301.123403 | 1332952 | 21 | 18 | 0 | 2 | 0 | 0 |
| 301.129329 | 301.129277 | 1133208 | 14 | 22 | 0 | 7 | 0 | 0 |
| 301.144544 | 301.144533 | 36185176 | 18 | 22 | 0 | 4 | 0 | 0 |
| 301.180929 | 301.180918 | 26680410 | 19 | 26 | 0 | 3 | 0 | 0 |
| 302.103422 | 302.103396 | 1248950 | 16 | 17 | 1 | 5 | 0 | 0 |
| 302.139788 | 302.139782 | 1505911 | 17 | 21 | 1 | 4 | 0 | 0 |
| 302.176125 | 302.176167 | 1252472 | 18 | 25 | 1 | 3 | 0 | 0 |
| 303.014628 | 303.014641 | 2375824 | 14 | 8 | 0 | 8 | 0 | 0 |
| 303.051014 | 303.051026 | 6279313 | 15 | 12 | 0 | 7 | 0 | 0 |
| 303.06632 | 303.066282 | 1830802 | 19 | 12 | 0 | 4 | 0 | 0 |
| 303.087406 | 303.087412 | 10380434 | 16 | 16 | 0 | 6 | 0 | 0 |
| 303.098652 | 303.098645 | 1201171 | 15 | 16 | 2 | 5 | 0 | 0 |
| 303.102667 | 303.102668 | 2506387 | 20 | 16 | 0 | 3 | 0 | 0 |
| 303.123782 | 303.123797 | 25668756 | 17 | 20 | 0 | 5 | 0 | 0 |
| 303.139034 | 303.139053 | 2890644 | 21 | 20 | 0 | 2 | 0 | 0 |
| 303.175439 | 303.175439 | 2254101 | 22 | 24 | 0 | 1 | 0 | 0 |
| 303.196556 | 303.196568 | 13331605 | 19 | 28 | 0 | 3 | 0 | 0 |
| 304.082551 | 304.082661 | 1289645 | 15 | 15 | 1 | 6 | 0 | 0 |
| 305.030283 | 305.030291 | 2402885 | 14 | 10 | 0 | 8 | 0 | 0 |
| 305.045533 | 305.045547 | 1889477 | 18 | 10 | 0 | 5 | 0 | 0 |
| 305.066681 | 305.066676 | 5073094 | 15 | 14 | 0 | 7 | 0 | 0 |
| 305.081932 | 305.081932 | 3132102 | 19 | 14 | 0 | 4 | 0 | 0 |
| 305.103048 | 305.103062 | 11168967 | 16 | 18 | 0 | 6 | 0 | 0 |
| 305.118328 | 305.118318 | 4824775 | 20 | 18 | 0 | 3 | 0 | 0 |
| 305.139439 | 305.139447 | 24794312 | 17 | 22 | 0 | 5 | 0 | 0 |
| 305.154691 | 305.154703 | 5978312 | 21 | 22 | 0 | 2 | 0 | 0 |
| 305.175845 | 305.175833 | 18801864 | 18 | 26 | 0 | 4 | 0 | 0 |
| 305.191053 | 305.191089 | 3149769 | 22 | 26 | 0 | 1 | 0 | 0 |
| 306.134678 | 306.134696 | 1223519 | 16 | 21 | 1 | 5 | 0 | 0 |
| 307.024837 | 307.024812 | 1723762 | 17 | 8 | 0 | 6 | 0 | 0 |
| 307.045929 | 307.045941 | 2033138 | 14 | 12 | 0 | 8 | 0 | 0 |
| 307.061184 | 307.061197 | 3994611 | 18 | 12 | 0 | 5 | 0 | 0 |
| 307.082318 | 307.082326 | 4302835 | 15 | 16 | 0 | 7 | 0 | 0 |
| 307.097584 | 307.097583 | 5724404 | 19 | 16 | 0 | 4 | 0 | 0 |
| 307.118706 | 307.118712 | 13547764 | 16 | 20 | 0 | 6 | 0 | 0 |
| 307.133951 | 307.133968 | 9380596 | 20 | 20 | 0 | 3 | 0 | 0 |
| 307.155101 | 307.155097 | 19208436 | 17 | 24 | 0 | 5 | 0 | 0 |
| 307.170351 | 307.170354 | 10395893 | 21 | 24 | 0 | 2 | 0 | 0 |
| 307.206729 | 307.206739 | 3870198 | 22 | 28 | 0 | 1 | 0 | 0 |
| 309.04046 | 309.040462 | 4426777 | 17 | 10 | 0 | 6 | 0 | 0 |
| 309.061593 | 309.061591 | 1364473 | 14 | 14 | 0 | 8 | 0 | 0 |
| 309.076835 | 309.076847 | 6922522 | 18 | 14 | 0 | 5 | 0 | 0 |
| 309.088122 | 309.08808 | 1147610 | 17 | 14 | 2 | 4 | 0 | 0 |
| 309.097976 | 309.097976 | 5244186 | 15 | 18 | 0 | 7 | 0 | 0 |
| 309.113215 | 309.113233 | 11548954 | 19 | 18 | 0 | 4 | 0 | 0 |
| 309.134367 | 309.134362 | 13308186 | 16 | 22 | 0 | 6 | 0 | 0 |
| 309.14961 | 309.149618 | 19282202 | 20 | 22 | 0 | 3 | 0 | 0 |
| 309.186007 | 309.186004 | 13900059 | 21 | 26 | 0 | 2 | 0 | 0 |
| 309.222381 | 309.222389 | 4472604 | 22 | 30 | 0 | 1 | 0 | 0 |
| 310.072106 | 310.072096 | 1186474 | 17 | 13 | 1 | 5 | 0 | 0 |
| 310.084732 | 310.084983 | 1688234 | 14 | 18 | 1 | 5 | 0 | 1 |
| 310.108491 | 310.108482 | 1139243 | 18 | 17 | 1 | 4 | 0 | 0 |
| 310.121086 | 310.121368 | 1931435 | 15 | 22 | 1 | 4 | 0 | 1 |
| 310.144764 | 310.144867 | 1184555 | 19 | 21 | 1 | 3 | 0 | 0 |
| 311.019732 | 311.019726 | 2808633 | 16 | 8 | 0 | 7 | 0 | 0 |
| 311.056104 | 311.056112 | 7382842 | 17 | 12 | 0 | 6 | 0 | 0 |
| 311.077189 | 311.077241 | 1282490 | 14 | 16 | 0 | 8 | 0 | 0 |
| 311.092487 | 311.092497 | 9900346 | 18 | 16 | 0 | 5 | 0 | 0 |
| 311.103718 | 311.103731 | 2543162 | 17 | 16 | 2 | 4 | 0 | 0 |
| 311.113626 | 311.113627 | 5081403 | 15 | 20 | 0 | 7 | 0 | 0 |
| 311.128868 | 311.128883 | 19658042 | 19 | 20 | 0 | 4 | 0 | 0 |
| 311.132186 | 311.132253 | 1421499 | 16 | 24 | 0 | 4 | 1 | 0 |
| 311.165275 | 311.165268 | 25617724 | 20 | 24 | 0 | 3 | 0 | 0 |
| 311.201664 | 311.201654 | 12942652 | 21 | 28 | 0 | 2 | 0 | 0 |
| 311.23806 | 311.238039 | 2329276 | 22 | 32 | 0 | 1 | 0 | 0 |
| 312.063979 | 312.064247 | 1589576 | 13 | 16 | 1 | 6 | 0 | 1 |
| 312.124198 | 312.124132 | 1340489 | 18 | 19 | 1 | 4 | 0 | 0 |
| 312.136713 | 312.137018 | 1144713 | 15 | 24 | 1 | 4 | 0 | 1 |
| 312.160556 | 312.160517 | 1347017 | 19 | 23 | 1 | 3 | 0 | 0 |
| 313.035376 | 313.035376 | 4908117 | 16 | 10 | 0 | 7 | 0 | 0 |
| 313.071773 | 313.071762 | 8010581 | 17 | 14 | 0 | 6 | 0 | 0 |
| 313.108131 | 313.108147 | 13578581 | 18 | 18 | 0 | 5 | 0 | 0 |
| 313.111481 | 313.111518 | 1174421 | 15 | 22 | 0 | 5 | 1 | 0 |
| 313.119418 | 313.119381 | 1869653 | 17 | 18 | 2 | 4 | 0 | 0 |
| 313.129299 | 313.129277 | 3405398 | 15 | 22 | 0 | 7 | 0 | 0 |
| 313.144518 | 313.144533 | 27643222 | 19 | 22 | 0 | 4 | 0 | 0 |
| 313.165652 | 313.165662 | 2465878 | 16 | 26 | 0 | 6 | 0 | 0 |
| 313.180902 | 313.180918 | 23592278 | 20 | 26 | 0 | 3 | 0 | 0 |
| 313.217298 | 313.217304 | 7032663 | 21 | 30 | 0 | 2 | 0 | 0 |
| 314.043211 | 314.043512 | 1176672 | 12 | 14 | 1 | 7 | 0 | 1 |
| 314.067015 | 314.067011 | 1323489 | 16 | 13 | 1 | 6 | 0 | 0 |
| 314.103438 | 314.103396 | 1282145 | 17 | 17 | 1 | 5 | 0 | 0 |
| 314.139738 | 314.139782 | 1268321 | 18 | 21 | 1 | 4 | 0 | 0 |
| 315.014632 | 315.014641 | 2193131 | 15 | 8 | 0 | 8 | 0 | 0 |
| 315.05103 | 315.051026 | 6086507 | 16 | 12 | 0 | 7 | 0 | 0 |
| 315.087403 | 315.087412 | 8332652 | 17 | 16 | 0 | 6 | 0 | 0 |
| 315.098706 | 315.098645 | 1403500 | 16 | 16 | 2 | 5 | 0 | 0 |
| 315.10267 | 315.102668 | 1421804 | 21 | 16 | 0 | 3 | 0 | 0 |
| 315.123814 | 315.123797 | 20479340 | 18 | 20 | 0 | 5 | 0 | 0 |
| 315.127191 | 315.127168 | 2021996 | 15 | 24 | 0 | 5 | 1 | 0 |
| 315.135048 | 315.135031 | 1254380 | 17 | 20 | 2 | 4 | 0 | 0 |
| 315.13912 | 315.139053 | 1240812 | 22 | 20 | 0 | 2 | 0 | 0 |
| 315.145019 | 315.144927 | 1861996 | 15 | 24 | 0 | 7 | 0 | 0 |
| 315.160177 | 315.160183 | 29597036 | 19 | 24 | 0 | 4 | 0 | 0 |
| 315.175407 | 315.175439 | 1336684 | 23 | 24 | 0 | 1 | 0 | 0 |
| 315.196566 | 315.196568 | 15605101 | 20 | 28 | 0 | 3 | 0 | 0 |
| 315.232943 | 315.232954 | 2882157 | 21 | 32 | 0 | 2 | 0 | 0 |
| 316.119073 | 316.119046 | 1423733 | 17 | 19 | 1 | 5 | 0 | 0 |
| 316.131667 | 316.131933 | 1385846 | 14 | 24 | 1 | 5 | 0 | 1 |
| 316.15546 | 316.155432 | 1448950 | 18 | 23 | 1 | 4 | 0 | 0 |
| 317.03033 | 317.030291 | 3204989 | 15 | 10 | 0 | 8 | 0 | 0 |
| 317.066684 | 317.066676 | 5682558 | 16 | 14 | 0 | 7 | 0 | 0 |
| 317.081943 | 317.081932 | 1912446 | 20 | 14 | 0 | 4 | 0 | 0 |
| 317.103046 | 317.103062 | 10680702 | 17 | 18 | 0 | 6 | 0 | 0 |
| 317.114366 | 317.114295 | 1696254 | 16 | 18 | 2 | 5 | 0 | 0 |
| 317.118359 | 317.118318 | 2538366 | 21 | 18 | 0 | 3 | 0 | 0 |
| 317.139446 | 317.139447 | 25343358 | 18 | 22 | 0 | 5 | 0 | 0 |
| 317.142755 | 317.142818 | 2197502 | 15 | 26 | 0 | 5 | 1 | 0 |
| 317.154728 | 317.154703 | 2581246 | 22 | 22 | 0 | 2 | 0 | 0 |
| 317.175855 | 317.175833 | 24241534 | 19 | 26 | 0 | 4 | 0 | 0 |
| 317.191092 | 317.191089 | 2065407 | 23 | 26 | 0 | 1 | 0 | 0 |
| 318.134756 | 318.134696 | 1425798 | 17 | 21 | 1 | 5 | 0 | 0 |
| 319.009536 | 319.009555 | 1181708 | 14 | 8 | 0 | 9 | 0 | 0 |
| 319.045946 | 319.045941 | 3899788 | 15 | 12 | 0 | 8 | 0 | 0 |
| 319.061166 | 319.061197 | 2937996 | 19 | 12 | 0 | 5 | 0 | 0 |
| 319.082338 | 319.082326 | 4878221 | 16 | 16 | 0 | 7 | 0 | 0 |
| 319.097593 | 319.097583 | 3909517 | 20 | 16 | 0 | 4 | 0 | 0 |
| 319.118722 | 319.118712 | 14028173 | 17 | 20 | 0 | 6 | 0 | 0 |
| 319.133971 | 319.133968 | 5965197 | 21 | 20 | 0 | 3 | 0 | 0 |
| 319.155095 | 319.155097 | 26127756 | 18 | 24 | 0 | 5 | 0 | 0 |
| 319.170392 | 319.170354 | 6349709 | 22 | 24 | 0 | 2 | 0 | 0 |
| 319.206727 | 319.206739 | 3285901 | 23 | 28 | 0 | 1 | 0 | 0 |
| 320.113946 | 320.113961 | 1192723 | 16 | 19 | 1 | 6 | 0 | 0 |
| 321.040429 | 321.040462 | 2847896 | 18 | 10 | 0 | 6 | 0 | 0 |
| 321.061557 | 321.061591 | 1697944 | 15 | 14 | 0 | 8 | 0 | 0 |
| 321.076842 | 321.076847 | 4303769 | 19 | 14 | 0 | 5 | 0 | 0 |
| 321.09797 | 321.097976 | 5083033 | 16 | 18 | 0 | 7 | 0 | 0 |
| 321.113234 | 321.113233 | 6194073 | 20 | 18 | 0 | 4 | 0 | 0 |
| 321.134353 | 321.134362 | 14975385 | 17 | 22 | 0 | 6 | 0 | 0 |
| 321.149621 | 321.149618 | 10173849 | 21 | 22 | 0 | 3 | 0 | 0 |
| 321.185999 | 321.186004 | 9360793 | 22 | 26 | 0 | 2 | 0 | 0 |
| 321.222369 | 321.222389 | 3273369 | 23 | 30 | 0 | 1 | 0 | 0 |
| 323.019712 | 323.019726 | 1946658 | 17 | 8 | 0 | 7 | 0 | 0 |
| 323.056135 | 323.056112 | 4579234 | 18 | 12 | 0 | 6 | 0 | 0 |
| 323.077176 | 323.077241 | 1547042 | 15 | 16 | 0 | 8 | 0 | 0 |
| 323.092483 | 323.092497 | 5835682 | 19 | 16 | 0 | 5 | 0 | 0 |
| 323.113634 | 323.113627 | 5972898 | 16 | 20 | 0 | 7 | 0 | 0 |
| 323.128871 | 323.128883 | 10308002 | 20 | 20 | 0 | 4 | 0 | 0 |
| 323.150013 | 323.150012 | 12012962 | 17 | 24 | 0 | 6 | 0 | 0 |
| 323.165279 | 323.165268 | 15067554 | 21 | 24 | 0 | 3 | 0 | 0 |
| 323.186381 | 323.186398 | 7397283 | 18 | 28 | 0 | 5 | 0 | 0 |
| 323.20164 | 323.201654 | 10486178 | 22 | 28 | 0 | 2 | 0 | 0 |
| 323.22278 | 323.222783 | 1266979 | 19 | 32 | 0 | 4 | 0 | 0 |
| 323.238047 | 323.238039 | 2312867 | 23 | 32 | 0 | 1 | 0 | 0 |
| 324.087707 | 324.087746 | 1542566 | 18 | 15 | 1 | 5 | 0 | 0 |
| 324.124108 | 324.124132 | 1263654 | 19 | 19 | 1 | 4 | 0 | 0 |
| 324.160609 | 324.160517 | 1278502 | 20 | 23 | 1 | 3 | 0 | 0 |
| 325.035388 | 325.035376 | 3856810 | 17 | 10 | 0 | 7 | 0 | 0 |
| 325.071776 | 325.071762 | 5921706 | 18 | 14 | 0 | 6 | 0 | 0 |
| 325.092866 | 325.092891 | 1689386 | 15 | 18 | 0 | 8 | 0 | 0 |
| 325.108154 | 325.108147 | 9354666 | 19 | 18 | 0 | 5 | 0 | 0 |
| 325.119457 | 325.119381 | 1193002 | 18 | 18 | 2 | 4 | 0 | 0 |
| 325.129278 | 325.129277 | 5911978 | 16 | 22 | 0 | 7 | 0 | 0 |
| 325.144521 | 325.144533 | 18503082 | 20 | 22 | 0 | 4 | 0 | 0 |
| 325.165665 | 325.165662 | 6436778 | 17 | 26 | 0 | 6 | 0 | 0 |
| 325.178421 | 325.178549 | 1165098 | 14 | 31 | 0 | 6 | 0 | 1 |
| 325.180931 | 325.180918 | 19039658 | 21 | 26 | 0 | 3 | 0 | 0 |
| 325.217311 | 325.217304 | 9281962 | 22 | 30 | 0 | 2 | 0 | 0 |
| 326.07961 | 326.079897 | 1260461 | 14 | 18 | 1 | 6 | 0 | 1 |
| 326.139729 | 326.139782 | 1268142 | 19 | 21 | 1 | 4 | 0 | 0 |
| 327.014644 | 327.014641 | 1893169 | 16 | 8 | 0 | 8 | 0 | 0 |
| 327.051036 | 327.051026 | 4919729 | 17 | 12 | 0 | 7 | 0 | 0 |
| 327.087393 | 327.087412 | 7914417 | 18 | 16 | 0 | 6 | 0 | 0 |
| 327.108545 | 327.108541 | 1248433 | 15 | 20 | 0 | 8 | 0 | 0 |
| 327.123787 | 327.123797 | 15943089 | 19 | 20 | 0 | 5 | 0 | 0 |
| 327.135009 | 327.135031 | 1269297 | 18 | 20 | 2 | 4 | 0 | 0 |
| 327.144946 | 327.144927 | 3006897 | 16 | 24 | 0 | 7 | 0 | 0 |
| 327.160194 | 327.160183 | 24905138 | 20 | 24 | 0 | 4 | 0 | 0 |
| 327.163555 | 327.163554 | 2384049 | 17 | 28 | 0 | 4 | 1 | 0 |
| 327.18132 | 327.181312 | 2180017 | 17 | 28 | 0 | 6 | 0 | 0 |
| 327.196579 | 327.196568 | 16810418 | 21 | 28 | 0 | 3 | 0 | 0 |
| 327.232937 | 327.232954 | 4816817 | 22 | 32 | 0 | 2 | 0 | 0 |
| 328.095371 | 328.095547 | 1145012 | 14 | 20 | 1 | 6 | 0 | 1 |
| 328.119039 | 328.119046 | 1386804 | 18 | 19 | 1 | 5 | 0 | 0 |
| 328.131658 | 328.131933 | 1467572 | 15 | 24 | 1 | 5 | 0 | 1 |
| 328.155386 | 328.155432 | 1593268 | 19 | 23 | 1 | 4 | 0 | 0 |
| 329.030331 | 329.030291 | 2992823 | 16 | 10 | 0 | 8 | 0 | 0 |
| 329.066669 | 329.066676 | 5459895 | 17 | 14 | 0 | 7 | 0 | 0 |
| 329.08191 | 329.081932 | 1159991 | 21 | 14 | 0 | 4 | 0 | 0 |
| 329.10306 | 329.103062 | 10446263 | 18 | 18 | 0 | 6 | 0 | 0 |
| 329.106392 | 329.106433 | 1347895 | 15 | 22 | 0 | 6 | 1 | 0 |
| 329.114303 | 329.114295 | 1637559 | 17 | 18 | 2 | 5 | 0 | 0 |
| 329.118302 | 329.118318 | 1654071 | 22 | 18 | 0 | 3 | 0 | 0 |
| 329.139455 | 329.139447 | 22719928 | 19 | 22 | 0 | 5 | 0 | 0 |
| 329.154706 | 329.154703 | 1544759 | 23 | 22 | 0 | 2 | 0 | 0 |
| 329.160601 | 329.160577 | 1515447 | 16 | 26 | 0 | 7 | 0 | 0 |
| 329.175827 | 329.175833 | 25343416 | 20 | 26 | 0 | 4 | 0 | 0 |
| 329.212199 | 329.212218 | 10031544 | 21 | 30 | 0 | 3 | 0 | 0 |
| 329.248626 | 329.248604 | 1812792 | 22 | 34 | 0 | 2 | 0 | 0 |
| 330.134695 | 330.134696 | 1360315 | 18 | 21 | 1 | 5 | 0 | 0 |
| 331.045935 | 331.045941 | 3312830 | 16 | 12 | 0 | 8 | 0 | 0 |
| 331.061191 | 331.061197 | 2229694 | 20 | 12 | 0 | 5 | 0 | 0 |
| 331.082302 | 331.082326 | 5654974 | 17 | 16 | 0 | 7 | 0 | 0 |
| 331.09757 | 331.097583 | 2093502 | 21 | 16 | 0 | 4 | 0 | 0 |
| 331.118692 | 331.118712 | 12614078 | 18 | 20 | 0 | 6 | 0 | 0 |
| 331.12205 | 331.122083 | 2004670 | 15 | 24 | 0 | 6 | 1 | 0 |
| 331.133972 | 331.133968 | 3103934 | 22 | 20 | 0 | 3 | 0 | 0 |
| 331.155105 | 331.155097 | 24214974 | 19 | 24 | 0 | 5 | 0 | 0 |
| 331.170341 | 331.170354 | 3058878 | 23 | 24 | 0 | 2 | 0 | 0 |
| 331.191491 | 331.191483 | 17094078 | 20 | 28 | 0 | 4 | 0 | 0 |
| 331.206802 | 331.206739 | 1717566 | 24 | 28 | 0 | 1 | 0 | 0 |
| 331.227853 | 331.227868 | 4181438 | 21 | 32 | 0 | 3 | 0 | 0 |
| 333.025199 | 333.025205 | 1144773 | 15 | 10 | 0 | 9 | 0 | 0 |
| 333.040422 | 333.040462 | 1530565 | 19 | 10 | 0 | 6 | 0 | 0 |
| 333.061553 | 333.061591 | 3649989 | 16 | 14 | 0 | 8 | 0 | 0 |
| 333.076813 | 333.076847 | 2623173 | 20 | 14 | 0 | 5 | 0 | 0 |
| 333.097992 | 333.097976 | 5104069 | 17 | 18 | 0 | 7 | 0 | 0 |
| 333.113214 | 333.113233 | 3751109 | 21 | 18 | 0 | 4 | 0 | 0 |
| 333.134362 | 333.134362 | 16086469 | 18 | 22 | 0 | 6 | 0 | 0 |
| 333.137778 | 333.137733 | 1300165 | 15 | 26 | 0 | 6 | 1 | 0 |
| 333.149623 | 333.149618 | 5212102 | 22 | 22 | 0 | 3 | 0 | 0 |
| 333.170736 | 333.170747 | 20043206 | 19 | 26 | 0 | 5 | 0 | 0 |
| 333.186017 | 333.186004 | 4768198 | 23 | 26 | 0 | 2 | 0 | 0 |
| 333.222354 | 333.222389 | 1763014 | 24 | 30 | 0 | 1 | 0 | 0 |
| 335.056137 | 335.056112 | 3141070 | 19 | 12 | 0 | 6 | 0 | 0 |
| 335.077249 | 335.077241 | 1868238 | 16 | 16 | 0 | 8 | 0 | 0 |
| 335.092498 | 335.092497 | 4312526 | 20 | 16 | 0 | 5 | 0 | 0 |
| 335.113649 | 335.113627 | 6536142 | 17 | 20 | 0 | 7 | 0 | 0 |
| 335.128882 | 335.128883 | 6945742 | 21 | 20 | 0 | 4 | 0 | 0 |
| 335.150003 | 335.150012 | 17038798 | 18 | 24 | 0 | 6 | 0 | 0 |
| 335.16528 | 335.165268 | 9379278 | 22 | 24 | 0 | 3 | 0 | 0 |
| 335.186387 | 335.186398 | 13235662 | 19 | 28 | 0 | 5 | 0 | 0 |
| 335.201635 | 335.201654 | 6653903 | 23 | 28 | 0 | 2 | 0 | 0 |
| 335.238045 | 335.238039 | 1603150 | 24 | 32 | 0 | 1 | 0 | 0 |
| 337.035355 | 337.035376 | 3532504 | 18 | 10 | 0 | 7 | 0 | 0 |
| 337.071796 | 337.071762 | 4934617 | 19 | 14 | 0 | 6 | 0 | 0 |
| 337.092896 | 337.092891 | 2583001 | 16 | 18 | 0 | 8 | 0 | 0 |
| 337.108129 | 337.108147 | 6030297 | 20 | 18 | 0 | 5 | 0 | 0 |
| 337.129279 | 337.129277 | 9070041 | 17 | 22 | 0 | 7 | 0 | 0 |
| 337.144531 | 337.144533 | 12098009 | 21 | 22 | 0 | 4 | 0 | 0 |
| 337.165654 | 337.165662 | 12882393 | 18 | 26 | 0 | 6 | 0 | 0 |
| 337.180919 | 337.180918 | 13954521 | 22 | 26 | 0 | 3 | 0 | 0 |
| 337.217299 | 337.217304 | 7860697 | 23 | 30 | 0 | 2 | 0 | 0 |
| 337.253593 | 337.253689 | 1236953 | 24 | 34 | 0 | 1 | 0 | 0 |
| 338.079648 | 338.079897 | 1142879 | 15 | 18 | 1 | 6 | 0 | 1 |
| 339.014651 | 339.014641 | 1725925 | 17 | 8 | 0 | 8 | 0 | 0 |
| 339.051011 | 339.051026 | 4660710 | 18 | 12 | 0 | 7 | 0 | 0 |
| 339.087388 | 339.087412 | 5898214 | 19 | 16 | 0 | 6 | 0 | 0 |
| 339.108505 | 339.108541 | 2413030 | 16 | 20 | 0 | 8 | 0 | 0 |
| 339.123804 | 339.123797 | 10348006 | 20 | 20 | 0 | 5 | 0 | 0 |
| 339.144902 | 339.144927 | 7453670 | 17 | 24 | 0 | 7 | 0 | 0 |
| 339.160172 | 339.160183 | 18605542 | 21 | 24 | 0 | 4 | 0 | 0 |
| 339.196563 | 339.196568 | 16348646 | 22 | 28 | 0 | 3 | 0 | 0 |
| 339.217725 | 339.217698 | 1265127 | 19 | 32 | 0 | 5 | 0 | 0 |
| 339.232975 | 339.232954 | 5741543 | 23 | 32 | 0 | 2 | 0 | 0 |
| 340.155461 | 340.155432 | 1288558 | 20 | 23 | 1 | 4 | 0 | 0 |
| 341.030273 | 341.030291 | 2678262 | 17 | 10 | 0 | 8 | 0 | 0 |
| 341.066668 | 341.066676 | 4534262 | 18 | 14 | 0 | 7 | 0 | 0 |
| 341.103068 | 341.103062 | 7812598 | 19 | 18 | 0 | 6 | 0 | 0 |
| 341.124175 | 341.124191 | 2084598 | 16 | 22 | 0 | 8 | 0 | 0 |
| 341.139445 | 341.139447 | 15828470 | 20 | 22 | 0 | 5 | 0 | 0 |
| 341.160593 | 341.160577 | 3633655 | 17 | 26 | 0 | 7 | 0 | 0 |
| 341.175822 | 341.175833 | 21011958 | 21 | 26 | 0 | 4 | 0 | 0 |
| 341.212227 | 341.212218 | 12362231 | 22 | 30 | 0 | 3 | 0 | 0 |
| 341.248637 | 341.248604 | 3108600 | 23 | 34 | 0 | 2 | 0 | 0 |
| 342.098365 | 342.098311 | 1159552 | 18 | 17 | 1 | 6 | 0 | 0 |
| 342.171099 | 342.171082 | 1272193 | 20 | 25 | 1 | 4 | 0 | 0 |
| 343.045938 | 343.045941 | 2995978 | 17 | 12 | 0 | 8 | 0 | 0 |
| 343.061124 | 343.061197 | 1167114 | 21 | 12 | 0 | 5 | 0 | 0 |
| 343.082357 | 343.082326 | 5308426 | 18 | 16 | 0 | 7 | 0 | 0 |
| 343.097596 | 343.097583 | 1425546 | 22 | 16 | 0 | 4 | 0 | 0 |
| 343.118704 | 343.118712 | 10230283 | 19 | 20 | 0 | 6 | 0 | 0 |
| 343.133875 | 343.133968 | 1609611 | 23 | 20 | 0 | 3 | 0 | 0 |
| 343.155112 | 343.155097 | 19377674 | 20 | 24 | 0 | 5 | 0 | 0 |
| 343.170295 | 343.170354 | 1245771 | 24 | 24 | 0 | 2 | 0 | 0 |
| 343.19148 | 343.191483 | 16530955 | 21 | 28 | 0 | 4 | 0 | 0 |
| 343.206759 | 343.206739 | 1153164 | 25 | 28 | 0 | 1 | 0 | 0 |
| 343.227848 | 343.227868 | 6645260 | 22 | 32 | 0 | 3 | 0 | 0 |
| 345.025254 | 345.025205 | 1294114 | 16 | 10 | 0 | 9 | 0 | 0 |
| 345.061599 | 345.061591 | 3108131 | 17 | 14 | 0 | 8 | 0 | 0 |
| 345.076824 | 345.076847 | 1705251 | 21 | 14 | 0 | 5 | 0 | 0 |
| 345.097974 | 345.097976 | 5688867 | 18 | 18 | 0 | 7 | 0 | 0 |
| 345.113244 | 345.113233 | 1681827 | 22 | 18 | 0 | 4 | 0 | 0 |
| 345.134364 | 345.134362 | 13379107 | 19 | 22 | 0 | 6 | 0 | 0 |
| 345.149628 | 345.149618 | 2791460 | 23 | 22 | 0 | 3 | 0 | 0 |
| 345.170755 | 345.170747 | 19529252 | 20 | 26 | 0 | 5 | 0 | 0 |
| 345.185988 | 345.186004 | 2541604 | 24 | 26 | 0 | 2 | 0 | 0 |
| 345.207136 | 345.207133 | 10469924 | 21 | 30 | 0 | 4 | 0 | 0 |
| 345.222404 | 345.222389 | 1367333 | 25 | 30 | 0 | 1 | 0 | 0 |
| 345.243519 | 345.243519 | 2011941 | 22 | 34 | 0 | 3 | 0 | 0 |
| 347.040793 | 347.040856 | 1695296 | 16 | 12 | 0 | 9 | 0 | 0 |
| 347.056065 | 347.056112 | 2186304 | 20 | 12 | 0 | 6 | 0 | 0 |
| 347.077254 | 347.077241 | 2787904 | 17 | 16 | 0 | 8 | 0 | 0 |
| 347.092548 | 347.092497 | 2699329 | 21 | 16 | 0 | 5 | 0 | 0 |
| 347.113617 | 347.113627 | 6921793 | 18 | 20 | 0 | 7 | 0 | 0 |
| 347.128867 | 347.128883 | 4479041 | 22 | 20 | 0 | 4 | 0 | 0 |
| 347.150013 | 347.150012 | 16649794 | 19 | 24 | 0 | 6 | 0 | 0 |
| 347.165262 | 347.165268 | 4975682 | 23 | 24 | 0 | 3 | 0 | 0 |
| 347.186397 | 347.186398 | 15945282 | 20 | 28 | 0 | 5 | 0 | 0 |
| 347.20165 | 347.201654 | 3860034 | 24 | 28 | 0 | 2 | 0 | 0 |
| 347.222792 | 347.222783 | 4480579 | 21 | 32 | 0 | 4 | 0 | 0 |
| 347.238055 | 347.238039 | 1271235 | 25 | 32 | 0 | 1 | 0 | 0 |
| 349.035404 | 349.035376 | 2455651 | 19 | 10 | 0 | 7 | 0 | 0 |
| 349.056446 | 349.056506 | 1422947 | 16 | 14 | 0 | 9 | 0 | 0 |
| 349.071782 | 349.071762 | 3642980 | 20 | 14 | 0 | 6 | 0 | 0 |
| 349.092903 | 349.092891 | 2792804 | 17 | 18 | 0 | 8 | 0 | 0 |
| 349.108138 | 349.108147 | 4558948 | 21 | 18 | 0 | 5 | 0 | 0 |
| 349.129294 | 349.129277 | 9724517 | 18 | 22 | 0 | 7 | 0 | 0 |
| 349.144543 | 349.144533 | 6600293 | 22 | 22 | 0 | 4 | 0 | 0 |
| 349.165649 | 349.165662 | 16610917 | 19 | 26 | 0 | 6 | 0 | 0 |
| 349.180933 | 349.180918 | 8034918 | 23 | 26 | 0 | 3 | 0 | 0 |
| 349.202063 | 349.202048 | 10367590 | 20 | 30 | 0 | 5 | 0 | 0 |
| 349.217311 | 349.217304 | 5411431 | 24 | 30 | 0 | 2 | 0 | 0 |
| 349.238416 | 349.238433 | 1638503 | 21 | 34 | 0 | 4 | 0 | 0 |
| 349.253654 | 349.253689 | 1406823 | 25 | 34 | 0 | 1 | 0 | 0 |
| 351.01473 | 351.014641 | 1458956 | 18 | 8 | 0 | 8 | 0 | 0 |
| 351.051036 | 351.051026 | 4080781 | 19 | 12 | 0 | 7 | 0 | 0 |
| 351.087425 | 351.087412 | 4924558 | 20 | 16 | 0 | 6 | 0 | 0 |
| 351.108512 | 351.108541 | 3305358 | 17 | 20 | 0 | 8 | 0 | 0 |
| 351.123776 | 351.123797 | 6232206 | 21 | 20 | 0 | 5 | 0 | 0 |
| 351.144925 | 351.144927 | 11110031 | 18 | 24 | 0 | 7 | 0 | 0 |
| 351.160196 | 351.160183 | 11149967 | 22 | 24 | 0 | 4 | 0 | 0 |
| 351.181305 | 351.181312 | 11778703 | 19 | 28 | 0 | 6 | 0 | 0 |
| 351.196557 | 351.196568 | 11604624 | 23 | 28 | 0 | 3 | 0 | 0 |
| 351.232973 | 351.232954 | 5074065 | 24 | 32 | 0 | 2 | 0 | 0 |
| 351.290652 | 351.290469 | 1211922 | 22 | 40 | 0 | 3 | 0 | 0 |
| 353.03031 | 353.030291 | 3308220 | 18 | 10 | 0 | 8 | 0 | 0 |
| 353.066673 | 353.066676 | 3918525 | 19 | 14 | 0 | 7 | 0 | 0 |
| 353.103042 | 353.103062 | 5954750 | 20 | 18 | 0 | 6 | 0 | 0 |
| 353.124191 | 353.124191 | 3965119 | 17 | 22 | 0 | 8 | 0 | 0 |
| 353.139461 | 353.139447 | 11202239 | 21 | 22 | 0 | 5 | 0 | 0 |
| 353.160576 | 353.160577 | 7626432 | 18 | 26 | 0 | 7 | 0 | 0 |
| 353.17582 | 353.175833 | 15974080 | 22 | 26 | 0 | 4 | 0 | 0 |
| 353.196951 | 353.196962 | 4714177 | 19 | 30 | 0 | 6 | 0 | 0 |
| 353.212231 | 353.212218 | 12053185 | 23 | 30 | 0 | 3 | 0 | 0 |
| 353.248611 | 353.248604 | 4011202 | 24 | 34 | 0 | 2 | 0 | 0 |
| 355.045942 | 355.045941 | 3399924 | 18 | 12 | 0 | 8 | 0 | 0 |
| 355.082335 | 355.082326 | 4489461 | 19 | 16 | 0 | 7 | 0 | 0 |
| 355.11871 | 355.118712 | 8305399 | 20 | 20 | 0 | 6 | 0 | 0 |
| 355.13404 | 355.133968 | 1186935 | 24 | 20 | 0 | 3 | 0 | 0 |
| 355.139855 | 355.139841 | 2879223 | 17 | 24 | 0 | 8 | 0 | 0 |
| 355.155086 | 355.155097 | 15798007 | 21 | 24 | 0 | 5 | 0 | 0 |
| 355.176211 | 355.176227 | 3018232 | 18 | 28 | 0 | 7 | 0 | 0 |
| 355.191482 | 355.191483 | 16295672 | 22 | 28 | 0 | 4 | 0 | 0 |
| 355.212672 | 355.212612 | 1325561 | 19 | 32 | 0 | 6 | 0 | 0 |
| 355.227869 | 355.227868 | 8767226 | 23 | 32 | 0 | 3 | 0 | 0 |
| 355.264226 | 355.264254 | 1807867 | 24 | 36 | 0 | 2 | 0 | 0 |
| 356.150291 | 356.150346 | 1179414 | 20 | 23 | 1 | 5 | 0 | 0 |
| 357.025136 | 357.025205 | 1683506 | 17 | 10 | 0 | 9 | 0 | 0 |
| 357.061572 | 357.061591 | 3346228 | 18 | 14 | 0 | 8 | 0 | 0 |
| 357.097979 | 357.097976 | 5265205 | 19 | 18 | 0 | 7 | 0 | 0 |
| 357.134343 | 357.134362 | 11938614 | 20 | 22 | 0 | 6 | 0 | 0 |
| 357.149669 | 357.149618 | 1622455 | 24 | 22 | 0 | 3 | 0 | 0 |
| 357.170748 | 357.170747 | 17590072 | 21 | 26 | 0 | 5 | 0 | 0 |
| 357.185969 | 357.186004 | 1365432 | 25 | 26 | 0 | 2 | 0 | 0 |
| 357.207119 | 357.207133 | 12091192 | 22 | 30 | 0 | 4 | 0 | 0 |
| 357.243519 | 357.243519 | 4542778 | 23 | 34 | 0 | 3 | 0 | 0 |
| 359.040832 | 359.040856 | 1513081 | 17 | 12 | 0 | 9 | 0 | 0 |
| 359.056105 | 359.056112 | 1537658 | 21 | 12 | 0 | 6 | 0 | 0 |
| 359.077269 | 359.077241 | 2895994 | 18 | 16 | 0 | 8 | 0 | 0 |
| 359.092511 | 359.092497 | 1671803 | 22 | 16 | 0 | 5 | 0 | 0 |
| 359.113645 | 359.113627 | 6992764 | 19 | 20 | 0 | 7 | 0 | 0 |
| 359.128895 | 359.128883 | 2025340 | 23 | 20 | 0 | 4 | 0 | 0 |
| 359.150006 | 359.150012 | 15181693 | 20 | 24 | 0 | 6 | 0 | 0 |
| 359.165278 | 359.165268 | 2381950 | 24 | 24 | 0 | 3 | 0 | 0 |
| 359.186413 | 359.186398 | 15589246 | 21 | 28 | 0 | 5 | 0 | 0 |
| 359.201627 | 359.201654 | 2035071 | 25 | 28 | 0 | 2 | 0 | 0 |
| 359.222772 | 359.222783 | 6632320 | 22 | 32 | 0 | 4 | 0 | 0 |
| 359.259191 | 359.259169 | 1861761 | 23 | 36 | 0 | 3 | 0 | 0 |
| 361.035362 | 361.035376 | 1607621 | 20 | 10 | 0 | 7 | 0 | 0 |
| 361.056596 | 361.056506 | 1501894 | 17 | 14 | 0 | 9 | 0 | 0 |
| 361.071751 | 361.071762 | 2374599 | 21 | 14 | 0 | 6 | 0 | 0 |
| 361.09285 | 361.092891 | 3477448 | 18 | 18 | 0 | 8 | 0 | 0 |
| 361.10818 | 361.108147 | 2889672 | 22 | 18 | 0 | 5 | 0 | 0 |
| 361.12928 | 361.129277 | 8920009 | 19 | 22 | 0 | 7 | 0 | 0 |
| 361.144517 | 361.144533 | 4363210 | 23 | 22 | 0 | 4 | 0 | 0 |
| 361.165651 | 361.165662 | 17571786 | 20 | 26 | 0 | 6 | 0 | 0 |
| 361.180916 | 361.180918 | 5312459 | 24 | 26 | 0 | 3 | 0 | 0 |
| 361.202046 | 361.202048 | 10373068 | 21 | 30 | 0 | 5 | 0 | 0 |
| 361.217282 | 361.217304 | 3657677 | 25 | 30 | 0 | 2 | 0 | 0 |
| 361.238464 | 361.238433 | 2633934 | 22 | 34 | 0 | 4 | 0 | 0 |
| 361.253661 | 361.253689 | 1205966 | 26 | 34 | 0 | 1 | 0 | 0 |
| 363.051023 | 363.051026 | 2875161 | 20 | 12 | 0 | 7 | 0 | 0 |
| 363.07216 | 363.072156 | 1583642 | 17 | 16 | 0 | 9 | 0 | 0 |
| 363.087412 | 363.087412 | 3549723 | 21 | 16 | 0 | 6 | 0 | 0 |
| 363.108552 | 363.108541 | 3823132 | 18 | 20 | 0 | 8 | 0 | 0 |
| 363.123786 | 363.123797 | 4752925 | 22 | 20 | 0 | 5 | 0 | 0 |
| 363.144933 | 363.144927 | 12924957 | 19 | 24 | 0 | 7 | 0 | 0 |
| 363.160178 | 363.160183 | 6987806 | 23 | 24 | 0 | 4 | 0 | 0 |
| 363.181307 | 363.181312 | 15972383 | 20 | 28 | 0 | 6 | 0 | 0 |
| 363.196554 | 363.196568 | 7503904 | 24 | 28 | 0 | 3 | 0 | 0 |
| 363.217691 | 363.217698 | 4780577 | 21 | 32 | 0 | 5 | 0 | 0 |
| 363.232983 | 363.232954 | 4240417 | 25 | 32 | 0 | 2 | 0 | 0 |
| 365.030318 | 365.030291 | 2474354 | 19 | 10 | 0 | 8 | 0 | 0 |
| 365.066726 | 365.066676 | 3776115 | 20 | 14 | 0 | 7 | 0 | 0 |
| 365.087821 | 365.087806 | 1889140 | 17 | 18 | 0 | 9 | 0 | 0 |
| 365.103022 | 365.103062 | 4690037 | 21 | 18 | 0 | 6 | 0 | 0 |
| 365.124206 | 365.124191 | 6060150 | 18 | 22 | 0 | 8 | 0 | 0 |
| 365.139458 | 365.139447 | 6759543 | 22 | 22 | 0 | 5 | 0 | 0 |
| 365.160571 | 365.160577 | 12718200 | 19 | 26 | 0 | 7 | 0 | 0 |
| 365.175814 | 365.175833 | 11195512 | 23 | 26 | 0 | 4 | 0 | 0 |
| 365.196983 | 365.196962 | 10384505 | 20 | 30 | 0 | 6 | 0 | 0 |
| 365.21223 | 365.212218 | 8475770 | 24 | 30 | 0 | 3 | 0 | 0 |
| 365.233397 | 365.233348 | 1622011 | 21 | 34 | 0 | 5 | 0 | 0 |
| 365.248588 | 365.248604 | 4018300 | 25 | 34 | 0 | 2 | 0 | 0 |
| 367.045969 | 367.045941 | 3206354 | 19 | 12 | 0 | 8 | 0 | 0 |
| 367.082346 | 367.082326 | 4228307 | 20 | 16 | 0 | 7 | 0 | 0 |
| 367.10348 | 367.103456 | 1538004 | 17 | 20 | 0 | 9 | 0 | 0 |
| 367.11869 | 367.118712 | 6100181 | 21 | 20 | 0 | 6 | 0 | 0 |
| 367.139825 | 367.139841 | 5855446 | 18 | 24 | 0 | 8 | 0 | 0 |
| 367.155116 | 367.155097 | 11280599 | 22 | 24 | 0 | 5 | 0 | 0 |
| 367.176223 | 367.176227 | 7423192 | 19 | 28 | 0 | 7 | 0 | 0 |
| 367.191463 | 367.191483 | 13914329 | 23 | 28 | 0 | 4 | 0 | 0 |
| 367.227867 | 367.227868 | 8764634 | 24 | 32 | 0 | 3 | 0 | 0 |
| 367.264266 | 367.264254 | 2396380 | 25 | 36 | 0 | 2 | 0 | 0 |
| 368.1044 | 368.104065 | 1314566 | 22 | 15 | 3 | 3 | 0 | 0 |
| 368.133777 | 368.134058 | 2270728 | 11 | 24 | 5 | 7 | 0 | 1 |
| 368.150405 | 368.150346 | 1496840 | 21 | 23 | 1 | 5 | 0 | 0 |
| 369.025189 | 369.025205 | 1684021 | 18 | 10 | 0 | 9 | 0 | 0 |
| 369.061615 | 369.061591 | 3142967 | 19 | 14 | 0 | 8 | 0 | 0 |
| 369.097965 | 369.097976 | 4950841 | 20 | 18 | 0 | 7 | 0 | 0 |
| 369.119135 | 369.119106 | 1516090 | 17 | 22 | 0 | 9 | 0 | 0 |
| 369.134373 | 369.134362 | 8883515 | 21 | 22 | 0 | 6 | 0 | 0 |
| 369.155471 | 369.155491 | 3591996 | 18 | 26 | 0 | 8 | 0 | 0 |
| 369.170735 | 369.170747 | 15190332 | 22 | 26 | 0 | 5 | 0 | 0 |
| 369.191842 | 369.191877 | 2556734 | 19 | 30 | 0 | 7 | 0 | 0 |
| 369.207147 | 369.207133 | 14356798 | 23 | 30 | 0 | 4 | 0 | 0 |
| 369.228272 | 369.228262 | 1204032 | 20 | 34 | 0 | 6 | 0 | 0 |
| 369.243506 | 369.243519 | 5822785 | 24 | 34 | 0 | 3 | 0 | 0 |
| 371.040861 | 371.040856 | 2122144 | 18 | 12 | 0 | 9 | 0 | 0 |
| 371.077236 | 371.077241 | 3484834 | 19 | 16 | 0 | 8 | 0 | 0 |
| 371.092556 | 371.092497 | 1232290 | 23 | 16 | 0 | 5 | 0 | 0 |
| 371.113629 | 371.113627 | 5732772 | 20 | 20 | 0 | 7 | 0 | 0 |
| 371.128986 | 371.128883 | 1350052 | 24 | 20 | 0 | 4 | 0 | 0 |
| 371.150025 | 371.150012 | 13343141 | 21 | 24 | 0 | 6 | 0 | 0 |
| 371.165257 | 371.165268 | 1445286 | 25 | 24 | 0 | 3 | 0 | 0 |
| 371.171145 | 371.171141 | 1451687 | 18 | 28 | 0 | 8 | 0 | 0 |
| 371.186388 | 371.186398 | 16712103 | 22 | 28 | 0 | 5 | 0 | 0 |
| 371.201613 | 371.201654 | 1509800 | 26 | 28 | 0 | 2 | 0 | 0 |
| 371.222784 | 371.222783 | 10148265 | 23 | 32 | 0 | 4 | 0 | 0 |
| 371.259165 | 371.259169 | 3018924 | 24 | 36 | 0 | 3 | 0 | 0 |
| 373.035254 | 373.035376 | 1193870 | 21 | 10 | 0 | 7 | 0 | 0 |
| 373.056494 | 373.056506 | 1861903 | 18 | 14 | 0 | 9 | 0 | 0 |
| 373.071758 | 373.071762 | 2219024 | 22 | 14 | 0 | 6 | 0 | 0 |
| 373.092877 | 373.092891 | 3659281 | 19 | 18 | 0 | 8 | 0 | 0 |
| 373.108142 | 373.108147 | 2354450 | 23 | 18 | 0 | 5 | 0 | 0 |
| 373.129288 | 373.129277 | 8683027 | 20 | 22 | 0 | 7 | 0 | 0 |
| 373.144536 | 373.144533 | 2536980 | 24 | 22 | 0 | 4 | 0 | 0 |
| 373.16565 | 373.165662 | 14461461 | 21 | 26 | 0 | 6 | 0 | 0 |
| 373.180886 | 373.180918 | 2700054 | 25 | 26 | 0 | 3 | 0 | 0 |
| 373.202074 | 373.202048 | 12696087 | 22 | 30 | 0 | 5 | 0 | 0 |
| 373.21728 | 373.217304 | 1884440 | 26 | 30 | 0 | 2 | 0 | 0 |
| 373.238413 | 373.238433 | 5339162 | 23 | 34 | 0 | 4 | 0 | 0 |
| 375.051059 | 375.051026 | 2226818 | 21 | 12 | 0 | 7 | 0 | 0 |
| 375.072168 | 375.072156 | 2090115 | 18 | 16 | 0 | 9 | 0 | 0 |
| 375.08743 | 375.087412 | 2795652 | 22 | 16 | 0 | 6 | 0 | 0 |
| 375.108553 | 375.108541 | 3796102 | 19 | 20 | 0 | 8 | 0 | 0 |
| 375.123816 | 375.123797 | 2778758 | 23 | 20 | 0 | 5 | 0 | 0 |
| 375.144928 | 375.144927 | 11132552 | 20 | 24 | 0 | 7 | 0 | 0 |
| 375.160196 | 375.160183 | 4178569 | 24 | 24 | 0 | 4 | 0 | 0 |
| 375.18131 | 375.181312 | 14434954 | 21 | 28 | 0 | 6 | 0 | 0 |
| 375.196588 | 375.196568 | 4654731 | 25 | 28 | 0 | 3 | 0 | 0 |
| 375.2177 | 375.217698 | 7134860 | 22 | 32 | 0 | 5 | 0 | 0 |
| 375.232938 | 375.232954 | 2595981 | 26 | 32 | 0 | 2 | 0 | 0 |
| 375.254066 | 375.254083 | 2044302 | 23 | 36 | 0 | 4 | 0 | 0 |
| 377.030301 | 377.030291 | 1934584 | 20 | 10 | 0 | 8 | 0 | 0 |
| 377.066631 | 377.066676 | 3006459 | 21 | 14 | 0 | 7 | 0 | 0 |
| 377.087901 | 377.087806 | 1816316 | 18 | 18 | 0 | 9 | 0 | 0 |
| 377.10309 | 377.103062 | 3047677 | 22 | 18 | 0 | 6 | 0 | 0 |
| 377.124176 | 377.124191 | 5943038 | 19 | 22 | 0 | 8 | 0 | 0 |
| 377.139462 | 377.139447 | 4690175 | 23 | 22 | 0 | 5 | 0 | 0 |
| 377.160585 | 377.160577 | 14363392 | 20 | 26 | 0 | 7 | 0 | 0 |
| 377.175854 | 377.175833 | 6139649 | 24 | 26 | 0 | 4 | 0 | 0 |
| 377.196959 | 377.196962 | 9814786 | 21 | 30 | 0 | 6 | 0 | 0 |
| 377.212212 | 377.212218 | 5704452 | 25 | 30 | 0 | 3 | 0 | 0 |
| 377.23336 | 377.233348 | 3299845 | 22 | 34 | 0 | 5 | 0 | 0 |
| 377.248654 | 377.248604 | 2932998 | 26 | 34 | 0 | 2 | 0 | 0 |
| 379.045932 | 379.045941 | 2937461 | 20 | 12 | 0 | 8 | 0 | 0 |
| 379.082302 | 379.082326 | 3597175 | 21 | 16 | 0 | 7 | 0 | 0 |
| 379.103477 | 379.103456 | 2378360 | 18 | 20 | 0 | 9 | 0 | 0 |
| 379.118704 | 379.118712 | 4736889 | 22 | 20 | 0 | 6 | 0 | 0 |
| 379.139834 | 379.139841 | 8443771 | 19 | 24 | 0 | 8 | 0 | 0 |
| 379.155116 | 379.155097 | 8121212 | 23 | 24 | 0 | 5 | 0 | 0 |
| 379.176224 | 379.176227 | 12202877 | 20 | 28 | 0 | 7 | 0 | 0 |
| 379.191468 | 379.191483 | 9921406 | 24 | 28 | 0 | 4 | 0 | 0 |
| 379.212609 | 379.212612 | 5136767 | 21 | 32 | 0 | 6 | 0 | 0 |
| 379.22788 | 379.227868 | 6448000 | 25 | 32 | 0 | 3 | 0 | 0 |
| 379.264269 | 379.264254 | 2720898 | 26 | 36 | 0 | 2 | 0 | 0 |
| 381.025219 | 381.025205 | 1892082 | 19 | 10 | 0 | 9 | 0 | 0 |
| 381.061577 | 381.061591 | 3572213 | 20 | 14 | 0 | 8 | 0 | 0 |
| 381.097987 | 381.097976 | 3995127 | 21 | 18 | 0 | 7 | 0 | 0 |
| 381.119058 | 381.119106 | 2812152 | 18 | 22 | 0 | 9 | 0 | 0 |
| 381.134359 | 381.134362 | 6706170 | 22 | 22 | 0 | 6 | 0 | 0 |
| 381.155499 | 381.155491 | 8301563 | 19 | 26 | 0 | 8 | 0 | 0 |
| 381.17075 | 381.170747 | 10799100 | 23 | 26 | 0 | 5 | 0 | 0 |
| 381.191883 | 381.191877 | 7514109 | 20 | 30 | 0 | 7 | 0 | 0 |
| 381.207128 | 381.207133 | 11660286 | 24 | 30 | 0 | 4 | 0 | 0 |
| 381.228223 | 381.228262 | 1575679 | 21 | 34 | 0 | 6 | 0 | 0 |
| 381.243528 | 381.243519 | 6659073 | 25 | 34 | 0 | 3 | 0 | 0 |
| 381.27989 | 381.279904 | 1710339 | 26 | 38 | 0 | 2 | 0 | 0 |
| 383.040875 | 383.040856 | 2215542 | 19 | 12 | 0 | 9 | 0 | 0 |
| 383.077248 | 383.077241 | 4001912 | 20 | 16 | 0 | 8 | 0 | 0 |
| 383.113623 | 383.113627 | 5100155 | 21 | 20 | 0 | 7 | 0 | 0 |
| 383.134752 | 383.134756 | 2434940 | 18 | 24 | 0 | 9 | 0 | 0 |
| 383.150027 | 383.150012 | 10705021 | 22 | 24 | 0 | 6 | 0 | 0 |
| 383.165335 | 383.165268 | 1316094 | 26 | 24 | 0 | 3 | 0 | 0 |
| 383.171152 | 383.171141 | 3603070 | 19 | 28 | 0 | 8 | 0 | 0 |
| 383.186398 | 383.186398 | 14498943 | 23 | 28 | 0 | 5 | 0 | 0 |
| 383.207532 | 383.207527 | 2599553 | 20 | 32 | 0 | 7 | 0 | 0 |
| 383.222806 | 383.222783 | 11609218 | 24 | 32 | 0 | 4 | 0 | 0 |
| 383.259174 | 383.259169 | 4434564 | 25 | 36 | 0 | 3 | 0 | 0 |
| 385.056584 | 385.056506 | 2087164 | 19 | 14 | 0 | 9 | 0 | 0 |
| 385.071756 | 385.071762 | 1439741 | 23 | 14 | 0 | 6 | 0 | 0 |
| 385.092921 | 385.092891 | 4015359 | 20 | 18 | 0 | 8 | 0 | 0 |
| 385.108207 | 385.108147 | 1403776 | 24 | 18 | 0 | 5 | 0 | 0 |
| 385.129269 | 385.129277 | 7388417 | 21 | 22 | 0 | 7 | 0 | 0 |
| 385.144545 | 385.144533 | 1686530 | 25 | 22 | 0 | 4 | 0 | 0 |
| 385.16568 | 385.165662 | 14073091 | 22 | 26 | 0 | 6 | 0 | 0 |
| 385.18087 | 385.180918 | 1900036 | 26 | 26 | 0 | 3 | 0 | 0 |
| 385.186742 | 385.186791 | 1993221 | 19 | 30 | 0 | 8 | 0 | 0 |
| 385.202045 | 385.202048 | 15506694 | 23 | 30 | 0 | 5 | 0 | 0 |
| 385.21729 | 385.217304 | 1665287 | 27 | 30 | 0 | 2 | 0 | 0 |
| 385.238449 | 385.238433 | 8710408 | 24 | 34 | 0 | 4 | 0 | 0 |
| 385.274761 | 385.274819 | 1876235 | 25 | 38 | 0 | 3 | 0 | 0 |
| 387.035672 | 387.03577 | 1562499 | 18 | 12 | 0 | 10 | 0 | 0 |
| 387.051024 | 387.051026 | 1986692 | 22 | 12 | 0 | 7 | 0 | 0 |
| 387.072145 | 387.072156 | 2738565 | 19 | 16 | 0 | 9 | 0 | 0 |
| 387.087417 | 387.087412 | 2579334 | 23 | 16 | 0 | 6 | 0 | 0 |
| 387.10858 | 387.108541 | 5052808 | 20 | 20 | 0 | 8 | 0 | 0 |
| 387.12377 | 387.123797 | 2240137 | 24 | 20 | 0 | 5 | 0 | 0 |
| 387.144901 | 387.144927 | 10802570 | 21 | 24 | 0 | 7 | 0 | 0 |
| 387.160184 | 387.160183 | 2843019 | 25 | 24 | 0 | 4 | 0 | 0 |
| 387.181322 | 387.181312 | 15734157 | 22 | 28 | 0 | 6 | 0 | 0 |
| 387.196608 | 387.196568 | 3122062 | 26 | 28 | 0 | 3 | 0 | 0 |
| 387.217682 | 387.217698 | 11859343 | 23 | 32 | 0 | 5 | 0 | 0 |
| 387.232975 | 387.232954 | 1893520 | 27 | 32 | 0 | 2 | 0 | 0 |
| 387.254068 | 387.254083 | 4883858 | 24 | 36 | 0 | 4 | 0 | 0 |
| 388.176592 | 388.176561 | 1634769 | 21 | 27 | 1 | 6 | 0 | 0 |
| 389.030268 | 389.030291 | 1365005 | 21 | 10 | 0 | 8 | 0 | 0 |
| 389.066721 | 389.066676 | 2980367 | 22 | 14 | 0 | 7 | 0 | 0 |
| 389.087785 | 389.087806 | 2054673 | 19 | 18 | 0 | 9 | 0 | 0 |
| 389.103021 | 389.103062 | 3082258 | 23 | 18 | 0 | 6 | 0 | 0 |
| 389.124205 | 389.124191 | 6053395 | 20 | 22 | 0 | 8 | 0 | 0 |
| 389.139472 | 389.139447 | 3319316 | 24 | 22 | 0 | 5 | 0 | 0 |
| 389.160588 | 389.160577 | 13481494 | 21 | 26 | 0 | 7 | 0 | 0 |
| 389.175841 | 389.175833 | 8078871 | 25 | 26 | 0 | 4 | 0 | 0 |
| 389.196952 | 389.196962 | 14439960 | 22 | 30 | 0 | 6 | 0 | 0 |
| 389.212204 | 389.212218 | 4735002 | 26 | 30 | 0 | 3 | 0 | 0 |
| 389.233367 | 389.233348 | 7419419 | 23 | 34 | 0 | 5 | 0 | 0 |
| 389.248615 | 389.248604 | 2529820 | 27 | 34 | 0 | 2 | 0 | 0 |
| 389.269799 | 389.269733 | 1874205 | 24 | 38 | 0 | 4 | 0 | 0 |
| 391.045949 | 391.045941 | 2554010 | 21 | 12 | 0 | 8 | 0 | 0 |
| 391.082383 | 391.082326 | 3115677 | 22 | 16 | 0 | 7 | 0 | 0 |
| 391.103474 | 391.103456 | 2354078 | 19 | 20 | 0 | 9 | 0 | 0 |
| 391.118721 | 391.118712 | 3288223 | 23 | 20 | 0 | 6 | 0 | 0 |
| 391.13984 | 391.139841 | 9056929 | 20 | 24 | 0 | 8 | 0 | 0 |
| 391.155105 | 391.155097 | 5428898 | 24 | 24 | 0 | 5 | 0 | 0 |
| 391.176232 | 391.176227 | 13960867 | 21 | 28 | 0 | 7 | 0 | 0 |
| 391.1915 | 391.191483 | 7185061 | 25 | 28 | 0 | 4 | 0 | 0 |
| 391.212615 | 391.212612 | 8732326 | 22 | 32 | 0 | 6 | 0 | 0 |
| 391.227853 | 391.227868 | 5481127 | 26 | 32 | 0 | 3 | 0 | 0 |
| 391.249014 | 391.248998 | 2509481 | 23 | 36 | 0 | 5 | 0 | 0 |
| 391.264178 | 391.264254 | 2401450 | 27 | 36 | 0 | 2 | 0 | 0 |
| 393.025257 | 393.025205 | 1503271 | 20 | 10 | 0 | 9 | 0 | 0 |
| 393.061625 | 393.061591 | 3290410 | 21 | 14 | 0 | 8 | 0 | 0 |
| 393.097977 | 393.097976 | 3685164 | 22 | 18 | 0 | 7 | 0 | 0 |
| 393.119139 | 393.119106 | 3630894 | 19 | 22 | 0 | 9 | 0 | 0 |
| 393.134386 | 393.134362 | 5193519 | 23 | 22 | 0 | 6 | 0 | 0 |
| 393.155518 | 393.155491 | 10493744 | 20 | 26 | 0 | 8 | 0 | 0 |
| 393.170743 | 393.170747 | 7877426 | 24 | 26 | 0 | 5 | 0 | 0 |
| 393.191878 | 393.191877 | 9648947 | 21 | 30 | 0 | 7 | 0 | 0 |
| 393.207158 | 393.207133 | 9453364 | 25 | 30 | 0 | 4 | 0 | 0 |
| 393.228295 | 393.228262 | 3767094 | 22 | 34 | 0 | 6 | 0 | 0 |
| 393.243551 | 393.243519 | 6389559 | 26 | 34 | 0 | 3 | 0 | 0 |
| 393.264647 | 393.264648 | 1272248 | 23 | 38 | 0 | 5 | 0 | 0 |
| 393.279909 | 393.279904 | 1842745 | 27 | 38 | 0 | 2 | 0 | 0 |
| 395.040897 | 395.040856 | 2622392 | 20 | 12 | 0 | 9 | 0 | 0 |
| 395.077206 | 395.077241 | 2770874 | 21 | 16 | 0 | 8 | 0 | 0 |
| 395.113635 | 395.113627 | 4306365 | 22 | 20 | 0 | 7 | 0 | 0 |
| 395.134788 | 395.134756 | 4436415 | 19 | 24 | 0 | 9 | 0 | 0 |
| 395.150024 | 395.150012 | 6991808 | 23 | 24 | 0 | 6 | 0 | 0 |
| 395.171137 | 395.171141 | 7222209 | 20 | 28 | 0 | 8 | 0 | 0 |
| 395.186413 | 395.186398 | 10778562 | 24 | 28 | 0 | 5 | 0 | 0 |
| 395.207602 | 395.207527 | 4094404 | 21 | 32 | 0 | 7 | 0 | 0 |
| 395.222801 | 395.222783 | 8715205 | 25 | 32 | 0 | 4 | 0 | 0 |
| 395.259176 | 395.259169 | 4633544 | 26 | 36 | 0 | 3 | 0 | 0 |
| 395.295568 | 395.295554 | 1283530 | 27 | 40 | 0 | 2 | 0 | 0 |
| 397.020145 | 397.02012 | 1349703 | 19 | 10 | 0 | 10 | 0 | 0 |
| 397.056521 | 397.056506 | 2607178 | 20 | 14 | 0 | 9 | 0 | 0 |
| 397.092891 | 397.092891 | 3502668 | 21 | 18 | 0 | 8 | 0 | 0 |
| 397.129318 | 397.129277 | 5824591 | 22 | 22 | 0 | 7 | 0 | 0 |
| 397.150426 | 397.150406 | 2995793 | 19 | 26 | 0 | 9 | 0 | 0 |
| 397.165657 | 397.165662 | 9984081 | 23 | 26 | 0 | 6 | 0 | 0 |
| 397.181007 | 397.180918 | 1201491 | 27 | 26 | 0 | 3 | 0 | 0 |
| 397.186794 | 397.186791 | 3062867 | 20 | 30 | 0 | 8 | 0 | 0 |
| 397.202081 | 397.202048 | 12361812 | 24 | 30 | 0 | 5 | 0 | 0 |
| 397.223219 | 397.223177 | 1355862 | 21 | 34 | 0 | 7 | 0 | 0 |
| 397.238442 | 397.238433 | 8365143 | 25 | 34 | 0 | 4 | 0 | 0 |
| 397.274823 | 397.274819 | 3167834 | 26 | 38 | 0 | 3 | 0 | 0 |
| 399.035802 | 399.03577 | 1216090 | 19 | 12 | 0 | 10 | 0 | 0 |
| 399.072144 | 399.072156 | 2459100 | 20 | 16 | 0 | 9 | 0 | 0 |
| 399.087434 | 399.087412 | 1539805 | 24 | 16 | 0 | 6 | 0 | 0 |
| 399.108543 | 399.108541 | 4594399 | 21 | 20 | 0 | 8 | 0 | 0 |
| 399.12372 | 399.123797 | 1273824 | 25 | 20 | 0 | 5 | 0 | 0 |
| 399.144945 | 399.144927 | 7767266 | 22 | 24 | 0 | 7 | 0 | 0 |
| 399.148301 | 399.148297 | 1513442 | 19 | 28 | 0 | 7 | 1 | 0 |
| 399.160258 | 399.160183 | 1454307 | 26 | 24 | 0 | 4 | 0 | 0 |
| 399.16605 | 399.166056 | 2430179 | 19 | 28 | 0 | 9 | 0 | 0 |
| 399.181309 | 399.181312 | 12835044 | 23 | 28 | 0 | 6 | 0 | 0 |
| 399.196573 | 399.196568 | 2031077 | 27 | 28 | 0 | 3 | 0 | 0 |
| 399.20239 | 399.202442 | 1316582 | 20 | 32 | 0 | 8 | 0 | 0 |
| 399.217692 | 399.217698 | 12531943 | 24 | 32 | 0 | 5 | 0 | 0 |
| 399.254086 | 399.254083 | 5689578 | 25 | 36 | 0 | 4 | 0 | 0 |
| 399.290464 | 399.290469 | 1190636 | 26 | 40 | 0 | 3 | 0 | 0 |
| 401.051428 | 401.05142 | 1413741 | 19 | 14 | 0 | 10 | 0 | 0 |
| 401.066694 | 401.066676 | 1961326 | 23 | 14 | 0 | 7 | 0 | 0 |
| 401.087757 | 401.087806 | 2530672 | 20 | 18 | 0 | 9 | 0 | 0 |
| 401.103062 | 401.103062 | 2650481 | 24 | 18 | 0 | 6 | 0 | 0 |
| 401.124208 | 401.124191 | 5805426 | 21 | 22 | 0 | 8 | 0 | 0 |
| 401.139516 | 401.139447 | 2206835 | 25 | 22 | 0 | 5 | 0 | 0 |
| 401.160583 | 401.160577 | 11209077 | 22 | 26 | 0 | 7 | 0 | 0 |
| 401.175809 | 401.175833 | 2729334 | 26 | 26 | 0 | 4 | 0 | 0 |
| 401.196956 | 401.196962 | 14541175 | 23 | 30 | 0 | 6 | 0 | 0 |
| 401.212229 | 401.212218 | 2888569 | 27 | 30 | 0 | 3 | 0 | 0 |
| 401.233346 | 401.233348 | 10467706 | 24 | 34 | 0 | 5 | 0 | 0 |
| 401.248598 | 401.248604 | 1643899 | 28 | 34 | 0 | 2 | 0 | 0 |
| 401.269718 | 401.269733 | 3053437 | 25 | 38 | 0 | 4 | 0 | 0 |
| 403.045906 | 403.045941 | 2267903 | 22 | 12 | 0 | 8 | 0 | 0 |
| 403.082373 | 403.082326 | 3279873 | 23 | 16 | 0 | 7 | 0 | 0 |
| 403.103426 | 403.103456 | 2928643 | 20 | 20 | 0 | 9 | 0 | 0 |
| 403.118694 | 403.118712 | 3309060 | 24 | 20 | 0 | 6 | 0 | 0 |
| 403.139858 | 403.139841 | 7907846 | 21 | 24 | 0 | 8 | 0 | 0 |
| 403.143228 | 403.143212 | 1419270 | 18 | 28 | 0 | 8 | 1 | 0 |
| 403.155057 | 403.155097 | 3837447 | 25 | 24 | 0 | 5 | 0 | 0 |
| 403.176233 | 403.176227 | 15071752 | 22 | 28 | 0 | 7 | 0 | 0 |
| 403.191494 | 403.191483 | 4678154 | 26 | 28 | 0 | 4 | 0 | 0 |
| 403.21262 | 403.212612 | 12517899 | 23 | 32 | 0 | 6 | 0 | 0 |
| 403.227858 | 403.227868 | 3795980 | 27 | 32 | 0 | 3 | 0 | 0 |
| 403.249011 | 403.248998 | 5699086 | 24 | 36 | 0 | 5 | 0 | 0 |
| 403.264383 | 403.264254 | 1443599 | 28 | 36 | 0 | 2 | 0 | 0 |
| 403.285313 | 403.285383 | 1514256 | 25 | 40 | 0 | 4 | 0 | 0 |
| 404.161537 | 404.16158 | 1289040 | 23 | 23 | 3 | 4 | 0 | 0 |
| 405.025251 | 405.025205 | 1618319 | 21 | 10 | 0 | 9 | 0 | 0 |
| 405.061538 | 405.061591 | 2879634 | 22 | 14 | 0 | 8 | 0 | 0 |
| 405.082783 | 405.08272 | 1658260 | 19 | 18 | 0 | 10 | 0 | 0 |
| 405.097959 | 405.097976 | 3570837 | 23 | 18 | 0 | 7 | 0 | 0 |
| 405.119084 | 405.119106 | 4198550 | 20 | 22 | 0 | 9 | 0 | 0 |
| 405.134354 | 405.134362 | 4210327 | 24 | 22 | 0 | 6 | 0 | 0 |
| 405.155486 | 405.155491 | 11387545 | 21 | 26 | 0 | 8 | 0 | 0 |
| 405.170749 | 405.170747 | 8023706 | 25 | 26 | 0 | 5 | 0 | 0 |
| 405.191883 | 405.191877 | 14203547 | 22 | 30 | 0 | 7 | 0 | 0 |
| 405.207128 | 405.207133 | 6611613 | 26 | 30 | 0 | 4 | 0 | 0 |
| 405.228263 | 405.228262 | 8202910 | 23 | 34 | 0 | 6 | 0 | 0 |
| 405.243502 | 405.243519 | 5494431 | 27 | 34 | 0 | 3 | 0 | 0 |
| 405.264643 | 405.264648 | 2609057 | 24 | 38 | 0 | 5 | 0 | 0 |
| 405.279952 | 405.279904 | 1803682 | 28 | 38 | 0 | 2 | 0 | 0 |
| 407.040833 | 407.040856 | 2688290 | 21 | 12 | 0 | 9 | 0 | 0 |
| 407.077249 | 407.077241 | 3428133 | 22 | 16 | 0 | 8 | 0 | 0 |
| 407.098383 | 407.09837 | 1795622 | 19 | 20 | 0 | 10 | 0 | 0 |
| 407.113633 | 407.113627 | 3410727 | 23 | 20 | 0 | 7 | 0 | 0 |
| 407.134733 | 407.134756 | 6357801 | 20 | 24 | 0 | 9 | 0 | 0 |
| 407.150016 | 407.150012 | 5804842 | 24 | 24 | 0 | 6 | 0 | 0 |
| 407.171131 | 407.171141 | 11905836 | 21 | 28 | 0 | 8 | 0 | 0 |
| 407.186402 | 407.186398 | 8869677 | 25 | 28 | 0 | 5 | 0 | 0 |
| 407.207522 | 407.207527 | 9501486 | 22 | 32 | 0 | 7 | 0 | 0 |
| 407.222773 | 407.222783 | 9225007 | 26 | 32 | 0 | 4 | 0 | 0 |
| 407.243893 | 407.243912 | 2631729 | 23 | 36 | 0 | 6 | 0 | 0 |
| 407.259188 | 407.259169 | 5502770 | 27 | 36 | 0 | 3 | 0 | 0 |
| 407.295553 | 407.295554 | 2261813 | 28 | 40 | 0 | 2 | 0 | 0 |
| 409.020087 | 409.02012 | 1621938 | 20 | 10 | 0 | 10 | 0 | 0 |
| 409.056532 | 409.056506 | 3286964 | 21 | 14 | 0 | 9 | 0 | 0 |
| 409.09288 | 409.092891 | 3980215 | 22 | 18 | 0 | 8 | 0 | 0 |
| 409.114053 | 409.11402 | 1448888 | 19 | 22 | 0 | 10 | 0 | 0 |
| 409.129278 | 409.129277 | 5544890 | 23 | 22 | 0 | 7 | 0 | 0 |
| 409.132622 | 409.132647 | 1301434 | 20 | 26 | 0 | 7 | 1 | 0 |
| 409.150395 | 409.150406 | 7293883 | 20 | 26 | 0 | 9 | 0 | 0 |
| 409.16564 | 409.165662 | 9718716 | 24 | 26 | 0 | 6 | 0 | 0 |
| 409.186803 | 409.186791 | 8081342 | 21 | 30 | 0 | 8 | 0 | 0 |
| 409.202059 | 409.202048 | 11817919 | 25 | 30 | 0 | 5 | 0 | 0 |
| 409.223182 | 409.223177 | 3778496 | 22 | 34 | 0 | 7 | 0 | 0 |
| 409.238429 | 409.238433 | 9757633 | 26 | 34 | 0 | 4 | 0 | 0 |
| 409.259686 | 409.259562 | 1258691 | 23 | 38 | 0 | 6 | 0 | 0 |
| 409.274815 | 409.274819 | 4729796 | 27 | 38 | 0 | 3 | 0 | 0 |
| 409.311217 | 409.311204 | 1380167 | 28 | 42 | 0 | 2 | 0 | 0 |
| 411.035745 | 411.03577 | 1440963 | 20 | 12 | 0 | 10 | 0 | 0 |
| 411.072111 | 411.072156 | 3006021 | 21 | 16 | 0 | 9 | 0 | 0 |
| 411.08741 | 411.087412 | 1565510 | 25 | 16 | 0 | 6 | 0 | 0 |
| 411.108553 | 411.108541 | 4291656 | 22 | 20 | 0 | 8 | 0 | 0 |
| 411.111948 | 411.111912 | 1235016 | 19 | 24 | 0 | 8 | 1 | 0 |
| 411.123743 | 411.123797 | 1219913 | 26 | 20 | 0 | 5 | 0 | 0 |
| 411.129648 | 411.129671 | 2017865 | 19 | 24 | 0 | 10 | 0 | 0 |
| 411.144931 | 411.144927 | 7899211 | 23 | 24 | 0 | 7 | 0 | 0 |
| 411.148282 | 411.148297 | 1457995 | 20 | 28 | 0 | 7 | 1 | 0 |
| 411.16604 | 411.166056 | 4441164 | 20 | 28 | 0 | 9 | 0 | 0 |
| 411.181307 | 411.181312 | 12430413 | 24 | 28 | 0 | 6 | 0 | 0 |
| 411.202457 | 411.202442 | 3590223 | 21 | 32 | 0 | 8 | 0 | 0 |
| 411.217697 | 411.217698 | 13022288 | 25 | 32 | 0 | 5 | 0 | 0 |
| 411.254093 | 411.254083 | 8171602 | 26 | 36 | 0 | 4 | 0 | 0 |
| 411.290491 | 411.290469 | 2901077 | 27 | 40 | 0 | 3 | 0 | 0 |
| 413.051468 | 413.05142 | 1731026 | 20 | 14 | 0 | 10 | 0 | 0 |
| 413.066663 | 413.066676 | 1650643 | 24 | 14 | 0 | 7 | 0 | 0 |
| 413.087826 | 413.087806 | 3183317 | 21 | 18 | 0 | 9 | 0 | 0 |
| 413.103055 | 413.103062 | 1961942 | 25 | 18 | 0 | 6 | 0 | 0 |
| 413.12418 | 413.124191 | 5486807 | 22 | 22 | 0 | 8 | 0 | 0 |
| 413.127544 | 413.127562 | 1785816 | 19 | 26 | 0 | 8 | 1 | 0 |
| 413.139524 | 413.139447 | 1299544 | 26 | 22 | 0 | 5 | 0 | 0 |
| 413.160586 | 413.160577 | 10319066 | 23 | 26 | 0 | 7 | 0 | 0 |
| 413.164003 | 413.163948 | 1957594 | 20 | 30 | 0 | 7 | 1 | 0 |
| 413.175813 | 413.175833 | 1931483 | 27 | 26 | 0 | 4 | 0 | 0 |
| 413.181666 | 413.181706 | 1855451 | 20 | 30 | 0 | 9 | 0 | 0 |
| 413.19697 | 413.196962 | 14925020 | 24 | 30 | 0 | 6 | 0 | 0 |
| 413.217946 | 413.218092 | 1552350 | 21 | 34 | 0 | 8 | 0 | 0 |
| 413.233339 | 413.233348 | 11910367 | 25 | 34 | 0 | 5 | 0 | 0 |
| 413.269726 | 413.269733 | 5720290 | 26 | 38 | 0 | 4 | 0 | 0 |
| 413.306065 | 413.306119 | 1320164 | 27 | 42 | 0 | 3 | 0 | 0 |
| 415.046031 | 415.045941 | 1487967 | 23 | 12 | 0 | 8 | 0 | 0 |
| 415.067157 | 415.06707 | 1676640 | 20 | 16 | 0 | 10 | 0 | 0 |
| 415.082305 | 415.082326 | 2432865 | 24 | 16 | 0 | 7 | 0 | 0 |
| 415.103456 | 415.103456 | 3357539 | 21 | 20 | 0 | 9 | 0 | 0 |
| 415.11876 | 415.118712 | 2595172 | 25 | 20 | 0 | 6 | 0 | 0 |
| 415.139869 | 415.139841 | 7766373 | 22 | 24 | 0 | 8 | 0 | 0 |
| 415.143242 | 415.143212 | 1799525 | 19 | 28 | 0 | 8 | 1 | 0 |
| 415.155073 | 415.155097 | 2402662 | 26 | 24 | 0 | 5 | 0 | 0 |
| 415.176238 | 415.176227 | 13895016 | 23 | 28 | 0 | 7 | 0 | 0 |
| 415.179551 | 415.179598 | 1465448 | 20 | 32 | 0 | 7 | 1 | 0 |
| 415.1915 | 415.191483 | 2949993 | 27 | 28 | 0 | 4 | 0 | 0 |
| 415.212607 | 415.212612 | 15527274 | 24 | 32 | 0 | 6 | 0 | 0 |
| 415.2279 | 415.227868 | 2244971 | 28 | 32 | 0 | 3 | 0 | 0 |
| 415.249032 | 415.248998 | 9149805 | 25 | 36 | 0 | 5 | 0 | 0 |
| 415.285468 | 415.285383 | 2574703 | 26 | 40 | 0 | 4 | 0 | 0 |
| 417.025254 | 417.025205 | 1447144 | 22 | 10 | 0 | 9 | 0 | 0 |
| 417.061554 | 417.061591 | 3110890 | 23 | 14 | 0 | 8 | 0 | 0 |
| 417.082701 | 417.08272 | 1938156 | 20 | 18 | 0 | 10 | 0 | 0 |
| 417.098 | 417.097976 | 3271149 | 24 | 18 | 0 | 7 | 0 | 0 |
| 417.119127 | 417.119106 | 4094446 | 21 | 22 | 0 | 9 | 0 | 0 |
| 417.134329 | 417.134362 | 2744815 | 25 | 22 | 0 | 6 | 0 | 0 |
| 417.155518 | 417.155491 | 9997809 | 22 | 26 | 0 | 8 | 0 | 0 |
| 417.158804 | 417.158862 | 1422833 | 19 | 30 | 0 | 8 | 1 | 0 |
| 417.170753 | 417.170747 | 4143602 | 26 | 26 | 0 | 5 | 0 | 0 |
| 417.191895 | 417.191877 | 14472691 | 23 | 30 | 0 | 7 | 0 | 0 |
| 417.207106 | 417.207133 | 4362741 | 27 | 30 | 0 | 4 | 0 | 0 |
| 417.228271 | 417.228262 | 12387830 | 24 | 34 | 0 | 6 | 0 | 0 |
| 417.243498 | 417.243519 | 2897399 | 28 | 34 | 0 | 3 | 0 | 0 |
| 417.264661 | 417.264648 | 4999673 | 25 | 38 | 0 | 5 | 0 | 0 |
| 419.040926 | 419.040856 | 2310770 | 22 | 12 | 0 | 9 | 0 | 0 |
| 419.077248 | 419.077241 | 3533940 | 23 | 16 | 0 | 8 | 0 | 0 |
| 419.09837 | 419.09837 | 1850485 | 20 | 20 | 0 | 10 | 0 | 0 |
| 419.113646 | 419.113627 | 3593846 | 24 | 20 | 0 | 7 | 0 | 0 |
| 419.134781 | 419.134756 | 5903992 | 21 | 24 | 0 | 9 | 0 | 0 |
| 419.15 | 419.150012 | 4462713 | 25 | 24 | 0 | 6 | 0 | 0 |
| 419.171159 | 419.171141 | 12719738 | 22 | 28 | 0 | 8 | 0 | 0 |
| 419.186412 | 419.186398 | 6067835 | 26 | 28 | 0 | 5 | 0 | 0 |
| 419.207559 | 419.207527 | 12359293 | 23 | 32 | 0 | 7 | 0 | 0 |
| 419.222807 | 419.222783 | 5990014 | 27 | 32 | 0 | 4 | 0 | 0 |
| 419.243916 | 419.243912 | 7444095 | 24 | 36 | 0 | 6 | 0 | 0 |
| 419.25918 | 419.259169 | 3685504 | 28 | 36 | 0 | 3 | 0 | 0 |
| 419.280347 | 419.280298 | 1602946 | 25 | 40 | 0 | 5 | 0 | 0 |
| 421.056561 | 421.056506 | 3241208 | 22 | 14 | 0 | 9 | 0 | 0 |
| 421.092948 | 421.092891 | 3891963 | 23 | 18 | 0 | 8 | 0 | 0 |
| 421.114106 | 421.11402 | 2051836 | 20 | 22 | 0 | 10 | 0 | 0 |
| 421.129301 | 421.129277 | 4517117 | 24 | 22 | 0 | 7 | 0 | 0 |
| 421.150444 | 421.150406 | 7912191 | 21 | 26 | 0 | 9 | 0 | 0 |
| 421.165674 | 421.165662 | 6182656 | 25 | 26 | 0 | 6 | 0 | 0 |
| 421.186811 | 421.186791 | 11198209 | 22 | 30 | 0 | 8 | 0 | 0 |
| 421.20209 | 421.202048 | 7788290 | 26 | 30 | 0 | 5 | 0 | 0 |
| 421.223187 | 421.223177 | 7070468 | 23 | 34 | 0 | 7 | 0 | 0 |
| 421.238472 | 421.238433 | 7037701 | 27 | 34 | 0 | 4 | 0 | 0 |
| 421.259586 | 421.259562 | 2694406 | 24 | 38 | 0 | 6 | 0 | 0 |
| 421.274832 | 421.274819 | 4064519 | 28 | 38 | 0 | 3 | 0 | 0 |
| 423.035784 | 423.03577 | 2175866 | 21 | 12 | 0 | 10 | 0 | 0 |
| 423.072182 | 423.072156 | 3078524 | 22 | 16 | 0 | 9 | 0 | 0 |
| 423.087462 | 423.087412 | 1303421 | 26 | 16 | 0 | 6 | 0 | 0 |
| 423.108525 | 423.108541 | 3703679 | 23 | 20 | 0 | 8 | 0 | 0 |
| 423.129702 | 423.129671 | 3050880 | 20 | 24 | 0 | 10 | 0 | 0 |
| 423.144939 | 423.144927 | 5817217 | 24 | 24 | 0 | 7 | 0 | 0 |
| 423.166093 | 423.166056 | 6159235 | 21 | 28 | 0 | 9 | 0 | 0 |
| 423.181327 | 423.181312 | 8913795 | 25 | 28 | 0 | 6 | 0 | 0 |
| 423.202437 | 423.202442 | 6344581 | 22 | 32 | 0 | 8 | 0 | 0 |
| 423.217721 | 423.217698 | 10135430 | 26 | 32 | 0 | 5 | 0 | 0 |
| 423.238902 | 423.238827 | 2423687 | 23 | 36 | 0 | 7 | 0 | 0 |
| 423.254071 | 423.254083 | 8005512 | 27 | 36 | 0 | 4 | 0 | 0 |
| 423.290486 | 423.290469 | 3865482 | 28 | 40 | 0 | 3 | 0 | 0 |
| 423.293933 | 423.29384 | 1455243 | 25 | 44 | 0 | 3 | 1 | 0 |
| 424.176694 | 424.176561 | 1205187 | 24 | 27 | 1 | 6 | 0 | 0 |
| 425.051443 | 425.05142 | 2085883 | 21 | 14 | 0 | 10 | 0 | 0 |
| 425.066633 | 425.066676 | 1308156 | 25 | 14 | 0 | 7 | 0 | 0 |
| 425.087818 | 425.087806 | 3315709 | 22 | 18 | 0 | 9 | 0 | 0 |
| 425.103093 | 425.103062 | 1576702 | 26 | 18 | 0 | 6 | 0 | 0 |
| 425.124206 | 425.124191 | 4593152 | 23 | 22 | 0 | 8 | 0 | 0 |
| 425.127661 | 425.127562 | 1507200 | 20 | 26 | 0 | 8 | 1 | 0 |
| 425.145348 | 425.145321 | 2269697 | 20 | 26 | 0 | 10 | 0 | 0 |
| 425.160573 | 425.160577 | 7758850 | 24 | 26 | 0 | 7 | 0 | 0 |
| 425.163901 | 425.163948 | 1291906 | 21 | 30 | 0 | 7 | 1 | 0 |
| 425.175783 | 425.175833 | 1383683 | 28 | 26 | 0 | 4 | 0 | 0 |
| 425.181701 | 425.181706 | 3720195 | 21 | 30 | 0 | 9 | 0 | 0 |
| 425.196967 | 425.196962 | 11425796 | 25 | 30 | 0 | 6 | 0 | 0 |
| 425.218099 | 425.218092 | 2384390 | 22 | 34 | 0 | 8 | 0 | 0 |
| 425.233357 | 425.233348 | 10928135 | 26 | 34 | 0 | 5 | 0 | 0 |
| 425.269752 | 425.269733 | 6431753 | 27 | 38 | 0 | 4 | 0 | 0 |
| 425.306043 | 425.306119 | 2491403 | 28 | 42 | 0 | 3 | 0 | 0 |
| 427.067033 | 427.06707 | 2010746 | 21 | 16 | 0 | 10 | 0 | 0 |
| 427.082293 | 427.082326 | 1825914 | 25 | 16 | 0 | 7 | 0 | 0 |
| 427.103453 | 427.103456 | 3029116 | 22 | 20 | 0 | 9 | 0 | 0 |
| 427.106878 | 427.106827 | 1311612 | 19 | 24 | 0 | 9 | 1 | 0 |
| 427.118778 | 427.118712 | 1913725 | 26 | 20 | 0 | 6 | 0 | 0 |
| 427.139849 | 427.139841 | 6248574 | 23 | 24 | 0 | 8 | 0 | 0 |
| 427.143225 | 427.143212 | 2329726 | 20 | 28 | 0 | 8 | 1 | 0 |
| 427.15503 | 427.155097 | 1501823 | 27 | 24 | 0 | 5 | 0 | 0 |
| 427.176236 | 427.176227 | 10928256 | 24 | 28 | 0 | 7 | 0 | 0 |
| 427.191499 | 427.191483 | 1613953 | 28 | 28 | 0 | 4 | 0 | 0 |
| 427.197369 | 427.197356 | 2049922 | 21 | 32 | 0 | 9 | 0 | 0 |
| 427.21264 | 427.212612 | 12427395 | 25 | 32 | 0 | 6 | 0 | 0 |
| 427.233717 | 427.233742 | 1270660 | 22 | 36 | 0 | 8 | 0 | 0 |
| 427.249027 | 427.248998 | 9429125 | 26 | 36 | 0 | 5 | 0 | 0 |
| 427.285368 | 427.285383 | 4187271 | 27 | 40 | 0 | 4 | 0 | 0 |
| 429.061563 | 429.061591 | 2551540 | 24 | 14 | 0 | 8 | 0 | 0 |
| 429.082721 | 429.08272 | 2123254 | 21 | 18 | 0 | 10 | 0 | 0 |
| 429.098005 | 429.097976 | 2374903 | 25 | 18 | 0 | 7 | 0 | 0 |
| 429.119146 | 429.119106 | 4094712 | 22 | 22 | 0 | 9 | 0 | 0 |
| 429.122555 | 429.122477 | 1721592 | 19 | 26 | 0 | 9 | 1 | 0 |
| 429.13438 | 429.134362 | 2285049 | 26 | 22 | 0 | 6 | 0 | 0 |
| 429.155493 | 429.155491 | 8570106 | 23 | 26 | 0 | 8 | 0 | 0 |
| 429.158841 | 429.158862 | 1913594 | 20 | 30 | 0 | 8 | 1 | 0 |
| 429.170713 | 429.170747 | 2401787 | 27 | 26 | 0 | 5 | 0 | 0 |
| 429.191882 | 429.191877 | 13402364 | 24 | 30 | 0 | 7 | 0 | 0 |
| 429.207135 | 429.207133 | 2245629 | 28 | 30 | 0 | 4 | 0 | 0 |
| 429.22826 | 429.228262 | 13422846 | 25 | 34 | 0 | 6 | 0 | 0 |
| 429.264653 | 429.264648 | 6615297 | 26 | 38 | 0 | 5 | 0 | 0 |
| 429.301066 | 429.301033 | 2083587 | 27 | 42 | 0 | 4 | 0 | 0 |
| 431.040862 | 431.040856 | 2183020 | 23 | 12 | 0 | 9 | 0 | 0 |
| 431.077245 | 431.077241 | 3075438 | 24 | 16 | 0 | 8 | 0 | 0 |
| 431.098256 | 431.09837 | 2699631 | 21 | 20 | 0 | 10 | 0 | 0 |
| 431.113629 | 431.113627 | 2962288 | 25 | 20 | 0 | 7 | 0 | 0 |
| 431.134779 | 431.134756 | 5494130 | 22 | 24 | 0 | 9 | 0 | 0 |
| 431.138184 | 431.138127 | 1497458 | 19 | 28 | 0 | 9 | 1 | 0 |
| 431.150005 | 431.150012 | 3129715 | 26 | 24 | 0 | 6 | 0 | 0 |
| 431.171159 | 431.171141 | 10905972 | 23 | 28 | 0 | 8 | 0 | 0 |
| 431.174572 | 431.174512 | 1342068 | 20 | 32 | 0 | 8 | 1 | 0 |
| 431.186479 | 431.186398 | 3898741 | 27 | 28 | 0 | 5 | 0 | 0 |
| 431.20754 | 431.207527 | 15495542 | 24 | 32 | 0 | 7 | 0 | 0 |
| 431.222774 | 431.222783 | 3829623 | 28 | 32 | 0 | 4 | 0 | 0 |
| 431.243902 | 431.243912 | 11319672 | 25 | 36 | 0 | 6 | 0 | 0 |
| 431.259106 | 431.259169 | 2027897 | 29 | 36 | 0 | 3 | 0 | 0 |
| 431.280265 | 431.280298 | 3487610 | 26 | 40 | 0 | 5 | 0 | 0 |
| 433.056515 | 433.056506 | 3431396 | 23 | 14 | 0 | 9 | 0 | 0 |
| 433.077605 | 433.077635 | 1581541 | 20 | 18 | 0 | 11 | 0 | 0 |
| 433.09289 | 433.092891 | 3081190 | 24 | 18 | 0 | 8 | 0 | 0 |
| 433.114021 | 433.11402 | 2567143 | 21 | 22 | 0 | 10 | 0 | 0 |
| 433.129279 | 433.129277 | 3716584 | 25 | 22 | 0 | 7 | 0 | 0 |
| 433.150423 | 433.150406 | 8235498 | 22 | 26 | 0 | 9 | 0 | 0 |
| 433.153769 | 433.153777 | 1285354 | 19 | 30 | 0 | 9 | 1 | 0 |
| 433.165667 | 433.165662 | 4592106 | 26 | 26 | 0 | 6 | 0 | 0 |
| 433.186803 | 433.186791 | 13816299 | 23 | 30 | 0 | 8 | 0 | 0 |
| 433.20204 | 433.202048 | 5608941 | 27 | 30 | 0 | 5 | 0 | 0 |
| 433.223177 | 433.223177 | 13109742 | 24 | 34 | 0 | 7 | 0 | 0 |
| 433.238465 | 433.238433 | 4464111 | 28 | 34 | 0 | 4 | 0 | 0 |
| 433.259558 | 433.259562 | 6137328 | 25 | 38 | 0 | 6 | 0 | 0 |
| 433.274766 | 433.274819 | 1960433 | 29 | 38 | 0 | 3 | 0 | 0 |
| 434.172096 | 434.172144 | 1705253 | 24 | 25 | 3 | 5 | 0 | 0 |
| 435.035783 | 435.03577 | 1554776 | 22 | 12 | 0 | 10 | 0 | 0 |
| 435.072118 | 435.072156 | 3852890 | 23 | 16 | 0 | 9 | 0 | 0 |
| 435.108474 | 435.108541 | 4021852 | 24 | 20 | 0 | 8 | 0 | 0 |
| 435.129702 | 435.129671 | 3341405 | 21 | 24 | 0 | 10 | 0 | 0 |
| 435.144909 | 435.144927 | 5056094 | 25 | 24 | 0 | 7 | 0 | 0 |
| 435.166055 | 435.166056 | 9332319 | 22 | 28 | 0 | 9 | 0 | 0 |
| 435.181315 | 435.181312 | 7116384 | 26 | 28 | 0 | 6 | 0 | 0 |
| 435.202449 | 435.202442 | 11598433 | 23 | 32 | 0 | 8 | 0 | 0 |
| 435.217675 | 435.217698 | 8571490 | 27 | 32 | 0 | 5 | 0 | 0 |
| 435.238809 | 435.238827 | 7223908 | 24 | 36 | 0 | 7 | 0 | 0 |
| 435.254076 | 435.254083 | 6892133 | 28 | 36 | 0 | 4 | 0 | 0 |
| 435.275263 | 435.275213 | 2283878 | 25 | 40 | 0 | 6 | 0 | 0 |
| 435.290449 | 435.290469 | 2578023 | 29 | 40 | 0 | 3 | 0 | 0 |
| 437.051432 | 437.05142 | 2458316 | 22 | 14 | 0 | 10 | 0 | 0 |
| 437.087795 | 437.087806 | 3444430 | 23 | 18 | 0 | 9 | 0 | 0 |
| 437.108975 | 437.108935 | 1480143 | 20 | 22 | 0 | 11 | 0 | 0 |
| 437.124164 | 437.124191 | 4543184 | 24 | 22 | 0 | 8 | 0 | 0 |
| 437.145305 | 437.145321 | 4521682 | 21 | 26 | 0 | 10 | 0 | 0 |
| 437.16056 | 437.160577 | 7168722 | 25 | 26 | 0 | 7 | 0 | 0 |
| 437.163944 | 437.163948 | 1684690 | 22 | 30 | 0 | 7 | 1 | 0 |
| 437.181682 | 437.181706 | 7968468 | 22 | 30 | 0 | 9 | 0 | 0 |
| 437.196955 | 437.196962 | 10111700 | 26 | 30 | 0 | 6 | 0 | 0 |
| 437.218091 | 437.218092 | 6259414 | 23 | 34 | 0 | 8 | 0 | 0 |
| 437.233348 | 437.233348 | 10474198 | 27 | 34 | 0 | 5 | 0 | 0 |
| 437.254465 | 437.254477 | 2946776 | 24 | 38 | 0 | 7 | 0 | 0 |
| 437.269717 | 437.269733 | 6934233 | 28 | 38 | 0 | 4 | 0 | 0 |
| 437.306114 | 437.306119 | 2440411 | 29 | 42 | 0 | 3 | 0 | 0 |
| 439.030684 | 439.030685 | 1274941 | 21 | 12 | 0 | 11 | 0 | 0 |
| 439.067081 | 439.06707 | 2971455 | 22 | 16 | 0 | 10 | 0 | 0 |
| 439.082349 | 439.082326 | 1637184 | 26 | 16 | 0 | 7 | 0 | 0 |
| 439.103462 | 439.103456 | 3668801 | 23 | 20 | 0 | 9 | 0 | 0 |
| 439.106938 | 439.106827 | 1360705 | 20 | 24 | 0 | 9 | 1 | 0 |
| 439.118708 | 439.118712 | 1661762 | 27 | 20 | 0 | 6 | 0 | 0 |
| 439.124591 | 439.124585 | 1396546 | 20 | 24 | 0 | 11 | 0 | 0 |
| 439.139866 | 439.139841 | 5267267 | 24 | 24 | 0 | 8 | 0 | 0 |
| 439.143183 | 439.143212 | 1813315 | 21 | 28 | 0 | 8 | 1 | 0 |
| 439.160995 | 439.160971 | 3400004 | 21 | 28 | 0 | 10 | 0 | 0 |
| 439.176229 | 439.176227 | 10349381 | 25 | 28 | 0 | 7 | 0 | 0 |
| 439.179605 | 439.179598 | 1388357 | 22 | 32 | 0 | 7 | 1 | 0 |
| 439.197362 | 439.197356 | 3877702 | 22 | 32 | 0 | 9 | 0 | 0 |
| 439.212606 | 439.212612 | 12612423 | 26 | 32 | 0 | 6 | 0 | 0 |
| 439.233783 | 439.233742 | 2468169 | 23 | 36 | 0 | 8 | 0 | 0 |
| 439.248988 | 439.248998 | 11586377 | 27 | 36 | 0 | 5 | 0 | 0 |
| 439.285346 | 439.285383 | 6764364 | 28 | 40 | 0 | 4 | 0 | 0 |
| 439.321738 | 439.321769 | 1717069 | 29 | 44 | 0 | 3 | 0 | 0 |
| 441.046356 | 441.046335 | 1343407 | 21 | 14 | 0 | 11 | 0 | 0 |
| 441.061615 | 441.061591 | 2160560 | 25 | 14 | 0 | 8 | 0 | 0 |
| 441.082657 | 441.08272 | 2510769 | 22 | 18 | 0 | 10 | 0 | 0 |
| 441.098007 | 441.097976 | 2275762 | 26 | 18 | 0 | 7 | 0 | 0 |
| 441.119147 | 441.119106 | 4221363 | 23 | 22 | 0 | 9 | 0 | 0 |
| 441.122503 | 441.122477 | 2049203 | 20 | 26 | 0 | 9 | 1 | 0 |
| 441.134391 | 441.134362 | 2168756 | 27 | 22 | 0 | 6 | 0 | 0 |
| 441.155492 | 441.155491 | 8374197 | 24 | 26 | 0 | 8 | 0 | 0 |
| 441.158852 | 441.158862 | 2526133 | 21 | 30 | 0 | 8 | 1 | 0 |
| 441.170702 | 441.170747 | 1972150 | 28 | 26 | 0 | 5 | 0 | 0 |
| 441.176614 | 441.176621 | 1800118 | 21 | 30 | 0 | 10 | 0 | 0 |
| 441.191872 | 441.191877 | 13304759 | 25 | 30 | 0 | 7 | 0 | 0 |
| 441.207153 | 441.207133 | 2212792 | 29 | 30 | 0 | 4 | 0 | 0 |
| 441.213028 | 441.213006 | 1631672 | 22 | 34 | 0 | 9 | 0 | 0 |
| 441.228248 | 441.228262 | 14791609 | 26 | 34 | 0 | 6 | 0 | 0 |
| 441.264653 | 441.264648 | 9823163 | 27 | 38 | 0 | 5 | 0 | 0 |
| 441.301068 | 441.301033 | 4541373 | 28 | 42 | 0 | 4 | 0 | 0 |
| 443.040923 | 443.040856 | 2037278 | 24 | 12 | 0 | 9 | 0 | 0 |
| 443.061978 | 443.061985 | 1594655 | 21 | 16 | 0 | 11 | 0 | 0 |
| 443.077252 | 443.077241 | 3056160 | 25 | 16 | 0 | 8 | 0 | 0 |
| 443.098378 | 443.09837 | 2092065 | 22 | 20 | 0 | 10 | 0 | 0 |
| 443.113716 | 443.113627 | 2192674 | 26 | 20 | 0 | 7 | 0 | 0 |
| 443.134736 | 443.134756 | 5150756 | 23 | 24 | 0 | 9 | 0 | 0 |
| 443.138145 | 443.138127 | 2702884 | 20 | 28 | 0 | 9 | 1 | 0 |
| 443.150006 | 443.150012 | 2663972 | 27 | 24 | 0 | 6 | 0 | 0 |
| 443.171144 | 443.171141 | 10978341 | 24 | 28 | 0 | 8 | 0 | 0 |
| 443.174522 | 443.174512 | 1519654 | 21 | 32 | 0 | 8 | 1 | 0 |
| 443.18642 | 443.186398 | 3160614 | 28 | 28 | 0 | 5 | 0 | 0 |
| 443.207511 | 443.207527 | 16677927 | 25 | 32 | 0 | 7 | 0 | 0 |
| 443.22282 | 443.222783 | 2160168 | 29 | 32 | 0 | 4 | 0 | 0 |
| 443.243911 | 443.243912 | 12663849 | 26 | 36 | 0 | 6 | 0 | 0 |
| 443.280289 | 443.280298 | 6317100 | 27 | 40 | 0 | 5 | 0 | 0 |
| 443.316744 | 443.316683 | 1856302 | 28 | 44 | 0 | 4 | 0 | 0 |
| 445.020027 | 445.02012 | 1309196 | 23 | 10 | 0 | 10 | 0 | 0 |
| 445.056474 | 445.056506 | 2530958 | 24 | 14 | 0 | 9 | 0 | 0 |
| 445.092854 | 445.092891 | 3444880 | 25 | 18 | 0 | 8 | 0 | 0 |
| 445.114037 | 445.11402 | 2590865 | 22 | 22 | 0 | 10 | 0 | 0 |
| 445.129269 | 445.129277 | 3152530 | 26 | 22 | 0 | 7 | 0 | 0 |
| 445.150379 | 445.150406 | 7584915 | 23 | 26 | 0 | 9 | 0 | 0 |
| 445.153743 | 445.153777 | 2210707 | 20 | 30 | 0 | 9 | 1 | 0 |
| 445.165657 | 445.165662 | 4004500 | 27 | 26 | 0 | 6 | 0 | 0 |
| 445.186804 | 445.186791 | 15334549 | 24 | 30 | 0 | 8 | 0 | 0 |
| 445.202056 | 445.202048 | 4080790 | 28 | 30 | 0 | 5 | 0 | 0 |
| 445.223182 | 445.223177 | 16780440 | 25 | 34 | 0 | 7 | 0 | 0 |
| 445.238381 | 445.238433 | 3060888 | 29 | 34 | 0 | 4 | 0 | 0 |
| 445.259546 | 445.259562 | 8791193 | 26 | 38 | 0 | 6 | 0 | 0 |
| 445.295957 | 445.295948 | 3762331 | 27 | 42 | 0 | 5 | 0 | 0 |
| 446.172141 | 446.172144 | 1289164 | 25 | 25 | 3 | 5 | 0 | 0 |
| 447.035755 | 447.03577 | 1879803 | 23 | 12 | 0 | 10 | 0 | 0 |
| 447.07216 | 447.072156 | 3265789 | 24 | 16 | 0 | 9 | 0 | 0 |
| 447.093309 | 447.093285 | 2306047 | 21 | 20 | 0 | 11 | 0 | 0 |
| 447.108548 | 447.108541 | 3481855 | 25 | 20 | 0 | 8 | 0 | 0 |
| 447.129667 | 447.129671 | 4120833 | 22 | 24 | 0 | 10 | 0 | 0 |
| 447.144934 | 447.144927 | 3995905 | 26 | 24 | 0 | 7 | 0 | 0 |
| 447.166039 | 447.166056 | 9913602 | 23 | 28 | 0 | 9 | 0 | 0 |
| 447.181315 | 447.181312 | 5726467 | 27 | 28 | 0 | 6 | 0 | 0 |
| 447.202445 | 447.202442 | 15582468 | 24 | 32 | 0 | 8 | 0 | 0 |
| 447.217686 | 447.217698 | 5727493 | 28 | 32 | 0 | 5 | 0 | 0 |
| 447.238803 | 447.238827 | 13147398 | 25 | 36 | 0 | 7 | 0 | 0 |
| 447.254088 | 447.254083 | 3431175 | 29 | 36 | 0 | 4 | 0 | 0 |
| 447.275222 | 447.275213 | 4966665 | 26 | 40 | 0 | 6 | 0 | 0 |
| 447.311539 | 447.311598 | 1439754 | 27 | 44 | 0 | 5 | 0 | 0 |
| 449.01495 | 449.015035 | 1235048 | 22 | 10 | 0 | 11 | 0 | 0 |
| 449.051492 | 449.05142 | 2724714 | 23 | 14 | 0 | 10 | 0 | 0 |
| 449.087829 | 449.087806 | 3988332 | 24 | 18 | 0 | 9 | 0 | 0 |
| 449.10895 | 449.108935 | 1425517 | 21 | 22 | 0 | 11 | 0 | 0 |
| 449.124187 | 449.124191 | 4345198 | 25 | 22 | 0 | 8 | 0 | 0 |
| 449.145303 | 449.145321 | 5934447 | 22 | 26 | 0 | 10 | 0 | 0 |
| 449.160588 | 449.160577 | 5529968 | 26 | 26 | 0 | 7 | 0 | 0 |
| 449.163961 | 449.163948 | 1252720 | 23 | 30 | 0 | 7 | 1 | 0 |
| 449.1817 | 449.181706 | 10589553 | 23 | 30 | 0 | 9 | 0 | 0 |
| 449.196948 | 449.196962 | 7571826 | 27 | 30 | 0 | 6 | 0 | 0 |
| 449.218079 | 449.218092 | 11214195 | 24 | 34 | 0 | 8 | 0 | 0 |
| 449.233351 | 449.233348 | 7035252 | 28 | 34 | 0 | 5 | 0 | 0 |
| 449.254446 | 449.254477 | 7419253 | 25 | 38 | 0 | 7 | 0 | 0 |
| 449.269759 | 449.269733 | 4163958 | 29 | 38 | 0 | 4 | 0 | 0 |
| 449.290861 | 449.290863 | 1527671 | 26 | 42 | 0 | 6 | 0 | 0 |
| 451.030627 | 451.030685 | 1286871 | 22 | 12 | 0 | 11 | 0 | 0 |
| 451.067059 | 451.06707 | 3137497 | 23 | 16 | 0 | 10 | 0 | 0 |
| 451.10346 | 451.103456 | 3800027 | 24 | 20 | 0 | 9 | 0 | 0 |
| 451.118719 | 451.118712 | 1221852 | 28 | 20 | 0 | 6 | 0 | 0 |
| 451.124565 | 451.124585 | 1982940 | 21 | 24 | 0 | 11 | 0 | 0 |
| 451.139828 | 451.139841 | 4927965 | 25 | 24 | 0 | 8 | 0 | 0 |
| 451.143176 | 451.143212 | 1857245 | 22 | 28 | 0 | 8 | 1 | 0 |
| 451.160967 | 451.160971 | 5670366 | 22 | 28 | 0 | 10 | 0 | 0 |
| 451.176218 | 451.176227 | 7498207 | 26 | 28 | 0 | 7 | 0 | 0 |
| 451.17962 | 451.179598 | 1269727 | 23 | 32 | 0 | 7 | 1 | 0 |
| 451.197346 | 451.197356 | 8273376 | 23 | 32 | 0 | 9 | 0 | 0 |
| 451.212633 | 451.212612 | 9805281 | 27 | 32 | 0 | 6 | 0 | 0 |
| 451.233727 | 451.233742 | 5751266 | 24 | 36 | 0 | 8 | 0 | 0 |
| 451.249002 | 451.248998 | 9614819 | 28 | 36 | 0 | 5 | 0 | 0 |
| 451.270135 | 451.270127 | 2449380 | 25 | 40 | 0 | 7 | 0 | 0 |
| 451.285415 | 451.285383 | 5079525 | 29 | 40 | 0 | 4 | 0 | 0 |
| 453.046381 | 453.046335 | 1830981 | 22 | 14 | 0 | 11 | 0 | 0 |
| 453.061682 | 453.061591 | 1535558 | 26 | 14 | 0 | 8 | 0 | 0 |
| 453.082703 | 453.08272 | 2809927 | 23 | 18 | 0 | 10 | 0 | 0 |
| 453.097909 | 453.097976 | 1698888 | 27 | 18 | 0 | 7 | 0 | 0 |
| 453.119106 | 453.119106 | 4023881 | 24 | 22 | 0 | 9 | 0 | 0 |
| 453.122492 | 453.122477 | 1496393 | 21 | 26 | 0 | 9 | 1 | 0 |
| 453.140257 | 453.140235 | 2029130 | 21 | 26 | 0 | 11 | 0 | 0 |
| 453.155502 | 453.155491 | 6469195 | 25 | 26 | 0 | 8 | 0 | 0 |
| 453.158934 | 453.158862 | 2657356 | 22 | 30 | 0 | 8 | 1 | 0 |
| 453.170756 | 453.170747 | 1527628 | 29 | 26 | 0 | 5 | 0 | 0 |
| 453.17666 | 453.176621 | 3386445 | 22 | 30 | 0 | 10 | 0 | 0 |
| 453.191874 | 453.191877 | 10821197 | 26 | 30 | 0 | 7 | 0 | 0 |
| 453.195161 | 453.195248 | 1594445 | 23 | 34 | 0 | 7 | 1 | 0 |
| 453.207201 | 453.207133 | 1295182 | 30 | 30 | 0 | 4 | 0 | 0 |
| 453.213012 | 453.213006 | 3931215 | 23 | 34 | 0 | 9 | 0 | 0 |
| 453.22826 | 453.228262 | 12404303 | 27 | 34 | 0 | 6 | 0 | 0 |
| 453.249368 | 453.249392 | 1844560 | 24 | 38 | 0 | 8 | 0 | 0 |
| 453.264643 | 453.264648 | 9928273 | 28 | 38 | 0 | 5 | 0 | 0 |
| 453.301043 | 453.301033 | 5072467 | 29 | 42 | 0 | 4 | 0 | 0 |
| 455.040885 | 455.040856 | 1623987 | 25 | 12 | 0 | 9 | 0 | 0 |
| 455.062016 | 455.061985 | 1779124 | 22 | 16 | 0 | 11 | 0 | 0 |
| 455.077279 | 455.077241 | 2397877 | 26 | 16 | 0 | 8 | 0 | 0 |
| 455.098403 | 455.09837 | 3087030 | 23 | 20 | 0 | 10 | 0 | 0 |
| 455.11369 | 455.113627 | 2246583 | 27 | 20 | 0 | 7 | 0 | 0 |
| 455.134787 | 455.134756 | 4705976 | 24 | 24 | 0 | 9 | 0 | 0 |
| 455.138077 | 455.138127 | 2542776 | 21 | 28 | 0 | 9 | 1 | 0 |
| 455.149966 | 455.150012 | 1734841 | 28 | 24 | 0 | 6 | 0 | 0 |
| 455.171157 | 455.171141 | 8883898 | 25 | 28 | 0 | 8 | 0 | 0 |
| 455.174469 | 455.174512 | 2301370 | 22 | 32 | 0 | 8 | 1 | 0 |
| 455.186457 | 455.186398 | 1919419 | 29 | 28 | 0 | 5 | 0 | 0 |
| 455.192308 | 455.192271 | 1857979 | 22 | 32 | 0 | 10 | 0 | 0 |
| 455.207536 | 455.207527 | 13344444 | 26 | 32 | 0 | 7 | 0 | 0 |
| 455.222826 | 455.222783 | 1484349 | 30 | 32 | 0 | 4 | 0 | 0 |
| 455.228644 | 455.228656 | 1607613 | 23 | 36 | 0 | 9 | 0 | 0 |
| 455.243915 | 455.243912 | 14044862 | 27 | 36 | 0 | 6 | 0 | 0 |
| 455.280307 | 455.280298 | 8683200 | 28 | 40 | 0 | 5 | 0 | 0 |
| 455.316681 | 455.316683 | 4171970 | 29 | 44 | 0 | 4 | 0 | 0 |
| 455.353129 | 455.353069 | 3761348 | 30 | 48 | 0 | 3 | 0 | 0 |
| 457.056526 | 457.056506 | 2489378 | 25 | 14 | 0 | 9 | 0 | 0 |
| 457.077637 | 457.077635 | 1957155 | 22 | 18 | 0 | 11 | 0 | 0 |
| 457.09286 | 457.092891 | 3205924 | 26 | 18 | 0 | 8 | 0 | 0 |
| 457.114051 | 457.11402 | 2761509 | 23 | 22 | 0 | 10 | 0 | 0 |
| 457.117359 | 457.117391 | 1384741 | 20 | 26 | 0 | 10 | 1 | 0 |
| 457.129337 | 457.129277 | 2792230 | 27 | 22 | 0 | 7 | 0 | 0 |
| 457.150428 | 457.150406 | 6961959 | 24 | 26 | 0 | 9 | 0 | 0 |
| 457.153818 | 457.153777 | 2680615 | 21 | 30 | 0 | 9 | 1 | 0 |
| 457.165659 | 457.165662 | 2778920 | 28 | 26 | 0 | 6 | 0 | 0 |
| 457.186802 | 457.186791 | 12719913 | 25 | 30 | 0 | 8 | 0 | 0 |
| 457.190177 | 457.190162 | 1572905 | 22 | 34 | 0 | 8 | 1 | 0 |
| 457.202111 | 457.202048 | 2597674 | 29 | 30 | 0 | 5 | 0 | 0 |
| 457.223186 | 457.223177 | 14767915 | 26 | 34 | 0 | 7 | 0 | 0 |
| 457.238471 | 457.238433 | 1843756 | 30 | 34 | 0 | 4 | 0 | 0 |
| 457.259574 | 457.259562 | 12080941 | 27 | 38 | 0 | 6 | 0 | 0 |
| 457.295978 | 457.295948 | 6381359 | 28 | 42 | 0 | 5 | 0 | 0 |
| 457.332382 | 457.332334 | 1650481 | 29 | 46 | 0 | 4 | 0 | 0 |
| 459.035852 | 459.03577 | 1697424 | 24 | 12 | 0 | 10 | 0 | 0 |
| 459.072165 | 459.072156 | 3399058 | 25 | 16 | 0 | 9 | 0 | 0 |
| 459.093219 | 459.093285 | 1357203 | 22 | 20 | 0 | 11 | 0 | 0 |
| 459.10855 | 459.108541 | 2978708 | 26 | 20 | 0 | 8 | 0 | 0 |
| 459.12968 | 459.129671 | 3912597 | 23 | 24 | 0 | 10 | 0 | 0 |
| 459.13302 | 459.133041 | 1657749 | 20 | 28 | 0 | 10 | 1 | 0 |
| 459.144943 | 459.144927 | 3080598 | 27 | 24 | 0 | 7 | 0 | 0 |
| 459.166071 | 459.166056 | 9224087 | 24 | 28 | 0 | 9 | 0 | 0 |
| 459.169322 | 459.169427 | 1805719 | 21 | 32 | 0 | 9 | 1 | 0 |
| 459.18131 | 459.181312 | 4120472 | 28 | 28 | 0 | 6 | 0 | 0 |
| 459.202455 | 459.202442 | 15107993 | 25 | 32 | 0 | 8 | 0 | 0 |
| 459.217658 | 459.217698 | 3015066 | 29 | 32 | 0 | 5 | 0 | 0 |
| 459.238825 | 459.238827 | 13748123 | 26 | 36 | 0 | 7 | 0 | 0 |
| 459.254136 | 459.254083 | 1867676 | 30 | 36 | 0 | 4 | 0 | 0 |
| 459.275214 | 459.275213 | 8880029 | 27 | 40 | 0 | 6 | 0 | 0 |
| 459.311619 | 459.311598 | 2869663 | 28 | 44 | 0 | 5 | 0 | 0 |
| 461.051434 | 461.05142 | 2631168 | 24 | 14 | 0 | 10 | 0 | 0 |
| 461.087792 | 461.087806 | 3262466 | 25 | 18 | 0 | 9 | 0 | 0 |
| 461.108873 | 461.108935 | 1794563 | 22 | 22 | 0 | 11 | 0 | 0 |
| 461.124161 | 461.124191 | 3440132 | 26 | 22 | 0 | 8 | 0 | 0 |
| 461.145336 | 461.145321 | 5017606 | 23 | 26 | 0 | 10 | 0 | 0 |
| 461.160597 | 461.160577 | 4333575 | 27 | 26 | 0 | 7 | 0 | 0 |
| 461.181725 | 461.181706 | 11212807 | 24 | 30 | 0 | 9 | 0 | 0 |
| 461.196981 | 461.196962 | 4845577 | 28 | 30 | 0 | 6 | 0 | 0 |
| 461.218092 | 461.218092 | 15343625 | 25 | 34 | 0 | 8 | 0 | 0 |
| 461.233337 | 461.233348 | 4313099 | 29 | 34 | 0 | 5 | 0 | 0 |
| 461.254483 | 461.254477 | 10031116 | 26 | 38 | 0 | 7 | 0 | 0 |
| 461.269711 | 461.269733 | 2227980 | 30 | 38 | 0 | 4 | 0 | 0 |
| 461.290871 | 461.290863 | 3987470 | 27 | 42 | 0 | 6 | 0 | 0 |
| 463.030794 | 463.030685 | 1471600 | 23 | 12 | 0 | 11 | 0 | 0 |
| 463.067067 | 463.06707 | 3061874 | 24 | 16 | 0 | 10 | 0 | 0 |
| 463.103418 | 463.103456 | 3737716 | 25 | 20 | 0 | 9 | 0 | 0 |
| 463.124535 | 463.124585 | 2511477 | 22 | 24 | 0 | 11 | 0 | 0 |
| 463.139831 | 463.139841 | 3913846 | 26 | 24 | 0 | 8 | 0 | 0 |
| 463.160964 | 463.160971 | 6376567 | 23 | 28 | 0 | 10 | 0 | 0 |
| 463.176226 | 463.176227 | 5655672 | 27 | 28 | 0 | 7 | 0 | 0 |
| 463.197368 | 463.197356 | 11290745 | 24 | 32 | 0 | 9 | 0 | 0 |
| 463.212608 | 463.212612 | 6915194 | 28 | 32 | 0 | 6 | 0 | 0 |
| 463.233759 | 463.233742 | 10223739 | 25 | 36 | 0 | 8 | 0 | 0 |
| 463.248989 | 463.248998 | 5188732 | 29 | 36 | 0 | 5 | 0 | 0 |
| 463.270128 | 463.270127 | 4652157 | 26 | 40 | 0 | 7 | 0 | 0 |
| 463.285434 | 463.285383 | 2036350 | 30 | 40 | 0 | 4 | 0 | 0 |
| 463.306562 | 463.306513 | 1299583 | 27 | 44 | 0 | 6 | 0 | 0 |
| 463.325211 | 463.32514 | 1403776 | 28 | 48 | 0 | 3 | 1 | 0 |
| 465.046373 | 465.046335 | 1998818 | 23 | 14 | 0 | 11 | 0 | 0 |
| 465.082691 | 465.08272 | 3096804 | 24 | 18 | 0 | 10 | 0 | 0 |
| 465.098133 | 465.097976 | 1447909 | 28 | 18 | 0 | 7 | 0 | 0 |
| 465.119097 | 465.119106 | 3377894 | 25 | 22 | 0 | 9 | 0 | 0 |
| 465.122426 | 465.122477 | 1255399 | 22 | 26 | 0 | 9 | 1 | 0 |
| 465.140259 | 465.140235 | 2479336 | 22 | 26 | 0 | 11 | 0 | 0 |
| 465.155525 | 465.155491 | 5264617 | 26 | 26 | 0 | 8 | 0 | 0 |
| 465.158936 | 465.158862 | 1672169 | 23 | 30 | 0 | 8 | 1 | 0 |
| 465.176597 | 465.176621 | 6171882 | 23 | 30 | 0 | 10 | 0 | 0 |
| 465.191868 | 465.191877 | 7445739 | 27 | 30 | 0 | 7 | 0 | 0 |
| 465.19524 | 465.195248 | 1309675 | 24 | 34 | 0 | 7 | 1 | 0 |
| 465.212973 | 465.213006 | 7528684 | 24 | 34 | 0 | 9 | 0 | 0 |
| 465.228255 | 465.228262 | 8617197 | 28 | 34 | 0 | 6 | 0 | 0 |
| 465.249391 | 465.249392 | 5073134 | 25 | 38 | 0 | 8 | 0 | 0 |
| 465.264683 | 465.264648 | 6259951 | 29 | 38 | 0 | 5 | 0 | 0 |
| 465.285762 | 465.285777 | 2090736 | 26 | 42 | 0 | 7 | 0 | 0 |
| 465.300979 | 465.301033 | 1782257 | 30 | 42 | 0 | 4 | 0 | 0 |
| 467.061986 | 467.061985 | 2061910 | 23 | 16 | 0 | 11 | 0 | 0 |
| 467.077247 | 467.077241 | 1868887 | 27 | 16 | 0 | 8 | 0 | 0 |
| 467.098373 | 467.09837 | 3098968 | 24 | 20 | 0 | 10 | 0 | 0 |
| 467.113661 | 467.113627 | 1632089 | 28 | 20 | 0 | 7 | 0 | 0 |
| 467.134709 | 467.134756 | 3828059 | 25 | 24 | 0 | 9 | 0 | 0 |
| 467.138127 | 467.138127 | 2123099 | 22 | 28 | 0 | 9 | 1 | 0 |
| 467.149998 | 467.150012 | 1321819 | 29 | 24 | 0 | 6 | 0 | 0 |
| 467.155845 | 467.155885 | 2385756 | 22 | 28 | 0 | 11 | 0 | 0 |
| 467.171147 | 467.171141 | 7350621 | 26 | 28 | 0 | 8 | 0 | 0 |
| 467.174521 | 467.174512 | 2048861 | 23 | 32 | 0 | 8 | 1 | 0 |
| 467.186307 | 467.186398 | 1496797 | 30 | 28 | 0 | 5 | 0 | 0 |
| 467.192254 | 467.192271 | 3965790 | 23 | 32 | 0 | 10 | 0 | 0 |
| 467.207539 | 467.207527 | 10484063 | 27 | 32 | 0 | 7 | 0 | 0 |
| 467.211018 | 467.210898 | 1437023 | 24 | 36 | 0 | 7 | 1 | 0 |
| 467.228662 | 467.228656 | 3688800 | 24 | 36 | 0 | 9 | 0 | 0 |
| 467.243921 | 467.243912 | 10922337 | 28 | 36 | 0 | 6 | 0 | 0 |
| 467.256932 | 467.256799 | 1918818 | 25 | 41 | 0 | 6 | 0 | 1 |
| 467.265113 | 467.265042 | 1480290 | 25 | 40 | 0 | 8 | 0 | 0 |
| 467.280289 | 467.280298 | 7298403 | 29 | 40 | 0 | 5 | 0 | 0 |
| 467.31671 | 467.316683 | 1556325 | 30 | 44 | 0 | 4 | 0 | 0 |
| 469.056665 | 469.056506 | 1774027 | 26 | 14 | 0 | 9 | 0 | 0 |
| 469.077587 | 469.077635 | 2394572 | 23 | 18 | 0 | 11 | 0 | 0 |
| 469.092833 | 469.092891 | 2271181 | 27 | 18 | 0 | 8 | 0 | 0 |
| 469.114041 | 469.11402 | 3010510 | 24 | 22 | 0 | 10 | 0 | 0 |
| 469.117426 | 469.117391 | 1238734 | 21 | 26 | 0 | 10 | 1 | 0 |
| 469.129218 | 469.129277 | 1919439 | 28 | 22 | 0 | 7 | 0 | 0 |
| 469.150383 | 469.150406 | 5594576 | 25 | 26 | 0 | 9 | 0 | 0 |
| 469.15373 | 469.153777 | 3043792 | 22 | 30 | 0 | 9 | 1 | 0 |
| 469.165821 | 469.165662 | 1654737 | 29 | 26 | 0 | 6 | 0 | 0 |
| 469.171556 | 469.171535 | 1422801 | 22 | 30 | 0 | 11 | 0 | 0 |
| 469.186808 | 469.186791 | 9957842 | 26 | 30 | 0 | 8 | 0 | 0 |
| 469.190183 | 469.190162 | 2567635 | 23 | 34 | 0 | 8 | 1 | 0 |
| 469.202036 | 469.202048 | 1811155 | 30 | 30 | 0 | 5 | 0 | 0 |
| 469.207995 | 469.207921 | 2129364 | 23 | 34 | 0 | 10 | 0 | 0 |
| 469.223179 | 469.223177 | 13009364 | 27 | 34 | 0 | 7 | 0 | 0 |
| 469.238564 | 469.238433 | 1370709 | 31 | 34 | 0 | 4 | 0 | 0 |
| 469.259575 | 469.259562 | 11629015 | 28 | 38 | 0 | 6 | 0 | 0 |
| 469.295934 | 469.295948 | 7859673 | 29 | 42 | 0 | 5 | 0 | 0 |
| 469.33243 | 469.332334 | 1413595 | 30 | 46 | 0 | 4 | 0 | 0 |
| 471.035783 | 471.03577 | 1488192 | 25 | 12 | 0 | 10 | 0 | 0 |
| 471.072089 | 471.072156 | 2576962 | 26 | 16 | 0 | 9 | 0 | 0 |
| 471.093356 | 471.093285 | 1848899 | 23 | 20 | 0 | 11 | 0 | 0 |
| 471.108474 | 471.108541 | 1915460 | 27 | 20 | 0 | 8 | 0 | 0 |
| 471.129714 | 471.129671 | 3641414 | 24 | 24 | 0 | 10 | 0 | 0 |
| 471.133069 | 471.133041 | 1964102 | 21 | 28 | 0 | 10 | 1 | 0 |
| 471.144943 | 471.144927 | 2630214 | 28 | 24 | 0 | 7 | 0 | 0 |
| 471.166035 | 471.166056 | 7591496 | 25 | 28 | 0 | 9 | 0 | 0 |
| 471.169367 | 471.169427 | 2732104 | 22 | 32 | 0 | 9 | 1 | 0 |
| 471.181311 | 471.181312 | 2527305 | 29 | 28 | 0 | 6 | 0 | 0 |
| 471.202439 | 471.202442 | 12546634 | 26 | 32 | 0 | 8 | 0 | 0 |
| 471.2176 | 471.217698 | 2149451 | 30 | 32 | 0 | 5 | 0 | 0 |
| 471.238825 | 471.238827 | 14248524 | 27 | 36 | 0 | 7 | 0 | 0 |
| 471.253977 | 471.254083 | 1546317 | 31 | 36 | 0 | 4 | 0 | 0 |
| 471.275214 | 471.275213 | 11395662 | 28 | 40 | 0 | 6 | 0 | 0 |
| 471.311591 | 471.311598 | 6606417 | 29 | 44 | 0 | 5 | 0 | 0 |
| 471.347975 | 471.347984 | 2154323 | 30 | 48 | 0 | 4 | 0 | 0 |
| 473.051479 | 473.05142 | 2448057 | 25 | 14 | 0 | 10 | 0 | 0 |
| 473.087758 | 473.087806 | 3048636 | 26 | 18 | 0 | 9 | 0 | 0 |
| 473.109013 | 473.108935 | 2101181 | 23 | 22 | 0 | 11 | 0 | 0 |
| 473.124184 | 473.124191 | 2888894 | 27 | 22 | 0 | 8 | 0 | 0 |
| 473.145325 | 473.145321 | 4785855 | 24 | 26 | 0 | 10 | 0 | 0 |
| 473.148728 | 473.148691 | 1615295 | 21 | 30 | 0 | 10 | 1 | 0 |
| 473.160508 | 473.160577 | 2740928 | 28 | 26 | 0 | 7 | 0 | 0 |
| 473.181699 | 473.181706 | 10111681 | 25 | 30 | 0 | 9 | 0 | 0 |
| 473.18503 | 473.185077 | 1730497 | 22 | 34 | 0 | 9 | 1 | 0 |
| 473.196892 | 473.196962 | 3250882 | 29 | 30 | 0 | 6 | 0 | 0 |
| 473.218078 | 473.218092 | 14695107 | 26 | 34 | 0 | 8 | 0 | 0 |
| 473.233363 | 473.233348 | 3039428 | 30 | 34 | 0 | 5 | 0 | 0 |
| 473.254472 | 473.254477 | 13791942 | 27 | 38 | 0 | 7 | 0 | 0 |
| 473.290889 | 473.290863 | 7615176 | 28 | 42 | 0 | 6 | 0 | 0 |
| 473.327235 | 473.327248 | 4225738 | 29 | 46 | 0 | 5 | 0 | 0 |
| 475.030661 | 475.030685 | 1319475 | 24 | 12 | 0 | 11 | 0 | 0 |
| 475.067065 | 475.06707 | 3314997 | 25 | 16 | 0 | 10 | 0 | 0 |
| 475.10345 | 475.103456 | 3489591 | 26 | 20 | 0 | 9 | 0 | 0 |
| 475.124592 | 475.124585 | 2266936 | 23 | 24 | 0 | 11 | 0 | 0 |
| 475.139788 | 475.139841 | 3646777 | 27 | 24 | 0 | 8 | 0 | 0 |
| 475.160992 | 475.160971 | 6459195 | 24 | 28 | 0 | 10 | 0 | 0 |
| 475.176263 | 475.176227 | 3993404 | 28 | 28 | 0 | 7 | 0 | 0 |
| 475.197348 | 475.197356 | 13988669 | 25 | 32 | 0 | 9 | 0 | 0 |
| 475.212629 | 475.212612 | 4081982 | 29 | 32 | 0 | 6 | 0 | 0 |
| 475.233746 | 475.233742 | 13826879 | 26 | 36 | 0 | 8 | 0 | 0 |
| 475.248927 | 475.248998 | 3010368 | 30 | 36 | 0 | 5 | 0 | 0 |
| 475.270091 | 475.270127 | 9833281 | 27 | 40 | 0 | 7 | 0 | 0 |
| 475.285363 | 475.285383 | 1382466 | 31 | 40 | 0 | 4 | 0 | 0 |
| 475.306553 | 475.306513 | 3516228 | 28 | 44 | 0 | 6 | 0 | 0 |
| 477.046302 | 477.046335 | 2086320 | 24 | 14 | 0 | 11 | 0 | 0 |
| 477.082685 | 477.08272 | 3274163 | 25 | 18 | 0 | 10 | 0 | 0 |
| 477.119134 | 477.119106 | 3632053 | 26 | 22 | 0 | 9 | 0 | 0 |
| 477.140244 | 477.140235 | 2934710 | 23 | 26 | 0 | 11 | 0 | 0 |
| 477.155487 | 477.155491 | 4480952 | 27 | 26 | 0 | 8 | 0 | 0 |
| 477.176622 | 477.176621 | 7721913 | 24 | 30 | 0 | 10 | 0 | 0 |
| 477.19186 | 477.191877 | 5268410 | 28 | 30 | 0 | 7 | 0 | 0 |
| 477.212985 | 477.213006 | 11811771 | 25 | 34 | 0 | 9 | 0 | 0 |
| 477.228286 | 477.228262 | 5000124 | 29 | 34 | 0 | 6 | 0 | 0 |
| 477.249394 | 477.249392 | 8448957 | 26 | 38 | 0 | 8 | 0 | 0 |
| 477.264657 | 477.264648 | 3407806 | 30 | 38 | 0 | 5 | 0 | 0 |
| 477.2858 | 477.285777 | 4019136 | 27 | 42 | 0 | 7 | 0 | 0 |
| 478.16197 | 478.161974 | 1323767 | 25 | 25 | 3 | 7 | 0 | 0 |
| 479.062011 | 479.061985 | 2202161 | 24 | 16 | 0 | 11 | 0 | 0 |
| 479.098343 | 479.09837 | 3268147 | 25 | 20 | 0 | 10 | 0 | 0 |
| 479.113585 | 479.113627 | 1539892 | 29 | 20 | 0 | 7 | 0 | 0 |
| 479.134718 | 479.134756 | 3958326 | 26 | 24 | 0 | 9 | 0 | 0 |
| 479.155842 | 479.155885 | 3174455 | 23 | 28 | 0 | 11 | 0 | 0 |
| 479.17111 | 479.171141 | 5805112 | 27 | 28 | 0 | 8 | 0 | 0 |
| 479.174449 | 479.174512 | 1333304 | 24 | 32 | 0 | 8 | 1 | 0 |
| 479.192251 | 479.192271 | 6986810 | 24 | 32 | 0 | 10 | 0 | 0 |
| 479.207516 | 479.207527 | 7332923 | 28 | 32 | 0 | 7 | 0 | 0 |
| 479.228666 | 479.228656 | 7960636 | 25 | 36 | 0 | 9 | 0 | 0 |
| 479.243947 | 479.243912 | 7138365 | 29 | 36 | 0 | 6 | 0 | 0 |
| 479.265006 | 479.265042 | 4612158 | 26 | 40 | 0 | 8 | 0 | 0 |
| 479.280275 | 479.280298 | 3161151 | 30 | 40 | 0 | 5 | 0 | 0 |
| 479.301385 | 479.301427 | 1625664 | 27 | 44 | 0 | 7 | 0 | 0 |
| 481.041285 | 481.041249 | 1565362 | 23 | 14 | 0 | 12 | 0 | 0 |
| 481.056396 | 481.056506 | 1408179 | 27 | 14 | 0 | 9 | 0 | 0 |
| 481.077662 | 481.077635 | 2527412 | 24 | 18 | 0 | 11 | 0 | 0 |
| 481.092824 | 481.092891 | 2173621 | 28 | 18 | 0 | 8 | 0 | 0 |
| 481.114025 | 481.11402 | 3455671 | 25 | 22 | 0 | 10 | 0 | 0 |
| 481.129303 | 481.129277 | 1536952 | 29 | 22 | 0 | 7 | 0 | 0 |
| 481.1504 | 481.150406 | 5339321 | 26 | 26 | 0 | 9 | 0 | 0 |
| 481.153727 | 481.153777 | 1670585 | 23 | 30 | 0 | 9 | 1 | 0 |
| 481.165588 | 481.165662 | 1484730 | 30 | 26 | 0 | 6 | 0 | 0 |
| 481.17145 | 481.171535 | 2486459 | 23 | 30 | 0 | 11 | 0 | 0 |
| 481.186754 | 481.186791 | 7704764 | 27 | 30 | 0 | 8 | 0 | 0 |
| 481.190233 | 481.190162 | 2437820 | 24 | 34 | 0 | 8 | 1 | 0 |
| 481.202088 | 481.202048 | 2116797 | 31 | 30 | 0 | 5 | 0 | 0 |
| 481.207912 | 481.207921 | 4402365 | 24 | 34 | 0 | 10 | 0 | 0 |
| 481.22318 | 481.223177 | 10378430 | 28 | 34 | 0 | 7 | 0 | 0 |
| 481.244313 | 481.244306 | 3411647 | 25 | 38 | 0 | 9 | 0 | 0 |
| 481.25958 | 481.259562 | 8520896 | 29 | 38 | 0 | 6 | 0 | 0 |
| 481.280672 | 481.280692 | 1783490 | 26 | 42 | 0 | 8 | 0 | 0 |
| 481.295973 | 481.295948 | 3533507 | 30 | 42 | 0 | 5 | 0 | 0 |
| 481.3088 | 481.308835 | 5558468 | 27 | 47 | 0 | 5 | 0 | 1 |
| 483.05695 | 483.0569 | 1690424 | 23 | 16 | 0 | 12 | 0 | 0 |
| 483.072155 | 483.072156 | 2670905 | 27 | 16 | 0 | 9 | 0 | 0 |
| 483.093309 | 483.093285 | 2309435 | 24 | 20 | 0 | 11 | 0 | 0 |
| 483.108511 | 483.108541 | 2348092 | 28 | 20 | 0 | 8 | 0 | 0 |
| 483.129601 | 483.129671 | 3644733 | 25 | 24 | 0 | 10 | 0 | 0 |
| 483.133087 | 483.133041 | 1658429 | 22 | 28 | 0 | 10 | 1 | 0 |
| 483.144916 | 483.144927 | 2249022 | 29 | 24 | 0 | 7 | 0 | 0 |
| 483.166055 | 483.166056 | 6340928 | 26 | 28 | 0 | 9 | 0 | 0 |
| 483.169414 | 483.169427 | 2769728 | 23 | 32 | 0 | 9 | 1 | 0 |
| 483.181313 | 483.181312 | 1997121 | 30 | 28 | 0 | 6 | 0 | 0 |
| 483.187265 | 483.187185 | 1688385 | 23 | 32 | 0 | 11 | 0 | 0 |
| 483.202424 | 483.202442 | 10427714 | 27 | 32 | 0 | 8 | 0 | 0 |
| 483.205802 | 483.205812 | 2455874 | 24 | 36 | 0 | 8 | 1 | 0 |
| 483.21763 | 483.217698 | 1498691 | 31 | 32 | 0 | 5 | 0 | 0 |
| 483.223706 | 483.223571 | 1604931 | 24 | 36 | 0 | 10 | 0 | 0 |
| 483.238846 | 483.238827 | 12643652 | 28 | 36 | 0 | 7 | 0 | 0 |
| 483.260058 | 483.259956 | 1262918 | 25 | 40 | 0 | 9 | 0 | 0 |
| 483.275205 | 483.275213 | 10028359 | 29 | 40 | 0 | 6 | 0 | 0 |
| 483.311688 | 483.311598 | 3795273 | 30 | 44 | 0 | 5 | 0 | 0 |
| 485.051443 | 485.05142 | 1909696 | 26 | 14 | 0 | 10 | 0 | 0 |
| 485.072638 | 485.07255 | 1686978 | 23 | 18 | 0 | 12 | 0 | 0 |
| 485.087827 | 485.087806 | 2848707 | 27 | 18 | 0 | 9 | 0 | 0 |
| 485.108947 | 485.108935 | 2567108 | 24 | 22 | 0 | 11 | 0 | 0 |
| 485.124201 | 485.124191 | 2522053 | 28 | 22 | 0 | 8 | 0 | 0 |
| 485.145333 | 485.145321 | 4425159 | 25 | 26 | 0 | 10 | 0 | 0 |
| 485.148678 | 485.148691 | 2595783 | 22 | 30 | 0 | 10 | 1 | 0 |
| 485.16053 | 485.160577 | 2196424 | 29 | 26 | 0 | 7 | 0 | 0 |
| 485.181675 | 485.181706 | 8826313 | 26 | 30 | 0 | 9 | 0 | 0 |
| 485.185074 | 485.185077 | 2392266 | 23 | 34 | 0 | 9 | 1 | 0 |
| 485.196939 | 485.196962 | 3720650 | 30 | 30 | 0 | 6 | 0 | 0 |
| 485.21807 | 485.218092 | 13446604 | 27 | 34 | 0 | 8 | 0 | 0 |
| 485.23321 | 485.233348 | 2462669 | 31 | 34 | 0 | 5 | 0 | 0 |
| 485.254473 | 485.254477 | 13995470 | 28 | 38 | 0 | 7 | 0 | 0 |
| 485.290865 | 485.290863 | 10874321 | 29 | 42 | 0 | 6 | 0 | 0 |
| 485.32724 | 485.327248 | 4999635 | 30 | 46 | 0 | 5 | 0 | 0 |
| 487.030744 | 487.030685 | 1417547 | 25 | 12 | 0 | 11 | 0 | 0 |
| 487.067113 | 487.06707 | 2960973 | 26 | 16 | 0 | 10 | 0 | 0 |
| 487.103446 | 487.103456 | 3364432 | 27 | 20 | 0 | 9 | 0 | 0 |
| 487.124518 | 487.124585 | 2504273 | 24 | 24 | 0 | 11 | 0 | 0 |
| 487.127835 | 487.127956 | 1479761 | 21 | 28 | 0 | 11 | 1 | 0 |
| 487.139839 | 487.139841 | 2948690 | 28 | 24 | 0 | 8 | 0 | 0 |
| 487.16097 | 487.160971 | 6113876 | 25 | 28 | 0 | 10 | 0 | 0 |
| 487.164266 | 487.164341 | 2667092 | 22 | 32 | 0 | 10 | 1 | 0 |
| 487.176276 | 487.176227 | 3511381 | 29 | 28 | 0 | 7 | 0 | 0 |
| 487.197346 | 487.197356 | 12136022 | 26 | 32 | 0 | 9 | 0 | 0 |
| 487.2126 | 487.212612 | 3349079 | 30 | 32 | 0 | 6 | 0 | 0 |
| 487.23373 | 487.233742 | 15812185 | 27 | 36 | 0 | 8 | 0 | 0 |
| 487.248988 | 487.248998 | 2463322 | 31 | 36 | 0 | 5 | 0 | 0 |
| 487.270108 | 487.270127 | 12793435 | 28 | 40 | 0 | 7 | 0 | 0 |
| 487.306511 | 487.306513 | 8869470 | 29 | 44 | 0 | 6 | 0 | 0 |
| 487.34287 | 487.342898 | 3850849 | 30 | 48 | 0 | 5 | 0 | 0 |
| 489.046225 | 489.046335 | 2175195 | 25 | 14 | 0 | 11 | 0 | 0 |
| 489.082603 | 489.08272 | 3450078 | 26 | 18 | 0 | 10 | 0 | 0 |
| 489.119162 | 489.119106 | 3267808 | 27 | 22 | 0 | 9 | 0 | 0 |
| 489.140181 | 489.140235 | 3271394 | 24 | 26 | 0 | 11 | 0 | 0 |
| 489.143747 | 489.143606 | 1285858 | 21 | 30 | 0 | 11 | 1 | 0 |
| 489.155536 | 489.155491 | 3413219 | 28 | 26 | 0 | 8 | 0 | 0 |
| 489.176619 | 489.176621 | 9293540 | 25 | 30 | 0 | 10 | 0 | 0 |
| 489.180097 | 489.179992 | 1418469 | 22 | 34 | 0 | 10 | 1 | 0 |
| 489.191862 | 489.191877 | 4003558 | 29 | 30 | 0 | 7 | 0 | 0 |
| 489.212972 | 489.213006 | 14530279 | 26 | 34 | 0 | 9 | 0 | 0 |
| 489.228236 | 489.228262 | 3580136 | 30 | 34 | 0 | 6 | 0 | 0 |
| 489.249382 | 489.249392 | 13803242 | 27 | 38 | 0 | 8 | 0 | 0 |
| 489.264639 | 489.264648 | 2353387 | 31 | 38 | 0 | 5 | 0 | 0 |
| 489.285798 | 489.285777 | 9051884 | 28 | 42 | 0 | 7 | 0 | 0 |
| 489.322133 | 489.322163 | 5085935 | 29 | 46 | 0 | 6 | 0 | 0 |
| 491.061973 | 491.061985 | 2450799 | 25 | 16 | 0 | 11 | 0 | 0 |
| 491.098331 | 491.09837 | 2876274 | 26 | 20 | 0 | 10 | 0 | 0 |
| 491.134699 | 491.134756 | 3794804 | 27 | 24 | 0 | 9 | 0 | 0 |
| 491.138229 | 491.138127 | 1508469 | 24 | 28 | 0 | 9 | 1 | 0 |
| 491.155894 | 491.155885 | 3997046 | 24 | 28 | 0 | 11 | 0 | 0 |
| 491.171128 | 491.171141 | 4132727 | 28 | 28 | 0 | 8 | 0 | 0 |
| 491.192266 | 491.192271 | 9516921 | 25 | 32 | 0 | 10 | 0 | 0 |
| 491.207472 | 491.207527 | 4501370 | 29 | 32 | 0 | 7 | 0 | 0 |
| 491.228651 | 491.228656 | 11827067 | 26 | 36 | 0 | 9 | 0 | 0 |
| 491.243874 | 491.243912 | 4067197 | 30 | 36 | 0 | 6 | 0 | 0 |
| 491.265053 | 491.265042 | 9148286 | 27 | 40 | 0 | 8 | 0 | 0 |
| 491.280315 | 491.280298 | 2270079 | 31 | 40 | 0 | 5 | 0 | 0 |
| 491.3014 | 491.301427 | 4409217 | 28 | 44 | 0 | 7 | 0 | 0 |
| 493.077596 | 493.077635 | 2857991 | 25 | 18 | 0 | 11 | 0 | 0 |
| 493.092932 | 493.092891 | 1331208 | 29 | 18 | 0 | 8 | 0 | 0 |
| 493.113932 | 493.11402 | 3057161 | 26 | 22 | 0 | 10 | 0 | 0 |
| 493.135129 | 493.13515 | 1562123 | 23 | 26 | 0 | 12 | 0 | 0 |
| 493.150348 | 493.150406 | 4029452 | 27 | 26 | 0 | 9 | 0 | 0 |
| 493.15356 | 493.153777 | 1449740 | 24 | 30 | 0 | 9 | 1 | 0 |
| 493.171525 | 493.171535 | 4459534 | 24 | 30 | 0 | 11 | 0 | 0 |
| 493.186764 | 493.186791 | 6299663 | 28 | 30 | 0 | 8 | 0 | 0 |
| 493.190151 | 493.190162 | 1277711 | 25 | 34 | 0 | 8 | 1 | 0 |
| 493.207894 | 493.207921 | 7961617 | 25 | 34 | 0 | 10 | 0 | 0 |
| 493.223191 | 493.223177 | 6254610 | 29 | 34 | 0 | 7 | 0 | 0 |
| 493.244334 | 493.244306 | 6883347 | 26 | 38 | 0 | 9 | 0 | 0 |
| 493.259528 | 493.259562 | 4903957 | 30 | 38 | 0 | 6 | 0 | 0 |
| 493.280635 | 493.280692 | 3893782 | 27 | 42 | 0 | 8 | 0 | 0 |
| 493.295987 | 493.295948 | 1594391 | 31 | 42 | 0 | 5 | 0 | 0 |
| 493.372181 | 493.37209 | 1776925 | 30 | 54 | 0 | 3 | 1 | 0 |
| 495.056906 | 495.0569 | 1789598 | 24 | 16 | 0 | 12 | 0 | 0 |
| 495.072095 | 495.072156 | 1393823 | 28 | 16 | 0 | 9 | 0 | 0 |
| 495.093297 | 495.093285 | 2653345 | 25 | 20 | 0 | 11 | 0 | 0 |
| 495.108597 | 495.108541 | 1797282 | 29 | 20 | 0 | 8 | 0 | 0 |
| 495.129617 | 495.129671 | 3590820 | 26 | 24 | 0 | 10 | 0 | 0 |
| 495.133012 | 495.133041 | 1581476 | 23 | 28 | 0 | 10 | 1 | 0 |
| 495.144894 | 495.144927 | 1379493 | 30 | 24 | 0 | 7 | 0 | 0 |
| 495.150769 | 495.1508 | 1432486 | 23 | 28 | 0 | 12 | 0 | 0 |
| 495.166015 | 495.166056 | 5240999 | 27 | 28 | 0 | 9 | 0 | 0 |
| 495.169459 | 495.169427 | 2451623 | 24 | 32 | 0 | 9 | 1 | 0 |
| 495.187146 | 495.187185 | 3310248 | 24 | 32 | 0 | 11 | 0 | 0 |
| 495.202412 | 495.202442 | 8161450 | 28 | 32 | 0 | 8 | 0 | 0 |
| 495.2059 | 495.205812 | 1821098 | 25 | 36 | 0 | 8 | 1 | 0 |
| 495.223553 | 495.223571 | 4549803 | 25 | 36 | 0 | 10 | 0 | 0 |
| 495.238814 | 495.238827 | 8652972 | 29 | 36 | 0 | 7 | 0 | 0 |
| 495.259872 | 495.259956 | 2891438 | 26 | 40 | 0 | 9 | 0 | 0 |
| 495.275163 | 495.275213 | 5019823 | 30 | 40 | 0 | 6 | 0 | 0 |
| 495.288098 | 495.288099 | 2961584 | 27 | 45 | 0 | 6 | 0 | 1 |
| 495.296341 | 495.296342 | 1550257 | 27 | 44 | 0 | 8 | 0 | 0 |
| 497.051352 | 497.05142 | 1307194 | 27 | 14 | 0 | 10 | 0 | 0 |
| 497.072542 | 497.07255 | 1739579 | 24 | 18 | 0 | 12 | 0 | 0 |
| 497.087787 | 497.087806 | 2326845 | 28 | 18 | 0 | 9 | 0 | 0 |
| 497.10894 | 497.108935 | 2394942 | 25 | 22 | 0 | 11 | 0 | 0 |
| 497.124115 | 497.124191 | 2297920 | 29 | 22 | 0 | 8 | 0 | 0 |
| 497.145318 | 497.145321 | 4231489 | 26 | 26 | 0 | 10 | 0 | 0 |
| 497.148653 | 497.148691 | 1960257 | 23 | 30 | 0 | 10 | 1 | 0 |
| 497.160554 | 497.160577 | 2048834 | 30 | 26 | 0 | 7 | 0 | 0 |
| 497.181675 | 497.181706 | 6919492 | 27 | 30 | 0 | 9 | 0 | 0 |
| 497.184993 | 497.185077 | 2804548 | 24 | 34 | 0 | 9 | 1 | 0 |
| 497.197128 | 497.196962 | 1619781 | 31 | 30 | 0 | 6 | 0 | 0 |
| 497.202874 | 497.202835 | 1662022 | 24 | 34 | 0 | 11 | 0 | 0 |
| 497.218055 | 497.218092 | 10205511 | 28 | 34 | 0 | 8 | 0 | 0 |
| 497.239308 | 497.239221 | 1800009 | 25 | 38 | 0 | 10 | 0 | 0 |
| 497.254457 | 497.254477 | 11133258 | 29 | 38 | 0 | 7 | 0 | 0 |
| 497.290825 | 497.290863 | 6041933 | 30 | 42 | 0 | 6 | 0 | 0 |
| 498.289443 | 498.289482 | 2130331 | 26 | 45 | 1 | 6 | 1 | 0 |
| 499.030637 | 499.030685 | 1261782 | 26 | 12 | 0 | 11 | 0 | 0 |
| 499.066891 | 499.06707 | 2190553 | 27 | 16 | 0 | 10 | 0 | 0 |
| 499.088258 | 499.0882 | 1789915 | 24 | 20 | 0 | 12 | 0 | 0 |
| 499.103493 | 499.103456 | 2740700 | 28 | 20 | 0 | 9 | 0 | 0 |
| 499.124641 | 499.124585 | 2808798 | 25 | 24 | 0 | 11 | 0 | 0 |
| 499.128063 | 499.127956 | 1420766 | 22 | 28 | 0 | 11 | 1 | 0 |
| 499.139808 | 499.139841 | 2431455 | 29 | 24 | 0 | 8 | 0 | 0 |
| 499.160955 | 499.160971 | 5166561 | 26 | 28 | 0 | 10 | 0 | 0 |
| 499.164319 | 499.164341 | 2572769 | 23 | 32 | 0 | 10 | 1 | 0 |
| 499.176202 | 499.176227 | 2098658 | 30 | 28 | 0 | 7 | 0 | 0 |
| 499.19731 | 499.197356 | 9908707 | 27 | 32 | 0 | 9 | 0 | 0 |
| 499.200701 | 499.200727 | 2553828 | 24 | 36 | 0 | 9 | 1 | 0 |
| 499.212602 | 499.212612 | 2211813 | 31 | 32 | 0 | 6 | 0 | 0 |
| 499.233705 | 499.233742 | 13064678 | 28 | 36 | 0 | 8 | 0 | 0 |
| 499.248837 | 499.248998 | 1586920 | 32 | 36 | 0 | 5 | 0 | 0 |
| 499.270116 | 499.270127 | 12667369 | 29 | 40 | 0 | 7 | 0 | 0 |
| 499.306519 | 499.306513 | 7469548 | 30 | 44 | 0 | 6 | 0 | 0 |
| 501.04629 | 501.046335 | 1759863 | 26 | 14 | 0 | 11 | 0 | 0 |
| 501.082696 | 501.08272 | 2654330 | 27 | 18 | 0 | 10 | 0 | 0 |
| 501.103871 | 501.10385 | 1894012 | 24 | 22 | 0 | 12 | 0 | 0 |
| 501.119092 | 501.119106 | 2879101 | 28 | 22 | 0 | 9 | 0 | 0 |
| 501.14021 | 501.140235 | 2997887 | 25 | 26 | 0 | 11 | 0 | 0 |
| 501.143543 | 501.143606 | 1591679 | 22 | 30 | 0 | 11 | 1 | 0 |
| 501.155349 | 501.155491 | 2848896 | 29 | 26 | 0 | 8 | 0 | 0 |
| 501.176583 | 501.176621 | 7438978 | 26 | 30 | 0 | 10 | 0 | 0 |
| 501.179898 | 501.179992 | 1456002 | 23 | 34 | 0 | 10 | 1 | 0 |
| 501.191941 | 501.191877 | 2767491 | 30 | 30 | 0 | 7 | 0 | 0 |
| 501.213002 | 501.213006 | 12475013 | 27 | 34 | 0 | 9 | 0 | 0 |
| 501.216434 | 501.216377 | 1488005 | 24 | 38 | 0 | 9 | 1 | 0 |
| 501.228258 | 501.228262 | 2500230 | 31 | 34 | 0 | 6 | 0 | 0 |
| 501.249376 | 501.249392 | 14979720 | 28 | 38 | 0 | 8 | 0 | 0 |
| 501.264607 | 501.264648 | 1889417 | 32 | 38 | 0 | 5 | 0 | 0 |
| 501.285769 | 501.285777 | 12862091 | 29 | 42 | 0 | 7 | 0 | 0 |
| 501.322145 | 501.322163 | 10150541 | 30 | 46 | 0 | 6 | 0 | 0 |
| 503.062044 | 503.061985 | 1999130 | 26 | 16 | 0 | 11 | 0 | 0 |
| 503.098399 | 503.09837 | 2756893 | 27 | 20 | 0 | 10 | 0 | 0 |
| 503.119868 | 503.1195 | 1456927 | 24 | 24 | 0 | 12 | 0 | 0 |
| 503.13474 | 503.134756 | 3117344 | 28 | 24 | 0 | 9 | 0 | 0 |
| 503.155867 | 503.155885 | 4294434 | 25 | 28 | 0 | 11 | 0 | 0 |
| 503.17119 | 503.171141 | 3011363 | 29 | 28 | 0 | 8 | 0 | 0 |
| 503.192271 | 503.192271 | 9810724 | 26 | 32 | 0 | 10 | 0 | 0 |
| 503.207495 | 503.207527 | 3278630 | 30 | 32 | 0 | 7 | 0 | 0 |
| 503.228629 | 503.228656 | 14398247 | 27 | 36 | 0 | 9 | 0 | 0 |
| 503.243801 | 503.243912 | 3318569 | 31 | 36 | 0 | 6 | 0 | 0 |
| 503.265001 | 503.265042 | 13179690 | 28 | 40 | 0 | 8 | 0 | 0 |
| 503.280381 | 503.280298 | 1483564 | 32 | 40 | 0 | 5 | 0 | 0 |
| 503.301402 | 503.301427 | 9274157 | 29 | 44 | 0 | 7 | 0 | 0 |
| 503.337845 | 503.337813 | 6140720 | 30 | 48 | 0 | 6 | 0 | 0 |
| 505.041113 | 505.041249 | 1276347 | 25 | 14 | 0 | 12 | 0 | 0 |
| 505.077505 | 505.077635 | 2450366 | 26 | 18 | 0 | 11 | 0 | 0 |
| 505.113981 | 505.11402 | 3540417 | 27 | 22 | 0 | 10 | 0 | 0 |
| 505.135168 | 505.13515 | 1676994 | 24 | 26 | 0 | 12 | 0 | 0 |
| 505.150355 | 505.150406 | 3828676 | 28 | 26 | 0 | 9 | 0 | 0 |
| 505.171515 | 505.171535 | 5654469 | 25 | 30 | 0 | 11 | 0 | 0 |
| 505.186779 | 505.186791 | 3910599 | 29 | 30 | 0 | 8 | 0 | 0 |
| 505.207884 | 505.207921 | 10654664 | 26 | 34 | 0 | 10 | 0 | 0 |
| 505.223171 | 505.223177 | 4397002 | 30 | 34 | 0 | 7 | 0 | 0 |
| 505.244281 | 505.244306 | 11129803 | 27 | 38 | 0 | 9 | 0 | 0 |
| 505.259569 | 505.259562 | 3149773 | 31 | 38 | 0 | 6 | 0 | 0 |
| 505.280673 | 505.280692 | 7883726 | 28 | 42 | 0 | 8 | 0 | 0 |
| 505.295869 | 505.295948 | 1548495 | 32 | 42 | 0 | 5 | 0 | 0 |
| 505.317082 | 505.317077 | 4292049 | 29 | 46 | 0 | 7 | 0 | 0 |
| 507.056865 | 507.0569 | 1565535 | 25 | 16 | 0 | 12 | 0 | 0 |
| 507.072127 | 507.072156 | 1318496 | 29 | 16 | 0 | 9 | 0 | 0 |
| 507.093283 | 507.093285 | 2673762 | 26 | 20 | 0 | 11 | 0 | 0 |
| 507.108603 | 507.108541 | 1363811 | 30 | 20 | 0 | 8 | 0 | 0 |
| 507.129631 | 507.129671 | 3373157 | 27 | 24 | 0 | 10 | 0 | 0 |
| 507.144901 | 507.144927 | 1271142 | 31 | 24 | 0 | 7 | 0 | 0 |
| 507.150817 | 507.1508 | 2081383 | 24 | 28 | 0 | 12 | 0 | 0 |
| 507.165953 | 507.166056 | 4417640 | 28 | 28 | 0 | 9 | 0 | 0 |
| 507.169508 | 507.169427 | 1633640 | 25 | 32 | 0 | 9 | 1 | 0 |
| 507.187144 | 507.187185 | 5386346 | 25 | 32 | 0 | 11 | 0 | 0 |
| 507.20244 | 507.202442 | 5170283 | 29 | 32 | 0 | 8 | 0 | 0 |
| 507.223536 | 507.223571 | 8213613 | 26 | 36 | 0 | 10 | 0 | 0 |
| 507.23881 | 507.238827 | 4788334 | 30 | 36 | 0 | 7 | 0 | 0 |
| 507.259945 | 507.259956 | 6017136 | 27 | 40 | 0 | 9 | 0 | 0 |
| 507.275178 | 507.275213 | 3082865 | 31 | 40 | 0 | 6 | 0 | 0 |
| 507.296345 | 507.296342 | 3112051 | 28 | 44 | 0 | 8 | 0 | 0 |
| 507.332637 | 507.332727 | 1343606 | 29 | 48 | 0 | 7 | 0 | 0 |
| 509.051274 | 509.05142 | 1461250 | 28 | 14 | 0 | 10 | 0 | 0 |
| 509.072608 | 509.07255 | 1696004 | 25 | 18 | 0 | 12 | 0 | 0 |
| 509.087754 | 509.087806 | 1574405 | 29 | 18 | 0 | 9 | 0 | 0 |
| 509.108921 | 509.108935 | 2579719 | 26 | 22 | 0 | 11 | 0 | 0 |
| 509.12414 | 509.124191 | 1527304 | 30 | 22 | 0 | 8 | 0 | 0 |
| 509.14532 | 509.145321 | 3844362 | 27 | 26 | 0 | 10 | 0 | 0 |
| 509.14867 | 509.148691 | 1458186 | 24 | 30 | 0 | 10 | 1 | 0 |
| 509.160591 | 509.160577 | 1453067 | 31 | 26 | 0 | 7 | 0 | 0 |
| 509.166562 | 509.16645 | 2033163 | 24 | 30 | 0 | 12 | 0 | 0 |
| 509.18167 | 509.181706 | 5362957 | 28 | 30 | 0 | 9 | 0 | 0 |
| 509.18527 | 509.185077 | 1893645 | 25 | 34 | 0 | 9 | 1 | 0 |
| 509.202781 | 509.202835 | 4406542 | 25 | 34 | 0 | 11 | 0 | 0 |
| 509.218085 | 509.218092 | 6916368 | 29 | 34 | 0 | 8 | 0 | 0 |
| 509.221241 | 509.221462 | 1376016 | 26 | 38 | 0 | 8 | 1 | 0 |
| 509.239227 | 509.239221 | 4146449 | 26 | 38 | 0 | 10 | 0 | 0 |
| 509.254451 | 509.254477 | 5618963 | 30 | 38 | 0 | 7 | 0 | 0 |
| 509.275569 | 509.275606 | 2747668 | 27 | 42 | 0 | 9 | 0 | 0 |
| 509.290894 | 509.290863 | 2549014 | 31 | 42 | 0 | 6 | 0 | 0 |
| 511.067046 | 511.06707 | 1884582 | 28 | 16 | 0 | 10 | 0 | 0 |
| 511.08821 | 511.0882 | 1861800 | 25 | 20 | 0 | 12 | 0 | 0 |
| 511.103694 | 511.103456 | 1555625 | 29 | 20 | 0 | 9 | 0 | 0 |
| 511.124518 | 511.124585 | 2649003 | 26 | 24 | 0 | 11 | 0 | 0 |
| 511.139895 | 511.139841 | 1911980 | 30 | 24 | 0 | 8 | 0 | 0 |
| 511.160964 | 511.160971 | 4359598 | 27 | 28 | 0 | 10 | 0 | 0 |
| 511.164273 | 511.164341 | 2350510 | 24 | 32 | 0 | 10 | 1 | 0 |
| 511.176285 | 511.176227 | 1414831 | 31 | 28 | 0 | 7 | 0 | 0 |
| 511.182003 | 511.1821 | 1427888 | 24 | 32 | 0 | 12 | 0 | 0 |
| 511.197318 | 511.197356 | 6690225 | 28 | 32 | 0 | 9 | 0 | 0 |
| 511.200677 | 511.200727 | 2436529 | 25 | 36 | 0 | 9 | 1 | 0 |
| 511.21849 | 511.218486 | 2079155 | 25 | 36 | 0 | 11 | 0 | 0 |
| 511.233714 | 511.233742 | 9256372 | 29 | 36 | 0 | 8 | 0 | 0 |
| 511.254893 | 511.254871 | 1740726 | 26 | 40 | 0 | 10 | 0 | 0 |
| 511.270087 | 511.270127 | 6561207 | 30 | 40 | 0 | 7 | 0 | 0 |
| 511.306469 | 511.306513 | 2232762 | 31 | 44 | 0 | 6 | 0 | 0 |
| 513.046265 | 513.046335 | 1476680 | 27 | 14 | 0 | 11 | 0 | 0 |
| 513.082827 | 513.08272 | 2515019 | 28 | 18 | 0 | 10 | 0 | 0 |
| 513.103753 | 513.10385 | 1905228 | 25 | 22 | 0 | 12 | 0 | 0 |
| 513.119107 | 513.119106 | 2713166 | 29 | 22 | 0 | 9 | 0 | 0 |
| 513.140293 | 513.140235 | 2751567 | 26 | 26 | 0 | 11 | 0 | 0 |
| 513.143404 | 513.143606 | 1363279 | 23 | 30 | 0 | 11 | 1 | 0 |
| 513.155512 | 513.155491 | 1986128 | 30 | 26 | 0 | 8 | 0 | 0 |
| 513.176631 | 513.176621 | 6000210 | 27 | 30 | 0 | 10 | 0 | 0 |
| 513.180065 | 513.179992 | 2469971 | 24 | 34 | 0 | 10 | 1 | 0 |
| 513.191857 | 513.191877 | 1914451 | 31 | 30 | 0 | 7 | 0 | 0 |
| 513.213001 | 513.213006 | 9674325 | 28 | 34 | 0 | 9 | 0 | 0 |
| 513.216297 | 513.216377 | 1885525 | 25 | 38 | 0 | 9 | 1 | 0 |
| 513.22823 | 513.228262 | 2024534 | 32 | 34 | 0 | 6 | 0 | 0 |
| 513.249349 | 513.249392 | 12406360 | 29 | 38 | 0 | 8 | 0 | 0 |
| 513.264645 | 513.264648 | 1331801 | 33 | 38 | 0 | 5 | 0 | 0 |
| 513.285765 | 513.285777 | 8785499 | 30 | 42 | 0 | 7 | 0 | 0 |
| 513.322206 | 513.322163 | 1728862 | 31 | 46 | 0 | 6 | 0 | 0 |
| 514.284336 | 514.284397 | 4788908 | 26 | 45 | 1 | 7 | 1 | 0 |
| 515.061992 | 515.061985 | 1786347 | 27 | 16 | 0 | 11 | 0 | 0 |
| 515.098216 | 515.09837 | 2493422 | 28 | 20 | 0 | 10 | 0 | 0 |
| 515.119599 | 515.1195 | 1555951 | 25 | 24 | 0 | 12 | 0 | 0 |
| 515.134749 | 515.134756 | 2853617 | 29 | 24 | 0 | 9 | 0 | 0 |
| 515.155853 | 515.155885 | 3636466 | 26 | 28 | 0 | 11 | 0 | 0 |
| 515.171161 | 515.171141 | 2215156 | 30 | 28 | 0 | 8 | 0 | 0 |
| 515.192222 | 515.192271 | 8335093 | 27 | 32 | 0 | 10 | 0 | 0 |
| 515.195584 | 515.195642 | 2063606 | 24 | 36 | 0 | 10 | 1 | 0 |
| 515.207499 | 515.207527 | 2211319 | 31 | 32 | 0 | 7 | 0 | 0 |
| 515.228627 | 515.228656 | 12959480 | 28 | 36 | 0 | 9 | 0 | 0 |
| 515.243864 | 515.243912 | 1749753 | 32 | 36 | 0 | 6 | 0 | 0 |
| 515.265031 | 515.265042 | 13862651 | 29 | 40 | 0 | 8 | 0 | 0 |
| 515.301416 | 515.301427 | 9617150 | 30 | 44 | 0 | 7 | 0 | 0 |
| 517.041179 | 517.041249 | 1382282 | 26 | 14 | 0 | 12 | 0 | 0 |
| 517.077611 | 517.077635 | 2291085 | 27 | 18 | 0 | 11 | 0 | 0 |
| 517.113916 | 517.11402 | 2520976 | 28 | 22 | 0 | 10 | 0 | 0 |
| 517.135124 | 517.13515 | 2175889 | 25 | 26 | 0 | 12 | 0 | 0 |
| 517.150368 | 517.150406 | 2774931 | 29 | 26 | 0 | 9 | 0 | 0 |
| 517.171496 | 517.171535 | 5191572 | 26 | 30 | 0 | 11 | 0 | 0 |
| 517.174719 | 517.174906 | 1385108 | 23 | 34 | 0 | 11 | 1 | 0 |
| 517.186867 | 517.186791 | 3043221 | 30 | 30 | 0 | 8 | 0 | 0 |
| 517.207889 | 517.207921 | 10831767 | 27 | 34 | 0 | 10 | 0 | 0 |
| 517.223197 | 517.223177 | 3002776 | 31 | 34 | 0 | 7 | 0 | 0 |
| 517.244282 | 517.244306 | 13281178 | 28 | 38 | 0 | 9 | 0 | 0 |
| 517.259529 | 517.259562 | 2076059 | 32 | 38 | 0 | 6 | 0 | 0 |
| 517.280649 | 517.280692 | 12543901 | 29 | 42 | 0 | 8 | 0 | 0 |
| 517.317044 | 517.317077 | 16353184 | 30 | 46 | 0 | 7 | 0 | 0 |
| 519.056963 | 519.0569 | 1547818 | 26 | 16 | 0 | 12 | 0 | 0 |
| 519.093319 | 519.093285 | 2695213 | 27 | 20 | 0 | 11 | 0 | 0 |
| 519.129685 | 519.129671 | 2947120 | 28 | 24 | 0 | 10 | 0 | 0 |
| 519.150772 | 519.1508 | 2545714 | 25 | 28 | 0 | 12 | 0 | 0 |
| 519.16602 | 519.166056 | 3702835 | 29 | 28 | 0 | 9 | 0 | 0 |
| 519.187078 | 519.187185 | 6468661 | 26 | 32 | 0 | 11 | 0 | 0 |
| 519.202362 | 519.202442 | 3040822 | 30 | 32 | 0 | 8 | 0 | 0 |
| 519.205709 | 519.205812 | 1337910 | 27 | 36 | 0 | 8 | 1 | 0 |
| 519.223546 | 519.223571 | 10543159 | 27 | 36 | 0 | 10 | 0 | 0 |
| 519.238788 | 519.238827 | 3126840 | 31 | 36 | 0 | 7 | 0 | 0 |
| 519.259904 | 519.259956 | 10614842 | 28 | 40 | 0 | 9 | 0 | 0 |
| 519.275196 | 519.275213 | 2148667 | 32 | 40 | 0 | 6 | 0 | 0 |
| 519.296317 | 519.296342 | 7972925 | 29 | 44 | 0 | 8 | 0 | 0 |
| 519.332723 | 519.332727 | 8712256 | 30 | 48 | 0 | 7 | 0 | 0 |
| 521.072462 | 521.07255 | 1364168 | 26 | 18 | 0 | 12 | 0 | 0 |
| 521.08781 | 521.087806 | 1252681 | 30 | 18 | 0 | 9 | 0 | 0 |
| 521.108942 | 521.108935 | 3017931 | 27 | 22 | 0 | 11 | 0 | 0 |
| 521.124164 | 521.124191 | 1864396 | 31 | 22 | 0 | 8 | 0 | 0 |
| 521.145216 | 521.145321 | 3620046 | 28 | 26 | 0 | 10 | 0 | 0 |
| 521.148609 | 521.148691 | 1518286 | 25 | 30 | 0 | 10 | 1 | 0 |
| 521.166467 | 521.16645 | 2791120 | 25 | 30 | 0 | 12 | 0 | 0 |
| 521.18166 | 521.181706 | 3760337 | 29 | 30 | 0 | 9 | 0 | 0 |
| 521.185204 | 521.185077 | 1911505 | 26 | 34 | 0 | 9 | 1 | 0 |
| 521.202828 | 521.202835 | 5999827 | 26 | 34 | 0 | 11 | 0 | 0 |
| 521.218106 | 521.218092 | 4776148 | 30 | 34 | 0 | 8 | 0 | 0 |
| 521.221496 | 521.221462 | 1511380 | 27 | 38 | 0 | 8 | 1 | 0 |
| 521.239195 | 521.239221 | 7704789 | 27 | 38 | 0 | 10 | 0 | 0 |
| 521.254509 | 521.254477 | 3898583 | 31 | 38 | 0 | 7 | 0 | 0 |
| 521.275523 | 521.275606 | 5257432 | 28 | 42 | 0 | 9 | 0 | 0 |
| 521.290821 | 521.290863 | 2142937 | 32 | 42 | 0 | 6 | 0 | 0 |
| 521.311975 | 521.311992 | 3250395 | 29 | 46 | 0 | 8 | 0 | 0 |
| 521.348396 | 521.348378 | 1315038 | 30 | 50 | 0 | 7 | 0 | 0 |
| 523.088206 | 523.0882 | 1739620 | 26 | 20 | 0 | 12 | 0 | 0 |
| 523.103424 | 523.103456 | 1659749 | 30 | 20 | 0 | 9 | 0 | 0 |
| 523.124562 | 523.124585 | 2622823 | 27 | 24 | 0 | 11 | 0 | 0 |
| 523.160955 | 523.160971 | 3456362 | 28 | 28 | 0 | 10 | 0 | 0 |
| 523.164345 | 523.164341 | 1742954 | 25 | 32 | 0 | 10 | 1 | 0 |
| 523.182063 | 523.1821 | 2751339 | 25 | 32 | 0 | 12 | 0 | 0 |
| 523.197295 | 523.197356 | 4871533 | 29 | 32 | 0 | 9 | 0 | 0 |
| 523.200649 | 523.200727 | 1680237 | 26 | 36 | 0 | 9 | 1 | 0 |
| 523.212636 | 523.212612 | 1267310 | 33 | 32 | 0 | 6 | 0 | 0 |
| 523.218473 | 523.218486 | 4488558 | 26 | 36 | 0 | 11 | 0 | 0 |
| 523.233775 | 523.233742 | 5359983 | 30 | 36 | 0 | 8 | 0 | 0 |
| 523.23716 | 523.237112 | 1448047 | 27 | 40 | 0 | 8 | 1 | 0 |
| 523.254911 | 523.254871 | 4090225 | 27 | 40 | 0 | 10 | 0 | 0 |
| 523.27004 | 523.270127 | 2953074 | 31 | 40 | 0 | 7 | 0 | 0 |
| 523.291261 | 523.291257 | 2190196 | 28 | 44 | 0 | 9 | 0 | 0 |
| 523.306654 | 523.306513 | 1604981 | 32 | 44 | 0 | 6 | 0 | 0 |
| 525.082621 | 525.08272 | 1833467 | 29 | 18 | 0 | 10 | 0 | 0 |
| 525.103766 | 525.10385 | 1909501 | 26 | 22 | 0 | 12 | 0 | 0 |
| 525.118992 | 525.119106 | 1842174 | 30 | 22 | 0 | 9 | 0 | 0 |
| 525.140193 | 525.140235 | 3038208 | 27 | 26 | 0 | 11 | 0 | 0 |
| 525.15544 | 525.155491 | 1518337 | 31 | 26 | 0 | 8 | 0 | 0 |
| 525.176526 | 525.176621 | 4605443 | 28 | 30 | 0 | 10 | 0 | 0 |
| 525.179938 | 525.179992 | 2263043 | 25 | 34 | 0 | 10 | 1 | 0 |
| 525.191806 | 525.191877 | 1380100 | 32 | 30 | 0 | 7 | 0 | 0 |
| 525.197895 | 525.19775 | 1254916 | 25 | 34 | 0 | 12 | 0 | 0 |
| 525.212997 | 525.213006 | 5985797 | 29 | 34 | 0 | 9 | 0 | 0 |
| 525.216278 | 525.216377 | 1750021 | 26 | 38 | 0 | 9 | 1 | 0 |
| 525.234122 | 525.234136 | 2053383 | 26 | 38 | 0 | 11 | 0 | 0 |
| 525.249354 | 525.249392 | 6446600 | 30 | 38 | 0 | 8 | 0 | 0 |
| 525.270488 | 525.270521 | 1812234 | 27 | 42 | 0 | 10 | 0 | 0 |
| 525.285698 | 525.285777 | 3319819 | 31 | 42 | 0 | 7 | 0 | 0 |
| 525.322047 | 525.322163 | 1285389 | 32 | 46 | 0 | 6 | 0 | 0 |
| 527.062003 | 527.061985 | 1571983 | 28 | 16 | 0 | 11 | 0 | 0 |
| 527.098338 | 527.09837 | 2138257 | 29 | 20 | 0 | 10 | 0 | 0 |
| 527.119598 | 527.1195 | 1756563 | 26 | 24 | 0 | 12 | 0 | 0 |
| 527.134733 | 527.134756 | 2636948 | 30 | 24 | 0 | 9 | 0 | 0 |
| 527.155892 | 527.155885 | 3237525 | 27 | 28 | 0 | 11 | 0 | 0 |
| 527.159208 | 527.159256 | 1379734 | 24 | 32 | 0 | 11 | 1 | 0 |
| 527.171106 | 527.171141 | 1837719 | 31 | 28 | 0 | 8 | 0 | 0 |
| 527.192259 | 527.192271 | 5796504 | 28 | 32 | 0 | 10 | 0 | 0 |
| 527.195654 | 527.195642 | 2556056 | 25 | 36 | 0 | 10 | 1 | 0 |
| 527.228638 | 527.228656 | 9364123 | 29 | 36 | 0 | 9 | 0 | 0 |
| 527.243911 | 527.243912 | 1500828 | 33 | 36 | 0 | 6 | 0 | 0 |
| 527.265032 | 527.265042 | 8557213 | 30 | 40 | 0 | 8 | 0 | 0 |
| 527.301347 | 527.301427 | 3300512 | 31 | 44 | 0 | 7 | 0 | 0 |
| 529.077521 | 529.077635 | 2247457 | 28 | 18 | 0 | 11 | 0 | 0 |
| 529.09879 | 529.098764 | 1364258 | 25 | 22 | 0 | 13 | 0 | 0 |
| 529.113963 | 529.11402 | 2252579 | 29 | 22 | 0 | 10 | 0 | 0 |
| 529.135055 | 529.13515 | 1732901 | 26 | 26 | 0 | 12 | 0 | 0 |
| 529.150347 | 529.150406 | 2303270 | 30 | 26 | 0 | 9 | 0 | 0 |
| 529.171501 | 529.171535 | 3909416 | 27 | 30 | 0 | 11 | 0 | 0 |
| 529.174864 | 529.174906 | 2209576 | 24 | 34 | 0 | 11 | 1 | 0 |
| 529.186787 | 529.186791 | 2204457 | 31 | 30 | 0 | 8 | 0 | 0 |
| 529.207885 | 529.207921 | 8397610 | 28 | 34 | 0 | 10 | 0 | 0 |
| 529.223222 | 529.223177 | 2030379 | 32 | 34 | 0 | 7 | 0 | 0 |
| 529.244262 | 529.244306 | 11424557 | 29 | 38 | 0 | 9 | 0 | 0 |
| 529.259484 | 529.259562 | 1752878 | 33 | 38 | 0 | 6 | 0 | 0 |
| 529.280679 | 529.280692 | 10076975 | 30 | 42 | 0 | 8 | 0 | 0 |
| 529.317124 | 529.317077 | 2769714 | 31 | 46 | 0 | 7 | 0 | 0 |
| 531.056901 | 531.0569 | 1598124 | 27 | 16 | 0 | 12 | 0 | 0 |
| 531.093296 | 531.093285 | 1615407 | 28 | 20 | 0 | 11 | 0 | 0 |
| 531.129618 | 531.129671 | 2297266 | 29 | 24 | 0 | 10 | 0 | 0 |
| 531.150819 | 531.1508 | 1993139 | 26 | 28 | 0 | 12 | 0 | 0 |
| 531.166143 | 531.166056 | 2448820 | 30 | 28 | 0 | 9 | 0 | 0 |
| 531.187156 | 531.187185 | 5831606 | 27 | 32 | 0 | 11 | 0 | 0 |
| 531.202422 | 531.202442 | 2466743 | 31 | 32 | 0 | 8 | 0 | 0 |
| 531.223554 | 531.223571 | 10210232 | 28 | 36 | 0 | 10 | 0 | 0 |
| 531.23884 | 531.238827 | 2451897 | 32 | 36 | 0 | 7 | 0 | 0 |
| 531.259962 | 531.259956 | 12622778 | 29 | 40 | 0 | 9 | 0 | 0 |
| 531.275285 | 531.275213 | 1883580 | 33 | 40 | 0 | 6 | 0 | 0 |
| 531.296325 | 531.296342 | 9237437 | 30 | 44 | 0 | 8 | 0 | 0 |
| 531.311596 | 531.311598 | 1305790 | 34 | 44 | 0 | 5 | 0 | 0 |
| 531.33269 | 531.332727 | 1969856 | 31 | 48 | 0 | 7 | 0 | 0 |
| 533.072509 | 533.07255 | 2016310 | 27 | 18 | 0 | 12 | 0 | 0 |
| 533.108946 | 533.108935 | 2418745 | 28 | 22 | 0 | 11 | 0 | 0 |
| 533.145334 | 533.145321 | 2279739 | 29 | 26 | 0 | 10 | 0 | 0 |
| 533.166452 | 533.16645 | 2576957 | 26 | 30 | 0 | 12 | 0 | 0 |
| 533.181611 | 533.181706 | 3146814 | 30 | 30 | 0 | 9 | 0 | 0 |
| 533.202789 | 533.202835 | 6681663 | 27 | 34 | 0 | 11 | 0 | 0 |
| 533.218042 | 533.218092 | 3318848 | 31 | 34 | 0 | 8 | 0 | 0 |
| 533.221537 | 533.221462 | 1416768 | 28 | 38 | 0 | 8 | 1 | 0 |
| 533.239212 | 533.239221 | 10058817 | 28 | 38 | 0 | 10 | 0 | 0 |
| 533.254411 | 533.254477 | 2815555 | 32 | 38 | 0 | 7 | 0 | 0 |
| 533.275606 | 533.275606 | 9163844 | 29 | 42 | 0 | 9 | 0 | 0 |
| 533.29089 | 533.290863 | 1676869 | 33 | 42 | 0 | 6 | 0 | 0 |
| 533.31206 | 533.311992 | 8666182 | 30 | 46 | 0 | 8 | 0 | 0 |
| 535.051855 | 535.051814 | 1437113 | 26 | 16 | 0 | 13 | 0 | 0 |
| 535.088067 | 535.0882 | 2147003 | 27 | 20 | 0 | 12 | 0 | 0 |
| 535.124554 | 535.124585 | 3075262 | 28 | 24 | 0 | 11 | 0 | 0 |
| 535.161017 | 535.160971 | 2958528 | 29 | 28 | 0 | 10 | 0 | 0 |
| 535.164478 | 535.164341 | 1561536 | 26 | 32 | 0 | 10 | 1 | 0 |
| 535.181991 | 535.1821 | 3032258 | 26 | 32 | 0 | 12 | 0 | 0 |
| 535.197354 | 535.197356 | 3350211 | 30 | 32 | 0 | 9 | 0 | 0 |
| 535.200699 | 535.200727 | 2003651 | 27 | 36 | 0 | 9 | 1 | 0 |
| 535.2185 | 535.218486 | 5983428 | 27 | 36 | 0 | 11 | 0 | 0 |
| 535.233722 | 535.233742 | 3543749 | 31 | 36 | 0 | 8 | 0 | 0 |
| 535.237199 | 535.237112 | 2713797 | 28 | 40 | 0 | 8 | 1 | 0 |
| 535.25482 | 535.254871 | 6716614 | 28 | 40 | 0 | 10 | 0 | 0 |
| 535.270215 | 535.270127 | 2400455 | 32 | 40 | 0 | 7 | 0 | 0 |
| 535.291276 | 535.291257 | 4795593 | 29 | 44 | 0 | 9 | 0 | 0 |
| 535.306636 | 535.306513 | 1372874 | 33 | 44 | 0 | 6 | 0 | 0 |
| 535.327622 | 535.327642 | 3191499 | 30 | 48 | 0 | 8 | 0 | 0 |
| 537.067418 | 537.067464 | 1475641 | 26 | 18 | 0 | 13 | 0 | 0 |
| 537.082754 | 537.08272 | 1347386 | 30 | 18 | 0 | 10 | 0 | 0 |
| 537.103812 | 537.10385 | 2099515 | 27 | 22 | 0 | 12 | 0 | 0 |
| 537.140206 | 537.140235 | 2046270 | 28 | 26 | 0 | 11 | 0 | 0 |
| 537.17663 | 537.176621 | 3883328 | 29 | 30 | 0 | 10 | 0 | 0 |
| 537.179961 | 537.179992 | 1853760 | 26 | 34 | 0 | 10 | 1 | 0 |
| 537.197675 | 537.19775 | 2679105 | 26 | 34 | 0 | 12 | 0 | 0 |
| 537.212911 | 537.213006 | 4418882 | 30 | 34 | 0 | 9 | 0 | 0 |
| 537.216321 | 537.216377 | 2063682 | 27 | 38 | 0 | 9 | 1 | 0 |
| 537.234164 | 537.234136 | 4008259 | 27 | 38 | 0 | 11 | 0 | 0 |
| 537.24932 | 537.249392 | 3631940 | 31 | 38 | 0 | 8 | 0 | 0 |
| 537.252601 | 537.252763 | 1428293 | 28 | 42 | 0 | 8 | 1 | 0 |
| 537.270479 | 537.270521 | 3099974 | 28 | 42 | 0 | 10 | 0 | 0 |
| 537.285785 | 537.285777 | 2721607 | 32 | 42 | 0 | 7 | 0 | 0 |
| 537.306768 | 537.306907 | 1704776 | 29 | 46 | 0 | 9 | 0 | 0 |
| 539.061851 | 539.061985 | 1401778 | 29 | 16 | 0 | 11 | 0 | 0 |
| 539.083027 | 539.083114 | 1282228 | 26 | 20 | 0 | 13 | 0 | 0 |
| 539.098456 | 539.09837 | 1605812 | 30 | 20 | 0 | 10 | 0 | 0 |
| 539.119334 | 539.1195 | 1523894 | 27 | 24 | 0 | 12 | 0 | 0 |
| 539.134867 | 539.134756 | 1427383 | 31 | 24 | 0 | 9 | 0 | 0 |
| 539.155853 | 539.155885 | 2877368 | 28 | 28 | 0 | 11 | 0 | 0 |
| 539.159338 | 539.159256 | 1502392 | 25 | 32 | 0 | 11 | 1 | 0 |
| 539.171155 | 539.171141 | 1731769 | 32 | 28 | 0 | 8 | 0 | 0 |
| 539.192293 | 539.192271 | 3917242 | 29 | 32 | 0 | 10 | 0 | 0 |
| 539.19565 | 539.195642 | 2497978 | 26 | 36 | 0 | 10 | 1 | 0 |
| 539.213337 | 539.2134 | 1631419 | 26 | 36 | 0 | 12 | 0 | 0 |
| 539.228642 | 539.228656 | 5520828 | 30 | 36 | 0 | 9 | 0 | 0 |
| 539.232124 | 539.232027 | 2189756 | 27 | 40 | 0 | 9 | 1 | 0 |
| 539.249891 | 539.249786 | 1838525 | 27 | 40 | 0 | 11 | 0 | 0 |
| 539.265043 | 539.265042 | 4024766 | 31 | 40 | 0 | 8 | 0 | 0 |
| 539.301411 | 539.301427 | 1980096 | 32 | 44 | 0 | 7 | 0 | 0 |
| 541.077721 | 541.077635 | 1775143 | 29 | 18 | 0 | 11 | 0 | 0 |
| 541.098716 | 541.098764 | 1368360 | 26 | 22 | 0 | 13 | 0 | 0 |
| 541.113964 | 541.11402 | 1990185 | 30 | 22 | 0 | 10 | 0 | 0 |
| 541.135196 | 541.13515 | 2058282 | 27 | 26 | 0 | 12 | 0 | 0 |
| 541.150258 | 541.150406 | 1413931 | 31 | 26 | 0 | 9 | 0 | 0 |
| 541.171448 | 541.171535 | 3431468 | 28 | 30 | 0 | 11 | 0 | 0 |
| 541.174747 | 541.174906 | 1835564 | 25 | 34 | 0 | 11 | 1 | 0 |
| 541.186801 | 541.186791 | 1672237 | 32 | 30 | 0 | 8 | 0 | 0 |
| 541.207904 | 541.207921 | 5196334 | 29 | 34 | 0 | 10 | 0 | 0 |
| 541.211193 | 541.211292 | 1986350 | 26 | 38 | 0 | 10 | 1 | 0 |
| 541.244344 | 541.244306 | 6456880 | 30 | 38 | 0 | 9 | 0 | 0 |
| 541.28069 | 541.280692 | 4228658 | 31 | 42 | 0 | 8 | 0 | 0 |
| 541.317102 | 541.317077 | 1631796 | 32 | 46 | 0 | 7 | 0 | 0 |
| 543.093245 | 543.093285 | 2276501 | 29 | 20 | 0 | 11 | 0 | 0 |
| 543.129729 | 543.129671 | 1568919 | 30 | 24 | 0 | 10 | 0 | 0 |
| 543.150699 | 543.1508 | 2591896 | 27 | 28 | 0 | 12 | 0 | 0 |
| 543.165964 | 543.166056 | 1812633 | 31 | 28 | 0 | 9 | 0 | 0 |
| 543.187123 | 543.187185 | 4510362 | 28 | 32 | 0 | 11 | 0 | 0 |
| 543.190336 | 543.190556 | 1364122 | 25 | 36 | 0 | 11 | 1 | 0 |
| 543.20246 | 543.202442 | 2039451 | 32 | 32 | 0 | 8 | 0 | 0 |
| 543.223555 | 543.223571 | 7896732 | 29 | 36 | 0 | 10 | 0 | 0 |
| 543.238937 | 543.238827 | 1512093 | 33 | 36 | 0 | 7 | 0 | 0 |
| 543.259974 | 543.259956 | 8580766 | 30 | 40 | 0 | 9 | 0 | 0 |
| 543.27531 | 543.275213 | 1245087 | 34 | 40 | 0 | 6 | 0 | 0 |
| 543.296299 | 543.296342 | 3287200 | 31 | 44 | 0 | 8 | 0 | 0 |
| 543.332903 | 543.332727 | 1541794 | 32 | 48 | 0 | 7 | 0 | 0 |
| 545.072449 | 545.07255 | 1731067 | 28 | 18 | 0 | 12 | 0 | 0 |
| 545.109034 | 545.108935 | 1601277 | 29 | 22 | 0 | 11 | 0 | 0 |
| 545.145301 | 545.145321 | 1918206 | 30 | 26 | 0 | 10 | 0 | 0 |
| 545.166469 | 545.16645 | 2498303 | 27 | 30 | 0 | 12 | 0 | 0 |
| 545.181615 | 545.181706 | 1872640 | 31 | 30 | 0 | 9 | 0 | 0 |
| 545.202816 | 545.202835 | 6103809 | 28 | 34 | 0 | 11 | 0 | 0 |
| 545.218009 | 545.218092 | 2514178 | 32 | 34 | 0 | 8 | 0 | 0 |
| 545.239202 | 545.239221 | 9160451 | 29 | 38 | 0 | 10 | 0 | 0 |
| 545.254463 | 545.254477 | 2197764 | 33 | 38 | 0 | 7 | 0 | 0 |
| 545.275619 | 545.275606 | 9357061 | 30 | 42 | 0 | 9 | 0 | 0 |
| 545.311932 | 545.311992 | 3302663 | 31 | 46 | 0 | 8 | 0 | 0 |
| 547.051722 | 547.051814 | 1346138 | 27 | 16 | 0 | 13 | 0 | 0 |
| 547.088237 | 547.0882 | 1804123 | 28 | 20 | 0 | 12 | 0 | 0 |
| 547.124518 | 547.124585 | 2184541 | 29 | 24 | 0 | 11 | 0 | 0 |
| 547.145761 | 547.145715 | 1438558 | 26 | 28 | 0 | 13 | 0 | 0 |
| 547.160854 | 547.160971 | 2479967 | 30 | 28 | 0 | 10 | 0 | 0 |
| 547.18215 | 547.1821 | 3690336 | 27 | 32 | 0 | 12 | 0 | 0 |
| 547.197311 | 547.197356 | 2204768 | 31 | 32 | 0 | 9 | 0 | 0 |
| 547.218403 | 547.218486 | 6973281 | 28 | 36 | 0 | 11 | 0 | 0 |
| 547.233699 | 547.233742 | 2312546 | 32 | 36 | 0 | 8 | 0 | 0 |
| 547.254848 | 547.254871 | 8761187 | 29 | 40 | 0 | 10 | 0 | 0 |
| 547.269998 | 547.270127 | 2327396 | 33 | 40 | 0 | 7 | 0 | 0 |
| 547.291214 | 547.291257 | 7337829 | 30 | 44 | 0 | 9 | 0 | 0 |
| 547.306489 | 547.306513 | 1588837 | 34 | 44 | 0 | 6 | 0 | 0 |
| 547.327704 | 547.327642 | 1930086 | 31 | 48 | 0 | 8 | 0 | 0 |
| 549.067282 | 549.067464 | 1407411 | 27 | 18 | 0 | 13 | 0 | 0 |
| 549.1038 | 549.10385 | 1855412 | 28 | 22 | 0 | 12 | 0 | 0 |
| 549.140169 | 549.140235 | 2202038 | 29 | 26 | 0 | 11 | 0 | 0 |
| 549.161447 | 549.161365 | 1752759 | 26 | 30 | 0 | 13 | 0 | 0 |
| 549.176598 | 549.176621 | 2397879 | 30 | 30 | 0 | 10 | 0 | 0 |
| 549.179904 | 549.179992 | 1632439 | 27 | 34 | 0 | 10 | 1 | 0 |
| 549.19773 | 549.19775 | 3829688 | 27 | 34 | 0 | 12 | 0 | 0 |
| 549.213001 | 549.213006 | 2949561 | 31 | 34 | 0 | 9 | 0 | 0 |
| 549.216294 | 549.216377 | 1828793 | 28 | 38 | 0 | 9 | 1 | 0 |
| 549.234133 | 549.234136 | 5975994 | 28 | 38 | 0 | 11 | 0 | 0 |
| 549.249329 | 549.249392 | 2830266 | 32 | 38 | 0 | 8 | 0 | 0 |
| 549.252839 | 549.252763 | 1513402 | 29 | 42 | 0 | 8 | 1 | 0 |
| 549.270469 | 549.270521 | 5450683 | 29 | 42 | 0 | 10 | 0 | 0 |
| 549.285747 | 549.285777 | 2123708 | 33 | 42 | 0 | 7 | 0 | 0 |
| 549.306902 | 549.306907 | 4615101 | 30 | 46 | 0 | 9 | 0 | 0 |
| 551.083064 | 551.083114 | 1505540 | 27 | 20 | 0 | 13 | 0 | 0 |
| 551.09827 | 551.09837 | 1506308 | 31 | 20 | 0 | 10 | 0 | 0 |
| 551.119459 | 551.1195 | 1759749 | 28 | 24 | 0 | 12 | 0 | 0 |
| 551.134551 | 551.134756 | 1264902 | 32 | 24 | 0 | 9 | 0 | 0 |
| 551.155917 | 551.155885 | 2888711 | 29 | 28 | 0 | 11 | 0 | 0 |
| 551.176901 | 551.177015 | 1586695 | 26 | 32 | 0 | 13 | 0 | 0 |
| 551.192221 | 551.192271 | 2914312 | 30 | 32 | 0 | 10 | 0 | 0 |
| 551.195706 | 551.195642 | 1882120 | 27 | 36 | 0 | 10 | 1 | 0 |
| 551.21346 | 551.2134 | 3181065 | 27 | 36 | 0 | 12 | 0 | 0 |
| 551.228585 | 551.228656 | 2935817 | 31 | 36 | 0 | 9 | 0 | 0 |
| 551.23213 | 551.232027 | 2781706 | 28 | 40 | 0 | 9 | 1 | 0 |
| 551.249784 | 551.249786 | 3974154 | 28 | 40 | 0 | 11 | 0 | 0 |
| 551.265092 | 551.265042 | 3152907 | 32 | 40 | 0 | 8 | 0 | 0 |
| 551.268523 | 551.268413 | 1664011 | 29 | 44 | 0 | 8 | 1 | 0 |
| 551.286149 | 551.286171 | 3511308 | 29 | 44 | 0 | 10 | 0 | 0 |
| 551.301407 | 551.301427 | 1921292 | 33 | 44 | 0 | 7 | 0 | 0 |
| 551.322511 | 551.322557 | 2041869 | 30 | 48 | 0 | 9 | 0 | 0 |
| 553.077818 | 553.077635 | 1305676 | 30 | 18 | 0 | 11 | 0 | 0 |
| 553.098746 | 553.098764 | 1776717 | 27 | 22 | 0 | 13 | 0 | 0 |
| 553.114153 | 553.11402 | 1246285 | 31 | 22 | 0 | 10 | 0 | 0 |
| 553.135196 | 553.13515 | 1978446 | 28 | 26 | 0 | 12 | 0 | 0 |
| 553.150548 | 553.150406 | 1424463 | 32 | 26 | 0 | 9 | 0 | 0 |
| 553.171393 | 553.171535 | 2796623 | 29 | 30 | 0 | 11 | 0 | 0 |
| 553.174807 | 553.174906 | 1989455 | 26 | 34 | 0 | 11 | 1 | 0 |
| 553.207827 | 553.207921 | 3571281 | 30 | 34 | 0 | 10 | 0 | 0 |
| 553.211195 | 553.211292 | 2343505 | 27 | 38 | 0 | 10 | 1 | 0 |
| 553.22322 | 553.223177 | 1254865 | 34 | 34 | 0 | 7 | 0 | 0 |
| 553.228952 | 553.22905 | 1619793 | 27 | 38 | 0 | 12 | 0 | 0 |
| 553.244228 | 553.244306 | 3407442 | 31 | 38 | 0 | 9 | 0 | 0 |
| 553.247633 | 553.247677 | 2696274 | 28 | 42 | 0 | 9 | 1 | 0 |
| 553.265418 | 553.265436 | 1565010 | 28 | 42 | 0 | 11 | 0 | 0 |
| 553.280638 | 553.280692 | 2869843 | 32 | 42 | 0 | 8 | 0 | 0 |
| 553.317171 | 553.317077 | 1373780 | 33 | 46 | 0 | 7 | 0 | 0 |
| 555.056769 | 555.0569 | 1649547 | 29 | 16 | 0 | 12 | 0 | 0 |
| 555.093264 | 555.093285 | 1678732 | 30 | 20 | 0 | 11 | 0 | 0 |
| 555.129967 | 555.129671 | 1350029 | 31 | 24 | 0 | 10 | 0 | 0 |
| 555.150681 | 555.1508 | 2175118 | 28 | 28 | 0 | 12 | 0 | 0 |
| 555.166155 | 555.166056 | 1348495 | 32 | 28 | 0 | 9 | 0 | 0 |
| 555.187275 | 555.187185 | 3288719 | 29 | 32 | 0 | 11 | 0 | 0 |
| 555.190587 | 555.190556 | 1631887 | 26 | 36 | 0 | 11 | 1 | 0 |
| 555.202398 | 555.202442 | 1396624 | 33 | 32 | 0 | 8 | 0 | 0 |
| 555.2236 | 555.223571 | 4746384 | 30 | 36 | 0 | 10 | 0 | 0 |
| 555.22693 | 555.226942 | 1870736 | 27 | 40 | 0 | 10 | 1 | 0 |
| 555.259903 | 555.259956 | 3483793 | 31 | 40 | 0 | 9 | 0 | 0 |
| 555.263377 | 555.263327 | 1580433 | 28 | 44 | 0 | 9 | 1 | 0 |
| 555.296388 | 555.296342 | 2835090 | 32 | 44 | 0 | 8 | 0 | 0 |
| 557.072581 | 557.07255 | 1286851 | 29 | 18 | 0 | 12 | 0 | 0 |
| 557.108961 | 557.108935 | 2016452 | 30 | 22 | 0 | 11 | 0 | 0 |
| 557.145379 | 557.145321 | 2201285 | 31 | 26 | 0 | 10 | 0 | 0 |
| 557.16644 | 557.16645 | 2604741 | 28 | 30 | 0 | 12 | 0 | 0 |
| 557.18164 | 557.181706 | 1393093 | 32 | 30 | 0 | 9 | 0 | 0 |
| 557.202805 | 557.202835 | 4179142 | 29 | 34 | 0 | 11 | 0 | 0 |
| 557.206449 | 557.206206 | 1469382 | 26 | 38 | 0 | 11 | 1 | 0 |
| 557.218225 | 557.218092 | 1411526 | 33 | 34 | 0 | 8 | 0 | 0 |
| 557.239228 | 557.239221 | 5998791 | 30 | 38 | 0 | 10 | 0 | 0 |
| 557.27556 | 557.275606 | 4477128 | 31 | 42 | 0 | 9 | 0 | 0 |
| 557.311751 | 557.311992 | 2081737 | 32 | 46 | 0 | 8 | 0 | 0 |
| 559.088359 | 559.0882 | 2080497 | 29 | 20 | 0 | 12 | 0 | 0 |
| 559.124449 | 559.124585 | 1720818 | 30 | 24 | 0 | 11 | 0 | 0 |
| 559.145621 | 559.145715 | 1372402 | 27 | 28 | 0 | 13 | 0 | 0 |
| 559.16094 | 559.160971 | 1899250 | 31 | 28 | 0 | 10 | 0 | 0 |
| 559.18209 | 559.1821 | 3287795 | 28 | 32 | 0 | 12 | 0 | 0 |
| 559.197482 | 559.197356 | 1664243 | 32 | 32 | 0 | 9 | 0 | 0 |
| 559.218496 | 559.218486 | 5264628 | 29 | 36 | 0 | 11 | 0 | 0 |
| 559.233635 | 559.233742 | 1991924 | 33 | 36 | 0 | 8 | 0 | 0 |
| 559.254861 | 559.254871 | 6689012 | 30 | 40 | 0 | 10 | 0 | 0 |
| 559.270309 | 559.270127 | 1410293 | 34 | 40 | 0 | 7 | 0 | 0 |
| 559.291209 | 559.291257 | 3279605 | 31 | 44 | 0 | 9 | 0 | 0 |
| 559.327546 | 559.327642 | 1958134 | 32 | 48 | 0 | 8 | 0 | 0 |
| 561.067602 | 561.067464 | 1392405 | 28 | 18 | 0 | 13 | 0 | 0 |
| 561.103788 | 561.10385 | 1801750 | 29 | 22 | 0 | 12 | 0 | 0 |
| 561.140145 | 561.140235 | 1733143 | 30 | 26 | 0 | 11 | 0 | 0 |
| 561.161263 | 561.161365 | 1278487 | 27 | 30 | 0 | 13 | 0 | 0 |
| 561.176648 | 561.176621 | 2003735 | 31 | 30 | 0 | 10 | 0 | 0 |
| 561.197712 | 561.19775 | 3993880 | 28 | 34 | 0 | 12 | 0 | 0 |
| 561.212866 | 561.213006 | 2337560 | 32 | 34 | 0 | 9 | 0 | 0 |
| 561.234148 | 561.234136 | 5926168 | 29 | 38 | 0 | 11 | 0 | 0 |
| 561.249313 | 561.249392 | 2635032 | 33 | 38 | 0 | 8 | 0 | 0 |
| 561.270541 | 561.270521 | 6732057 | 30 | 42 | 0 | 10 | 0 | 0 |
| 561.285855 | 561.285777 | 1561625 | 34 | 42 | 0 | 7 | 0 | 0 |
| 561.306878 | 561.306907 | 2615577 | 31 | 46 | 0 | 9 | 0 | 0 |
| 563.083108 | 563.083114 | 1626674 | 28 | 20 | 0 | 13 | 0 | 0 |
| 563.119385 | 563.1195 | 1276211 | 29 | 24 | 0 | 12 | 0 | 0 |
| 563.155974 | 563.155885 | 2149171 | 30 | 28 | 0 | 11 | 0 | 0 |
| 563.17691 | 563.177015 | 1983283 | 27 | 32 | 0 | 13 | 0 | 0 |
| 563.192283 | 563.192271 | 2432307 | 31 | 32 | 0 | 10 | 0 | 0 |
| 563.195827 | 563.195642 | 1423667 | 28 | 36 | 0 | 10 | 1 | 0 |
| 563.213427 | 563.2134 | 4023604 | 28 | 36 | 0 | 12 | 0 | 0 |
| 563.228636 | 563.228656 | 2521908 | 32 | 36 | 0 | 9 | 0 | 0 |
| 563.249852 | 563.249786 | 5662004 | 29 | 40 | 0 | 11 | 0 | 0 |
| 563.265112 | 563.265042 | 2391348 | 33 | 40 | 0 | 8 | 0 | 0 |
| 563.286243 | 563.286171 | 4931893 | 30 | 44 | 0 | 10 | 0 | 0 |
| 563.301348 | 563.301427 | 1363509 | 34 | 44 | 0 | 7 | 0 | 0 |
| 563.322495 | 563.322557 | 1743925 | 31 | 48 | 0 | 9 | 0 | 0 |
| 565.114028 | 565.11402 | 1324103 | 32 | 22 | 0 | 10 | 0 | 0 |
| 565.135101 | 565.13515 | 1912647 | 29 | 26 | 0 | 12 | 0 | 0 |
| 565.150471 | 565.150406 | 1370439 | 33 | 26 | 0 | 9 | 0 | 0 |
| 565.171567 | 565.171535 | 1935687 | 30 | 30 | 0 | 11 | 0 | 0 |
| 565.192725 | 565.192665 | 1953095 | 27 | 34 | 0 | 13 | 0 | 0 |
| 565.2078 | 565.207921 | 3305287 | 31 | 34 | 0 | 10 | 0 | 0 |
| 565.211299 | 565.211292 | 2312008 | 28 | 38 | 0 | 10 | 1 | 0 |
| 565.229109 | 565.22905 | 3441992 | 28 | 38 | 0 | 12 | 0 | 0 |
| 565.244173 | 565.244306 | 2957640 | 32 | 38 | 0 | 9 | 0 | 0 |
| 565.247774 | 565.247677 | 2266440 | 29 | 42 | 0 | 9 | 1 | 0 |
| 565.265402 | 565.265436 | 3231560 | 29 | 42 | 0 | 11 | 0 | 0 |
| 565.280661 | 565.280692 | 2320200 | 33 | 42 | 0 | 8 | 0 | 0 |
| 565.284029 | 565.284063 | 1581896 | 30 | 46 | 0 | 8 | 1 | 0 |
| 565.301754 | 565.301821 | 2926920 | 30 | 46 | 0 | 10 | 0 | 0 |
| 565.317002 | 565.317077 | 1489736 | 34 | 46 | 0 | 7 | 0 | 0 |
| 567.093017 | 567.093285 | 1668947 | 31 | 20 | 0 | 11 | 0 | 0 |
| 567.114464 | 567.114414 | 1791571 | 28 | 24 | 0 | 13 | 0 | 0 |
| 567.15076 | 567.1508 | 1759571 | 29 | 28 | 0 | 12 | 0 | 0 |
| 567.187082 | 567.187185 | 2042707 | 30 | 32 | 0 | 11 | 0 | 0 |
| 567.190638 | 567.190556 | 2028883 | 27 | 36 | 0 | 11 | 1 | 0 |
| 567.208318 | 567.208315 | 1583187 | 27 | 36 | 0 | 13 | 0 | 0 |
| 567.2236 | 567.223571 | 3463508 | 31 | 36 | 0 | 10 | 0 | 0 |
| 567.226963 | 567.226942 | 3035988 | 28 | 40 | 0 | 10 | 1 | 0 |
| 567.244714 | 567.2447 | 2008916 | 28 | 40 | 0 | 12 | 0 | 0 |
| 567.259946 | 567.259956 | 3447124 | 32 | 40 | 0 | 9 | 0 | 0 |
| 567.263334 | 567.263327 | 2574164 | 29 | 44 | 0 | 9 | 1 | 0 |
| 567.281065 | 567.281086 | 1670740 | 29 | 44 | 0 | 11 | 0 | 0 |
| 567.296336 | 567.296342 | 2197844 | 33 | 44 | 0 | 8 | 0 | 0 |
| 567.317421 | 567.317471 | 1298772 | 30 | 48 | 0 | 10 | 0 | 0 |
| 569.109165 | 569.108935 | 1436760 | 31 | 22 | 0 | 11 | 0 | 0 |
| 569.130168 | 569.130064 | 1290584 | 28 | 26 | 0 | 13 | 0 | 0 |
| 569.145345 | 569.145321 | 1582424 | 32 | 26 | 0 | 10 | 0 | 0 |
| 569.166347 | 569.16645 | 2499416 | 29 | 30 | 0 | 12 | 0 | 0 |
| 569.181756 | 569.181706 | 1401176 | 33 | 30 | 0 | 9 | 0 | 0 |
| 569.202836 | 569.202835 | 3084120 | 30 | 34 | 0 | 11 | 0 | 0 |
| 569.206143 | 569.206206 | 2305880 | 27 | 38 | 0 | 11 | 1 | 0 |
| 569.218271 | 569.218092 | 1453400 | 34 | 34 | 0 | 8 | 0 | 0 |
| 569.239145 | 569.239221 | 3673944 | 31 | 38 | 0 | 10 | 0 | 0 |
| 569.242569 | 569.242592 | 2374488 | 28 | 42 | 0 | 10 | 1 | 0 |
| 569.260519 | 569.26035 | 1264216 | 28 | 42 | 0 | 12 | 0 | 0 |
| 569.27551 | 569.275606 | 3055448 | 32 | 42 | 0 | 9 | 0 | 0 |
| 569.311896 | 569.311992 | 1982808 | 33 | 46 | 0 | 8 | 0 | 0 |
| 571.088139 | 571.0882 | 1413462 | 30 | 20 | 0 | 12 | 0 | 0 |
| 571.124513 | 571.124585 | 1533526 | 31 | 24 | 0 | 11 | 0 | 0 |
| 571.145661 | 571.145715 | 1560918 | 28 | 28 | 0 | 13 | 0 | 0 |
| 571.160958 | 571.160971 | 1445718 | 32 | 28 | 0 | 10 | 0 | 0 |
| 571.182071 | 571.1821 | 2473302 | 29 | 32 | 0 | 12 | 0 | 0 |
| 571.185449 | 571.185471 | 1546326 | 26 | 36 | 0 | 12 | 1 | 0 |
| 571.197408 | 571.197356 | 1656150 | 33 | 32 | 0 | 9 | 0 | 0 |
| 571.218481 | 571.218486 | 4134230 | 30 | 36 | 0 | 11 | 0 | 0 |
| 571.233639 | 571.233742 | 1373014 | 34 | 36 | 0 | 8 | 0 | 0 |
| 571.254773 | 571.254871 | 4110678 | 31 | 40 | 0 | 10 | 0 | 0 |
| 571.269993 | 571.270127 | 1436245 | 35 | 40 | 0 | 7 | 0 | 0 |
| 571.29129 | 571.291257 | 2973525 | 32 | 44 | 0 | 9 | 0 | 0 |
| 571.306268 | 571.306513 | 1240405 | 36 | 44 | 0 | 6 | 0 | 0 |
| 571.327602 | 571.327642 | 1556821 | 33 | 48 | 0 | 8 | 0 | 0 |
| 573.067456 | 573.067464 | 1473613 | 29 | 18 | 0 | 13 | 0 | 0 |
| 573.103903 | 573.10385 | 1350733 | 30 | 22 | 0 | 12 | 0 | 0 |
| 573.140204 | 573.140235 | 1761869 | 31 | 26 | 0 | 11 | 0 | 0 |
| 573.161197 | 573.161365 | 1355341 | 28 | 30 | 0 | 13 | 0 | 0 |
| 573.1765 | 573.176621 | 1613645 | 32 | 30 | 0 | 10 | 0 | 0 |
| 573.197674 | 573.19775 | 3383629 | 29 | 34 | 0 | 12 | 0 | 0 |
| 573.213016 | 573.213006 | 1866061 | 33 | 34 | 0 | 9 | 0 | 0 |
| 573.234028 | 573.234136 | 4623693 | 30 | 38 | 0 | 11 | 0 | 0 |
| 573.249502 | 573.249392 | 1323340 | 34 | 38 | 0 | 8 | 0 | 0 |
| 573.270387 | 573.270521 | 3842380 | 31 | 42 | 0 | 10 | 0 | 0 |
| 573.285652 | 573.285777 | 1653580 | 35 | 42 | 0 | 7 | 0 | 0 |
| 573.306707 | 573.306907 | 2706252 | 32 | 46 | 0 | 9 | 0 | 0 |
| 575.119527 | 575.1195 | 1622590 | 30 | 24 | 0 | 12 | 0 | 0 |
| 575.15586 | 575.155885 | 2005822 | 31 | 28 | 0 | 11 | 0 | 0 |
| 575.176857 | 575.177015 | 1983805 | 28 | 32 | 0 | 13 | 0 | 0 |
| 575.19213 | 575.192271 | 1690173 | 32 | 32 | 0 | 10 | 0 | 0 |
| 575.213439 | 575.2134 | 4167997 | 29 | 36 | 0 | 12 | 0 | 0 |
| 575.228646 | 575.228656 | 2021181 | 33 | 36 | 0 | 9 | 0 | 0 |
| 575.249742 | 575.249786 | 5465405 | 30 | 40 | 0 | 11 | 0 | 0 |
| 575.265141 | 575.265042 | 2341181 | 34 | 40 | 0 | 8 | 0 | 0 |
| 575.286135 | 575.286171 | 3265340 | 31 | 44 | 0 | 10 | 0 | 0 |
| 575.301416 | 575.301427 | 1357116 | 35 | 44 | 0 | 7 | 0 | 0 |
| 575.322467 | 575.322557 | 1474876 | 32 | 48 | 0 | 9 | 0 | 0 |
| 577.135085 | 577.13515 | 2162984 | 30 | 26 | 0 | 12 | 0 | 0 |
| 577.1714 | 577.171535 | 1847592 | 31 | 30 | 0 | 11 | 0 | 0 |
| 577.192734 | 577.192665 | 2036776 | 28 | 34 | 0 | 13 | 0 | 0 |
| 577.207948 | 577.207921 | 1898280 | 32 | 34 | 0 | 10 | 0 | 0 |
| 577.229046 | 577.22905 | 3985191 | 29 | 38 | 0 | 12 | 0 | 0 |
| 577.244238 | 577.244306 | 2528551 | 33 | 38 | 0 | 9 | 0 | 0 |
| 577.265366 | 577.265436 | 4305191 | 30 | 42 | 0 | 11 | 0 | 0 |
| 577.280621 | 577.280692 | 1918759 | 34 | 42 | 0 | 8 | 0 | 0 |
| 577.301882 | 577.301821 | 2042918 | 31 | 46 | 0 | 10 | 0 | 0 |
| 579.093374 | 579.093285 | 1281550 | 32 | 20 | 0 | 11 | 0 | 0 |
| 579.114452 | 579.114414 | 1432846 | 29 | 24 | 0 | 13 | 0 | 0 |
| 579.150575 | 579.1508 | 1405197 | 30 | 28 | 0 | 12 | 0 | 0 |
| 579.187137 | 579.187185 | 2551565 | 31 | 32 | 0 | 11 | 0 | 0 |
| 579.190449 | 579.190556 | 2065677 | 28 | 36 | 0 | 11 | 1 | 0 |
| 579.208377 | 579.208315 | 1828364 | 28 | 36 | 0 | 13 | 0 | 0 |
| 579.223603 | 579.223571 | 2303756 | 32 | 36 | 0 | 10 | 0 | 0 |
| 579.227026 | 579.226942 | 2286348 | 29 | 40 | 0 | 10 | 1 | 0 |
| 579.244584 | 579.2447 | 3017484 | 29 | 40 | 0 | 12 | 0 | 0 |
| 579.259977 | 579.259956 | 2289420 | 33 | 40 | 0 | 9 | 0 | 0 |
| 579.263521 | 579.263327 | 2131724 | 30 | 44 | 0 | 9 | 1 | 0 |
| 579.281134 | 579.281086 | 3251979 | 30 | 44 | 0 | 11 | 0 | 0 |
| 579.296229 | 579.296342 | 1660171 | 34 | 44 | 0 | 8 | 0 | 0 |
| 581.13014 | 581.130064 | 1757421 | 29 | 26 | 0 | 13 | 0 | 0 |
| 581.166435 | 581.16645 | 1776877 | 30 | 30 | 0 | 12 | 0 | 0 |
| 581.202647 | 581.202835 | 2007276 | 31 | 34 | 0 | 11 | 0 | 0 |
| 581.206232 | 581.206206 | 1803500 | 28 | 38 | 0 | 11 | 1 | 0 |
| 581.238965 | 581.239221 | 2500331 | 32 | 38 | 0 | 10 | 0 | 0 |
| 581.24275 | 581.242592 | 2411243 | 29 | 42 | 0 | 10 | 1 | 0 |
| 581.260164 | 581.26035 | 1663467 | 29 | 42 | 0 | 12 | 0 | 0 |
| 581.275358 | 581.275606 | 2459371 | 33 | 42 | 0 | 9 | 0 | 0 |
| 581.278949 | 581.278977 | 1574891 | 30 | 46 | 0 | 9 | 1 | 0 |
| 581.296715 | 581.296736 | 1673962 | 30 | 46 | 0 | 11 | 0 | 0 |
| 581.312145 | 581.311992 | 1636842 | 34 | 46 | 0 | 8 | 0 | 0 |
| 583.088357 | 583.0882 | 1525961 | 31 | 20 | 0 | 12 | 0 | 0 |
| 583.124426 | 583.124585 | 1357512 | 32 | 24 | 0 | 11 | 0 | 0 |
| 583.16091 | 583.160971 | 1452743 | 33 | 28 | 0 | 10 | 0 | 0 |
| 583.182059 | 583.1821 | 2177735 | 30 | 32 | 0 | 12 | 0 | 0 |
| 583.185348 | 583.185471 | 1611975 | 27 | 36 | 0 | 12 | 1 | 0 |
| 583.197181 | 583.197356 | 1417415 | 34 | 32 | 0 | 9 | 0 | 0 |
| 583.218467 | 583.218486 | 2589894 | 31 | 36 | 0 | 11 | 0 | 0 |
| 583.221887 | 583.221856 | 2673862 | 28 | 40 | 0 | 11 | 1 | 0 |
| 583.254888 | 583.254871 | 2778822 | 32 | 40 | 0 | 10 | 0 | 0 |
| 583.258312 | 583.258242 | 2276550 | 29 | 44 | 0 | 10 | 1 | 0 |
| 583.291118 | 583.291257 | 2216645 | 33 | 44 | 0 | 9 | 0 | 0 |
| 583.327507 | 583.327642 | 1335492 | 34 | 48 | 0 | 8 | 0 | 0 |
| 585.103842 | 585.10385 | 1440671 | 31 | 22 | 0 | 12 | 0 | 0 |
| 585.140646 | 585.140235 | 1400734 | 32 | 26 | 0 | 11 | 0 | 0 |
| 585.161498 | 585.161365 | 1622173 | 29 | 30 | 0 | 13 | 0 | 0 |
| 585.176746 | 585.176621 | 1488285 | 33 | 30 | 0 | 10 | 0 | 0 |
| 585.197711 | 585.19775 | 2670237 | 30 | 34 | 0 | 12 | 0 | 0 |
| 585.201061 | 585.201121 | 1284253 | 27 | 38 | 0 | 12 | 1 | 0 |
| 585.213334 | 585.213006 | 1459356 | 34 | 34 | 0 | 9 | 0 | 0 |
| 585.234122 | 585.234136 | 3132060 | 31 | 38 | 0 | 11 | 0 | 0 |
| 585.237649 | 585.237506 | 1803164 | 28 | 42 | 0 | 11 | 1 | 0 |
| 585.249396 | 585.249392 | 1794203 | 35 | 38 | 0 | 8 | 0 | 0 |
| 585.270501 | 585.270521 | 3078811 | 32 | 42 | 0 | 10 | 0 | 0 |
| 585.306958 | 585.306907 | 2179226 | 33 | 46 | 0 | 9 | 0 | 0 |
| 587.119491 | 587.1195 | 1517168 | 31 | 24 | 0 | 12 | 0 | 0 |
| 587.155968 | 587.155885 | 1559663 | 32 | 28 | 0 | 11 | 0 | 0 |
| 587.176878 | 587.177015 | 2027631 | 29 | 32 | 0 | 13 | 0 | 0 |
| 587.192381 | 587.192271 | 1300078 | 33 | 32 | 0 | 10 | 0 | 0 |
| 587.213329 | 587.2134 | 3039342 | 30 | 36 | 0 | 12 | 0 | 0 |
| 587.228979 | 587.228656 | 1342062 | 34 | 36 | 0 | 9 | 0 | 0 |
| 587.249896 | 587.249786 | 3030125 | 31 | 40 | 0 | 11 | 0 | 0 |
| 587.264938 | 587.265042 | 1481581 | 35 | 40 | 0 | 8 | 0 | 0 |
| 587.286163 | 587.286171 | 2751084 | 32 | 44 | 0 | 10 | 0 | 0 |
| 587.322606 | 587.322557 | 2083947 | 33 | 48 | 0 | 9 | 0 | 0 |
| 589.098798 | 589.098764 | 1552447 | 30 | 22 | 0 | 13 | 0 | 0 |
| 589.135172 | 589.13515 | 1329214 | 31 | 26 | 0 | 12 | 0 | 0 |
| 589.156274 | 589.156279 | 1402941 | 28 | 30 | 0 | 14 | 0 | 0 |
| 589.171536 | 589.171535 | 1693245 | 32 | 30 | 0 | 11 | 0 | 0 |
| 589.192574 | 589.192665 | 2191420 | 29 | 34 | 0 | 13 | 0 | 0 |
| 589.20795 | 589.207921 | 1463100 | 33 | 34 | 0 | 10 | 0 | 0 |
| 589.229086 | 589.22905 | 3354171 | 30 | 38 | 0 | 12 | 0 | 0 |
| 589.244229 | 589.244306 | 1915963 | 34 | 38 | 0 | 9 | 0 | 0 |
| 589.265357 | 589.265436 | 3169338 | 31 | 42 | 0 | 11 | 0 | 0 |
| 589.280787 | 589.280692 | 1563706 | 35 | 42 | 0 | 8 | 0 | 0 |
| 589.301767 | 589.301821 | 2113594 | 32 | 46 | 0 | 10 | 0 | 0 |
| 591.15093 | 591.1508 | 1584648 | 31 | 28 | 0 | 12 | 0 | 0 |
| 591.18718 | 591.187185 | 1926151 | 32 | 32 | 0 | 11 | 0 | 0 |
| 591.208154 | 591.208315 | 1922310 | 29 | 36 | 0 | 13 | 0 | 0 |
| 591.223456 | 591.223571 | 2294790 | 33 | 36 | 0 | 10 | 0 | 0 |
| 591.244583 | 591.2447 | 3303429 | 30 | 40 | 0 | 12 | 0 | 0 |
| 591.259841 | 591.259956 | 1985541 | 34 | 40 | 0 | 9 | 0 | 0 |
| 591.281047 | 591.281086 | 2397700 | 31 | 44 | 0 | 11 | 0 | 0 |
| 591.296209 | 591.296342 | 1400580 | 35 | 44 | 0 | 8 | 0 | 0 |
| 591.317674 | 591.317471 | 1478915 | 32 | 48 | 0 | 10 | 0 | 0 |
| 593.130044 | 593.130064 | 1385167 | 30 | 26 | 0 | 13 | 0 | 0 |
| 593.145237 | 593.145321 | 1253327 | 34 | 26 | 0 | 10 | 0 | 0 |
| 593.166317 | 593.16645 | 1360590 | 31 | 30 | 0 | 12 | 0 | 0 |
| 593.202677 | 593.202835 | 1834189 | 32 | 34 | 0 | 11 | 0 | 0 |
| 593.206168 | 593.206206 | 1684941 | 29 | 38 | 0 | 11 | 1 | 0 |
| 593.223841 | 593.223965 | 1902029 | 29 | 38 | 0 | 13 | 0 | 0 |
| 593.239153 | 593.239221 | 2490828 | 33 | 38 | 0 | 10 | 0 | 0 |
| 593.242529 | 593.242592 | 1816524 | 30 | 42 | 0 | 10 | 1 | 0 |
| 593.260467 | 593.26035 | 2305996 | 30 | 42 | 0 | 12 | 0 | 0 |
| 593.275579 | 593.275606 | 2150347 | 34 | 42 | 0 | 9 | 0 | 0 |
| 593.29686 | 593.296736 | 1953739 | 31 | 46 | 0 | 11 | 0 | 0 |
| 593.311872 | 593.311992 | 1575370 | 35 | 46 | 0 | 8 | 0 | 0 |
| 595.109288 | 595.109329 | 1366164 | 29 | 24 | 0 | 14 | 0 | 0 |
| 595.145985 | 595.145715 | 1330067 | 30 | 28 | 0 | 13 | 0 | 0 |
| 595.181901 | 595.1821 | 1845138 | 31 | 32 | 0 | 12 | 0 | 0 |
| 595.218283 | 595.218486 | 2243473 | 32 | 36 | 0 | 11 | 0 | 0 |
| 595.22196 | 595.221856 | 2305169 | 29 | 40 | 0 | 11 | 1 | 0 |
| 595.254676 | 595.254871 | 2312592 | 33 | 40 | 0 | 10 | 0 | 0 |
| 595.258246 | 595.258242 | 2099344 | 30 | 44 | 0 | 10 | 1 | 0 |
| 595.27598 | 595.276 | 1790863 | 30 | 44 | 0 | 12 | 0 | 0 |
| 595.291225 | 595.291257 | 2067855 | 34 | 44 | 0 | 9 | 0 | 0 |
| 595.327575 | 595.327642 | 1495438 | 35 | 48 | 0 | 8 | 0 | 0 |
| 597.161276 | 597.161365 | 1559380 | 30 | 30 | 0 | 13 | 0 | 0 |
| 597.197686 | 597.19775 | 1919315 | 31 | 34 | 0 | 12 | 0 | 0 |
| 597.201054 | 597.201121 | 2084947 | 28 | 38 | 0 | 12 | 1 | 0 |
| 597.234125 | 597.234136 | 2813266 | 32 | 38 | 0 | 11 | 0 | 0 |
| 597.237516 | 597.237506 | 2405202 | 29 | 42 | 0 | 11 | 1 | 0 |
| 597.270401 | 597.270521 | 2169169 | 33 | 42 | 0 | 10 | 0 | 0 |
| 597.273692 | 597.273892 | 1566801 | 30 | 46 | 0 | 10 | 1 | 0 |
| 597.291486 | 597.29165 | 1252432 | 30 | 46 | 0 | 12 | 0 | 0 |
| 597.30672 | 597.306907 | 2159440 | 34 | 46 | 0 | 9 | 0 | 0 |
| 599.155571 | 599.155885 | 1381652 | 33 | 28 | 0 | 11 | 0 | 0 |
| 599.213448 | 599.2134 | 2226962 | 31 | 36 | 0 | 12 | 0 | 0 |
| 599.216904 | 599.216771 | 1571858 | 28 | 40 | 0 | 12 | 1 | 0 |
| 599.22855 | 599.228656 | 1333010 | 35 | 36 | 0 | 9 | 0 | 0 |
| 599.249793 | 599.249786 | 2922257 | 32 | 40 | 0 | 11 | 0 | 0 |
| 599.253253 | 599.253156 | 1523473 | 29 | 44 | 0 | 11 | 1 | 0 |
| 599.265105 | 599.265042 | 1260049 | 36 | 40 | 0 | 8 | 0 | 0 |
| 599.286165 | 599.286171 | 1957648 | 33 | 44 | 0 | 10 | 0 | 0 |
| 599.322505 | 599.322557 | 1459471 | 34 | 48 | 0 | 9 | 0 | 0 |
| 601.098976 | 601.098764 | 1523924 | 31 | 22 | 0 | 13 | 0 | 0 |
| 601.135236 | 601.13515 | 1685971 | 32 | 26 | 0 | 12 | 0 | 0 |
| 601.192619 | 601.192665 | 1365713 | 30 | 34 | 0 | 13 | 0 | 0 |
| 601.228923 | 601.22905 | 2382032 | 31 | 38 | 0 | 12 | 0 | 0 |
| 601.232435 | 601.232421 | 1331407 | 28 | 42 | 0 | 12 | 1 | 0 |
| 601.244364 | 601.244306 | 1389007 | 35 | 38 | 0 | 9 | 0 | 0 |
| 601.265379 | 601.265436 | 2593486 | 32 | 42 | 0 | 11 | 0 | 0 |
| 601.280721 | 601.280692 | 1283534 | 36 | 42 | 0 | 8 | 0 | 0 |
| 601.301746 | 601.301821 | 1888973 | 33 | 46 | 0 | 10 | 0 | 0 |
| 601.338126 | 601.338207 | 1405644 | 34 | 50 | 0 | 9 | 0 | 0 |
| 603.114581 | 603.114414 | 1501328 | 31 | 24 | 0 | 13 | 0 | 0 |
| 603.150688 | 603.1508 | 1405070 | 32 | 28 | 0 | 12 | 0 | 0 |
| 603.18683 | 603.187185 | 1451917 | 33 | 32 | 0 | 11 | 0 | 0 |
| 603.208145 | 603.208315 | 1982604 | 30 | 36 | 0 | 13 | 0 | 0 |
| 603.223543 | 603.223571 | 1665164 | 34 | 36 | 0 | 10 | 0 | 0 |
| 603.244457 | 603.2447 | 2387083 | 31 | 40 | 0 | 12 | 0 | 0 |
| 603.259769 | 603.259956 | 1376651 | 35 | 40 | 0 | 9 | 0 | 0 |
| 603.281074 | 603.281086 | 2639498 | 32 | 44 | 0 | 11 | 0 | 0 |
| 605.166404 | 605.16645 | 1426761 | 32 | 30 | 0 | 12 | 0 | 0 |
| 605.202864 | 605.202835 | 1815112 | 33 | 34 | 0 | 11 | 0 | 0 |
| 605.223704 | 605.223965 | 2075207 | 30 | 38 | 0 | 13 | 0 | 0 |
| 605.239108 | 605.239221 | 1875015 | 34 | 38 | 0 | 10 | 0 | 0 |
| 605.260389 | 605.26035 | 2522694 | 31 | 42 | 0 | 12 | 0 | 0 |
| 605.275549 | 605.275606 | 2128453 | 35 | 42 | 0 | 9 | 0 | 0 |
| 605.296604 | 605.296736 | 1650757 | 32 | 46 | 0 | 11 | 0 | 0 |
| 605.311847 | 605.311992 | 1575492 | 36 | 46 | 0 | 8 | 0 | 0 |
| 605.332724 | 605.333121 | 1361987 | 33 | 50 | 0 | 10 | 0 | 0 |
| 607.109278 | 607.109329 | 1319173 | 30 | 24 | 0 | 14 | 0 | 0 |
| 607.18212 | 607.1821 | 1488387 | 32 | 32 | 0 | 12 | 0 | 0 |
| 607.185783 | 607.185471 | 1511683 | 29 | 36 | 0 | 12 | 1 | 0 |
| 607.20338 | 607.203229 | 1383938 | 29 | 36 | 0 | 14 | 0 | 0 |
| 607.218644 | 607.218486 | 1728514 | 33 | 36 | 0 | 11 | 0 | 0 |
| 607.221759 | 607.221856 | 1712642 | 30 | 40 | 0 | 11 | 1 | 0 |
| 607.239547 | 607.239615 | 2176513 | 30 | 40 | 0 | 13 | 0 | 0 |
| 607.254635 | 607.254871 | 1634816 | 34 | 40 | 0 | 10 | 0 | 0 |
| 607.276085 | 607.276 | 1836800 | 31 | 44 | 0 | 12 | 0 | 0 |
| 607.29114 | 607.291257 | 1630975 | 35 | 44 | 0 | 9 | 0 | 0 |
| 607.327329 | 607.327642 | 1270782 | 36 | 48 | 0 | 8 | 0 | 0 |
| 609.12515 | 609.124979 | 1355455 | 30 | 26 | 0 | 14 | 0 | 0 |
| 609.161835 | 609.161365 | 1215166 | 31 | 30 | 0 | 13 | 0 | 0 |
| 609.197602 | 609.19775 | 2149308 | 32 | 34 | 0 | 12 | 0 | 0 |
| 609.201131 | 609.201121 | 1609660 | 29 | 38 | 0 | 12 | 1 | 0 |
| 609.234136 | 609.234136 | 1527739 | 33 | 38 | 0 | 11 | 0 | 0 |
| 609.237618 | 609.237506 | 1534139 | 30 | 42 | 0 | 11 | 1 | 0 |
| 609.27063 | 609.270521 | 1889722 | 34 | 42 | 0 | 10 | 0 | 0 |
| 609.29143 | 609.29165 | 1247929 | 31 | 46 | 0 | 12 | 0 | 0 |
| 609.306838 | 609.306907 | 1859001 | 35 | 46 | 0 | 9 | 0 | 0 |
| 609.343006 | 609.343292 | 1518519 | 36 | 50 | 0 | 8 | 0 | 0 |
| 611.140533 | 611.140629 | 1506680 | 30 | 28 | 0 | 14 | 0 | 0 |
| 611.176904 | 611.177015 | 1598839 | 31 | 32 | 0 | 13 | 0 | 0 |
| 611.180552 | 611.180524 | 1268599 | 49 | 24 | 0 | 0 | 0 | 0 |
| 611.192382 | 611.192271 | 1335671 | 35 | 32 | 0 | 10 | 0 | 0 |
| 611.213357 | 611.2134 | 1700726 | 32 | 36 | 0 | 12 | 0 | 0 |
| 611.216797 | 611.216771 | 1878390 | 29 | 40 | 0 | 12 | 1 | 0 |
| 611.249736 | 611.249786 | 2187125 | 33 | 40 | 0 | 11 | 0 | 0 |
| 611.253194 | 611.253156 | 1998708 | 30 | 44 | 0 | 11 | 1 | 0 |
| 611.285983 | 611.286171 | 2252659 | 34 | 44 | 0 | 10 | 0 | 0 |
| 611.322411 | 611.322557 | 1395314 | 35 | 48 | 0 | 9 | 0 | 0 |
| 613.135242 | 613.13515 | 1464883 | 33 | 26 | 0 | 12 | 0 | 0 |
| 613.192626 | 613.192665 | 1694001 | 31 | 34 | 0 | 13 | 0 | 0 |
| 613.229072 | 613.22905 | 2135856 | 32 | 38 | 0 | 12 | 0 | 0 |
| 613.265397 | 613.265436 | 2465582 | 33 | 42 | 0 | 11 | 0 | 0 |
| 613.280728 | 613.280692 | 1249838 | 37 | 42 | 0 | 8 | 0 | 0 |
| 613.301652 | 613.301821 | 1847597 | 34 | 46 | 0 | 10 | 0 | 0 |
| 615.150856 | 615.1508 | 1497581 | 33 | 28 | 0 | 12 | 0 | 0 |
| 615.172049 | 615.171929 | 1284332 | 30 | 32 | 0 | 14 | 0 | 0 |
| 615.208264 | 615.208315 | 2144491 | 31 | 36 | 0 | 13 | 0 | 0 |
| 615.244574 | 615.2447 | 2470122 | 32 | 40 | 0 | 12 | 0 | 0 |
| 615.281223 | 615.281086 | 2305257 | 33 | 44 | 0 | 11 | 0 | 0 |
| 615.317431 | 615.317471 | 1868519 | 34 | 48 | 0 | 10 | 0 | 0 |
| 617.20287 | 617.202835 | 1218471 | 34 | 34 | 0 | 11 | 0 | 0 |
| 617.223865 | 617.223965 | 1613479 | 31 | 38 | 0 | 13 | 0 | 0 |
| 617.239076 | 617.239221 | 1633446 | 35 | 38 | 0 | 10 | 0 | 0 |
| 617.260214 | 617.26035 | 2420389 | 32 | 42 | 0 | 12 | 0 | 0 |
| 617.275589 | 617.275606 | 1363109 | 36 | 42 | 0 | 9 | 0 | 0 |
| 617.296645 | 617.296736 | 1884324 | 33 | 46 | 0 | 11 | 0 | 0 |
| 617.333153 | 617.333121 | 1476771 | 34 | 50 | 0 | 10 | 0 | 0 |
| 619.109414 | 619.109329 | 1320552 | 31 | 24 | 0 | 14 | 0 | 0 |
| 619.145819 | 619.145715 | 1301862 | 32 | 28 | 0 | 13 | 0 | 0 |
| 619.202882 | 619.203229 | 1284965 | 30 | 36 | 0 | 14 | 0 | 0 |
| 619.218336 | 619.218486 | 1841252 | 34 | 36 | 0 | 11 | 0 | 0 |
| 619.239545 | 619.239615 | 1641059 | 31 | 40 | 0 | 13 | 0 | 0 |
| 619.275971 | 619.276 | 2178146 | 32 | 44 | 0 | 12 | 0 | 0 |
| 619.291197 | 619.291257 | 1384546 | 36 | 44 | 0 | 9 | 0 | 0 |
| 621.161405 | 621.161365 | 1209637 | 32 | 30 | 0 | 13 | 0 | 0 |
| 621.176563 | 621.176621 | 1238820 | 36 | 30 | 0 | 10 | 0 | 0 |
| 621.197745 | 621.19775 | 1418788 | 33 | 34 | 0 | 12 | 0 | 0 |
| 621.234148 | 621.234136 | 1742370 | 34 | 38 | 0 | 11 | 0 | 0 |
| 621.255204 | 621.255265 | 1391138 | 31 | 42 | 0 | 13 | 0 | 0 |
| 621.270542 | 621.270521 | 1501985 | 35 | 42 | 0 | 10 | 0 | 0 |
| 621.291439 | 621.29165 | 1394721 | 32 | 46 | 0 | 12 | 0 | 0 |
| 621.306768 | 621.306907 | 1405984 | 36 | 46 | 0 | 9 | 0 | 0 |
| 623.155718 | 623.155885 | 1211622 | 35 | 28 | 0 | 11 | 0 | 0 |
| 623.176968 | 623.177015 | 1683941 | 32 | 32 | 0 | 13 | 0 | 0 |
| 623.213317 | 623.2134 | 1419492 | 33 | 36 | 0 | 12 | 0 | 0 |
| 623.216514 | 623.216771 | 1226468 | 30 | 40 | 0 | 12 | 1 | 0 |
| 623.24961 | 623.249786 | 2594787 | 34 | 40 | 0 | 11 | 0 | 0 |
| 623.286208 | 623.286171 | 1862882 | 35 | 44 | 0 | 10 | 0 | 0 |
| 623.322491 | 623.322557 | 1465825 | 36 | 48 | 0 | 9 | 0 | 0 |
| 625.192475 | 625.192665 | 1279912 | 32 | 34 | 0 | 13 | 0 | 0 |
| 625.229029 | 625.22905 | 1336487 | 33 | 38 | 0 | 12 | 0 | 0 |
| 625.232574 | 625.232421 | 1885095 | 30 | 42 | 0 | 12 | 1 | 0 |
| 625.26535 | 625.265436 | 1791398 | 34 | 42 | 0 | 11 | 0 | 0 |
| 625.280753 | 625.280692 | 1255078 | 38 | 42 | 0 | 8 | 0 | 0 |
| 625.3016 | 625.301821 | 1684389 | 35 | 46 | 0 | 10 | 0 | 0 |
| 627.208214 | 627.208315 | 1378926 | 32 | 36 | 0 | 13 | 0 | 0 |
| 627.244512 | 627.2447 | 2291053 | 33 | 40 | 0 | 12 | 0 | 0 |
| 627.281057 | 627.281086 | 1992044 | 34 | 44 | 0 | 11 | 0 | 0 |
| 627.317632 | 627.317471 | 1399915 | 35 | 48 | 0 | 10 | 0 | 0 |
| 629.129983 | 629.130064 | 1426744 | 33 | 26 | 0 | 13 | 0 | 0 |
| 629.202748 | 629.202835 | 1266230 | 35 | 34 | 0 | 11 | 0 | 0 |
| 629.223748 | 629.223965 | 1707318 | 32 | 38 | 0 | 13 | 0 | 0 |
| 629.239137 | 629.239221 | 1324597 | 36 | 38 | 0 | 10 | 0 | 0 |
| 629.260085 | 629.26035 | 2216757 | 33 | 42 | 0 | 12 | 0 | 0 |
| 629.275112 | 629.275606 | 1256244 | 37 | 42 | 0 | 9 | 0 | 0 |
| 629.296656 | 629.296736 | 1591092 | 34 | 46 | 0 | 11 | 0 | 0 |
| 629.333208 | 629.333121 | 1343283 | 35 | 50 | 0 | 10 | 0 | 0 |
| 631.18231 | 631.1821 | 1351937 | 34 | 32 | 0 | 12 | 0 | 0 |
| 631.239911 | 631.239615 | 1554176 | 32 | 40 | 0 | 13 | 0 | 0 |
| 631.276095 | 631.276 | 1874175 | 33 | 44 | 0 | 12 | 0 | 0 |
| 631.312257 | 631.312386 | 1699070 | 34 | 48 | 0 | 11 | 0 | 0 |
| 633.233917 | 633.234136 | 1528524 | 35 | 38 | 0 | 11 | 0 | 0 |
| 633.255188 | 633.255265 | 1359564 | 32 | 42 | 0 | 13 | 0 | 0 |
| 633.270502 | 633.270521 | 1241291 | 36 | 42 | 0 | 10 | 0 | 0 |
| 633.291537 | 633.29165 | 1832139 | 33 | 46 | 0 | 12 | 0 | 0 |
| 633.307016 | 633.306907 | 1352906 | 37 | 46 | 0 | 9 | 0 | 0 |
| 635.176878 | 635.177015 | 1490588 | 33 | 32 | 0 | 13 | 0 | 0 |
| 635.249775 | 635.249786 | 1773210 | 35 | 40 | 0 | 11 | 0 | 0 |
| 635.271012 | 635.270915 | 1470105 | 32 | 44 | 0 | 13 | 0 | 0 |
| 635.286443 | 635.286171 | 1435033 | 36 | 44 | 0 | 10 | 0 | 0 |
| 637.192772 | 637.192665 | 1307243 | 33 | 34 | 0 | 13 | 0 | 0 |
| 637.229155 | 637.22905 | 1466986 | 34 | 38 | 0 | 12 | 0 | 0 |
| 637.250491 | 637.25018 | 1512554 | 31 | 42 | 0 | 14 | 0 | 0 |
| 637.265302 | 637.265436 | 1667177 | 35 | 42 | 0 | 11 | 0 | 0 |
| 637.286814 | 637.286565 | 1332329 | 32 | 46 | 0 | 13 | 0 | 0 |
| 637.302072 | 637.301821 | 1515368 | 36 | 46 | 0 | 10 | 0 | 0 |
| 639.171704 | 639.171929 | 1195068 | 32 | 32 | 0 | 14 | 0 | 0 |
| 639.208514 | 639.208315 | 1359932 | 33 | 36 | 0 | 13 | 0 | 0 |
| 639.24464 | 639.2447 | 1524795 | 34 | 40 | 0 | 12 | 0 | 0 |
| 639.281103 | 639.281086 | 1628730 | 35 | 44 | 0 | 11 | 0 | 0 |
| 639.317322 | 639.317471 | 1595449 | 36 | 48 | 0 | 10 | 0 | 0 |
| 641.18761 | 641.187579 | 1336590 | 32 | 34 | 0 | 14 | 0 | 0 |
| 641.223925 | 641.223965 | 1558029 | 33 | 38 | 0 | 13 | 0 | 0 |
| 641.260136 | 641.26035 | 1841933 | 34 | 42 | 0 | 12 | 0 | 0 |
| 641.296937 | 641.296736 | 1966604 | 35 | 46 | 0 | 11 | 0 | 0 |
| 641.333231 | 641.333121 | 1238539 | 36 | 50 | 0 | 10 | 0 | 0 |
| 643.239679 | 643.239615 | 1614816 | 33 | 40 | 0 | 13 | 0 | 0 |
| 643.254767 | 643.254871 | 1205216 | 37 | 40 | 0 | 10 | 0 | 0 |
| 643.27595 | 643.276 | 2121695 | 34 | 44 | 0 | 12 | 0 | 0 |
| 643.312245 | 643.312386 | 1619167 | 35 | 48 | 0 | 11 | 0 | 0 |
| 645.218922 | 645.218879 | 1306548 | 32 | 38 | 0 | 14 | 0 | 0 |
| 645.234252 | 645.234136 | 1268660 | 36 | 38 | 0 | 11 | 0 | 0 |
| 645.255032 | 645.255265 | 1726644 | 33 | 42 | 0 | 13 | 0 | 0 |
| 645.270652 | 645.270521 | 1229235 | 37 | 42 | 0 | 10 | 0 | 0 |
| 645.291757 | 645.29165 | 1788339 | 34 | 46 | 0 | 12 | 0 | 0 |
| 647.1769 | 647.177015 | 1246346 | 34 | 32 | 0 | 13 | 0 | 0 |
| 647.234411 | 647.23453 | 1503625 | 32 | 40 | 0 | 14 | 0 | 0 |
| 647.271296 | 647.270915 | 1542536 | 33 | 44 | 0 | 13 | 0 | 0 |
| 647.285915 | 647.286171 | 1229959 | 37 | 44 | 0 | 10 | 0 | 0 |
| 649.229273 | 649.22905 | 1255261 | 35 | 38 | 0 | 12 | 0 | 0 |
| 649.265609 | 649.265436 | 1435485 | 36 | 42 | 0 | 11 | 0 | 0 |
| 651.134911 | 651.135544 | 1244212 | 32 | 28 | 0 | 15 | 0 | 0 |
| 651.244663 | 651.2447 | 1503282 | 35 | 40 | 0 | 12 | 0 | 0 |
| 651.281109 | 651.281086 | 1530673 | 36 | 44 | 0 | 11 | 0 | 0 |
| 653.260402 | 653.26035 | 1884422 | 35 | 42 | 0 | 12 | 0 | 0 |
| 653.296687 | 653.296736 | 1394438 | 36 | 46 | 0 | 11 | 0 | 0 |
| 655.239449 | 655.239615 | 1595100 | 34 | 40 | 0 | 13 | 0 | 0 |
| 655.275776 | 655.276 | 1699547 | 35 | 44 | 0 | 12 | 0 | 0 |
| 655.312089 | 655.312386 | 1359578 | 36 | 48 | 0 | 11 | 0 | 0 |
| 655.348754 | 655.348771 | 1199321 | 37 | 52 | 0 | 10 | 0 | 0 |
| 657.255207 | 657.255265 | 1237423 | 34 | 42 | 0 | 13 | 0 | 0 |
| 657.291417 | 657.29165 | 1258415 | 35 | 46 | 0 | 12 | 0 | 0 |
| 659.271013 | 659.270915 | 1360771 | 34 | 44 | 0 | 13 | 0 | 0 |
| 659.307555 | 659.307301 | 1261954 | 35 | 48 | 0 | 12 | 0 | 0 |
| 661.250308 | 661.25018 | 1413206 | 33 | 42 | 0 | 14 | 0 | 0 |
| 661.301729 | 661.301821 | 1196884 | 38 | 46 | 0 | 10 | 0 | 0 |
| 663.244807 | 663.2447 | 1178663 | 36 | 40 | 0 | 12 | 0 | 0 |
| 663.317395 | 663.317471 | 1229350 | 38 | 48 | 0 | 10 | 0 | 0 |
| 665.259928 | 665.26035 | 1241847 | 36 | 42 | 0 | 12 | 0 | 0 |
| 665.296253 | 665.296736 | 1340918 | 37 | 46 | 0 | 11 | 0 | 0 |
| 667.276008 | 667.276 | 1354438 | 36 | 44 | 0 | 12 | 0 | 0 |
| 667.311884 | 667.312386 | 1266117 | 37 | 48 | 0 | 11 | 0 | 0 |
| 669.291545 | 669.29165 | 1562003 | 36 | 46 | 0 | 12 | 0 | 0 |
| 669.328017 | 669.328036 | 1360786 | 37 | 50 | 0 | 11 | 0 | 0 |
| 671.176617 | 671.177015 | 1269857 | 36 | 32 | 0 | 13 | 0 | 0 |
| 671.270561 | 671.270915 | 1425503 | 35 | 44 | 0 | 13 | 0 | 0 |
| 673.229068 | 673.22905 | 1205801 | 37 | 38 | 0 | 12 | 0 | 0 |
| 675.265524 | 675.26583 | 1433071 | 34 | 44 | 0 | 14 | 0 | 0 |
| 677.260175 | 677.26035 | 1326516 | 37 | 42 | 0 | 12 | 0 | 0 |
| 679.16598 | 679.166192 | 1485945 | 26 | 36 | 2 | 17 | 1 | 0 |
| 679.202985 | 679.203229 | 1357944 | 35 | 36 | 0 | 14 | 0 | 0 |
| 679.239767 | 679.239615 | 1671287 | 36 | 40 | 0 | 13 | 0 | 0 |
| 679.276293 | 679.276 | 1428598 | 37 | 44 | 0 | 12 | 0 | 0 |
| 679.312008 | 679.312386 | 1168756 | 38 | 48 | 0 | 11 | 0 | 0 |
| 679.45004 | 679.449882 | 1189488 | 32 | 64 | 4 | 11 | 0 | 0 |
| 681.255143 | 681.255265 | 1380149 | 36 | 42 | 0 | 13 | 0 | 0 |
| 681.291553 | 681.29165 | 1233716 | 37 | 46 | 0 | 12 | 0 | 0 |
| 685.250372 | 685.25018 | 1360811 | 35 | 42 | 0 | 14 | 0 | 0 |
| 685.286628 | 685.286565 | 1223593 | 36 | 46 | 0 | 13 | 0 | 0 |
| 689.224176 | 689.223965 | 1182739 | 37 | 38 | 0 | 13 | 0 | 0 |

3. Sample: P1_T_0_

| Experimental mass | Exact mass | Peak height | C | H | N | O | S | P |
| --- | --- | --- | --- | --- | --- | --- | --- | --- |
| 209.081967 | 209.081932 | 1403717 | 11 | 14 | 0 | 4 | 0 | 0 |
| 215.144116 | 215.144139 | 1115995 | 15 | 20 | 0 | 1 | 0 | 0 |
| 217.087014 | 217.087018 | 1200884 | 13 | 14 | 0 | 3 | 0 | 0 |
| 217.123458 | 217.123403 | 1814549 | 14 | 18 | 0 | 2 | 0 | 0 |
| 219.10269 | 219.102668 | 1453382 | 13 | 16 | 0 | 3 | 0 | 0 |
| 219.139064 | 219.139053 | 1237447 | 14 | 20 | 0 | 2 | 0 | 0 |
| 221.118249 | 221.118318 | 1262767 | 13 | 18 | 0 | 3 | 0 | 0 |
| 223.061176 | 223.061197 | 1081891 | 11 | 12 | 0 | 5 | 0 | 0 |
| 223.097624 | 223.097583 | 2515141 | 12 | 16 | 0 | 4 | 0 | 0 |
| 223.133981 | 223.133968 | 1240159 | 13 | 20 | 0 | 3 | 0 | 0 |
| 225.076845 | 225.076847 | 1601809 | 11 | 14 | 0 | 5 | 0 | 0 |
| 225.113275 | 225.113233 | 1146867 | 12 | 18 | 0 | 4 | 0 | 0 |
| 225.128528 | 225.128489 | 1221780 | 16 | 18 | 0 | 1 | 0 | 0 |
| 227.10780 | 227.107753 | 1389868 | 15 | 16 | 0 | 2 | 0 | 0 |
| 227.144154 | 227.144139 | 1459247 | 16 | 20 | 0 | 1 | 0 | 0 |
| 229.087026 | 229.087018 | 1530401 | 14 | 14 | 0 | 3 | 0 | 0 |
| 229.123404 | 229.123403 | 2600339 | 15 | 18 | 0 | 2 | 0 | 0 |
| 229.159742 | 229.159789 | 1891158 | 16 | 22 | 0 | 1 | 0 | 0 |
| 231.102697 | 231.102668 | 3385667 | 14 | 16 | 0 | 3 | 0 | 0 |
| 231.139066 | 231.139053 | 4801094 | 15 | 20 | 0 | 2 | 0 | 0 |
| 231.17546 | 231.175439 | 1805064 | 16 | 24 | 0 | 1 | 0 | 0 |
| 233.081963 | 233.081932 | 2171997 | 13 | 14 | 0 | 4 | 0 | 0 |
| 233.118346 | 233.118318 | 4018848 | 14 | 18 | 0 | 3 | 0 | 0 |
| 233.154731 | 233.154703 | 3394659 | 15 | 22 | 0 | 2 | 0 | 0 |
| 235.097615 | 235.097583 | 3223300 | 13 | 16 | 0 | 4 | 0 | 0 |
| 235.134008 | 235.133968 | 4596167 | 14 | 20 | 0 | 3 | 0 | 0 |
| 235.170373 | 235.170354 | 2429386 | 15 | 24 | 0 | 2 | 0 | 0 |
| 237.076863 | 237.076847 | 2428209 | 12 | 14 | 0 | 5 | 0 | 0 |
| 237.113261 | 237.113233 | 5542132 | 13 | 18 | 0 | 4 | 0 | 0 |
| 237.128504 | 237.128489 | 1263190 | 17 | 18 | 0 | 1 | 0 | 0 |
| 237.14965 | 237.149618 | 4119800 | 14 | 22 | 0 | 3 | 0 | 0 |
| 237.186084 | 237.186004 | 1148827 | 15 | 26 | 0 | 2 | 0 | 0 |
| 239.092531 | 239.092497 | 3306026 | 12 | 16 | 0 | 5 | 0 | 0 |
| 239.107772 | 239.107753 | 2200171 | 16 | 16 | 0 | 2 | 0 | 0 |
| 239.128905 | 239.128883 | 2736973 | 13 | 20 | 0 | 4 | 0 | 0 |
| 239.144167 | 239.144139 | 2285038 | 17 | 20 | 0 | 1 | 0 | 0 |
| 239.165179 | 239.165268 | 1381616 | 14 | 24 | 0 | 3 | 0 | 0 |
| 241.087043 | 241.087018 | 2457448 | 15 | 14 | 0 | 3 | 0 | 0 |
| 241.108118 | 241.108147 | 1864842 | 12 | 18 | 0 | 5 | 0 | 0 |
| 241.123405 | 241.123403 | 5608171 | 16 | 18 | 0 | 2 | 0 | 0 |
| 241.159803 | 241.159789 | 4621039 | 17 | 22 | 0 | 1 | 0 | 0 |
| 243.066293 | 243.066282 | 2134891 | 14 | 12 | 0 | 4 | 0 | 0 |
| 243.102698 | 243.102668 | 4826031 | 15 | 16 | 0 | 3 | 0 | 0 |
| 243.13909 | 243.139053 | 8811570 | 16 | 20 | 0 | 2 | 0 | 0 |
| 243.175464 | 243.175439 | 4935862 | 17 | 24 | 0 | 1 | 0 | 0 |
| 245.081939 | 245.081932 | 3254520 | 14 | 14 | 0 | 4 | 0 | 0 |
| 245.118327 | 245.118318 | 8724476 | 15 | 18 | 0 | 3 | 0 | 0 |
| 245.154729 | 245.154703 | 9217535 | 16 | 22 | 0 | 2 | 0 | 0 |
| 245.191109 | 245.191089 | 3001987 | 17 | 26 | 0 | 1 | 0 | 0 |
| 247.061228 | 247.061197 | 1737606 | 13 | 12 | 0 | 5 | 0 | 0 |
| 247.097593 | 247.097583 | 7146315 | 14 | 16 | 0 | 4 | 0 | 0 |
| 247.133985 | 247.133968 | 13819086 | 15 | 20 | 0 | 3 | 0 | 0 |
| 247.170371 | 247.170354 | 7876946 | 16 | 24 | 0 | 2 | 0 | 0 |
| 247.206761 | 247.206739 | 1268598 | 17 | 28 | 0 | 1 | 0 | 0 |
| 249.076855 | 249.076847 | 3750941 | 13 | 14 | 0 | 5 | 0 | 0 |
| 249.113228 | 249.113233 | 9377697 | 14 | 18 | 0 | 4 | 0 | 0 |
| 249.128524 | 249.128489 | 1485795 | 18 | 18 | 0 | 1 | 0 | 0 |
| 249.149653 | 249.149618 | 11048869 | 15 | 22 | 0 | 3 | 0 | 0 |
| 249.186022 | 249.186004 | 2599785 | 16 | 26 | 0 | 2 | 0 | 0 |
| 251.07142 | 251.071368 | 1592054 | 16 | 12 | 0 | 3 | 0 | 0 |
| 251.092513 | 251.092497 | 8603256 | 13 | 16 | 0 | 5 | 0 | 0 |
| 251.107766 | 251.107753 | 3178234 | 17 | 16 | 0 | 2 | 0 | 0 |
| 251.128908 | 251.128883 | 15240828 | 14 | 20 | 0 | 4 | 0 | 0 |
| 251.144153 | 251.144139 | 3987710 | 18 | 20 | 0 | 1 | 0 | 0 |
| 251.18053 | 251.180524 | 1359938 | 19 | 24 | 0 | 0 | 0 | 0 |
| 251.201622 | 251.201654 | 1447236 | 16 | 28 | 0 | 2 | 0 | 0 |
| 253.071795 | 253.071762 | 2136530 | 12 | 14 | 0 | 6 | 0 | 0 |
| 253.087048 | 253.087018 | 2781715 | 16 | 14 | 0 | 3 | 0 | 0 |
| 253.108178 | 253.108147 | 6168790 | 13 | 18 | 0 | 5 | 0 | 0 |
| 253.123425 | 253.123403 | 5721560 | 17 | 18 | 0 | 2 | 0 | 0 |
| 253.144547 | 253.144533 | 7831898 | 14 | 22 | 0 | 4 | 0 | 0 |
| 253.159821 | 253.159789 | 5223772 | 18 | 22 | 0 | 1 | 0 | 0 |
| 255.066308 | 255.066282 | 2838447 | 15 | 12 | 0 | 4 | 0 | 0 |
| 255.087438 | 255.087412 | 3291314 | 12 | 16 | 0 | 6 | 0 | 0 |
| 255.102688 | 255.102668 | 8391219 | 16 | 16 | 0 | 3 | 0 | 0 |
| 255.123814 | 255.123797 | 5921078 | 13 | 20 | 0 | 5 | 0 | 0 |
| 255.139078 | 255.139053 | 16133175 | 17 | 20 | 0 | 2 | 0 | 0 |
| 255.175456 | 255.175439 | 11126843 | 18 | 24 | 0 | 1 | 0 | 0 |
| 256.134303 | 256.134302 | 1117735 | 16 | 19 | 1 | 2 | 0 | 0 |
| 257.045615 | 257.045547 | 1212941 | 14 | 10 | 0 | 5 | 0 | 0 |
| 257.081949 | 257.081932 | 5123601 | 15 | 14 | 0 | 4 | 0 | 0 |
| 257.118343 | 257.118318 | 13567253 | 16 | 18 | 0 | 3 | 0 | 0 |
| 257.139463 | 257.139447 | 1738775 | 13 | 22 | 0 | 5 | 0 | 0 |
| 257.154709 | 257.154703 | 19007256 | 17 | 22 | 0 | 2 | 0 | 0 |
| 257.191114 | 257.191089 | 8362525 | 18 | 26 | 0 | 1 | 0 | 0 |
| 259.061214 | 259.061197 | 2897391 | 14 | 12 | 0 | 5 | 0 | 0 |
| 259.097606 | 259.097583 | 11914739 | 15 | 16 | 0 | 4 | 0 | 0 |
| 259.133981 | 259.133968 | 26504182 | 16 | 20 | 0 | 3 | 0 | 0 |
| 259.170375 | 259.170354 | 23507962 | 17 | 24 | 0 | 2 | 0 | 0 |
| 259.206751 | 259.206739 | 5111551 | 18 | 28 | 0 | 1 | 0 | 0 |
| 261.076844 | 261.076847 | 4910033 | 14 | 14 | 0 | 5 | 0 | 0 |
| 261.09204 | 261.092103 | 1223122 | 18 | 14 | 0 | 2 | 0 | 0 |
| 261.113254 | 261.113233 | 16509653 | 15 | 18 | 0 | 4 | 0 | 0 |
| 261.128512 | 261.128489 | 1361622 | 19 | 18 | 0 | 1 | 0 | 0 |
| 261.149617 | 261.149618 | 24482008 | 16 | 22 | 0 | 3 | 0 | 0 |
| 261.186015 | 261.186004 | 11313373 | 17 | 26 | 0 | 2 | 0 | 0 |
| 261.222372 | 261.222389 | 1707873 | 18 | 30 | 0 | 1 | 0 | 0 |
| 263.056129 | 263.056112 | 2273518 | 13 | 12 | 0 | 6 | 0 | 0 |
| 263.071386 | 263.071368 | 1624943 | 17 | 12 | 0 | 3 | 0 | 0 |
| 263.092513 | 263.092497 | 10852274 | 14 | 16 | 0 | 5 | 0 | 0 |
| 263.107761 | 263.107753 | 2989108 | 18 | 16 | 0 | 2 | 0 | 0 |
| 263.128917 | 263.128883 | 28569014 | 15 | 20 | 0 | 4 | 0 | 0 |
| 263.14414 | 263.144139 | 3382328 | 19 | 20 | 0 | 1 | 0 | 0 |
| 263.165278 | 263.165268 | 21086650 | 16 | 24 | 0 | 3 | 0 | 0 |
| 263.201683 | 263.201654 | 4789694 | 17 | 28 | 0 | 2 | 0 | 0 |
| 265.071769 | 265.071762 | 2967693 | 13 | 14 | 0 | 6 | 0 | 0 |
| 265.087027 | 265.087018 | 3569551 | 17 | 14 | 0 | 3 | 0 | 0 |
| 265.108152 | 265.108147 | 15418001 | 14 | 18 | 0 | 5 | 0 | 0 |
| 265.123417 | 265.123403 | 7412115 | 18 | 18 | 0 | 2 | 0 | 0 |
| 265.144528 | 265.144533 | 31816342 | 15 | 22 | 0 | 4 | 0 | 0 |
| 265.159787 | 265.159789 | 6947735 | 19 | 22 | 0 | 1 | 0 | 0 |
| 265.180924 | 265.180918 | 9193625 | 16 | 26 | 0 | 3 | 0 | 0 |
| 265.217329 | 265.217304 | 1195837 | 17 | 30 | 0 | 2 | 0 | 0 |
| 266.094907 | 266.095154 | 1138494 | 13 | 18 | 1 | 3 | 0 | 1 |
| 266.118624 | 266.118652 | 1144640 | 17 | 17 | 1 | 2 | 0 | 0 |
| 267.066284 | 267.066282 | 3625704 | 16 | 12 | 0 | 4 | 0 | 0 |
| 267.087423 | 267.087412 | 5698411 | 13 | 16 | 0 | 6 | 0 | 0 |
| 267.102674 | 267.102668 | 9364332 | 17 | 16 | 0 | 3 | 0 | 0 |
| 267.123811 | 267.123797 | 18322798 | 14 | 20 | 0 | 5 | 0 | 0 |
| 267.139064 | 267.139053 | 16250224 | 18 | 20 | 0 | 2 | 0 | 0 |
| 267.175452 | 267.175439 | 12422004 | 19 | 24 | 0 | 1 | 0 | 0 |
| 268.077015 | 268.076788 | 1122422 | 19 | 11 | 1 | 1 | 0 | 0 |
| 268.097943 | 268.097917 | 1364440 | 16 | 15 | 1 | 3 | 0 | 0 |
| 268.110538 | 268.110804 | 2062425 | 13 | 20 | 1 | 3 | 0 | 1 |
| 269.045492 | 269.045547 | 1839550 | 15 | 10 | 0 | 5 | 0 | 0 |
| 269.066557 | 269.066676 | 1257664 | 12 | 14 | 0 | 7 | 0 | 0 |
| 269.081942 | 269.081932 | 7887682 | 16 | 14 | 0 | 4 | 0 | 0 |
| 269.103039 | 269.103062 | 6444101 | 13 | 18 | 0 | 6 | 0 | 0 |
| 269.118334 | 269.118318 | 20123718 | 17 | 18 | 0 | 3 | 0 | 0 |
| 269.139454 | 269.139447 | 14395464 | 14 | 22 | 0 | 5 | 0 | 0 |
| 269.154703 | 269.154703 | 31465546 | 18 | 22 | 0 | 2 | 0 | 0 |
| 269.175854 | 269.175833 | 10016332 | 15 | 26 | 0 | 4 | 0 | 0 |
| 269.191086 | 269.191089 | 15916110 | 19 | 26 | 0 | 1 | 0 | 0 |
| 270.077162 | 270.077181 | 1553260 | 15 | 13 | 1 | 4 | 0 | 0 |
| 270.113571 | 270.113567 | 1467280 | 16 | 17 | 1 | 3 | 0 | 0 |
| 270.126184 | 270.126454 | 2023602 | 13 | 22 | 1 | 3 | 0 | 1 |
| 271.061217 | 271.061197 | 3861012 | 15 | 12 | 0 | 5 | 0 | 0 |
| 271.097585 | 271.097583 | 12943640 | 16 | 16 | 0 | 4 | 0 | 0 |
| 271.118731 | 271.118712 | 2927770 | 13 | 20 | 0 | 6 | 0 | 0 |
| 271.133971 | 271.133968 | 30923036 | 17 | 20 | 0 | 3 | 0 | 0 |
| 271.155117 | 271.155097 | 5287710 | 14 | 24 | 0 | 5 | 0 | 0 |
| 271.170361 | 271.170354 | 34670880 | 18 | 24 | 0 | 2 | 0 | 0 |
| 271.191499 | 271.191483 | 1549026 | 15 | 28 | 0 | 4 | 0 | 0 |
| 271.206745 | 271.206739 | 8536356 | 19 | 28 | 0 | 1 | 0 | 0 |
| 272.092871 | 272.092832 | 1987776 | 15 | 15 | 1 | 4 | 0 | 0 |
| 272.129187 | 272.129217 | 1624516 | 16 | 19 | 1 | 3 | 0 | 0 |
| 272.141835 | 272.142104 | 1969989 | 13 | 24 | 1 | 3 | 0 | 1 |
| 273.040448 | 273.040462 | 1884706 | 14 | 10 | 0 | 6 | 0 | 0 |
| 273.076872 | 273.076847 | 8531430 | 15 | 14 | 0 | 5 | 0 | 0 |
| 273.113239 | 273.113233 | 26939882 | 16 | 18 | 0 | 4 | 0 | 0 |
| 273.128475 | 273.128489 | 1284779 | 20 | 18 | 0 | 1 | 0 | 0 |
| 273.1344 | 273.134362 | 1267116 | 13 | 22 | 0 | 6 | 0 | 0 |
| 273.149626 | 273.149618 | 51712492 | 17 | 22 | 0 | 3 | 0 | 0 |
| 273.170679 | 273.170747 | 1332655 | 14 | 26 | 0 | 5 | 0 | 0 |
| 273.186015 | 273.186004 | 34249200 | 18 | 26 | 0 | 2 | 0 | 0 |
| 273.207209 | 273.207133 | 1399219 | 15 | 30 | 0 | 4 | 0 | 0 |
| 273.222418 | 273.222389 | 4641013 | 19 | 30 | 0 | 1 | 0 | 0 |
| 274.077028 | 274.076804 | 1599180 | 12 | 13 | 5 | 1 | 1 | 0 |
| 274.108466 | 274.108482 | 2097359 | 15 | 17 | 1 | 4 | 0 | 0 |
| 274.144839 | 274.144867 | 2492754 | 16 | 21 | 1 | 3 | 0 | 0 |
| 274.157509 | 274.157754 | 1314132 | 13 | 26 | 1 | 3 | 0 | 1 |
| 275.056123 | 275.056112 | 2855854 | 14 | 12 | 0 | 6 | 0 | 0 |
| 275.071385 | 275.071368 | 1237487 | 18 | 12 | 0 | 3 | 0 | 0 |
| 275.092494 | 275.092497 | 12513457 | 15 | 16 | 0 | 5 | 0 | 0 |
| 275.095879 | 275.095868 | 1597874 | 12 | 20 | 0 | 5 | 1 | 0 |
| 275.10773 | 275.107753 | 1812339 | 19 | 16 | 0 | 2 | 0 | 0 |
| 275.128889 | 275.128883 | 32475830 | 16 | 20 | 0 | 4 | 0 | 0 |
| 275.144131 | 275.144139 | 2450103 | 20 | 20 | 0 | 1 | 0 | 0 |
| 275.165281 | 275.165268 | 39279288 | 17 | 24 | 0 | 3 | 0 | 0 |
| 275.201661 | 275.201654 | 15204028 | 18 | 28 | 0 | 2 | 0 | 0 |
| 276.08773 | 276.087746 | 1144916 | 14 | 15 | 1 | 5 | 0 | 0 |
| 276.124139 | 276.124132 | 1555223 | 15 | 19 | 1 | 4 | 0 | 0 |
| 277.071769 | 277.071762 | 4737396 | 14 | 14 | 0 | 6 | 0 | 0 |
| 277.087039 | 277.087018 | 2877685 | 18 | 14 | 0 | 3 | 0 | 0 |
| 277.10816 | 277.108147 | 20894584 | 15 | 18 | 0 | 5 | 0 | 0 |
| 277.123418 | 277.123403 | 6238073 | 19 | 18 | 0 | 2 | 0 | 0 |
| 277.144556 | 277.144533 | 40739708 | 16 | 22 | 0 | 4 | 0 | 0 |
| 277.159789 | 277.159789 | 5232252 | 20 | 22 | 0 | 1 | 0 | 0 |
| 277.180947 | 277.180918 | 27504510 | 17 | 26 | 0 | 3 | 0 | 0 |
| 277.217329 | 277.217304 | 4703874 | 18 | 30 | 0 | 2 | 0 | 0 |
| 278.094904 | 278.095154 | 1185365 | 14 | 18 | 1 | 3 | 0 | 1 |
| 278.103442 | 278.103396 | 1328662 | 14 | 17 | 1 | 5 | 0 | 0 |
| 278.116029 | 278.116283 | 2551127 | 11 | 22 | 1 | 5 | 0 | 1 |
| 278.131268 | 278.131539 | 1280409 | 15 | 22 | 1 | 2 | 0 | 1 |
| 278.139799 | 278.139782 | 1784666 | 15 | 21 | 1 | 4 | 0 | 0 |
| 279.066272 | 279.066282 | 3583280 | 17 | 12 | 0 | 4 | 0 | 0 |
| 279.087399 | 279.087412 | 7571506 | 14 | 16 | 0 | 6 | 0 | 0 |
| 279.102683 | 279.102668 | 8330804 | 18 | 16 | 0 | 3 | 0 | 0 |
| 279.123788 | 279.123797 | 35228724 | 15 | 20 | 0 | 5 | 0 | 0 |
| 279.139063 | 279.139053 | 13900343 | 19 | 20 | 0 | 2 | 0 | 0 |
| 279.160172 | 279.160183 | 36391992 | 16 | 24 | 0 | 4 | 0 | 0 |
| 279.175446 | 279.175439 | 9346618 | 20 | 24 | 0 | 1 | 0 | 0 |
| 279.196564 | 279.196568 | 11789372 | 17 | 28 | 0 | 3 | 0 | 0 |
| 279.2118 | 279.211824 | 1880894 | 21 | 28 | 0 | 0 | 0 | 0 |
| 281.066683 | 281.066676 | 1399334 | 13 | 14 | 0 | 7 | 0 | 0 |
| 281.08192 | 281.081932 | 6616551 | 17 | 14 | 0 | 4 | 0 | 0 |
| 281.103049 | 281.103062 | 10061545 | 14 | 18 | 0 | 6 | 0 | 0 |
| 281.118335 | 281.118318 | 14315242 | 18 | 18 | 0 | 3 | 0 | 0 |
| 281.139451 | 281.139447 | 31732972 | 15 | 22 | 0 | 5 | 0 | 0 |
| 281.154711 | 281.154703 | 21949678 | 19 | 22 | 0 | 2 | 0 | 0 |
| 281.175834 | 281.175833 | 15835375 | 16 | 26 | 0 | 4 | 0 | 0 |
| 281.19109 | 281.191089 | 11193585 | 20 | 26 | 0 | 1 | 0 | 0 |
| 281.212225 | 281.212218 | 2129011 | 17 | 30 | 0 | 3 | 0 | 0 |
| 282.077171 | 282.077181 | 1463742 | 16 | 13 | 1 | 4 | 0 | 0 |
| 282.113552 | 282.113567 | 1616065 | 17 | 17 | 1 | 3 | 0 | 0 |
| 282.149988 | 282.149952 | 1862981 | 18 | 21 | 1 | 2 | 0 | 0 |
| 283.061189 | 283.061197 | 4378770 | 16 | 12 | 0 | 5 | 0 | 0 |
| 283.082327 | 283.082326 | 2076820 | 13 | 16 | 0 | 7 | 0 | 0 |
| 283.097593 | 283.097583 | 14604693 | 17 | 16 | 0 | 4 | 0 | 0 |
| 283.100934 | 283.100953 | 1364758 | 14 | 20 | 0 | 4 | 1 | 0 |
| 283.108826 | 283.108816 | 1571926 | 16 | 16 | 2 | 3 | 0 | 0 |
| 283.118708 | 283.118712 | 12282775 | 14 | 20 | 0 | 6 | 0 | 0 |
| 283.133983 | 283.133968 | 34379160 | 18 | 20 | 0 | 3 | 0 | 0 |
| 283.145142 | 283.145201 | 1182233 | 17 | 20 | 2 | 2 | 0 | 0 |
| 283.155089 | 283.155097 | 28147098 | 15 | 24 | 0 | 5 | 0 | 0 |
| 283.170365 | 283.170354 | 42136988 | 19 | 24 | 0 | 2 | 0 | 0 |
| 283.206747 | 283.206739 | 13019550 | 20 | 28 | 0 | 1 | 0 | 0 |
| 284.069056 | 284.069333 | 1291238 | 12 | 16 | 1 | 5 | 0 | 1 |
| 284.092839 | 284.092832 | 1931944 | 16 | 15 | 1 | 4 | 0 | 0 |
| 284.129191 | 284.129217 | 2824683 | 17 | 19 | 1 | 3 | 0 | 0 |
| 284.141821 | 284.142104 | 2337772 | 14 | 24 | 1 | 3 | 0 | 1 |
| 284.165617 | 284.165603 | 1840750 | 18 | 23 | 1 | 2 | 0 | 0 |
| 285.040441 | 285.040462 | 2111540 | 15 | 10 | 0 | 6 | 0 | 0 |
| 285.07684 | 285.076847 | 8973879 | 16 | 14 | 0 | 5 | 0 | 0 |
| 285.08809 | 285.08808 | 1138296 | 15 | 14 | 2 | 4 | 0 | 0 |
| 285.097965 | 285.097976 | 2548665 | 13 | 18 | 0 | 7 | 0 | 0 |
| 285.113228 | 285.113233 | 26792506 | 17 | 18 | 0 | 4 | 0 | 0 |
| 285.124511 | 285.124466 | 1815739 | 16 | 18 | 2 | 3 | 0 | 0 |
| 285.134358 | 285.134362 | 8262204 | 14 | 22 | 0 | 6 | 0 | 0 |
| 285.149613 | 285.149618 | 54298172 | 18 | 22 | 0 | 3 | 0 | 0 |
| 285.17073 | 285.170747 | 11385407 | 15 | 26 | 0 | 5 | 0 | 0 |
| 285.186001 | 285.186004 | 46614080 | 19 | 26 | 0 | 2 | 0 | 0 |
| 285.222384 | 285.222389 | 7441731 | 20 | 30 | 0 | 1 | 0 | 0 |
| 286.072073 | 286.072096 | 1344005 | 15 | 13 | 1 | 5 | 0 | 0 |
| 286.084706 | 286.084983 | 1420102 | 12 | 18 | 1 | 5 | 0 | 1 |
| 286.108458 | 286.108482 | 2556424 | 16 | 17 | 1 | 4 | 0 | 0 |
| 286.144876 | 286.144867 | 2768011 | 17 | 21 | 1 | 3 | 0 | 0 |
| 287.056145 | 287.056112 | 2897487 | 15 | 12 | 0 | 6 | 0 | 0 |
| 287.092509 | 287.092497 | 12265170 | 16 | 16 | 0 | 5 | 0 | 0 |
| 287.107739 | 287.107753 | 1517843 | 20 | 16 | 0 | 2 | 0 | 0 |
| 287.128886 | 287.128883 | 36935380 | 17 | 20 | 0 | 4 | 0 | 0 |
| 287.14011 | 287.140116 | 1150198 | 16 | 20 | 2 | 3 | 0 | 0 |
| 287.144174 | 287.144139 | 1471382 | 21 | 20 | 0 | 1 | 0 | 0 |
| 287.150007 | 287.150012 | 2785494 | 14 | 24 | 0 | 6 | 0 | 0 |
| 287.165269 | 287.165268 | 54167256 | 18 | 24 | 0 | 3 | 0 | 0 |
| 287.186419 | 287.186398 | 1250777 | 15 | 28 | 0 | 5 | 0 | 0 |
| 287.201651 | 287.201654 | 29017818 | 19 | 28 | 0 | 2 | 0 | 0 |
| 287.238008 | 287.238039 | 2462557 | 20 | 32 | 0 | 1 | 0 | 0 |
| 288.087691 | 288.087746 | 1546971 | 15 | 15 | 1 | 5 | 0 | 0 |
| 288.124054 | 288.124132 | 2331165 | 16 | 19 | 1 | 4 | 0 | 0 |
| 288.136744 | 288.137018 | 2167966 | 13 | 24 | 1 | 4 | 0 | 1 |
| 288.160476 | 288.160517 | 1900832 | 17 | 23 | 1 | 3 | 0 | 0 |
| 289.03538 | 289.035376 | 1406878 | 14 | 10 | 0 | 7 | 0 | 0 |
| 289.071749 | 289.071762 | 6152544 | 15 | 14 | 0 | 6 | 0 | 0 |
| 289.087021 | 289.087018 | 2292321 | 19 | 14 | 0 | 3 | 0 | 0 |
| 289.108148 | 289.108147 | 23924578 | 16 | 18 | 0 | 5 | 0 | 0 |
| 289.119412 | 289.119381 | 1437284 | 15 | 18 | 2 | 4 | 0 | 0 |
| 289.123419 | 289.123403 | 3696100 | 20 | 18 | 0 | 2 | 0 | 0 |
| 289.144528 | 289.144533 | 59561828 | 17 | 22 | 0 | 4 | 0 | 0 |
| 289.159805 | 289.159789 | 3696742 | 21 | 22 | 0 | 1 | 0 | 0 |
| 289.180912 | 289.180918 | 53974888 | 18 | 26 | 0 | 3 | 0 | 0 |
| 289.196149 | 289.196174 | 1148457 | 22 | 26 | 0 | 0 | 0 | 0 |
| 289.217305 | 289.217304 | 14363498 | 19 | 30 | 0 | 2 | 0 | 0 |
| 290.103392 | 290.103396 | 1987879 | 15 | 17 | 1 | 5 | 0 | 0 |
| 290.139762 | 290.139782 | 2378921 | 16 | 21 | 1 | 4 | 0 | 0 |
| 290.176136 | 290.176167 | 1429227 | 17 | 25 | 1 | 3 | 0 | 0 |
| 291.050991 | 291.051026 | 1391077 | 14 | 12 | 0 | 7 | 0 | 0 |
| 291.066286 | 291.066282 | 2582758 | 18 | 12 | 0 | 4 | 0 | 0 |
| 291.087398 | 291.087412 | 8796647 | 15 | 16 | 0 | 6 | 0 | 0 |
| 291.102664 | 291.102668 | 5094888 | 19 | 16 | 0 | 3 | 0 | 0 |
| 291.123784 | 291.123797 | 31133674 | 16 | 20 | 0 | 5 | 0 | 0 |
| 291.134977 | 291.135031 | 1133130 | 15 | 20 | 2 | 4 | 0 | 0 |
| 291.139055 | 291.139053 | 7966187 | 20 | 20 | 0 | 2 | 0 | 0 |
| 291.160167 | 291.160183 | 57489388 | 17 | 24 | 0 | 4 | 0 | 0 |
| 291.175442 | 291.175439 | 7595501 | 21 | 24 | 0 | 1 | 0 | 0 |
| 291.196553 | 291.196568 | 29990894 | 18 | 28 | 0 | 3 | 0 | 0 |
| 291.23291 | 291.232954 | 2810097 | 19 | 32 | 0 | 2 | 0 | 0 |
| 292.119084 | 292.119046 | 1550889 | 15 | 19 | 1 | 5 | 0 | 0 |
| 292.134237 | 292.134302 | 1319402 | 19 | 19 | 1 | 2 | 0 | 0 |
| 292.155398 | 292.155432 | 1284011 | 16 | 23 | 1 | 4 | 0 | 0 |
| 293.066703 | 293.066676 | 1882083 | 14 | 14 | 0 | 7 | 0 | 0 |
| 293.081924 | 293.081932 | 4912740 | 18 | 14 | 0 | 4 | 0 | 0 |
| 293.103051 | 293.103062 | 11056229 | 15 | 18 | 0 | 6 | 0 | 0 |
| 293.118328 | 293.118318 | 10011238 | 19 | 18 | 0 | 3 | 0 | 0 |
| 293.139437 | 293.139447 | 34226280 | 16 | 22 | 0 | 5 | 0 | 0 |
| 293.154708 | 293.154703 | 14997608 | 20 | 22 | 0 | 2 | 0 | 0 |
| 293.191096 | 293.191089 | 9259626 | 21 | 26 | 0 | 1 | 0 | 0 |
| 293.212208 | 293.212218 | 8881260 | 18 | 30 | 0 | 3 | 0 | 0 |
| 293.227498 | 293.227475 | 1209836 | 22 | 30 | 0 | 0 | 0 | 0 |
| 294.113578 | 294.113567 | 1455073 | 18 | 17 | 1 | 3 | 0 | 0 |
| 294.126227 | 294.126454 | 1436449 | 15 | 22 | 1 | 3 | 0 | 1 |
| 294.147316 | 294.147583 | 1207651 | 12 | 26 | 1 | 5 | 0 | 1 |
| 294.14996 | 294.149952 | 1300803 | 19 | 21 | 1 | 2 | 0 | 0 |
| 295.061203 | 295.061197 | 4127703 | 17 | 12 | 0 | 5 | 0 | 0 |
| 295.082325 | 295.082326 | 2496216 | 14 | 16 | 0 | 7 | 0 | 0 |
| 295.097585 | 295.097583 | 11535577 | 18 | 16 | 0 | 4 | 0 | 0 |
| 295.108826 | 295.108816 | 1458777 | 17 | 16 | 2 | 3 | 0 | 0 |
| 295.118722 | 295.118712 | 19354842 | 15 | 20 | 0 | 6 | 0 | 0 |
| 295.133965 | 295.133968 | 23609562 | 19 | 20 | 0 | 3 | 0 | 0 |
| 295.155098 | 295.155097 | 34816220 | 16 | 24 | 0 | 5 | 0 | 0 |
| 295.170357 | 295.170354 | 27304156 | 20 | 24 | 0 | 2 | 0 | 0 |
| 295.191484 | 295.191483 | 18835678 | 17 | 28 | 0 | 4 | 0 | 0 |
| 295.20675 | 295.206739 | 12137695 | 21 | 28 | 0 | 1 | 0 | 0 |
| 296.092857 | 296.092832 | 1703567 | 17 | 15 | 1 | 4 | 0 | 0 |
| 296.129199 | 296.129217 | 2494609 | 18 | 19 | 1 | 3 | 0 | 0 |
| 296.141843 | 296.142104 | 2353042 | 15 | 24 | 1 | 3 | 0 | 1 |
| 296.165634 | 296.165603 | 1856915 | 19 | 23 | 1 | 2 | 0 | 0 |
| 297.040461 | 297.040462 | 1882689 | 16 | 10 | 0 | 6 | 0 | 0 |
| 297.076821 | 297.076847 | 7924547 | 17 | 14 | 0 | 5 | 0 | 0 |
| 297.088128 | 297.08808 | 1279012 | 16 | 14 | 2 | 4 | 0 | 0 |
| 297.097996 | 297.097976 | 3921732 | 14 | 18 | 0 | 7 | 0 | 0 |
| 297.11321 | 297.113233 | 20434246 | 18 | 18 | 0 | 4 | 0 | 0 |
| 297.116577 | 297.116603 | 1753925 | 15 | 22 | 0 | 4 | 1 | 0 |
| 297.124398 | 297.124466 | 1352838 | 17 | 18 | 2 | 3 | 0 | 0 |
| 297.134369 | 297.134362 | 19669318 | 15 | 22 | 0 | 6 | 0 | 0 |
| 297.149603 | 297.149618 | 42156360 | 19 | 22 | 0 | 3 | 0 | 0 |
| 297.170751 | 297.170747 | 19538248 | 16 | 26 | 0 | 5 | 0 | 0 |
| 297.185987 | 297.186004 | 34832712 | 20 | 26 | 0 | 2 | 0 | 0 |
| 297.207152 | 297.207133 | 4562506 | 17 | 30 | 0 | 4 | 0 | 0 |
| 297.222375 | 297.222389 | 9511755 | 21 | 30 | 0 | 1 | 0 | 0 |
| 298.108498 | 298.108482 | 1565176 | 17 | 17 | 1 | 4 | 0 | 0 |
| 298.14488 | 298.144867 | 2473082 | 18 | 21 | 1 | 3 | 0 | 0 |
| 298.181234 | 298.181253 | 1409659 | 19 | 25 | 1 | 2 | 0 | 0 |
| 299.056121 | 299.056112 | 2693414 | 16 | 12 | 0 | 6 | 0 | 0 |
| 299.092492 | 299.092497 | 12388776 | 17 | 16 | 0 | 5 | 0 | 0 |
| 299.103764 | 299.103731 | 1732008 | 16 | 16 | 2 | 4 | 0 | 0 |
| 299.113619 | 299.113627 | 2849449 | 14 | 20 | 0 | 7 | 0 | 0 |
| 299.128878 | 299.128883 | 29510058 | 18 | 20 | 0 | 4 | 0 | 0 |
| 299.140091 | 299.140116 | 1234666 | 17 | 20 | 2 | 3 | 0 | 0 |
| 299.15001 | 299.150012 | 9867178 | 15 | 24 | 0 | 6 | 0 | 0 |
| 299.165273 | 299.165268 | 48581036 | 19 | 24 | 0 | 3 | 0 | 0 |
| 299.186398 | 299.186398 | 5696428 | 16 | 28 | 0 | 5 | 0 | 0 |
| 299.201658 | 299.201654 | 28074412 | 20 | 28 | 0 | 2 | 0 | 0 |
| 299.222768 | 299.222783 | 1238830 | 17 | 32 | 0 | 4 | 0 | 0 |
| 299.238069 | 299.238039 | 4054191 | 21 | 32 | 0 | 1 | 0 | 0 |
| 300.087765 | 300.087746 | 1524567 | 16 | 15 | 1 | 5 | 0 | 0 |
| 300.124115 | 300.124132 | 2128216 | 17 | 19 | 1 | 4 | 0 | 0 |
| 300.160475 | 300.160517 | 2151642 | 18 | 23 | 1 | 3 | 0 | 0 |
| 301.071775 | 301.071762 | 4774403 | 16 | 14 | 0 | 6 | 0 | 0 |
| 301.087084 | 301.087018 | 1535620 | 20 | 14 | 0 | 3 | 0 | 0 |
| 301.108157 | 301.108147 | 17655300 | 17 | 18 | 0 | 5 | 0 | 0 |
| 301.11943 | 301.119381 | 1837189 | 16 | 18 | 2 | 4 | 0 | 0 |
| 301.123406 | 301.123403 | 2125446 | 21 | 18 | 0 | 2 | 0 | 0 |
| 301.129256 | 301.129277 | 2063622 | 14 | 22 | 0 | 7 | 0 | 0 |
| 301.144538 | 301.144533 | 46158344 | 18 | 22 | 0 | 4 | 0 | 0 |
| 301.159733 | 301.159789 | 1895687 | 22 | 22 | 0 | 1 | 0 | 0 |
| 301.165647 | 301.165662 | 4403464 | 15 | 26 | 0 | 6 | 0 | 0 |
| 301.180918 | 301.180918 | 58333704 | 19 | 26 | 0 | 3 | 0 | 0 |
| 301.202042 | 301.202048 | 1237705 | 16 | 30 | 0 | 5 | 0 | 0 |
| 301.217302 | 301.217304 | 16579082 | 20 | 30 | 0 | 2 | 0 | 0 |
| 301.253679 | 301.253689 | 1881099 | 21 | 34 | 0 | 1 | 0 | 0 |
| 302.103395 | 302.103396 | 2066992 | 16 | 17 | 1 | 5 | 0 | 0 |
| 302.139763 | 302.139782 | 2536242 | 17 | 21 | 1 | 4 | 0 | 0 |
| 302.17618 | 302.176167 | 2234675 | 18 | 25 | 1 | 3 | 0 | 0 |
| 303.051052 | 303.051026 | 2233688 | 15 | 12 | 0 | 7 | 0 | 0 |
| 303.066241 | 303.066282 | 1239768 | 19 | 12 | 0 | 4 | 0 | 0 |
| 303.087403 | 303.087412 | 8903769 | 16 | 16 | 0 | 6 | 0 | 0 |
| 303.102676 | 303.102668 | 2777434 | 20 | 16 | 0 | 3 | 0 | 0 |
| 303.123787 | 303.123797 | 32066138 | 17 | 20 | 0 | 5 | 0 | 0 |
| 303.135105 | 303.135031 | 1220187 | 16 | 20 | 2 | 4 | 0 | 0 |
| 303.139069 | 303.139053 | 4392795 | 21 | 20 | 0 | 2 | 0 | 0 |
| 303.160171 | 303.160183 | 60844636 | 18 | 24 | 0 | 4 | 0 | 0 |
| 303.175464 | 303.175439 | 4263261 | 22 | 24 | 0 | 1 | 0 | 0 |
| 303.196555 | 303.196568 | 52017756 | 19 | 28 | 0 | 3 | 0 | 0 |
| 303.232939 | 303.232954 | 7926879 | 20 | 32 | 0 | 2 | 0 | 0 |
| 304.082642 | 304.082661 | 1194241 | 15 | 15 | 1 | 6 | 0 | 0 |
| 304.11909 | 304.119046 | 1694339 | 16 | 19 | 1 | 5 | 0 | 0 |
| 304.15538 | 304.155432 | 2303364 | 17 | 23 | 1 | 4 | 0 | 0 |
| 304.191809 | 304.191817 | 1498245 | 18 | 27 | 1 | 3 | 0 | 0 |
| 305.066653 | 305.066676 | 2498471 | 15 | 14 | 0 | 7 | 0 | 0 |
| 305.081896 | 305.081932 | 3461032 | 19 | 14 | 0 | 4 | 0 | 0 |
| 305.103051 | 305.103062 | 12446376 | 16 | 18 | 0 | 6 | 0 | 0 |
| 305.118306 | 305.118318 | 6467753 | 20 | 18 | 0 | 3 | 0 | 0 |
| 305.139439 | 305.139447 | 36407976 | 17 | 22 | 0 | 5 | 0 | 0 |
| 305.154694 | 305.154703 | 9727658 | 21 | 22 | 0 | 2 | 0 | 0 |
| 305.175828 | 305.175833 | 50387628 | 18 | 26 | 0 | 4 | 0 | 0 |
| 305.19107 | 305.191089 | 6552748 | 22 | 26 | 0 | 1 | 0 | 0 |
| 305.227394 | 305.227475 | 1171565 | 23 | 30 | 0 | 0 | 0 | 0 |
| 305.248621 | 305.248604 | 2466478 | 20 | 34 | 0 | 2 | 0 | 0 |
| 306.113561 | 306.113567 | 1143118 | 19 | 17 | 1 | 3 | 0 | 0 |
| 306.126156 | 306.126454 | 1345358 | 16 | 22 | 1 | 3 | 0 | 1 |
| 306.134726 | 306.134696 | 1374671 | 16 | 21 | 1 | 5 | 0 | 0 |
| 306.171126 | 306.171082 | 1347792 | 17 | 25 | 1 | 4 | 0 | 0 |
| 307.06119 | 307.061197 | 2379119 | 18 | 12 | 0 | 5 | 0 | 0 |
| 307.082311 | 307.082326 | 3126000 | 15 | 16 | 0 | 7 | 0 | 0 |
| 307.097569 | 307.097583 | 5840625 | 19 | 16 | 0 | 4 | 0 | 0 |
| 307.118708 | 307.118712 | 15163121 | 16 | 20 | 0 | 6 | 0 | 0 |
| 307.133951 | 307.133968 | 12230386 | 20 | 20 | 0 | 3 | 0 | 0 |
| 307.155099 | 307.155097 | 38517492 | 17 | 24 | 0 | 5 | 0 | 0 |
| 307.17034 | 307.170354 | 17082100 | 21 | 24 | 0 | 2 | 0 | 0 |
| 307.191481 | 307.191483 | 30153460 | 18 | 28 | 0 | 4 | 0 | 0 |
| 307.206735 | 307.206739 | 8581364 | 22 | 28 | 0 | 1 | 0 | 0 |
| 307.227854 | 307.227868 | 6749941 | 19 | 32 | 0 | 3 | 0 | 0 |
| 308.105451 | 308.105718 | 1662355 | 15 | 20 | 1 | 4 | 0 | 1 |
| 308.12923 | 308.129217 | 1374548 | 19 | 19 | 1 | 3 | 0 | 0 |
| 308.141802 | 308.142104 | 1631188 | 16 | 24 | 1 | 3 | 0 | 1 |
| 308.150264 | 308.150346 | 1204116 | 16 | 23 | 1 | 5 | 0 | 0 |
| 309.076841 | 309.076847 | 4993331 | 18 | 14 | 0 | 5 | 0 | 0 |
| 309.097982 | 309.097976 | 4673332 | 15 | 18 | 0 | 7 | 0 | 0 |
| 309.11321 | 309.113233 | 12978996 | 19 | 18 | 0 | 4 | 0 | 0 |
| 309.134372 | 309.134362 | 20922164 | 16 | 22 | 0 | 6 | 0 | 0 |
| 309.149611 | 309.149618 | 23857972 | 20 | 22 | 0 | 3 | 0 | 0 |
| 309.170747 | 309.170747 | 31699766 | 17 | 26 | 0 | 5 | 0 | 0 |
| 309.186002 | 309.186004 | 25271094 | 21 | 26 | 0 | 2 | 0 | 0 |
| 309.207127 | 309.207133 | 14345015 | 18 | 30 | 0 | 4 | 0 | 0 |
| 309.22239 | 309.222389 | 9340215 | 22 | 30 | 0 | 1 | 0 | 0 |
| 309.24359 | 309.243519 | 1287608 | 19 | 34 | 0 | 3 | 0 | 0 |
| 310.084708 | 310.084983 | 1214674 | 14 | 18 | 1 | 5 | 0 | 1 |
| 310.10845 | 310.108482 | 1967827 | 18 | 17 | 1 | 4 | 0 | 0 |
| 310.14488 | 310.144867 | 2286292 | 19 | 21 | 1 | 3 | 0 | 0 |
| 310.181233 | 310.181253 | 1264341 | 20 | 25 | 1 | 2 | 0 | 0 |
| 311.056123 | 311.056112 | 3098224 | 17 | 12 | 0 | 6 | 0 | 0 |
| 311.092493 | 311.092497 | 9878385 | 18 | 16 | 0 | 5 | 0 | 0 |
| 311.10374 | 311.103731 | 1545457 | 17 | 16 | 2 | 4 | 0 | 0 |
| 311.113647 | 311.113627 | 6521713 | 15 | 20 | 0 | 7 | 0 | 0 |
| 311.128864 | 311.128883 | 24566642 | 19 | 20 | 0 | 4 | 0 | 0 |
| 311.132225 | 311.132253 | 1813746 | 16 | 24 | 0 | 4 | 1 | 0 |
| 311.150025 | 311.150012 | 18337650 | 16 | 24 | 0 | 6 | 0 | 0 |
| 311.165264 | 311.165268 | 37577588 | 20 | 24 | 0 | 3 | 0 | 0 |
| 311.186396 | 311.186398 | 16357235 | 17 | 28 | 0 | 5 | 0 | 0 |
| 311.201662 | 311.201654 | 27239284 | 21 | 28 | 0 | 2 | 0 | 0 |
| 311.238044 | 311.238039 | 6594421 | 22 | 32 | 0 | 1 | 0 | 0 |
| 312.087793 | 312.087746 | 1344013 | 17 | 15 | 1 | 5 | 0 | 0 |
| 312.100322 | 312.100633 | 1270478 | 14 | 20 | 1 | 5 | 0 | 1 |
| 312.124122 | 312.124132 | 2283662 | 18 | 19 | 1 | 4 | 0 | 0 |
| 312.136722 | 312.137018 | 2032655 | 15 | 24 | 1 | 4 | 0 | 1 |
| 312.160499 | 312.160517 | 2225679 | 19 | 23 | 1 | 3 | 0 | 0 |
| 313.07175 | 313.071762 | 4358825 | 17 | 14 | 0 | 6 | 0 | 0 |
| 313.108132 | 313.108147 | 14196650 | 18 | 18 | 0 | 5 | 0 | 0 |
| 313.119365 | 313.119381 | 1619370 | 17 | 18 | 2 | 4 | 0 | 0 |
| 313.129281 | 313.129277 | 5073578 | 15 | 22 | 0 | 7 | 0 | 0 |
| 313.144516 | 313.144533 | 35322796 | 19 | 22 | 0 | 4 | 0 | 0 |
| 313.16567 | 313.165662 | 8236459 | 16 | 26 | 0 | 6 | 0 | 0 |
| 313.180904 | 313.180918 | 41565100 | 20 | 26 | 0 | 3 | 0 | 0 |
| 313.202037 | 313.202048 | 4293036 | 17 | 30 | 0 | 5 | 0 | 0 |
| 313.217302 | 313.217304 | 20429740 | 21 | 30 | 0 | 2 | 0 | 0 |
| 313.253694 | 313.253689 | 2483118 | 22 | 34 | 0 | 1 | 0 | 0 |
| 314.103428 | 314.103396 | 1328196 | 17 | 17 | 1 | 5 | 0 | 0 |
| 314.115979 | 314.116283 | 1342916 | 14 | 22 | 1 | 5 | 0 | 1 |
| 314.139769 | 314.139782 | 2065477 | 18 | 21 | 1 | 4 | 0 | 0 |
| 314.176179 | 314.176167 | 1712070 | 19 | 25 | 1 | 3 | 0 | 0 |
| 315.051063 | 315.051026 | 1666396 | 16 | 12 | 0 | 7 | 0 | 0 |
| 315.087409 | 315.087412 | 6347741 | 17 | 16 | 0 | 6 | 0 | 0 |
| 315.102677 | 315.102668 | 1445598 | 21 | 16 | 0 | 3 | 0 | 0 |
| 315.123799 | 315.123797 | 21216222 | 18 | 20 | 0 | 5 | 0 | 0 |
| 315.127243 | 315.127168 | 1362206 | 15 | 24 | 0 | 5 | 1 | 0 |
| 315.135139 | 315.135031 | 1347678 | 17 | 20 | 2 | 4 | 0 | 0 |
| 315.139085 | 315.139053 | 1780830 | 22 | 20 | 0 | 2 | 0 | 0 |
| 315.144939 | 315.144927 | 2322399 | 15 | 24 | 0 | 7 | 0 | 0 |
| 315.160176 | 315.160183 | 44559328 | 19 | 24 | 0 | 4 | 0 | 0 |
| 315.175451 | 315.175439 | 1387103 | 23 | 24 | 0 | 1 | 0 | 0 |
| 315.196557 | 315.196568 | 39943136 | 20 | 28 | 0 | 3 | 0 | 0 |
| 315.232941 | 315.232954 | 8839137 | 21 | 32 | 0 | 2 | 0 | 0 |
| 316.11903 | 316.119046 | 1649270 | 17 | 19 | 1 | 5 | 0 | 0 |
| 316.15544 | 316.155432 | 2278647 | 18 | 23 | 1 | 4 | 0 | 0 |
| 316.191819 | 316.191817 | 1296248 | 19 | 27 | 1 | 3 | 0 | 0 |
| 317.066666 | 317.066676 | 2664461 | 16 | 14 | 0 | 7 | 0 | 0 |
| 317.081941 | 317.081932 | 1414029 | 20 | 14 | 0 | 4 | 0 | 0 |
| 317.103053 | 317.103062 | 10616846 | 17 | 18 | 0 | 6 | 0 | 0 |
| 317.114345 | 317.114295 | 1184270 | 16 | 18 | 2 | 5 | 0 | 0 |
| 317.118322 | 317.118318 | 3515918 | 21 | 18 | 0 | 3 | 0 | 0 |
| 317.139451 | 317.139447 | 30219278 | 18 | 22 | 0 | 5 | 0 | 0 |
| 317.154673 | 317.154703 | 4223247 | 22 | 22 | 0 | 2 | 0 | 0 |
| 317.160575 | 317.160577 | 1192591 | 15 | 26 | 0 | 7 | 0 | 0 |
| 317.175843 | 317.175833 | 50719760 | 19 | 26 | 0 | 4 | 0 | 0 |
| 317.191077 | 317.191089 | 3149072 | 23 | 26 | 0 | 1 | 0 | 0 |
| 317.212224 | 317.212218 | 29350928 | 20 | 30 | 0 | 3 | 0 | 0 |
| 317.24858 | 317.248604 | 2696465 | 21 | 34 | 0 | 2 | 0 | 0 |
| 318.134666 | 318.134696 | 1765797 | 17 | 21 | 1 | 5 | 0 | 0 |
| 318.171041 | 318.171082 | 2018470 | 18 | 25 | 1 | 4 | 0 | 0 |
| 319.061179 | 319.061197 | 1589945 | 19 | 12 | 0 | 5 | 0 | 0 |
| 319.082325 | 319.082326 | 3388218 | 16 | 16 | 0 | 7 | 0 | 0 |
| 319.097566 | 319.097583 | 4879930 | 20 | 16 | 0 | 4 | 0 | 0 |
| 319.11873 | 319.118712 | 15924282 | 17 | 20 | 0 | 6 | 0 | 0 |
| 319.133975 | 319.133968 | 7092795 | 21 | 20 | 0 | 3 | 0 | 0 |
| 319.155096 | 319.155097 | 39255100 | 18 | 24 | 0 | 5 | 0 | 0 |
| 319.170362 | 319.170354 | 9269307 | 22 | 24 | 0 | 2 | 0 | 0 |
| 319.191473 | 319.191483 | 53259324 | 19 | 28 | 0 | 4 | 0 | 0 |
| 319.206737 | 319.206739 | 5785660 | 23 | 28 | 0 | 1 | 0 | 0 |
| 320.141828 | 320.142104 | 1242768 | 17 | 24 | 1 | 3 | 0 | 1 |
| 320.150364 | 320.150346 | 1473360 | 17 | 23 | 1 | 5 | 0 | 0 |
| 321.076845 | 321.076847 | 3107171 | 19 | 14 | 0 | 5 | 0 | 0 |
| 321.09796 | 321.097976 | 4480099 | 16 | 18 | 0 | 7 | 0 | 0 |
| 321.113222 | 321.113233 | 6441060 | 20 | 18 | 0 | 4 | 0 | 0 |
| 321.134348 | 321.134362 | 19049572 | 17 | 22 | 0 | 6 | 0 | 0 |
| 321.149629 | 321.149618 | 12331108 | 21 | 22 | 0 | 3 | 0 | 0 |
| 321.170731 | 321.170747 | 37465188 | 18 | 26 | 0 | 5 | 0 | 0 |
| 321.185993 | 321.186004 | 14460005 | 22 | 26 | 0 | 2 | 0 | 0 |
| 321.207134 | 321.207133 | 28925030 | 19 | 30 | 0 | 4 | 0 | 0 |
| 321.222374 | 321.222389 | 6559846 | 23 | 30 | 0 | 1 | 0 | 0 |
| 321.24354 | 321.243519 | 2789606 | 20 | 34 | 0 | 3 | 0 | 0 |
| 322.108414 | 322.108482 | 1319799 | 19 | 17 | 1 | 4 | 0 | 0 |
| 322.144843 | 322.144867 | 1660152 | 20 | 21 | 1 | 3 | 0 | 0 |
| 323.056125 | 323.056112 | 1473801 | 18 | 12 | 0 | 6 | 0 | 0 |
| 323.092495 | 323.092497 | 4918410 | 19 | 16 | 0 | 5 | 0 | 0 |
| 323.103744 | 323.103731 | 1256970 | 18 | 16 | 2 | 4 | 0 | 0 |
| 323.113623 | 323.113627 | 5682314 | 16 | 20 | 0 | 7 | 0 | 0 |
| 323.12887 | 323.128883 | 11342986 | 20 | 20 | 0 | 4 | 0 | 0 |
| 323.150001 | 323.150012 | 20915338 | 17 | 24 | 0 | 6 | 0 | 0 |
| 323.165275 | 323.165268 | 19897484 | 21 | 24 | 0 | 3 | 0 | 0 |
| 323.168618 | 323.168639 | 1361035 | 18 | 28 | 0 | 3 | 1 | 0 |
| 323.186382 | 323.186398 | 24902796 | 18 | 28 | 0 | 5 | 0 | 0 |
| 323.20165 | 323.201654 | 19057804 | 22 | 28 | 0 | 2 | 0 | 0 |
| 323.222786 | 323.222783 | 10325132 | 19 | 32 | 0 | 4 | 0 | 0 |
| 323.238025 | 323.238039 | 6272653 | 23 | 32 | 0 | 1 | 0 | 0 |
| 324.124147 | 324.124132 | 1430429 | 19 | 19 | 1 | 4 | 0 | 0 |
| 324.1367 | 324.137018 | 1642269 | 16 | 24 | 1 | 4 | 0 | 1 |
| 324.160534 | 324.160517 | 1433117 | 20 | 23 | 1 | 3 | 0 | 0 |
| 324.173097 | 324.173404 | 1533342 | 17 | 28 | 1 | 3 | 0 | 1 |
| 325.071783 | 325.071762 | 3151278 | 18 | 14 | 0 | 6 | 0 | 0 |
| 325.108153 | 325.108147 | 9419950 | 19 | 18 | 0 | 5 | 0 | 0 |
| 325.129274 | 325.129277 | 7872175 | 16 | 22 | 0 | 7 | 0 | 0 |
| 325.144523 | 325.144533 | 19850414 | 20 | 22 | 0 | 4 | 0 | 0 |
| 325.147886 | 325.147904 | 1239983 | 17 | 26 | 0 | 4 | 1 | 0 |
| 325.165673 | 325.165662 | 15175855 | 17 | 26 | 0 | 6 | 0 | 0 |
| 325.180926 | 325.180918 | 26299568 | 21 | 26 | 0 | 3 | 0 | 0 |
| 325.20204 | 325.202048 | 11123888 | 18 | 30 | 0 | 5 | 0 | 0 |
| 325.21731 | 325.217304 | 19362992 | 22 | 30 | 0 | 2 | 0 | 0 |
| 325.238407 | 325.238433 | 2213553 | 19 | 34 | 0 | 4 | 0 | 0 |
| 325.253698 | 325.253689 | 4114097 | 23 | 34 | 0 | 1 | 0 | 0 |
| 325.274864 | 325.274819 | 1800241 | 20 | 38 | 0 | 3 | 0 | 0 |
| 326.103454 | 326.103396 | 1312960 | 18 | 17 | 1 | 5 | 0 | 0 |
| 326.115959 | 326.116283 | 1220672 | 15 | 22 | 1 | 5 | 0 | 1 |
| 326.139792 | 326.139782 | 1948608 | 19 | 21 | 1 | 4 | 0 | 0 |
| 326.152361 | 326.152668 | 1345217 | 16 | 26 | 1 | 4 | 0 | 1 |
| 326.176212 | 326.176167 | 1527617 | 20 | 25 | 1 | 3 | 0 | 0 |
| 327.051098 | 327.051026 | 1203536 | 17 | 12 | 0 | 7 | 0 | 0 |
| 327.087415 | 327.087412 | 6027473 | 18 | 16 | 0 | 6 | 0 | 0 |
| 327.108618 | 327.108541 | 1258065 | 15 | 20 | 0 | 8 | 0 | 0 |
| 327.12379 | 327.123797 | 17546450 | 19 | 20 | 0 | 5 | 0 | 0 |
| 327.135084 | 327.135031 | 1253073 | 18 | 20 | 2 | 4 | 0 | 0 |
| 327.144942 | 327.144927 | 5648594 | 16 | 24 | 0 | 7 | 0 | 0 |
| 327.16019 | 327.160183 | 29940946 | 20 | 24 | 0 | 4 | 0 | 0 |
| 327.163585 | 327.163554 | 2260178 | 17 | 28 | 0 | 4 | 1 | 0 |
| 327.181299 | 327.181312 | 6571730 | 17 | 28 | 0 | 6 | 0 | 0 |
| 327.196567 | 327.196568 | 30766290 | 21 | 28 | 0 | 3 | 0 | 0 |
| 327.217706 | 327.217698 | 4392147 | 18 | 32 | 0 | 5 | 0 | 0 |
| 327.23294 | 327.232954 | 21454034 | 22 | 32 | 0 | 2 | 0 | 0 |
| 327.269295 | 327.269339 | 1582292 | 23 | 36 | 0 | 1 | 0 | 0 |
| 328.119041 | 328.119046 | 1299170 | 18 | 19 | 1 | 5 | 0 | 0 |
| 328.131654 | 328.131933 | 1190242 | 15 | 24 | 1 | 5 | 0 | 1 |
| 328.155437 | 328.155432 | 2148450 | 19 | 23 | 1 | 4 | 0 | 0 |
| 328.191807 | 328.191817 | 1803363 | 20 | 27 | 1 | 3 | 0 | 0 |
| 329.066687 | 329.066676 | 2720753 | 17 | 14 | 0 | 7 | 0 | 0 |
| 329.103052 | 329.103062 | 10659058 | 18 | 18 | 0 | 6 | 0 | 0 |
| 329.106426 | 329.106433 | 1504498 | 15 | 22 | 0 | 6 | 1 | 0 |
| 329.118318 | 329.118318 | 1786226 | 22 | 18 | 0 | 3 | 0 | 0 |
| 329.139457 | 329.139447 | 25591026 | 19 | 22 | 0 | 5 | 0 | 0 |
| 329.154724 | 329.154703 | 2371571 | 23 | 22 | 0 | 2 | 0 | 0 |
| 329.16057 | 329.160577 | 2555891 | 16 | 26 | 0 | 7 | 0 | 0 |
| 329.175826 | 329.175833 | 46746868 | 20 | 26 | 0 | 4 | 0 | 0 |
| 329.191117 | 329.191089 | 1303603 | 24 | 26 | 0 | 1 | 0 | 0 |
| 329.196968 | 329.196962 | 2710771 | 17 | 30 | 0 | 6 | 0 | 0 |
| 329.212202 | 329.212218 | 24980724 | 21 | 30 | 0 | 3 | 0 | 0 |
| 329.233359 | 329.233348 | 1930228 | 18 | 34 | 0 | 5 | 0 | 0 |
| 329.248602 | 329.248604 | 7396084 | 22 | 34 | 0 | 2 | 0 | 0 |
| 330.134706 | 330.134696 | 1607938 | 18 | 21 | 1 | 5 | 0 | 0 |
| 330.17109 | 330.171082 | 1677699 | 19 | 25 | 1 | 4 | 0 | 0 |
| 331.082317 | 331.082326 | 3286290 | 17 | 16 | 0 | 7 | 0 | 0 |
| 331.097569 | 331.097583 | 1977874 | 21 | 16 | 0 | 4 | 0 | 0 |
| 331.118688 | 331.118712 | 12429586 | 18 | 20 | 0 | 6 | 0 | 0 |
| 331.122071 | 331.122083 | 1325266 | 15 | 24 | 0 | 6 | 1 | 0 |
| 331.133973 | 331.133968 | 3965202 | 22 | 20 | 0 | 3 | 0 | 0 |
| 331.155095 | 331.155097 | 31665426 | 19 | 24 | 0 | 5 | 0 | 0 |
| 331.170309 | 331.170354 | 4584723 | 23 | 24 | 0 | 2 | 0 | 0 |
| 331.191482 | 331.191483 | 49380628 | 20 | 28 | 0 | 4 | 0 | 0 |
[truncated: 249,108 more chars]
