# Supplementary material for: Use of ESI-FTICR-MS to Characterize Dissolved Organic Matter in Headwater Streams Draining Forest-Dominated and Pasture-Dominated Watersheds
Source: PLoS One. 2015 Dec 29;10(12):e0145639. doi: 10.1371/journal.pone.0145639 (PMC4694922; doi:10.1371/journal.pone.0145639)
Supplement: S5 Appendix — (DOCX) [file pone.0145639.s005.docx]

**S5 Appendix V: Parameters for peaks with assigned formulas in T_15_ samples (sample number continued from S4 Appendix IV).**

5. Sample: F1_T_15_ (bacteria-only)

| Experimental mass | Exact mass | Peak height | C | H | N | O | S | P |
| --- | --- | --- | --- | --- | --- | --- | --- | --- |
| 215.107756 | 215.107753 | 1556067 | 14 | 16 | 0 | 2 | 0 | 0 |
| 217.086826 | 217.087018 | 1461678 | 13 | 14 | 0 | 3 | 0 | 0 |
| 217.123399 | 217.123403 | 2061535 | 14 | 18 | 0 | 2 | 0 | 0 |
| 219.066223 | 219.066282 | 1418154 | 12 | 12 | 0 | 4 | 0 | 0 |
| 219.102677 | 219.102668 | 1731276 | 13 | 16 | 0 | 3 | 0 | 0 |
| 219.13908 | 219.139053 | 1563677 | 14 | 20 | 0 | 2 | 0 | 0 |
| 221.081931 | 221.081932 | 1374442 | 12 | 14 | 0 | 4 | 0 | 0 |
| 221.118338 | 221.118318 | 1318204 | 13 | 18 | 0 | 3 | 0 | 0 |
| 223.097583 | 223.097583 | 1857915 | 12 | 16 | 0 | 4 | 0 | 0 |
| 225.076862 | 225.076847 | 1867675 | 11 | 14 | 0 | 5 | 0 | 0 |
| 227.107802 | 227.107753 | 1693357 | 15 | 16 | 0 | 2 | 0 | 0 |
| 227.144162 | 227.144139 | 1859184 | 16 | 20 | 0 | 1 | 0 | 0 |
| 229.086954 | 229.087018 | 1535335 | 14 | 14 | 0 | 3 | 0 | 0 |
| 229.123415 | 229.123403 | 3050574 | 15 | 18 | 0 | 2 | 0 | 0 |
| 231.102541 | 231.102668 | 2081170 | 14 | 16 | 0 | 3 | 0 | 0 |
| 231.139063 | 231.139053 | 2819485 | 15 | 20 | 0 | 2 | 0 | 0 |
| 233.081939 | 233.081932 | 2971443 | 13 | 14 | 0 | 4 | 0 | 0 |
| 233.118339 | 233.118318 | 3997494 | 14 | 18 | 0 | 3 | 0 | 0 |
| 233.154744 | 233.154703 | 2572410 | 15 | 22 | 0 | 2 | 0 | 0 |
| 235.061233 | 235.061197 | 1679959 | 12 | 12 | 0 | 5 | 0 | 0 |
| 235.097645 | 235.097583 | 3479803 | 13 | 16 | 0 | 4 | 0 | 0 |
| 235.134027 | 235.133968 | 3024127 | 14 | 20 | 0 | 3 | 0 | 0 |
| 237.076847 | 237.076847 | 2078508 | 12 | 14 | 0 | 5 | 0 | 0 |
| 237.092155 | 237.092103 | 1173582 | 16 | 14 | 0 | 2 | 0 | 0 |
| 237.113281 | 237.113233 | 2525904 | 13 | 18 | 0 | 4 | 0 | 0 |
| 239.092519 | 239.092497 | 3430123 | 12 | 16 | 0 | 5 | 0 | 0 |
| 239.107771 | 239.107753 | 2015629 | 16 | 16 | 0 | 2 | 0 | 0 |
| 239.128905 | 239.128883 | 1602192 | 13 | 20 | 0 | 4 | 0 | 0 |
| 239.143995 | 239.144139 | 1891761 | 17 | 20 | 0 | 1 | 0 | 0 |
| 241.087071 | 241.087018 | 2489875 | 15 | 14 | 0 | 3 | 0 | 0 |
| 241.123441 | 241.123403 | 3906839 | 16 | 18 | 0 | 2 | 0 | 0 |
| 241.159835 | 241.159789 | 3003739 | 17 | 22 | 0 | 1 | 0 | 0 |
| 243.066289 | 243.066282 | 2050946 | 14 | 12 | 0 | 4 | 0 | 0 |
| 243.102701 | 243.102668 | 4112198 | 15 | 16 | 0 | 3 | 0 | 0 |
| 243.139102 | 243.139053 | 5665931 | 16 | 20 | 0 | 2 | 0 | 0 |
| 243.175486 | 243.175439 | 2279887 | 17 | 24 | 0 | 1 | 0 | 0 |
| 245.045613 | 245.045547 | 1560825 | 13 | 10 | 0 | 5 | 0 | 0 |
| 245.081986 | 245.081932 | 4035645 | 14 | 14 | 0 | 4 | 0 | 0 |
| 245.118331 | 245.118318 | 8038914 | 15 | 18 | 0 | 3 | 0 | 0 |
| 245.154726 | 245.154703 | 6707719 | 16 | 22 | 0 | 2 | 0 | 0 |
| 245.19113 | 245.191089 | 1541515 | 17 | 26 | 0 | 1 | 0 | 0 |
| 247.061179 | 247.061197 | 1903900 | 13 | 12 | 0 | 5 | 0 | 0 |
| 247.097606 | 247.097583 | 5191233 | 14 | 16 | 0 | 4 | 0 | 0 |
| 247.133995 | 247.133968 | 6799110 | 15 | 20 | 0 | 3 | 0 | 0 |
| 247.170404 | 247.170354 | 2562315 | 16 | 24 | 0 | 2 | 0 | 0 |
| 249.040511 | 249.040462 | 1566978 | 12 | 10 | 0 | 6 | 0 | 0 |
| 249.076873 | 249.076847 | 3711495 | 13 | 14 | 0 | 5 | 0 | 0 |
| 249.113275 | 249.113233 | 7291660 | 14 | 18 | 0 | 4 | 0 | 0 |
| 249.149684 | 249.149618 | 5835537 | 15 | 22 | 0 | 3 | 0 | 0 |
| 251.056179 | 251.056112 | 1437395 | 12 | 12 | 0 | 6 | 0 | 0 |
| 251.09249 | 251.092497 | 3904920 | 13 | 16 | 0 | 5 | 0 | 0 |
| 251.10779 | 251.107753 | 1716618 | 17 | 16 | 0 | 2 | 0 | 0 |
| 251.128924 | 251.128883 | 5145501 | 14 | 20 | 0 | 4 | 0 | 0 |
| 251.144174 | 251.144139 | 2479391 | 18 | 20 | 0 | 1 | 0 | 0 |
| 253.050606 | 253.050632 | 1503590 | 15 | 10 | 0 | 4 | 0 | 0 |
| 253.071823 | 253.071762 | 2167977 | 12 | 14 | 0 | 6 | 0 | 0 |
| 253.087089 | 253.087018 | 3262380 | 16 | 14 | 0 | 3 | 0 | 0 |
| 253.108227 | 253.108147 | 4769071 | 13 | 18 | 0 | 5 | 0 | 0 |
| 253.123411 | 253.123403 | 6067185 | 17 | 18 | 0 | 2 | 0 | 0 |
| 253.144578 | 253.144533 | 2614932 | 14 | 22 | 0 | 4 | 0 | 0 |
| 253.159847 | 253.159789 | 4467190 | 18 | 22 | 0 | 1 | 0 | 0 |
| 255.066382 | 255.066282 | 2691202 | 15 | 12 | 0 | 4 | 0 | 0 |
| 255.087482 | 255.087412 | 1550532 | 12 | 16 | 0 | 6 | 0 | 0 |
| 255.102668 | 255.102668 | 7230663 | 16 | 16 | 0 | 3 | 0 | 0 |
| 255.123894 | 255.123797 | 1902026 | 13 | 20 | 0 | 5 | 0 | 0 |
| 255.139142 | 255.139053 | 9561420 | 17 | 20 | 0 | 2 | 0 | 0 |
| 255.175478 | 255.175439 | 4793297 | 18 | 24 | 0 | 1 | 0 | 0 |
| 256.097937 | 256.097917 | 1347188 | 15 | 15 | 1 | 3 | 0 | 0 |
| 257.045612 | 257.045547 | 2337371 | 14 | 10 | 0 | 5 | 0 | 0 |
| 257.081995 | 257.081932 | 5647073 | 15 | 14 | 0 | 4 | 0 | 0 |
| 257.118391 | 257.118318 | 13129830 | 16 | 18 | 0 | 3 | 0 | 0 |
| 257.154733 | 257.154703 | 13579883 | 17 | 22 | 0 | 2 | 0 | 0 |
| 257.191121 | 257.191089 | 3501936 | 18 | 26 | 0 | 1 | 0 | 0 |
| 259.024894 | 259.024812 | 1406552 | 13 | 8 | 0 | 6 | 0 | 0 |
| 259.06122 | 259.061197 | 3734525 | 14 | 12 | 0 | 5 | 0 | 0 |
| 259.097645 | 259.097583 | 9043843 | 15 | 16 | 0 | 4 | 0 | 0 |
| 259.133986 | 259.133968 | 15257736 | 16 | 20 | 0 | 3 | 0 | 0 |
| 259.170433 | 259.170354 | 9434253 | 17 | 24 | 0 | 2 | 0 | 0 |
| 259.206661 | 259.206739 | 1223730 | 18 | 28 | 0 | 1 | 0 | 0 |
| 261.040504 | 261.040462 | 2830301 | 13 | 10 | 0 | 6 | 0 | 0 |
| 261.076864 | 261.076847 | 5685922 | 14 | 14 | 0 | 5 | 0 | 0 |
| 261.113301 | 261.113233 | 14021543 | 15 | 18 | 0 | 4 | 0 | 0 |
| 261.12848 | 261.128489 | 1275369 | 19 | 18 | 0 | 1 | 0 | 0 |
| 261.149599 | 261.149618 | 13291948 | 16 | 22 | 0 | 3 | 0 | 0 |
| 261.186067 | 261.186004 | 4293874 | 17 | 26 | 0 | 2 | 0 | 0 |
| 263.056149 | 263.056112 | 2779074 | 13 | 12 | 0 | 6 | 0 | 0 |
| 263.071328 | 263.071368 | 1222885 | 17 | 12 | 0 | 3 | 0 | 0 |
| 263.092534 | 263.092497 | 7211464 | 14 | 16 | 0 | 5 | 0 | 0 |
| 263.107795 | 263.107753 | 2025450 | 18 | 16 | 0 | 2 | 0 | 0 |
| 263.128941 | 263.128883 | 11954637 | 15 | 20 | 0 | 4 | 0 | 0 |
| 263.144047 | 263.144139 | 2310607 | 19 | 20 | 0 | 1 | 0 | 0 |
| 263.16526 | 263.165268 | 6544082 | 16 | 24 | 0 | 3 | 0 | 0 |
| 263.201737 | 263.201654 | 1295192 | 17 | 28 | 0 | 2 | 0 | 0 |
| 265.035374 | 265.035376 | 1213572 | 12 | 10 | 0 | 7 | 0 | 0 |
| 265.050734 | 265.050632 | 1486822 | 16 | 10 | 0 | 4 | 0 | 0 |
| 265.071808 | 265.071762 | 3258089 | 13 | 14 | 0 | 6 | 0 | 0 |
| 265.087046 | 265.087018 | 3536811 | 17 | 14 | 0 | 3 | 0 | 0 |
| 265.10819 | 265.108147 | 11015662 | 14 | 18 | 0 | 5 | 0 | 0 |
| 265.123418 | 265.123403 | 6692465 | 18 | 18 | 0 | 2 | 0 | 0 |
| 265.144558 | 265.144533 | 11290868 | 15 | 22 | 0 | 4 | 0 | 0 |
| 265.159842 | 265.159789 | 5063286 | 19 | 22 | 0 | 1 | 0 | 0 |
| 265.180848 | 265.180918 | 2551737 | 16 | 26 | 0 | 3 | 0 | 0 |
| 267.029919 | 267.029897 | 1403143 | 15 | 8 | 0 | 5 | 0 | 0 |
| 267.051119 | 267.051026 | 1285066 | 12 | 12 | 0 | 7 | 0 | 0 |
| 267.066325 | 267.066282 | 3711885 | 16 | 12 | 0 | 4 | 0 | 0 |
| 267.087416 | 267.087412 | 4125200 | 13 | 16 | 0 | 6 | 0 | 0 |
| 267.102689 | 267.102668 | 7942418 | 17 | 16 | 0 | 3 | 0 | 0 |
| 267.123815 | 267.123797 | 7053589 | 14 | 20 | 0 | 5 | 0 | 0 |
| 267.139067 | 267.139053 | 12070423 | 18 | 20 | 0 | 2 | 0 | 0 |
| 267.175441 | 267.175439 | 5838877 | 19 | 24 | 0 | 1 | 0 | 0 |
| 269.045538 | 269.045547 | 3455277 | 15 | 10 | 0 | 5 | 0 | 0 |
| 269.082 | 269.081932 | 7477938 | 16 | 14 | 0 | 4 | 0 | 0 |
| 269.103059 | 269.103062 | 3408181 | 13 | 18 | 0 | 6 | 0 | 0 |
| 269.118373 | 269.118318 | 18202936 | 17 | 18 | 0 | 3 | 0 | 0 |
| 269.139526 | 269.139447 | 4103483 | 14 | 22 | 0 | 5 | 0 | 0 |
| 269.154747 | 269.154703 | 21915964 | 18 | 22 | 0 | 2 | 0 | 0 |
| 269.175875 | 269.175833 | 1754048 | 15 | 26 | 0 | 4 | 0 | 0 |
| 269.191125 | 269.191089 | 6774978 | 19 | 26 | 0 | 1 | 0 | 0 |
| 270.040798 | 270.040796 | 1211325 | 14 | 9 | 1 | 5 | 0 | 0 |
| 270.05344 | 270.053683 | 1538751 | 11 | 14 | 1 | 5 | 0 | 1 |
| 270.07725 | 270.077181 | 1796291 | 15 | 13 | 1 | 4 | 0 | 0 |
| 270.089852 | 270.090068 | 1922884 | 12 | 18 | 1 | 4 | 0 | 1 |
| 270.113656 | 270.113567 | 1615784 | 16 | 17 | 1 | 3 | 0 | 0 |
| 270.126224 | 270.126454 | 2155018 | 13 | 22 | 1 | 3 | 0 | 1 |
| 271.024857 | 271.024812 | 2631756 | 14 | 8 | 0 | 6 | 0 | 0 |
| 271.061252 | 271.061197 | 5621585 | 15 | 12 | 0 | 5 | 0 | 0 |
| 271.097604 | 271.097583 | 13454678 | 16 | 16 | 0 | 4 | 0 | 0 |
| 271.11882 | 271.118712 | 2006169 | 13 | 20 | 0 | 6 | 0 | 0 |
| 271.133992 | 271.133968 | 26139228 | 17 | 20 | 0 | 3 | 0 | 0 |
| 271.170387 | 271.170354 | 20047968 | 18 | 24 | 0 | 2 | 0 | 0 |
| 271.206757 | 271.206739 | 2848870 | 19 | 28 | 0 | 1 | 0 | 0 |
| 272.056494 | 272.056446 | 1396512 | 14 | 11 | 1 | 5 | 0 | 0 |
| 272.092944 | 272.092832 | 1497574 | 15 | 15 | 1 | 4 | 0 | 0 |
| 272.105491 | 272.105718 | 1655911 | 12 | 20 | 1 | 4 | 0 | 1 |
| 272.129167 | 272.129217 | 1696491 | 16 | 19 | 1 | 3 | 0 | 0 |
| 273.04047 | 273.040462 | 3742701 | 14 | 10 | 0 | 6 | 0 | 0 |
| 273.07692 | 273.076847 | 9006194 | 15 | 14 | 0 | 5 | 0 | 0 |
| 273.113252 | 273.113233 | 20916088 | 16 | 18 | 0 | 4 | 0 | 0 |
| 273.14964 | 273.149618 | 27959164 | 17 | 22 | 0 | 3 | 0 | 0 |
| 273.186022 | 273.186004 | 12048514 | 18 | 26 | 0 | 2 | 0 | 0 |
| 274.072133 | 274.072096 | 1448960 | 14 | 13 | 1 | 5 | 0 | 0 |
| 274.108533 | 274.108482 | 1551238 | 15 | 17 | 1 | 4 | 0 | 0 |
| 274.144921 | 274.144867 | 1668427 | 16 | 21 | 1 | 3 | 0 | 0 |
| 275.019749 | 275.019726 | 1736711 | 13 | 8 | 0 | 7 | 0 | 0 |
| 275.056122 | 275.056112 | 5180556 | 14 | 12 | 0 | 6 | 0 | 0 |
| 275.092508 | 275.092497 | 12411537 | 15 | 16 | 0 | 5 | 0 | 0 |
| 275.107777 | 275.107753 | 1848147 | 19 | 16 | 0 | 2 | 0 | 0 |
| 275.128916 | 275.128883 | 23463062 | 16 | 20 | 0 | 4 | 0 | 0 |
| 275.144134 | 275.144139 | 2262232 | 20 | 20 | 0 | 1 | 0 | 0 |
| 275.165348 | 275.165268 | 20212892 | 17 | 24 | 0 | 3 | 0 | 0 |
| 275.201639 | 275.201654 | 4517280 | 18 | 28 | 0 | 2 | 0 | 0 |
| 276.124234 | 276.124132 | 1251682 | 15 | 19 | 1 | 4 | 0 | 0 |
| 277.035315 | 277.035376 | 2381153 | 13 | 10 | 0 | 7 | 0 | 0 |
| 277.050443 | 277.050632 | 1220067 | 17 | 10 | 0 | 4 | 0 | 0 |
| 277.071776 | 277.071762 | 4898470 | 14 | 14 | 0 | 6 | 0 | 0 |
| 277.087041 | 277.087018 | 3005096 | 18 | 14 | 0 | 3 | 0 | 0 |
| 277.108151 | 277.108147 | 15511467 | 15 | 18 | 0 | 5 | 0 | 0 |
| 277.123434 | 277.123403 | 4938285 | 19 | 18 | 0 | 2 | 0 | 0 |
| 277.159795 | 277.159789 | 4559538 | 20 | 22 | 0 | 1 | 0 | 0 |
| 277.180967 | 277.180918 | 8439477 | 17 | 26 | 0 | 3 | 0 | 0 |
| 278.11602 | 278.116283 | 1426294 | 11 | 22 | 1 | 5 | 0 | 1 |
| 279.050989 | 279.051026 | 2105271 | 13 | 12 | 0 | 7 | 0 | 0 |
| 279.066349 | 279.066282 | 3517369 | 17 | 12 | 0 | 4 | 0 | 0 |
| 279.087417 | 279.087412 | 6008764 | 14 | 16 | 0 | 6 | 0 | 0 |
| 279.102675 | 279.102668 | 8142782 | 18 | 16 | 0 | 3 | 0 | 0 |
| 279.123778 | 279.123797 | 18642624 | 15 | 20 | 0 | 5 | 0 | 0 |
| 279.139083 | 279.139053 | 11958978 | 19 | 20 | 0 | 2 | 0 | 0 |
| 279.160191 | 279.160183 | 12153285 | 16 | 24 | 0 | 4 | 0 | 0 |
| 279.17547 | 279.175439 | 6664392 | 20 | 24 | 0 | 1 | 0 | 0 |
| 279.196543 | 279.196568 | 2050186 | 17 | 28 | 0 | 3 | 0 | 0 |
| 280.074212 | 280.074418 | 1710529 | 13 | 16 | 1 | 4 | 0 | 1 |
| 280.098038 | 280.097917 | 1379716 | 17 | 15 | 1 | 3 | 0 | 0 |
| 280.110553 | 280.110804 | 2183174 | 14 | 20 | 1 | 3 | 0 | 1 |
| 280.131657 | 280.131933 | 1471177 | 11 | 24 | 1 | 5 | 0 | 1 |
| 281.045566 | 281.045547 | 3608644 | 16 | 10 | 0 | 5 | 0 | 0 |
| 281.066694 | 281.066676 | 2069254 | 13 | 14 | 0 | 7 | 0 | 0 |
| 281.081996 | 281.081932 | 7894217 | 17 | 14 | 0 | 4 | 0 | 0 |
| 281.103062 | 281.103062 | 8000203 | 14 | 18 | 0 | 6 | 0 | 0 |
| 281.118377 | 281.118318 | 16594893 | 18 | 18 | 0 | 3 | 0 | 0 |
| 281.139462 | 281.139447 | 15435728 | 15 | 22 | 0 | 5 | 0 | 0 |
| 281.154735 | 281.154703 | 20907474 | 19 | 22 | 0 | 2 | 0 | 0 |
| 281.175891 | 281.175833 | 3792469 | 16 | 26 | 0 | 4 | 0 | 0 |
| 281.191077 | 281.191089 | 8241367 | 20 | 26 | 0 | 1 | 0 | 0 |
| 282.077081 | 282.077181 | 1291948 | 16 | 13 | 1 | 4 | 0 | 0 |
| 282.089868 | 282.090068 | 1807310 | 13 | 18 | 1 | 4 | 0 | 1 |
| 282.113538 | 282.113567 | 1873489 | 17 | 17 | 1 | 3 | 0 | 0 |
| 283.024846 | 283.024812 | 1986248 | 15 | 8 | 0 | 6 | 0 | 0 |
| 283.061202 | 283.061197 | 6546637 | 16 | 12 | 0 | 5 | 0 | 0 |
| 283.082366 | 283.082326 | 1584112 | 13 | 16 | 0 | 7 | 0 | 0 |
| 283.097599 | 283.097583 | 13773010 | 17 | 16 | 0 | 4 | 0 | 0 |
| 283.10885 | 283.108816 | 1724243 | 16 | 16 | 2 | 3 | 0 | 0 |
| 283.118701 | 283.118712 | 5893077 | 14 | 20 | 0 | 6 | 0 | 0 |
| 283.133998 | 283.133968 | 28327126 | 18 | 20 | 0 | 3 | 0 | 0 |
| 283.155038 | 283.155097 | 7056857 | 15 | 24 | 0 | 5 | 0 | 0 |
| 283.170391 | 283.170354 | 24936668 | 19 | 24 | 0 | 2 | 0 | 0 |
| 283.206732 | 283.206739 | 5389920 | 20 | 28 | 0 | 1 | 0 | 0 |
| 284.056417 | 284.056446 | 1724622 | 15 | 11 | 1 | 5 | 0 | 0 |
| 284.069051 | 284.069333 | 1930703 | 12 | 16 | 1 | 5 | 0 | 1 |
| 284.09287 | 284.092832 | 2308754 | 16 | 15 | 1 | 4 | 0 | 0 |
| 284.105484 | 284.105718 | 2211540 | 13 | 20 | 1 | 4 | 0 | 1 |
| 284.12929 | 284.129217 | 1639383 | 17 | 19 | 1 | 3 | 0 | 0 |
| 284.165617 | 284.165603 | 1296540 | 18 | 23 | 1 | 2 | 0 | 0 |
| 285.040432 | 285.040462 | 5396683 | 15 | 10 | 0 | 6 | 0 | 0 |
| 285.076843 | 285.076847 | 11494351 | 16 | 14 | 0 | 5 | 0 | 0 |
| 285.088058 | 285.08808 | 1945809 | 15 | 14 | 2 | 4 | 0 | 0 |
| 285.09791 | 285.097976 | 2013618 | 13 | 18 | 0 | 7 | 0 | 0 |
| 285.113259 | 285.113233 | 26680788 | 17 | 18 | 0 | 4 | 0 | 0 |
| 285.124523 | 285.124466 | 2178773 | 16 | 18 | 2 | 3 | 0 | 0 |
| 285.128491 | 285.128489 | 1258006 | 21 | 18 | 0 | 1 | 0 | 0 |
| 285.134356 | 285.134362 | 2919575 | 14 | 22 | 0 | 6 | 0 | 0 |
| 285.149622 | 285.149618 | 44560856 | 18 | 22 | 0 | 3 | 0 | 0 |
| 285.170665 | 285.170747 | 1822171 | 15 | 26 | 0 | 5 | 0 | 0 |
| 285.186004 | 285.186004 | 23627228 | 19 | 26 | 0 | 2 | 0 | 0 |
| 285.222397 | 285.222389 | 3138402 | 20 | 30 | 0 | 1 | 0 | 0 |
| 286.048406 | 286.048597 | 1275593 | 11 | 14 | 1 | 6 | 0 | 1 |
| 286.072076 | 286.072096 | 1850060 | 15 | 13 | 1 | 5 | 0 | 0 |
| 286.084709 | 286.084983 | 1996557 | 12 | 18 | 1 | 5 | 0 | 1 |
| 286.108521 | 286.108482 | 2646352 | 16 | 17 | 1 | 4 | 0 | 0 |
| 286.121132 | 286.121368 | 1769938 | 13 | 22 | 1 | 4 | 0 | 1 |
| 286.14479 | 286.144867 | 1941461 | 17 | 21 | 1 | 3 | 0 | 0 |
| 287.019729 | 287.019726 | 2475841 | 14 | 8 | 0 | 7 | 0 | 0 |
| 287.056112 | 287.056112 | 6658501 | 15 | 12 | 0 | 6 | 0 | 0 |
| 287.092521 | 287.092497 | 14291657 | 16 | 16 | 0 | 5 | 0 | 0 |
| 287.103778 | 287.103731 | 1897163 | 15 | 16 | 2 | 4 | 0 | 0 |
| 287.107644 | 287.107753 | 1626315 | 20 | 16 | 0 | 2 | 0 | 0 |
| 287.128906 | 287.128883 | 33263310 | 17 | 20 | 0 | 4 | 0 | 0 |
| 287.144187 | 287.144139 | 1576400 | 21 | 20 | 0 | 1 | 0 | 0 |
| 287.150009 | 287.150012 | 1343505 | 14 | 24 | 0 | 6 | 0 | 0 |
| 287.165284 | 287.165268 | 34983636 | 18 | 24 | 0 | 3 | 0 | 0 |
| 287.201674 | 287.201654 | 10661079 | 19 | 28 | 0 | 2 | 0 | 0 |
| 288.087668 | 288.087746 | 1539458 | 15 | 15 | 1 | 5 | 0 | 0 |
| 288.124079 | 288.124132 | 1944518 | 16 | 19 | 1 | 4 | 0 | 0 |
| 288.136812 | 288.137018 | 1373768 | 13 | 24 | 1 | 4 | 0 | 1 |
| 289.035388 | 289.035376 | 3522995 | 14 | 10 | 0 | 7 | 0 | 0 |
| 289.071779 | 289.071762 | 7564471 | 15 | 14 | 0 | 6 | 0 | 0 |
| 289.083067 | 289.082995 | 1687737 | 14 | 14 | 2 | 5 | 0 | 0 |
| 289.087033 | 289.087018 | 2626617 | 19 | 14 | 0 | 3 | 0 | 0 |
| 289.108146 | 289.108147 | 18462652 | 16 | 18 | 0 | 5 | 0 | 0 |
| 289.123391 | 289.123403 | 3470269 | 20 | 18 | 0 | 2 | 0 | 0 |
| 289.144531 | 289.144533 | 34337728 | 17 | 22 | 0 | 4 | 0 | 0 |
| 289.159812 | 289.159789 | 3154114 | 21 | 22 | 0 | 1 | 0 | 0 |
| 289.180906 | 289.180918 | 19334084 | 18 | 26 | 0 | 3 | 0 | 0 |
| 289.217284 | 289.217304 | 2851464 | 19 | 30 | 0 | 2 | 0 | 0 |
| 290.103283 | 290.103396 | 1409712 | 15 | 17 | 1 | 5 | 0 | 0 |
| 290.131348 | 290.131539 | 1269299 | 16 | 22 | 1 | 2 | 0 | 1 |
| 290.139865 | 290.139782 | 1380852 | 16 | 21 | 1 | 4 | 0 | 0 |
| 291.051075 | 291.051026 | 2983709 | 14 | 12 | 0 | 7 | 0 | 0 |
| 291.066362 | 291.066282 | 2873503 | 18 | 12 | 0 | 4 | 0 | 0 |
| 291.0874 | 291.087412 | 8990113 | 15 | 16 | 0 | 6 | 0 | 0 |
| 291.102718 | 291.102668 | 5990819 | 19 | 16 | 0 | 3 | 0 | 0 |
| 291.123808 | 291.123797 | 23957670 | 16 | 20 | 0 | 5 | 0 | 0 |
| 291.139085 | 291.139053 | 9073319 | 20 | 20 | 0 | 2 | 0 | 0 |
| 291.160173 | 291.160183 | 28015786 | 17 | 24 | 0 | 4 | 0 | 0 |
| 291.175444 | 291.175439 | 6536364 | 21 | 24 | 0 | 1 | 0 | 0 |
| 291.196558 | 291.196568 | 8365486 | 18 | 28 | 0 | 3 | 0 | 0 |
| 292.097988 | 292.097917 | 1330356 | 18 | 15 | 1 | 3 | 0 | 0 |
| 293.045636 | 293.045547 | 2814782 | 17 | 10 | 0 | 5 | 0 | 0 |
| 293.066638 | 293.066676 | 2443776 | 14 | 14 | 0 | 7 | 0 | 0 |
| 293.081961 | 293.081932 | 6367618 | 18 | 14 | 0 | 4 | 0 | 0 |
| 293.103031 | 293.103062 | 10653828 | 15 | 18 | 0 | 6 | 0 | 0 |
| 293.11835 | 293.118318 | 12474246 | 19 | 18 | 0 | 3 | 0 | 0 |
| 293.139432 | 293.139447 | 20661640 | 16 | 22 | 0 | 5 | 0 | 0 |
| 293.154718 | 293.154703 | 16038794 | 20 | 22 | 0 | 2 | 0 | 0 |
| 293.191105 | 293.191089 | 7611022 | 21 | 26 | 0 | 1 | 0 | 0 |
| 294.077253 | 294.077181 | 1349103 | 17 | 13 | 1 | 4 | 0 | 0 |
| 294.089796 | 294.090068 | 2224113 | 14 | 18 | 1 | 4 | 0 | 1 |
| 294.113572 | 294.113567 | 1243443 | 18 | 17 | 1 | 3 | 0 | 0 |
| 294.14995 | 294.149952 | 1264183 | 19 | 21 | 1 | 2 | 0 | 0 |
| 295.024869 | 295.024812 | 1916486 | 16 | 8 | 0 | 6 | 0 | 0 |
| 295.061225 | 295.061197 | 6199386 | 17 | 12 | 0 | 5 | 0 | 0 |
| 295.082357 | 295.082326 | 2257180 | 14 | 16 | 0 | 7 | 0 | 0 |
| 295.097599 | 295.097583 | 12631646 | 18 | 16 | 0 | 4 | 0 | 0 |
| 295.118716 | 295.118712 | 12331616 | 15 | 20 | 0 | 6 | 0 | 0 |
| 295.133977 | 295.133968 | 23650914 | 19 | 20 | 0 | 3 | 0 | 0 |
| 295.155106 | 295.155097 | 11148388 | 16 | 24 | 0 | 5 | 0 | 0 |
| 295.17035 | 295.170354 | 23167590 | 20 | 24 | 0 | 2 | 0 | 0 |
| 295.191487 | 295.191483 | 3058792 | 17 | 28 | 0 | 4 | 0 | 0 |
| 295.206755 | 295.206739 | 7118697 | 21 | 28 | 0 | 1 | 0 | 0 |
| 296.056524 | 296.056446 | 1950276 | 16 | 11 | 1 | 5 | 0 | 0 |
| 296.092841 | 296.092832 | 2233544 | 17 | 15 | 1 | 4 | 0 | 0 |
| 296.10547 | 296.105718 | 2465481 | 14 | 20 | 1 | 4 | 0 | 1 |
| 296.129255 | 296.129217 | 2051083 | 18 | 19 | 1 | 3 | 0 | 0 |
| 296.141925 | 296.142104 | 2177549 | 15 | 24 | 1 | 3 | 0 | 1 |
| 297.04048 | 297.040462 | 5015467 | 16 | 10 | 0 | 6 | 0 | 0 |
| 297.076823 | 297.076847 | 11504942 | 17 | 14 | 0 | 5 | 0 | 0 |
| 297.08812 | 297.08808 | 2019952 | 16 | 14 | 2 | 4 | 0 | 0 |
| 297.097973 | 297.097976 | 3312561 | 14 | 18 | 0 | 7 | 0 | 0 |
| 297.113211 | 297.113233 | 25220914 | 18 | 18 | 0 | 4 | 0 | 0 |
| 297.124514 | 297.124466 | 1648723 | 17 | 18 | 2 | 3 | 0 | 0 |
| 297.134407 | 297.134362 | 11369012 | 15 | 22 | 0 | 6 | 0 | 0 |
| 297.149596 | 297.149618 | 42886968 | 19 | 22 | 0 | 3 | 0 | 0 |
| 297.170781 | 297.170747 | 5120824 | 16 | 26 | 0 | 5 | 0 | 0 |
| 297.185978 | 297.186004 | 27113274 | 20 | 26 | 0 | 2 | 0 | 0 |
| 297.222384 | 297.222389 | 5324862 | 21 | 30 | 0 | 1 | 0 | 0 |
| 298.072102 | 298.072096 | 1838293 | 16 | 13 | 1 | 5 | 0 | 0 |
| 298.108482 | 298.108482 | 2381721 | 17 | 17 | 1 | 4 | 0 | 0 |
| 298.144881 | 298.144867 | 2336413 | 18 | 21 | 1 | 3 | 0 | 0 |
| 298.15746 | 298.157754 | 1967518 | 15 | 26 | 1 | 3 | 0 | 1 |
| 299.019691 | 299.019726 | 2934646 | 15 | 8 | 0 | 7 | 0 | 0 |
| 299.056113 | 299.056112 | 8463865 | 16 | 12 | 0 | 6 | 0 | 0 |
| 299.09249 | 299.092497 | 15857661 | 17 | 16 | 0 | 5 | 0 | 0 |
| 299.095938 | 299.095868 | 1375677 | 14 | 20 | 0 | 5 | 1 | 0 |
| 299.103739 | 299.103731 | 2271230 | 16 | 16 | 2 | 4 | 0 | 0 |
| 299.113739 | 299.113627 | 2149247 | 14 | 20 | 0 | 7 | 0 | 0 |
| 299.128883 | 299.128883 | 36972544 | 18 | 20 | 0 | 4 | 0 | 0 |
| 299.132263 | 299.132253 | 1353537 | 15 | 24 | 0 | 4 | 1 | 0 |
| 299.1401 | 299.140116 | 1539074 | 17 | 20 | 2 | 3 | 0 | 0 |
| 299.14997 | 299.150012 | 4254467 | 15 | 24 | 0 | 6 | 0 | 0 |
| 299.165285 | 299.165268 | 44957700 | 19 | 24 | 0 | 3 | 0 | 0 |
| 299.20167 | 299.201654 | 16470536 | 20 | 28 | 0 | 2 | 0 | 0 |
| 299.238035 | 299.238039 | 2016651 | 21 | 32 | 0 | 1 | 0 | 0 |
| 300.064008 | 300.064247 | 1280734 | 12 | 16 | 1 | 6 | 0 | 1 |
| 300.087712 | 300.087746 | 1730272 | 16 | 15 | 1 | 5 | 0 | 0 |
| 300.124111 | 300.124132 | 2545252 | 17 | 19 | 1 | 4 | 0 | 0 |
| 300.160539 | 300.160517 | 2194536 | 18 | 23 | 1 | 3 | 0 | 0 |
| 301.035436 | 301.035376 | 4705726 | 15 | 10 | 0 | 7 | 0 | 0 |
| 301.07178 | 301.071762 | 8448193 | 16 | 14 | 0 | 6 | 0 | 0 |
| 301.082901 | 301.082995 | 1244098 | 15 | 14 | 2 | 5 | 0 | 0 |
| 301.087064 | 301.087018 | 1755843 | 20 | 14 | 0 | 3 | 0 | 0 |
| 301.108176 | 301.108147 | 20590788 | 17 | 18 | 0 | 5 | 0 | 0 |
| 301.119458 | 301.119381 | 2113606 | 16 | 18 | 2 | 4 | 0 | 0 |
| 301.123417 | 301.123403 | 2315206 | 21 | 18 | 0 | 2 | 0 | 0 |
| 301.14455 | 301.144533 | 41941192 | 18 | 22 | 0 | 4 | 0 | 0 |
| 301.147748 | 301.147904 | 1260105 | 15 | 26 | 0 | 4 | 1 | 0 |
| 301.159796 | 301.159789 | 1790986 | 22 | 22 | 0 | 1 | 0 | 0 |
| 301.180918 | 301.180918 | 29896908 | 19 | 26 | 0 | 3 | 0 | 0 |
| 301.217277 | 301.217304 | 6859984 | 20 | 30 | 0 | 2 | 0 | 0 |
| 302.103404 | 302.103396 | 1643685 | 16 | 17 | 1 | 5 | 0 | 0 |
| 302.139798 | 302.139782 | 2177833 | 17 | 21 | 1 | 4 | 0 | 0 |
| 302.152369 | 302.152668 | 1262378 | 14 | 26 | 1 | 4 | 0 | 1 |
| 302.176092 | 302.176167 | 1370796 | 18 | 25 | 1 | 3 | 0 | 0 |
| 303.051031 | 303.051026 | 4890240 | 15 | 12 | 0 | 7 | 0 | 0 |
| 303.066185 | 303.066282 | 1672065 | 19 | 12 | 0 | 4 | 0 | 0 |
| 303.087406 | 303.087412 | 9765251 | 16 | 16 | 0 | 6 | 0 | 0 |
| 303.098839 | 303.098645 | 1466628 | 15 | 16 | 2 | 5 | 0 | 0 |
| 303.10268 | 303.102668 | 3986821 | 20 | 16 | 0 | 3 | 0 | 0 |
| 303.123768 | 303.123797 | 27509126 | 17 | 20 | 0 | 5 | 0 | 0 |
| 303.139112 | 303.139053 | 4734345 | 21 | 20 | 0 | 2 | 0 | 0 |
| 303.160171 | 303.160183 | 39065996 | 18 | 24 | 0 | 4 | 0 | 0 |
| 303.175514 | 303.175439 | 3605388 | 22 | 24 | 0 | 1 | 0 | 0 |
| 303.196545 | 303.196568 | 16544142 | 19 | 28 | 0 | 3 | 0 | 0 |
| 304.110622 | 304.110804 | 1301700 | 16 | 20 | 1 | 3 | 0 | 1 |
| 304.119071 | 304.119046 | 1717989 | 16 | 19 | 1 | 5 | 0 | 0 |
| 304.155471 | 304.155432 | 1337064 | 17 | 23 | 1 | 4 | 0 | 0 |
| 305.030248 | 305.030291 | 2061689 | 14 | 10 | 0 | 8 | 0 | 0 |
| 305.045495 | 305.045547 | 2153243 | 18 | 10 | 0 | 5 | 0 | 0 |
| 305.066635 | 305.066676 | 5720125 | 15 | 14 | 0 | 7 | 0 | 0 |
| 305.081927 | 305.081932 | 5157438 | 19 | 14 | 0 | 4 | 0 | 0 |
| 305.103034 | 305.103062 | 13447744 | 16 | 18 | 0 | 6 | 0 | 0 |
| 305.118302 | 305.118318 | 9177153 | 20 | 18 | 0 | 3 | 0 | 0 |
| 305.139448 | 305.139447 | 30713412 | 17 | 22 | 0 | 5 | 0 | 0 |
| 305.154698 | 305.154703 | 11823173 | 21 | 22 | 0 | 2 | 0 | 0 |
| 305.175861 | 305.175833 | 25926214 | 18 | 26 | 0 | 4 | 0 | 0 |
| 305.191068 | 305.191089 | 6416968 | 22 | 26 | 0 | 1 | 0 | 0 |
| 306.053434 | 306.053683 | 1398935 | 14 | 14 | 1 | 5 | 0 | 1 |
| 306.089799 | 306.090068 | 1734682 | 15 | 18 | 1 | 4 | 0 | 1 |
| 306.11358 | 306.113567 | 1627548 | 19 | 17 | 1 | 3 | 0 | 0 |
| 307.02482 | 307.024812 | 1596975 | 17 | 8 | 0 | 6 | 0 | 0 |
| 307.045952 | 307.045941 | 1407345 | 14 | 12 | 0 | 8 | 0 | 0 |
| 307.061206 | 307.061197 | 4703730 | 18 | 12 | 0 | 5 | 0 | 0 |
| 307.08231 | 307.082326 | 4436980 | 15 | 16 | 0 | 7 | 0 | 0 |
| 307.097584 | 307.097583 | 10450165 | 19 | 16 | 0 | 4 | 0 | 0 |
| 307.1187 | 307.118712 | 15424247 | 16 | 20 | 0 | 6 | 0 | 0 |
| 307.133957 | 307.133968 | 18487032 | 20 | 20 | 0 | 3 | 0 | 0 |
| 307.155112 | 307.155097 | 23592698 | 17 | 24 | 0 | 5 | 0 | 0 |
| 307.170334 | 307.170354 | 20120316 | 21 | 24 | 0 | 2 | 0 | 0 |
| 307.206752 | 307.206739 | 7273215 | 22 | 28 | 0 | 1 | 0 | 0 |
| 308.069153 | 308.069333 | 1735692 | 14 | 16 | 1 | 5 | 0 | 1 |
| 308.092956 | 308.092832 | 1822030 | 18 | 15 | 1 | 4 | 0 | 0 |
| 308.105439 | 308.105718 | 2197967 | 15 | 20 | 1 | 4 | 0 | 1 |
| 308.129162 | 308.129217 | 1415569 | 19 | 19 | 1 | 3 | 0 | 0 |
| 308.141874 | 308.142104 | 2636242 | 16 | 24 | 1 | 3 | 0 | 1 |
| 309.040478 | 309.040462 | 4500897 | 17 | 10 | 0 | 6 | 0 | 0 |
| 309.076853 | 309.076847 | 8771492 | 18 | 14 | 0 | 5 | 0 | 0 |
| 309.088145 | 309.08808 | 1557413 | 17 | 14 | 2 | 4 | 0 | 0 |
| 309.097971 | 309.097976 | 5432999 | 15 | 18 | 0 | 7 | 0 | 0 |
| 309.113216 | 309.113233 | 17883048 | 19 | 18 | 0 | 4 | 0 | 0 |
| 309.124671 | 309.124466 | 1387305 | 18 | 18 | 2 | 3 | 0 | 0 |
| 309.134367 | 309.134362 | 14564265 | 16 | 22 | 0 | 6 | 0 | 0 |
| 309.149606 | 309.149618 | 30098346 | 20 | 22 | 0 | 3 | 0 | 0 |
| 309.186014 | 309.186004 | 21809070 | 21 | 26 | 0 | 2 | 0 | 0 |
| 309.222403 | 309.222389 | 5818033 | 22 | 30 | 0 | 1 | 0 | 0 |
| 310.072109 | 310.072096 | 1779771 | 17 | 13 | 1 | 5 | 0 | 0 |
| 310.108454 | 310.108482 | 1879486 | 18 | 17 | 1 | 4 | 0 | 0 |
| 310.144971 | 310.144867 | 1732609 | 19 | 21 | 1 | 3 | 0 | 0 |
| 311.019737 | 311.019726 | 2088652 | 16 | 8 | 0 | 7 | 0 | 0 |
| 311.056081 | 311.056112 | 7160911 | 17 | 12 | 0 | 6 | 0 | 0 |
| 311.092466 | 311.092497 | 14963794 | 18 | 16 | 0 | 5 | 0 | 0 |
| 311.095865 | 311.095868 | 1304211 | 15 | 20 | 0 | 5 | 1 | 0 |
| 311.103822 | 311.103731 | 2434515 | 17 | 16 | 2 | 4 | 0 | 0 |
| 311.113645 | 311.113627 | 6656084 | 15 | 20 | 0 | 7 | 0 | 0 |
| 311.128853 | 311.128883 | 32052310 | 19 | 20 | 0 | 4 | 0 | 0 |
| 311.140074 | 311.140116 | 1263318 | 18 | 20 | 2 | 3 | 0 | 0 |
| 311.150048 | 311.150012 | 9722455 | 16 | 24 | 0 | 6 | 0 | 0 |
| 311.165282 | 311.165268 | 40852568 | 20 | 24 | 0 | 3 | 0 | 0 |
| 311.186408 | 311.186398 | 3851098 | 17 | 28 | 0 | 5 | 0 | 0 |
| 311.201702 | 311.201654 | 20200540 | 21 | 28 | 0 | 2 | 0 | 0 |
| 311.238014 | 311.238039 | 3042271 | 22 | 32 | 0 | 1 | 0 | 0 |
| 312.051326 | 312.051361 | 1402403 | 16 | 11 | 1 | 6 | 0 | 0 |
| 312.087783 | 312.087746 | 2139687 | 17 | 15 | 1 | 5 | 0 | 0 |
| 312.100367 | 312.100633 | 2431912 | 14 | 20 | 1 | 5 | 0 | 1 |
| 312.124102 | 312.124132 | 2461354 | 18 | 19 | 1 | 4 | 0 | 0 |
| 312.136743 | 312.137018 | 2653867 | 15 | 24 | 1 | 4 | 0 | 1 |
| 312.160474 | 312.160517 | 2370477 | 19 | 23 | 1 | 3 | 0 | 0 |
| 313.035453 | 313.035376 | 4910326 | 16 | 10 | 0 | 7 | 0 | 0 |
| 313.071763 | 313.071762 | 10124537 | 17 | 14 | 0 | 6 | 0 | 0 |
| 313.083078 | 313.082995 | 1734650 | 16 | 14 | 2 | 5 | 0 | 0 |
| 313.108119 | 313.108147 | 23007484 | 18 | 18 | 0 | 5 | 0 | 0 |
| 313.111455 | 313.111518 | 2875900 | 15 | 22 | 0 | 5 | 1 | 0 |
| 313.119326 | 313.119381 | 2215677 | 17 | 18 | 2 | 4 | 0 | 0 |
| 313.123579 | 313.123403 | 1397245 | 22 | 18 | 0 | 2 | 0 | 0 |
| 313.129278 | 313.129277 | 4555646 | 15 | 22 | 0 | 7 | 0 | 0 |
| 313.144511 | 313.144533 | 46238976 | 19 | 22 | 0 | 4 | 0 | 0 |
| 313.159802 | 313.159789 | 1280896 | 23 | 22 | 0 | 1 | 0 | 0 |
| 313.165681 | 313.165662 | 3398785 | 16 | 26 | 0 | 6 | 0 | 0 |
| 313.180911 | 313.180918 | 41139456 | 20 | 26 | 0 | 3 | 0 | 0 |
| 313.217326 | 313.217304 | 11900165 | 21 | 30 | 0 | 2 | 0 | 0 |
| 314.066976 | 314.067011 | 1718411 | 16 | 13 | 1 | 6 | 0 | 0 |
| 314.103361 | 314.103396 | 1872270 | 17 | 17 | 1 | 5 | 0 | 0 |
| 314.116082 | 314.116283 | 1844431 | 14 | 22 | 1 | 5 | 0 | 1 |
| 314.139782 | 314.139782 | 2923601 | 18 | 21 | 1 | 4 | 0 | 0 |
| 314.152463 | 314.152668 | 1643218 | 15 | 26 | 1 | 4 | 0 | 1 |
| 314.176165 | 314.176167 | 2047188 | 19 | 25 | 1 | 3 | 0 | 0 |
| 315.014549 | 315.014641 | 1842456 | 15 | 8 | 0 | 8 | 0 | 0 |
| 315.051014 | 315.051026 | 5954971 | 16 | 12 | 0 | 7 | 0 | 0 |
| 315.066248 | 315.066282 | 1777436 | 20 | 12 | 0 | 4 | 0 | 0 |
| 315.087429 | 315.087412 | 12463518 | 17 | 16 | 0 | 6 | 0 | 0 |
| 315.090863 | 315.090783 | 1516062 | 14 | 20 | 0 | 6 | 1 | 0 |
| 315.098729 | 315.098645 | 1599903 | 16 | 16 | 2 | 5 | 0 | 0 |
| 315.102616 | 315.102668 | 2530975 | 21 | 16 | 0 | 3 | 0 | 0 |
| 315.123807 | 315.123797 | 27542946 | 18 | 20 | 0 | 5 | 0 | 0 |
| 315.127177 | 315.127168 | 4365986 | 15 | 24 | 0 | 5 | 1 | 0 |
| 315.13508 | 315.135031 | 1358178 | 17 | 20 | 2 | 4 | 0 | 0 |
| 315.139062 | 315.139053 | 3444642 | 22 | 20 | 0 | 2 | 0 | 0 |
| 315.144894 | 315.144927 | 1800227 | 15 | 24 | 0 | 7 | 0 | 0 |
| 315.160178 | 315.160183 | 43965860 | 19 | 24 | 0 | 4 | 0 | 0 |
| 315.175459 | 315.175439 | 2539301 | 23 | 24 | 0 | 1 | 0 | 0 |
| 315.196551 | 315.196568 | 24124838 | 20 | 28 | 0 | 3 | 0 | 0 |
| 315.232984 | 315.232954 | 3970218 | 21 | 32 | 0 | 2 | 0 | 0 |
| 316.119047 | 316.119046 | 1989489 | 17 | 19 | 1 | 5 | 0 | 0 |
| 316.155526 | 316.155432 | 2316148 | 18 | 23 | 1 | 4 | 0 | 0 |
| 317.030206 | 317.030291 | 2580026 | 15 | 10 | 0 | 8 | 0 | 0 |
| 317.066663 | 317.066676 | 6891581 | 16 | 14 | 0 | 7 | 0 | 0 |
| 317.081963 | 317.081932 | 3184702 | 20 | 14 | 0 | 4 | 0 | 0 |
| 317.103044 | 317.103062 | 13506624 | 17 | 18 | 0 | 6 | 0 | 0 |
| 317.114442 | 317.114295 | 1607233 | 16 | 18 | 2 | 5 | 0 | 0 |
| 317.118319 | 317.118318 | 4779841 | 21 | 18 | 0 | 3 | 0 | 0 |
| 317.13945 | 317.139447 | 31991362 | 18 | 22 | 0 | 5 | 0 | 0 |
| 317.142812 | 317.142818 | 3303235 | 15 | 26 | 0 | 5 | 1 | 0 |
| 317.154666 | 317.154703 | 6490692 | 22 | 22 | 0 | 2 | 0 | 0 |
| 317.175858 | 317.175833 | 32243270 | 19 | 26 | 0 | 4 | 0 | 0 |
| 317.191054 | 317.191089 | 3302983 | 23 | 26 | 0 | 1 | 0 | 0 |
| 318.098216 | 318.098311 | 1251743 | 16 | 17 | 1 | 6 | 0 | 0 |
| 318.1262 | 318.126454 | 1850385 | 17 | 22 | 1 | 3 | 0 | 1 |
| 318.134652 | 318.134696 | 1683985 | 17 | 21 | 1 | 5 | 0 | 0 |
| 319.045906 | 319.045941 | 2843865 | 15 | 12 | 0 | 8 | 0 | 0 |
| 319.061194 | 319.061197 | 3970778 | 19 | 12 | 0 | 5 | 0 | 0 |
| 319.082318 | 319.082326 | 5976028 | 16 | 16 | 0 | 7 | 0 | 0 |
| 319.097577 | 319.097583 | 6799069 | 20 | 16 | 0 | 4 | 0 | 0 |
| 319.118749 | 319.118712 | 16402142 | 17 | 20 | 0 | 6 | 0 | 0 |
| 319.13396 | 319.133968 | 12231392 | 21 | 20 | 0 | 3 | 0 | 0 |
| 319.155112 | 319.155097 | 32048866 | 18 | 24 | 0 | 5 | 0 | 0 |
| 319.170363 | 319.170354 | 12873442 | 22 | 24 | 0 | 2 | 0 | 0 |
| 319.191449 | 319.191483 | 20119268 | 19 | 28 | 0 | 4 | 0 | 0 |
| 319.206712 | 319.206739 | 5228773 | 23 | 28 | 0 | 1 | 0 | 0 |
| 320.077572 | 320.077575 | 1299369 | 15 | 15 | 1 | 7 | 0 | 0 |
| 320.092825 | 320.092832 | 1626922 | 19 | 15 | 1 | 4 | 0 | 0 |
| 320.129183 | 320.129217 | 1553965 | 20 | 19 | 1 | 3 | 0 | 0 |
| 321.040477 | 321.040462 | 3181043 | 18 | 10 | 0 | 6 | 0 | 0 |
| 321.06159 | 321.061591 | 1950836 | 15 | 14 | 0 | 8 | 0 | 0 |
| 321.076827 | 321.076847 | 7440246 | 19 | 14 | 0 | 5 | 0 | 0 |
| 321.088298 | 321.08808 | 1336566 | 18 | 14 | 2 | 4 | 0 | 0 |
| 321.097993 | 321.097976 | 5866103 | 16 | 18 | 0 | 7 | 0 | 0 |
| 321.113249 | 321.113233 | 13692280 | 20 | 18 | 0 | 4 | 0 | 0 |
| 321.12457 | 321.124466 | 1505081 | 19 | 18 | 2 | 3 | 0 | 0 |
| 321.134364 | 321.134362 | 21436282 | 17 | 22 | 0 | 6 | 0 | 0 |
| 321.149656 | 321.149618 | 22743930 | 21 | 22 | 0 | 3 | 0 | 0 |
| 321.170728 | 321.170747 | 22572924 | 18 | 26 | 0 | 5 | 0 | 0 |
| 321.186009 | 321.186004 | 19972990 | 22 | 26 | 0 | 2 | 0 | 0 |
| 321.222355 | 321.222389 | 6380929 | 23 | 30 | 0 | 1 | 0 | 0 |
| 322.048487 | 322.048597 | 1403199 | 14 | 14 | 1 | 6 | 0 | 1 |
| 322.072319 | 322.072096 | 1577025 | 18 | 13 | 1 | 5 | 0 | 0 |
| 322.084677 | 322.084983 | 2156546 | 15 | 18 | 1 | 5 | 0 | 1 |
| 322.108634 | 322.108482 | 1815108 | 19 | 17 | 1 | 4 | 0 | 0 |
| 322.121099 | 322.121368 | 2764741 | 16 | 22 | 1 | 4 | 0 | 1 |
| 322.144794 | 322.144867 | 1705031 | 20 | 21 | 1 | 3 | 0 | 0 |
| 322.181341 | 322.181253 | 1337801 | 21 | 25 | 1 | 2 | 0 | 0 |
| 323.019787 | 323.019726 | 1415113 | 17 | 8 | 0 | 7 | 0 | 0 |
| 323.056096 | 323.056112 | 7037964 | 18 | 12 | 0 | 6 | 0 | 0 |
| 323.077195 | 323.077241 | 1887245 | 15 | 16 | 0 | 8 | 0 | 0 |
| 323.092486 | 323.092497 | 11472910 | 19 | 16 | 0 | 5 | 0 | 0 |
| 323.103753 | 323.103731 | 1985807 | 18 | 16 | 2 | 4 | 0 | 0 |
| 323.113627 | 323.113627 | 7389712 | 16 | 20 | 0 | 7 | 0 | 0 |
| 323.128871 | 323.128883 | 24121360 | 20 | 20 | 0 | 4 | 0 | 0 |
| 323.140214 | 323.140116 | 1624466 | 19 | 20 | 2 | 3 | 0 | 0 |
| 323.149976 | 323.150012 | 19836946 | 17 | 24 | 0 | 6 | 0 | 0 |
| 323.165287 | 323.165268 | 33939476 | 21 | 24 | 0 | 3 | 0 | 0 |
| 323.186374 | 323.186398 | 11415061 | 18 | 28 | 0 | 5 | 0 | 0 |
| 323.201652 | 323.201654 | 20968470 | 22 | 28 | 0 | 2 | 0 | 0 |
| 323.23801 | 323.238039 | 4672537 | 23 | 32 | 0 | 1 | 0 | 0 |
| 324.087715 | 324.087746 | 1932377 | 18 | 15 | 1 | 5 | 0 | 0 |
| 324.124151 | 324.124132 | 2870619 | 19 | 19 | 1 | 4 | 0 | 0 |
| 324.16055 | 324.160517 | 1940958 | 20 | 23 | 1 | 3 | 0 | 0 |
| 325.035307 | 325.035376 | 3977375 | 17 | 10 | 0 | 7 | 0 | 0 |
| 325.071765 | 325.071762 | 9186466 | 18 | 14 | 0 | 6 | 0 | 0 |
| 325.083045 | 325.082995 | 1402531 | 17 | 14 | 2 | 5 | 0 | 0 |
| 325.092871 | 325.092891 | 1746979 | 15 | 18 | 0 | 8 | 0 | 0 |
| 325.108169 | 325.108147 | 17136804 | 19 | 18 | 0 | 5 | 0 | 0 |
| 325.119339 | 325.119381 | 2229029 | 18 | 18 | 2 | 4 | 0 | 0 |
| 325.129297 | 325.129277 | 8258726 | 16 | 22 | 0 | 7 | 0 | 0 |
| 325.144512 | 325.144533 | 33691816 | 20 | 22 | 0 | 4 | 0 | 0 |
| 325.155681 | 325.155766 | 1291688 | 19 | 22 | 2 | 3 | 0 | 0 |
| 325.16568 | 325.165662 | 8975529 | 17 | 26 | 0 | 6 | 0 | 0 |
| 325.180914 | 325.180918 | 36767912 | 21 | 26 | 0 | 3 | 0 | 0 |
| 325.217311 | 325.217304 | 16837804 | 22 | 30 | 0 | 2 | 0 | 0 |
| 325.253668 | 325.253689 | 2034351 | 23 | 34 | 0 | 1 | 0 | 0 |
| 326.067042 | 326.067011 | 1583595 | 17 | 13 | 1 | 6 | 0 | 0 |
| 326.103442 | 326.103396 | 1847406 | 18 | 17 | 1 | 5 | 0 | 0 |
| 326.13967 | 326.139782 | 2466545 | 19 | 21 | 1 | 4 | 0 | 0 |
| 326.176078 | 326.176167 | 1709043 | 20 | 25 | 1 | 3 | 0 | 0 |
| 327.014623 | 327.014641 | 1759025 | 16 | 8 | 0 | 8 | 0 | 0 |
| 327.051037 | 327.051026 | 5920052 | 17 | 12 | 0 | 7 | 0 | 0 |
| 327.087402 | 327.087412 | 11252534 | 18 | 16 | 0 | 6 | 0 | 0 |
| 327.090713 | 327.090783 | 2102838 | 15 | 20 | 0 | 6 | 1 | 0 |
| 327.098696 | 327.098645 | 1783991 | 17 | 16 | 2 | 5 | 0 | 0 |
| 327.102736 | 327.102668 | 1319031 | 22 | 16 | 0 | 3 | 0 | 0 |
| 327.108574 | 327.108541 | 2279224 | 15 | 20 | 0 | 8 | 0 | 0 |
| 327.123782 | 327.123797 | 26139960 | 19 | 20 | 0 | 5 | 0 | 0 |
| 327.135 | 327.135031 | 1853626 | 18 | 20 | 2 | 4 | 0 | 0 |
| 327.139132 | 327.139053 | 1704826 | 23 | 20 | 0 | 2 | 0 | 0 |
| 327.144932 | 327.144927 | 4100666 | 16 | 24 | 0 | 7 | 0 | 0 |
| 327.160193 | 327.160183 | 41290044 | 20 | 24 | 0 | 4 | 0 | 0 |
| 327.196576 | 327.196568 | 29526334 | 21 | 28 | 0 | 3 | 0 | 0 |
| 327.232954 | 327.232954 | 7874881 | 22 | 32 | 0 | 2 | 0 | 0 |
| 328.082573 | 328.082661 | 1494975 | 17 | 15 | 1 | 6 | 0 | 0 |
| 328.118987 | 328.119046 | 2133121 | 18 | 19 | 1 | 5 | 0 | 0 |
| 328.122312 | 328.122417 | 1621250 | 15 | 23 | 1 | 5 | 1 | 0 |
| 328.155399 | 328.155432 | 1959556 | 19 | 23 | 1 | 4 | 0 | 0 |
| 328.191819 | 328.191817 | 1291079 | 20 | 27 | 1 | 3 | 0 | 0 |
| 329.030241 | 329.030291 | 3116996 | 16 | 10 | 0 | 8 | 0 | 0 |
| 329.066673 | 329.066676 | 6749894 | 17 | 14 | 0 | 7 | 0 | 0 |
| 329.070035 | 329.070047 | 1345094 | 14 | 18 | 0 | 7 | 1 | 0 |
| 329.082018 | 329.081932 | 3005639 | 21 | 14 | 0 | 4 | 0 | 0 |
| 329.103044 | 329.103062 | 15521225 | 18 | 18 | 0 | 6 | 0 | 0 |
| 329.10641 | 329.106433 | 4336073 | 15 | 22 | 0 | 6 | 1 | 0 |
| 329.114314 | 329.114295 | 1883210 | 17 | 18 | 2 | 5 | 0 | 0 |
| 329.118313 | 329.118318 | 3339210 | 22 | 18 | 0 | 3 | 0 | 0 |
| 329.124103 | 329.124191 | 1439178 | 15 | 22 | 0 | 8 | 0 | 0 |
| 329.139465 | 329.139447 | 32950732 | 19 | 22 | 0 | 5 | 0 | 0 |
| 329.154671 | 329.154703 | 4334797 | 23 | 22 | 0 | 2 | 0 | 0 |
| 329.175824 | 329.175833 | 39129548 | 20 | 26 | 0 | 4 | 0 | 0 |
| 329.191087 | 329.191089 | 2339279 | 24 | 26 | 0 | 1 | 0 | 0 |
| 329.212196 | 329.212218 | 16595409 | 21 | 30 | 0 | 3 | 0 | 0 |
| 329.248722 | 329.248604 | 2450771 | 22 | 34 | 0 | 2 | 0 | 0 |
| 330.098263 | 330.098311 | 1445329 | 17 | 17 | 1 | 6 | 0 | 0 |
| 330.126188 | 330.126454 | 1274899 | 18 | 22 | 1 | 3 | 0 | 1 |
| 330.134799 | 330.134696 | 2134931 | 18 | 21 | 1 | 5 | 0 | 0 |
| 330.147291 | 330.147583 | 1558676 | 15 | 26 | 1 | 5 | 0 | 1 |
| 330.171025 | 330.171082 | 1774998 | 19 | 25 | 1 | 4 | 0 | 0 |
| 331.045983 | 331.045941 | 3566165 | 16 | 12 | 0 | 8 | 0 | 0 |
| 331.06115 | 331.061197 | 2738262 | 20 | 12 | 0 | 5 | 0 | 0 |
| 331.082328 | 331.082326 | 8548951 | 17 | 16 | 0 | 7 | 0 | 0 |
| 331.085706 | 331.085697 | 1292631 | 14 | 20 | 0 | 7 | 1 | 0 |
| 331.09759 | 331.097583 | 4775512 | 21 | 16 | 0 | 4 | 0 | 0 |
| 331.118679 | 331.118712 | 20417114 | 18 | 20 | 0 | 6 | 0 | 0 |
| 331.122047 | 331.122083 | 6500954 | 15 | 24 | 0 | 6 | 1 | 0 |
| 331.133943 | 331.133968 | 7265883 | 22 | 20 | 0 | 3 | 0 | 0 |
| 331.155108 | 331.155097 | 38898268 | 19 | 24 | 0 | 5 | 0 | 0 |
| 331.170322 | 331.170354 | 8163934 | 23 | 24 | 0 | 2 | 0 | 0 |
| 331.191503 | 331.191483 | 29215326 | 20 | 28 | 0 | 4 | 0 | 0 |
| 331.206759 | 331.206739 | 3776224 | 24 | 28 | 0 | 1 | 0 | 0 |
| 331.227837 | 331.227868 | 6214242 | 21 | 32 | 0 | 3 | 0 | 0 |
| 332.092815 | 332.092832 | 1421856 | 20 | 15 | 1 | 4 | 0 | 0 |
| 332.105413 | 332.105718 | 1757088 | 17 | 20 | 1 | 4 | 0 | 1 |
| 332.113966 | 332.113961 | 1794081 | 17 | 19 | 1 | 6 | 0 | 0 |
| 332.129171 | 332.129217 | 1488546 | 21 | 19 | 1 | 3 | 0 | 0 |
| 332.141876 | 332.142104 | 1551651 | 18 | 24 | 1 | 3 | 0 | 1 |
| 332.150468 | 332.150346 | 1597220 | 18 | 23 | 1 | 5 | 0 | 0 |
| 333.040633 | 333.040462 | 2304099 | 19 | 10 | 0 | 6 | 0 | 0 |
| 333.061565 | 333.061591 | 4373989 | 16 | 14 | 0 | 8 | 0 | 0 |
| 333.076782 | 333.076847 | 5792998 | 20 | 14 | 0 | 5 | 0 | 0 |
| 333.097965 | 333.097976 | 7631591 | 17 | 18 | 0 | 7 | 0 | 0 |
| 333.101282 | 333.101347 | 1406183 | 14 | 22 | 0 | 7 | 1 | 0 |
| 333.113177 | 333.113233 | 9276136 | 21 | 18 | 0 | 4 | 0 | 0 |
| 333.124524 | 333.124466 | 1281641 | 20 | 18 | 2 | 3 | 0 | 0 |
| 333.134378 | 333.134362 | 23005930 | 18 | 22 | 0 | 6 | 0 | 0 |
| 333.137702 | 333.137733 | 3493354 | 15 | 26 | 0 | 6 | 1 | 0 |
| 333.149617 | 333.149618 | 14130923 | 22 | 22 | 0 | 3 | 0 | 0 |
| 333.170729 | 333.170747 | 32582380 | 19 | 26 | 0 | 5 | 0 | 0 |
| 333.186003 | 333.186004 | 12410605 | 23 | 26 | 0 | 2 | 0 | 0 |
| 333.222319 | 333.222389 | 4783856 | 24 | 30 | 0 | 1 | 0 | 0 |
| 333.243686 | 333.243519 | 1316114 | 21 | 34 | 0 | 3 | 0 | 0 |
| 334.072232 | 334.072096 | 1523117 | 19 | 13 | 1 | 5 | 0 | 0 |
| 334.084714 | 334.084983 | 1705581 | 16 | 18 | 1 | 5 | 0 | 1 |
| 334.108386 | 334.108482 | 1468207 | 20 | 17 | 1 | 4 | 0 | 0 |
| 334.144803 | 334.144867 | 1375666 | 21 | 21 | 1 | 3 | 0 | 0 |
| 334.157446 | 334.157754 | 1465523 | 18 | 26 | 1 | 3 | 0 | 1 |
| 335.019696 | 335.019726 | 1528048 | 18 | 8 | 0 | 7 | 0 | 0 |
| 335.056142 | 335.056112 | 4755827 | 19 | 12 | 0 | 6 | 0 | 0 |
| 335.077247 | 335.077241 | 3114356 | 16 | 16 | 0 | 8 | 0 | 0 |
| 335.092477 | 335.092497 | 8649077 | 20 | 16 | 0 | 5 | 0 | 0 |
| 335.103691 | 335.103731 | 1886582 | 19 | 16 | 2 | 4 | 0 | 0 |
| 335.113623 | 335.113627 | 8768886 | 17 | 20 | 0 | 7 | 0 | 0 |
| 335.12888 | 335.128883 | 16296823 | 21 | 20 | 0 | 4 | 0 | 0 |
| 335.140238 | 335.140116 | 1329528 | 20 | 20 | 2 | 3 | 0 | 0 |
| 335.149971 | 335.150012 | 24683384 | 18 | 24 | 0 | 6 | 0 | 0 |
| 335.165282 | 335.165268 | 22610810 | 22 | 24 | 0 | 3 | 0 | 0 |
| 335.186384 | 335.186398 | 19556220 | 19 | 28 | 0 | 5 | 0 | 0 |
| 335.201624 | 335.201654 | 16519037 | 23 | 28 | 0 | 2 | 0 | 0 |
| 335.23806 | 335.238039 | 4575103 | 24 | 32 | 0 | 1 | 0 | 0 |
| 336.087794 | 336.087746 | 1723963 | 19 | 15 | 1 | 5 | 0 | 0 |
| 336.100326 | 336.100633 | 1700284 | 16 | 20 | 1 | 5 | 0 | 1 |
| 336.12412 | 336.124132 | 2075582 | 20 | 19 | 1 | 4 | 0 | 0 |
| 336.136692 | 336.137018 | 2210303 | 17 | 24 | 1 | 4 | 0 | 1 |
| 336.160502 | 336.160517 | 1486145 | 21 | 23 | 1 | 3 | 0 | 0 |
| 336.173211 | 336.173404 | 2017474 | 18 | 28 | 1 | 3 | 0 | 1 |
| 337.035326 | 337.035376 | 3686143 | 18 | 10 | 0 | 7 | 0 | 0 |
| 337.071768 | 337.071762 | 7824897 | 19 | 14 | 0 | 6 | 0 | 0 |
| 337.092815 | 337.092891 | 3138563 | 16 | 18 | 0 | 8 | 0 | 0 |
| 337.108127 | 337.108147 | 12961796 | 20 | 18 | 0 | 5 | 0 | 0 |
| 337.119556 | 337.119381 | 1579524 | 19 | 18 | 2 | 4 | 0 | 0 |
| 337.12931 | 337.129277 | 11819013 | 17 | 22 | 0 | 7 | 0 | 0 |
| 337.144521 | 337.144533 | 26668038 | 21 | 22 | 0 | 4 | 0 | 0 |
| 337.165641 | 337.165662 | 17367048 | 18 | 26 | 0 | 6 | 0 | 0 |
| 337.180931 | 337.180918 | 30735368 | 22 | 26 | 0 | 3 | 0 | 0 |
| 337.217272 | 337.217304 | 17565708 | 23 | 30 | 0 | 2 | 0 | 0 |
| 337.253668 | 337.253689 | 2457102 | 24 | 34 | 0 | 1 | 0 | 0 |
| 338.066948 | 338.067011 | 1379912 | 18 | 13 | 1 | 6 | 0 | 0 |
| 338.079628 | 338.079897 | 1620680 | 15 | 18 | 1 | 6 | 0 | 1 |
| 338.103423 | 338.103396 | 2342218 | 19 | 17 | 1 | 5 | 0 | 0 |
| 338.116003 | 338.116283 | 1355211 | 16 | 22 | 1 | 5 | 0 | 1 |
| 338.139795 | 338.139782 | 2276301 | 20 | 21 | 1 | 4 | 0 | 0 |
| 338.152344 | 338.152668 | 2086094 | 17 | 26 | 1 | 4 | 0 | 1 |
| 338.176166 | 338.176167 | 2062927 | 21 | 25 | 1 | 3 | 0 | 0 |
| 339.014735 | 339.014641 | 1863691 | 17 | 8 | 0 | 8 | 0 | 0 |
| 339.05102 | 339.051026 | 5733517 | 18 | 12 | 0 | 7 | 0 | 0 |
| 339.087388 | 339.087412 | 11179152 | 19 | 16 | 0 | 6 | 0 | 0 |
| 339.098534 | 339.098645 | 1932432 | 18 | 16 | 2 | 5 | 0 | 0 |
| 339.108447 | 339.108541 | 3032209 | 16 | 20 | 0 | 8 | 0 | 0 |
| 339.123821 | 339.123797 | 20129938 | 20 | 20 | 0 | 5 | 0 | 0 |
| 339.135056 | 339.135031 | 1550483 | 19 | 20 | 2 | 4 | 0 | 0 |
| 339.144893 | 339.144927 | 10885268 | 17 | 24 | 0 | 7 | 0 | 0 |
| 339.16016 | 339.160183 | 37600404 | 21 | 24 | 0 | 4 | 0 | 0 |
| 339.181304 | 339.181312 | 8480406 | 18 | 28 | 0 | 6 | 0 | 0 |
| 339.196538 | 339.196568 | 32343192 | 22 | 28 | 0 | 3 | 0 | 0 |
| 339.232978 | 339.232954 | 12425370 | 23 | 32 | 0 | 2 | 0 | 0 |
| 339.26936 | 339.269339 | 1698205 | 24 | 36 | 0 | 1 | 0 | 0 |
| 340.058848 | 340.059162 | 1381204 | 14 | 16 | 1 | 7 | 0 | 1 |
| 340.082611 | 340.082661 | 1525974 | 18 | 15 | 1 | 6 | 0 | 0 |
| 340.118998 | 340.119046 | 2334553 | 19 | 19 | 1 | 5 | 0 | 0 |
| 340.155445 | 340.155432 | 2637275 | 20 | 23 | 1 | 4 | 0 | 0 |
| 340.168025 | 340.168318 | 1595868 | 17 | 28 | 1 | 4 | 0 | 1 |
| 340.191818 | 340.191817 | 1450014 | 21 | 27 | 1 | 3 | 0 | 0 |
| 341.030378 | 341.030291 | 3234713 | 17 | 10 | 0 | 8 | 0 | 0 |
| 341.066651 | 341.066676 | 7143708 | 18 | 14 | 0 | 7 | 0 | 0 |
| 341.070084 | 341.070047 | 1643548 | 15 | 18 | 0 | 7 | 1 | 0 |
| 341.081924 | 341.081932 | 1521949 | 22 | 14 | 0 | 4 | 0 | 0 |
| 341.103047 | 341.103062 | 15110430 | 19 | 18 | 0 | 6 | 0 | 0 |
| 341.106464 | 341.106433 | 1518591 | 16 | 22 | 0 | 6 | 1 | 0 |
| 341.114398 | 341.114295 | 1484575 | 18 | 18 | 2 | 5 | 0 | 0 |
| 341.118344 | 341.118318 | 1912736 | 23 | 18 | 0 | 3 | 0 | 0 |
| 341.124127 | 341.124191 | 2592544 | 16 | 22 | 0 | 8 | 0 | 0 |
| 341.139439 | 341.139447 | 29717792 | 20 | 22 | 0 | 5 | 0 | 0 |
| 341.154775 | 341.154703 | 2133666 | 24 | 22 | 0 | 2 | 0 | 0 |
| 341.160561 | 341.160577 | 4350755 | 17 | 26 | 0 | 7 | 0 | 0 |
| 341.17581 | 341.175833 | 39740708 | 21 | 26 | 0 | 4 | 0 | 0 |
| 341.190925 | 341.191089 | 1605925 | 25 | 26 | 0 | 1 | 0 | 0 |
| 341.196944 | 341.196962 | 1833381 | 18 | 30 | 0 | 6 | 0 | 0 |
| 341.21223 | 341.212218 | 23835942 | 22 | 30 | 0 | 3 | 0 | 0 |
| 341.248545 | 341.248604 | 5962281 | 23 | 34 | 0 | 2 | 0 | 0 |
| 342.098353 | 342.098311 | 1639077 | 18 | 17 | 1 | 6 | 0 | 0 |
| 342.134674 | 342.134696 | 2219368 | 19 | 21 | 1 | 5 | 0 | 0 |
| 342.147306 | 342.147583 | 1567593 | 16 | 26 | 1 | 5 | 0 | 1 |
| 342.170961 | 342.171082 | 2317162 | 20 | 25 | 1 | 4 | 0 | 0 |
| 343.045949 | 343.045941 | 3381225 | 17 | 12 | 0 | 8 | 0 | 0 |
| 343.061139 | 343.061197 | 1713066 | 21 | 12 | 0 | 5 | 0 | 0 |
| 343.082329 | 343.082326 | 8013739 | 18 | 16 | 0 | 7 | 0 | 0 |
| 343.085706 | 343.085697 | 2079659 | 15 | 20 | 0 | 7 | 1 | 0 |
| 343.097563 | 343.097583 | 3241900 | 22 | 16 | 0 | 4 | 0 | 0 |
| 343.118682 | 343.118712 | 19733934 | 19 | 20 | 0 | 6 | 0 | 0 |
| 343.121957 | 343.122083 | 1532334 | 16 | 24 | 0 | 6 | 1 | 0 |
| 343.133928 | 343.133968 | 4444591 | 23 | 20 | 0 | 3 | 0 | 0 |
| 343.139808 | 343.139841 | 1756335 | 16 | 24 | 0 | 8 | 0 | 0 |
| 343.155124 | 343.155097 | 35313072 | 20 | 24 | 0 | 5 | 0 | 0 |
| 343.170328 | 343.170354 | 5194674 | 24 | 24 | 0 | 2 | 0 | 0 |
| 343.176138 | 343.176227 | 1380274 | 17 | 28 | 0 | 7 | 0 | 0 |
| 343.191462 | 343.191483 | 31714738 | 21 | 28 | 0 | 4 | 0 | 0 |
| 343.206669 | 343.206739 | 2493876 | 25 | 28 | 0 | 1 | 0 | 0 |
| 343.227839 | 343.227868 | 11311541 | 22 | 32 | 0 | 3 | 0 | 0 |
| 343.248935 | 343.248998 | 2216631 | 19 | 36 | 0 | 5 | 0 | 0 |
| 343.264214 | 343.264254 | 1767864 | 23 | 36 | 0 | 2 | 0 | 0 |
| 344.11405 | 344.113961 | 1657333 | 18 | 19 | 1 | 6 | 0 | 0 |
| 344.117315 | 344.117332 | 1335413 | 15 | 23 | 1 | 6 | 1 | 0 |
| 344.150322 | 344.150346 | 2422775 | 19 | 23 | 1 | 5 | 0 | 0 |
| 344.186659 | 344.186732 | 1426426 | 20 | 27 | 1 | 4 | 0 | 0 |
| 345.025023 | 345.025205 | 1335862 | 16 | 10 | 0 | 9 | 0 | 0 |
| 345.040426 | 345.040462 | 1674679 | 20 | 10 | 0 | 6 | 0 | 0 |
| 345.061587 | 345.061591 | 4145977 | 17 | 14 | 0 | 8 | 0 | 0 |
| 345.076879 | 345.076847 | 3893818 | 21 | 14 | 0 | 5 | 0 | 0 |
| 345.097946 | 345.097976 | 9502267 | 18 | 18 | 0 | 7 | 0 | 0 |
| 345.101278 | 345.101347 | 4156219 | 15 | 22 | 0 | 7 | 1 | 0 |
| 345.11327 | 345.113233 | 5978684 | 22 | 18 | 0 | 4 | 0 | 0 |
| 345.13437 | 345.134362 | 24866366 | 19 | 22 | 0 | 6 | 0 | 0 |
| 345.149637 | 345.149618 | 8613951 | 23 | 22 | 0 | 3 | 0 | 0 |
| 345.170743 | 345.170747 | 34072128 | 20 | 26 | 0 | 5 | 0 | 0 |
| 345.185997 | 345.186004 | 7717954 | 24 | 26 | 0 | 2 | 0 | 0 |
| 345.207127 | 345.207133 | 19246658 | 21 | 30 | 0 | 4 | 0 | 0 |
| 345.222407 | 345.222389 | 2758212 | 25 | 30 | 0 | 1 | 0 | 0 |
| 345.243555 | 345.243519 | 3918150 | 22 | 34 | 0 | 3 | 0 | 0 |
| 346.084732 | 346.084983 | 1721090 | 17 | 18 | 1 | 5 | 0 | 1 |
| 346.108569 | 346.108482 | 1672708 | 21 | 17 | 1 | 4 | 0 | 0 |
| 346.166136 | 346.165996 | 1723400 | 19 | 25 | 1 | 5 | 0 | 0 |
| 347.019783 | 347.019726 | 1341382 | 19 | 8 | 0 | 7 | 0 | 0 |
| 347.040812 | 347.040856 | 1627847 | 16 | 12 | 0 | 9 | 0 | 0 |
| 347.056057 | 347.056112 | 3981512 | 20 | 12 | 0 | 6 | 0 | 0 |
| 347.077275 | 347.077241 | 3835082 | 17 | 16 | 0 | 8 | 0 | 0 |
| 347.092549 | 347.092497 | 6269131 | 21 | 16 | 0 | 5 | 0 | 0 |
| 347.113602 | 347.113627 | 11316940 | 18 | 20 | 0 | 7 | 0 | 0 |
| 347.116973 | 347.116997 | 3839437 | 15 | 24 | 0 | 7 | 1 | 0 |
| 347.128843 | 347.128883 | 10229453 | 22 | 20 | 0 | 4 | 0 | 0 |
| 347.150025 | 347.150012 | 26056398 | 19 | 24 | 0 | 6 | 0 | 0 |
| 347.165265 | 347.165268 | 14699216 | 23 | 24 | 0 | 3 | 0 | 0 |
| 347.186369 | 347.186398 | 26195666 | 20 | 28 | 0 | 5 | 0 | 0 |
| 347.201669 | 347.201654 | 11859667 | 24 | 28 | 0 | 2 | 0 | 0 |
| 347.222801 | 347.222783 | 8240340 | 21 | 32 | 0 | 4 | 0 | 0 |
| 347.238015 | 347.238039 | 3359445 | 25 | 32 | 0 | 1 | 0 | 0 |
| 348.063931 | 348.064247 | 1548689 | 16 | 16 | 1 | 6 | 0 | 1 |
| 348.087819 | 348.087746 | 1564563 | 20 | 15 | 1 | 5 | 0 | 0 |
| 348.100302 | 348.100633 | 1577556 | 17 | 20 | 1 | 5 | 0 | 1 |
| 348.124244 | 348.124132 | 1682454 | 21 | 19 | 1 | 4 | 0 | 0 |
| 348.160453 | 348.160517 | 1529752 | 22 | 23 | 1 | 3 | 0 | 0 |
| 349.035372 | 349.035376 | 2897496 | 19 | 10 | 0 | 7 | 0 | 0 |
| 349.056441 | 349.056506 | 1547738 | 16 | 14 | 0 | 9 | 0 | 0 |
| 349.071774 | 349.071762 | 6569819 | 20 | 14 | 0 | 6 | 0 | 0 |
| 349.092824 | 349.092891 | 3619932 | 17 | 18 | 0 | 8 | 0 | 0 |
| 349.108101 | 349.108147 | 9586525 | 21 | 18 | 0 | 5 | 0 | 0 |
| 349.129313 | 349.129277 | 12921695 | 18 | 22 | 0 | 7 | 0 | 0 |
| 349.132722 | 349.132647 | 1783519 | 15 | 26 | 0 | 7 | 1 | 0 |
| 349.144549 | 349.144533 | 17206112 | 22 | 22 | 0 | 4 | 0 | 0 |
| 349.165617 | 349.165662 | 24235874 | 19 | 26 | 0 | 6 | 0 | 0 |
| 349.180905 | 349.180918 | 21600098 | 23 | 26 | 0 | 3 | 0 | 0 |
| 349.217287 | 349.217304 | 13520741 | 24 | 30 | 0 | 2 | 0 | 0 |
| 349.238497 | 349.238433 | 2024039 | 21 | 34 | 0 | 4 | 0 | 0 |
| 349.253657 | 349.253689 | 3052648 | 25 | 34 | 0 | 1 | 0 | 0 |
| 350.067032 | 350.067011 | 1646244 | 19 | 13 | 1 | 6 | 0 | 0 |
| 350.079702 | 350.079897 | 1795237 | 16 | 18 | 1 | 6 | 0 | 1 |
| 350.103522 | 350.103396 | 1814438 | 20 | 17 | 1 | 5 | 0 | 0 |
| 350.139809 | 350.139782 | 1770601 | 21 | 21 | 1 | 4 | 0 | 0 |
| 350.152325 | 350.152668 | 2162858 | 18 | 26 | 1 | 4 | 0 | 1 |
| 350.176119 | 350.176167 | 1587884 | 22 | 25 | 1 | 3 | 0 | 0 |
| 351.01466 | 351.014641 | 1930218 | 18 | 8 | 0 | 8 | 0 | 0 |
| 351.05106 | 351.051026 | 5564909 | 19 | 12 | 0 | 7 | 0 | 0 |
| 351.072157 | 351.072156 | 1340398 | 16 | 16 | 0 | 9 | 0 | 0 |
| 351.087445 | 351.087412 | 9052655 | 20 | 16 | 0 | 6 | 0 | 0 |
| 351.098625 | 351.098645 | 1471088 | 19 | 16 | 2 | 5 | 0 | 0 |
| 351.108596 | 351.108541 | 3563377 | 17 | 20 | 0 | 8 | 0 | 0 |
| 351.123773 | 351.123797 | 15822834 | 21 | 20 | 0 | 5 | 0 | 0 |
| 351.135039 | 351.135031 | 1610355 | 20 | 20 | 2 | 4 | 0 | 0 |
| 351.144936 | 351.144927 | 14987251 | 18 | 24 | 0 | 7 | 0 | 0 |
| 351.16022 | 351.160183 | 27178996 | 22 | 24 | 0 | 4 | 0 | 0 |
| 351.181292 | 351.181312 | 15967222 | 19 | 28 | 0 | 6 | 0 | 0 |
| 351.196568 | 351.196568 | 27142136 | 23 | 28 | 0 | 3 | 0 | 0 |
| 351.232962 | 351.232954 | 11984890 | 24 | 32 | 0 | 2 | 0 | 0 |
| 351.269402 | 351.269339 | 1814397 | 25 | 36 | 0 | 1 | 0 | 0 |
| 352.059086 | 352.059162 | 1436983 | 15 | 16 | 1 | 7 | 0 | 1 |
| 352.082601 | 352.082661 | 1673913 | 19 | 15 | 1 | 6 | 0 | 0 |
| 352.095233 | 352.095547 | 1711418 | 16 | 20 | 1 | 6 | 0 | 1 |
| 352.119061 | 352.119046 | 1832252 | 20 | 19 | 1 | 5 | 0 | 0 |
| 352.131608 | 352.131933 | 2170429 | 17 | 24 | 1 | 5 | 0 | 1 |
| 352.155336 | 352.155432 | 2088510 | 21 | 23 | 1 | 4 | 0 | 0 |
| 352.191748 | 352.191817 | 1446209 | 22 | 27 | 1 | 3 | 0 | 0 |
| 353.030333 | 353.030291 | 3289472 | 18 | 10 | 0 | 8 | 0 | 0 |
| 353.066672 | 353.066676 | 7318147 | 19 | 14 | 0 | 7 | 0 | 0 |
| 353.103032 | 353.103062 | 11786373 | 20 | 18 | 0 | 6 | 0 | 0 |
| 353.114264 | 353.114295 | 1976966 | 19 | 18 | 2 | 5 | 0 | 0 |
| 353.124137 | 353.124191 | 5579399 | 17 | 22 | 0 | 8 | 0 | 0 |
| 353.13946 | 353.139447 | 23488648 | 21 | 22 | 0 | 5 | 0 | 0 |
| 353.154735 | 353.154703 | 1536649 | 25 | 22 | 0 | 2 | 0 | 0 |
| 353.160568 | 353.160577 | 11654282 | 18 | 26 | 0 | 7 | 0 | 0 |
| 353.175805 | 353.175833 | 33716364 | 22 | 26 | 0 | 4 | 0 | 0 |
| 353.191241 | 353.191089 | 1320972 | 26 | 26 | 0 | 1 | 0 | 0 |
| 353.212248 | 353.212218 | 26630286 | 23 | 30 | 0 | 3 | 0 | 0 |
| 353.248592 | 353.248604 | 7195281 | 24 | 34 | 0 | 2 | 0 | 0 |
| 354.062134 | 354.061925 | 1393358 | 18 | 13 | 1 | 7 | 0 | 0 |
| 354.098536 | 354.098311 | 1446993 | 19 | 17 | 1 | 6 | 0 | 0 |
| 354.134631 | 354.134696 | 2577107 | 20 | 21 | 1 | 5 | 0 | 0 |
| 354.14758 | 354.147583 | 1374036 | 17 | 26 | 1 | 5 | 0 | 1 |
| 354.171113 | 354.171082 | 1878614 | 21 | 25 | 1 | 4 | 0 | 0 |
| 354.18367 | 354.183969 | 1407959 | 18 | 30 | 1 | 4 | 0 | 1 |
| 355.045952 | 355.045941 | 4506905 | 18 | 12 | 0 | 8 | 0 | 0 |
| 355.08228 | 355.082326 | 8133404 | 19 | 16 | 0 | 7 | 0 | 0 |
| 355.085647 | 355.085697 | 1421724 | 16 | 20 | 0 | 7 | 1 | 0 |
| 355.097582 | 355.097583 | 2172957 | 23 | 16 | 0 | 4 | 0 | 0 |
| 355.118725 | 355.118712 | 16290078 | 20 | 20 | 0 | 6 | 0 | 0 |
| 355.122138 | 355.122083 | 1434111 | 17 | 24 | 0 | 6 | 1 | 0 |
| 355.130042 | 355.129945 | 1541023 | 19 | 20 | 2 | 5 | 0 | 0 |
| 355.134055 | 355.133968 | 2167584 | 24 | 20 | 0 | 3 | 0 | 0 |
| 355.139817 | 355.139841 | 4036128 | 17 | 24 | 0 | 8 | 0 | 0 |
| 355.155108 | 355.155097 | 30372130 | 21 | 24 | 0 | 5 | 0 | 0 |
| 355.17039 | 355.170354 | 2350882 | 25 | 24 | 0 | 2 | 0 | 0 |
| 355.176192 | 355.176227 | 4765475 | 18 | 28 | 0 | 7 | 0 | 0 |
| 355.19147 | 355.191483 | 33517860 | 22 | 28 | 0 | 4 | 0 | 0 |
| 355.206687 | 355.206739 | 1617189 | 26 | 28 | 0 | 1 | 0 | 0 |
| 355.227874 | 355.227868 | 17332518 | 23 | 32 | 0 | 3 | 0 | 0 |
| 355.264207 | 355.264254 | 2986026 | 24 | 36 | 0 | 2 | 0 | 0 |
| 356.114102 | 356.113961 | 1496235 | 19 | 19 | 1 | 6 | 0 | 0 |
| 356.150351 | 356.150346 | 2160750 | 20 | 23 | 1 | 5 | 0 | 0 |
| 356.163074 | 356.163233 | 1432174 | 17 | 28 | 1 | 5 | 0 | 1 |
| 356.186878 | 356.186732 | 1503088 | 21 | 27 | 1 | 4 | 0 | 0 |
| 357.025337 | 357.025205 | 1740337 | 17 | 10 | 0 | 9 | 0 | 0 |
| 357.040539 | 357.040462 | 1289778 | 21 | 10 | 0 | 6 | 0 | 0 |
| 357.061532 | 357.061591 | 4398260 | 18 | 14 | 0 | 8 | 0 | 0 |
| 357.076735 | 357.076847 | 2223541 | 22 | 14 | 0 | 5 | 0 | 0 |
| 357.097989 | 357.097976 | 9159607 | 19 | 18 | 0 | 7 | 0 | 0 |
| 357.10142 | 357.101347 | 1940407 | 16 | 22 | 0 | 7 | 1 | 0 |
| 357.113228 | 357.113233 | 3735992 | 23 | 18 | 0 | 4 | 0 | 0 |
| 357.134311 | 357.134362 | 20949434 | 20 | 22 | 0 | 6 | 0 | 0 |
| 357.137773 | 357.137733 | 1583994 | 17 | 26 | 0 | 6 | 1 | 0 |
| 357.149589 | 357.149618 | 5161915 | 24 | 22 | 0 | 3 | 0 | 0 |
| 357.170762 | 357.170747 | 33581500 | 21 | 26 | 0 | 5 | 0 | 0 |
| 357.185932 | 357.186004 | 4493758 | 25 | 26 | 0 | 2 | 0 | 0 |
| 357.207105 | 357.207133 | 25366976 | 22 | 30 | 0 | 4 | 0 | 0 |
| 357.222365 | 357.222389 | 2276289 | 26 | 30 | 0 | 1 | 0 | 0 |
| 357.243538 | 357.243519 | 7642050 | 23 | 34 | 0 | 3 | 0 | 0 |
| 358.129608 | 358.129611 | 1365575 | 19 | 21 | 1 | 6 | 0 | 0 |
| 358.165945 | 358.165996 | 2095626 | 20 | 25 | 1 | 5 | 0 | 0 |
| 358.202403 | 358.202382 | 1479053 | 21 | 29 | 1 | 4 | 0 | 0 |
| 359.040767 | 359.040856 | 1706575 | 17 | 12 | 0 | 9 | 0 | 0 |
| 359.056093 | 359.056112 | 2780496 | 21 | 12 | 0 | 6 | 0 | 0 |
| 359.07725 | 359.077241 | 4791378 | 18 | 16 | 0 | 8 | 0 | 0 |
| 359.080538 | 359.080612 | 1533522 | 15 | 20 | 0 | 8 | 1 | 0 |
| 359.092554 | 359.092497 | 3976019 | 22 | 16 | 0 | 5 | 0 | 0 |
| 359.113613 | 359.113627 | 11395669 | 19 | 20 | 0 | 7 | 0 | 0 |
| 359.116944 | 359.116997 | 2155861 | 16 | 24 | 0 | 7 | 1 | 0 |
| 359.128886 | 359.128883 | 6939222 | 23 | 20 | 0 | 4 | 0 | 0 |
| 359.149997 | 359.150012 | 26126936 | 20 | 24 | 0 | 6 | 0 | 0 |
| 359.165233 | 359.165268 | 8452185 | 24 | 24 | 0 | 3 | 0 | 0 |
| 359.186394 | 359.186398 | 28469850 | 21 | 28 | 0 | 5 | 0 | 0 |
| 359.201671 | 359.201654 | 7344732 | 25 | 28 | 0 | 2 | 0 | 0 |
| 359.222769 | 359.222783 | 12642909 | 22 | 32 | 0 | 4 | 0 | 0 |
| 359.237987 | 359.238039 | 2900062 | 26 | 32 | 0 | 1 | 0 | 0 |
| 359.259241 | 359.259169 | 2434912 | 23 | 36 | 0 | 3 | 0 | 0 |
| 360.124174 | 360.124132 | 1590437 | 22 | 19 | 1 | 4 | 0 | 0 |
| 360.136697 | 360.137018 | 1593638 | 19 | 24 | 1 | 4 | 0 | 1 |
| 360.181639 | 360.181647 | 1318697 | 20 | 27 | 1 | 5 | 0 | 0 |
| 361.03543 | 361.035376 | 2136301 | 20 | 10 | 0 | 7 | 0 | 0 |
| 361.056475 | 361.056506 | 2118895 | 17 | 14 | 0 | 9 | 0 | 0 |
| 361.071738 | 361.071762 | 5065456 | 21 | 14 | 0 | 6 | 0 | 0 |
| 361.092822 | 361.092891 | 5329138 | 18 | 18 | 0 | 8 | 0 | 0 |
| 361.096216 | 361.096262 | 2036210 | 15 | 22 | 0 | 8 | 1 | 0 |
| 361.108147 | 361.108147 | 7074035 | 22 | 18 | 0 | 5 | 0 | 0 |
| 361.119288 | 361.119381 | 1290228 | 21 | 18 | 2 | 4 | 0 | 0 |
| 361.129285 | 361.129277 | 14245621 | 19 | 22 | 0 | 7 | 0 | 0 |
| 361.132653 | 361.132647 | 1729781 | 16 | 26 | 0 | 7 | 1 | 0 |
| 361.144491 | 361.144533 | 11358966 | 23 | 22 | 0 | 4 | 0 | 0 |
| 361.165622 | 361.165662 | 26700536 | 20 | 26 | 0 | 6 | 0 | 0 |
| 361.180918 | 361.180918 | 14238457 | 24 | 26 | 0 | 3 | 0 | 0 |
| 361.202033 | 361.202048 | 17336058 | 21 | 30 | 0 | 5 | 0 | 0 |
| 361.217261 | 361.217304 | 9538300 | 25 | 30 | 0 | 2 | 0 | 0 |
| 361.238451 | 361.238433 | 4721150 | 22 | 34 | 0 | 4 | 0 | 0 |
| 361.253609 | 361.253689 | 2717951 | 26 | 34 | 0 | 1 | 0 | 0 |
| 362.079588 | 362.079897 | 1360577 | 17 | 18 | 1 | 6 | 0 | 1 |
| 362.103405 | 362.103396 | 1616259 | 21 | 17 | 1 | 5 | 0 | 0 |
| 362.116003 | 362.116283 | 1573956 | 18 | 22 | 1 | 5 | 0 | 1 |
| 362.139691 | 362.139782 | 1936454 | 22 | 21 | 1 | 4 | 0 | 0 |
| 362.152368 | 362.152668 | 1579271 | 19 | 26 | 1 | 4 | 0 | 1 |
| 362.160953 | 362.160911 | 1350344 | 19 | 25 | 1 | 6 | 0 | 0 |
| 363.014415 | 363.014641 | 1345165 | 19 | 8 | 0 | 8 | 0 | 0 |
| 363.05095 | 363.051026 | 4369552 | 20 | 12 | 0 | 7 | 0 | 0 |
| 363.072137 | 363.072156 | 1393362 | 17 | 16 | 0 | 9 | 0 | 0 |
| 363.087388 | 363.087412 | 6841747 | 21 | 16 | 0 | 6 | 0 | 0 |
| 363.108501 | 363.108541 | 5127573 | 18 | 20 | 0 | 8 | 0 | 0 |
| 363.111831 | 363.111912 | 2148245 | 15 | 24 | 0 | 8 | 1 | 0 |
| 363.123746 | 363.123797 | 10772374 | 22 | 20 | 0 | 5 | 0 | 0 |
| 363.144912 | 363.144927 | 17464216 | 19 | 24 | 0 | 7 | 0 | 0 |
| 363.160188 | 363.160183 | 17855384 | 23 | 24 | 0 | 4 | 0 | 0 |
| 363.181259 | 363.181312 | 22999962 | 20 | 28 | 0 | 6 | 0 | 0 |
| 363.196528 | 363.196568 | 20437916 | 24 | 28 | 0 | 3 | 0 | 0 |
| 363.217675 | 363.217698 | 8099230 | 21 | 32 | 0 | 5 | 0 | 0 |
| 363.232952 | 363.232954 | 11021215 | 25 | 32 | 0 | 2 | 0 | 0 |
| 363.254118 | 363.254083 | 1341473 | 22 | 36 | 0 | 4 | 0 | 0 |
| 363.269353 | 363.269339 | 1853730 | 26 | 36 | 0 | 1 | 0 | 0 |
| 364.082566 | 364.082661 | 1663460 | 20 | 15 | 1 | 6 | 0 | 0 |
| 364.095196 | 364.095547 | 1520613 | 17 | 20 | 1 | 6 | 0 | 1 |
| 364.119046 | 364.119046 | 2078439 | 21 | 19 | 1 | 5 | 0 | 0 |
| 364.131699 | 364.131933 | 1515752 | 18 | 24 | 1 | 5 | 0 | 1 |
| 364.155478 | 364.155432 | 2156778 | 22 | 23 | 1 | 4 | 0 | 0 |
| 365.03018 | 365.030291 | 2661426 | 19 | 10 | 0 | 8 | 0 | 0 |
| 365.066692 | 365.066676 | 7036469 | 20 | 14 | 0 | 7 | 0 | 0 |
| 365.087827 | 365.087806 | 1855543 | 17 | 18 | 0 | 9 | 0 | 0 |
| 365.103023 | 365.103062 | 9523256 | 21 | 18 | 0 | 6 | 0 | 0 |
| 365.114215 | 365.114295 | 1537337 | 20 | 18 | 2 | 5 | 0 | 0 |
| 365.124174 | 365.124191 | 7117882 | 18 | 22 | 0 | 8 | 0 | 0 |
| 365.139485 | 365.139447 | 17035324 | 22 | 22 | 0 | 5 | 0 | 0 |
| 365.160535 | 365.160577 | 17519676 | 19 | 26 | 0 | 7 | 0 | 0 |
| 365.175757 | 365.175833 | 26555454 | 23 | 26 | 0 | 4 | 0 | 0 |
| 365.212263 | 365.212218 | 21271618 | 24 | 30 | 0 | 3 | 0 | 0 |
| 365.233306 | 365.233348 | 2522947 | 21 | 34 | 0 | 5 | 0 | 0 |
| 365.248532 | 365.248604 | 8842308 | 25 | 34 | 0 | 2 | 0 | 0 |
| 365.285038 | 365.284989 | 1677767 | 26 | 38 | 0 | 1 | 0 | 0 |
| 366.098335 | 366.098311 | 1646859 | 20 | 17 | 1 | 6 | 0 | 0 |
| 366.134698 | 366.134696 | 1886734 | 21 | 21 | 1 | 5 | 0 | 0 |
| 366.17105 | 366.171082 | 1736081 | 22 | 25 | 1 | 4 | 0 | 0 |
| 367.045929 | 367.045941 | 3934170 | 19 | 12 | 0 | 8 | 0 | 0 |
| 367.082354 | 367.082326 | 7048413 | 20 | 16 | 0 | 7 | 0 | 0 |
| 367.097575 | 367.097583 | 1318238 | 24 | 16 | 0 | 4 | 0 | 0 |
| 367.103482 | 367.103456 | 1891935 | 17 | 20 | 0 | 9 | 0 | 0 |
| 367.118672 | 367.118712 | 12210400 | 21 | 20 | 0 | 6 | 0 | 0 |
| 367.122187 | 367.122083 | 1690080 | 18 | 24 | 0 | 6 | 1 | 0 |
| 367.129827 | 367.129945 | 1585121 | 20 | 20 | 2 | 5 | 0 | 0 |
| 367.133857 | 367.133968 | 1411809 | 25 | 20 | 0 | 3 | 0 | 0 |
| 367.139867 | 367.139841 | 6930658 | 18 | 24 | 0 | 8 | 0 | 0 |
| 367.155098 | 367.155097 | 24952034 | 22 | 24 | 0 | 5 | 0 | 0 |
| 367.170514 | 367.170354 | 1385956 | 26 | 24 | 0 | 2 | 0 | 0 |
| 367.176185 | 367.176227 | 10781413 | 19 | 28 | 0 | 7 | 0 | 0 |
| 367.19144 | 367.191483 | 29809894 | 23 | 28 | 0 | 4 | 0 | 0 |
| 367.227899 | 367.227868 | 19219688 | 24 | 32 | 0 | 3 | 0 | 0 |
| 367.264189 | 367.264254 | 5088492 | 25 | 36 | 0 | 2 | 0 | 0 |
| 368.077553 | 368.077575 | 1471664 | 19 | 15 | 1 | 7 | 0 | 0 |
| 368.114031 | 368.113961 | 1737524 | 20 | 19 | 1 | 6 | 0 | 0 |
| 368.150351 | 368.150346 | 2002743 | 21 | 23 | 1 | 5 | 0 | 0 |
| 368.186807 | 368.186732 | 2011450 | 22 | 27 | 1 | 4 | 0 | 0 |
| 369.025133 | 369.025205 | 1580161 | 18 | 10 | 0 | 9 | 0 | 0 |
| 369.06171 | 369.061591 | 4416900 | 19 | 14 | 0 | 8 | 0 | 0 |
| 369.097921 | 369.097976 | 8241543 | 20 | 18 | 0 | 7 | 0 | 0 |
| 369.101185 | 369.101347 | 1670407 | 17 | 22 | 0 | 7 | 1 | 0 |
| 369.113133 | 369.113233 | 2331272 | 24 | 18 | 0 | 4 | 0 | 0 |
| 369.118978 | 369.119106 | 1672201 | 17 | 22 | 0 | 9 | 0 | 0 |
| 369.134366 | 369.134362 | 17308042 | 21 | 22 | 0 | 6 | 0 | 0 |
| 369.137701 | 369.137733 | 1874570 | 18 | 26 | 0 | 6 | 1 | 0 |
| 369.149621 | 369.149618 | 2844171 | 25 | 22 | 0 | 3 | 0 | 0 |
| 369.155447 | 369.155491 | 4867468 | 18 | 26 | 0 | 8 | 0 | 0 |
| 369.170712 | 369.170747 | 30231950 | 22 | 26 | 0 | 5 | 0 | 0 |
| 369.1859 | 369.186004 | 2111118 | 26 | 26 | 0 | 2 | 0 | 0 |
| 369.191849 | 369.191877 | 4448143 | 19 | 30 | 0 | 7 | 0 | 0 |
| 369.207146 | 369.207133 | 27778448 | 23 | 30 | 0 | 4 | 0 | 0 |
| 369.24349 | 369.243519 | 11587987 | 24 | 34 | 0 | 3 | 0 | 0 |
| 369.279823 | 369.279904 | 1701654 | 25 | 38 | 0 | 2 | 0 | 0 |
| 370.129536 | 370.129611 | 1609695 | 20 | 21 | 1 | 6 | 0 | 0 |
| 370.165976 | 370.165996 | 1703522 | 21 | 25 | 1 | 5 | 0 | 0 |
| 371.040958 | 371.040856 | 2066861 | 18 | 12 | 0 | 9 | 0 | 0 |
| 371.056152 | 371.056112 | 1791919 | 22 | 12 | 0 | 6 | 0 | 0 |
| 371.077284 | 371.077241 | 5129521 | 19 | 16 | 0 | 8 | 0 | 0 |
| 371.080468 | 371.080612 | 1588401 | 16 | 20 | 0 | 8 | 1 | 0 |
| 371.092454 | 371.092497 | 3267122 | 23 | 16 | 0 | 5 | 0 | 0 |
| 371.113573 | 371.113627 | 10228276 | 20 | 20 | 0 | 7 | 0 | 0 |
| 371.116862 | 371.116997 | 3311924 | 17 | 24 | 0 | 7 | 1 | 0 |
| 371.12488 | 371.12486 | 1457717 | 19 | 20 | 2 | 6 | 0 | 0 |
| 371.128868 | 371.128883 | 3717685 | 24 | 20 | 0 | 4 | 0 | 0 |
| 371.150008 | 371.150012 | 21800502 | 21 | 24 | 0 | 6 | 0 | 0 |
| 371.165229 | 371.165268 | 5598776 | 25 | 24 | 0 | 3 | 0 | 0 |
| 371.171195 | 371.171141 | 1751737 | 18 | 28 | 0 | 8 | 0 | 0 |
| 371.186368 | 371.186398 | 28991034 | 22 | 28 | 0 | 5 | 0 | 0 |
| 371.201612 | 371.201654 | 4239931 | 26 | 28 | 0 | 2 | 0 | 0 |
| 371.222805 | 371.222783 | 19664444 | 23 | 32 | 0 | 4 | 0 | 0 |
| 371.237985 | 371.238039 | 1389246 | 27 | 32 | 0 | 1 | 0 | 0 |
| 371.259158 | 371.259169 | 4965440 | 24 | 36 | 0 | 3 | 0 | 0 |
| 372.145225 | 372.145261 | 1625741 | 20 | 23 | 1 | 6 | 0 | 0 |
| 372.181523 | 372.181647 | 1910928 | 21 | 27 | 1 | 5 | 0 | 0 |
| 373.056446 | 373.056506 | 2494556 | 18 | 14 | 0 | 9 | 0 | 0 |
| 373.071717 | 373.071762 | 3400414 | 22 | 14 | 0 | 6 | 0 | 0 |
| 373.09295 | 373.092891 | 4633696 | 19 | 18 | 0 | 8 | 0 | 0 |
| 373.096177 | 373.096262 | 2030560 | 16 | 22 | 0 | 8 | 1 | 0 |
| 373.108113 | 373.108147 | 4803809 | 23 | 18 | 0 | 5 | 0 | 0 |
| 373.129294 | 373.129277 | 14483171 | 20 | 22 | 0 | 7 | 0 | 0 |
| 373.132711 | 373.132647 | 2704099 | 17 | 26 | 0 | 7 | 1 | 0 |
| 373.144508 | 373.144533 | 7217892 | 24 | 22 | 0 | 4 | 0 | 0 |
| 373.16563 | 373.165662 | 24088294 | 21 | 26 | 0 | 6 | 0 | 0 |
| 373.180913 | 373.180918 | 8492263 | 25 | 26 | 0 | 3 | 0 | 0 |
| 373.202068 | 373.202048 | 22456042 | 22 | 30 | 0 | 5 | 0 | 0 |
| 373.217265 | 373.217304 | 6059243 | 26 | 30 | 0 | 2 | 0 | 0 |
| 373.238367 | 373.238433 | 10309356 | 23 | 34 | 0 | 4 | 0 | 0 |
| 373.253638 | 373.253689 | 1918190 | 27 | 34 | 0 | 1 | 0 | 0 |
| 373.259513 | 373.259562 | 3303406 | 20 | 38 | 0 | 6 | 0 | 0 |
| 373.274911 | 373.274819 | 2075375 | 24 | 38 | 0 | 3 | 0 | 0 |
| 374.103346 | 374.103396 | 1388088 | 22 | 17 | 1 | 5 | 0 | 0 |
| 374.116116 | 374.116283 | 1523257 | 19 | 22 | 1 | 5 | 0 | 1 |
| 374.160909 | 374.160911 | 2030909 | 20 | 25 | 1 | 6 | 0 | 0 |
| 374.176035 | 374.176167 | 1399999 | 24 | 25 | 1 | 3 | 0 | 0 |
| 375.051105 | 375.051026 | 3403916 | 21 | 12 | 0 | 7 | 0 | 0 |
| 375.072088 | 375.072156 | 2433934 | 18 | 16 | 0 | 9 | 0 | 0 |
| 375.087323 | 375.087412 | 5890447 | 22 | 16 | 0 | 6 | 0 | 0 |
| 375.108553 | 375.108541 | 5632401 | 19 | 20 | 0 | 8 | 0 | 0 |
| 375.112152 | 375.111912 | 1641105 | 16 | 24 | 0 | 8 | 1 | 0 |
| 375.123805 | 375.123797 | 7800211 | 23 | 20 | 0 | 5 | 0 | 0 |
| 375.144932 | 375.144927 | 16353172 | 20 | 24 | 0 | 7 | 0 | 0 |
| 375.148189 | 375.148297 | 1337493 | 17 | 28 | 0 | 7 | 1 | 0 |
| 375.160135 | 375.160183 | 12287894 | 24 | 24 | 0 | 4 | 0 | 0 |
| 375.181251 | 375.181312 | 21229464 | 21 | 28 | 0 | 6 | 0 | 0 |
| 375.196581 | 375.196568 | 13231001 | 25 | 28 | 0 | 3 | 0 | 0 |
| 375.217719 | 375.217698 | 13031323 | 22 | 32 | 0 | 5 | 0 | 0 |
| 375.232931 | 375.232954 | 7556508 | 26 | 32 | 0 | 2 | 0 | 0 |
| 375.254025 | 375.254083 | 3317790 | 23 | 36 | 0 | 4 | 0 | 0 |
| 375.269288 | 375.269339 | 1753759 | 27 | 36 | 0 | 1 | 0 | 0 |
| 375.275247 | 375.275213 | 2629792 | 20 | 40 | 0 | 6 | 0 | 0 |
| 376.082645 | 376.082661 | 1842408 | 21 | 15 | 1 | 6 | 0 | 0 |
| 376.140117 | 376.140176 | 1324013 | 19 | 23 | 1 | 7 | 0 | 0 |
| 376.176304 | 376.176561 | 1382896 | 20 | 27 | 1 | 6 | 0 | 0 |
| 377.030424 | 377.030291 | 1771197 | 20 | 10 | 0 | 8 | 0 | 0 |
| 377.066602 | 377.066676 | 5482048 | 21 | 14 | 0 | 7 | 0 | 0 |
| 377.087753 | 377.087806 | 2075330 | 18 | 18 | 0 | 9 | 0 | 0 |
| 377.103078 | 377.103062 | 6641732 | 22 | 18 | 0 | 6 | 0 | 0 |
| 377.124162 | 377.124191 | 7205445 | 19 | 22 | 0 | 8 | 0 | 0 |
| 377.139417 | 377.139447 | 11814983 | 23 | 22 | 0 | 5 | 0 | 0 |
| 377.160588 | 377.160577 | 18508872 | 20 | 26 | 0 | 7 | 0 | 0 |
| 377.175849 | 377.175833 | 19544138 | 24 | 26 | 0 | 4 | 0 | 0 |
| 377.196932 | 377.196962 | 15551564 | 21 | 30 | 0 | 6 | 0 | 0 |
| 377.21216 | 377.212218 | 16309325 | 25 | 30 | 0 | 3 | 0 | 0 |
| 377.23339 | 377.233348 | 5510735 | 22 | 34 | 0 | 5 | 0 | 0 |
| 377.248666 | 377.248604 | 8108113 | 26 | 34 | 0 | 2 | 0 | 0 |
| 377.284888 | 377.284989 | 1561940 | 27 | 38 | 0 | 1 | 0 | 0 |
| 378.098302 | 378.098311 | 1491613 | 21 | 17 | 1 | 6 | 0 | 0 |
| 378.134717 | 378.134696 | 1579553 | 22 | 21 | 1 | 5 | 0 | 0 |
| 378.147245 | 378.147583 | 1570722 | 19 | 26 | 1 | 5 | 0 | 1 |
| 378.170995 | 378.171082 | 1882532 | 23 | 25 | 1 | 4 | 0 | 0 |
| 378.183633 | 378.183969 | 1565093 | 20 | 30 | 1 | 4 | 0 | 1 |
| 379.045887 | 379.045941 | 3807475 | 20 | 12 | 0 | 8 | 0 | 0 |
| 379.082341 | 379.082326 | 6824695 | 21 | 16 | 0 | 7 | 0 | 0 |
| 379.085667 | 379.085697 | 1498359 | 18 | 20 | 0 | 7 | 1 | 0 |
| 379.103373 | 379.103456 | 2534649 | 18 | 20 | 0 | 9 | 0 | 0 |
| 379.118641 | 379.118712 | 9514746 | 22 | 20 | 0 | 6 | 0 | 0 |
| 379.122192 | 379.122083 | 1715962 | 19 | 24 | 0 | 6 | 1 | 0 |
| 379.130134 | 379.129945 | 1498075 | 21 | 20 | 2 | 5 | 0 | 0 |
| 379.139817 | 379.139841 | 9835772 | 19 | 24 | 0 | 8 | 0 | 0 |
| 379.155096 | 379.155097 | 17341694 | 23 | 24 | 0 | 5 | 0 | 0 |
| 379.176211 | 379.176227 | 16618751 | 20 | 28 | 0 | 7 | 0 | 0 |
| 379.191448 | 379.191483 | 23077120 | 24 | 28 | 0 | 4 | 0 | 0 |
| 379.212621 | 379.212612 | 7794947 | 21 | 32 | 0 | 6 | 0 | 0 |
| 379.22785 | 379.227868 | 16384260 | 25 | 32 | 0 | 3 | 0 | 0 |
| 379.248991 | 379.248998 | 1779334 | 22 | 36 | 0 | 5 | 0 | 0 |
| 379.264204 | 379.264254 | 6094087 | 26 | 36 | 0 | 2 | 0 | 0 |
| 380.113888 | 380.113961 | 1942101 | 21 | 19 | 1 | 6 | 0 | 0 |
| 380.150347 | 380.150346 | 2143064 | 22 | 23 | 1 | 5 | 0 | 0 |
| 380.163048 | 380.163233 | 1346394 | 19 | 28 | 1 | 5 | 0 | 1 |
| 380.18675 | 380.186732 | 1478812 | 23 | 27 | 1 | 4 | 0 | 0 |
| 381.025116 | 381.025205 | 1917609 | 19 | 10 | 0 | 9 | 0 | 0 |
| 381.061581 | 381.061591 | 4624301 | 20 | 14 | 0 | 8 | 0 | 0 |
| 381.076914 | 381.076847 | 1379886 | 24 | 14 | 0 | 5 | 0 | 0 |
| 381.098071 | 381.097976 | 7144368 | 21 | 18 | 0 | 7 | 0 | 0 |
| 381.101394 | 381.101347 | 1532976 | 18 | 22 | 0 | 7 | 1 | 0 |
| 381.10925 | 381.10921 | 1393457 | 20 | 18 | 2 | 6 | 0 | 0 |
| 381.113221 | 381.113233 | 1450161 | 25 | 18 | 0 | 4 | 0 | 0 |
| 381.119114 | 381.119106 | 2690482 | 18 | 22 | 0 | 9 | 0 | 0 |
| 381.134338 | 381.134362 | 13877683 | 22 | 22 | 0 | 6 | 0 | 0 |
| 381.137665 | 381.137733 | 2210995 | 19 | 26 | 0 | 6 | 1 | 0 |
| 381.149679 | 381.149618 | 1666101 | 26 | 22 | 0 | 3 | 0 | 0 |
| 381.155475 | 381.155491 | 9097653 | 19 | 26 | 0 | 8 | 0 | 0 |
| 381.170764 | 381.170747 | 24895926 | 23 | 26 | 0 | 5 | 0 | 0 |
| 381.191872 | 381.191877 | 10159544 | 20 | 30 | 0 | 7 | 0 | 0 |
| 381.207105 | 381.207133 | 24431034 | 24 | 30 | 0 | 4 | 0 | 0 |
| 381.228243 | 381.228262 | 3072444 | 21 | 34 | 0 | 6 | 0 | 0 |
| 381.24354 | 381.243519 | 13814205 | 25 | 34 | 0 | 3 | 0 | 0 |
| 381.279871 | 381.279904 | 3460033 | 26 | 38 | 0 | 2 | 0 | 0 |
| 382.129631 | 382.129611 | 1945103 | 21 | 21 | 1 | 6 | 0 | 0 |
| 382.14212 | 382.142498 | 1556497 | 18 | 26 | 1 | 6 | 0 | 1 |
| 382.165919 | 382.165996 | 1609747 | 22 | 25 | 1 | 5 | 0 | 0 |
| 382.178526 | 382.178883 | 1376404 | 19 | 30 | 1 | 5 | 0 | 1 |
| 382.202517 | 382.202382 | 1675286 | 23 | 29 | 1 | 4 | 0 | 0 |
| 383.040871 | 383.040856 | 2709093 | 19 | 12 | 0 | 9 | 0 | 0 |
| 383.077281 | 383.077241 | 5730408 | 20 | 16 | 0 | 8 | 0 | 0 |
| 383.092658 | 383.092497 | 1822825 | 24 | 16 | 0 | 5 | 0 | 0 |
| 383.113578 | 383.113627 | 9622123 | 21 | 20 | 0 | 7 | 0 | 0 |
| 383.116865 | 383.116997 | 2927212 | 18 | 24 | 0 | 7 | 1 | 0 |
| 383.124855 | 383.12486 | 1572076 | 20 | 20 | 2 | 6 | 0 | 0 |
| 383.128772 | 383.128883 | 2012141 | 25 | 20 | 0 | 4 | 0 | 0 |
| 383.134707 | 383.134756 | 2427501 | 18 | 24 | 0 | 9 | 0 | 0 |
| 383.150038 | 383.150012 | 19509870 | 22 | 24 | 0 | 6 | 0 | 0 |
| 383.153278 | 383.153383 | 2293231 | 19 | 28 | 0 | 6 | 1 | 0 |
| 383.165301 | 383.165268 | 3222640 | 26 | 24 | 0 | 3 | 0 | 0 |
| 383.171101 | 383.171141 | 4651633 | 19 | 28 | 0 | 8 | 0 | 0 |
| 383.186366 | 383.186398 | 27304562 | 23 | 28 | 0 | 5 | 0 | 0 |
| 383.201549 | 383.201654 | 2406516 | 27 | 28 | 0 | 2 | 0 | 0 |
| 383.222776 | 383.222783 | 22837878 | 24 | 32 | 0 | 4 | 0 | 0 |
| 383.259148 | 383.259169 | 7386745 | 25 | 36 | 0 | 3 | 0 | 0 |
| 383.295547 | 383.295554 | 1375228 | 26 | 40 | 0 | 2 | 0 | 0 |
| 384.145394 | 384.145261 | 1986892 | 21 | 23 | 1 | 6 | 0 | 0 |
| 384.181765 | 384.181647 | 1637584 | 22 | 27 | 1 | 5 | 0 | 0 |
| 385.056443 | 385.056506 | 2716963 | 19 | 14 | 0 | 9 | 0 | 0 |
| 385.071751 | 385.071762 | 2757412 | 23 | 14 | 0 | 6 | 0 | 0 |
| 385.092909 | 385.092891 | 6185766 | 20 | 18 | 0 | 8 | 0 | 0 |
| 385.096394 | 385.096262 | 1720102 | 17 | 22 | 0 | 8 | 1 | 0 |
| 385.108051 | 385.108147 | 3019431 | 24 | 18 | 0 | 5 | 0 | 0 |
| 385.129299 | 385.129277 | 11218729 | 21 | 22 | 0 | 7 | 0 | 0 |
| 385.132638 | 385.132647 | 3725098 | 18 | 26 | 0 | 7 | 1 | 0 |
| 385.144603 | 385.144533 | 4583211 | 25 | 22 | 0 | 4 | 0 | 0 |
| 385.150309 | 385.150406 | 1692971 | 18 | 26 | 0 | 9 | 0 | 0 |
| 385.165678 | 385.165662 | 23386924 | 22 | 26 | 0 | 6 | 0 | 0 |
| 385.180906 | 385.180918 | 5479215 | 26 | 26 | 0 | 3 | 0 | 0 |
| 385.202032 | 385.202048 | 25928496 | 23 | 30 | 0 | 5 | 0 | 0 |
| 385.217313 | 385.217304 | 3292082 | 27 | 30 | 0 | 2 | 0 | 0 |
| 385.238479 | 385.238433 | 15113012 | 24 | 34 | 0 | 4 | 0 | 0 |
| 385.253824 | 385.253689 | 1325877 | 28 | 34 | 0 | 1 | 0 | 0 |
| 385.275 | 385.274819 | 3201847 | 25 | 38 | 0 | 3 | 0 | 0 |
| 386.197411 | 386.197297 | 1550607 | 22 | 29 | 1 | 5 | 0 | 0 |
| 387.051017 | 387.051026 | 2588385 | 22 | 12 | 0 | 7 | 0 | 0 |
| 387.072079 | 387.072156 | 2757603 | 19 | 16 | 0 | 9 | 0 | 0 |
| 387.087439 | 387.087412 | 4689381 | 23 | 16 | 0 | 6 | 0 | 0 |
| 387.10865 | 387.108541 | 6713319 | 20 | 20 | 0 | 8 | 0 | 0 |
| 387.111889 | 387.111912 | 2952679 | 17 | 24 | 0 | 8 | 1 | 0 |
| 387.123803 | 387.123797 | 5727720 | 24 | 20 | 0 | 5 | 0 | 0 |
| 387.144914 | 387.144927 | 14238698 | 21 | 24 | 0 | 7 | 0 | 0 |
| 387.148326 | 387.148297 | 3029226 | 18 | 28 | 0 | 7 | 1 | 0 |
| 387.160147 | 387.160183 | 7828972 | 25 | 24 | 0 | 4 | 0 | 0 |
| 387.181348 | 387.181312 | 23858158 | 22 | 28 | 0 | 6 | 0 | 0 |
| 387.196606 | 387.196568 | 8511471 | 26 | 28 | 0 | 3 | 0 | 0 |
| 387.217671 | 387.217698 | 18683888 | 23 | 32 | 0 | 5 | 0 | 0 |
| 387.23295 | 387.232954 | 4411379 | 27 | 32 | 0 | 2 | 0 | 0 |
| 387.254131 | 387.254083 | 6833909 | 24 | 36 | 0 | 4 | 0 | 0 |
| 387.269581 | 387.269339 | 1370614 | 28 | 36 | 0 | 1 | 0 | 0 |
| 388.095432 | 388.095547 | 1320006 | 19 | 20 | 1 | 6 | 0 | 1 |
| 388.131894 | 388.131933 | 1454793 | 20 | 24 | 1 | 5 | 0 | 1 |
| 389.030264 | 389.030291 | 1883552 | 21 | 10 | 0 | 8 | 0 | 0 |
| 389.066699 | 389.066676 | 3963556 | 22 | 14 | 0 | 7 | 0 | 0 |
| 389.087818 | 389.087806 | 3305126 | 19 | 18 | 0 | 9 | 0 | 0 |
| 389.103085 | 389.103062 | 5674152 | 23 | 18 | 0 | 6 | 0 | 0 |
| 389.124166 | 389.124191 | 6555818 | 20 | 22 | 0 | 8 | 0 | 0 |
| 389.127509 | 389.127562 | 2127786 | 17 | 26 | 0 | 8 | 1 | 0 |
| 389.139432 | 389.139447 | 8897707 | 24 | 22 | 0 | 5 | 0 | 0 |
| 389.160611 | 389.160577 | 17910958 | 21 | 26 | 0 | 7 | 0 | 0 |
| 389.16374 | 389.163948 | 1640621 | 18 | 30 | 0 | 7 | 1 | 0 |
| 389.175844 | 389.175833 | 13196463 | 25 | 26 | 0 | 4 | 0 | 0 |
| 389.196931 | 389.196962 | 18897072 | 22 | 30 | 0 | 6 | 0 | 0 |
| 389.212195 | 389.212218 | 11563186 | 26 | 30 | 0 | 3 | 0 | 0 |
| 389.233337 | 389.233348 | 10447028 | 23 | 34 | 0 | 5 | 0 | 0 |
| 389.248577 | 389.248604 | 5444790 | 27 | 34 | 0 | 2 | 0 | 0 |
| 389.254423 | 389.254477 | 1666998 | 20 | 38 | 0 | 7 | 0 | 0 |
| 389.269786 | 389.269733 | 2931384 | 24 | 38 | 0 | 4 | 0 | 0 |
| 390.098484 | 390.098311 | 1347337 | 22 | 17 | 1 | 6 | 0 | 0 |
| 390.134809 | 390.134696 | 1447052 | 23 | 21 | 1 | 5 | 0 | 0 |
| 390.147219 | 390.147583 | 1388430 | 20 | 26 | 1 | 5 | 0 | 1 |
| 390.170781 | 390.17043 | 1329168 | 16 | 29 | 3 | 6 | 1 | 0 |
| 390.183701 | 390.183969 | 1416209 | 21 | 30 | 1 | 4 | 0 | 1 |
| 391.045934 | 391.045941 | 2891110 | 21 | 12 | 0 | 8 | 0 | 0 |
| 391.082332 | 391.082326 | 5375338 | 22 | 16 | 0 | 7 | 0 | 0 |
| 391.103602 | 391.103456 | 2667884 | 19 | 20 | 0 | 9 | 0 | 0 |
| 391.118704 | 391.118712 | 7156589 | 23 | 20 | 0 | 6 | 0 | 0 |
| 391.121884 | 391.122083 | 1412845 | 20 | 24 | 0 | 6 | 1 | 0 |
| 391.130134 | 391.129945 | 1588078 | 22 | 20 | 2 | 5 | 0 | 0 |
| 391.139799 | 391.139841 | 9569135 | 20 | 24 | 0 | 8 | 0 | 0 |
| 391.155123 | 391.155097 | 11965297 | 24 | 24 | 0 | 5 | 0 | 0 |
| 391.176239 | 391.176227 | 17228146 | 21 | 28 | 0 | 7 | 0 | 0 |
| 391.191501 | 391.191483 | 17009012 | 25 | 28 | 0 | 4 | 0 | 0 |
| 391.212573 | 391.212612 | 12778870 | 22 | 32 | 0 | 6 | 0 | 0 |
| 391.22783 | 391.227868 | 13109624 | 26 | 32 | 0 | 3 | 0 | 0 |
| 391.248989 | 391.248998 | 4000122 | 23 | 36 | 0 | 5 | 0 | 0 |
| 391.264276 | 391.264254 | 5797244 | 27 | 36 | 0 | 2 | 0 | 0 |
| 392.113808 | 392.113961 | 1469776 | 22 | 19 | 1 | 6 | 0 | 0 |
| 392.150333 | 392.150346 | 1425747 | 23 | 23 | 1 | 5 | 0 | 0 |
| 393.025258 | 393.025205 | 1419946 | 20 | 10 | 0 | 9 | 0 | 0 |
| 393.061652 | 393.061591 | 4608558 | 21 | 14 | 0 | 8 | 0 | 0 |
| 393.097926 | 393.097976 | 6526514 | 22 | 18 | 0 | 7 | 0 | 0 |
| 393.101407 | 393.101347 | 2034226 | 19 | 22 | 0 | 7 | 1 | 0 |
| 393.11906 | 393.119106 | 3334452 | 19 | 22 | 0 | 9 | 0 | 0 |
| 393.134423 | 393.134362 | 11239989 | 23 | 22 | 0 | 6 | 0 | 0 |
| 393.137975 | 393.137733 | 1463350 | 20 | 26 | 0 | 6 | 1 | 0 |
| 393.155444 | 393.155491 | 12163639 | 20 | 26 | 0 | 8 | 0 | 0 |
| 393.170705 | 393.170747 | 17096248 | 24 | 26 | 0 | 5 | 0 | 0 |
| 393.191833 | 393.191877 | 12885563 | 21 | 30 | 0 | 7 | 0 | 0 |
| 393.207139 | 393.207133 | 20993596 | 25 | 30 | 0 | 4 | 0 | 0 |
| 393.228299 | 393.228262 | 5393471 | 22 | 34 | 0 | 6 | 0 | 0 |
| 393.243498 | 393.243519 | 13148736 | 26 | 34 | 0 | 3 | 0 | 0 |
| 393.279878 | 393.279904 | 4333124 | 27 | 38 | 0 | 2 | 0 | 0 |
| 394.093211 | 394.093225 | 1442453 | 21 | 17 | 1 | 7 | 0 | 0 |
| 394.129483 | 394.129611 | 1630361 | 22 | 21 | 1 | 6 | 0 | 0 |
| 394.165877 | 394.165996 | 1835165 | 23 | 25 | 1 | 5 | 0 | 0 |
| 394.20264 | 394.202382 | 1638816 | 24 | 29 | 1 | 4 | 0 | 0 |
| 395.040991 | 395.040856 | 2579701 | 20 | 12 | 0 | 9 | 0 | 0 |
| 395.077211 | 395.077241 | 4649465 | 21 | 16 | 0 | 8 | 0 | 0 |
| 395.113643 | 395.113627 | 8260860 | 22 | 20 | 0 | 7 | 0 | 0 |
| 395.11698 | 395.116997 | 2753788 | 19 | 24 | 0 | 7 | 1 | 0 |
| 395.124892 | 395.12486 | 1649789 | 21 | 20 | 2 | 6 | 0 | 0 |
| 395.128867 | 395.128883 | 1806590 | 26 | 20 | 0 | 4 | 0 | 0 |
| 395.134745 | 395.134756 | 4899582 | 19 | 24 | 0 | 9 | 0 | 0 |
| 395.14999 | 395.150012 | 14254848 | 23 | 24 | 0 | 6 | 0 | 0 |
| 395.153179 | 395.153383 | 1861120 | 20 | 28 | 0 | 6 | 1 | 0 |
| 395.165108 | 395.165268 | 1594497 | 27 | 24 | 0 | 3 | 0 | 0 |
| 395.171096 | 395.171141 | 9126658 | 20 | 28 | 0 | 8 | 0 | 0 |
| 395.186429 | 395.186398 | 22634244 | 24 | 28 | 0 | 5 | 0 | 0 |
| 395.207612 | 395.207527 | 6468870 | 21 | 32 | 0 | 7 | 0 | 0 |
| 395.22278 | 395.222783 | 20565768 | 25 | 32 | 0 | 4 | 0 | 0 |
| 395.243689 | 395.243912 | 1484041 | 22 | 36 | 0 | 6 | 0 | 0 |
| 395.259142 | 395.259169 | 10203915 | 26 | 36 | 0 | 3 | 0 | 0 |
| 395.295635 | 395.295554 | 2493711 | 27 | 40 | 0 | 2 | 0 | 0 |
| 396.109071 | 396.108876 | 1525089 | 21 | 19 | 1 | 7 | 0 | 0 |
| 396.144949 | 396.144609 | 1440869 | 14 | 27 | 3 | 8 | 1 | 0 |
| 396.181546 | 396.181647 | 1756008 | 23 | 27 | 1 | 5 | 0 | 0 |
| 397.056649 | 397.056506 | 2536130 | 20 | 14 | 0 | 9 | 0 | 0 |
| 397.071807 | 397.071762 | 1628099 | 24 | 14 | 0 | 6 | 0 | 0 |
| 397.092844 | 397.092891 | 4803526 | 21 | 18 | 0 | 8 | 0 | 0 |
| 397.09601 | 397.096262 | 1761478 | 18 | 22 | 0 | 8 | 1 | 0 |
| 397.108107 | 397.108147 | 2491207 | 25 | 18 | 0 | 5 | 0 | 0 |
| 397.129297 | 397.129277 | 10173385 | 22 | 22 | 0 | 7 | 0 | 0 |
| 397.132751 | 397.132647 | 3507658 | 19 | 26 | 0 | 7 | 1 | 0 |
| 397.140436 | 397.14051 | 1480138 | 21 | 22 | 2 | 6 | 0 | 0 |
| 397.144636 | 397.144533 | 3096779 | 26 | 22 | 0 | 4 | 0 | 0 |
| 397.150334 | 397.150406 | 3004875 | 19 | 26 | 0 | 9 | 0 | 0 |
| 397.165577 | 397.165662 | 18844620 | 23 | 26 | 0 | 6 | 0 | 0 |
| 397.168927 | 397.169033 | 2045517 | 20 | 30 | 0 | 6 | 1 | 0 |
| 397.18091 | 397.180918 | 2655694 | 27 | 26 | 0 | 3 | 0 | 0 |
| 397.186723 | 397.186791 | 4292559 | 20 | 30 | 0 | 8 | 0 | 0 |
| 397.202088 | 397.202048 | 25778128 | 24 | 30 | 0 | 5 | 0 | 0 |
| 397.217415 | 397.217304 | 1942866 | 28 | 30 | 0 | 2 | 0 | 0 |
| 397.22343 | 397.223177 | 2229715 | 21 | 34 | 0 | 7 | 0 | 0 |
| 397.238402 | 397.238433 | 15766484 | 25 | 34 | 0 | 4 | 0 | 0 |
| 397.274724 | 397.274819 | 6032344 | 26 | 38 | 0 | 3 | 0 | 0 |
| 398.124453 | 398.124526 | 1517231 | 21 | 21 | 1 | 7 | 0 | 0 |
| 398.161171 | 398.160911 | 1508659 | 22 | 25 | 1 | 6 | 0 | 0 |
| 398.197378 | 398.197297 | 1351415 | 23 | 29 | 1 | 5 | 0 | 0 |
| 399.050935 | 399.051026 | 1731471 | 23 | 12 | 0 | 7 | 0 | 0 |
| 399.07219 | 399.072156 | 3227665 | 20 | 16 | 0 | 9 | 0 | 0 |
| 399.087332 | 399.087412 | 2653075 | 24 | 16 | 0 | 6 | 0 | 0 |
| 399.108539 | 399.108541 | 5751957 | 21 | 20 | 0 | 8 | 0 | 0 |
| 399.112075 | 399.111912 | 2680597 | 18 | 24 | 0 | 8 | 1 | 0 |
| 399.123713 | 399.123797 | 3723415 | 25 | 20 | 0 | 5 | 0 | 0 |
| 399.14494 | 399.144927 | 13672601 | 22 | 24 | 0 | 7 | 0 | 0 |
| 399.148277 | 399.148297 | 3795353 | 19 | 28 | 0 | 7 | 1 | 0 |
| 399.160268 | 399.160183 | 4750234 | 26 | 24 | 0 | 4 | 0 | 0 |
| 399.16601 | 399.166056 | 1393019 | 19 | 28 | 0 | 9 | 0 | 0 |
| 399.181321 | 399.181312 | 23294108 | 23 | 28 | 0 | 6 | 0 | 0 |
| 399.184412 | 399.184683 | 2548125 | 20 | 32 | 0 | 6 | 1 | 0 |
| 399.19654 | 399.196568 | 4466078 | 27 | 28 | 0 | 3 | 0 | 0 |
| 399.202353 | 399.202442 | 1427807 | 20 | 32 | 0 | 8 | 0 | 0 |
| 399.217632 | 399.217698 | 22130848 | 24 | 32 | 0 | 5 | 0 | 0 |
| 399.232896 | 399.232954 | 2713762 | 28 | 32 | 0 | 2 | 0 | 0 |
| 399.254104 | 399.254083 | 10662052 | 25 | 36 | 0 | 4 | 0 | 0 |
| 399.29058 | 399.290469 | 1861416 | 26 | 40 | 0 | 3 | 0 | 0 |
| 400.176446 | 400.176561 | 1414788 | 22 | 27 | 1 | 6 | 0 | 0 |
| 401.051324 | 401.05142 | 1332703 | 19 | 14 | 0 | 10 | 0 | 0 |
| 401.066701 | 401.066676 | 3640673 | 23 | 14 | 0 | 7 | 0 | 0 |
| 401.087788 | 401.087806 | 3394659 | 20 | 18 | 0 | 9 | 0 | 0 |
| 401.091146 | 401.091177 | 1475044 | 17 | 22 | 0 | 9 | 1 | 0 |
| 401.10311 | 401.103062 | 3987301 | 24 | 18 | 0 | 6 | 0 | 0 |
| 401.124164 | 401.124191 | 7741799 | 21 | 22 | 0 | 8 | 0 | 0 |
| 401.127569 | 401.127562 | 3613799 | 18 | 26 | 0 | 8 | 1 | 0 |
| 401.139442 | 401.139447 | 5819753 | 25 | 22 | 0 | 5 | 0 | 0 |
| 401.160538 | 401.160577 | 15654251 | 22 | 26 | 0 | 7 | 0 | 0 |
| 401.163896 | 401.163948 | 3183467 | 19 | 30 | 0 | 7 | 1 | 0 |
| 401.175849 | 401.175833 | 7401325 | 26 | 26 | 0 | 4 | 0 | 0 |
| 401.196912 | 401.196962 | 21518702 | 23 | 30 | 0 | 6 | 0 | 0 |
| 401.212155 | 401.212218 | 6724976 | 27 | 30 | 0 | 3 | 0 | 0 |
| 401.233354 | 401.233348 | 16478578 | 24 | 34 | 0 | 5 | 0 | 0 |
| 401.24868 | 401.248604 | 3577972 | 28 | 34 | 0 | 2 | 0 | 0 |
| 401.26968 | 401.269733 | 4635254 | 25 | 38 | 0 | 4 | 0 | 0 |
| 402.145969 | 402.14593 | 1332050 | 23 | 21 | 3 | 4 | 0 | 0 |
| 402.155856 | 402.155826 | 1371091 | 21 | 25 | 1 | 7 | 0 | 0 |
| 403.045955 | 403.045941 | 2872370 | 22 | 12 | 0 | 8 | 0 | 0 |
| 403.067128 | 403.06707 | 1602868 | 19 | 16 | 0 | 10 | 0 | 0 |
| 403.08232 | 403.082326 | 4528438 | 23 | 16 | 0 | 7 | 0 | 0 |
| 403.103432 | 403.103456 | 3648824 | 20 | 20 | 0 | 9 | 0 | 0 |
| 403.10664 | 403.106827 | 1931064 | 17 | 24 | 0 | 9 | 1 | 0 |
| 403.118607 | 403.118712 | 5840954 | 24 | 20 | 0 | 6 | 0 | 0 |
| 403.139796 | 403.139841 | 8887868 | 21 | 24 | 0 | 8 | 0 | 0 |
| 403.143161 | 403.143212 | 3136316 | 18 | 28 | 0 | 8 | 1 | 0 |
| 403.155134 | 403.155097 | 9167933 | 25 | 24 | 0 | 5 | 0 | 0 |
| 403.176192 | 403.176227 | 17526336 | 22 | 28 | 0 | 7 | 0 | 0 |
| 403.191465 | 403.191483 | 12195393 | 26 | 28 | 0 | 4 | 0 | 0 |
| 403.2126 | 403.212612 | 16759363 | 23 | 32 | 0 | 6 | 0 | 0 |
| 403.227854 | 403.227868 | 9151557 | 27 | 32 | 0 | 3 | 0 | 0 |
| 403.249031 | 403.248998 | 8541767 | 24 | 36 | 0 | 5 | 0 | 0 |
| 403.264296 | 403.264254 | 3639497 | 28 | 36 | 0 | 2 | 0 | 0 |
| 403.285344 | 403.285383 | 1808459 | 25 | 40 | 0 | 4 | 0 | 0 |
| 404.126461 | 404.126848 | 1360677 | 20 | 24 | 1 | 6 | 0 | 1 |
| 404.150413 | 404.150346 | 1484455 | 24 | 23 | 1 | 5 | 0 | 0 |
| 404.161705 | 404.16158 | 1473704 | 23 | 23 | 3 | 4 | 0 | 0 |
| 404.186786 | 404.186732 | 1870763 | 25 | 27 | 1 | 4 | 0 | 0 |
| 405.025264 | 405.025205 | 1601925 | 21 | 10 | 0 | 9 | 0 | 0 |
| 405.061508 | 405.061591 | 3381001 | 22 | 14 | 0 | 8 | 0 | 0 |
| 405.083046 | 405.08272 | 1613067 | 19 | 18 | 0 | 10 | 0 | 0 |
| 405.098031 | 405.097976 | 5517837 | 23 | 18 | 0 | 7 | 0 | 0 |
| 405.119305 | 405.119106 | 4269839 | 20 | 22 | 0 | 9 | 0 | 0 |
| 405.134392 | 405.134362 | 7825681 | 24 | 22 | 0 | 6 | 0 | 0 |
| 405.137467 | 405.137733 | 1331217 | 21 | 26 | 0 | 6 | 1 | 0 |
| 405.155462 | 405.155491 | 11891475 | 21 | 26 | 0 | 8 | 0 | 0 |
| 405.17077 | 405.170747 | 12633876 | 25 | 26 | 0 | 5 | 0 | 0 |
| 405.191872 | 405.191877 | 17405718 | 22 | 30 | 0 | 7 | 0 | 0 |
| 405.207176 | 405.207133 | 14926616 | 26 | 30 | 0 | 4 | 0 | 0 |
| 405.228284 | 405.228262 | 10649370 | 23 | 34 | 0 | 6 | 0 | 0 |
| 405.243493 | 405.243519 | 10818332 | 27 | 34 | 0 | 3 | 0 | 0 |
| 405.279894 | 405.279904 | 3683488 | 28 | 38 | 0 | 2 | 0 | 0 |
| 406.129524 | 406.129611 | 1393916 | 23 | 21 | 1 | 6 | 0 | 0 |
| 406.166162 | 406.165996 | 1444479 | 24 | 25 | 1 | 5 | 0 | 0 |
| 406.202272 | 406.202382 | 1450627 | 25 | 29 | 1 | 4 | 0 | 0 |
| 407.040873 | 407.040856 | 2474462 | 21 | 12 | 0 | 9 | 0 | 0 |
| 407.077223 | 407.077241 | 5132770 | 22 | 16 | 0 | 8 | 0 | 0 |
| 407.113595 | 407.113627 | 7232998 | 23 | 20 | 0 | 7 | 0 | 0 |
| 407.116958 | 407.116997 | 1476646 | 20 | 24 | 0 | 7 | 1 | 0 |
| 407.134814 | 407.134756 | 5401577 | 20 | 24 | 0 | 9 | 0 | 0 |
| 407.149951 | 407.150012 | 11363306 | 24 | 24 | 0 | 6 | 0 | 0 |
| 407.153194 | 407.153383 | 1512938 | 21 | 28 | 0 | 6 | 1 | 0 |
| 407.17115 | 407.171141 | 12044268 | 21 | 28 | 0 | 8 | 0 | 0 |
| 407.186399 | 407.186398 | 17083374 | 25 | 28 | 0 | 5 | 0 | 0 |
| 407.207504 | 407.207527 | 10531824 | 22 | 32 | 0 | 7 | 0 | 0 |
| 407.222762 | 407.222783 | 18173938 | 26 | 32 | 0 | 4 | 0 | 0 |
| 407.243859 | 407.243912 | 4256244 | 23 | 36 | 0 | 6 | 0 | 0 |
| 407.259135 | 407.259169 | 10160118 | 27 | 36 | 0 | 3 | 0 | 0 |
| 407.295505 | 407.295554 | 3125242 | 28 | 40 | 0 | 2 | 0 | 0 |
| 408.145268 | 408.145261 | 1511254 | 23 | 23 | 1 | 6 | 0 | 0 |
| 408.181768 | 408.181647 | 1857626 | 24 | 27 | 1 | 5 | 0 | 0 |
| 409.056562 | 409.056506 | 2745914 | 21 | 14 | 0 | 9 | 0 | 0 |
| 409.092995 | 409.092891 | 5305278 | 22 | 18 | 0 | 8 | 0 | 0 |
| 409.096265 | 409.096262 | 1690559 | 19 | 22 | 0 | 8 | 1 | 0 |
| 409.108306 | 409.108147 | 1824704 | 26 | 18 | 0 | 5 | 0 | 0 |
| 409.113989 | 409.11402 | 1863872 | 19 | 22 | 0 | 10 | 0 | 0 |
| 409.129284 | 409.129277 | 8569026 | 23 | 22 | 0 | 7 | 0 | 0 |
| 409.132542 | 409.132647 | 2619587 | 20 | 26 | 0 | 7 | 1 | 0 |
| 409.144607 | 409.144533 | 1817284 | 27 | 22 | 0 | 4 | 0 | 0 |
| 409.150431 | 409.150406 | 5754565 | 20 | 26 | 0 | 9 | 0 | 0 |
| 409.165662 | 409.165662 | 15067334 | 24 | 26 | 0 | 6 | 0 | 0 |
| 409.169312 | 409.169033 | 1369927 | 21 | 30 | 0 | 6 | 1 | 0 |
| 409.181064 | 409.180918 | 1390024 | 28 | 26 | 0 | 3 | 0 | 0 |
| 409.186854 | 409.186791 | 8341193 | 21 | 30 | 0 | 8 | 0 | 0 |
| 409.202071 | 409.202048 | 21671114 | 25 | 30 | 0 | 5 | 0 | 0 |
| 409.223354 | 409.223177 | 3944397 | 22 | 34 | 0 | 7 | 0 | 0 |
| 409.2384 | 409.238433 | 17082574 | 26 | 34 | 0 | 4 | 0 | 0 |
| 409.274735 | 409.274819 | 7451858 | 27 | 38 | 0 | 3 | 0 | 0 |
| 409.311285 | 409.311204 | 1849174 | 28 | 42 | 0 | 2 | 0 | 0 |
| 410.160847 | 410.160911 | 2060596 | 23 | 25 | 1 | 6 | 0 | 0 |
| 410.197412 | 410.197297 | 1636664 | 24 | 29 | 1 | 5 | 0 | 0 |
| 411.072023 | 411.072156 | 3273881 | 21 | 16 | 0 | 9 | 0 | 0 |
| 411.087511 | 411.087412 | 1836954 | 25 | 16 | 0 | 6 | 0 | 0 |
| 411.108425 | 411.108541 | 5914013 | 22 | 20 | 0 | 8 | 0 | 0 |
| 411.111787 | 411.111912 | 3109789 | 19 | 24 | 0 | 8 | 1 | 0 |
| 411.124037 | 411.123797 | 2477214 | 26 | 20 | 0 | 5 | 0 | 0 |
| 411.129641 | 411.129671 | 1519007 | 19 | 24 | 0 | 10 | 0 | 0 |
| 411.144932 | 411.144927 | 10916257 | 23 | 24 | 0 | 7 | 0 | 0 |
| 411.148301 | 411.148297 | 4019361 | 20 | 28 | 0 | 7 | 1 | 0 |
| 411.160237 | 411.160183 | 3216035 | 27 | 24 | 0 | 4 | 0 | 0 |
| 411.166045 | 411.166056 | 4127139 | 20 | 28 | 0 | 9 | 0 | 0 |
| 411.18131 | 411.181312 | 20364708 | 24 | 28 | 0 | 6 | 0 | 0 |
| 411.196754 | 411.196568 | 2988967 | 28 | 28 | 0 | 3 | 0 | 0 |
| 411.202527 | 411.202442 | 3436711 | 21 | 32 | 0 | 8 | 0 | 0 |
| 411.217673 | 411.217698 | 20497832 | 25 | 32 | 0 | 5 | 0 | 0 |
| 411.232854 | 411.232954 | 1471659 | 29 | 32 | 0 | 2 | 0 | 0 |
| 411.254149 | 411.254083 | 12864429 | 26 | 36 | 0 | 4 | 0 | 0 |
| 411.290487 | 411.290469 | 4537265 | 27 | 40 | 0 | 3 | 0 | 0 |
| 413.051502 | 413.05142 | 1909878 | 20 | 14 | 0 | 10 | 0 | 0 |
| 413.066651 | 413.066676 | 2491959 | 24 | 14 | 0 | 7 | 0 | 0 |
| 413.087826 | 413.087806 | 3368058 | 21 | 18 | 0 | 9 | 0 | 0 |
| 413.091278 | 413.091177 | 1737594 | 18 | 22 | 0 | 9 | 1 | 0 |
| 413.103047 | 413.103062 | 3094267 | 25 | 18 | 0 | 6 | 0 | 0 |
| 413.124269 | 413.124191 | 7759998 | 22 | 22 | 0 | 8 | 0 | 0 |
| 413.127635 | 413.127562 | 4379006 | 19 | 26 | 0 | 8 | 1 | 0 |
| 413.139554 | 413.139447 | 4022656 | 26 | 22 | 0 | 5 | 0 | 0 |
| 413.160579 | 413.160577 | 14980738 | 23 | 26 | 0 | 7 | 0 | 0 |
| 413.163993 | 413.163948 | 4139650 | 20 | 30 | 0 | 7 | 1 | 0 |
| 413.175762 | 413.175833 | 4675204 | 27 | 26 | 0 | 4 | 0 | 0 |
| 413.18153 | 413.181706 | 1718532 | 20 | 30 | 0 | 9 | 0 | 0 |
| 413.196957 | 413.196962 | 21276294 | 24 | 30 | 0 | 6 | 0 | 0 |
| 413.21223 | 413.212218 | 4400776 | 28 | 30 | 0 | 3 | 0 | 0 |
| 413.233356 | 413.233348 | 17684106 | 25 | 34 | 0 | 5 | 0 | 0 |
| 413.248583 | 413.248604 | 1787532 | 29 | 34 | 0 | 2 | 0 | 0 |
| 413.269745 | 413.269733 | 7593614 | 26 | 38 | 0 | 4 | 0 | 0 |
| 413.306254 | 413.306119 | 1493266 | 27 | 42 | 0 | 3 | 0 | 0 |
| 415.045995 | 415.045941 | 1922071 | 23 | 12 | 0 | 8 | 0 | 0 |
| 415.067158 | 415.06707 | 2013785 | 20 | 16 | 0 | 10 | 0 | 0 |
| 415.082212 | 415.082326 | 3703643 | 24 | 16 | 0 | 7 | 0 | 0 |
| 415.103547 | 415.103456 | 3695965 | 21 | 20 | 0 | 9 | 0 | 0 |
| 415.107054 | 415.106827 | 2201694 | 18 | 24 | 0 | 9 | 1 | 0 |
| 415.11861 | 415.118712 | 4389215 | 25 | 20 | 0 | 6 | 0 | 0 |
| 415.139894 | 415.139841 | 9082209 | 22 | 24 | 0 | 8 | 0 | 0 |
| 415.143229 | 415.143212 | 4984674 | 19 | 28 | 0 | 8 | 1 | 0 |
| 415.155166 | 415.155097 | 6114147 | 26 | 24 | 0 | 5 | 0 | 0 |
| 415.176236 | 415.176227 | 16760677 | 23 | 28 | 0 | 7 | 0 | 0 |
| 415.179601 | 415.179598 | 3556454 | 20 | 32 | 0 | 7 | 1 | 0 |
| 415.191458 | 415.191483 | 7154023 | 27 | 28 | 0 | 4 | 0 | 0 |
| 415.21262 | 415.212612 | 19195754 | 24 | 32 | 0 | 6 | 0 | 0 |
| 415.227889 | 415.227868 | 5116524 | 28 | 32 | 0 | 3 | 0 | 0 |
| 415.249013 | 415.248998 | 12557166 | 25 | 36 | 0 | 5 | 0 | 0 |
| 415.26431 | 415.264254 | 2094960 | 29 | 36 | 0 | 2 | 0 | 0 |
| 415.285442 | 415.285383 | 3404914 | 26 | 40 | 0 | 4 | 0 | 0 |
| 416.150427 | 416.150346 | 1357780 | 25 | 23 | 1 | 5 | 0 | 0 |
| 416.161482 | 416.16158 | 1460950 | 24 | 23 | 3 | 4 | 0 | 0 |
| 416.186758 | 416.186732 | 1579481 | 26 | 27 | 1 | 4 | 0 | 0 |
| 417.025152 | 417.025205 | 1511225 | 22 | 10 | 0 | 9 | 0 | 0 |
| 417.061441 | 417.061591 | 2850109 | 23 | 14 | 0 | 8 | 0 | 0 |
| 417.082585 | 417.08272 | 1760831 | 20 | 18 | 0 | 10 | 0 | 0 |
| 417.098002 | 417.097976 | 4360257 | 24 | 18 | 0 | 7 | 0 | 0 |
| 417.119301 | 417.119106 | 4347460 | 21 | 22 | 0 | 9 | 0 | 0 |
| 417.12255 | 417.122477 | 2446148 | 18 | 26 | 0 | 9 | 1 | 0 |
| 417.134329 | 417.134362 | 5722181 | 25 | 22 | 0 | 6 | 0 | 0 |
| 417.155502 | 417.155491 | 10713159 | 22 | 26 | 0 | 8 | 0 | 0 |
| 417.158785 | 417.158862 | 3045704 | 19 | 30 | 0 | 8 | 1 | 0 |
| 417.170773 | 417.170747 | 8212553 | 26 | 26 | 0 | 5 | 0 | 0 |
| 417.191903 | 417.191877 | 17208396 | 23 | 30 | 0 | 7 | 0 | 0 |
| 417.207145 | 417.207133 | 10485325 | 27 | 30 | 0 | 4 | 0 | 0 |
| 417.228273 | 417.228262 | 16094288 | 24 | 34 | 0 | 6 | 0 | 0 |
| 417.243483 | 417.243519 | 7005778 | 28 | 34 | 0 | 3 | 0 | 0 |
| 417.264622 | 417.264648 | 6803540 | 25 | 38 | 0 | 5 | 0 | 0 |
| 417.280097 | 417.279904 | 2290262 | 29 | 38 | 0 | 2 | 0 | 0 |
| 417.300965 | 417.301033 | 1401176 | 26 | 42 | 0 | 4 | 0 | 0 |
| 418.129841 | 418.129611 | 1456952 | 24 | 21 | 1 | 6 | 0 | 0 |
| 418.140777 | 418.140844 | 1363513 | 23 | 21 | 3 | 5 | 0 | 0 |
| 418.166366 | 418.165996 | 1353404 | 25 | 25 | 1 | 5 | 0 | 0 |
| 419.040971 | 419.040856 | 1943585 | 22 | 12 | 0 | 9 | 0 | 0 |
| 419.077331 | 419.077241 | 4668454 | 23 | 16 | 0 | 8 | 0 | 0 |
| 419.098525 | 419.09837 | 1530152 | 20 | 20 | 0 | 10 | 0 | 0 |
| 419.11364 | 419.113627 | 6535978 | 24 | 20 | 0 | 7 | 0 | 0 |
| 419.134807 | 419.134756 | 5323308 | 21 | 24 | 0 | 9 | 0 | 0 |
| 419.138252 | 419.138127 | 1754540 | 18 | 28 | 0 | 9 | 1 | 0 |
| 419.150037 | 419.150012 | 8689966 | 25 | 24 | 0 | 6 | 0 | 0 |
| 419.171154 | 419.171141 | 13628720 | 22 | 28 | 0 | 8 | 0 | 0 |
| 419.174528 | 419.174512 | 1528625 | 19 | 32 | 0 | 8 | 1 | 0 |
| 419.18642 | 419.186398 | 13669682 | 26 | 28 | 0 | 5 | 0 | 0 |
| 419.207604 | 419.207527 | 15089972 | 23 | 32 | 0 | 7 | 0 | 0 |
| 419.222857 | 419.222783 | 12574006 | 27 | 32 | 0 | 4 | 0 | 0 |
| 419.24392 | 419.243912 | 9227577 | 24 | 36 | 0 | 6 | 0 | 0 |
| 419.259211 | 419.259169 | 7814971 | 28 | 36 | 0 | 3 | 0 | 0 |
| 419.280361 | 419.280298 | 2596413 | 25 | 40 | 0 | 5 | 0 | 0 |
| 419.295672 | 419.295554 | 2175807 | 29 | 40 | 0 | 2 | 0 | 0 |
| 420.156378 | 420.156494 | 1695139 | 23 | 23 | 3 | 5 | 0 | 0 |
| 421.056404 | 421.056506 | 2868492 | 22 | 14 | 0 | 9 | 0 | 0 |
| 421.09285 | 421.092891 | 5122065 | 23 | 18 | 0 | 8 | 0 | 0 |
| 421.096635 | 421.096262 | 1366289 | 20 | 22 | 0 | 8 | 1 | 0 |
| 421.114047 | 421.11402 | 2240275 | 20 | 22 | 0 | 10 | 0 | 0 |
| 421.129284 | 421.129277 | 6995861 | 24 | 22 | 0 | 7 | 0 | 0 |
| 421.132458 | 421.132647 | 1746709 | 21 | 26 | 0 | 7 | 1 | 0 |
| 421.150503 | 421.150406 | 6438935 | 21 | 26 | 0 | 9 | 0 | 0 |
| 421.165702 | 421.165662 | 11123225 | 25 | 26 | 0 | 6 | 0 | 0 |
| 421.16891 | 421.169033 | 1401369 | 22 | 30 | 0 | 6 | 1 | 0 |
| 421.186828 | 421.186791 | 12097051 | 22 | 30 | 0 | 8 | 0 | 0 |
| 421.202104 | 421.202048 | 17164830 | 26 | 30 | 0 | 5 | 0 | 0 |
| 421.223169 | 421.223177 | 9096736 | 23 | 34 | 0 | 7 | 0 | 0 |
| 421.238469 | 421.238433 | 14767649 | 27 | 34 | 0 | 4 | 0 | 0 |
| 421.259634 | 421.259562 | 3047076 | 24 | 38 | 0 | 6 | 0 | 0 |
| 421.274829 | 421.274819 | 7468070 | 28 | 38 | 0 | 3 | 0 | 0 |
| 421.311312 | 421.311204 | 1520874 | 29 | 42 | 0 | 2 | 0 | 0 |
| 422.16077 | 422.160911 | 1376142 | 24 | 25 | 1 | 6 | 0 | 0 |
| 422.197234 | 422.197297 | 1386386 | 25 | 29 | 1 | 5 | 0 | 0 |
| 423.035994 | 423.03577 | 1622517 | 21 | 12 | 0 | 10 | 0 | 0 |
| 423.072161 | 423.072156 | 3397626 | 22 | 16 | 0 | 9 | 0 | 0 |
| 423.087278 | 423.087412 | 1427451 | 26 | 16 | 0 | 6 | 0 | 0 |
| 423.108535 | 423.108541 | 5195390 | 23 | 20 | 0 | 8 | 0 | 0 |
| 423.111966 | 423.111912 | 2125438 | 20 | 24 | 0 | 8 | 1 | 0 |
| 423.123993 | 423.123797 | 1694080 | 27 | 20 | 0 | 5 | 0 | 0 |
| 423.129505 | 423.129671 | 2529281 | 20 | 24 | 0 | 10 | 0 | 0 |
| 423.145001 | 423.144927 | 9344258 | 24 | 24 | 0 | 7 | 0 | 0 |
| 423.148365 | 423.148297 | 2702851 | 21 | 28 | 0 | 7 | 1 | 0 |
| 423.159985 | 423.160183 | 1747460 | 28 | 24 | 0 | 4 | 0 | 0 |
| 423.166013 | 423.166056 | 6706437 | 21 | 28 | 0 | 9 | 0 | 0 |
| 423.181349 | 423.181312 | 15325959 | 25 | 28 | 0 | 6 | 0 | 0 |
| 423.18468 | 423.184683 | 1604359 | 22 | 32 | 0 | 6 | 1 | 0 |
| 423.196599 | 423.196568 | 1485192 | 29 | 28 | 0 | 3 | 0 | 0 |
| 423.202464 | 423.202442 | 6714633 | 22 | 32 | 0 | 8 | 0 | 0 |
| 423.217664 | 423.217698 | 19367690 | 26 | 32 | 0 | 5 | 0 | 0 |
| 423.238788 | 423.238827 | 3639053 | 23 | 36 | 0 | 7 | 0 | 0 |
| 423.254133 | 423.254083 | 13901583 | 27 | 36 | 0 | 4 | 0 | 0 |
| 423.290445 | 423.290469 | 5879572 | 28 | 40 | 0 | 3 | 0 | 0 |
| 424.140181 | 424.140176 | 1433464 | 23 | 23 | 1 | 7 | 0 | 0 |
| 425.051184 | 425.05142 | 1754341 | 21 | 14 | 0 | 10 | 0 | 0 |
| 425.0668 | 425.066676 | 2000615 | 25 | 14 | 0 | 7 | 0 | 0 |
| 425.087902 | 425.087806 | 3307113 | 22 | 18 | 0 | 9 | 0 | 0 |
| 425.091302 | 425.091177 | 1839850 | 19 | 22 | 0 | 9 | 1 | 0 |
| 425.103128 | 425.103062 | 2394987 | 26 | 18 | 0 | 6 | 0 | 0 |
| 425.124132 | 425.124191 | 6512622 | 23 | 22 | 0 | 8 | 0 | 0 |
| 425.127766 | 425.127562 | 3634158 | 20 | 26 | 0 | 8 | 1 | 0 |
| 425.13963 | 425.139447 | 2685936 | 27 | 22 | 0 | 5 | 0 | 0 |
| 425.145367 | 425.145321 | 2284784 | 20 | 26 | 0 | 10 | 0 | 0 |
| 425.16055 | 425.160577 | 12526066 | 24 | 26 | 0 | 7 | 0 | 0 |
| 425.164057 | 425.163948 | 3289331 | 21 | 30 | 0 | 7 | 1 | 0 |
| 425.17573 | 425.175833 | 2981108 | 28 | 26 | 0 | 4 | 0 | 0 |
| 425.181822 | 425.181706 | 3458805 | 21 | 30 | 0 | 9 | 0 | 0 |
| 425.196992 | 425.196962 | 19349494 | 25 | 30 | 0 | 6 | 0 | 0 |
| 425.212235 | 425.212218 | 2313720 | 29 | 30 | 0 | 3 | 0 | 0 |
| 425.218081 | 425.218092 | 2589433 | 22 | 34 | 0 | 8 | 0 | 0 |
| 425.233353 | 425.233348 | 19226618 | 26 | 34 | 0 | 5 | 0 | 0 |
| 425.269748 | 425.269733 | 10281983 | 27 | 38 | 0 | 4 | 0 | 0 |
| 425.306216 | 425.306119 | 3880964 | 28 | 42 | 0 | 3 | 0 | 0 |
| 426.192451 | 426.192211 | 1464685 | 24 | 29 | 1 | 6 | 0 | 0 |
| 427.045945 | 427.045941 | 1923540 | 24 | 12 | 0 | 8 | 0 | 0 |
| 427.066962 | 427.06707 | 2258902 | 21 | 16 | 0 | 10 | 0 | 0 |
| 427.082277 | 427.082326 | 2394840 | 25 | 16 | 0 | 7 | 0 | 0 |
| 427.103504 | 427.103456 | 4226779 | 22 | 20 | 0 | 9 | 0 | 0 |
| 427.106822 | 427.106827 | 2972379 | 19 | 24 | 0 | 9 | 1 | 0 |
| 427.11888 | 427.118712 | 3479133 | 26 | 20 | 0 | 6 | 0 | 0 |
| 427.139827 | 427.139841 | 8164063 | 23 | 24 | 0 | 8 | 0 | 0 |
| 427.14318 | 427.143212 | 5279456 | 20 | 28 | 0 | 8 | 1 | 0 |
| 427.155116 | 427.155097 | 3873505 | 27 | 24 | 0 | 5 | 0 | 0 |
| 427.176232 | 427.176227 | 16095459 | 24 | 28 | 0 | 7 | 0 | 0 |
| 427.179631 | 427.179598 | 2960100 | 21 | 32 | 0 | 7 | 1 | 0 |
| 427.191575 | 427.191483 | 3945317 | 28 | 28 | 0 | 4 | 0 | 0 |
| 427.197613 | 427.197356 | 1430758 | 21 | 32 | 0 | 9 | 0 | 0 |
| 427.212652 | 427.212612 | 20638952 | 25 | 32 | 0 | 6 | 0 | 0 |
| 427.22806 | 427.227868 | 2995946 | 29 | 32 | 0 | 3 | 0 | 0 |
| 427.249044 | 427.248998 | 14632172 | 26 | 36 | 0 | 5 | 0 | 0 |
| 427.285328 | 427.285383 | 6412017 | 27 | 40 | 0 | 4 | 0 | 0 |
| 429.061482 | 429.061591 | 2627783 | 24 | 14 | 0 | 8 | 0 | 0 |
| 429.082805 | 429.08272 | 2406857 | 21 | 18 | 0 | 10 | 0 | 0 |
| 429.085984 | 429.086091 | 1467337 | 18 | 22 | 0 | 10 | 1 | 0 |
| 429.097917 | 429.097976 | 3958475 | 25 | 18 | 0 | 7 | 0 | 0 |
| 429.119236 | 429.119106 | 4456654 | 22 | 22 | 0 | 9 | 0 | 0 |
| 429.122591 | 429.122477 | 3918798 | 19 | 26 | 0 | 9 | 1 | 0 |
| 429.134407 | 429.134362 | 4948944 | 26 | 22 | 0 | 6 | 0 | 0 |
| 429.155565 | 429.155491 | 10044370 | 23 | 26 | 0 | 8 | 0 | 0 |
| 429.158913 | 429.158862 | 5418450 | 20 | 30 | 0 | 8 | 1 | 0 |
| 429.170787 | 429.170747 | 6326228 | 27 | 26 | 0 | 5 | 0 | 0 |
| 429.191905 | 429.191877 | 17545686 | 24 | 30 | 0 | 7 | 0 | 0 |
| 429.195406 | 429.195248 | 1549143 | 21 | 34 | 0 | 7 | 1 | 0 |
| 429.207193 | 429.207133 | 6284760 | 28 | 30 | 0 | 4 | 0 | 0 |
| 429.228244 | 429.228262 | 17494490 | 25 | 34 | 0 | 6 | 0 | 0 |
| 429.243606 | 429.243519 | 3828189 | 29 | 34 | 0 | 3 | 0 | 0 |
| 429.26466 | 429.264648 | 9327071 | 26 | 38 | 0 | 5 | 0 | 0 |
| 429.301003 | 429.301033 | 2858467 | 27 | 42 | 0 | 4 | 0 | 0 |
| 431.040629 | 431.040856 | 2032822 | 23 | 12 | 0 | 9 | 0 | 0 |
| 431.077374 | 431.077241 | 3700666 | 24 | 16 | 0 | 8 | 0 | 0 |
| 431.098604 | 431.09837 | 2286781 | 21 | 20 | 0 | 10 | 0 | 0 |
| 431.101734 | 431.101741 | 1636285 | 18 | 24 | 0 | 10 | 1 | 0 |
| 431.113717 | 431.113627 | 4685247 | 25 | 20 | 0 | 7 | 0 | 0 |
| 431.134793 | 431.134756 | 5656769 | 22 | 24 | 0 | 9 | 0 | 0 |
| 431.138061 | 431.138127 | 3571393 | 19 | 28 | 0 | 9 | 1 | 0 |
| 431.150085 | 431.150012 | 6386371 | 26 | 24 | 0 | 6 | 0 | 0 |
| 431.171169 | 431.171141 | 12516037 | 23 | 28 | 0 | 8 | 0 | 0 |
| 431.174549 | 431.174512 | 3380166 | 20 | 32 | 0 | 8 | 1 | 0 |
| 431.186398 | 431.186398 | 8375495 | 27 | 28 | 0 | 5 | 0 | 0 |
| 431.207634 | 431.207527 | 17821386 | 24 | 32 | 0 | 7 | 0 | 0 |
| 431.222736 | 431.222783 | 8421068 | 28 | 32 | 0 | 4 | 0 | 0 |
| 431.243921 | 431.243912 | 12979918 | 25 | 36 | 0 | 6 | 0 | 0 |
| 431.259298 | 431.259169 | 3967184 | 29 | 36 | 0 | 3 | 0 | 0 |
| 431.280224 | 431.280298 | 4298451 | 26 | 40 | 0 | 5 | 0 | 0 |
| 432.156656 | 432.156494 | 1500605 | 24 | 23 | 3 | 5 | 0 | 0 |
| 433.056538 | 433.056506 | 2988202 | 23 | 14 | 0 | 9 | 0 | 0 |
| 433.092949 | 433.092891 | 4729006 | 24 | 18 | 0 | 8 | 0 | 0 |
| 433.096391 | 433.096262 | 1357998 | 21 | 22 | 0 | 8 | 1 | 0 |
| 433.114023 | 433.11402 | 2448049 | 21 | 22 | 0 | 10 | 0 | 0 |
| 433.129294 | 433.129277 | 6207155 | 25 | 22 | 0 | 7 | 0 | 0 |
| 433.132855 | 433.132647 | 1760051 | 22 | 26 | 0 | 7 | 1 | 0 |
| 433.150455 | 433.150406 | 8037301 | 22 | 26 | 0 | 9 | 0 | 0 |
| 433.153882 | 433.153777 | 2482357 | 19 | 30 | 0 | 9 | 1 | 0 |
| 433.165689 | 433.165662 | 8639415 | 26 | 26 | 0 | 6 | 0 | 0 |
| 433.18685 | 433.186791 | 13696953 | 23 | 30 | 0 | 8 | 0 | 0 |
| 433.190405 | 433.190162 | 1529274 | 20 | 34 | 0 | 8 | 1 | 0 |
| 433.202081 | 433.202048 | 11519419 | 27 | 30 | 0 | 5 | 0 | 0 |
| 433.223175 | 433.223177 | 13929406 | 24 | 34 | 0 | 7 | 0 | 0 |
| 433.238342 | 433.238433 | 9482176 | 28 | 34 | 0 | 4 | 0 | 0 |
| 433.259631 | 433.259562 | 7988674 | 25 | 38 | 0 | 6 | 0 | 0 |
| 433.27487 | 433.274819 | 4619716 | 29 | 38 | 0 | 3 | 0 | 0 |
| 433.295986 | 433.295948 | 2010823 | 26 | 42 | 0 | 5 | 0 | 0 |
| 434.160821 | 434.160911 | 1548719 | 25 | 25 | 1 | 6 | 0 | 0 |
| 434.172094 | 434.172144 | 1671473 | 24 | 25 | 3 | 5 | 0 | 0 |
| 434.197529 | 434.197297 | 1442868 | 26 | 29 | 1 | 5 | 0 | 0 |
| 435.035553 | 435.03577 | 1471001 | 22 | 12 | 0 | 10 | 0 | 0 |
| 435.072014 | 435.072156 | 3220510 | 23 | 16 | 0 | 9 | 0 | 0 |
| 435.087716 | 435.087412 | 1366943 | 27 | 16 | 0 | 6 | 0 | 0 |
| 435.10842 | 435.108541 | 5430946 | 24 | 20 | 0 | 8 | 0 | 0 |
| 435.111907 | 435.111912 | 1576610 | 21 | 24 | 0 | 8 | 1 | 0 |
| 435.12952 | 435.129671 | 2711205 | 21 | 24 | 0 | 10 | 0 | 0 |
| 435.144899 | 435.144927 | 7347879 | 25 | 24 | 0 | 7 | 0 | 0 |
| 435.148353 | 435.148297 | 1735975 | 22 | 28 | 0 | 7 | 1 | 0 |
| 435.166103 | 435.166056 | 8226985 | 22 | 28 | 0 | 9 | 0 | 0 |
| 435.18133 | 435.181312 | 12537003 | 26 | 28 | 0 | 6 | 0 | 0 |
| 435.202473 | 435.202442 | 11231405 | 23 | 32 | 0 | 8 | 0 | 0 |
| 435.217738 | 435.217698 | 15038639 | 27 | 32 | 0 | 5 | 0 | 0 |
| 435.238794 | 435.238827 | 7453362 | 24 | 36 | 0 | 7 | 0 | 0 |
| 435.253986 | 435.254083 | 10582195 | 28 | 36 | 0 | 4 | 0 | 0 |
| 435.2753 | 435.275213 | 3168950 | 25 | 40 | 0 | 6 | 0 | 0 |
| 435.290508 | 435.290469 | 3343704 | 29 | 40 | 0 | 3 | 0 | 0 |
| 437.051323 | 437.05142 | 2027917 | 22 | 14 | 0 | 10 | 0 | 0 |
| 437.087793 | 437.087806 | 4139921 | 23 | 18 | 0 | 9 | 0 | 0 |
| 437.091226 | 437.091177 | 2006161 | 20 | 22 | 0 | 9 | 1 | 0 |
| 437.103032 | 437.103062 | 1646483 | 27 | 18 | 0 | 6 | 0 | 0 |
| 437.12426 | 437.124191 | 5752470 | 24 | 22 | 0 | 8 | 0 | 0 |
| 437.127333 | 437.127562 | 2563478 | 21 | 26 | 0 | 8 | 1 | 0 |
| 437.13956 | 437.139447 | 2006167 | 28 | 22 | 0 | 5 | 0 | 0 |
| 437.14529 | 437.145321 | 3447192 | 21 | 26 | 0 | 10 | 0 | 0 |
| 437.160679 | 437.160577 | 10353050 | 25 | 26 | 0 | 7 | 0 | 0 |
| 437.163776 | 437.163948 | 2939674 | 22 | 30 | 0 | 7 | 1 | 0 |
| 437.175735 | 437.175833 | 2005916 | 29 | 26 | 0 | 4 | 0 | 0 |
| 437.181682 | 437.181706 | 6031005 | 22 | 30 | 0 | 9 | 0 | 0 |
| 437.196979 | 437.196962 | 16266654 | 26 | 30 | 0 | 6 | 0 | 0 |
| 437.218182 | 437.218092 | 6487969 | 23 | 34 | 0 | 8 | 0 | 0 |
| 437.233393 | 437.233348 | 17111458 | 27 | 34 | 0 | 5 | 0 | 0 |
| 437.254481 | 437.254477 | 3188389 | 24 | 38 | 0 | 7 | 0 | 0 |
| 437.269745 | 437.269733 | 10369447 | 28 | 38 | 0 | 4 | 0 | 0 |
| 437.306178 | 437.306119 | 3193003 | 29 | 42 | 0 | 3 | 0 | 0 |
| 438.155972 | 438.155826 | 1437714 | 24 | 25 | 1 | 7 | 0 | 0 |
| 438.192268 | 438.192211 | 1792790 | 25 | 29 | 1 | 6 | 0 | 0 |
| 439.030691 | 439.030685 | 1395067 | 21 | 12 | 0 | 11 | 0 | 0 |
| 439.067007 | 439.06707 | 2304127 | 22 | 16 | 0 | 10 | 0 | 0 |
| 439.082513 | 439.082326 | 2119297 | 26 | 16 | 0 | 7 | 0 | 0 |
| 439.103609 | 439.103456 | 4462724 | 23 | 20 | 0 | 9 | 0 | 0 |
| 439.106862 | 439.106827 | 3076228 | 20 | 24 | 0 | 9 | 1 | 0 |
| 439.118626 | 439.118712 | 2567046 | 27 | 20 | 0 | 6 | 0 | 0 |
| 439.139947 | 439.139841 | 7455368 | 24 | 24 | 0 | 8 | 0 | 0 |
| 439.143346 | 439.143212 | 4403593 | 21 | 28 | 0 | 8 | 1 | 0 |
| 439.155184 | 439.155097 | 2825866 | 28 | 24 | 0 | 5 | 0 | 0 |
| 439.161178 | 439.160971 | 2770315 | 21 | 28 | 0 | 10 | 0 | 0 |
| 439.17625 | 439.176227 | 13618828 | 25 | 28 | 0 | 7 | 0 | 0 |
| 439.179679 | 439.179598 | 4135053 | 22 | 32 | 0 | 7 | 1 | 0 |
| 439.191465 | 439.191483 | 2769806 | 29 | 28 | 0 | 4 | 0 | 0 |
| 439.197354 | 439.197356 | 3178255 | 22 | 32 | 0 | 9 | 0 | 0 |
| 439.212673 | 439.212612 | 18194064 | 26 | 32 | 0 | 6 | 0 | 0 |
| 439.22761 | 439.227868 | 1752979 | 30 | 32 | 0 | 3 | 0 | 0 |
| 439.233741 | 439.233742 | 2789267 | 23 | 36 | 0 | 8 | 0 | 0 |
| 439.248987 | 439.248998 | 15728277 | 27 | 36 | 0 | 5 | 0 | 0 |
| 439.285432 | 439.285383 | 9599641 | 28 | 40 | 0 | 4 | 0 | 0 |
| 439.321847 | 439.321769 | 2552734 | 29 | 44 | 0 | 3 | 0 | 0 |
| 440.208093 | 440.207861 | 1373192 | 25 | 31 | 1 | 6 | 0 | 0 |
| 441.061414 | 441.061591 | 2185326 | 25 | 14 | 0 | 8 | 0 | 0 |
| 441.082805 | 441.08272 | 2575729 | 22 | 18 | 0 | 10 | 0 | 0 |
| 441.097973 | 441.097976 | 3026035 | 26 | 18 | 0 | 7 | 0 | 0 |
| 441.119024 | 441.119106 | 4535925 | 23 | 22 | 0 | 9 | 0 | 0 |
| 441.122518 | 441.122477 | 3990902 | 20 | 26 | 0 | 9 | 1 | 0 |
| 441.134417 | 441.134362 | 3379319 | 27 | 22 | 0 | 6 | 0 | 0 |
| 441.155603 | 441.155491 | 9468793 | 24 | 26 | 0 | 8 | 0 | 0 |
| 441.158943 | 441.158862 | 5644666 | 21 | 30 | 0 | 8 | 1 | 0 |
| 441.170845 | 441.170747 | 3639931 | 28 | 26 | 0 | 5 | 0 | 0 |
| 441.176441 | 441.176621 | 1429628 | 21 | 30 | 0 | 10 | 0 | 0 |
| 441.191927 | 441.191877 | 16363390 | 25 | 30 | 0 | 7 | 0 | 0 |
| 441.195303 | 441.195248 | 2444926 | 22 | 34 | 0 | 7 | 1 | 0 |
| 441.207141 | 441.207133 | 4311936 | 29 | 30 | 0 | 4 | 0 | 0 |
| 441.213273 | 441.213006 | 1475328 | 22 | 34 | 0 | 9 | 0 | 0 |
| 441.22822 | 441.228262 | 17210242 | 26 | 34 | 0 | 6 | 0 | 0 |
| 441.243186 | 441.243519 | 2111364 | 30 | 34 | 0 | 3 | 0 | 0 |
| 441.264615 | 441.264648 | 12522374 | 27 | 38 | 0 | 5 | 0 | 0 |
| 441.301082 | 441.301033 | 5645195 | 28 | 42 | 0 | 4 | 0 | 0 |
| 443.041147 | 443.040856 | 1572698 | 24 | 12 | 0 | 9 | 0 | 0 |
| 443.07717 | 443.077241 | 2785118 | 25 | 16 | 0 | 8 | 0 | 0 |
| 443.098549 | 443.09837 | 2570080 | 22 | 20 | 0 | 10 | 0 | 0 |
| 443.101943 | 443.101741 | 1897313 | 19 | 24 | 0 | 10 | 1 | 0 |
| 443.113631 | 443.113627 | 3995746 | 26 | 20 | 0 | 7 | 0 | 0 |
| 443.134845 | 443.134756 | 5948517 | 23 | 24 | 0 | 9 | 0 | 0 |
| 443.138231 | 443.138127 | 5007589 | 20 | 28 | 0 | 9 | 1 | 0 |
| 443.150057 | 443.150012 | 5110375 | 27 | 24 | 0 | 6 | 0 | 0 |
| 443.171154 | 443.171141 | 12421225 | 24 | 28 | 0 | 8 | 0 | 0 |
| 443.174702 | 443.174512 | 4178025 | 21 | 32 | 0 | 8 | 1 | 0 |
| 443.186462 | 443.186398 | 5657195 | 28 | 28 | 0 | 5 | 0 | 0 |
| 443.207505 | 443.207527 | 17355886 | 25 | 32 | 0 | 7 | 0 | 0 |
| 443.222781 | 443.222783 | 5126255 | 29 | 32 | 0 | 4 | 0 | 0 |
| 443.243885 | 443.243912 | 14571634 | 26 | 36 | 0 | 6 | 0 | 0 |
| 443.259168 | 443.259169 | 3009907 | 30 | 36 | 0 | 3 | 0 | 0 |
| 443.280353 | 443.280298 | 7699062 | 27 | 40 | 0 | 5 | 0 | 0 |
| 443.316727 | 443.316683 | 2408058 | 28 | 44 | 0 | 4 | 0 | 0 |
| 445.056545 | 445.056506 | 2096455 | 24 | 14 | 0 | 9 | 0 | 0 |
| 445.077396 | 445.077635 | 1469770 | 21 | 18 | 0 | 11 | 0 | 0 |
| 445.092906 | 445.092891 | 4144715 | 25 | 18 | 0 | 8 | 0 | 0 |
| 445.113932 | 445.11402 | 3070286 | 22 | 22 | 0 | 10 | 0 | 0 |
| 445.117616 | 445.117391 | 2424142 | 19 | 26 | 0 | 10 | 1 | 0 |
| 445.129123 | 445.129277 | 3817168 | 26 | 22 | 0 | 7 | 0 | 0 |
| 445.150388 | 445.150406 | 7039314 | 23 | 26 | 0 | 9 | 0 | 0 |
| 445.153722 | 445.153777 | 4422227 | 20 | 30 | 0 | 9 | 1 | 0 |
| 445.16575 | 445.165662 | 6115156 | 27 | 26 | 0 | 6 | 0 | 0 |
| 445.186753 | 445.186791 | 14242134 | 24 | 30 | 0 | 8 | 0 | 0 |
| 445.190137 | 445.190162 | 1889239 | 21 | 34 | 0 | 8 | 1 | 0 |
| 445.202129 | 445.202048 | 7915864 | 28 | 30 | 0 | 5 | 0 | 0 |
| 445.223221 | 445.223177 | 17716570 | 25 | 34 | 0 | 7 | 0 | 0 |
| 445.238406 | 445.238433 | 5796445 | 29 | 34 | 0 | 4 | 0 | 0 |
| 445.259615 | 445.259562 | 10263903 | 26 | 38 | 0 | 6 | 0 | 0 |
| 445.275018 | 445.274819 | 2400609 | 30 | 38 | 0 | 3 | 0 | 0 |
| 447.035859 | 447.03577 | 1429294 | 23 | 12 | 0 | 10 | 0 | 0 |
| 447.072358 | 447.072156 | 2954802 | 24 | 16 | 0 | 9 | 0 | 0 |
| 447.108629 | 447.108541 | 4664886 | 25 | 20 | 0 | 8 | 0 | 0 |
| 447.129699 | 447.129671 | 3265849 | 22 | 24 | 0 | 10 | 0 | 0 |
| 447.132804 | 447.133041 | 1752633 | 19 | 28 | 0 | 10 | 1 | 0 |
| 447.144971 | 447.144927 | 5889339 | 26 | 24 | 0 | 7 | 0 | 0 |
| 447.148347 | 447.148297 | 1801275 | 23 | 28 | 0 | 7 | 1 | 0 |
| 447.166031 | 447.166056 | 9129021 | 23 | 28 | 0 | 9 | 0 | 0 |
| 447.169681 | 447.169427 | 2434877 | 20 | 32 | 0 | 9 | 1 | 0 |
| 447.181407 | 447.181312 | 9505343 | 27 | 28 | 0 | 6 | 0 | 0 |
| 447.202396 | 447.202442 | 14737985 | 24 | 32 | 0 | 8 | 0 | 0 |
| 447.217766 | 447.217698 | 9559619 | 28 | 32 | 0 | 5 | 0 | 0 |
| 447.238869 | 447.238827 | 13661765 | 25 | 36 | 0 | 7 | 0 | 0 |
| 447.254113 | 447.254083 | 6705735 | 29 | 36 | 0 | 4 | 0 | 0 |
| 447.275284 | 447.275213 | 5590090 | 26 | 40 | 0 | 6 | 0 | 0 |
| 447.290582 | 447.290469 | 2184267 | 30 | 40 | 0 | 3 | 0 | 0 |
| 447.311824 | 447.311598 | 1853006 | 27 | 44 | 0 | 5 | 0 | 0 |
| 448.140401 | 448.140176 | 1520557 | 25 | 23 | 1 | 7 | 0 | 0 |
| 449.051494 | 449.05142 | 1888022 | 23 | 14 | 0 | 10 | 0 | 0 |
| 449.087995 | 449.087806 | 3808026 | 24 | 18 | 0 | 9 | 0 | 0 |
| 449.124282 | 449.124191 | 5059870 | 25 | 22 | 0 | 8 | 0 | 0 |
| 449.127636 | 449.127562 | 2233887 | 22 | 26 | 0 | 8 | 1 | 0 |
| 449.145377 | 449.145321 | 4292897 | 22 | 26 | 0 | 10 | 0 | 0 |
| 449.148442 | 449.148691 | 1379617 | 19 | 30 | 0 | 10 | 1 | 0 |
| 449.160499 | 449.160577 | 7847203 | 26 | 26 | 0 | 7 | 0 | 0 |
| 449.163886 | 449.163948 | 2044451 | 23 | 30 | 0 | 7 | 1 | 0 |
| 449.181766 | 449.181706 | 8875813 | 23 | 30 | 0 | 9 | 0 | 0 |
| 449.196944 | 449.196962 | 12378918 | 27 | 30 | 0 | 6 | 0 | 0 |
| 449.218199 | 449.218092 | 11525929 | 24 | 34 | 0 | 8 | 0 | 0 |
| 449.23336 | 449.233348 | 12940075 | 28 | 34 | 0 | 5 | 0 | 0 |
| 449.254505 | 449.254477 | 6721837 | 25 | 38 | 0 | 7 | 0 | 0 |
| 449.269765 | 449.269733 | 6427951 | 29 | 38 | 0 | 4 | 0 | 0 |
| 449.29075 | 449.290863 | 1941553 | 26 | 42 | 0 | 6 | 0 | 0 |
| 449.306051 | 449.306119 | 1896627 | 30 | 42 | 0 | 3 | 0 | 0 |
| 450.156037 | 450.155826 | 1453460 | 25 | 25 | 1 | 7 | 0 | 0 |
| 450.191985 | 450.191559 | 1413528 | 18 | 33 | 3 | 8 | 1 | 0 |
| 451.067095 | 451.06707 | 2303995 | 23 | 16 | 0 | 10 | 0 | 0 |
| 451.081932 | 451.082326 | 1466364 | 27 | 16 | 0 | 7 | 0 | 0 |
| 451.103428 | 451.103456 | 4001023 | 24 | 20 | 0 | 9 | 0 | 0 |
| 451.107076 | 451.106827 | 1863167 | 21 | 24 | 0 | 9 | 1 | 0 |
| 451.118869 | 451.118712 | 1680128 | 28 | 20 | 0 | 6 | 0 | 0 |
| 451.139934 | 451.139841 | 6367747 | 25 | 24 | 0 | 8 | 0 | 0 |
| 451.143363 | 451.143212 | 3562499 | 22 | 28 | 0 | 8 | 1 | 0 |
| 451.155294 | 451.155097 | 2134532 | 29 | 24 | 0 | 5 | 0 | 0 |
| 451.160798 | 451.160971 | 3846149 | 22 | 28 | 0 | 10 | 0 | 0 |
| 451.176281 | 451.176227 | 10560519 | 26 | 28 | 0 | 7 | 0 | 0 |
| 451.179692 | 451.179598 | 2639623 | 23 | 32 | 0 | 7 | 1 | 0 |
| 451.191756 | 451.191483 | 1455369 | 30 | 28 | 0 | 4 | 0 | 0 |
| 451.197192 | 451.197356 | 6511113 | 23 | 32 | 0 | 9 | 0 | 0 |
| 451.212626 | 451.212612 | 14882827 | 27 | 32 | 0 | 6 | 0 | 0 |
| 451.233749 | 451.233742 | 6193165 | 24 | 36 | 0 | 8 | 0 | 0 |
| 451.24904 | 451.248998 | 13783055 | 28 | 36 | 0 | 5 | 0 | 0 |
| 451.270193 | 451.270127 | 3097617 | 25 | 40 | 0 | 7 | 0 | 0 |
| 451.285461 | 451.285383 | 6204947 | 29 | 40 | 0 | 4 | 0 | 0 |
| 452.171684 | 452.171476 | 1558390 | 25 | 27 | 1 | 7 | 0 | 0 |
| 453.061645 | 453.061591 | 1604569 | 26 | 14 | 0 | 8 | 0 | 0 |
| 453.082914 | 453.08272 | 2571483 | 23 | 18 | 0 | 10 | 0 | 0 |
| 453.086359 | 453.086091 | 1751260 | 20 | 22 | 0 | 10 | 1 | 0 |
| 453.09793 | 453.097976 | 2058973 | 27 | 18 | 0 | 7 | 0 | 0 |
| 453.119193 | 453.119106 | 4498911 | 24 | 22 | 0 | 9 | 0 | 0 |
| 453.122446 | 453.122477 | 3289824 | 21 | 26 | 0 | 9 | 1 | 0 |
| 453.134466 | 453.134362 | 2754529 | 28 | 22 | 0 | 6 | 0 | 0 |
| 453.155599 | 453.155491 | 7861475 | 25 | 26 | 0 | 8 | 0 | 0 |
| 453.158907 | 453.158862 | 5716196 | 22 | 30 | 0 | 8 | 1 | 0 |
| 453.170875 | 453.170747 | 2419429 | 29 | 26 | 0 | 5 | 0 | 0 |
| 453.17679 | 453.176621 | 2631526 | 22 | 30 | 0 | 10 | 0 | 0 |
| 453.191999 | 453.191877 | 14461159 | 26 | 30 | 0 | 7 | 0 | 0 |
| 453.195291 | 453.195248 | 2621416 | 23 | 34 | 0 | 7 | 1 | 0 |
| 453.207354 | 453.207133 | 1832425 | 30 | 30 | 0 | 4 | 0 | 0 |
| 453.213164 | 453.213006 | 3474922 | 23 | 34 | 0 | 9 | 0 | 0 |
| 453.228351 | 453.228262 | 16601323 | 27 | 34 | 0 | 6 | 0 | 0 |
| 453.243408 | 453.243519 | 1853677 | 31 | 34 | 0 | 3 | 0 | 0 |
| 453.249419 | 453.249392 | 2226926 | 24 | 38 | 0 | 8 | 0 | 0 |
| 453.264641 | 453.264648 | 12924143 | 28 | 38 | 0 | 5 | 0 | 0 |
| 453.301021 | 453.301033 | 5891827 | 29 | 42 | 0 | 4 | 0 | 0 |
| 455.062068 | 455.061985 | 1979828 | 22 | 16 | 0 | 11 | 0 | 0 |
| 455.077474 | 455.077241 | 2629045 | 26 | 16 | 0 | 8 | 0 | 0 |
| 455.098556 | 455.09837 | 3117240 | 23 | 20 | 0 | 10 | 0 | 0 |
| 455.101606 | 455.101741 | 1685816 | 20 | 24 | 0 | 10 | 1 | 0 |
| 455.113662 | 455.113627 | 3133369 | 27 | 20 | 0 | 7 | 0 | 0 |
| 455.134803 | 455.134756 | 4892092 | 24 | 24 | 0 | 9 | 0 | 0 |
| 455.138283 | 455.138127 | 5178812 | 21 | 28 | 0 | 9 | 1 | 0 |
| 455.150061 | 455.150012 | 3477693 | 28 | 24 | 0 | 6 | 0 | 0 |
| 455.171222 | 455.171141 | 10203583 | 25 | 28 | 0 | 8 | 0 | 0 |
| 455.174633 | 455.174512 | 5579200 | 22 | 32 | 0 | 8 | 1 | 0 |
| 455.186538 | 455.186398 | 3583425 | 29 | 28 | 0 | 5 | 0 | 0 |
| 455.207519 | 455.207527 | 17301956 | 26 | 32 | 0 | 7 | 0 | 0 |
| 455.222937 | 455.222783 | 3111365 | 30 | 32 | 0 | 4 | 0 | 0 |
| 455.228681 | 455.228656 | 1523910 | 23 | 36 | 0 | 9 | 0 | 0 |
| 455.24393 | 455.243912 | 16906696 | 27 | 36 | 0 | 6 | 0 | 0 |
| 455.259143 | 455.259169 | 1551817 | 31 | 36 | 0 | 3 | 0 | 0 |
| 455.280257 | 455.280298 | 11080139 | 28 | 40 | 0 | 5 | 0 | 0 |
| 455.316743 | 455.316683 | 4750799 | 29 | 44 | 0 | 4 | 0 | 0 |
| 457.056576 | 457.056506 | 2338953 | 25 | 14 | 0 | 9 | 0 | 0 |
| 457.077788 | 457.077635 | 1515147 | 22 | 18 | 0 | 11 | 0 | 0 |
| 457.092924 | 457.092891 | 3702925 | 26 | 18 | 0 | 8 | 0 | 0 |
| 457.113835 | 457.11402 | 2944911 | 23 | 22 | 0 | 10 | 0 | 0 |
| 457.117214 | 457.117391 | 2841616 | 20 | 26 | 0 | 10 | 1 | 0 |
| 457.129383 | 457.129277 | 3948689 | 27 | 22 | 0 | 7 | 0 | 0 |
| 457.150486 | 457.150406 | 7027347 | 24 | 26 | 0 | 9 | 0 | 0 |
| 457.153828 | 457.153777 | 5666452 | 21 | 30 | 0 | 9 | 1 | 0 |
| 457.165766 | 457.165662 | 4773525 | 28 | 26 | 0 | 6 | 0 | 0 |
| 457.186799 | 457.186791 | 13167255 | 25 | 30 | 0 | 8 | 0 | 0 |
| 457.190346 | 457.190162 | 3592343 | 22 | 34 | 0 | 8 | 1 | 0 |
| 457.202071 | 457.202048 | 5425305 | 29 | 30 | 0 | 5 | 0 | 0 |
| 457.223233 | 457.223177 | 16431771 | 26 | 34 | 0 | 7 | 0 | 0 |
| 457.238411 | 457.238433 | 4050332 | 30 | 34 | 0 | 4 | 0 | 0 |
| 457.25968 | 457.259562 | 12960415 | 27 | 38 | 0 | 6 | 0 | 0 |
| 457.274803 | 457.274819 | 1710880 | 31 | 38 | 0 | 3 | 0 | 0 |
| 457.295928 | 457.295948 | 7486115 | 28 | 42 | 0 | 5 | 0 | 0 |
| 457.332604 | 457.332334 | 2276518 | 29 | 46 | 0 | 4 | 0 | 0 |
| 459.072191 | 459.072156 | 3258204 | 25 | 16 | 0 | 9 | 0 | 0 |
| 459.093366 | 459.093285 | 1621854 | 22 | 20 | 0 | 11 | 0 | 0 |
| 459.108507 | 459.108541 | 4146016 | 26 | 20 | 0 | 8 | 0 | 0 |
| 459.129696 | 459.129671 | 3412450 | 23 | 24 | 0 | 10 | 0 | 0 |
| 459.133051 | 459.133041 | 3157346 | 20 | 28 | 0 | 10 | 1 | 0 |
| 459.145004 | 459.144927 | 4718436 | 27 | 24 | 0 | 7 | 0 | 0 |
| 459.166158 | 459.166056 | 8146790 | 24 | 28 | 0 | 9 | 0 | 0 |
| 459.169515 | 459.169427 | 4071270 | 21 | 32 | 0 | 9 | 1 | 0 |
| 459.181396 | 459.181312 | 6559591 | 28 | 28 | 0 | 6 | 0 | 0 |
| 459.202486 | 459.202442 | 15756137 | 25 | 32 | 0 | 8 | 0 | 0 |
| 459.217745 | 459.217698 | 6246763 | 29 | 32 | 0 | 5 | 0 | 0 |
| 459.238849 | 459.238827 | 14003053 | 26 | 36 | 0 | 7 | 0 | 0 |
| 459.253907 | 459.254083 | 4000623 | 30 | 36 | 0 | 4 | 0 | 0 |
| 459.275252 | 459.275213 | 8594289 | 27 | 40 | 0 | 6 | 0 | 0 |
| 459.290209 | 459.290469 | 1660786 | 31 | 40 | 0 | 3 | 0 | 0 |
| 459.311707 | 459.311598 | 3011957 | 28 | 44 | 0 | 5 | 0 | 0 |
| 460.234137 | 460.234076 | 1391059 | 25 | 35 | 1 | 7 | 0 | 0 |
| 461.051652 | 461.05142 | 1676325 | 24 | 14 | 0 | 10 | 0 | 0 |
| 461.087938 | 461.087806 | 3616809 | 25 | 18 | 0 | 9 | 0 | 0 |
| 461.109111 | 461.108935 | 1729067 | 22 | 22 | 0 | 11 | 0 | 0 |
| 461.124282 | 461.124191 | 5041197 | 26 | 22 | 0 | 8 | 0 | 0 |
| 461.145339 | 461.145321 | 4123695 | 23 | 26 | 0 | 10 | 0 | 0 |
| 461.148903 | 461.148691 | 2436399 | 20 | 30 | 0 | 10 | 1 | 0 |
| 461.160642 | 461.160577 | 6347824 | 27 | 26 | 0 | 7 | 0 | 0 |
| 461.181677 | 461.181706 | 10931250 | 24 | 30 | 0 | 9 | 0 | 0 |
| 461.185246 | 461.185077 | 1744947 | 21 | 34 | 0 | 9 | 1 | 0 |
| 461.196958 | 461.196962 | 8049716 | 28 | 30 | 0 | 6 | 0 | 0 |
| 461.218077 | 461.218092 | 14481462 | 25 | 34 | 0 | 8 | 0 | 0 |
| 461.233337 | 461.233348 | 7325240 | 29 | 34 | 0 | 5 | 0 | 0 |
| 461.254497 | 461.254477 | 9960506 | 26 | 38 | 0 | 7 | 0 | 0 |
| 461.26985 | 461.269733 | 3618875 | 30 | 38 | 0 | 4 | 0 | 0 |
| 461.290981 | 461.290863 | 4565053 | 27 | 42 | 0 | 6 | 0 | 0 |
| 461.306043 | 461.306119 | 1409599 | 31 | 42 | 0 | 3 | 0 | 0 |
| 463.067144 | 463.06707 | 2713068 | 24 | 16 | 0 | 10 | 0 | 0 |
| 463.103384 | 463.103456 | 3762672 | 25 | 20 | 0 | 9 | 0 | 0 |
| 463.124259 | 463.124585 | 1450610 | 22 | 24 | 0 | 11 | 0 | 0 |
| 463.139934 | 463.139841 | 5493492 | 26 | 24 | 0 | 8 | 0 | 0 |
| 463.142963 | 463.143212 | 2442228 | 23 | 28 | 0 | 8 | 1 | 0 |
| 463.155321 | 463.155097 | 1626101 | 30 | 24 | 0 | 5 | 0 | 0 |
| 463.160957 | 463.160971 | 4732918 | 23 | 28 | 0 | 10 | 0 | 0 |
| 463.176304 | 463.176227 | 7539959 | 27 | 28 | 0 | 7 | 0 | 0 |
| 463.179764 | 463.179598 | 1932791 | 24 | 32 | 0 | 7 | 1 | 0 |
| 463.197396 | 463.197356 | 10501369 | 24 | 32 | 0 | 9 | 0 | 0 |
| 463.212671 | 463.212612 | 11438330 | 28 | 32 | 0 | 6 | 0 | 0 |
| 463.233753 | 463.233742 | 10875133 | 25 | 36 | 0 | 8 | 0 | 0 |
| 463.248989 | 463.248998 | 8809726 | 29 | 36 | 0 | 5 | 0 | 0 |
| 463.270127 | 463.270127 | 4925696 | 26 | 40 | 0 | 7 | 0 | 0 |
| 463.285204 | 463.285383 | 3111170 | 30 | 40 | 0 | 4 | 0 | 0 |
| 463.30657 | 463.306513 | 1971972 | 27 | 44 | 0 | 6 | 0 | 0 |
| 465.082636 | 465.08272 | 2834733 | 24 | 18 | 0 | 10 | 0 | 0 |
| 465.09803 | 465.097976 | 1486510 | 28 | 18 | 0 | 7 | 0 | 0 |
| 465.119307 | 465.119106 | 4517297 | 25 | 22 | 0 | 9 | 0 | 0 |
| 465.122613 | 465.122477 | 2559409 | 22 | 26 | 0 | 9 | 1 | 0 |
| 465.134726 | 465.134362 | 1686322 | 29 | 22 | 0 | 6 | 0 | 0 |
| 465.140395 | 465.140235 | 1719730 | 22 | 26 | 0 | 11 | 0 | 0 |
| 465.155521 | 465.155491 | 7351220 | 26 | 26 | 0 | 8 | 0 | 0 |
| 465.158864 | 465.158862 | 3726516 | 23 | 30 | 0 | 8 | 1 | 0 |
| 465.171133 | 465.170747 | 1962421 | 30 | 26 | 0 | 5 | 0 | 0 |
| 465.176579 | 465.176621 | 4705590 | 23 | 30 | 0 | 10 | 0 | 0 |
| 465.191875 | 465.191877 | 9968055 | 27 | 30 | 0 | 7 | 0 | 0 |
| 465.195382 | 465.195248 | 2723512 | 24 | 34 | 0 | 7 | 1 | 0 |
| 465.206712 | 465.207133 | 1692345 | 31 | 30 | 0 | 4 | 0 | 0 |
| 465.213011 | 465.213006 | 6770617 | 24 | 34 | 0 | 9 | 0 | 0 |
| 465.228234 | 465.228262 | 12903867 | 28 | 34 | 0 | 6 | 0 | 0 |
| 465.249428 | 465.249392 | 5547965 | 25 | 38 | 0 | 8 | 0 | 0 |
| 465.264714 | 465.264648 | 9032126 | 29 | 38 | 0 | 5 | 0 | 0 |
| 465.28573 | 465.285777 | 2266816 | 26 | 42 | 0 | 7 | 0 | 0 |
| 465.301139 | 465.301033 | 2719938 | 30 | 42 | 0 | 4 | 0 | 0 |
| 466.187223 | 466.187126 | 1650196 | 26 | 29 | 1 | 7 | 0 | 0 |
| 467.061858 | 467.061985 | 2241380 | 23 | 16 | 0 | 11 | 0 | 0 |
| 467.07737 | 467.077241 | 1594085 | 27 | 16 | 0 | 8 | 0 | 0 |
| 467.098079 | 467.09837 | 3095655 | 24 | 20 | 0 | 10 | 0 | 0 |
| 467.101465 | 467.101741 | 1582695 | 21 | 24 | 0 | 10 | 1 | 0 |
| 467.113717 | 467.113627 | 2381928 | 28 | 20 | 0 | 7 | 0 | 0 |
| 467.134683 | 467.134756 | 4968042 | 25 | 24 | 0 | 9 | 0 | 0 |
| 467.138191 | 467.138127 | 4738667 | 22 | 28 | 0 | 9 | 1 | 0 |
| 467.149923 | 467.150012 | 2325612 | 29 | 24 | 0 | 6 | 0 | 0 |
| 467.171161 | 467.171141 | 8957549 | 26 | 28 | 0 | 8 | 0 | 0 |
| 467.174525 | 467.174512 | 4657774 | 23 | 32 | 0 | 8 | 1 | 0 |
| 467.186354 | 467.186398 | 2249455 | 30 | 28 | 0 | 5 | 0 | 0 |
| 467.192014 | 467.192271 | 3390575 | 23 | 32 | 0 | 10 | 0 | 0 |
| 467.207505 | 467.207527 | 15490673 | 27 | 32 | 0 | 7 | 0 | 0 |
| 467.211076 | 467.210898 | 2648945 | 24 | 36 | 0 | 7 | 1 | 0 |
| 467.222952 | 467.222783 | 1926258 | 31 | 32 | 0 | 4 | 0 | 0 |
| 467.228691 | 467.228656 | 3580531 | 24 | 36 | 0 | 9 | 0 | 0 |
| 467.243988 | 467.243912 | 14894708 | 28 | 36 | 0 | 6 | 0 | 0 |
| 467.2646 | 467.265042 | 2015862 | 25 | 40 | 0 | 8 | 0 | 0 |
| 467.280296 | 467.280298 | 9353847 | 29 | 40 | 0 | 5 | 0 | 0 |
| 467.316524 | 467.316683 | 2239355 | 30 | 44 | 0 | 4 | 0 | 0 |
| 468.166637 | 468.16639 | 1631686 | 25 | 27 | 1 | 8 | 0 | 0 |
| 469.056581 | 469.056506 | 1761812 | 26 | 14 | 0 | 9 | 0 | 0 |
| 469.07773 | 469.077635 | 1837590 | 23 | 18 | 0 | 11 | 0 | 0 |
| 469.093081 | 469.092891 | 2296088 | 27 | 18 | 0 | 8 | 0 | 0 |
| 469.113964 | 469.11402 | 3276313 | 24 | 22 | 0 | 10 | 0 | 0 |
| 469.117604 | 469.117391 | 2386714 | 21 | 26 | 0 | 10 | 1 | 0 |
| 469.129386 | 469.129277 | 2940699 | 28 | 22 | 0 | 7 | 0 | 0 |
| 469.150346 | 469.150406 | 5620509 | 25 | 26 | 0 | 9 | 0 | 0 |
| 469.153947 | 469.153777 | 6505245 | 22 | 30 | 0 | 9 | 1 | 0 |
| 469.165689 | 469.165662 | 3343134 | 29 | 26 | 0 | 6 | 0 | 0 |
| 469.186862 | 469.186791 | 11267872 | 26 | 30 | 0 | 8 | 0 | 0 |
| 469.19025 | 469.190162 | 5289760 | 23 | 34 | 0 | 8 | 1 | 0 |
| 469.202201 | 469.202048 | 3364897 | 30 | 30 | 0 | 5 | 0 | 0 |
| 469.22322 | 469.223177 | 16035619 | 27 | 34 | 0 | 7 | 0 | 0 |
| 469.238522 | 469.238433 | 2433060 | 31 | 34 | 0 | 4 | 0 | 0 |
| 469.244418 | 469.244306 | 1676965 | 24 | 38 | 0 | 9 | 0 | 0 |
| 469.259632 | 469.259562 | 14733094 | 28 | 38 | 0 | 6 | 0 | 0 |
| 469.295944 | 469.295948 | 8663337 | 29 | 42 | 0 | 5 | 0 | 0 |
| 469.332357 | 469.332334 | 1675820 | 30 | 46 | 0 | 4 | 0 | 0 |
| 471.072203 | 471.072156 | 2279359 | 26 | 16 | 0 | 9 | 0 | 0 |
| 471.093055 | 471.093285 | 1962561 | 23 | 20 | 0 | 11 | 0 | 0 |
| 471.108651 | 471.108541 | 3378114 | 27 | 20 | 0 | 8 | 0 | 0 |
| 471.129748 | 471.129671 | 3325892 | 24 | 24 | 0 | 10 | 0 | 0 |
| 471.13318 | 471.133041 | 3746244 | 21 | 28 | 0 | 10 | 1 | 0 |
| 471.14504 | 471.144927 | 3166661 | 28 | 24 | 0 | 7 | 0 | 0 |
| 471.166058 | 471.166056 | 7743431 | 25 | 28 | 0 | 9 | 0 | 0 |
| 471.169595 | 471.169427 | 5677511 | 22 | 32 | 0 | 9 | 1 | 0 |
| 471.18128 | 471.181312 | 3781320 | 29 | 28 | 0 | 6 | 0 | 0 |
| 471.202478 | 471.202442 | 14489546 | 26 | 32 | 0 | 8 | 0 | 0 |
| 471.205902 | 471.205812 | 3382730 | 23 | 36 | 0 | 8 | 1 | 0 |
| 471.217747 | 471.217698 | 4330443 | 30 | 32 | 0 | 5 | 0 | 0 |
| 471.238891 | 471.238827 | 16963532 | 27 | 36 | 0 | 7 | 0 | 0 |
| 471.254215 | 471.254083 | 2864590 | 31 | 36 | 0 | 4 | 0 | 0 |
| 471.275231 | 471.275213 | 11860944 | 28 | 40 | 0 | 6 | 0 | 0 |
| 471.311654 | 471.311598 | 9152467 | 29 | 44 | 0 | 5 | 0 | 0 |
| 473.051152 | 473.05142 | 1538911 | 25 | 14 | 0 | 10 | 0 | 0 |
| 473.087821 | 473.087806 | 3066210 | 26 | 18 | 0 | 9 | 0 | 0 |
| 473.108834 | 473.108935 | 1978724 | 23 | 22 | 0 | 11 | 0 | 0 |
| 473.11189 | 473.112306 | 1408996 | 20 | 26 | 0 | 11 | 1 | 0 |
| 473.124207 | 473.124191 | 3682917 | 27 | 22 | 0 | 8 | 0 | 0 |
| 473.145487 | 473.145321 | 4109415 | 24 | 26 | 0 | 10 | 0 | 0 |
| 473.148757 | 473.148691 | 3652199 | 21 | 30 | 0 | 10 | 1 | 0 |
| 473.160643 | 473.160577 | 4840552 | 28 | 26 | 0 | 7 | 0 | 0 |
| 473.181648 | 473.181706 | 10202217 | 25 | 30 | 0 | 9 | 0 | 0 |
| 473.185184 | 473.185077 | 3790954 | 22 | 34 | 0 | 9 | 1 | 0 |
| 473.196868 | 473.196962 | 5656171 | 29 | 30 | 0 | 6 | 0 | 0 |
| 473.218141 | 473.218092 | 14622828 | 26 | 34 | 0 | 8 | 0 | 0 |
| 473.233387 | 473.233348 | 4692078 | 30 | 34 | 0 | 5 | 0 | 0 |
| 473.254595 | 473.254477 | 14098543 | 27 | 38 | 0 | 7 | 0 | 0 |
| 473.269983 | 473.269733 | 2840176 | 31 | 38 | 0 | 4 | 0 | 0 |
| 473.290943 | 473.290863 | 7806578 | 28 | 42 | 0 | 6 | 0 | 0 |
| 473.327233 | 473.327248 | 3078261 | 29 | 46 | 0 | 5 | 0 | 0 |
| 475.06718 | 475.06707 | 2228986 | 25 | 16 | 0 | 10 | 0 | 0 |
| 475.103404 | 475.103456 | 4115196 | 26 | 20 | 0 | 9 | 0 | 0 |
| 475.12463 | 475.124585 | 2248702 | 23 | 24 | 0 | 11 | 0 | 0 |
| 475.127862 | 475.127956 | 1939582 | 20 | 28 | 0 | 11 | 1 | 0 |
| 475.139877 | 475.139841 | 4643071 | 27 | 24 | 0 | 8 | 0 | 0 |
| 475.161073 | 475.160971 | 6031105 | 24 | 28 | 0 | 10 | 0 | 0 |
| 475.16454 | 475.164341 | 2472449 | 21 | 32 | 0 | 10 | 1 | 0 |
| 475.176273 | 475.176227 | 6473474 | 28 | 28 | 0 | 7 | 0 | 0 |
| 475.197379 | 475.197356 | 11830531 | 25 | 32 | 0 | 9 | 0 | 0 |
| 475.212659 | 475.212612 | 7312132 | 29 | 32 | 0 | 6 | 0 | 0 |
| 475.233804 | 475.233742 | 14056710 | 26 | 36 | 0 | 8 | 0 | 0 |
| 475.248991 | 475.248998 | 5069575 | 30 | 36 | 0 | 5 | 0 | 0 |
| 475.270107 | 475.270127 | 9781512 | 27 | 40 | 0 | 7 | 0 | 0 |
| 475.285475 | 475.285383 | 2024842 | 31 | 40 | 0 | 4 | 0 | 0 |
| 475.306585 | 475.306513 | 3872011 | 28 | 44 | 0 | 6 | 0 | 0 |
| 477.082764 | 477.08272 | 2932619 | 25 | 18 | 0 | 10 | 0 | 0 |
| 477.119122 | 477.119106 | 4328845 | 26 | 22 | 0 | 9 | 0 | 0 |
| 477.122249 | 477.122477 | 1611661 | 23 | 26 | 0 | 9 | 1 | 0 |
| 477.134092 | 477.134362 | 1412750 | 30 | 22 | 0 | 6 | 0 | 0 |
| 477.140192 | 477.140235 | 2350223 | 23 | 26 | 0 | 11 | 0 | 0 |
| 477.155641 | 477.155491 | 5414288 | 27 | 26 | 0 | 8 | 0 | 0 |
| 477.159154 | 477.158862 | 2423184 | 24 | 30 | 0 | 8 | 1 | 0 |
| 477.176608 | 477.176621 | 6570641 | 24 | 30 | 0 | 10 | 0 | 0 |
| 477.191867 | 477.191877 | 7726482 | 28 | 30 | 0 | 7 | 0 | 0 |
| 477.195143 | 477.195248 | 1614867 | 25 | 34 | 0 | 7 | 1 | 0 |
| 477.213015 | 477.213006 | 11331988 | 25 | 34 | 0 | 9 | 0 | 0 |
| 477.228349 | 477.228262 | 9004437 | 29 | 34 | 0 | 6 | 0 | 0 |
| 477.249409 | 477.249392 | 8905622 | 26 | 38 | 0 | 8 | 0 | 0 |
| 477.264674 | 477.264648 | 5473688 | 30 | 38 | 0 | 5 | 0 | 0 |
| 477.301176 | 477.301033 | 1606810 | 31 | 42 | 0 | 4 | 0 | 0 |
| 477.322395 | 477.322163 | 1635739 | 28 | 46 | 0 | 6 | 0 | 0 |
| 479.062001 | 479.061985 | 1923344 | 24 | 16 | 0 | 11 | 0 | 0 |
| 479.098416 | 479.09837 | 3069715 | 25 | 20 | 0 | 10 | 0 | 0 |
| 479.101795 | 479.101741 | 1514259 | 22 | 24 | 0 | 10 | 1 | 0 |
| 479.113798 | 479.113627 | 1526291 | 29 | 20 | 0 | 7 | 0 | 0 |
| 479.134743 | 479.134756 | 4474901 | 26 | 24 | 0 | 9 | 0 | 0 |
| 479.138113 | 479.138127 | 3251733 | 23 | 28 | 0 | 9 | 1 | 0 |
| 479.149757 | 479.150012 | 1914902 | 30 | 24 | 0 | 6 | 0 | 0 |
| 479.155964 | 479.155885 | 2152470 | 23 | 28 | 0 | 11 | 0 | 0 |
| 479.171262 | 479.171141 | 6875671 | 27 | 28 | 0 | 8 | 0 | 0 |
| 479.174483 | 479.174512 | 3737623 | 24 | 32 | 0 | 8 | 1 | 0 |
| 479.18602 | 479.186398 | 1879064 | 31 | 28 | 0 | 5 | 0 | 0 |
| 479.192343 | 479.192271 | 6103577 | 24 | 32 | 0 | 10 | 0 | 0 |
| 479.207568 | 479.207527 | 10950170 | 28 | 32 | 0 | 7 | 0 | 0 |
| 479.211076 | 479.210898 | 2233882 | 25 | 36 | 0 | 7 | 1 | 0 |
| 479.22863 | 479.228656 | 8014363 | 25 | 36 | 0 | 9 | 0 | 0 |
| 479.244051 | 479.243912 | 10240540 | 29 | 36 | 0 | 6 | 0 | 0 |
| 479.264996 | 479.265042 | 4081437 | 26 | 40 | 0 | 8 | 0 | 0 |
| 479.280289 | 479.280298 | 5077022 | 30 | 40 | 0 | 5 | 0 | 0 |
| 481.07751 | 481.077635 | 2073230 | 24 | 18 | 0 | 11 | 0 | 0 |
| 481.093026 | 481.092891 | 2273679 | 28 | 18 | 0 | 8 | 0 | 0 |
| 481.114169 | 481.11402 | 3182224 | 25 | 22 | 0 | 10 | 0 | 0 |
| 481.117404 | 481.117391 | 2615953 | 22 | 26 | 0 | 10 | 1 | 0 |
| 481.129284 | 481.129277 | 2527889 | 29 | 22 | 0 | 7 | 0 | 0 |
| 481.150399 | 481.150406 | 5616275 | 26 | 26 | 0 | 9 | 0 | 0 |
| 481.153744 | 481.153777 | 4350099 | 23 | 30 | 0 | 9 | 1 | 0 |
| 481.165842 | 481.165662 | 2477204 | 30 | 26 | 0 | 6 | 0 | 0 |
| 481.17157 | 481.171535 | 2104724 | 23 | 30 | 0 | 11 | 0 | 0 |
| 481.186762 | 481.186791 | 9430677 | 27 | 30 | 0 | 8 | 0 | 0 |
| 481.190281 | 481.190162 | 4962965 | 24 | 34 | 0 | 8 | 1 | 0 |
| 481.20203 | 481.202048 | 4154006 | 31 | 30 | 0 | 5 | 0 | 0 |
| 481.207855 | 481.207921 | 3461014 | 24 | 34 | 0 | 10 | 0 | 0 |
| 481.223224 | 481.223177 | 13920919 | 28 | 34 | 0 | 7 | 0 | 0 |
| 481.226743 | 481.226548 | 2213015 | 25 | 38 | 0 | 7 | 1 | 0 |
| 481.238395 | 481.238433 | 1680536 | 32 | 34 | 0 | 4 | 0 | 0 |
| 481.244389 | 481.244306 | 3693720 | 25 | 38 | 0 | 9 | 0 | 0 |
| 481.259583 | 481.259562 | 10953369 | 29 | 38 | 0 | 6 | 0 | 0 |
| 481.296066 | 481.295948 | 5010075 | 30 | 42 | 0 | 5 | 0 | 0 |
| 483.072338 | 483.072156 | 1750529 | 27 | 16 | 0 | 9 | 0 | 0 |
| 483.093193 | 483.093285 | 2152194 | 24 | 20 | 0 | 11 | 0 | 0 |
| 483.108633 | 483.108541 | 3175683 | 28 | 20 | 0 | 8 | 0 | 0 |
| 483.129878 | 483.129671 | 3461380 | 25 | 24 | 0 | 10 | 0 | 0 |
| 483.133199 | 483.133041 | 4483332 | 22 | 28 | 0 | 10 | 1 | 0 |
| 483.144841 | 483.144927 | 2936325 | 29 | 24 | 0 | 7 | 0 | 0 |
| 483.165953 | 483.166056 | 6959366 | 26 | 28 | 0 | 9 | 0 | 0 |
| 483.169578 | 483.169427 | 6316806 | 23 | 32 | 0 | 9 | 1 | 0 |
| 483.181394 | 483.181312 | 2990599 | 30 | 28 | 0 | 6 | 0 | 0 |
| 483.202468 | 483.202442 | 12454664 | 27 | 32 | 0 | 8 | 0 | 0 |
| 483.205866 | 483.205812 | 5161224 | 24 | 36 | 0 | 8 | 1 | 0 |
| 483.217804 | 483.217698 | 2961929 | 31 | 32 | 0 | 5 | 0 | 0 |
| 483.223699 | 483.223571 | 1956105 | 24 | 36 | 0 | 10 | 0 | 0 |
| 483.238918 | 483.238827 | 14998282 | 28 | 36 | 0 | 7 | 0 | 0 |
| 483.254124 | 483.254083 | 2114571 | 32 | 36 | 0 | 4 | 0 | 0 |
| 483.260006 | 483.259956 | 1448459 | 25 | 40 | 0 | 9 | 0 | 0 |
| 483.275162 | 483.275213 | 11031308 | 29 | 40 | 0 | 6 | 0 | 0 |
| 483.31166 | 483.311598 | 4395278 | 30 | 44 | 0 | 5 | 0 | 0 |
| 484.197794 | 484.197691 | 1469501 | 26 | 31 | 1 | 8 | 0 | 0 |
| 485.08788 | 485.087806 | 2924138 | 27 | 18 | 0 | 9 | 0 | 0 |
| 485.108839 | 485.108935 | 2496107 | 24 | 22 | 0 | 11 | 0 | 0 |
| 485.112424 | 485.112306 | 1860459 | 21 | 26 | 0 | 11 | 1 | 0 |
| 485.124029 | 485.124191 | 3151724 | 28 | 22 | 0 | 8 | 0 | 0 |
| 485.145338 | 485.145321 | 4512621 | 25 | 26 | 0 | 10 | 0 | 0 |
| 485.148774 | 485.148691 | 4671853 | 22 | 30 | 0 | 10 | 1 | 0 |
| 485.160586 | 485.160577 | 3918190 | 29 | 26 | 0 | 7 | 0 | 0 |
| 485.181701 | 485.181706 | 8465262 | 26 | 30 | 0 | 9 | 0 | 0 |
| 485.185132 | 485.185077 | 5464431 | 23 | 34 | 0 | 9 | 1 | 0 |
| 485.196975 | 485.196962 | 4293487 | 30 | 30 | 0 | 6 | 0 | 0 |
| 485.218153 | 485.218092 | 14838640 | 27 | 34 | 0 | 8 | 0 | 0 |
| 485.221634 | 485.221462 | 2564465 | 24 | 38 | 0 | 8 | 1 | 0 |
| 485.233321 | 485.233348 | 3771505 | 31 | 34 | 0 | 5 | 0 | 0 |
| 485.254541 | 485.254477 | 15739762 | 28 | 38 | 0 | 7 | 0 | 0 |
| 485.269583 | 485.269733 | 2057331 | 32 | 38 | 0 | 4 | 0 | 0 |
| 485.290934 | 485.290863 | 11872116 | 29 | 42 | 0 | 6 | 0 | 0 |
| 485.327366 | 485.327248 | 4465526 | 30 | 46 | 0 | 5 | 0 | 0 |
| 487.06707 | 487.06707 | 1865286 | 26 | 16 | 0 | 10 | 0 | 0 |
| 487.103603 | 487.103456 | 2628040 | 27 | 20 | 0 | 9 | 0 | 0 |
| 487.12472 | 487.124585 | 1942473 | 24 | 24 | 0 | 11 | 0 | 0 |
| 487.128055 | 487.127956 | 2721225 | 21 | 28 | 0 | 11 | 1 | 0 |
| 487.139822 | 487.139841 | 3903433 | 28 | 24 | 0 | 8 | 0 | 0 |
| 487.161026 | 487.160971 | 5648330 | 25 | 28 | 0 | 10 | 0 | 0 |
| 487.164422 | 487.164341 | 4587979 | 22 | 32 | 0 | 10 | 1 | 0 |
| 487.176266 | 487.176227 | 4734411 | 29 | 28 | 0 | 7 | 0 | 0 |
| 487.197442 | 487.197356 | 11865036 | 26 | 32 | 0 | 9 | 0 | 0 |
| 487.200904 | 487.200727 | 3666380 | 23 | 36 | 0 | 9 | 1 | 0 |
| 487.212571 | 487.212612 | 4888013 | 30 | 32 | 0 | 6 | 0 | 0 |
| 487.233804 | 487.233742 | 14626765 | 27 | 36 | 0 | 8 | 0 | 0 |
| 487.248951 | 487.248998 | 3870158 | 31 | 36 | 0 | 5 | 0 | 0 |
| 487.270167 | 487.270127 | 13126607 | 28 | 40 | 0 | 7 | 0 | 0 |
| 487.285101 | 487.285383 | 1635024 | 32 | 40 | 0 | 4 | 0 | 0 |
| 487.306558 | 487.306513 | 9778129 | 29 | 44 | 0 | 6 | 0 | 0 |
| 487.343125 | 487.342898 | 3120594 | 30 | 48 | 0 | 5 | 0 | 0 |
| 489.082839 | 489.08272 | 2750489 | 26 | 18 | 0 | 10 | 0 | 0 |
| 489.119149 | 489.119106 | 4088346 | 27 | 22 | 0 | 9 | 0 | 0 |
| 489.140318 | 489.140235 | 2594075 | 24 | 26 | 0 | 11 | 0 | 0 |
| 489.143621 | 489.143606 | 1771803 | 21 | 30 | 0 | 11 | 1 | 0 |
| 489.155598 | 489.155491 | 4531740 | 28 | 26 | 0 | 8 | 0 | 0 |
| 489.176383 | 489.176621 | 6794524 | 25 | 30 | 0 | 10 | 0 | 0 |
| 489.179955 | 489.179992 | 2679452 | 22 | 34 | 0 | 10 | 1 | 0 |
| 489.191902 | 489.191877 | 5509149 | 29 | 30 | 0 | 7 | 0 | 0 |
| 489.212955 | 489.213006 | 12652573 | 26 | 34 | 0 | 9 | 0 | 0 |
| 489.22828 | 489.228262 | 5868574 | 30 | 34 | 0 | 6 | 0 | 0 |
| 489.249409 | 489.249392 | 12796959 | 27 | 38 | 0 | 8 | 0 | 0 |
| 489.264707 | 489.264648 | 4007456 | 31 | 38 | 0 | 5 | 0 | 0 |
| 489.285774 | 489.285777 | 9008160 | 28 | 42 | 0 | 7 | 0 | 0 |
| 489.301315 | 489.301033 | 1702177 | 32 | 42 | 0 | 4 | 0 | 0 |
| 489.322222 | 489.322163 | 5508642 | 29 | 46 | 0 | 6 | 0 | 0 |
| 491.061966 | 491.061985 | 1842014 | 25 | 16 | 0 | 11 | 0 | 0 |
| 491.098379 | 491.09837 | 3126367 | 26 | 20 | 0 | 10 | 0 | 0 |
| 491.134748 | 491.134756 | 3873888 | 27 | 24 | 0 | 9 | 0 | 0 |
| 491.138148 | 491.138127 | 2203745 | 24 | 28 | 0 | 9 | 1 | 0 |
| 491.15592 | 491.155885 | 3210849 | 24 | 28 | 0 | 11 | 0 | 0 |
| 491.171072 | 491.171141 | 5252194 | 28 | 28 | 0 | 8 | 0 | 0 |
| 491.174524 | 491.174512 | 2057634 | 25 | 32 | 0 | 8 | 1 | 0 |
| 491.192332 | 491.192271 | 8479842 | 25 | 32 | 0 | 10 | 0 | 0 |
| 491.207586 | 491.207527 | 7475299 | 29 | 32 | 0 | 7 | 0 | 0 |
| 491.22869 | 491.228656 | 10962019 | 26 | 36 | 0 | 9 | 0 | 0 |
| 491.243798 | 491.243912 | 6192740 | 30 | 36 | 0 | 6 | 0 | 0 |
| 491.265082 | 491.265042 | 8512612 | 27 | 40 | 0 | 8 | 0 | 0 |
| 491.280272 | 491.280298 | 3843685 | 31 | 40 | 0 | 5 | 0 | 0 |
| 491.301422 | 491.301427 | 3395174 | 28 | 44 | 0 | 7 | 0 | 0 |
| 493.077692 | 493.077635 | 1833625 | 25 | 18 | 0 | 11 | 0 | 0 |
| 493.114144 | 493.11402 | 2870938 | 26 | 22 | 0 | 10 | 0 | 0 |
| 493.11705 | 493.117391 | 1742746 | 23 | 26 | 0 | 10 | 1 | 0 |
| 493.129368 | 493.129277 | 1937819 | 30 | 22 | 0 | 7 | 0 | 0 |
| 493.150459 | 493.150406 | 4873883 | 27 | 26 | 0 | 9 | 0 | 0 |
| 493.15397 | 493.153777 | 3283099 | 24 | 30 | 0 | 9 | 1 | 0 |
| 493.171668 | 493.171535 | 3508892 | 24 | 30 | 0 | 11 | 0 | 0 |
| 493.18679 | 493.186791 | 7145628 | 28 | 30 | 0 | 8 | 0 | 0 |
| 493.190276 | 493.190162 | 3440284 | 25 | 34 | 0 | 8 | 1 | 0 |
| 493.208022 | 493.207921 | 6673565 | 25 | 34 | 0 | 10 | 0 | 0 |
| 493.223193 | 493.223177 | 9100445 | 29 | 34 | 0 | 7 | 0 | 0 |
| 493.244264 | 493.244306 | 6578846 | 26 | 38 | 0 | 9 | 0 | 0 |
| 493.259602 | 493.259562 | 7067806 | 30 | 38 | 0 | 6 | 0 | 0 |
| 493.295953 | 493.295948 | 2751135 | 31 | 42 | 0 | 5 | 0 | 0 |
| 495.072331 | 495.072156 | 1694153 | 28 | 16 | 0 | 9 | 0 | 0 |
| 495.093245 | 495.093285 | 2194378 | 25 | 20 | 0 | 11 | 0 | 0 |
| 495.108685 | 495.108541 | 1947850 | 29 | 20 | 0 | 8 | 0 | 0 |
| 495.129748 | 495.129671 | 3507914 | 26 | 24 | 0 | 10 | 0 | 0 |
| 495.13302 | 495.133041 | 2503882 | 23 | 28 | 0 | 10 | 1 | 0 |
| 495.145061 | 495.144927 | 2363083 | 30 | 24 | 0 | 7 | 0 | 0 |
| 495.16609 | 495.166056 | 5895883 | 27 | 28 | 0 | 9 | 0 | 0 |
| 495.169544 | 495.169427 | 5409995 | 24 | 32 | 0 | 9 | 1 | 0 |
| 495.181122 | 495.181312 | 1814859 | 31 | 28 | 0 | 6 | 0 | 0 |
| 495.187339 | 495.187185 | 2441420 | 24 | 32 | 0 | 11 | 0 | 0 |
| 495.202493 | 495.202442 | 9745612 | 28 | 32 | 0 | 8 | 0 | 0 |
| 495.205987 | 495.205812 | 3801804 | 25 | 36 | 0 | 8 | 1 | 0 |
| 495.21777 | 495.217698 | 2216652 | 32 | 32 | 0 | 5 | 0 | 0 |
| 495.223676 | 495.223571 | 4458700 | 25 | 36 | 0 | 10 | 0 | 0 |
| 495.23885 | 495.238827 | 10938573 | 29 | 36 | 0 | 7 | 0 | 0 |
| 495.254029 | 495.254083 | 1665485 | 33 | 36 | 0 | 4 | 0 | 0 |
| 495.25997 | 495.259956 | 2698701 | 26 | 40 | 0 | 9 | 0 | 0 |
| 495.275255 | 495.275213 | 6982862 | 30 | 40 | 0 | 6 | 0 | 0 |
| 495.31155 | 495.311598 | 2161102 | 31 | 44 | 0 | 5 | 0 | 0 |
| 497.088072 | 497.087806 | 1807087 | 28 | 18 | 0 | 9 | 0 | 0 |
| 497.109183 | 497.108935 | 2628335 | 25 | 22 | 0 | 11 | 0 | 0 |
| 497.112421 | 497.112306 | 2401519 | 22 | 26 | 0 | 11 | 1 | 0 |
| 497.124265 | 497.124191 | 2678511 | 29 | 22 | 0 | 8 | 0 | 0 |
| 497.145081 | 497.145321 | 3884784 | 26 | 26 | 0 | 10 | 0 | 0 |
| 497.148742 | 497.148691 | 4365552 | 23 | 30 | 0 | 10 | 1 | 0 |
| 497.160566 | 497.160577 | 2853360 | 30 | 26 | 0 | 7 | 0 | 0 |
| 497.18165 | 497.181706 | 7211761 | 27 | 30 | 0 | 9 | 0 | 0 |
| 497.18517 | 497.185077 | 5586161 | 24 | 34 | 0 | 9 | 1 | 0 |
| 497.197126 | 497.196962 | 2637809 | 31 | 30 | 0 | 6 | 0 | 0 |
| 497.218114 | 497.218092 | 12224753 | 28 | 34 | 0 | 8 | 0 | 0 |
| 497.221504 | 497.221462 | 3656689 | 25 | 38 | 0 | 8 | 1 | 0 |
| 497.23353 | 497.233348 | 2495729 | 32 | 34 | 0 | 5 | 0 | 0 |
| 497.239031 | 497.239221 | 1695921 | 25 | 38 | 0 | 10 | 0 | 0 |
| 497.254462 | 497.254477 | 12598513 | 29 | 38 | 0 | 7 | 0 | 0 |
| 497.290854 | 497.290863 | 7083762 | 30 | 42 | 0 | 6 | 0 | 0 |
| 497.327192 | 497.327248 | 1635059 | 31 | 46 | 0 | 5 | 0 | 0 |
| 499.06704 | 499.06707 | 1484042 | 27 | 16 | 0 | 10 | 0 | 0 |
| 499.103608 | 499.103456 | 2113547 | 28 | 20 | 0 | 9 | 0 | 0 |
| 499.124502 | 499.124585 | 2521099 | 25 | 24 | 0 | 11 | 0 | 0 |
| 499.128019 | 499.127956 | 2577419 | 22 | 28 | 0 | 11 | 1 | 0 |
| 499.139914 | 499.139841 | 3134219 | 29 | 24 | 0 | 8 | 0 | 0 |
| 499.161022 | 499.160971 | 4498443 | 26 | 28 | 0 | 10 | 0 | 0 |
| 499.164454 | 499.164341 | 4969739 | 23 | 32 | 0 | 10 | 1 | 0 |
| 499.176387 | 499.176227 | 3236619 | 30 | 28 | 0 | 7 | 0 | 0 |
| 499.197333 | 499.197356 | 10323211 | 27 | 32 | 0 | 9 | 0 | 0 |
| 499.200758 | 499.200727 | 4937996 | 24 | 36 | 0 | 9 | 1 | 0 |
| 499.212723 | 499.212612 | 3750156 | 31 | 32 | 0 | 6 | 0 | 0 |
| 499.233721 | 499.233742 | 13653260 | 28 | 36 | 0 | 8 | 0 | 0 |
| 499.237197 | 499.237112 | 2306060 | 25 | 40 | 0 | 8 | 1 | 0 |
| 499.248666 | 499.248998 | 2674444 | 32 | 36 | 0 | 5 | 0 | 0 |
| 499.270112 | 499.270127 | 12840204 | 29 | 40 | 0 | 7 | 0 | 0 |
| 499.285607 | 499.285383 | 1434893 | 33 | 40 | 0 | 4 | 0 | 0 |
| 499.30653 | 499.306513 | 7305485 | 30 | 44 | 0 | 6 | 0 | 0 |
| 501.082889 | 501.08272 | 1786140 | 27 | 18 | 0 | 10 | 0 | 0 |
| 501.119106 | 501.119106 | 3295005 | 28 | 22 | 0 | 9 | 0 | 0 |
| 501.140379 | 501.140235 | 2448669 | 25 | 26 | 0 | 11 | 0 | 0 |
| 501.143675 | 501.143606 | 3213085 | 22 | 30 | 0 | 11 | 1 | 0 |
| 501.155603 | 501.155491 | 3532061 | 29 | 26 | 0 | 8 | 0 | 0 |
| 501.176572 | 501.176621 | 6382877 | 26 | 30 | 0 | 10 | 0 | 0 |
| 501.179957 | 501.179992 | 4100893 | 23 | 34 | 0 | 10 | 1 | 0 |
| 501.191958 | 501.191877 | 4194589 | 30 | 30 | 0 | 7 | 0 | 0 |
| 501.213018 | 501.213006 | 11881757 | 27 | 34 | 0 | 9 | 0 | 0 |
| 501.216505 | 501.216377 | 2752797 | 24 | 38 | 0 | 9 | 1 | 0 |
| 501.228276 | 501.228262 | 4142365 | 31 | 34 | 0 | 6 | 0 | 0 |
| 501.249454 | 501.249392 | 15512861 | 28 | 38 | 0 | 8 | 0 | 0 |
| 501.264665 | 501.264648 | 2879518 | 32 | 38 | 0 | 5 | 0 | 0 |
| 501.285806 | 501.285777 | 12549406 | 29 | 42 | 0 | 7 | 0 | 0 |
| 501.300889 | 501.301033 | 1432094 | 33 | 42 | 0 | 4 | 0 | 0 |
| 501.322229 | 501.322163 | 6939934 | 30 | 46 | 0 | 6 | 0 | 0 |
| 503.098598 | 503.09837 | 2314021 | 27 | 20 | 0 | 10 | 0 | 0 |
| 503.119525 | 503.1195 | 1561253 | 24 | 24 | 0 | 12 | 0 | 0 |
| 503.134566 | 503.134756 | 3351334 | 28 | 24 | 0 | 9 | 0 | 0 |
| 503.138183 | 503.138127 | 1927974 | 25 | 28 | 0 | 9 | 1 | 0 |
| 503.150411 | 503.150012 | 1453606 | 32 | 24 | 0 | 6 | 0 | 0 |
| 503.155319 | 503.155233 | 3005222 | 17 | 32 | 2 | 13 | 1 | 0 |
| 503.159286 | 503.159256 | 2710566 | 22 | 32 | 0 | 11 | 1 | 0 |
| 503.171077 | 503.171141 | 4588838 | 29 | 28 | 0 | 8 | 0 | 0 |
| 503.174876 | 503.174512 | 1391654 | 26 | 32 | 0 | 8 | 1 | 0 |
| 503.192229 | 503.192271 | 8585510 | 26 | 32 | 0 | 10 | 0 | 0 |
| 503.195762 | 503.195642 | 2595110 | 23 | 36 | 0 | 10 | 1 | 0 |
| 503.207507 | 503.207527 | 4969766 | 30 | 32 | 0 | 7 | 0 | 0 |
| 503.22858 | 503.228656 | 12690726 | 27 | 36 | 0 | 9 | 0 | 0 |
| 503.243908 | 503.243912 | 4416294 | 31 | 36 | 0 | 6 | 0 | 0 |
| 503.26504 | 503.265042 | 11455782 | 28 | 40 | 0 | 8 | 0 | 0 |
| 503.280054 | 503.280298 | 2375334 | 32 | 40 | 0 | 5 | 0 | 0 |
| 503.301453 | 503.301427 | 7984934 | 29 | 44 | 0 | 7 | 0 | 0 |
| 503.337776 | 503.337813 | 5309222 | 30 | 48 | 0 | 6 | 0 | 0 |
| 505.077723 | 505.077635 | 2117414 | 26 | 18 | 0 | 11 | 0 | 0 |
| 505.114 | 505.11402 | 3427622 | 27 | 22 | 0 | 10 | 0 | 0 |
| 505.15055 | 505.150406 | 4145446 | 28 | 26 | 0 | 9 | 0 | 0 |
| 505.15343 | 505.153777 | 2022182 | 25 | 30 | 0 | 9 | 1 | 0 |
| 505.165564 | 505.165662 | 1483302 | 32 | 26 | 0 | 6 | 0 | 0 |
| 505.171332 | 505.171535 | 3842342 | 25 | 30 | 0 | 11 | 0 | 0 |
| 505.175097 | 505.174906 | 1481510 | 22 | 34 | 0 | 11 | 1 | 0 |
| 505.186659 | 505.186791 | 5614886 | 29 | 30 | 0 | 8 | 0 | 0 |
| 505.190299 | 505.190162 | 1927206 | 26 | 34 | 0 | 8 | 1 | 0 |
| 505.20786 | 505.207921 | 8996134 | 26 | 34 | 0 | 10 | 0 | 0 |
| 505.223084 | 505.223177 | 6181670 | 30 | 34 | 0 | 7 | 0 | 0 |
| 505.244291 | 505.244306 | 9531174 | 27 | 38 | 0 | 9 | 0 | 0 |
| 505.259613 | 505.259562 | 4807974 | 31 | 38 | 0 | 6 | 0 | 0 |
| 505.28076 | 505.280692 | 7762214 | 28 | 42 | 0 | 8 | 0 | 0 |
| 505.295974 | 505.295948 | 2365222 | 32 | 42 | 0 | 5 | 0 | 0 |
| 505.317137 | 505.317077 | 3460902 | 29 | 46 | 0 | 7 | 0 | 0 |
| 507.093429 | 507.093285 | 2145566 | 26 | 20 | 0 | 11 | 0 | 0 |
| 507.108221 | 507.108541 | 2031902 | 30 | 20 | 0 | 8 | 0 | 0 |
| 507.129788 | 507.129671 | 2998558 | 27 | 24 | 0 | 10 | 0 | 0 |
| 507.133209 | 507.133041 | 2073118 | 24 | 28 | 0 | 10 | 1 | 0 |
| 507.144839 | 507.144927 | 1449758 | 31 | 24 | 0 | 7 | 0 | 0 |
| 507.150388 | 507.1508 | 1496606 | 24 | 28 | 0 | 12 | 0 | 0 |
| 507.165548 | 507.165404 | 4004126 | 20 | 32 | 2 | 11 | 1 | 0 |
| 507.169356 | 507.169427 | 3363358 | 25 | 32 | 0 | 9 | 1 | 0 |
| 507.181483 | 507.181312 | 1687838 | 32 | 28 | 0 | 6 | 0 | 0 |
| 507.187299 | 507.187185 | 4313374 | 25 | 32 | 0 | 11 | 0 | 0 |
| 507.202523 | 507.202442 | 6746398 | 29 | 32 | 0 | 8 | 0 | 0 |
| 507.205969 | 507.205812 | 2717470 | 26 | 36 | 0 | 8 | 1 | 0 |
| 507.223567 | 507.223571 | 6997278 | 26 | 36 | 0 | 10 | 0 | 0 |
| 507.238806 | 507.238827 | 6373662 | 30 | 36 | 0 | 7 | 0 | 0 |
| 507.259911 | 507.259956 | 5608734 | 27 | 40 | 0 | 9 | 0 | 0 |
| 507.275197 | 507.275213 | 4284702 | 31 | 40 | 0 | 6 | 0 | 0 |
| 507.296279 | 507.296342 | 3312413 | 28 | 44 | 0 | 8 | 0 | 0 |
| 507.311414 | 507.311598 | 1620509 | 32 | 44 | 0 | 5 | 0 | 0 |
| 509.087722 | 509.087806 | 1867023 | 29 | 18 | 0 | 9 | 0 | 0 |
| 509.108871 | 509.108935 | 2112015 | 26 | 22 | 0 | 11 | 0 | 0 |
| 509.112406 | 509.112306 | 1448975 | 23 | 26 | 0 | 11 | 1 | 0 |
| 509.124331 | 509.124191 | 1902351 | 30 | 22 | 0 | 8 | 0 | 0 |
| 509.14521 | 509.145321 | 3836175 | 27 | 26 | 0 | 10 | 0 | 0 |
| 509.148733 | 509.148691 | 3567887 | 24 | 30 | 0 | 10 | 1 | 0 |
| 509.160894 | 509.160577 | 1995535 | 31 | 26 | 0 | 7 | 0 | 0 |
| 509.166349 | 509.16645 | 1724687 | 24 | 30 | 0 | 12 | 0 | 0 |
| 509.18171 | 509.181706 | 6184207 | 28 | 30 | 0 | 9 | 0 | 0 |
| 509.185168 | 509.185077 | 4122383 | 25 | 34 | 0 | 9 | 1 | 0 |
| 509.197051 | 509.196962 | 2345486 | 32 | 30 | 0 | 6 | 0 | 0 |
| 509.202707 | 509.202835 | 3438350 | 25 | 34 | 0 | 11 | 0 | 0 |
| 509.21803 | 509.218092 | 8514830 | 29 | 34 | 0 | 8 | 0 | 0 |
| 509.221427 | 509.221462 | 2640910 | 26 | 38 | 0 | 8 | 1 | 0 |
| 509.233006 | 509.233348 | 1706510 | 33 | 34 | 0 | 5 | 0 | 0 |
| 509.239235 | 509.239221 | 3913998 | 26 | 38 | 0 | 10 | 0 | 0 |
| 509.25448 | 509.254477 | 7083278 | 30 | 38 | 0 | 7 | 0 | 0 |
| 509.27563 | 509.275606 | 2836238 | 27 | 42 | 0 | 9 | 0 | 0 |
| 509.290952 | 509.290863 | 3823374 | 31 | 42 | 0 | 6 | 0 | 0 |
| 509.327149 | 509.327248 | 1724685 | 32 | 46 | 0 | 5 | 0 | 0 |
| 511.087995 | 511.0882 | 1775097 | 25 | 20 | 0 | 12 | 0 | 0 |
| 511.103509 | 511.103456 | 1720569 | 29 | 20 | 0 | 9 | 0 | 0 |
| 511.12458 | 511.124585 | 2516217 | 26 | 24 | 0 | 11 | 0 | 0 |
| 511.127583 | 511.127956 | 1809401 | 23 | 28 | 0 | 11 | 1 | 0 |
| 511.139679 | 511.139841 | 3009785 | 30 | 24 | 0 | 8 | 0 | 0 |
| 511.160977 | 511.160971 | 3955960 | 27 | 28 | 0 | 10 | 0 | 0 |
| 511.164351 | 511.164341 | 5132537 | 24 | 32 | 0 | 10 | 1 | 0 |
| 511.176319 | 511.176227 | 1953528 | 31 | 28 | 0 | 7 | 0 | 0 |
| 511.197325 | 511.197356 | 7546616 | 28 | 32 | 0 | 9 | 0 | 0 |
| 511.200798 | 511.200727 | 5754616 | 25 | 36 | 0 | 9 | 1 | 0 |
| 511.212597 | 511.212612 | 2520312 | 32 | 32 | 0 | 6 | 0 | 0 |
| 511.233611 | 511.233742 | 9820919 | 29 | 36 | 0 | 8 | 0 | 0 |
| 511.237262 | 511.237112 | 2039031 | 26 | 40 | 0 | 8 | 1 | 0 |
| 511.249147 | 511.248998 | 2084343 | 33 | 36 | 0 | 5 | 0 | 0 |
| 511.255048 | 511.254871 | 1759223 | 26 | 40 | 0 | 10 | 0 | 0 |
| 511.270073 | 511.270127 | 7986423 | 30 | 40 | 0 | 7 | 0 | 0 |
| 511.285186 | 511.285383 | 1526007 | 34 | 40 | 0 | 4 | 0 | 0 |
| 511.306487 | 511.306513 | 2842359 | 31 | 44 | 0 | 6 | 0 | 0 |
| 513.082852 | 513.08272 | 1608413 | 28 | 18 | 0 | 10 | 0 | 0 |
| 513.103835 | 513.10385 | 1565404 | 25 | 22 | 0 | 12 | 0 | 0 |
| 513.119204 | 513.119106 | 2514652 | 29 | 22 | 0 | 9 | 0 | 0 |
| 513.140186 | 513.140235 | 2800092 | 26 | 26 | 0 | 11 | 0 | 0 |
| 513.143472 | 513.143606 | 3015132 | 23 | 30 | 0 | 11 | 1 | 0 |
| 513.155234 | 513.155491 | 2584284 | 30 | 26 | 0 | 8 | 0 | 0 |
| 513.17657 | 513.176621 | 5923035 | 27 | 30 | 0 | 10 | 0 | 0 |
| 513.179972 | 513.179992 | 6168283 | 24 | 34 | 0 | 10 | 1 | 0 |
| 513.191841 | 513.191877 | 2851035 | 31 | 30 | 0 | 7 | 0 | 0 |
| 513.21302 | 513.213006 | 9765083 | 28 | 34 | 0 | 9 | 0 | 0 |
| 513.216478 | 513.216377 | 4836571 | 25 | 38 | 0 | 9 | 1 | 0 |
| 513.228251 | 513.228262 | 2880986 | 32 | 34 | 0 | 6 | 0 | 0 |
| 513.249376 | 513.249392 | 12174554 | 29 | 38 | 0 | 8 | 0 | 0 |
| 513.264541 | 513.264648 | 2347226 | 33 | 38 | 0 | 5 | 0 | 0 |
| 513.28585 | 513.285777 | 8520921 | 30 | 42 | 0 | 7 | 0 | 0 |
| 513.322248 | 513.322163 | 1937625 | 31 | 46 | 0 | 6 | 0 | 0 |
| 515.098339 | 515.09837 | 2627770 | 28 | 20 | 0 | 10 | 0 | 0 |
| 515.123158 | 515.122871 | 1404345 | 22 | 28 | 0 | 12 | 1 | 0 |
| 515.134801 | 515.134756 | 2799033 | 29 | 24 | 0 | 9 | 0 | 0 |
| 515.155843 | 515.155885 | 3068089 | 26 | 28 | 0 | 11 | 0 | 0 |
| 515.159302 | 515.159256 | 3166904 | 23 | 32 | 0 | 11 | 1 | 0 |
| 515.171061 | 515.171141 | 3018936 | 30 | 28 | 0 | 8 | 0 | 0 |
| 515.192173 | 515.192271 | 7267000 | 27 | 32 | 0 | 10 | 0 | 0 |
| 515.195738 | 515.195642 | 4073144 | 24 | 36 | 0 | 10 | 1 | 0 |
| 515.20734 | 515.207527 | 3307960 | 31 | 32 | 0 | 7 | 0 | 0 |
| 515.228676 | 515.228656 | 11461815 | 28 | 36 | 0 | 9 | 0 | 0 |
| 515.232027 | 515.232027 | 2288567 | 25 | 40 | 0 | 9 | 1 | 0 |
| 515.244008 | 515.243912 | 3457207 | 32 | 36 | 0 | 6 | 0 | 0 |
| 515.26495 | 515.265042 | 13174966 | 29 | 40 | 0 | 8 | 0 | 0 |
| 515.26831 | 515.268413 | 3656374 | 26 | 44 | 0 | 8 | 1 | 0 |
| 515.280155 | 515.280298 | 2398390 | 33 | 40 | 0 | 5 | 0 | 0 |
| 515.301454 | 515.301427 | 9332918 | 30 | 44 | 0 | 7 | 0 | 0 |
| 515.337665 | 515.337813 | 1621301 | 31 | 48 | 0 | 6 | 0 | 0 |
| 517.114006 | 517.11402 | 2805905 | 28 | 22 | 0 | 10 | 0 | 0 |
| 517.135212 | 517.13515 | 1478992 | 25 | 26 | 0 | 12 | 0 | 0 |
| 517.138641 | 517.138521 | 2070672 | 22 | 30 | 0 | 12 | 1 | 0 |
| 517.150349 | 517.150406 | 3398800 | 29 | 26 | 0 | 9 | 0 | 0 |
| 517.171489 | 517.171535 | 4265616 | 26 | 30 | 0 | 11 | 0 | 0 |
| 517.174906 | 517.174906 | 2467471 | 23 | 34 | 0 | 11 | 1 | 0 |
| 517.186875 | 517.186791 | 3740047 | 30 | 30 | 0 | 8 | 0 | 0 |
| 517.207922 | 517.207921 | 9013391 | 27 | 34 | 0 | 10 | 0 | 0 |
| 517.211446 | 517.211292 | 2792591 | 24 | 38 | 0 | 10 | 1 | 0 |
| 517.223291 | 517.223177 | 3611790 | 31 | 34 | 0 | 7 | 0 | 0 |
| 517.244255 | 517.244306 | 12112014 | 28 | 38 | 0 | 9 | 0 | 0 |
| 517.259488 | 517.259562 | 3033230 | 32 | 38 | 0 | 6 | 0 | 0 |
| 517.28054 | 517.280692 | 10235021 | 29 | 42 | 0 | 8 | 0 | 0 |
| 517.284019 | 517.284063 | 17396876 | 26 | 46 | 0 | 8 | 1 | 0 |
| 517.295211 | 517.295296 | 1858189 | 25 | 46 | 2 | 7 | 1 | 0 |
| 517.317074 | 517.317077 | 9245836 | 30 | 46 | 0 | 7 | 0 | 0 |
| 519.093499 | 519.093285 | 1729636 | 27 | 20 | 0 | 11 | 0 | 0 |
| 519.129622 | 519.129671 | 2913891 | 28 | 24 | 0 | 10 | 0 | 0 |
| 519.145327 | 519.144927 | 1411426 | 32 | 24 | 0 | 7 | 0 | 0 |
| 519.150676 | 519.1508 | 2096482 | 25 | 28 | 0 | 12 | 0 | 0 |
| 519.165811 | 519.166056 | 3555554 | 29 | 28 | 0 | 9 | 0 | 0 |
| 519.16933 | 519.169427 | 2234978 | 26 | 32 | 0 | 9 | 1 | 0 |
| 519.186942 | 519.187185 | 4985953 | 26 | 32 | 0 | 11 | 0 | 0 |
| 519.202466 | 519.202442 | 4544097 | 30 | 32 | 0 | 8 | 0 | 0 |
| 519.205936 | 519.205812 | 2123361 | 27 | 36 | 0 | 8 | 1 | 0 |
| 519.223581 | 519.223571 | 8852064 | 27 | 36 | 0 | 10 | 0 | 0 |
| 519.238696 | 519.238827 | 4336224 | 31 | 36 | 0 | 7 | 0 | 0 |
| 519.24163 | 519.242015 | 1588832 | 20 | 36 | 6 | 10 | 0 | 0 |
| 519.25995 | 519.259956 | 9619551 | 28 | 40 | 0 | 9 | 0 | 0 |
| 519.275231 | 519.275213 | 3341919 | 32 | 40 | 0 | 6 | 0 | 0 |
| 519.296254 | 519.296342 | 6318687 | 29 | 44 | 0 | 8 | 0 | 0 |
| 519.31161 | 519.311598 | 1720414 | 33 | 44 | 0 | 5 | 0 | 0 |
| 519.332778 | 519.332727 | 5485662 | 30 | 48 | 0 | 7 | 0 | 0 |
| 521.108844 | 521.108935 | 2524977 | 27 | 22 | 0 | 11 | 0 | 0 |
| 521.12399 | 521.124191 | 1905456 | 31 | 22 | 0 | 8 | 0 | 0 |
| 521.145209 | 521.145321 | 3292976 | 28 | 26 | 0 | 10 | 0 | 0 |
| 521.148583 | 521.148691 | 2900272 | 25 | 30 | 0 | 10 | 1 | 0 |
| 521.16002 | 521.159925 | 1862447 | 24 | 30 | 2 | 9 | 1 | 0 |
| 521.166323 | 521.16645 | 2246191 | 25 | 30 | 0 | 12 | 0 | 0 |
| 521.181781 | 521.181706 | 4228911 | 29 | 30 | 0 | 9 | 0 | 0 |
| 521.185151 | 521.185077 | 3074095 | 26 | 34 | 0 | 9 | 1 | 0 |
| 521.196526 | 521.196962 | 1399086 | 33 | 30 | 0 | 6 | 0 | 0 |
| 521.202816 | 521.202835 | 4709934 | 26 | 34 | 0 | 11 | 0 | 0 |
| 521.218063 | 521.218092 | 5568558 | 30 | 34 | 0 | 8 | 0 | 0 |
| 521.2215 | 521.221462 | 2806574 | 27 | 38 | 0 | 8 | 1 | 0 |
| 521.239165 | 521.239221 | 6551598 | 27 | 38 | 0 | 10 | 0 | 0 |
| 521.254417 | 521.254477 | 4641837 | 31 | 38 | 0 | 7 | 0 | 0 |
| 521.275591 | 521.275606 | 5394477 | 28 | 42 | 0 | 9 | 0 | 0 |
| 521.290919 | 521.290863 | 2477612 | 32 | 42 | 0 | 6 | 0 | 0 |
| 521.312054 | 521.311992 | 3046443 | 29 | 46 | 0 | 8 | 0 | 0 |
| 523.103418 | 523.103456 | 1728250 | 30 | 20 | 0 | 9 | 0 | 0 |
| 523.124639 | 523.124585 | 2620922 | 27 | 24 | 0 | 11 | 0 | 0 |
| 523.139745 | 523.139841 | 1754873 | 31 | 24 | 0 | 8 | 0 | 0 |
| 523.160941 | 523.160971 | 3970553 | 28 | 28 | 0 | 10 | 0 | 0 |
| 523.164493 | 523.164341 | 4062713 | 25 | 32 | 0 | 10 | 1 | 0 |
| 523.176255 | 523.176227 | 1830392 | 32 | 28 | 0 | 7 | 0 | 0 |
| 523.182155 | 523.1821 | 2001656 | 25 | 32 | 0 | 12 | 0 | 0 |
| 523.197298 | 523.197356 | 5743608 | 29 | 32 | 0 | 9 | 0 | 0 |
| 523.200769 | 523.200727 | 4488696 | 26 | 36 | 0 | 9 | 1 | 0 |
| 523.218703 | 523.218486 | 3367415 | 26 | 36 | 0 | 11 | 0 | 0 |
| 523.233744 | 523.233742 | 6703095 | 30 | 36 | 0 | 8 | 0 | 0 |
| 523.237268 | 523.237112 | 2695159 | 27 | 40 | 0 | 8 | 1 | 0 |
| 523.254822 | 523.254871 | 3546614 | 27 | 40 | 0 | 10 | 0 | 0 |
| 523.270075 | 523.270127 | 4877814 | 31 | 40 | 0 | 7 | 0 | 0 |
| 523.291428 | 523.291257 | 2598389 | 28 | 44 | 0 | 9 | 0 | 0 |
| 523.306458 | 523.306513 | 2415861 | 32 | 44 | 0 | 6 | 0 | 0 |
| 525.082552 | 525.08272 | 1531585 | 29 | 18 | 0 | 10 | 0 | 0 |
| 525.103599 | 525.10385 | 1509568 | 26 | 22 | 0 | 12 | 0 | 0 |
| 525.118639 | 525.119106 | 1528256 | 30 | 22 | 0 | 9 | 0 | 0 |
| 525.140228 | 525.140235 | 2506175 | 27 | 26 | 0 | 11 | 0 | 0 |
| 525.143668 | 525.143606 | 2587583 | 24 | 30 | 0 | 11 | 1 | 0 |
| 525.155588 | 525.155491 | 2143678 | 31 | 26 | 0 | 8 | 0 | 0 |
| 525.176394 | 525.176621 | 5003198 | 28 | 30 | 0 | 10 | 0 | 0 |
| 525.180011 | 525.179992 | 4690110 | 25 | 34 | 0 | 10 | 1 | 0 |
| 525.192057 | 525.191877 | 2149565 | 32 | 30 | 0 | 7 | 0 | 0 |
| 525.197699 | 525.19775 | 1599677 | 25 | 34 | 0 | 12 | 0 | 0 |
| 525.213038 | 525.213006 | 7646141 | 29 | 34 | 0 | 9 | 0 | 0 |
| 525.216386 | 525.216377 | 4351421 | 26 | 38 | 0 | 9 | 1 | 0 |
| 525.228422 | 525.228262 | 1911740 | 33 | 34 | 0 | 6 | 0 | 0 |
| 525.234168 | 525.234136 | 1940668 | 26 | 38 | 0 | 11 | 0 | 0 |
| 525.249374 | 525.249392 | 7920572 | 30 | 38 | 0 | 8 | 0 | 0 |
| 525.252875 | 525.252763 | 2260924 | 27 | 42 | 0 | 8 | 1 | 0 |
| 525.26459 | 525.264648 | 1431227 | 34 | 38 | 0 | 5 | 0 | 0 |
| 525.285846 | 525.285777 | 3900347 | 31 | 42 | 0 | 7 | 0 | 0 |
| 525.321883 | 525.322163 | 1905209 | 32 | 46 | 0 | 6 | 0 | 0 |
| 527.098296 | 527.09837 | 1971587 | 29 | 20 | 0 | 10 | 0 | 0 |
| 527.119329 | 527.1195 | 1597698 | 26 | 24 | 0 | 12 | 0 | 0 |
| 527.122898 | 527.122871 | 1638018 | 23 | 28 | 0 | 12 | 1 | 0 |
| 527.134868 | 527.134756 | 2049409 | 30 | 24 | 0 | 9 | 0 | 0 |
| 527.155722 | 527.155885 | 2614145 | 27 | 28 | 0 | 11 | 0 | 0 |
| 527.159328 | 527.159256 | 3354497 | 24 | 32 | 0 | 11 | 1 | 0 |
| 527.170994 | 527.171141 | 2268032 | 31 | 28 | 0 | 8 | 0 | 0 |
| 527.192319 | 527.192271 | 5380992 | 28 | 32 | 0 | 10 | 0 | 0 |
| 527.195645 | 527.195642 | 5410688 | 25 | 36 | 0 | 10 | 1 | 0 |
| 527.207603 | 527.207527 | 2316159 | 32 | 32 | 0 | 7 | 0 | 0 |
| 527.228603 | 527.228656 | 9212798 | 29 | 36 | 0 | 9 | 0 | 0 |
| 527.232224 | 527.232027 | 2581118 | 26 | 40 | 0 | 9 | 1 | 0 |
| 527.243832 | 527.243912 | 2784126 | 33 | 36 | 0 | 6 | 0 | 0 |
| 527.265025 | 527.265042 | 8927101 | 30 | 40 | 0 | 8 | 0 | 0 |
| 527.279962 | 527.280298 | 1898877 | 34 | 40 | 0 | 5 | 0 | 0 |
| 527.301219 | 527.301427 | 3271676 | 31 | 44 | 0 | 7 | 0 | 0 |
| 529.077543 | 529.077635 | 1587523 | 28 | 18 | 0 | 11 | 0 | 0 |
| 529.113692 | 529.11402 | 1547458 | 29 | 22 | 0 | 10 | 0 | 0 |
| 529.135354 | 529.13515 | 1552705 | 26 | 26 | 0 | 12 | 0 | 0 |
| 529.139053 | 529.139206 | 1489985 | 16 | 30 | 6 | 10 | 2 | 0 |
| 529.150219 | 529.150406 | 3015489 | 30 | 26 | 0 | 9 | 0 | 0 |
| 529.17147 | 529.171535 | 3660608 | 27 | 30 | 0 | 11 | 0 | 0 |
| 529.175001 | 529.174906 | 3845952 | 24 | 34 | 0 | 11 | 1 | 0 |
| 529.186736 | 529.186791 | 2684991 | 31 | 30 | 0 | 8 | 0 | 0 |
| 529.207791 | 529.207921 | 7473983 | 28 | 34 | 0 | 10 | 0 | 0 |
| 529.211361 | 529.211292 | 4431679 | 25 | 38 | 0 | 10 | 1 | 0 |
| 529.223258 | 529.223177 | 2998590 | 32 | 34 | 0 | 7 | 0 | 0 |
| 529.244308 | 529.244306 | 10609469 | 29 | 38 | 0 | 9 | 0 | 0 |
| 529.259547 | 529.259562 | 3454781 | 33 | 38 | 0 | 6 | 0 | 0 |
| 529.280583 | 529.280692 | 9043772 | 30 | 42 | 0 | 8 | 0 | 0 |
| 529.295838 | 529.295948 | 1653564 | 34 | 42 | 0 | 5 | 0 | 0 |
| 529.316963 | 529.317077 | 2924347 | 31 | 46 | 0 | 7 | 0 | 0 |
| 531.093295 | 531.093285 | 1590784 | 28 | 20 | 0 | 11 | 0 | 0 |
| 531.129675 | 531.129671 | 2547454 | 29 | 24 | 0 | 10 | 0 | 0 |
| 531.150701 | 531.1508 | 2167550 | 26 | 28 | 0 | 12 | 0 | 0 |
| 531.154239 | 531.154171 | 1411070 | 23 | 32 | 0 | 12 | 1 | 0 |
| 531.16604 | 531.166056 | 2751485 | 30 | 28 | 0 | 9 | 0 | 0 |
| 531.187172 | 531.187185 | 5022461 | 27 | 32 | 0 | 11 | 0 | 0 |
| 531.190656 | 531.190556 | 2804988 | 24 | 36 | 0 | 11 | 1 | 0 |
| 531.202456 | 531.202442 | 3299580 | 31 | 32 | 0 | 8 | 0 | 0 |
| 531.205815 | 531.205812 | 1935868 | 28 | 36 | 0 | 8 | 1 | 0 |
| 531.2236 | 531.223571 | 9528059 | 28 | 36 | 0 | 10 | 0 | 0 |
| 531.23885 | 531.238827 | 3534587 | 32 | 36 | 0 | 7 | 0 | 0 |
| 531.259889 | 531.259956 | 10905338 | 29 | 40 | 0 | 9 | 0 | 0 |
| 531.275237 | 531.275213 | 2749178 | 33 | 40 | 0 | 6 | 0 | 0 |
| 531.296345 | 531.296342 | 8556281 | 30 | 44 | 0 | 8 | 0 | 0 |
| 531.311512 | 531.311598 | 1483256 | 34 | 44 | 0 | 5 | 0 | 0 |
| 531.332596 | 531.332727 | 2344696 | 31 | 48 | 0 | 7 | 0 | 0 |
| 533.108953 | 533.108935 | 2079931 | 28 | 22 | 0 | 11 | 0 | 0 |
| 533.145253 | 533.145321 | 2819769 | 29 | 26 | 0 | 10 | 0 | 0 |
| 533.148686 | 533.148691 | 1586617 | 26 | 30 | 0 | 10 | 1 | 0 |
| 533.166517 | 533.16645 | 2153657 | 26 | 30 | 0 | 12 | 0 | 0 |
| 533.169878 | 533.169821 | 1517496 | 23 | 34 | 0 | 12 | 1 | 0 |
| 533.181703 | 533.181706 | 3221176 | 30 | 30 | 0 | 9 | 0 | 0 |
| 533.185154 | 533.185077 | 2599608 | 27 | 34 | 0 | 9 | 1 | 0 |
| 533.202688 | 533.202835 | 5646519 | 27 | 34 | 0 | 11 | 0 | 0 |
| 533.217947 | 533.218092 | 3610295 | 31 | 34 | 0 | 8 | 0 | 0 |
| 533.221492 | 533.221462 | 2599095 | 28 | 38 | 0 | 8 | 1 | 0 |
| 533.239133 | 533.239221 | 8777398 | 28 | 38 | 0 | 10 | 0 | 0 |
| 533.254482 | 533.254477 | 3982006 | 32 | 38 | 0 | 7 | 0 | 0 |
| 533.275591 | 533.275606 | 9147061 | 29 | 42 | 0 | 9 | 0 | 0 |
| 533.290912 | 533.290863 | 2765492 | 33 | 42 | 0 | 6 | 0 | 0 |
| 533.311925 | 533.311992 | 7265972 | 30 | 46 | 0 | 8 | 0 | 0 |
| 535.08768 | 535.0882 | 1763957 | 27 | 20 | 0 | 12 | 0 | 0 |
| 535.12467 | 535.124585 | 1897588 | 28 | 24 | 0 | 11 | 0 | 0 |
| 535.160746 | 535.160971 | 3391603 | 29 | 28 | 0 | 10 | 0 | 0 |
| 535.164196 | 535.164341 | 2731635 | 26 | 32 | 0 | 10 | 1 | 0 |
| 535.182169 | 535.1821 | 2500210 | 26 | 32 | 0 | 12 | 0 | 0 |
| 535.19736 | 535.197356 | 4073074 | 30 | 32 | 0 | 9 | 0 | 0 |
| 535.200808 | 535.200727 | 3959921 | 27 | 36 | 0 | 9 | 1 | 0 |
| 535.218476 | 535.218486 | 5067377 | 27 | 36 | 0 | 11 | 0 | 0 |
| 535.233663 | 535.233742 | 4789872 | 31 | 36 | 0 | 8 | 0 | 0 |
| 535.237126 | 535.237112 | 3139696 | 28 | 40 | 0 | 8 | 1 | 0 |
| 535.254816 | 535.254871 | 6042224 | 28 | 40 | 0 | 10 | 0 | 0 |
| 535.269965 | 535.270127 | 3669615 | 32 | 40 | 0 | 7 | 0 | 0 |
| 535.291185 | 535.291257 | 4649582 | 29 | 44 | 0 | 9 | 0 | 0 |
| 535.306519 | 535.306513 | 1993582 | 33 | 44 | 0 | 6 | 0 | 0 |
| 535.327517 | 535.327642 | 2830701 | 30 | 48 | 0 | 8 | 0 | 0 |
| 537.103575 | 537.10385 | 1809710 | 27 | 22 | 0 | 12 | 0 | 0 |
| 537.140276 | 537.140235 | 2227501 | 28 | 26 | 0 | 11 | 0 | 0 |
| 537.143432 | 537.143606 | 2198061 | 25 | 30 | 0 | 11 | 1 | 0 |
| 537.155525 | 537.155491 | 1497644 | 32 | 26 | 0 | 8 | 0 | 0 |
| 537.176617 | 537.176621 | 3665964 | 29 | 30 | 0 | 10 | 0 | 0 |
| 537.180024 | 537.179992 | 3639851 | 26 | 34 | 0 | 10 | 1 | 0 |
| 537.191689 | 537.191877 | 1871147 | 33 | 30 | 0 | 7 | 0 | 0 |
| 537.197887 | 537.19775 | 2160683 | 26 | 34 | 0 | 12 | 0 | 0 |
| 537.212934 | 537.213006 | 4661802 | 30 | 34 | 0 | 9 | 0 | 0 |
| 537.216436 | 537.216377 | 4298282 | 27 | 38 | 0 | 9 | 1 | 0 |
| 537.234075 | 537.234136 | 3769385 | 27 | 38 | 0 | 11 | 0 | 0 |
| 537.249325 | 537.249392 | 4484649 | 31 | 38 | 0 | 8 | 0 | 0 |
| 537.270483 | 537.270521 | 3753512 | 28 | 42 | 0 | 10 | 0 | 0 |
| 537.285375 | 537.285777 | 3255080 | 32 | 42 | 0 | 7 | 0 | 0 |
| 537.306532 | 537.306907 | 1697447 | 29 | 46 | 0 | 9 | 0 | 0 |
| 537.322423 | 537.322163 | 1721894 | 33 | 46 | 0 | 6 | 0 | 0 |
| 539.098262 | 539.09837 | 1382759 | 30 | 20 | 0 | 10 | 0 | 0 |
| 539.119235 | 539.1195 | 1596902 | 27 | 24 | 0 | 12 | 0 | 0 |
| 539.156038 | 539.155885 | 1926501 | 28 | 28 | 0 | 11 | 0 | 0 |
| 539.159184 | 539.159256 | 3403749 | 25 | 32 | 0 | 11 | 1 | 0 |
| 539.170931 | 539.171141 | 1849572 | 32 | 28 | 0 | 8 | 0 | 0 |
| 539.192289 | 539.192271 | 4036580 | 29 | 32 | 0 | 10 | 0 | 0 |
| 539.195791 | 539.195642 | 4596708 | 26 | 36 | 0 | 10 | 1 | 0 |
| 539.207317 | 539.207527 | 1587171 | 33 | 32 | 0 | 7 | 0 | 0 |
| 539.213623 | 539.2134 | 1378787 | 26 | 36 | 0 | 12 | 0 | 0 |
| 539.228588 | 539.228656 | 6049251 | 30 | 36 | 0 | 9 | 0 | 0 |
| 539.232092 | 539.232027 | 3896802 | 27 | 40 | 0 | 9 | 1 | 0 |
| 539.243864 | 539.243912 | 1780962 | 34 | 36 | 0 | 6 | 0 | 0 |
| 539.249895 | 539.249786 | 1734370 | 27 | 40 | 0 | 11 | 0 | 0 |
| 539.265007 | 539.265042 | 4690657 | 31 | 40 | 0 | 8 | 0 | 0 |
| 539.286386 | 539.286171 | 1670880 | 28 | 44 | 0 | 10 | 0 | 0 |
| 539.301462 | 539.301427 | 2811360 | 32 | 44 | 0 | 7 | 0 | 0 |
| 541.114073 | 541.11402 | 1383839 | 30 | 22 | 0 | 10 | 0 | 0 |
| 541.135105 | 541.13515 | 1596063 | 27 | 26 | 0 | 12 | 0 | 0 |
| 541.138154 | 541.138521 | 1503902 | 24 | 30 | 0 | 12 | 1 | 0 |
| 541.15044 | 541.150406 | 2042782 | 31 | 26 | 0 | 9 | 0 | 0 |
| 541.171484 | 541.171535 | 3199901 | 28 | 30 | 0 | 11 | 0 | 0 |
| 541.175029 | 541.174906 | 3914141 | 25 | 34 | 0 | 11 | 1 | 0 |
| 541.186251 | 541.186791 | 2144413 | 32 | 30 | 0 | 8 | 0 | 0 |
| 541.207683 | 541.207921 | 5282204 | 29 | 34 | 0 | 10 | 0 | 0 |
| 541.211354 | 541.211292 | 3755932 | 26 | 38 | 0 | 10 | 1 | 0 |
| 541.223219 | 541.223177 | 2303388 | 33 | 34 | 0 | 7 | 0 | 0 |
| 541.244249 | 541.244306 | 7597467 | 30 | 38 | 0 | 9 | 0 | 0 |
| 541.247708 | 541.247677 | 2338971 | 27 | 42 | 0 | 9 | 1 | 0 |
| 541.259149 | 541.259562 | 1811610 | 34 | 38 | 0 | 6 | 0 | 0 |
| 541.280577 | 541.280692 | 4414362 | 31 | 42 | 0 | 8 | 0 | 0 |
| 541.317083 | 541.317077 | 2429848 | 32 | 46 | 0 | 7 | 0 | 0 |
| 543.093255 | 543.093285 | 1604953 | 29 | 20 | 0 | 11 | 0 | 0 |
| 543.129494 | 543.129671 | 1661528 | 30 | 24 | 0 | 10 | 0 | 0 |
| 543.150095 | 543.150148 | 1786327 | 19 | 32 | 2 | 14 | 1 | 0 |
| 543.154024 | 543.154171 | 1548375 | 24 | 32 | 0 | 12 | 1 | 0 |
| 543.165924 | 543.166056 | 1561943 | 31 | 28 | 0 | 9 | 0 | 0 |
| 543.187251 | 543.187185 | 4124502 | 28 | 32 | 0 | 11 | 0 | 0 |
| 543.190538 | 543.190556 | 3637078 | 25 | 36 | 0 | 11 | 1 | 0 |
| 543.202377 | 543.202442 | 2763094 | 32 | 32 | 0 | 8 | 0 | 0 |
| 543.223516 | 543.223571 | 6839125 | 29 | 36 | 0 | 10 | 0 | 0 |
| 543.226941 | 543.226942 | 2679637 | 26 | 40 | 0 | 10 | 1 | 0 |
| 543.238821 | 543.238827 | 2438740 | 33 | 36 | 0 | 7 | 0 | 0 |
| 543.259975 | 543.259956 | 8956244 | 30 | 40 | 0 | 9 | 0 | 0 |
| 543.275241 | 543.275213 | 2183507 | 34 | 40 | 0 | 6 | 0 | 0 |
| 543.296303 | 543.296342 | 3937618 | 31 | 44 | 0 | 8 | 0 | 0 |
| 543.33257 | 543.332727 | 1600849 | 32 | 48 | 0 | 7 | 0 | 0 |
| 545.108668 | 545.108935 | 1523219 | 29 | 22 | 0 | 11 | 0 | 0 |
| 545.145312 | 545.145321 | 2069778 | 30 | 26 | 0 | 10 | 0 | 0 |
| 545.166063 | 545.16645 | 2111121 | 27 | 30 | 0 | 12 | 0 | 0 |
| 545.181767 | 545.181706 | 2954513 | 31 | 30 | 0 | 9 | 0 | 0 |
| 545.184611 | 545.185077 | 1501969 | 28 | 34 | 0 | 9 | 1 | 0 |
| 545.202927 | 545.202835 | 5363984 | 28 | 34 | 0 | 11 | 0 | 0 |
| 545.206063 | 545.206206 | 2405392 | 25 | 38 | 0 | 11 | 1 | 0 |
| 545.217688 | 545.218092 | 2934288 | 32 | 34 | 0 | 8 | 0 | 0 |
| 545.239191 | 545.239221 | 8517903 | 29 | 38 | 0 | 10 | 0 | 0 |
| 545.254348 | 545.254477 | 2602510 | 33 | 38 | 0 | 7 | 0 | 0 |
| 545.275635 | 545.275606 | 9177358 | 30 | 42 | 0 | 9 | 0 | 0 |
| 545.290945 | 545.290863 | 1891341 | 34 | 42 | 0 | 6 | 0 | 0 |
| 545.312073 | 545.311992 | 2949900 | 31 | 46 | 0 | 8 | 0 | 0 |
| 547.124794 | 547.124585 | 1870031 | 29 | 24 | 0 | 11 | 0 | 0 |
| 547.16076 | 547.160971 | 2424782 | 30 | 28 | 0 | 10 | 0 | 0 |
| 547.164241 | 547.164341 | 1906893 | 27 | 32 | 0 | 10 | 1 | 0 |
| 547.182111 | 547.1821 | 3021517 | 27 | 32 | 0 | 12 | 0 | 0 |
| 547.197373 | 547.197356 | 2802380 | 31 | 32 | 0 | 9 | 0 | 0 |
| 547.200536 | 547.200727 | 2414028 | 28 | 36 | 0 | 9 | 1 | 0 |
| 547.218374 | 547.218486 | 5691596 | 28 | 36 | 0 | 11 | 0 | 0 |
| 547.233769 | 547.233742 | 3568331 | 32 | 36 | 0 | 8 | 0 | 0 |
| 547.236926 | 547.237112 | 2267851 | 29 | 40 | 0 | 8 | 1 | 0 |
| 547.254746 | 547.254871 | 7826635 | 29 | 40 | 0 | 10 | 0 | 0 |
| 547.269971 | 547.270127 | 3307722 | 33 | 40 | 0 | 7 | 0 | 0 |
| 547.291152 | 547.291257 | 7085257 | 30 | 44 | 0 | 9 | 0 | 0 |
| 547.306171 | 547.306513 | 1400521 | 34 | 44 | 0 | 6 | 0 | 0 |
| 547.327682 | 547.327642 | 2024136 | 31 | 48 | 0 | 8 | 0 | 0 |
| 549.103728 | 549.10385 | 1921933 | 28 | 22 | 0 | 12 | 0 | 0 |
| 549.140276 | 549.140235 | 2105996 | 29 | 26 | 0 | 11 | 0 | 0 |
| 549.143448 | 549.143606 | 1689484 | 26 | 30 | 0 | 11 | 1 | 0 |
| 549.155588 | 549.155491 | 1409164 | 33 | 26 | 0 | 8 | 0 | 0 |
| 549.176676 | 549.176621 | 2644107 | 30 | 30 | 0 | 10 | 0 | 0 |
| 549.179804 | 549.179992 | 2648459 | 27 | 34 | 0 | 10 | 1 | 0 |
| 549.197587 | 549.19775 | 3339402 | 27 | 34 | 0 | 12 | 0 | 0 |
| 549.213122 | 549.213006 | 3245194 | 31 | 34 | 0 | 9 | 0 | 0 |
| 549.216302 | 549.216377 | 3668106 | 28 | 38 | 0 | 9 | 1 | 0 |
| 549.234113 | 549.234136 | 4621449 | 28 | 38 | 0 | 11 | 0 | 0 |
| 549.249242 | 549.249392 | 3900553 | 32 | 38 | 0 | 8 | 0 | 0 |
| 549.252701 | 549.252763 | 2329737 | 29 | 42 | 0 | 8 | 1 | 0 |
| 549.270433 | 549.270521 | 5529736 | 29 | 42 | 0 | 10 | 0 | 0 |
| 549.285737 | 549.285777 | 2866312 | 33 | 42 | 0 | 7 | 0 | 0 |
| 549.306793 | 549.306907 | 3904135 | 30 | 46 | 0 | 9 | 0 | 0 |
| 551.119203 | 551.1195 | 1542477 | 28 | 24 | 0 | 12 | 0 | 0 |
| 551.134667 | 551.134756 | 1664077 | 32 | 24 | 0 | 9 | 0 | 0 |
| 551.155916 | 551.155885 | 2166860 | 29 | 28 | 0 | 11 | 0 | 0 |
| 551.159151 | 551.159256 | 2564684 | 26 | 32 | 0 | 11 | 1 | 0 |
| 551.192159 | 551.192271 | 3691595 | 30 | 32 | 0 | 10 | 0 | 0 |
| 551.195645 | 551.195642 | 4878923 | 27 | 36 | 0 | 10 | 1 | 0 |
| 551.207673 | 551.207527 | 1515850 | 34 | 32 | 0 | 7 | 0 | 0 |
| 551.213376 | 551.2134 | 2651722 | 27 | 36 | 0 | 12 | 0 | 0 |
| 551.228675 | 551.228656 | 4136522 | 31 | 36 | 0 | 9 | 0 | 0 |
| 551.23202 | 551.232027 | 4416842 | 28 | 40 | 0 | 9 | 1 | 0 |
| 551.243629 | 551.243912 | 1378889 | 35 | 36 | 0 | 6 | 0 | 0 |
| 551.249828 | 551.249786 | 3603529 | 28 | 40 | 0 | 11 | 0 | 0 |
| 551.264949 | 551.265042 | 3542089 | 32 | 40 | 0 | 8 | 0 | 0 |
| 551.268099 | 551.268413 | 2356297 | 29 | 44 | 0 | 8 | 1 | 0 |
| 551.285974 | 551.286171 | 3124296 | 29 | 44 | 0 | 10 | 0 | 0 |
| 551.301423 | 551.301427 | 2606664 | 33 | 44 | 0 | 7 | 0 | 0 |
| 551.322335 | 551.322557 | 1729607 | 30 | 48 | 0 | 9 | 0 | 0 |
| 553.098884 | 553.098764 | 1398289 | 27 | 22 | 0 | 13 | 0 | 0 |
| 553.113887 | 553.11402 | 1403281 | 31 | 22 | 0 | 10 | 0 | 0 |
| 553.135126 | 553.13515 | 1446672 | 28 | 26 | 0 | 12 | 0 | 0 |
| 553.150414 | 553.150406 | 1629968 | 32 | 26 | 0 | 9 | 0 | 0 |
| 553.171369 | 553.171535 | 2410255 | 29 | 30 | 0 | 11 | 0 | 0 |
| 553.174927 | 553.174906 | 3949583 | 26 | 34 | 0 | 11 | 1 | 0 |
| 553.186766 | 553.186791 | 1641998 | 33 | 30 | 0 | 8 | 0 | 0 |
| 553.207579 | 553.207921 | 3418126 | 30 | 34 | 0 | 10 | 0 | 0 |
| 553.211294 | 553.211292 | 5047822 | 27 | 38 | 0 | 10 | 1 | 0 |
| 553.223169 | 553.223177 | 1413389 | 34 | 34 | 0 | 7 | 0 | 0 |
| 553.229373 | 553.22905 | 1457421 | 27 | 38 | 0 | 12 | 0 | 0 |
| 553.244213 | 553.244306 | 3911693 | 31 | 38 | 0 | 9 | 0 | 0 |
| 553.247532 | 553.247677 | 3531533 | 28 | 42 | 0 | 9 | 1 | 0 |
| 553.259478 | 553.259562 | 1387916 | 35 | 38 | 0 | 6 | 0 | 0 |
| 553.265165 | 553.265436 | 1918476 | 28 | 42 | 0 | 11 | 0 | 0 |
| 553.280758 | 553.280692 | 3127820 | 32 | 42 | 0 | 8 | 0 | 0 |
| 553.317084 | 553.317077 | 2053131 | 33 | 46 | 0 | 7 | 0 | 0 |
| 555.154357 | 555.154171 | 2202582 | 25 | 32 | 0 | 12 | 1 | 0 |
| 555.187031 | 555.187185 | 3180501 | 29 | 32 | 0 | 11 | 0 | 0 |
| 555.190446 | 555.190556 | 4176341 | 26 | 36 | 0 | 11 | 1 | 0 |
| 555.202488 | 555.202442 | 1982933 | 33 | 32 | 0 | 8 | 0 | 0 |
| 555.223386 | 555.223571 | 4981204 | 30 | 36 | 0 | 10 | 0 | 0 |
| 555.226974 | 555.226942 | 4564948 | 27 | 40 | 0 | 10 | 1 | 0 |
| 555.239005 | 555.238827 | 1968084 | 34 | 36 | 0 | 7 | 0 | 0 |
| 555.259681 | 555.259956 | 4781011 | 31 | 40 | 0 | 9 | 0 | 0 |
| 555.263493 | 555.263327 | 1853651 | 28 | 44 | 0 | 9 | 1 | 0 |
| 555.296318 | 555.296342 | 3274706 | 32 | 44 | 0 | 8 | 0 | 0 |
| 556.227299 | 556.227551 | 1560761 | 32 | 35 | 3 | 4 | 1 | 0 |
| 557.14525 | 557.145321 | 1874337 | 31 | 26 | 0 | 10 | 0 | 0 |
| 557.166332 | 557.16645 | 2140577 | 28 | 30 | 0 | 12 | 0 | 0 |
| 557.16987 | 557.169821 | 2558368 | 25 | 34 | 0 | 12 | 1 | 0 |
| 557.181836 | 557.181706 | 2445216 | 32 | 30 | 0 | 9 | 0 | 0 |
| 557.202793 | 557.202835 | 3861920 | 29 | 34 | 0 | 11 | 0 | 0 |
| 557.206109 | 557.206206 | 3420064 | 26 | 38 | 0 | 11 | 1 | 0 |
| 557.217739 | 557.218092 | 2326175 | 33 | 34 | 0 | 8 | 0 | 0 |
| 557.239239 | 557.239221 | 5800863 | 30 | 38 | 0 | 10 | 0 | 0 |
| 557.242633 | 557.242592 | 2218655 | 27 | 42 | 0 | 10 | 1 | 0 |
| 557.254392 | 557.254477 | 1844894 | 34 | 38 | 0 | 7 | 0 | 0 |
| 557.275455 | 557.275606 | 4268446 | 31 | 42 | 0 | 9 | 0 | 0 |
| 557.290888 | 557.290863 | 1416861 | 35 | 42 | 0 | 6 | 0 | 0 |
| 557.311795 | 557.311992 | 2515613 | 32 | 46 | 0 | 8 | 0 | 0 |
| 559.124421 | 559.124585 | 1514352 | 30 | 24 | 0 | 11 | 0 | 0 |
| 559.160962 | 559.160971 | 1487727 | 31 | 28 | 0 | 10 | 0 | 0 |
| 559.163985 | 559.164341 | 1405039 | 28 | 32 | 0 | 10 | 1 | 0 |
| 559.182092 | 559.1821 | 2865007 | 28 | 32 | 0 | 12 | 0 | 0 |
| 559.185223 | 559.185471 | 1867823 | 25 | 36 | 0 | 12 | 1 | 0 |
| 559.19742 | 559.197356 | 1817455 | 32 | 32 | 0 | 9 | 0 | 0 |
| 559.218309 | 559.218486 | 5422446 | 29 | 36 | 0 | 11 | 0 | 0 |
| 559.222113 | 559.221856 | 1811822 | 26 | 40 | 0 | 11 | 1 | 0 |
| 559.23334 | 559.233742 | 2354542 | 33 | 36 | 0 | 8 | 0 | 0 |
| 559.254746 | 559.254871 | 6975341 | 30 | 40 | 0 | 10 | 0 | 0 |
| 559.269973 | 559.270127 | 2071917 | 34 | 40 | 0 | 7 | 0 | 0 |
| 559.291152 | 559.291257 | 3772780 | 31 | 44 | 0 | 9 | 0 | 0 |
| 559.306656 | 559.306513 | 1419116 | 35 | 44 | 0 | 6 | 0 | 0 |
| 559.327604 | 559.327642 | 1889388 | 32 | 48 | 0 | 8 | 0 | 0 |
| 561.1763 | 561.176621 | 1956290 | 31 | 30 | 0 | 10 | 0 | 0 |
| 561.17988 | 561.179992 | 1893186 | 28 | 34 | 0 | 10 | 1 | 0 |
| 561.197462 | 561.19775 | 3073346 | 28 | 34 | 0 | 12 | 0 | 0 |
| 561.212987 | 561.213006 | 2266689 | 32 | 34 | 0 | 9 | 0 | 0 |
| 561.234051 | 561.234136 | 6233921 | 29 | 38 | 0 | 11 | 0 | 0 |
| 561.249297 | 561.249392 | 2641217 | 33 | 38 | 0 | 8 | 0 | 0 |
| 561.270501 | 561.270521 | 7367488 | 30 | 42 | 0 | 10 | 0 | 0 |
| 561.285882 | 561.285777 | 2215232 | 34 | 42 | 0 | 7 | 0 | 0 |
| 561.306708 | 561.306907 | 3230015 | 31 | 46 | 0 | 9 | 0 | 0 |
| 563.119521 | 563.1195 | 1733914 | 29 | 24 | 0 | 12 | 0 | 0 |
| 563.155989 | 563.155885 | 2111258 | 30 | 28 | 0 | 11 | 0 | 0 |
| 563.159146 | 563.159256 | 1917210 | 27 | 32 | 0 | 11 | 1 | 0 |
| 563.177008 | 563.177015 | 1511705 | 27 | 32 | 0 | 13 | 0 | 0 |
| 563.192179 | 563.192271 | 2414105 | 31 | 32 | 0 | 10 | 0 | 0 |
| 563.195566 | 563.195642 | 3198233 | 28 | 36 | 0 | 10 | 1 | 0 |
| 563.213268 | 563.2134 | 2909465 | 28 | 36 | 0 | 12 | 0 | 0 |
| 563.228525 | 563.228656 | 2703896 | 32 | 36 | 0 | 9 | 0 | 0 |
| 563.231965 | 563.232027 | 3371288 | 29 | 40 | 0 | 9 | 1 | 0 |
| 563.249704 | 563.249786 | 5787416 | 29 | 40 | 0 | 11 | 0 | 0 |
| 563.264924 | 563.265042 | 2830616 | 33 | 40 | 0 | 8 | 0 | 0 |
| 563.268281 | 563.268413 | 2002199 | 30 | 44 | 0 | 8 | 1 | 0 |
| 563.286142 | 563.286171 | 4990743 | 30 | 44 | 0 | 10 | 0 | 0 |
| 563.301081 | 563.301427 | 2181655 | 34 | 44 | 0 | 7 | 0 | 0 |
| 563.32219 | 563.322557 | 1451158 | 31 | 48 | 0 | 9 | 0 | 0 |
| 565.135142 | 565.13515 | 1590773 | 29 | 26 | 0 | 12 | 0 | 0 |
| 565.171137 | 565.171535 | 2023284 | 30 | 30 | 0 | 11 | 0 | 0 |
| 565.174909 | 565.174906 | 3500788 | 27 | 34 | 0 | 11 | 1 | 0 |
| 565.186496 | 565.186791 | 1440244 | 34 | 30 | 0 | 8 | 0 | 0 |
| 565.192714 | 565.192665 | 1716468 | 27 | 34 | 0 | 13 | 0 | 0 |
| 565.20779 | 565.207921 | 2983668 | 31 | 34 | 0 | 10 | 0 | 0 |
| 565.211268 | 565.211292 | 5153524 | 28 | 38 | 0 | 10 | 1 | 0 |
| 565.228785 | 565.22905 | 2422771 | 28 | 38 | 0 | 12 | 0 | 0 |
| 565.244289 | 565.244306 | 3527923 | 32 | 38 | 0 | 9 | 0 | 0 |
| 565.247715 | 565.247677 | 4232947 | 29 | 42 | 0 | 9 | 1 | 0 |
| 565.265262 | 565.265436 | 3487987 | 29 | 42 | 0 | 11 | 0 | 0 |
| 565.280685 | 565.280692 | 2898162 | 33 | 42 | 0 | 8 | 0 | 0 |
| 565.284083 | 565.284063 | 2142450 | 30 | 46 | 0 | 8 | 1 | 0 |
| 565.30175 | 565.301821 | 2380530 | 30 | 46 | 0 | 10 | 0 | 0 |
| 565.317291 | 565.317077 | 1469106 | 34 | 46 | 0 | 7 | 0 | 0 |
| 567.1146 | 567.114414 | 1370836 | 28 | 24 | 0 | 13 | 0 | 0 |
| 567.15085 | 567.1508 | 2251988 | 29 | 28 | 0 | 12 | 0 | 0 |
| 567.153404 | 567.153519 | 1616339 | 18 | 36 | 2 | 14 | 2 | 0 |
| 567.165948 | 567.166056 | 1549011 | 33 | 28 | 0 | 9 | 0 | 0 |
| 567.186846 | 567.187185 | 2245075 | 30 | 32 | 0 | 11 | 0 | 0 |
| 567.190581 | 567.190556 | 4786899 | 27 | 36 | 0 | 11 | 1 | 0 |
| 567.202359 | 567.202442 | 1430739 | 34 | 32 | 0 | 8 | 0 | 0 |
| 567.223261 | 567.223571 | 3092178 | 31 | 36 | 0 | 10 | 0 | 0 |
| 567.226904 | 567.226942 | 5462739 | 28 | 40 | 0 | 10 | 1 | 0 |
| 567.244431 | 567.2447 | 1847506 | 28 | 40 | 0 | 12 | 0 | 0 |
| 567.259894 | 567.259956 | 3794642 | 32 | 40 | 0 | 9 | 0 | 0 |
| 567.263315 | 567.263327 | 3868370 | 29 | 44 | 0 | 9 | 1 | 0 |
| 567.281142 | 567.281086 | 1460945 | 29 | 44 | 0 | 11 | 0 | 0 |
| 567.296317 | 567.296342 | 2554577 | 33 | 44 | 0 | 8 | 0 | 0 |
| 569.166025 | 569.16645 | 1800118 | 29 | 30 | 0 | 12 | 0 | 0 |
| 569.169909 | 569.169821 | 2139318 | 26 | 34 | 0 | 12 | 1 | 0 |
| 569.18151 | 569.181706 | 1498294 | 33 | 30 | 0 | 9 | 0 | 0 |
| 569.202952 | 569.202835 | 2469045 | 30 | 34 | 0 | 11 | 0 | 0 |
| 569.206173 | 569.206206 | 5357238 | 27 | 38 | 0 | 11 | 1 | 0 |
| 569.218068 | 569.218092 | 1500341 | 34 | 34 | 0 | 8 | 0 | 0 |
| 569.239044 | 569.239221 | 3302837 | 31 | 38 | 0 | 10 | 0 | 0 |
| 569.242582 | 569.242592 | 3651253 | 28 | 42 | 0 | 10 | 1 | 0 |
| 569.2542 | 569.254477 | 1916853 | 35 | 38 | 0 | 7 | 0 | 0 |
| 569.275532 | 569.275606 | 3979444 | 32 | 42 | 0 | 9 | 0 | 0 |
| 569.312198 | 569.311992 | 2480820 | 33 | 46 | 0 | 8 | 0 | 0 |
| 571.124621 | 571.124585 | 1471901 | 31 | 24 | 0 | 11 | 0 | 0 |
| 571.160951 | 571.160971 | 1698972 | 32 | 28 | 0 | 10 | 0 | 0 |
| 571.181623 | 571.1821 | 2096028 | 29 | 32 | 0 | 12 | 0 | 0 |
| 571.18542 | 571.185471 | 2539676 | 26 | 36 | 0 | 12 | 1 | 0 |
| 571.197334 | 571.197356 | 1876636 | 33 | 32 | 0 | 9 | 0 | 0 |
| 571.218397 | 571.218486 | 3700380 | 30 | 36 | 0 | 11 | 0 | 0 |
| 571.221746 | 571.221856 | 3384476 | 27 | 40 | 0 | 11 | 1 | 0 |
| 571.233736 | 571.233742 | 1538203 | 34 | 36 | 0 | 8 | 0 | 0 |
| 571.254691 | 571.254871 | 3920539 | 31 | 40 | 0 | 10 | 0 | 0 |
| 571.269289 | 571.269475 | 1688987 | 27 | 44 | 2 | 9 | 1 | 0 |
| 571.291065 | 571.291257 | 3368091 | 32 | 44 | 0 | 9 | 0 | 0 |
| 571.32774 | 571.327642 | 1970842 | 33 | 48 | 0 | 8 | 0 | 0 |
| 573.139881 | 573.140235 | 1580166 | 31 | 26 | 0 | 11 | 0 | 0 |
| 573.161536 | 573.161365 | 1474182 | 28 | 30 | 0 | 13 | 0 | 0 |
| 573.176444 | 573.176621 | 1681030 | 32 | 30 | 0 | 10 | 0 | 0 |
| 573.197579 | 573.19775 | 2810758 | 29 | 34 | 0 | 12 | 0 | 0 |
| 573.201073 | 573.201121 | 2681990 | 26 | 38 | 0 | 12 | 1 | 0 |
| 573.213067 | 573.213006 | 2086534 | 33 | 34 | 0 | 9 | 0 | 0 |
| 573.234081 | 573.234136 | 4730502 | 30 | 38 | 0 | 11 | 0 | 0 |
| 573.249269 | 573.249392 | 2025093 | 34 | 38 | 0 | 8 | 0 | 0 |
| 573.270462 | 573.270521 | 3550341 | 31 | 42 | 0 | 10 | 0 | 0 |
| 573.285763 | 573.285777 | 1585029 | 35 | 42 | 0 | 7 | 0 | 0 |
| 573.306674 | 573.306907 | 2686597 | 32 | 46 | 0 | 9 | 0 | 0 |
| 575.119245 | 575.1195 | 1395316 | 30 | 24 | 0 | 12 | 0 | 0 |
| 575.155341 | 575.155885 | 1413236 | 31 | 28 | 0 | 11 | 0 | 0 |
| 575.195636 | 575.195642 | 1700723 | 29 | 36 | 0 | 10 | 1 | 0 |
| 575.212924 | 575.2134 | 3377779 | 29 | 36 | 0 | 12 | 0 | 0 |
| 575.228565 | 575.228656 | 2567283 | 33 | 36 | 0 | 9 | 0 | 0 |
| 575.24967 | 575.249786 | 5169267 | 30 | 40 | 0 | 11 | 0 | 0 |
| 575.264781 | 575.265042 | 2591347 | 34 | 40 | 0 | 8 | 0 | 0 |
| 575.286115 | 575.286171 | 3831411 | 31 | 44 | 0 | 10 | 0 | 0 |
| 575.300891 | 575.301427 | 1527411 | 35 | 44 | 0 | 7 | 0 | 0 |
| 575.322027 | 575.322557 | 1756274 | 32 | 48 | 0 | 9 | 0 | 0 |
| 577.135118 | 577.13515 | 1686117 | 30 | 26 | 0 | 12 | 0 | 0 |
| 577.174937 | 577.174906 | 1694820 | 28 | 34 | 0 | 11 | 1 | 0 |
| 577.207609 | 577.207921 | 2275428 | 32 | 34 | 0 | 10 | 0 | 0 |
| 577.211044 | 577.211292 | 2989156 | 29 | 38 | 0 | 10 | 1 | 0 |
| 577.228982 | 577.22905 | 3392612 | 29 | 38 | 0 | 12 | 0 | 0 |
| 577.244011 | 577.244306 | 2613604 | 33 | 38 | 0 | 9 | 0 | 0 |
| 577.247752 | 577.247677 | 2493540 | 30 | 42 | 0 | 9 | 1 | 0 |
| 577.26517 | 577.265436 | 4831844 | 30 | 42 | 0 | 11 | 0 | 0 |
| 577.280191 | 577.280692 | 2145636 | 34 | 42 | 0 | 8 | 0 | 0 |
| 577.301889 | 577.301821 | 2373220 | 31 | 46 | 0 | 10 | 0 | 0 |
| 577.316865 | 577.317077 | 1578340 | 35 | 46 | 0 | 7 | 0 | 0 |
| 579.150067 | 579.150148 | 1380569 | 22 | 32 | 2 | 14 | 1 | 0 |
| 579.186635 | 579.187185 | 2032601 | 31 | 32 | 0 | 11 | 0 | 0 |
| 579.190415 | 579.190556 | 3506265 | 28 | 36 | 0 | 11 | 1 | 0 |
| 579.208218 | 579.208315 | 1961049 | 28 | 36 | 0 | 13 | 0 | 0 |
| 579.222895 | 579.222919 | 2491993 | 24 | 40 | 2 | 12 | 1 | 0 |
| 579.226921 | 579.226942 | 4428377 | 29 | 40 | 0 | 10 | 1 | 0 |
| 579.244594 | 579.2447 | 2751064 | 29 | 40 | 0 | 12 | 0 | 0 |
| 579.259885 | 579.259956 | 3337816 | 33 | 40 | 0 | 9 | 0 | 0 |
| 579.263011 | 579.263327 | 2802264 | 30 | 44 | 0 | 9 | 1 | 0 |
| 579.281 | 579.281086 | 2664536 | 30 | 44 | 0 | 11 | 0 | 0 |
| 579.296365 | 579.296342 | 2312792 | 34 | 44 | 0 | 8 | 0 | 0 |
| 579.317723 | 579.317471 | 1410648 | 31 | 48 | 0 | 10 | 0 | 0 |
| 581.144792 | 581.145321 | 1448784 | 33 | 26 | 0 | 10 | 0 | 0 |
| 581.1663 | 581.16645 | 1627984 | 30 | 30 | 0 | 12 | 0 | 0 |
| 581.169823 | 581.169821 | 2634832 | 27 | 34 | 0 | 12 | 1 | 0 |
| 581.202205 | 581.202184 | 2308176 | 23 | 38 | 2 | 13 | 1 | 0 |
| 581.206211 | 581.206206 | 4427344 | 28 | 38 | 0 | 11 | 1 | 0 |
| 581.223826 | 581.223965 | 1502288 | 28 | 38 | 0 | 13 | 0 | 0 |
| 581.239096 | 581.239221 | 3608656 | 32 | 38 | 0 | 10 | 0 | 0 |
| 581.242504 | 581.242592 | 4779600 | 29 | 42 | 0 | 10 | 1 | 0 |
| 581.259938 | 581.26035 | 2107472 | 29 | 42 | 0 | 12 | 0 | 0 |
| 581.27466 | 581.274955 | 2583632 | 25 | 46 | 2 | 11 | 1 | 0 |
| 581.278857 | 581.278977 | 2864720 | 30 | 46 | 0 | 9 | 1 | 0 |
| 581.296577 | 581.296736 | 1782864 | 30 | 46 | 0 | 11 | 0 | 0 |
| 581.31196 | 581.311992 | 1837392 | 34 | 46 | 0 | 8 | 0 | 0 |
| 583.185556 | 583.185471 | 2879051 | 27 | 36 | 0 | 12 | 1 | 0 |
| 583.217446 | 583.217834 | 2119243 | 23 | 40 | 2 | 13 | 1 | 0 |
| 583.221677 | 583.221856 | 4786251 | 28 | 40 | 0 | 11 | 1 | 0 |
| 583.254744 | 583.254871 | 3149899 | 32 | 40 | 0 | 10 | 0 | 0 |
| 583.257917 | 583.258242 | 3351627 | 29 | 44 | 0 | 10 | 1 | 0 |
| 583.290908 | 583.291257 | 2855755 | 33 | 44 | 0 | 9 | 0 | 0 |
| 583.32746 | 583.327642 | 1689418 | 34 | 48 | 0 | 8 | 0 | 0 |
| 585.17621 | 585.176621 | 1467720 | 33 | 30 | 0 | 10 | 0 | 0 |
| 585.19711 | 585.197098 | 2261576 | 22 | 38 | 2 | 14 | 1 | 0 |
| 585.200986 | 585.201121 | 3438152 | 27 | 38 | 0 | 12 | 1 | 0 |
| 585.21289 | 585.213006 | 1805128 | 34 | 34 | 0 | 9 | 0 | 0 |
| 585.233956 | 585.234136 | 3375176 | 31 | 38 | 0 | 11 | 0 | 0 |
| 585.23744 | 585.237506 | 3808840 | 28 | 42 | 0 | 11 | 1 | 0 |
| 585.249346 | 585.249392 | 1463624 | 35 | 38 | 0 | 8 | 0 | 0 |
| 585.270253 | 585.270521 | 3921480 | 32 | 42 | 0 | 10 | 0 | 0 |
| 585.273829 | 585.273892 | 2094664 | 29 | 46 | 0 | 10 | 1 | 0 |
| 585.285494 | 585.285777 | 1463368 | 36 | 42 | 0 | 7 | 0 | 0 |
| 585.306447 | 585.306907 | 2288392 | 33 | 46 | 0 | 9 | 0 | 0 |
| 587.176851 | 587.177015 | 1611081 | 29 | 32 | 0 | 13 | 0 | 0 |
| 587.191564 | 587.191619 | 1435273 | 25 | 36 | 2 | 12 | 1 | 0 |
| 587.213245 | 587.2134 | 2731081 | 30 | 36 | 0 | 12 | 0 | 0 |
| 587.216791 | 587.216771 | 2244169 | 27 | 40 | 0 | 12 | 1 | 0 |
| 587.228495 | 587.228656 | 2033737 | 34 | 36 | 0 | 9 | 0 | 0 |
| 587.249608 | 587.249786 | 3904073 | 31 | 40 | 0 | 11 | 0 | 0 |
| 587.264986 | 587.265042 | 1493833 | 35 | 40 | 0 | 8 | 0 | 0 |
| 587.286124 | 587.286171 | 3345993 | 32 | 44 | 0 | 10 | 0 | 0 |
| 587.322051 | 587.322557 | 1931849 | 33 | 48 | 0 | 9 | 0 | 0 |
| 589.134838 | 589.13515 | 1421260 | 31 | 26 | 0 | 12 | 0 | 0 |
| 589.171781 | 589.171535 | 1514060 | 32 | 30 | 0 | 11 | 0 | 0 |
| 589.192461 | 589.192665 | 1517901 | 29 | 34 | 0 | 13 | 0 | 0 |
| 589.207533 | 589.207921 | 1967437 | 33 | 34 | 0 | 10 | 0 | 0 |
| 589.21138 | 589.211292 | 1714253 | 30 | 38 | 0 | 10 | 1 | 0 |
| 589.228688 | 589.22905 | 3530573 | 30 | 38 | 0 | 12 | 0 | 0 |
| 589.244128 | 589.244306 | 2125901 | 34 | 38 | 0 | 9 | 0 | 0 |
| 589.265345 | 589.265436 | 3156557 | 31 | 42 | 0 | 11 | 0 | 0 |
| 589.280633 | 589.280692 | 1852493 | 35 | 42 | 0 | 8 | 0 | 0 |
| 589.301718 | 589.301821 | 2690637 | 32 | 46 | 0 | 10 | 0 | 0 |
| 591.150523 | 591.1508 | 1482067 | 31 | 28 | 0 | 12 | 0 | 0 |
| 591.186804 | 591.187185 | 1905747 | 32 | 32 | 0 | 11 | 0 | 0 |
| 591.190506 | 591.190556 | 2004051 | 29 | 36 | 0 | 11 | 1 | 0 |
| 591.208151 | 591.208315 | 1733715 | 29 | 36 | 0 | 13 | 0 | 0 |
| 591.226741 | 591.226942 | 2209363 | 30 | 40 | 0 | 10 | 1 | 0 |
| 591.244676 | 591.2447 | 3456595 | 30 | 40 | 0 | 12 | 0 | 0 |
| 591.259862 | 591.259956 | 2173779 | 34 | 40 | 0 | 9 | 0 | 0 |
| 591.280844 | 591.281086 | 2524243 | 31 | 44 | 0 | 11 | 0 | 0 |
| 591.296216 | 591.296342 | 2184787 | 35 | 44 | 0 | 8 | 0 | 0 |
| 591.316404 | 591.316819 | 1697875 | 24 | 52 | 2 | 12 | 1 | 0 |
| 593.170087 | 593.169821 | 2119260 | 28 | 34 | 0 | 12 | 1 | 0 |
| 593.206183 | 593.206206 | 3743324 | 29 | 38 | 0 | 11 | 1 | 0 |
| 593.224022 | 593.223965 | 1868892 | 29 | 38 | 0 | 13 | 0 | 0 |
| 593.242541 | 593.242592 | 3101276 | 30 | 42 | 0 | 10 | 1 | 0 |
| 593.260264 | 593.26035 | 2904668 | 30 | 42 | 0 | 12 | 0 | 0 |
| 593.275203 | 593.275606 | 2501724 | 34 | 42 | 0 | 9 | 0 | 0 |
| 593.296736 | 593.296736 | 1361500 | 31 | 46 | 0 | 11 | 0 | 0 |
| 593.311404 | 593.311992 | 2243932 | 35 | 46 | 0 | 8 | 0 | 0 |
| 595.144627 | 595.145063 | 1399655 | 22 | 32 | 2 | 15 | 1 | 0 |
| 595.185509 | 595.185471 | 2672743 | 28 | 36 | 0 | 12 | 1 | 0 |
| 595.218328 | 595.218486 | 2436200 | 32 | 36 | 0 | 11 | 0 | 0 |
| 595.221811 | 595.221856 | 5031528 | 29 | 40 | 0 | 11 | 1 | 0 |
| 595.233525 | 595.233742 | 1713256 | 36 | 36 | 0 | 8 | 0 | 0 |
| 595.254889 | 595.254871 | 2742888 | 33 | 40 | 0 | 10 | 0 | 0 |
| 595.258137 | 595.258242 | 3611752 | 30 | 44 | 0 | 10 | 1 | 0 |
| 595.275976 | 595.276 | 1868392 | 30 | 44 | 0 | 12 | 0 | 0 |
| 595.291196 | 595.291257 | 3079784 | 34 | 44 | 0 | 9 | 0 | 0 |
| 595.327085 | 595.327642 | 1743464 | 35 | 48 | 0 | 8 | 0 | 0 |
| 597.140193 | 597.140235 | 1750133 | 33 | 26 | 0 | 11 | 0 | 0 |
| 597.201042 | 597.201121 | 3688566 | 28 | 38 | 0 | 12 | 1 | 0 |
| 597.233712 | 597.234136 | 2156406 | 32 | 38 | 0 | 11 | 0 | 0 |
| 597.237379 | 597.237506 | 4565622 | 29 | 42 | 0 | 11 | 1 | 0 |
| 597.270135 | 597.270521 | 2685046 | 33 | 42 | 0 | 10 | 0 | 0 |
| 597.273743 | 597.273892 | 3433078 | 30 | 46 | 0 | 10 | 1 | 0 |
| 597.30677 | 597.306907 | 1991286 | 34 | 46 | 0 | 9 | 0 | 0 |
| 599.155382 | 599.155885 | 1422726 | 33 | 28 | 0 | 11 | 0 | 0 |
| 599.180237 | 599.180524 | 2357382 | 48 | 24 | 0 | 0 | 0 | 0 |
| 599.216728 | 599.216771 | 3590790 | 28 | 40 | 0 | 12 | 1 | 0 |
| 599.228772 | 599.228656 | 1494662 | 35 | 36 | 0 | 9 | 0 | 0 |
| 599.249623 | 599.249786 | 3313286 | 32 | 40 | 0 | 11 | 0 | 0 |
| 599.252872 | 599.253156 | 2638214 | 29 | 44 | 0 | 11 | 1 | 0 |
| 599.264858 | 599.265042 | 1419655 | 36 | 40 | 0 | 8 | 0 | 0 |
| 599.286113 | 599.286171 | 2371719 | 33 | 44 | 0 | 10 | 0 | 0 |
| 599.322628 | 599.322557 | 2139783 | 34 | 48 | 0 | 9 | 0 | 0 |
| 601.196047 | 601.196174 | 1581977 | 48 | 26 | 0 | 0 | 0 | 0 |
| 601.207803 | 601.207921 | 1544345 | 34 | 34 | 0 | 10 | 0 | 0 |
| 601.228932 | 601.22905 | 2262681 | 31 | 38 | 0 | 12 | 0 | 0 |
| 601.232334 | 601.232421 | 1952409 | 28 | 42 | 0 | 12 | 1 | 0 |
| 601.243872 | 601.244306 | 1586073 | 35 | 38 | 0 | 9 | 0 | 0 |
| 601.265284 | 601.265436 | 2762393 | 32 | 42 | 0 | 11 | 0 | 0 |
| 601.280348 | 601.280692 | 1478554 | 36 | 42 | 0 | 8 | 0 | 0 |
| 601.301819 | 601.301821 | 2014106 | 33 | 46 | 0 | 10 | 0 | 0 |
| 603.150449 | 603.1508 | 1342893 | 32 | 28 | 0 | 12 | 0 | 0 |
| 603.208138 | 603.208315 | 1952942 | 30 | 36 | 0 | 13 | 0 | 0 |
| 603.22358 | 603.223571 | 1794478 | 34 | 36 | 0 | 10 | 0 | 0 |
| 603.244492 | 603.2447 | 2556590 | 31 | 40 | 0 | 12 | 0 | 0 |
| 603.259586 | 603.259956 | 1836462 | 35 | 40 | 0 | 9 | 0 | 0 |
| 603.280774 | 603.281086 | 2764975 | 32 | 44 | 0 | 11 | 0 | 0 |
| 603.295481 | 603.29569 | 1806511 | 28 | 48 | 2 | 10 | 1 | 0 |
| 603.317429 | 603.317471 | 1432239 | 33 | 48 | 0 | 10 | 0 | 0 |
| 605.202065 | 605.202184 | 1931973 | 25 | 38 | 2 | 13 | 1 | 0 |
| 605.205768 | 605.206206 | 1848517 | 30 | 38 | 0 | 11 | 1 | 0 |
| 605.223819 | 605.223965 | 2317509 | 30 | 38 | 0 | 13 | 0 | 0 |
| 605.239099 | 605.239221 | 1724613 | 34 | 38 | 0 | 10 | 0 | 0 |
| 605.260221 | 605.26035 | 2675398 | 31 | 42 | 0 | 12 | 0 | 0 |
| 605.275197 | 605.275606 | 2458310 | 35 | 42 | 0 | 9 | 0 | 0 |
| 605.29656 | 605.296736 | 2164166 | 32 | 46 | 0 | 11 | 0 | 0 |
| 605.311931 | 605.311992 | 1415622 | 36 | 46 | 0 | 8 | 0 | 0 |
| 607.145962 | 607.145715 | 1335262 | 31 | 28 | 0 | 13 | 0 | 0 |
| 607.185256 | 607.185471 | 2404062 | 29 | 36 | 0 | 12 | 1 | 0 |
| 607.221643 | 607.221856 | 2859231 | 30 | 40 | 0 | 11 | 1 | 0 |
| 607.239404 | 607.239615 | 1945055 | 30 | 40 | 0 | 13 | 0 | 0 |
| 607.254402 | 607.254871 | 1758431 | 34 | 40 | 0 | 10 | 0 | 0 |
| 607.275967 | 607.276 | 1852639 | 31 | 44 | 0 | 12 | 0 | 0 |
| 607.290437 | 607.290605 | 1705695 | 27 | 48 | 2 | 11 | 1 | 0 |
| 607.312382 | 607.312386 | 1531104 | 32 | 48 | 0 | 11 | 0 | 0 |
| 609.164682 | 609.164874 | 1541369 | 49 | 22 | 0 | 0 | 0 | 0 |
| 609.200697 | 609.201121 | 2875641 | 29 | 38 | 0 | 12 | 1 | 0 |
| 609.233449 | 609.233484 | 2127226 | 25 | 42 | 2 | 13 | 1 | 0 |
| 609.237355 | 609.237506 | 4249338 | 30 | 42 | 0 | 11 | 1 | 0 |
| 609.255111 | 609.255265 | 1845498 | 30 | 42 | 0 | 13 | 0 | 0 |
| 609.270458 | 609.270521 | 2307066 | 34 | 42 | 0 | 10 | 0 | 0 |
| 609.306616 | 609.306907 | 2061051 | 35 | 46 | 0 | 9 | 0 | 0 |
| 609.343217 | 609.343292 | 1368571 | 36 | 50 | 0 | 8 | 0 | 0 |
| 611.140854 | 611.140629 | 1453846 | 30 | 28 | 0 | 14 | 0 | 0 |
| 611.180432 | 611.180524 | 2482966 | 49 | 24 | 0 | 0 | 0 | 0 |
| 611.192289 | 611.192271 | 1371926 | 35 | 32 | 0 | 10 | 0 | 0 |
| 611.21658 | 611.216771 | 3712791 | 29 | 40 | 0 | 12 | 1 | 0 |
| 611.252925 | 611.253156 | 3696919 | 30 | 44 | 0 | 11 | 1 | 0 |
| 611.286113 | 611.286171 | 2561816 | 34 | 44 | 0 | 10 | 0 | 0 |
| 611.321792 | 611.321905 | 1641112 | 27 | 52 | 2 | 11 | 1 | 0 |
| 613.195865 | 613.196174 | 2705717 | 49 | 26 | 0 | 0 | 0 | 0 |
| 613.229014 | 613.22905 | 2217782 | 32 | 38 | 0 | 12 | 0 | 0 |
| 613.232303 | 613.232421 | 3390774 | 29 | 42 | 0 | 12 | 1 | 0 |
| 613.265487 | 613.265436 | 2425142 | 33 | 42 | 0 | 11 | 0 | 0 |
| 613.268672 | 613.268807 | 2806582 | 30 | 46 | 0 | 11 | 1 | 0 |
| 613.301681 | 613.301821 | 2672439 | 34 | 46 | 0 | 10 | 0 | 0 |
| 613.337887 | 613.338207 | 1782584 | 35 | 50 | 0 | 9 | 0 | 0 |
| 615.207846 | 615.208315 | 1512790 | 31 | 36 | 0 | 13 | 0 | 0 |
| 615.211524 | 615.211824 | 1708886 | 49 | 28 | 0 | 0 | 0 | 0 |
| 615.22345 | 615.223571 | 1558358 | 35 | 36 | 0 | 10 | 0 | 0 |
| 615.244469 | 615.2447 | 1993815 | 32 | 40 | 0 | 12 | 0 | 0 |
| 615.259709 | 615.259956 | 1568343 | 36 | 40 | 0 | 9 | 0 | 0 |
| 615.28107 | 615.281086 | 3033431 | 33 | 44 | 0 | 11 | 0 | 0 |
| 615.317392 | 615.317471 | 1825112 | 34 | 48 | 0 | 10 | 0 | 0 |
| 615.353866 | 615.353857 | 1452888 | 35 | 52 | 0 | 9 | 0 | 0 |
| 617.130185 | 617.130064 | 1359991 | 32 | 26 | 0 | 13 | 0 | 0 |
| 617.187498 | 617.187579 | 1393016 | 30 | 34 | 0 | 14 | 0 | 0 |
| 617.202733 | 617.202835 | 1420920 | 34 | 34 | 0 | 11 | 0 | 0 |
| 617.223623 | 617.223965 | 1915512 | 31 | 38 | 0 | 13 | 0 | 0 |
| 617.239077 | 617.239221 | 1977209 | 35 | 38 | 0 | 10 | 0 | 0 |
| 617.260175 | 617.26035 | 2814841 | 32 | 42 | 0 | 12 | 0 | 0 |
| 617.275462 | 617.275606 | 1931641 | 36 | 42 | 0 | 9 | 0 | 0 |
| 617.296156 | 617.296736 | 2131066 | 33 | 46 | 0 | 11 | 0 | 0 |
| 617.33309 | 617.333121 | 1348474 | 34 | 50 | 0 | 10 | 0 | 0 |
| 619.181638 | 619.1821 | 1390747 | 33 | 32 | 0 | 12 | 0 | 0 |
| 619.218406 | 619.218486 | 1764764 | 34 | 36 | 0 | 11 | 0 | 0 |
| 619.239536 | 619.239615 | 1789340 | 31 | 40 | 0 | 13 | 0 | 0 |
| 619.254772 | 619.254871 | 1829021 | 35 | 40 | 0 | 10 | 0 | 0 |
| 619.27507 | 619.275348 | 2156445 | 24 | 48 | 2 | 14 | 1 | 0 |
| 619.291212 | 619.291257 | 1690013 | 36 | 44 | 0 | 9 | 0 | 0 |
| 619.312342 | 619.312386 | 1524638 | 33 | 48 | 0 | 11 | 0 | 0 |
| 621.200926 | 621.201121 | 2169793 | 30 | 38 | 0 | 12 | 1 | 0 |
| 621.233511 | 621.233484 | 1781441 | 26 | 42 | 2 | 13 | 1 | 0 |
| 621.255156 | 621.255265 | 1518146 | 31 | 42 | 0 | 13 | 0 | 0 |
| 621.270135 | 621.270521 | 2164930 | 35 | 42 | 0 | 10 | 0 | 0 |
| 621.291605 | 621.29165 | 1629378 | 32 | 46 | 0 | 12 | 0 | 0 |
| 623.176047 | 623.176363 | 1442535 | 24 | 36 | 2 | 15 | 1 | 0 |
| 623.216765 | 623.216771 | 3105767 | 30 | 40 | 0 | 12 | 1 | 0 |
| 623.249619 | 623.249786 | 1900520 | 34 | 40 | 0 | 11 | 0 | 0 |
| 623.253131 | 623.253156 | 1788904 | 31 | 44 | 0 | 11 | 1 | 0 |
| 623.284993 | 623.285519 | 2224105 | 27 | 48 | 2 | 12 | 1 | 0 |
| 623.321838 | 623.321905 | 1395561 | 28 | 52 | 2 | 11 | 1 | 0 |
| 625.195861 | 625.196174 | 2462223 | 50 | 26 | 0 | 0 | 0 | 0 |
| 625.232245 | 625.232421 | 3402767 | 30 | 42 | 0 | 12 | 1 | 0 |
| 625.265028 | 625.265436 | 2328592 | 34 | 42 | 0 | 11 | 0 | 0 |
| 625.268815 | 625.268807 | 1477136 | 31 | 46 | 0 | 11 | 1 | 0 |
| 625.301525 | 625.301821 | 2555409 | 35 | 46 | 0 | 10 | 0 | 0 |
| 625.338002 | 625.338207 | 1578769 | 36 | 50 | 0 | 9 | 0 | 0 |
| 627.211613 | 627.211824 | 2748984 | 50 | 28 | 0 | 0 | 0 | 0 |
| 627.247814 | 627.248071 | 2563640 | 30 | 44 | 0 | 12 | 1 | 0 |
| 627.280982 | 627.281086 | 2651193 | 34 | 44 | 0 | 11 | 0 | 0 |
| 627.317106 | 627.317471 | 2029882 | 35 | 48 | 0 | 10 | 0 | 0 |
| 629.190025 | 629.189785 | 1614177 | 33 | 34 | 4 | 5 | 2 | 0 |
| 629.227052 | 629.227475 | 2394210 | 50 | 30 | 0 | 0 | 0 | 0 |
| 629.259543 | 629.259698 | 2220898 | 25 | 46 | 2 | 14 | 1 | 0 |
| 629.275536 | 629.275606 | 1427299 | 37 | 42 | 0 | 9 | 0 | 0 |
| 629.296115 | 629.296736 | 2093155 | 34 | 46 | 0 | 11 | 0 | 0 |
| 629.333077 | 629.333121 | 1611876 | 35 | 50 | 0 | 10 | 0 | 0 |
| 631.217329 | 631.217834 | 1492876 | 27 | 40 | 2 | 13 | 1 | 0 |
| 631.238635 | 631.238963 | 1496460 | 24 | 44 | 2 | 15 | 1 | 0 |
| 631.254465 | 631.254871 | 1707917 | 36 | 40 | 0 | 10 | 0 | 0 |
| 631.275026 | 631.275348 | 1768077 | 25 | 48 | 2 | 14 | 1 | 0 |
| 631.291256 | 631.291257 | 1377934 | 37 | 44 | 0 | 9 | 0 | 0 |
| 631.312103 | 631.312386 | 1751694 | 34 | 48 | 0 | 11 | 0 | 0 |
| 633.197509 | 633.19775 | 1382839 | 34 | 34 | 0 | 12 | 0 | 0 |
| 633.21869 | 633.218879 | 1641143 | 31 | 38 | 0 | 14 | 0 | 0 |
| 633.25509 | 633.255265 | 1918904 | 32 | 42 | 0 | 13 | 0 | 0 |
| 633.270148 | 633.270521 | 1513912 | 36 | 42 | 0 | 10 | 0 | 0 |
| 633.291546 | 633.29165 | 2014393 | 33 | 46 | 0 | 12 | 0 | 0 |
| 633.305751 | 633.306255 | 1382809 | 29 | 50 | 2 | 11 | 1 | 0 |
| 633.326893 | 633.327384 | 1465530 | 26 | 54 | 2 | 13 | 1 | 0 |
| 635.180409 | 635.180385 | 1389538 | 30 | 36 | 0 | 13 | 1 | 0 |
| 635.21321 | 635.2134 | 1639139 | 34 | 36 | 0 | 12 | 0 | 0 |
| 635.249652 | 635.249786 | 2041572 | 35 | 40 | 0 | 11 | 0 | 0 |
| 635.286062 | 635.286171 | 2045669 | 36 | 44 | 0 | 10 | 0 | 0 |
| 635.306527 | 635.306649 | 1412965 | 25 | 52 | 2 | 14 | 1 | 0 |
| 635.322171 | 635.322557 | 2231014 | 37 | 48 | 0 | 9 | 0 | 0 |
| 637.195493 | 637.196036 | 1792271 | 30 | 38 | 0 | 13 | 1 | 0 |
| 637.228554 | 637.22905 | 1808656 | 34 | 38 | 0 | 12 | 0 | 0 |
| 637.265342 | 637.265436 | 1587985 | 35 | 42 | 0 | 11 | 0 | 0 |
| 637.301469 | 637.301821 | 2155794 | 36 | 46 | 0 | 10 | 0 | 0 |
| 637.337821 | 637.338207 | 1399570 | 37 | 50 | 0 | 9 | 0 | 0 |
| 639.211379 | 639.211686 | 2403133 | 30 | 40 | 0 | 13 | 1 | 0 |
| 639.244625 | 639.2447 | 1676093 | 34 | 40 | 0 | 12 | 0 | 0 |
| 639.28048 | 639.281086 | 2275134 | 35 | 44 | 0 | 11 | 0 | 0 |
| 639.31669 | 639.316819 | 1603135 | 28 | 52 | 2 | 12 | 1 | 0 |
| 641.189807 | 641.190298 | 1474921 | 21 | 42 | 2 | 16 | 2 | 0 |
| 641.227363 | 641.227336 | 2630506 | 30 | 42 | 0 | 13 | 1 | 0 |
| 641.260225 | 641.26035 | 2256235 | 34 | 42 | 0 | 12 | 0 | 0 |
| 641.296585 | 641.296736 | 2290540 | 35 | 46 | 0 | 11 | 0 | 0 |
| 643.242993 | 643.242986 | 1718168 | 30 | 44 | 0 | 13 | 1 | 0 |
| 643.275812 | 643.276 | 2415512 | 34 | 44 | 0 | 12 | 0 | 0 |
| 643.290799 | 643.291257 | 1432985 | 38 | 44 | 0 | 9 | 0 | 0 |
| 643.31127 | 643.311734 | 2115993 | 27 | 52 | 2 | 13 | 1 | 0 |
| 645.255313 | 645.255265 | 2041797 | 33 | 42 | 0 | 13 | 0 | 0 |
| 645.270654 | 645.270521 | 1467334 | 37 | 42 | 0 | 10 | 0 | 0 |
| 645.291578 | 645.29165 | 1707974 | 34 | 46 | 0 | 12 | 0 | 0 |
| 645.306716 | 645.306907 | 1621446 | 38 | 46 | 0 | 9 | 0 | 0 |
| 645.327682 | 645.328036 | 2054343 | 35 | 50 | 0 | 11 | 0 | 0 |
| 647.233685 | 647.233878 | 1533938 | 24 | 44 | 2 | 16 | 1 | 0 |
| 647.249771 | 647.249786 | 1621490 | 36 | 40 | 0 | 11 | 0 | 0 |
| 647.269961 | 647.270263 | 1631731 | 25 | 48 | 2 | 15 | 1 | 0 |
| 647.286023 | 647.286171 | 1559539 | 37 | 44 | 0 | 10 | 0 | 0 |
| 647.306524 | 647.306649 | 1518068 | 26 | 52 | 2 | 14 | 1 | 0 |
| 647.322274 | 647.322557 | 1391604 | 38 | 48 | 0 | 9 | 0 | 0 |
| 649.192117 | 649.192665 | 1372702 | 34 | 34 | 0 | 13 | 0 | 0 |
| 649.249894 | 649.25018 | 1331487 | 32 | 42 | 0 | 14 | 0 | 0 |
| 649.264412 | 649.264784 | 1698592 | 28 | 46 | 2 | 13 | 1 | 0 |
| 649.301067 | 649.301169 | 1690400 | 29 | 50 | 2 | 12 | 1 | 0 |
| 649.322052 | 649.322299 | 1416993 | 26 | 54 | 2 | 14 | 1 | 0 |
| 651.207824 | 651.208315 | 1432139 | 34 | 36 | 0 | 13 | 0 | 0 |
| 651.24464 | 651.2447 | 1952588 | 35 | 40 | 0 | 12 | 0 | 0 |
| 651.265563 | 651.26583 | 1362508 | 32 | 44 | 0 | 14 | 0 | 0 |
| 651.280853 | 651.281086 | 1868109 | 36 | 44 | 0 | 11 | 0 | 0 |
| 651.317222 | 651.317471 | 1713997 | 37 | 48 | 0 | 10 | 0 | 0 |
| 653.18717 | 653.187579 | 1334903 | 33 | 34 | 0 | 14 | 0 | 0 |
| 653.223304 | 653.223313 | 1672055 | 26 | 42 | 2 | 15 | 1 | 0 |
| 653.259795 | 653.26035 | 2160504 | 35 | 42 | 0 | 12 | 0 | 0 |
| 653.263594 | 653.263721 | 1380984 | 32 | 46 | 0 | 12 | 1 | 0 |
| 653.296589 | 653.296736 | 2642553 | 36 | 46 | 0 | 11 | 0 | 0 |
| 653.333041 | 653.333121 | 1450874 | 37 | 50 | 0 | 10 | 0 | 0 |
| 655.206483 | 655.2066 | 1760418 | 30 | 40 | 0 | 14 | 1 | 0 |
| 655.238893 | 655.238963 | 1607715 | 26 | 44 | 2 | 15 | 1 | 0 |
| 655.2756 | 655.276 | 1901348 | 35 | 44 | 0 | 12 | 0 | 0 |
| 655.312069 | 655.312386 | 1977509 | 36 | 48 | 0 | 11 | 0 | 0 |
| 657.222103 | 657.22225 | 1995982 | 30 | 42 | 0 | 14 | 1 | 0 |
| 657.254905 | 657.255265 | 1690830 | 34 | 42 | 0 | 13 | 0 | 0 |
| 657.258491 | 657.258636 | 1715406 | 31 | 46 | 0 | 13 | 1 | 0 |
| 657.291426 | 657.29165 | 1578639 | 35 | 46 | 0 | 12 | 0 | 0 |
| 657.327965 | 657.328036 | 1404624 | 36 | 50 | 0 | 11 | 0 | 0 |
| 659.237748 | 659.2379 | 1520888 | 30 | 44 | 0 | 14 | 1 | 0 |
| 659.270706 | 659.270915 | 1505529 | 34 | 44 | 0 | 13 | 0 | 0 |
| 659.285497 | 659.285519 | 1474809 | 30 | 48 | 2 | 12 | 1 | 0 |
| 659.307208 | 659.307301 | 1970169 | 35 | 48 | 0 | 12 | 0 | 0 |
| 661.228786 | 661.22905 | 1383457 | 36 | 38 | 0 | 12 | 0 | 0 |
| 661.250042 | 661.25018 | 1408289 | 33 | 42 | 0 | 14 | 0 | 0 |
| 661.264895 | 661.265436 | 1415969 | 37 | 42 | 0 | 11 | 0 | 0 |
| 661.286569 | 661.286565 | 2067746 | 34 | 46 | 0 | 13 | 0 | 0 |
| 661.301813 | 661.301821 | 1373602 | 38 | 46 | 0 | 10 | 0 | 0 |
| 661.337478 | 661.337555 | 1442339 | 31 | 54 | 2 | 11 | 1 | 0 |
| 663.244768 | 663.2447 | 1582921 | 36 | 40 | 0 | 12 | 0 | 0 |
| 663.280682 | 663.281086 | 2039113 | 37 | 44 | 0 | 11 | 0 | 0 |
| 663.30226 | 663.302215 | 1661258 | 34 | 48 | 0 | 13 | 0 | 0 |
| 663.316548 | 663.316819 | 1469258 | 30 | 52 | 2 | 12 | 1 | 0 |
| 665.223896 | 665.223965 | 1759087 | 35 | 38 | 0 | 13 | 0 | 0 |
| 665.227035 | 665.227336 | 1906543 | 32 | 42 | 0 | 13 | 1 | 0 |
| 665.259746 | 665.26035 | 1613168 | 36 | 42 | 0 | 12 | 0 | 0 |
| 665.296242 | 665.296736 | 2096496 | 37 | 46 | 0 | 11 | 0 | 0 |
| 667.242764 | 667.242986 | 1518485 | 32 | 44 | 0 | 13 | 1 | 0 |
| 667.275799 | 667.276 | 1870741 | 36 | 44 | 0 | 12 | 0 | 0 |
| 667.310744 | 667.310885 | 2002838 | 31 | 57 | 0 | 9 | 2 | 1 |
| 667.34812 | 667.348771 | 1520279 | 38 | 52 | 0 | 10 | 0 | 0 |
| 669.254536 | 669.254613 | 1560889 | 27 | 46 | 2 | 15 | 1 | 0 |
| 669.291477 | 669.29165 | 2224057 | 36 | 46 | 0 | 12 | 0 | 0 |
| 669.32742 | 669.328036 | 1537338 | 37 | 50 | 0 | 11 | 0 | 0 |
| 669.363085 | 669.36292 | 1395131 | 32 | 63 | 0 | 8 | 2 | 1 |
| 671.234376 | 671.23453 | 1567707 | 34 | 40 | 0 | 14 | 0 | 0 |
| 671.249522 | 671.249786 | 1331163 | 38 | 40 | 0 | 11 | 0 | 0 |
| 671.27067 | 671.270915 | 1934299 | 35 | 44 | 0 | 13 | 0 | 0 |
| 671.306998 | 671.307301 | 2205916 | 36 | 48 | 0 | 12 | 0 | 0 |
| 673.24998 | 673.25018 | 1552379 | 34 | 42 | 0 | 14 | 0 | 0 |
| 673.284951 | 673.285064 | 2253308 | 29 | 55 | 0 | 11 | 2 | 1 |
| 673.322726 | 673.322951 | 1665020 | 36 | 50 | 0 | 12 | 0 | 0 |
| 673.358527 | 673.358684 | 1355773 | 29 | 58 | 2 | 13 | 1 | 0 |
| 675.265387 | 675.26583 | 1534746 | 34 | 44 | 0 | 14 | 0 | 0 |
| 675.280789 | 675.281086 | 1345818 | 38 | 44 | 0 | 11 | 0 | 0 |
| 675.301895 | 675.302215 | 2342939 | 35 | 48 | 0 | 13 | 0 | 0 |
| 677.223512 | 677.223965 | 1621814 | 36 | 38 | 0 | 13 | 0 | 0 |
| 677.260204 | 677.26035 | 1599031 | 37 | 42 | 0 | 12 | 0 | 0 |
| 677.296338 | 677.296736 | 1848375 | 38 | 46 | 0 | 11 | 0 | 0 |
| 677.332737 | 677.333121 | 1480504 | 39 | 50 | 0 | 10 | 0 | 0 |
| 679.182212 | 679.1821 | 1637968 | 38 | 32 | 0 | 12 | 0 | 0 |
| 679.206517 | 679.2066 | 1657425 | 32 | 40 | 0 | 14 | 1 | 0 |
| 679.275073 | 679.275348 | 2348114 | 29 | 48 | 2 | 14 | 1 | 0 |
| 679.312411 | 679.312386 | 1638994 | 38 | 48 | 0 | 11 | 0 | 0 |
| 679.348769 | 679.348771 | 1853010 | 39 | 52 | 0 | 10 | 0 | 0 |
| 681.254742 | 681.255265 | 2139242 | 36 | 42 | 0 | 13 | 0 | 0 |
| 681.291264 | 681.29165 | 2038378 | 37 | 46 | 0 | 12 | 0 | 0 |
| 681.327621 | 681.328036 | 1895530 | 38 | 50 | 0 | 11 | 0 | 0 |
| 683.23451 | 683.23453 | 1596543 | 35 | 40 | 0 | 14 | 0 | 0 |
| 683.269617 | 683.270263 | 1766272 | 28 | 48 | 2 | 15 | 1 | 0 |
| 683.30702 | 683.307301 | 1920896 | 37 | 48 | 0 | 12 | 0 | 0 |
| 683.342641 | 683.343034 | 1390081 | 30 | 56 | 2 | 13 | 1 | 0 |
| 685.249719 | 685.25018 | 1513363 | 35 | 42 | 0 | 14 | 0 | 0 |
| 685.286306 | 685.286565 | 1879699 | 36 | 46 | 0 | 13 | 0 | 0 |
| 685.322559 | 685.322951 | 1799828 | 37 | 50 | 0 | 12 | 0 | 0 |
| 687.229382 | 687.229444 | 1441444 | 34 | 40 | 0 | 15 | 0 | 0 |
| 687.265497 | 687.26583 | 1886884 | 35 | 44 | 0 | 14 | 0 | 0 |
| 687.300869 | 687.300714 | 2003365 | 30 | 57 | 0 | 11 | 2 | 1 |
| 687.337989 | 687.338601 | 1366437 | 37 | 52 | 0 | 12 | 0 | 0 |
| 689.244836 | 689.245094 | 1346738 | 34 | 42 | 0 | 15 | 0 | 0 |
| 689.295269 | 689.295235 | 1533107 | 33 | 55 | 0 | 9 | 2 | 1 |
| 689.317707 | 689.317865 | 1875123 | 36 | 50 | 0 | 13 | 0 | 0 |
| 691.275779 | 691.276 | 1474238 | 38 | 44 | 0 | 12 | 0 | 0 |
| 691.312113 | 691.312386 | 1729726 | 39 | 48 | 0 | 11 | 0 | 0 |
| 693.291678 | 693.29165 | 1878215 | 38 | 46 | 0 | 12 | 0 | 0 |
| 693.327792 | 693.328036 | 1970375 | 39 | 50 | 0 | 11 | 0 | 0 |
| 695.269972 | 695.270263 | 1864908 | 29 | 48 | 2 | 15 | 1 | 0 |
| 695.306623 | 695.307301 | 2131660 | 38 | 48 | 0 | 12 | 0 | 0 |
| 695.343356 | 695.343686 | 2012876 | 39 | 52 | 0 | 11 | 0 | 0 |
| 697.216762 | 697.217165 | 1381582 | 32 | 42 | 0 | 15 | 1 | 0 |
| 697.249863 | 697.25018 | 2235598 | 36 | 42 | 0 | 14 | 0 | 0 |
| 697.284819 | 697.284196 | 1713870 | 30 | 51 | 0 | 16 | 0 | 1 |
| 697.322639 | 697.322951 | 2050510 | 38 | 50 | 0 | 12 | 0 | 0 |
| 697.357638 | 697.356967 | 1469134 | 32 | 59 | 0 | 14 | 0 | 1 |
| 699.265508 | 699.26583 | 1610446 | 36 | 44 | 0 | 14 | 0 | 0 |
| 699.301682 | 699.302215 | 2435278 | 37 | 48 | 0 | 13 | 0 | 0 |
| 699.338445 | 699.338601 | 1624782 | 38 | 52 | 0 | 12 | 0 | 0 |

6. Sample: F2_T_15_ (bacteria-only)

| 217.087021 | 217.087018 | 1580607 | 13 | 14 | 0 | 3 | 0 | 0 |
| --- | --- | --- | --- | --- | --- | --- | --- | --- |
| 217.123429 | 217.123403 | 2117960 | 14 | 18 | 0 | 2 | 0 | 0 |
| 219.066307 | 219.066282 | 1384771 | 12 | 12 | 0 | 4 | 0 | 0 |
| 219.102689 | 219.102668 | 2327157 | 13 | 16 | 0 | 3 | 0 | 0 |
| 219.139097 | 219.139053 | 1888230 | 14 | 20 | 0 | 2 | 0 | 0 |
| 221.081958 | 221.081932 | 1778862 | 12 | 14 | 0 | 4 | 0 | 0 |
| 221.118373 | 221.118318 | 1961936 | 13 | 18 | 0 | 3 | 0 | 0 |
| 223.097621 | 223.097583 | 1770215 | 12 | 16 | 0 | 4 | 0 | 0 |
| 223.134017 | 223.133968 | 1223705 | 13 | 20 | 0 | 3 | 0 | 0 |
| 225.076876 | 225.076847 | 1955244 | 11 | 14 | 0 | 5 | 0 | 0 |
| 227.071431 | 227.071368 | 1414845 | 14 | 12 | 0 | 3 | 0 | 0 |
| 227.107807 | 227.107753 | 2307295 | 15 | 16 | 0 | 2 | 0 | 0 |
| 227.144237 | 227.144139 | 1712161 | 16 | 20 | 0 | 1 | 0 | 0 |
| 229.087078 | 229.087018 | 2190364 | 14 | 14 | 0 | 3 | 0 | 0 |
| 229.123456 | 229.123403 | 3662622 | 15 | 18 | 0 | 2 | 0 | 0 |
| 229.159772 | 229.159789 | 1413952 | 16 | 22 | 0 | 1 | 0 | 0 |
| 231.066313 | 231.066282 | 2101123 | 13 | 12 | 0 | 4 | 0 | 0 |
| 231.102707 | 231.102668 | 3724198 | 14 | 16 | 0 | 3 | 0 | 0 |
| 231.139105 | 231.139053 | 3178344 | 15 | 20 | 0 | 2 | 0 | 0 |
| 233.045588 | 233.045547 | 2075637 | 12 | 10 | 0 | 5 | 0 | 0 |
| 233.081961 | 233.081932 | 3742200 | 13 | 14 | 0 | 4 | 0 | 0 |
| 233.118364 | 233.118318 | 6214651 | 14 | 18 | 0 | 3 | 0 | 0 |
| 233.154752 | 233.154703 | 2754621 | 15 | 22 | 0 | 2 | 0 | 0 |
| 235.061259 | 235.061197 | 2131412 | 12 | 12 | 0 | 5 | 0 | 0 |
| 235.097641 | 235.097583 | 5302295 | 13 | 16 | 0 | 4 | 0 | 0 |
| 235.134011 | 235.133968 | 4249050 | 14 | 20 | 0 | 3 | 0 | 0 |
| 235.17047 | 235.170354 | 1176125 | 15 | 24 | 0 | 2 | 0 | 0 |
| 237.076897 | 237.076847 | 2326780 | 12 | 14 | 0 | 5 | 0 | 0 |
| 237.113296 | 237.113233 | 3623487 | 13 | 18 | 0 | 4 | 0 | 0 |
| 237.149637 | 237.149618 | 1624098 | 14 | 22 | 0 | 3 | 0 | 0 |
| 239.056205 | 239.056112 | 1793835 | 11 | 12 | 0 | 6 | 0 | 0 |
| 239.071424 | 239.071368 | 1413164 | 15 | 12 | 0 | 3 | 0 | 0 |
| 239.092531 | 239.092497 | 3560750 | 12 | 16 | 0 | 5 | 0 | 0 |
| 239.107801 | 239.107753 | 2338991 | 16 | 16 | 0 | 2 | 0 | 0 |
| 239.12892 | 239.128883 | 2107665 | 13 | 20 | 0 | 4 | 0 | 0 |
| 239.144162 | 239.144139 | 1895379 | 17 | 20 | 0 | 1 | 0 | 0 |
| 241.050702 | 241.050632 | 1468707 | 14 | 10 | 0 | 4 | 0 | 0 |
| 241.071825 | 241.071762 | 1558821 | 11 | 14 | 0 | 6 | 0 | 0 |
| 241.08708 | 241.087018 | 2546470 | 15 | 14 | 0 | 3 | 0 | 0 |
| 241.108241 | 241.108147 | 1952872 | 12 | 18 | 0 | 5 | 0 | 0 |
| 241.123413 | 241.123403 | 3967306 | 16 | 18 | 0 | 2 | 0 | 0 |
| 241.159879 | 241.159789 | 2183533 | 17 | 22 | 0 | 1 | 0 | 0 |
| 243.029957 | 243.029897 | 1186497 | 13 | 8 | 0 | 5 | 0 | 0 |
| 243.066353 | 243.066282 | 2367973 | 14 | 12 | 0 | 4 | 0 | 0 |
| 243.102694 | 243.102668 | 5242088 | 15 | 16 | 0 | 3 | 0 | 0 |
| 243.139102 | 243.139053 | 6504940 | 16 | 20 | 0 | 2 | 0 | 0 |
| 243.175421 | 243.175439 | 1884719 | 17 | 24 | 0 | 1 | 0 | 0 |
| 245.009181 | 245.009161 | 1219750 | 12 | 6 | 0 | 6 | 0 | 0 |
| 245.045616 | 245.045547 | 2272042 | 13 | 10 | 0 | 5 | 0 | 0 |
| 245.081975 | 245.081932 | 5522222 | 14 | 14 | 0 | 4 | 0 | 0 |
| 245.118336 | 245.118318 | 10162097 | 15 | 18 | 0 | 3 | 0 | 0 |
| 245.15477 | 245.154703 | 7667125 | 16 | 22 | 0 | 2 | 0 | 0 |
| 245.191166 | 245.191089 | 1455929 | 17 | 26 | 0 | 1 | 0 | 0 |
| 247.024808 | 247.024812 | 1558006 | 12 | 8 | 0 | 6 | 0 | 0 |
| 247.061247 | 247.061197 | 3317626 | 13 | 12 | 0 | 5 | 0 | 0 |
| 247.097586 | 247.097583 | 7712510 | 14 | 16 | 0 | 4 | 0 | 0 |
| 247.133998 | 247.133968 | 8703234 | 15 | 20 | 0 | 3 | 0 | 0 |
| 247.170414 | 247.170354 | 2845126 | 16 | 24 | 0 | 2 | 0 | 0 |
| 249.040539 | 249.040462 | 2352332 | 12 | 10 | 0 | 6 | 0 | 0 |
| 249.076872 | 249.076847 | 5116176 | 13 | 14 | 0 | 5 | 0 | 0 |
| 249.113268 | 249.113233 | 10170324 | 14 | 18 | 0 | 4 | 0 | 0 |
| 251.056131 | 251.056112 | 2402664 | 12 | 12 | 0 | 6 | 0 | 0 |
| 251.09255 | 251.092497 | 5849324 | 13 | 16 | 0 | 5 | 0 | 0 |
| 251.107789 | 251.107753 | 1470830 | 17 | 16 | 0 | 2 | 0 | 0 |
| 251.144177 | 251.144139 | 1406962 | 18 | 20 | 0 | 1 | 0 | 0 |
| 253.035488 | 253.035376 | 1229381 | 11 | 10 | 0 | 7 | 0 | 0 |
| 253.050682 | 253.050632 | 1327366 | 15 | 10 | 0 | 4 | 0 | 0 |
| 253.071783 | 253.071762 | 3200777 | 12 | 14 | 0 | 6 | 0 | 0 |
| 253.087067 | 253.087018 | 2770443 | 16 | 14 | 0 | 3 | 0 | 0 |
| 253.108198 | 253.108147 | 7703949 | 13 | 18 | 0 | 5 | 0 | 0 |
| 253.123477 | 253.123403 | 4700559 | 17 | 18 | 0 | 2 | 0 | 0 |
| 253.15985 | 253.159789 | 3100307 | 18 | 22 | 0 | 1 | 0 | 0 |
| 255.066364 | 255.066282 | 3121388 | 15 | 12 | 0 | 4 | 0 | 0 |
| 255.087515 | 255.087412 | 2522158 | 12 | 16 | 0 | 6 | 0 | 0 |
| 255.102681 | 255.102668 | 5905520 | 16 | 16 | 0 | 3 | 0 | 0 |
| 255.139113 | 255.139053 | 8115572 | 17 | 20 | 0 | 2 | 0 | 0 |
| 255.175469 | 255.175439 | 3061688 | 18 | 24 | 0 | 1 | 0 | 0 |
| 257.045642 | 257.045547 | 2886288 | 14 | 10 | 0 | 5 | 0 | 0 |
| 257.081934 | 257.081932 | 6285397 | 15 | 14 | 0 | 4 | 0 | 0 |
| 257.103054 | 257.103062 | 1828695 | 12 | 18 | 0 | 6 | 0 | 0 |
| 257.118391 | 257.118318 | 12673881 | 16 | 18 | 0 | 3 | 0 | 0 |
| 257.15474 | 257.154703 | 10987357 | 17 | 22 | 0 | 2 | 0 | 0 |
| 257.191179 | 257.191089 | 2382433 | 18 | 26 | 0 | 1 | 0 | 0 |
| 259.024847 | 259.024812 | 1969336 | 13 | 8 | 0 | 6 | 0 | 0 |
| 259.061255 | 259.061197 | 4579901 | 14 | 12 | 0 | 5 | 0 | 0 |
| 259.097652 | 259.097583 | 10611777 | 15 | 16 | 0 | 4 | 0 | 0 |
| 259.133995 | 259.133968 | 15788357 | 16 | 20 | 0 | 3 | 0 | 0 |
| 259.170429 | 259.170354 | 7609674 | 17 | 24 | 0 | 2 | 0 | 0 |
| 260.105558 | 260.105718 | 1197944 | 11 | 20 | 1 | 4 | 0 | 1 |
| 261.004163 | 261.004076 | 1312803 | 12 | 6 | 0 | 7 | 0 | 0 |
| 261.040464 | 261.040462 | 3373767 | 13 | 10 | 0 | 6 | 0 | 0 |
| 261.076869 | 261.076847 | 7610028 | 14 | 14 | 0 | 5 | 0 | 0 |
| 261.113299 | 261.113233 | 17340976 | 15 | 18 | 0 | 4 | 0 | 0 |
| 261.149635 | 261.149618 | 14448436 | 16 | 22 | 0 | 3 | 0 | 0 |
| 261.186073 | 261.186004 | 3266105 | 17 | 26 | 0 | 2 | 0 | 0 |
| 263.019853 | 263.019726 | 2052628 | 12 | 8 | 0 | 7 | 0 | 0 |
| 263.056162 | 263.056112 | 3171480 | 13 | 12 | 0 | 6 | 0 | 0 |
| 263.092508 | 263.092497 | 10002973 | 14 | 16 | 0 | 5 | 0 | 0 |
| 265.035429 | 265.035376 | 2334503 | 12 | 10 | 0 | 7 | 0 | 0 |
| 265.05053 | 265.050632 | 1295944 | 16 | 10 | 0 | 4 | 0 | 0 |
| 265.071815 | 265.071762 | 5425419 | 13 | 14 | 0 | 6 | 0 | 0 |
| 265.087101 | 265.087018 | 1731533 | 17 | 14 | 0 | 3 | 0 | 0 |
| 265.108211 | 265.108147 | 14990607 | 14 | 18 | 0 | 5 | 0 | 0 |
| 265.123398 | 265.123403 | 3615441 | 18 | 18 | 0 | 2 | 0 | 0 |
| 265.159874 | 265.159789 | 2209814 | 19 | 22 | 0 | 1 | 0 | 0 |
| 265.180999 | 265.180918 | 1633272 | 16 | 26 | 0 | 3 | 0 | 0 |
| 266.094895 | 266.095154 | 1299847 | 13 | 18 | 1 | 3 | 0 | 1 |
| 267.02989 | 267.029897 | 1300408 | 15 | 8 | 0 | 5 | 0 | 0 |
| 267.051027 | 267.051026 | 1658362 | 12 | 12 | 0 | 7 | 0 | 0 |
| 267.066307 | 267.066282 | 2767612 | 16 | 12 | 0 | 4 | 0 | 0 |
| 267.087372 | 267.087412 | 6177023 | 13 | 16 | 0 | 6 | 0 | 0 |
| 267.102715 | 267.102668 | 4538113 | 17 | 16 | 0 | 3 | 0 | 0 |
| 267.123794 | 267.123797 | 11104003 | 14 | 20 | 0 | 5 | 0 | 0 |
| 267.139092 | 267.139053 | 6476933 | 18 | 20 | 0 | 2 | 0 | 0 |
| 267.175475 | 267.175439 | 2768905 | 19 | 24 | 0 | 1 | 0 | 0 |
| 269.045568 | 269.045547 | 3304684 | 15 | 10 | 0 | 5 | 0 | 0 |
| 269.066668 | 269.066676 | 1993262 | 12 | 14 | 0 | 7 | 0 | 0 |
| 269.081987 | 269.081932 | 5715888 | 16 | 14 | 0 | 4 | 0 | 0 |
| 269.103081 | 269.103062 | 6630643 | 13 | 18 | 0 | 6 | 0 | 0 |
| 269.118363 | 269.118318 | 12404724 | 17 | 18 | 0 | 3 | 0 | 0 |
| 269.154739 | 269.154703 | 12898553 | 18 | 22 | 0 | 2 | 0 | 0 |
| 269.19109 | 269.191089 | 3358461 | 19 | 26 | 0 | 1 | 0 | 0 |
| 270.07716 | 270.077181 | 1198632 | 15 | 13 | 1 | 4 | 0 | 0 |
| 271.024876 | 271.024812 | 2521691 | 14 | 8 | 0 | 6 | 0 | 0 |
| 271.061234 | 271.061197 | 5096927 | 15 | 12 | 0 | 5 | 0 | 0 |
| 271.082383 | 271.082326 | 1511458 | 12 | 16 | 0 | 7 | 0 | 0 |
| 271.097606 | 271.097583 | 11154660 | 16 | 16 | 0 | 4 | 0 | 0 |
| 271.118792 | 271.118712 | 2922214 | 13 | 20 | 0 | 6 | 0 | 0 |
| 271.133995 | 271.133968 | 19383016 | 17 | 20 | 0 | 3 | 0 | 0 |
| 271.155018 | 271.155097 | 1333291 | 14 | 24 | 0 | 5 | 0 | 0 |
| 271.170403 | 271.170354 | 12186860 | 18 | 24 | 0 | 2 | 0 | 0 |
| 271.206821 | 271.206739 | 1720817 | 19 | 28 | 0 | 1 | 0 | 0 |
| 272.093061 | 272.092832 | 1228604 | 15 | 15 | 1 | 4 | 0 | 0 |
| 273.00404 | 273.004076 | 1347465 | 13 | 6 | 0 | 7 | 0 | 0 |
| 273.040537 | 273.040462 | 4626254 | 14 | 10 | 0 | 6 | 0 | 0 |
| 273.076901 | 273.076847 | 7841746 | 15 | 14 | 0 | 5 | 0 | 0 |
| 273.113269 | 273.113233 | 19241430 | 16 | 18 | 0 | 4 | 0 | 0 |
| 273.12444 | 273.124466 | 1241784 | 15 | 18 | 2 | 3 | 0 | 0 |
| 273.149655 | 273.149618 | 22211034 | 17 | 22 | 0 | 3 | 0 | 0 |
| 273.186011 | 273.186004 | 7008607 | 18 | 26 | 0 | 2 | 0 | 0 |
| 274.072166 | 274.072096 | 1470474 | 14 | 13 | 1 | 5 | 0 | 0 |
| 274.076984 | 274.076804 | 1330890 | 12 | 13 | 5 | 1 | 1 | 0 |
| 275.019757 | 275.019726 | 2221691 | 13 | 8 | 0 | 7 | 0 | 0 |
| 275.056106 | 275.056112 | 5759679 | 14 | 12 | 0 | 6 | 0 | 0 |
| 275.092495 | 275.092497 | 14942404 | 15 | 16 | 0 | 5 | 0 | 0 |
| 275.12892 | 275.128883 | 24862920 | 16 | 20 | 0 | 4 | 0 | 0 |
| 275.165342 | 275.165268 | 15464140 | 17 | 24 | 0 | 3 | 0 | 0 |
| 275.2017 | 275.201654 | 3161425 | 18 | 28 | 0 | 2 | 0 | 0 |
| 276.087765 | 276.087746 | 1672506 | 14 | 15 | 1 | 5 | 0 | 0 |
| 276.124212 | 276.124132 | 1262974 | 15 | 19 | 1 | 4 | 0 | 0 |
| 277.035417 | 277.035376 | 2577707 | 13 | 10 | 0 | 7 | 0 | 0 |
| 277.07175 | 277.071762 | 6710447 | 14 | 14 | 0 | 6 | 0 | 0 |
| 277.108147 | 277.108147 | 19255732 | 15 | 18 | 0 | 5 | 0 | 0 |
| 277.12338 | 277.123403 | 1999669 | 19 | 18 | 0 | 2 | 0 | 0 |
| 277.159778 | 277.159789 | 1681529 | 20 | 22 | 0 | 1 | 0 | 0 |
| 277.18097 | 277.180918 | 5773500 | 17 | 26 | 0 | 3 | 0 | 0 |
| 279.051035 | 279.051026 | 3295384 | 13 | 12 | 0 | 7 | 0 | 0 |
| 279.066228 | 279.066282 | 2422297 | 17 | 12 | 0 | 4 | 0 | 0 |
| 279.087421 | 279.087412 | 10159004 | 14 | 16 | 0 | 6 | 0 | 0 |
| 279.102719 | 279.102668 | 3419870 | 18 | 16 | 0 | 3 | 0 | 0 |
| 279.139106 | 279.139053 | 5518754 | 19 | 20 | 0 | 2 | 0 | 0 |
| 279.175521 | 279.175439 | 2739878 | 20 | 24 | 0 | 1 | 0 | 0 |
| 279.196577 | 279.196568 | 2080361 | 17 | 28 | 0 | 3 | 0 | 0 |
| 280.097977 | 280.097917 | 1216562 | 17 | 15 | 1 | 3 | 0 | 0 |
| 281.030308 | 281.030291 | 1259965 | 12 | 10 | 0 | 8 | 0 | 0 |
| 281.04558 | 281.045547 | 2472191 | 16 | 10 | 0 | 5 | 0 | 0 |
| 281.066686 | 281.066676 | 3064322 | 13 | 14 | 0 | 7 | 0 | 0 |
| 281.08197 | 281.081932 | 4590595 | 17 | 14 | 0 | 4 | 0 | 0 |
| 281.103083 | 281.103062 | 12708742 | 14 | 18 | 0 | 6 | 0 | 0 |
| 281.118376 | 281.118318 | 7849864 | 18 | 18 | 0 | 3 | 0 | 0 |
| 281.154737 | 281.154703 | 9805708 | 19 | 22 | 0 | 2 | 0 | 0 |
| 281.175837 | 281.175833 | 2959758 | 16 | 26 | 0 | 4 | 0 | 0 |
| 281.191078 | 281.191089 | 3670800 | 20 | 26 | 0 | 1 | 0 | 0 |
| 283.024701 | 283.024812 | 1697185 | 15 | 8 | 0 | 6 | 0 | 0 |
| 283.061192 | 283.061197 | 4209894 | 16 | 12 | 0 | 5 | 0 | 0 |
| 283.082351 | 283.082326 | 3710120 | 13 | 16 | 0 | 7 | 0 | 0 |
| 283.097594 | 283.097583 | 9635945 | 17 | 16 | 0 | 4 | 0 | 0 |
| 283.118675 | 283.118712 | 9877612 | 14 | 20 | 0 | 6 | 0 | 0 |
| 283.13401 | 283.133968 | 15812206 | 18 | 20 | 0 | 3 | 0 | 0 |
| 283.170387 | 283.170354 | 12572786 | 19 | 24 | 0 | 2 | 0 | 0 |
| 283.206742 | 283.206739 | 1727990 | 20 | 28 | 0 | 1 | 0 | 0 |
| 284.129148 | 284.129217 | 1227102 | 17 | 19 | 1 | 3 | 0 | 0 |
| 285.040464 | 285.040462 | 4550979 | 15 | 10 | 0 | 6 | 0 | 0 |
| 285.061659 | 285.061591 | 1285990 | 12 | 14 | 0 | 8 | 0 | 0 |
| 285.076863 | 285.076847 | 8668487 | 16 | 14 | 0 | 5 | 0 | 0 |
| 285.088216 | 285.08808 | 1524809 | 15 | 14 | 2 | 4 | 0 | 0 |
| 285.097974 | 285.097976 | 3334026 | 13 | 18 | 0 | 7 | 0 | 0 |
| 285.113248 | 285.113233 | 19942732 | 17 | 18 | 0 | 4 | 0 | 0 |
| 285.124571 | 285.124466 | 1432333 | 16 | 18 | 2 | 3 | 0 | 0 |
| 285.134368 | 285.134362 | 4754894 | 14 | 22 | 0 | 6 | 0 | 0 |
| 285.149621 | 285.149618 | 28736848 | 18 | 22 | 0 | 3 | 0 | 0 |
| 285.186 | 285.186004 | 11489107 | 19 | 26 | 0 | 2 | 0 | 0 |
| 285.222451 | 285.222389 | 1544343 | 20 | 30 | 0 | 1 | 0 | 0 |
| 286.108437 | 286.108482 | 1524729 | 16 | 17 | 1 | 4 | 0 | 0 |
| 287.019788 | 287.019726 | 2805277 | 14 | 8 | 0 | 7 | 0 | 0 |
| 287.056121 | 287.056112 | 5853217 | 15 | 12 | 0 | 6 | 0 | 0 |
| 287.092493 | 287.092497 | 12923940 | 16 | 16 | 0 | 5 | 0 | 0 |
| 287.103658 | 287.103731 | 1735526 | 15 | 16 | 2 | 4 | 0 | 0 |
| 287.128899 | 287.128883 | 27999784 | 17 | 20 | 0 | 4 | 0 | 0 |
| 287.150154 | 287.150012 | 1462571 | 14 | 24 | 0 | 6 | 0 | 0 |
| 287.165277 | 287.165268 | 23583276 | 18 | 24 | 0 | 3 | 0 | 0 |
| 287.201701 | 287.201654 | 5001648 | 19 | 28 | 0 | 2 | 0 | 0 |
| 288.087824 | 288.087746 | 1590032 | 15 | 15 | 1 | 5 | 0 | 0 |
| 288.124099 | 288.124132 | 1487380 | 16 | 19 | 1 | 4 | 0 | 0 |
| 289.035399 | 289.035376 | 3844853 | 14 | 10 | 0 | 7 | 0 | 0 |
| 289.071768 | 289.071762 | 8000249 | 15 | 14 | 0 | 6 | 0 | 0 |
| 289.108144 | 289.108147 | 19975932 | 16 | 18 | 0 | 5 | 0 | 0 |
| 289.119437 | 289.119381 | 1384382 | 15 | 18 | 2 | 4 | 0 | 0 |
| 289.123382 | 289.123403 | 1523646 | 20 | 18 | 0 | 2 | 0 | 0 |
| 289.144533 | 289.144533 | 29264640 | 17 | 22 | 0 | 4 | 0 | 0 |
| 289.159732 | 289.159789 | 1356290 | 21 | 22 | 0 | 1 | 0 | 0 |
| 289.18092 | 289.180918 | 13330692 | 18 | 26 | 0 | 3 | 0 | 0 |
| 289.217281 | 289.217304 | 2089224 | 19 | 30 | 0 | 2 | 0 | 0 |
| 290.067028 | 290.067011 | 1528098 | 14 | 13 | 1 | 6 | 0 | 0 |
| 290.079622 | 290.079897 | 1258467 | 11 | 18 | 1 | 6 | 0 | 1 |
| 290.103492 | 290.103396 | 1679974 | 15 | 17 | 1 | 5 | 0 | 0 |
| 291.014547 | 291.014641 | 2087493 | 13 | 8 | 0 | 8 | 0 | 0 |
| 291.050985 | 291.051026 | 4736585 | 14 | 12 | 0 | 7 | 0 | 0 |
| 291.066327 | 291.066282 | 1807178 | 18 | 12 | 0 | 4 | 0 | 0 |
| 291.087408 | 291.087412 | 12106188 | 15 | 16 | 0 | 6 | 0 | 0 |
| 291.102778 | 291.102668 | 2561998 | 19 | 16 | 0 | 3 | 0 | 0 |
| 291.123794 | 291.123797 | 29619152 | 16 | 20 | 0 | 5 | 0 | 0 |
| 291.139043 | 291.139053 | 3541714 | 20 | 20 | 0 | 2 | 0 | 0 |
| 291.160177 | 291.160183 | 23831508 | 17 | 24 | 0 | 4 | 0 | 0 |
| 291.17547 | 291.175439 | 1925205 | 21 | 24 | 0 | 1 | 0 | 0 |
| 291.196555 | 291.196568 | 6228312 | 18 | 28 | 0 | 3 | 0 | 0 |
| 292.119035 | 292.119046 | 1253367 | 15 | 19 | 1 | 5 | 0 | 0 |
| 293.030318 | 293.030291 | 1794516 | 13 | 10 | 0 | 8 | 0 | 0 |
| 293.045617 | 293.045547 | 1948310 | 17 | 10 | 0 | 5 | 0 | 0 |
| 293.066633 | 293.066676 | 4182936 | 14 | 14 | 0 | 7 | 0 | 0 |
| 293.08202 | 293.081932 | 2907673 | 18 | 14 | 0 | 4 | 0 | 0 |
| 293.103056 | 293.103062 | 17757340 | 15 | 18 | 0 | 6 | 0 | 0 |
| 293.118342 | 293.118318 | 5137181 | 19 | 18 | 0 | 3 | 0 | 0 |
| 293.139433 | 293.139447 | 24013984 | 16 | 22 | 0 | 5 | 0 | 0 |
| 293.154751 | 293.154703 | 6090401 | 20 | 22 | 0 | 2 | 0 | 0 |
| 293.191113 | 293.191089 | 2697253 | 21 | 26 | 0 | 1 | 0 | 0 |
| 295.024787 | 295.024812 | 1305437 | 16 | 8 | 0 | 6 | 0 | 0 |
| 295.045916 | 295.045941 | 1658303 | 13 | 12 | 0 | 8 | 0 | 0 |
| 295.061241 | 295.061197 | 3718497 | 17 | 12 | 0 | 5 | 0 | 0 |
| 295.082354 | 295.082326 | 5082851 | 14 | 16 | 0 | 7 | 0 | 0 |
| 295.097593 | 295.097583 | 6923621 | 18 | 16 | 0 | 4 | 0 | 0 |
| 295.100519 | 295.100573 | 1325029 | 17 | 17 | 2 | 1 | 0 | 1 |
| 295.118718 | 295.118712 | 20792678 | 15 | 20 | 0 | 6 | 0 | 0 |
| 295.133956 | 295.133968 | 11725672 | 19 | 20 | 0 | 3 | 0 | 0 |
| 295.155102 | 295.155097 | 12518250 | 16 | 24 | 0 | 5 | 0 | 0 |
| 295.170383 | 295.170354 | 9547116 | 20 | 24 | 0 | 2 | 0 | 0 |
| 295.191422 | 295.191483 | 2547310 | 17 | 28 | 0 | 4 | 0 | 0 |
| 295.20671 | 295.206739 | 2062383 | 21 | 28 | 0 | 1 | 0 | 0 |
| 296.069049 | 296.069333 | 1260293 | 13 | 16 | 1 | 5 | 0 | 1 |
| 296.09277 | 296.092832 | 1250952 | 17 | 15 | 1 | 4 | 0 | 0 |
| 296.14186 | 296.142104 | 1308492 | 15 | 24 | 1 | 3 | 0 | 1 |
| 297.040472 | 297.040462 | 3678117 | 16 | 10 | 0 | 6 | 0 | 0 |
| 297.061645 | 297.061591 | 1362215 | 13 | 14 | 0 | 8 | 0 | 0 |
| 297.076826 | 297.076847 | 7276073 | 17 | 14 | 0 | 5 | 0 | 0 |
| 297.097976 | 297.097976 | 7162411 | 14 | 18 | 0 | 7 | 0 | 0 |
| 297.113219 | 297.113233 | 15812140 | 18 | 18 | 0 | 4 | 0 | 0 |
| 297.134413 | 297.134362 | 17338414 | 15 | 22 | 0 | 6 | 0 | 0 |
| 297.149586 | 297.149618 | 22477360 | 19 | 22 | 0 | 3 | 0 | 0 |
| 297.170793 | 297.170747 | 4075826 | 16 | 26 | 0 | 5 | 0 | 0 |
| 297.185967 | 297.186004 | 11956787 | 20 | 26 | 0 | 2 | 0 | 0 |
| 297.222377 | 297.222389 | 2006647 | 21 | 30 | 0 | 1 | 0 | 0 |
| 298.108547 | 298.108482 | 1497805 | 17 | 17 | 1 | 4 | 0 | 0 |
| 298.144736 | 298.144867 | 1354193 | 18 | 21 | 1 | 3 | 0 | 0 |
| 299.019711 | 299.019726 | 2918373 | 15 | 8 | 0 | 7 | 0 | 0 |
| 299.056089 | 299.056112 | 6312937 | 16 | 12 | 0 | 6 | 0 | 0 |
| 299.077248 | 299.077241 | 1589483 | 13 | 16 | 0 | 8 | 0 | 0 |
| 299.092494 | 299.092497 | 11839212 | 17 | 16 | 0 | 5 | 0 | 0 |
| 299.095769 | 299.095868 | 1543149 | 14 | 20 | 0 | 5 | 1 | 0 |
| 299.103665 | 299.103731 | 1658350 | 16 | 16 | 2 | 4 | 0 | 0 |
| 299.11365 | 299.113627 | 4958831 | 14 | 20 | 0 | 7 | 0 | 0 |
| 299.128884 | 299.128883 | 27423472 | 18 | 20 | 0 | 4 | 0 | 0 |
| 299.149992 | 299.150012 | 7059954 | 15 | 24 | 0 | 6 | 0 | 0 |
| 299.165285 | 299.165268 | 26479348 | 19 | 24 | 0 | 3 | 0 | 0 |
| 300.087697 | 300.087746 | 1398316 | 16 | 15 | 1 | 5 | 0 | 0 |
| 300.124161 | 300.124132 | 1712207 | 17 | 19 | 1 | 4 | 0 | 0 |
| 301.035393 | 301.035376 | 4673702 | 15 | 10 | 0 | 7 | 0 | 0 |
| 301.071768 | 301.071762 | 7580842 | 16 | 14 | 0 | 6 | 0 | 0 |
| 301.108166 | 301.108147 | 19774382 | 17 | 18 | 0 | 5 | 0 | 0 |
| 301.119476 | 301.119381 | 1507886 | 16 | 18 | 2 | 4 | 0 | 0 |
| 301.129237 | 301.129277 | 1895727 | 14 | 22 | 0 | 7 | 0 | 0 |
| 301.144538 | 301.144533 | 33536944 | 18 | 22 | 0 | 4 | 0 | 0 |
| 301.165652 | 301.165662 | 1856179 | 15 | 26 | 0 | 6 | 0 | 0 |
| 301.180922 | 301.180918 | 19531700 | 19 | 26 | 0 | 3 | 0 | 0 |
| 301.217356 | 301.217304 | 4393144 | 20 | 30 | 0 | 2 | 0 | 0 |
| 302.103339 | 302.103396 | 1756555 | 16 | 17 | 1 | 5 | 0 | 0 |
| 302.108387 | 302.108104 | 1457036 | 14 | 17 | 5 | 1 | 1 | 0 |
| 302.139762 | 302.139782 | 2016271 | 17 | 21 | 1 | 4 | 0 | 0 |
| 303.014628 | 303.014641 | 2170657 | 14 | 8 | 0 | 8 | 0 | 0 |
| 303.051034 | 303.051026 | 5627748 | 15 | 12 | 0 | 7 | 0 | 0 |
| 303.087406 | 303.087412 | 12325480 | 16 | 16 | 0 | 6 | 0 | 0 |
| 303.09875 | 303.098645 | 1548777 | 15 | 16 | 2 | 5 | 0 | 0 |
| 303.102685 | 303.102668 | 1622761 | 20 | 16 | 0 | 3 | 0 | 0 |
| 303.123779 | 303.123797 | 31261804 | 17 | 20 | 0 | 5 | 0 | 0 |
| 303.139088 | 303.139053 | 2371437 | 21 | 20 | 0 | 2 | 0 | 0 |
| 303.160162 | 303.160183 | 34395248 | 18 | 24 | 0 | 4 | 0 | 0 |
| 303.175489 | 303.175439 | 1514480 | 22 | 24 | 0 | 1 | 0 | 0 |
| 303.196543 | 303.196568 | 11017330 | 19 | 28 | 0 | 3 | 0 | 0 |
| 304.082713 | 304.082661 | 1478661 | 15 | 15 | 1 | 6 | 0 | 0 |
| 304.119145 | 304.119046 | 1663688 | 16 | 19 | 1 | 5 | 0 | 0 |
| 304.155364 | 304.155432 | 1499499 | 17 | 23 | 1 | 4 | 0 | 0 |
| 305.030331 | 305.030291 | 2918428 | 14 | 10 | 0 | 8 | 0 | 0 |
| 305.045506 | 305.045547 | 1601054 | 18 | 10 | 0 | 5 | 0 | 0 |
| 305.066646 | 305.066676 | 7537952 | 15 | 14 | 0 | 7 | 0 | 0 |
| 305.082017 | 305.081932 | 1913313 | 19 | 14 | 0 | 4 | 0 | 0 |
| 305.103035 | 305.103062 | 20274466 | 16 | 18 | 0 | 6 | 0 | 0 |
| 305.118241 | 305.118318 | 3333924 | 20 | 18 | 0 | 3 | 0 | 0 |
| 305.138271 | 305.138415 | 1790758 | 11 | 23 | 4 | 4 | 0 | 1 |
| 305.139448 | 305.139447 | 37625128 | 17 | 22 | 0 | 5 | 0 | 0 |
| 305.154696 | 305.154703 | 4084264 | 21 | 22 | 0 | 2 | 0 | 0 |
| 305.175852 | 305.175833 | 22055210 | 18 | 26 | 0 | 4 | 0 | 0 |
| 305.191089 | 305.191089 | 2113387 | 22 | 26 | 0 | 1 | 0 | 0 |
| 307.045883 | 307.045941 | 2890837 | 14 | 12 | 0 | 8 | 0 | 0 |
| 307.061228 | 307.061197 | 2826071 | 18 | 12 | 0 | 5 | 0 | 0 |
| 307.082326 | 307.082326 | 7640537 | 15 | 16 | 0 | 7 | 0 | 0 |
| 307.097556 | 307.097583 | 4578010 | 19 | 16 | 0 | 4 | 0 | 0 |
| 307.118701 | 307.118712 | 25447900 | 16 | 20 | 0 | 6 | 0 | 0 |
| 307.133937 | 307.133968 | 7127518 | 20 | 20 | 0 | 3 | 0 | 0 |
| 307.170325 | 307.170354 | 7434721 | 21 | 24 | 0 | 2 | 0 | 0 |
| 307.206757 | 307.206739 | 2593892 | 22 | 28 | 0 | 1 | 0 | 0 |
| 307.227956 | 307.227868 | 1362726 | 19 | 32 | 0 | 3 | 0 | 0 |
| 308.105489 | 308.105718 | 1298102 | 15 | 20 | 1 | 4 | 0 | 1 |
| 309.040488 | 309.040462 | 3224459 | 17 | 10 | 0 | 6 | 0 | 0 |
| 309.061544 | 309.061591 | 2928268 | 14 | 14 | 0 | 8 | 0 | 0 |
| 309.076829 | 309.076847 | 5055374 | 18 | 14 | 0 | 5 | 0 | 0 |
| 309.097968 | 309.097976 | 10723984 | 15 | 18 | 0 | 7 | 0 | 0 |
| 309.113234 | 309.113233 | 9454737 | 19 | 18 | 0 | 4 | 0 | 0 |
| 309.134366 | 309.134362 | 21894802 | 16 | 22 | 0 | 6 | 0 | 0 |
| 309.149604 | 309.149618 | 14225044 | 20 | 22 | 0 | 3 | 0 | 0 |
| 309.186022 | 309.186004 | 9309848 | 21 | 26 | 0 | 2 | 0 | 0 |
| 309.222423 | 309.222389 | 2896795 | 22 | 30 | 0 | 1 | 0 | 0 |
| 310.108489 | 310.108482 | 1474795 | 18 | 17 | 1 | 4 | 0 | 0 |
| 311.019869 | 311.019726 | 1868733 | 16 | 8 | 0 | 7 | 0 | 0 |
| 311.056094 | 311.056112 | 6447936 | 17 | 12 | 0 | 6 | 0 | 0 |
| 311.077254 | 311.077241 | 3056962 | 14 | 16 | 0 | 8 | 0 | 0 |
| 311.092476 | 311.092497 | 9996099 | 18 | 16 | 0 | 5 | 0 | 0 |
| 311.103679 | 311.103731 | 1844548 | 17 | 16 | 2 | 4 | 0 | 0 |
| 311.113648 | 311.113627 | 13187909 | 15 | 20 | 0 | 7 | 0 | 0 |
| 311.128844 | 311.128883 | 20902726 | 19 | 20 | 0 | 4 | 0 | 0 |
| 311.150035 | 311.150012 | 12326728 | 16 | 24 | 0 | 6 | 0 | 0 |
| 311.165284 | 311.165268 | 21971786 | 20 | 24 | 0 | 3 | 0 | 0 |
| 311.186393 | 311.186398 | 3634380 | 17 | 28 | 0 | 5 | 0 | 0 |
| 311.201681 | 311.201654 | 9761101 | 21 | 28 | 0 | 2 | 0 | 0 |
| 311.237999 | 311.238039 | 1419952 | 22 | 32 | 0 | 1 | 0 | 0 |
| 312.05137 | 312.051361 | 1404697 | 16 | 11 | 1 | 6 | 0 | 0 |
| 312.087701 | 312.087746 | 1858972 | 17 | 15 | 1 | 5 | 0 | 0 |
| 312.12412 | 312.124132 | 1588767 | 18 | 19 | 1 | 4 | 0 | 0 |
| 313.035348 | 313.035376 | 4719089 | 16 | 10 | 0 | 7 | 0 | 0 |
| 313.071751 | 313.071762 | 8230388 | 17 | 14 | 0 | 6 | 0 | 0 |
| 313.092845 | 313.092891 | 3065206 | 14 | 18 | 0 | 8 | 0 | 0 |
| 313.10812 | 313.108147 | 18647030 | 18 | 18 | 0 | 5 | 0 | 0 |
| 313.111486 | 313.111518 | 1604023 | 15 | 22 | 0 | 5 | 1 | 0 |
| 313.119497 | 313.119381 | 1817848 | 17 | 18 | 2 | 4 | 0 | 0 |
| 313.129288 | 313.129277 | 8597241 | 15 | 22 | 0 | 7 | 0 | 0 |
| 313.144509 | 313.144533 | 34838524 | 19 | 22 | 0 | 4 | 0 | 0 |
| 313.165669 | 313.165662 | 4493820 | 16 | 26 | 0 | 6 | 0 | 0 |
| 313.180906 | 313.180918 | 25425918 | 20 | 26 | 0 | 3 | 0 | 0 |
| 313.217325 | 313.217304 | 6820353 | 21 | 30 | 0 | 2 | 0 | 0 |
| 314.067024 | 314.067011 | 1392108 | 16 | 13 | 1 | 6 | 0 | 0 |
| 314.103377 | 314.103396 | 2038863 | 17 | 17 | 1 | 5 | 0 | 0 |
| 314.139778 | 314.139782 | 1921106 | 18 | 21 | 1 | 4 | 0 | 0 |
| 314.176158 | 314.176167 | 1390294 | 19 | 25 | 1 | 3 | 0 | 0 |
| 315.014618 | 315.014641 | 2203552 | 15 | 8 | 0 | 8 | 0 | 0 |
| 315.050999 | 315.051026 | 6685347 | 16 | 12 | 0 | 7 | 0 | 0 |
| 315.087426 | 315.087412 | 12010662 | 17 | 16 | 0 | 6 | 0 | 0 |
| 315.098611 | 315.098645 | 1672615 | 16 | 16 | 2 | 5 | 0 | 0 |
| 315.108451 | 315.108541 | 1316648 | 14 | 20 | 0 | 8 | 0 | 0 |
| 315.123802 | 315.123797 | 29841578 | 18 | 20 | 0 | 5 | 0 | 0 |
| 315.12723 | 315.127168 | 2048234 | 15 | 24 | 0 | 5 | 1 | 0 |
| 315.144909 | 315.144927 | 2633388 | 15 | 24 | 0 | 7 | 0 | 0 |
| 315.160171 | 315.160183 | 37935276 | 19 | 24 | 0 | 4 | 0 | 0 |
| 315.196556 | 315.196568 | 15726768 | 20 | 28 | 0 | 3 | 0 | 0 |
| 315.232988 | 315.232954 | 2100403 | 21 | 32 | 0 | 2 | 0 | 0 |
| 316.095325 | 316.095547 | 1602303 | 13 | 20 | 1 | 6 | 0 | 1 |
| 316.119 | 316.119046 | 2284418 | 17 | 19 | 1 | 5 | 0 | 0 |
| 316.155496 | 316.155432 | 1755141 | 18 | 23 | 1 | 4 | 0 | 0 |
| 317.03031 | 317.030291 | 3678546 | 15 | 10 | 0 | 8 | 0 | 0 |
| 317.066667 | 317.066676 | 7000149 | 16 | 14 | 0 | 7 | 0 | 0 |
| 317.08188 | 317.081932 | 1585750 | 20 | 14 | 0 | 4 | 0 | 0 |
| 317.103021 | 317.103062 | 17313112 | 17 | 18 | 0 | 6 | 0 | 0 |
| 317.114262 | 317.114295 | 2041305 | 16 | 18 | 2 | 5 | 0 | 0 |
| 317.118356 | 317.118318 | 2031194 | 21 | 18 | 0 | 3 | 0 | 0 |
| 317.139432 | 317.139447 | 40030556 | 18 | 22 | 0 | 5 | 0 | 0 |
| 317.154661 | 317.154703 | 2164573 | 22 | 22 | 0 | 2 | 0 | 0 |
| 317.175843 | 317.175833 | 29509982 | 19 | 26 | 0 | 4 | 0 | 0 |
| 318.098318 | 318.098311 | 1353520 | 16 | 17 | 1 | 6 | 0 | 0 |
| 318.134641 | 318.134696 | 1886899 | 17 | 21 | 1 | 5 | 0 | 0 |
| 318.171122 | 318.171082 | 1335286 | 18 | 25 | 1 | 4 | 0 | 0 |
| 319.009452 | 319.009555 | 1291200 | 14 | 8 | 0 | 9 | 0 | 0 |
| 319.045922 | 319.045941 | 3348163 | 15 | 12 | 0 | 8 | 0 | 0 |
| 319.061118 | 319.061197 | 1684996 | 19 | 12 | 0 | 5 | 0 | 0 |
| 319.082316 | 319.082326 | 8764422 | 16 | 16 | 0 | 7 | 0 | 0 |
| 319.097668 | 319.097583 | 2768392 | 20 | 16 | 0 | 4 | 0 | 0 |
| 319.118727 | 319.118712 | 27542026 | 17 | 20 | 0 | 6 | 0 | 0 |
| 319.129901 | 319.129945 | 1270218 | 16 | 20 | 2 | 5 | 0 | 0 |
| 319.133962 | 319.133968 | 4639755 | 21 | 20 | 0 | 3 | 0 | 0 |
| 319.170373 | 319.170354 | 4746766 | 22 | 24 | 0 | 2 | 0 | 0 |
| 319.191451 | 319.191483 | 16668176 | 19 | 28 | 0 | 4 | 0 | 0 |
| 319.206785 | 319.206739 | 2045329 | 23 | 28 | 0 | 1 | 0 | 0 |
| 320.150358 | 320.150346 | 1375908 | 17 | 23 | 1 | 5 | 0 | 0 |
| 321.025154 | 321.025205 | 1690417 | 14 | 10 | 0 | 9 | 0 | 0 |
| 321.040384 | 321.040462 | 2205618 | 18 | 10 | 0 | 6 | 0 | 0 |
| 321.061567 | 321.061591 | 3786420 | 15 | 14 | 0 | 8 | 0 | 0 |
| 321.076827 | 321.076847 | 4254134 | 19 | 14 | 0 | 5 | 0 | 0 |
| 321.098007 | 321.097976 | 13561527 | 16 | 18 | 0 | 7 | 0 | 0 |
| 321.113264 | 321.113233 | 6425785 | 20 | 18 | 0 | 4 | 0 | 0 |
| 321.134363 | 321.134362 | 36848316 | 17 | 22 | 0 | 6 | 0 | 0 |
| 321.14965 | 321.149618 | 9338556 | 21 | 22 | 0 | 3 | 0 | 0 |
| 321.17072 | 321.170747 | 28369598 | 18 | 26 | 0 | 5 | 0 | 0 |
| 321.185984 | 321.186004 | 7565503 | 22 | 26 | 0 | 2 | 0 | 0 |
| 321.222339 | 321.222389 | 2469826 | 23 | 30 | 0 | 1 | 0 | 0 |
| 322.048354 | 322.048597 | 1272843 | 14 | 14 | 1 | 6 | 0 | 1 |
| 322.072289 | 322.072096 | 1268749 | 18 | 13 | 1 | 5 | 0 | 0 |
| 323.019737 | 323.019726 | 1863776 | 17 | 8 | 0 | 7 | 0 | 0 |
| 323.040837 | 323.040856 | 1663970 | 14 | 12 | 0 | 9 | 0 | 0 |
| 323.056152 | 323.056112 | 4588131 | 18 | 12 | 0 | 6 | 0 | 0 |
| 323.077197 | 323.077241 | 4138085 | 15 | 16 | 0 | 8 | 0 | 0 |
| 323.092481 | 323.092497 | 7319399 | 19 | 16 | 0 | 5 | 0 | 0 |
| 323.11363 | 323.113627 | 19720040 | 16 | 20 | 0 | 7 | 0 | 0 |
| 323.128867 | 323.128883 | 12842858 | 20 | 20 | 0 | 4 | 0 | 0 |
| 323.149991 | 323.150012 | 30030700 | 17 | 24 | 0 | 6 | 0 | 0 |
| 323.165278 | 323.165268 | 16881516 | 21 | 24 | 0 | 3 | 0 | 0 |
| 323.201639 | 323.201654 | 9388400 | 22 | 28 | 0 | 2 | 0 | 0 |
| 323.222744 | 323.222783 | 2521458 | 19 | 32 | 0 | 4 | 0 | 0 |
| 323.238003 | 323.238039 | 1994483 | 23 | 32 | 0 | 1 | 0 | 0 |
| 324.06402 | 324.064247 | 1356348 | 14 | 16 | 1 | 6 | 0 | 1 |
| 324.124172 | 324.124132 | 1268353 | 19 | 19 | 1 | 4 | 0 | 0 |
| 324.136762 | 324.137018 | 1510722 | 16 | 24 | 1 | 4 | 0 | 1 |
| 324.160429 | 324.160517 | 1250116 | 20 | 23 | 1 | 3 | 0 | 0 |
| 325.035353 | 325.035376 | 4057105 | 17 | 10 | 0 | 7 | 0 | 0 |
| 325.071801 | 325.071762 | 6241557 | 18 | 14 | 0 | 6 | 0 | 0 |
| 325.092907 | 325.092891 | 5578775 | 15 | 18 | 0 | 8 | 0 | 0 |
| 325.108125 | 325.108147 | 12389400 | 19 | 18 | 0 | 5 | 0 | 0 |
| 325.129273 | 325.129277 | 17361946 | 16 | 22 | 0 | 7 | 0 | 0 |
| 325.144519 | 325.144533 | 21038106 | 20 | 22 | 0 | 4 | 0 | 0 |
| 325.165666 | 325.165662 | 12275741 | 17 | 26 | 0 | 6 | 0 | 0 |
| 325.180909 | 325.180918 | 19878942 | 21 | 26 | 0 | 3 | 0 | 0 |
| 325.217324 | 325.217304 | 7864866 | 22 | 30 | 0 | 2 | 0 | 0 |
| 326.103395 | 326.103396 | 1861103 | 18 | 17 | 1 | 5 | 0 | 0 |
| 326.13981 | 326.139782 | 1817843 | 19 | 21 | 1 | 4 | 0 | 0 |
| 327.014663 | 327.014641 | 2083264 | 16 | 8 | 0 | 8 | 0 | 0 |
| 327.051048 | 327.051026 | 6138051 | 17 | 12 | 0 | 7 | 0 | 0 |
| 327.072116 | 327.072156 | 1259589 | 14 | 16 | 0 | 9 | 0 | 0 |
| 327.087376 | 327.087412 | 9593030 | 18 | 16 | 0 | 6 | 0 | 0 |
| 327.090762 | 327.090783 | 1560007 | 15 | 20 | 0 | 6 | 1 | 0 |
| 327.098702 | 327.098645 | 1509703 | 17 | 16 | 2 | 5 | 0 | 0 |
| 327.108535 | 327.108541 | 5393608 | 15 | 20 | 0 | 8 | 0 | 0 |
| 327.123777 | 327.123797 | 23056586 | 19 | 20 | 0 | 5 | 0 | 0 |
| 327.134964 | 327.135031 | 1497675 | 18 | 20 | 2 | 4 | 0 | 0 |
| 327.144947 | 327.144927 | 7638220 | 16 | 24 | 0 | 7 | 0 | 0 |
| 327.160198 | 327.160183 | 31982796 | 20 | 24 | 0 | 4 | 0 | 0 |
| 327.181234 | 327.181312 | 3516111 | 17 | 28 | 0 | 6 | 0 | 0 |
| 327.196564 | 327.196568 | 17602768 | 21 | 28 | 0 | 3 | 0 | 0 |
| 327.232879 | 327.232954 | 4282067 | 22 | 32 | 0 | 2 | 0 | 0 |
| 328.082645 | 328.082661 | 1514783 | 17 | 15 | 1 | 6 | 0 | 0 |
| 328.119019 | 328.119046 | 1857186 | 18 | 19 | 1 | 5 | 0 | 0 |
| 328.131616 | 328.131933 | 1283491 | 15 | 24 | 1 | 5 | 0 | 1 |
| 328.15537 | 328.155432 | 1869605 | 19 | 23 | 1 | 4 | 0 | 0 |
| 329.030282 | 329.030291 | 3640691 | 16 | 10 | 0 | 8 | 0 | 0 |
| 329.06666 | 329.066676 | 7888758 | 17 | 14 | 0 | 7 | 0 | 0 |
| 329.103025 | 329.103062 | 16885114 | 18 | 18 | 0 | 6 | 0 | 0 |
| 329.10639 | 329.106433 | 3203705 | 15 | 22 | 0 | 6 | 1 | 0 |
| 329.114251 | 329.114295 | 2228090 | 17 | 18 | 2 | 5 | 0 | 0 |
| 329.124186 | 329.124191 | 2448635 | 15 | 22 | 0 | 8 | 0 | 0 |
| 329.139473 | 329.139447 | 37281148 | 19 | 22 | 0 | 5 | 0 | 0 |
| 329.154666 | 329.154703 | 1667710 | 23 | 22 | 0 | 2 | 0 | 0 |
| 329.160593 | 329.160577 | 2258046 | 16 | 26 | 0 | 7 | 0 | 0 |
| 329.175832 | 329.175833 | 33160576 | 20 | 26 | 0 | 4 | 0 | 0 |
| 329.212194 | 329.212218 | 10741123 | 21 | 30 | 0 | 3 | 0 | 0 |
| 329.248528 | 329.248604 | 1706630 | 22 | 34 | 0 | 2 | 0 | 0 |
| 330.098297 | 330.098311 | 1633362 | 17 | 17 | 1 | 6 | 0 | 0 |
| 330.134753 | 330.134696 | 2252757 | 18 | 21 | 1 | 5 | 0 | 0 |
| 330.171036 | 330.171082 | 1314264 | 19 | 25 | 1 | 4 | 0 | 0 |
| 331.009556 | 331.009555 | 2046115 | 15 | 8 | 0 | 9 | 0 | 0 |
| 331.045959 | 331.045941 | 4912423 | 16 | 12 | 0 | 8 | 0 | 0 |
| 331.061165 | 331.061197 | 1508648 | 20 | 12 | 0 | 5 | 0 | 0 |
| 331.082304 | 331.082326 | 11185706 | 17 | 16 | 0 | 7 | 0 | 0 |
| 331.093491 | 331.09356 | 1525931 | 16 | 16 | 2 | 6 | 0 | 0 |
| 331.09759 | 331.097583 | 1912747 | 21 | 16 | 0 | 4 | 0 | 0 |
| 331.11868 | 331.118712 | 28511790 | 18 | 20 | 0 | 6 | 0 | 0 |
| 331.122017 | 331.122083 | 3742509 | 15 | 24 | 0 | 6 | 1 | 0 |
| 331.133928 | 331.133968 | 2491694 | 22 | 20 | 0 | 3 | 0 | 0 |
| 331.1551 | 331.155097 | 47775280 | 19 | 24 | 0 | 5 | 0 | 0 |
| 331.170308 | 331.170354 | 2469938 | 23 | 24 | 0 | 2 | 0 | 0 |
| 331.191504 | 331.191483 | 25265716 | 20 | 28 | 0 | 4 | 0 | 0 |
| 331.227828 | 331.227868 | 4092471 | 21 | 32 | 0 | 3 | 0 | 0 |
| 332.077643 | 332.077575 | 1427203 | 16 | 15 | 1 | 7 | 0 | 0 |
| 332.114008 | 332.113961 | 2231687 | 17 | 19 | 1 | 6 | 0 | 0 |
| 332.150364 | 332.150346 | 2197642 | 18 | 23 | 1 | 5 | 0 | 0 |
| 332.186659 | 332.186732 | 1301005 | 19 | 27 | 1 | 4 | 0 | 0 |
| 333.025193 | 333.025205 | 2168281 | 15 | 10 | 0 | 9 | 0 | 0 |
| 333.040503 | 333.040462 | 1844826 | 19 | 10 | 0 | 6 | 0 | 0 |
| 333.061574 | 333.061591 | 5995228 | 16 | 14 | 0 | 8 | 0 | 0 |
| 333.076836 | 333.076847 | 2877917 | 20 | 14 | 0 | 5 | 0 | 0 |
| 333.097959 | 333.097976 | 14486239 | 17 | 18 | 0 | 7 | 0 | 0 |
| 333.109054 | 333.10921 | 1309792 | 16 | 18 | 2 | 6 | 0 | 0 |
| 333.113199 | 333.113233 | 4129505 | 21 | 18 | 0 | 4 | 0 | 0 |
| 333.134367 | 333.134362 | 42122980 | 18 | 22 | 0 | 6 | 0 | 0 |
| 333.137729 | 333.137733 | 2862307 | 15 | 26 | 0 | 6 | 1 | 0 |
| 333.149632 | 333.149618 | 5882596 | 22 | 22 | 0 | 3 | 0 | 0 |
| 333.170716 | 333.170747 | 42839780 | 19 | 26 | 0 | 5 | 0 | 0 |
| 333.185999 | 333.186004 | 4697319 | 23 | 26 | 0 | 2 | 0 | 0 |
| 333.222254 | 333.222389 | 1762794 | 24 | 30 | 0 | 1 | 0 | 0 |
| 333.243385 | 333.243519 | 1393516 | 21 | 34 | 0 | 3 | 0 | 0 |
| 334.048383 | 334.048597 | 1292213 | 15 | 14 | 1 | 6 | 0 | 1 |
| 334.093151 | 334.093225 | 1339833 | 16 | 17 | 1 | 7 | 0 | 0 |
| 334.129592 | 334.129611 | 1845949 | 17 | 21 | 1 | 6 | 0 | 0 |
| 334.165979 | 334.165996 | 1475264 | 18 | 25 | 1 | 5 | 0 | 0 |
| 335.019646 | 335.019726 | 1495950 | 18 | 8 | 0 | 7 | 0 | 0 |
| 335.040792 | 335.040856 | 2120272 | 15 | 12 | 0 | 9 | 0 | 0 |
| 335.05611 | 335.056112 | 3651473 | 19 | 12 | 0 | 6 | 0 | 0 |
| 335.077194 | 335.077241 | 4816531 | 16 | 16 | 0 | 8 | 0 | 0 |
| 335.092489 | 335.092497 | 4820629 | 20 | 16 | 0 | 5 | 0 | 0 |
| 335.11364 | 335.113627 | 21240726 | 17 | 20 | 0 | 7 | 0 | 0 |
| 335.128906 | 335.128883 | 7709080 | 21 | 20 | 0 | 4 | 0 | 0 |
| 335.149986 | 335.150012 | 44228504 | 18 | 24 | 0 | 6 | 0 | 0 |
| 335.165255 | 335.165268 | 10227099 | 22 | 24 | 0 | 3 | 0 | 0 |
| 335.201606 | 335.201654 | 6042015 | 23 | 28 | 0 | 2 | 0 | 0 |
| 335.238072 | 335.238039 | 1818146 | 24 | 32 | 0 | 1 | 0 | 0 |
| 336.108921 | 336.108876 | 1303922 | 16 | 19 | 1 | 7 | 0 | 0 |
| 336.145307 | 336.145261 | 1304693 | 17 | 23 | 1 | 6 | 0 | 0 |
| 337.035361 | 337.035376 | 3118407 | 18 | 10 | 0 | 7 | 0 | 0 |
| 337.056465 | 337.056506 | 1394313 | 15 | 14 | 0 | 9 | 0 | 0 |
| 337.071776 | 337.071762 | 5622090 | 19 | 14 | 0 | 6 | 0 | 0 |
| 337.092862 | 337.092891 | 7202892 | 16 | 18 | 0 | 8 | 0 | 0 |
| 337.108106 | 337.108147 | 8148558 | 20 | 18 | 0 | 5 | 0 | 0 |
| 337.129296 | 337.129277 | 29077584 | 17 | 22 | 0 | 7 | 0 | 0 |
| 337.144523 | 337.144533 | 15574097 | 21 | 22 | 0 | 4 | 0 | 0 |
| 337.180927 | 337.180918 | 16232532 | 22 | 26 | 0 | 3 | 0 | 0 |
| 337.217282 | 337.217304 | 7999576 | 23 | 30 | 0 | 2 | 0 | 0 |
| 338.067006 | 338.067011 | 1392166 | 18 | 13 | 1 | 6 | 0 | 0 |
| 338.103383 | 338.103396 | 1820458 | 19 | 17 | 1 | 5 | 0 | 0 |
| 338.139743 | 338.139782 | 1440813 | 20 | 21 | 1 | 4 | 0 | 0 |
| 339.014685 | 339.014641 | 2060287 | 17 | 8 | 0 | 8 | 0 | 0 |
| 339.051039 | 339.051026 | 5691138 | 18 | 12 | 0 | 7 | 0 | 0 |
| 339.07213 | 339.072156 | 2156676 | 15 | 16 | 0 | 9 | 0 | 0 |
| 339.087402 | 339.087412 | 7838982 | 19 | 16 | 0 | 6 | 0 | 0 |
| 339.108532 | 339.108541 | 11015432 | 16 | 20 | 0 | 8 | 0 | 0 |
| 339.123808 | 339.123797 | 15463689 | 20 | 20 | 0 | 5 | 0 | 0 |
| 339.160156 | 339.160183 | 24445196 | 21 | 24 | 0 | 4 | 0 | 0 |
| 339.196549 | 339.196568 | 18906384 | 22 | 28 | 0 | 3 | 0 | 0 |
| 339.232958 | 339.232954 | 5889300 | 23 | 32 | 0 | 2 | 0 | 0 |
| 339.254117 | 339.254083 | 1322773 | 20 | 36 | 0 | 4 | 0 | 0 |
| 340.11908 | 340.119046 | 1637863 | 19 | 19 | 1 | 5 | 0 | 0 |
| 340.155405 | 340.155432 | 1976810 | 20 | 23 | 1 | 4 | 0 | 0 |
| 341.030327 | 341.030291 | 3786685 | 17 | 10 | 0 | 8 | 0 | 0 |
| 341.066653 | 341.066676 | 7063489 | 18 | 14 | 0 | 7 | 0 | 0 |
| 341.069967 | 341.070047 | 1522881 | 15 | 18 | 0 | 7 | 1 | 0 |
| 341.087805 | 341.087806 | 2246594 | 15 | 18 | 0 | 9 | 0 | 0 |
| 341.103042 | 341.103062 | 13598148 | 19 | 18 | 0 | 6 | 0 | 0 |
| 341.124138 | 341.124191 | 8432070 | 16 | 22 | 0 | 8 | 0 | 0 |
| 341.13944 | 341.139447 | 28664264 | 20 | 22 | 0 | 5 | 0 | 0 |
| 341.160584 | 341.160577 | 8898505 | 17 | 26 | 0 | 7 | 0 | 0 |
| 341.175807 | 341.175833 | 30163402 | 21 | 26 | 0 | 4 | 0 | 0 |
| 341.212225 | 341.212218 | 14728654 | 22 | 30 | 0 | 3 | 0 | 0 |
| 341.248531 | 341.248604 | 2718930 | 23 | 34 | 0 | 2 | 0 | 0 |
| 342.098366 | 342.098311 | 1712419 | 18 | 17 | 1 | 6 | 0 | 0 |
| 342.134759 | 342.134696 | 2241062 | 19 | 21 | 1 | 5 | 0 | 0 |
| 342.171045 | 342.171082 | 1725354 | 20 | 25 | 1 | 4 | 0 | 0 |
| 343.009484 | 343.009555 | 1570810 | 16 | 8 | 0 | 9 | 0 | 0 |
| 343.045958 | 343.045941 | 5155454 | 17 | 12 | 0 | 8 | 0 | 0 |
| 343.082315 | 343.082326 | 9152641 | 18 | 16 | 0 | 7 | 0 | 0 |
| 343.085747 | 343.085697 | 2309666 | 15 | 20 | 0 | 7 | 1 | 0 |
| 343.093571 | 343.09356 | 1595010 | 17 | 16 | 2 | 6 | 0 | 0 |
| 343.103585 | 343.103456 | 1404035 | 15 | 20 | 0 | 9 | 0 | 0 |
| 343.118671 | 343.118712 | 23757444 | 19 | 20 | 0 | 6 | 0 | 0 |
| 343.133907 | 343.133968 | 1456710 | 23 | 20 | 0 | 3 | 0 | 0 |
| 343.139796 | 343.139841 | 3481223 | 16 | 24 | 0 | 8 | 0 | 0 |
| 343.155114 | 343.155097 | 39811720 | 20 | 24 | 0 | 5 | 0 | 0 |
| 343.170324 | 343.170354 | 1756810 | 24 | 24 | 0 | 2 | 0 | 0 |
| 343.176178 | 343.176227 | 2399114 | 17 | 28 | 0 | 7 | 0 | 0 |
| 343.19146 | 343.191483 | 26153612 | 21 | 28 | 0 | 4 | 0 | 0 |
| 343.227865 | 343.227868 | 7462544 | 22 | 32 | 0 | 3 | 0 | 0 |
| 344.077644 | 344.077575 | 1271010 | 17 | 15 | 1 | 7 | 0 | 0 |
| 344.113971 | 344.113961 | 1861861 | 18 | 19 | 1 | 6 | 0 | 0 |
| 344.150466 | 344.150346 | 2448617 | 19 | 23 | 1 | 5 | 0 | 0 |
| 344.186653 | 344.186732 | 1557868 | 20 | 27 | 1 | 4 | 0 | 0 |
| 345.025171 | 345.025205 | 2547262 | 16 | 10 | 0 | 9 | 0 | 0 |
| 345.040503 | 345.040462 | 1400896 | 20 | 10 | 0 | 6 | 0 | 0 |
| 345.06162 | 345.061591 | 6188354 | 17 | 14 | 0 | 8 | 0 | 0 |
| 345.076833 | 345.076847 | 1920067 | 21 | 14 | 0 | 5 | 0 | 0 |
| 345.097939 | 345.097976 | 14312261 | 18 | 18 | 0 | 7 | 0 | 0 |
| 345.101262 | 345.101347 | 3603782 | 15 | 22 | 0 | 7 | 1 | 0 |
| 345.109263 | 345.10921 | 1651399 | 17 | 18 | 2 | 6 | 0 | 0 |
| 345.113232 | 345.113233 | 2981703 | 22 | 18 | 0 | 4 | 0 | 0 |
| 345.134376 | 345.134362 | 38552392 | 19 | 22 | 0 | 6 | 0 | 0 |
| 345.149571 | 345.149618 | 2860875 | 23 | 22 | 0 | 3 | 0 | 0 |
| 345.186043 | 345.186004 | 2909006 | 24 | 26 | 0 | 2 | 0 | 0 |
| 345.207126 | 345.207133 | 15579984 | 21 | 30 | 0 | 4 | 0 | 0 |
| 345.243526 | 345.243519 | 2952788 | 22 | 34 | 0 | 3 | 0 | 0 |
| 346.129548 | 346.129611 | 2396715 | 18 | 21 | 1 | 6 | 0 | 0 |
| 346.165962 | 346.165996 | 1891119 | 19 | 25 | 1 | 5 | 0 | 0 |
| 346.202437 | 346.202382 | 1691570 | 20 | 29 | 1 | 4 | 0 | 0 |
| 347.040822 | 347.040856 | 3119366 | 16 | 12 | 0 | 9 | 0 | 0 |
| 347.056038 | 347.056112 | 2617864 | 20 | 12 | 0 | 6 | 0 | 0 |
| 347.07725 | 347.077241 | 6486538 | 17 | 16 | 0 | 8 | 0 | 0 |
| 347.092505 | 347.092497 | 3842059 | 21 | 16 | 0 | 5 | 0 | 0 |
| 347.113605 | 347.113627 | 22641678 | 18 | 20 | 0 | 7 | 0 | 0 |
| 347.116939 | 347.116997 | 2968590 | 15 | 24 | 0 | 7 | 1 | 0 |
| 347.128863 | 347.128883 | 4615183 | 22 | 20 | 0 | 4 | 0 | 0 |
| 347.150029 | 347.150012 | 47268880 | 19 | 24 | 0 | 6 | 0 | 0 |
| 347.165261 | 347.165268 | 6177811 | 23 | 24 | 0 | 3 | 0 | 0 |
| 347.201626 | 347.201654 | 3703574 | 24 | 28 | 0 | 2 | 0 | 0 |
| 347.222777 | 347.222783 | 6993432 | 21 | 32 | 0 | 4 | 0 | 0 |
| 347.237888 | 347.238039 | 1392922 | 25 | 32 | 0 | 1 | 0 | 0 |
| 348.145262 | 348.145261 | 1740789 | 18 | 23 | 1 | 6 | 0 | 0 |
| 349.035347 | 349.035376 | 2351823 | 19 | 10 | 0 | 7 | 0 | 0 |
| 349.056483 | 349.056506 | 2410833 | 16 | 14 | 0 | 9 | 0 | 0 |
| 349.0718 | 349.071762 | 4184275 | 20 | 14 | 0 | 6 | 0 | 0 |
| 349.092864 | 349.092891 | 8538325 | 17 | 18 | 0 | 8 | 0 | 0 |
| 349.096229 | 349.096262 | 1306709 | 14 | 22 | 0 | 8 | 1 | 0 |
| 349.108111 | 349.108147 | 5596887 | 21 | 18 | 0 | 5 | 0 | 0 |
| 349.129287 | 349.129277 | 36193496 | 18 | 22 | 0 | 7 | 0 | 0 |
| 349.132628 | 349.132647 | 2066649 | 15 | 26 | 0 | 7 | 1 | 0 |
| 349.144545 | 349.144533 | 8544986 | 22 | 22 | 0 | 4 | 0 | 0 |
| 349.18088 | 349.180918 | 9484510 | 23 | 26 | 0 | 3 | 0 | 0 |
| 349.217247 | 349.217304 | 5236450 | 24 | 30 | 0 | 2 | 0 | 0 |
| 349.238549 | 349.238433 | 1753828 | 21 | 34 | 0 | 4 | 0 | 0 |
| 350.124557 | 350.124526 | 1374143 | 17 | 21 | 1 | 7 | 0 | 0 |
| 350.160968 | 350.160911 | 1290690 | 18 | 25 | 1 | 6 | 0 | 0 |
| 351.014528 | 351.014641 | 1520539 | 18 | 8 | 0 | 8 | 0 | 0 |
| 351.035775 | 351.03577 | 1383197 | 15 | 12 | 0 | 10 | 0 | 0 |
| 351.051011 | 351.051026 | 4092063 | 19 | 12 | 0 | 7 | 0 | 0 |
| 351.072116 | 351.072156 | 3273121 | 16 | 16 | 0 | 9 | 0 | 0 |
| 351.087442 | 351.087412 | 7212963 | 20 | 16 | 0 | 6 | 0 | 0 |
| 351.108535 | 351.108541 | 16148900 | 17 | 20 | 0 | 8 | 0 | 0 |
| 351.123766 | 351.123797 | 10392486 | 21 | 20 | 0 | 5 | 0 | 0 |
| 351.144934 | 351.144927 | 40813992 | 18 | 24 | 0 | 7 | 0 | 0 |
| 351.160226 | 351.160183 | 15745450 | 22 | 24 | 0 | 4 | 0 | 0 |
| 351.196556 | 351.196568 | 14334382 | 23 | 28 | 0 | 3 | 0 | 0 |
| 351.23297 | 351.232954 | 5785010 | 24 | 32 | 0 | 2 | 0 | 0 |
| 352.119085 | 352.119046 | 1360526 | 20 | 19 | 1 | 5 | 0 | 0 |
| 353.030265 | 353.030291 | 3371631 | 18 | 10 | 0 | 8 | 0 | 0 |
| 353.066646 | 353.066676 | 6200691 | 19 | 14 | 0 | 7 | 0 | 0 |
| 353.087763 | 353.087806 | 4238453 | 16 | 18 | 0 | 9 | 0 | 0 |
| 353.103026 | 353.103062 | 9428598 | 20 | 18 | 0 | 6 | 0 | 0 |
| 353.124161 | 353.124191 | 19662456 | 17 | 22 | 0 | 8 | 0 | 0 |
| 353.139447 | 353.139447 | 18563706 | 21 | 22 | 0 | 5 | 0 | 0 |
| 353.175807 | 353.175833 | 24622718 | 22 | 26 | 0 | 4 | 0 | 0 |
| 353.212233 | 353.212218 | 15145602 | 23 | 30 | 0 | 3 | 0 | 0 |
| 353.248553 | 353.248604 | 4167814 | 24 | 34 | 0 | 2 | 0 | 0 |
| 354.061893 | 354.061925 | 1314525 | 18 | 13 | 1 | 7 | 0 | 0 |
| 354.098193 | 354.098311 | 1399648 | 19 | 17 | 1 | 6 | 0 | 0 |
| 354.134763 | 354.134696 | 1768164 | 20 | 21 | 1 | 5 | 0 | 0 |
| 355.009614 | 355.009555 | 1649603 | 17 | 8 | 0 | 9 | 0 | 0 |
| 355.045961 | 355.045941 | 4778567 | 18 | 12 | 0 | 8 | 0 | 0 |
| 355.082304 | 355.082326 | 8299339 | 19 | 16 | 0 | 7 | 0 | 0 |
| 355.085736 | 355.085697 | 1336523 | 16 | 20 | 0 | 7 | 1 | 0 |
| 355.103422 | 355.103456 | 4359501 | 16 | 20 | 0 | 9 | 0 | 0 |
| 355.118729 | 355.118712 | 16740175 | 20 | 20 | 0 | 6 | 0 | 0 |
| 355.122082 | 355.122083 | 1314383 | 17 | 24 | 0 | 6 | 1 | 0 |
| 355.139832 | 355.139841 | 12046161 | 17 | 24 | 0 | 8 | 0 | 0 |
| 355.155094 | 355.155097 | 30494546 | 21 | 24 | 0 | 5 | 0 | 0 |
| 355.176236 | 355.176227 | 9426773 | 18 | 28 | 0 | 7 | 0 | 0 |
| 355.191474 | 355.191483 | 26910550 | 22 | 28 | 0 | 4 | 0 | 0 |
| 355.212616 | 355.212612 | 2075993 | 19 | 32 | 0 | 6 | 0 | 0 |
| 355.227841 | 355.227868 | 11705178 | 23 | 32 | 0 | 3 | 0 | 0 |
| 355.264226 | 355.264254 | 1863774 | 24 | 36 | 0 | 2 | 0 | 0 |
| 356.077605 | 356.077575 | 1344311 | 18 | 15 | 1 | 7 | 0 | 0 |
| 356.114161 | 356.113961 | 1645115 | 19 | 19 | 1 | 6 | 0 | 0 |
| 356.150308 | 356.150346 | 2330047 | 20 | 23 | 1 | 5 | 0 | 0 |
| 356.186766 | 356.186732 | 1440579 | 21 | 27 | 1 | 4 | 0 | 0 |
| 357.025208 | 357.025205 | 2650656 | 17 | 10 | 0 | 9 | 0 | 0 |
| 357.061541 | 357.061591 | 5773860 | 18 | 14 | 0 | 8 | 0 | 0 |
| 357.076858 | 357.076847 | 1623653 | 22 | 14 | 0 | 5 | 0 | 0 |
| 357.097995 | 357.097976 | 12485672 | 19 | 18 | 0 | 7 | 0 | 0 |
| 357.101427 | 357.101347 | 1702440 | 16 | 22 | 0 | 7 | 1 | 0 |
| 357.113225 | 357.113233 | 1689513 | 23 | 18 | 0 | 4 | 0 | 0 |
| 357.119093 | 357.119106 | 2192426 | 16 | 22 | 0 | 9 | 0 | 0 |
| 357.134317 | 357.134362 | 28100652 | 20 | 22 | 0 | 6 | 0 | 0 |
| 357.149462 | 357.149618 | 1549869 | 24 | 22 | 0 | 3 | 0 | 0 |
| 357.155466 | 357.155491 | 4155438 | 17 | 26 | 0 | 8 | 0 | 0 |
| 357.170762 | 357.170747 | 37029936 | 21 | 26 | 0 | 5 | 0 | 0 |
| 357.186042 | 357.186004 | 1615025 | 25 | 26 | 0 | 2 | 0 | 0 |
| 357.191943 | 357.191877 | 2330930 | 18 | 30 | 0 | 7 | 0 | 0 |
| 357.207102 | 357.207133 | 19640372 | 22 | 30 | 0 | 4 | 0 | 0 |
| 357.243517 | 357.243519 | 6035000 | 23 | 34 | 0 | 3 | 0 | 0 |
| 358.093058 | 358.093225 | 1426710 | 18 | 17 | 1 | 7 | 0 | 0 |
| 358.129568 | 358.129611 | 1904411 | 19 | 21 | 1 | 6 | 0 | 0 |
| 358.16598 | 358.165996 | 2070815 | 20 | 25 | 1 | 5 | 0 | 0 |
| 359.040791 | 359.040856 | 3224577 | 17 | 12 | 0 | 9 | 0 | 0 |
| 359.056128 | 359.056112 | 1700355 | 21 | 12 | 0 | 6 | 0 | 0 |
| 359.07724 | 359.077241 | 7088901 | 18 | 16 | 0 | 8 | 0 | 0 |
| 359.080588 | 359.080612 | 2315525 | 15 | 20 | 0 | 8 | 1 | 0 |
| 359.092458 | 359.092497 | 2088455 | 22 | 16 | 0 | 5 | 0 | 0 |
| 359.113614 | 359.113627 | 18849034 | 19 | 20 | 0 | 7 | 0 | 0 |
| 359.11687 | 359.116997 | 1688457 | 16 | 24 | 0 | 7 | 1 | 0 |
| 359.129026 | 359.128883 | 2608267 | 23 | 20 | 0 | 4 | 0 | 0 |
| 359.150006 | 359.150012 | 42068236 | 20 | 24 | 0 | 6 | 0 | 0 |
| 359.16522 | 359.165268 | 3329551 | 24 | 24 | 0 | 3 | 0 | 0 |
| 359.186393 | 359.186398 | 33159442 | 21 | 28 | 0 | 5 | 0 | 0 |
| 359.201668 | 359.201654 | 2140819 | 25 | 28 | 0 | 2 | 0 | 0 |
| 359.222758 | 359.222783 | 11484437 | 22 | 32 | 0 | 4 | 0 | 0 |
| 359.259173 | 359.259169 | 1820954 | 23 | 36 | 0 | 3 | 0 | 0 |
| 360.108745 | 360.108876 | 1306618 | 18 | 19 | 1 | 7 | 0 | 0 |
| 360.1453 | 360.145261 | 2004862 | 19 | 23 | 1 | 6 | 0 | 0 |
| 360.181694 | 360.181647 | 1994114 | 20 | 27 | 1 | 5 | 0 | 0 |
| 361.020033 | 361.02012 | 1484898 | 16 | 10 | 0 | 10 | 0 | 0 |
| 361.035367 | 361.035376 | 1322980 | 20 | 10 | 0 | 7 | 0 | 0 |
| 361.056467 | 361.056506 | 4026342 | 17 | 14 | 0 | 9 | 0 | 0 |
| 361.071835 | 361.071762 | 2681576 | 21 | 14 | 0 | 6 | 0 | 0 |
| 361.092855 | 361.092891 | 9330666 | 18 | 18 | 0 | 8 | 0 | 0 |
| 361.096303 | 361.096262 | 2582891 | 15 | 22 | 0 | 8 | 1 | 0 |
| 361.104175 | 361.104124 | 1436652 | 17 | 18 | 2 | 7 | 0 | 0 |
| 361.108132 | 361.108147 | 3877356 | 22 | 18 | 0 | 5 | 0 | 0 |
| 361.129277 | 361.129277 | 32750062 | 19 | 22 | 0 | 7 | 0 | 0 |
| 361.144478 | 361.144533 | 5349872 | 23 | 22 | 0 | 4 | 0 | 0 |
| 361.165628 | 361.165662 | 46729716 | 20 | 26 | 0 | 6 | 0 | 0 |
| 361.180942 | 361.180918 | 5790197 | 24 | 26 | 0 | 3 | 0 | 0 |
| 361.202026 | 361.202048 | 20742646 | 21 | 30 | 0 | 5 | 0 | 0 |
| 361.217242 | 361.217304 | 3514361 | 25 | 30 | 0 | 2 | 0 | 0 |
| 361.238416 | 361.238433 | 3914491 | 22 | 34 | 0 | 4 | 0 | 0 |
| 362.124463 | 362.124526 | 1753953 | 18 | 21 | 1 | 7 | 0 | 0 |
| 362.160856 | 362.160911 | 1958502 | 19 | 25 | 1 | 6 | 0 | 0 |
| 363.035827 | 363.03577 | 1538827 | 16 | 12 | 0 | 10 | 0 | 0 |
| 363.050983 | 363.051026 | 3720909 | 20 | 12 | 0 | 7 | 0 | 0 |
| 363.072132 | 363.072156 | 4184528 | 17 | 16 | 0 | 9 | 0 | 0 |
| 363.08738 | 363.087412 | 4836049 | 21 | 16 | 0 | 6 | 0 | 0 |
| 363.108489 | 363.108541 | 16724692 | 18 | 20 | 0 | 8 | 0 | 0 |
| 363.111838 | 363.111912 | 2054612 | 15 | 24 | 0 | 8 | 1 | 0 |
| 363.123758 | 363.123797 | 6852822 | 22 | 20 | 0 | 5 | 0 | 0 |
| 363.16016 | 363.160183 | 10068186 | 23 | 24 | 0 | 4 | 0 | 0 |
| 363.19651 | 363.196568 | 9491678 | 24 | 28 | 0 | 3 | 0 | 0 |
| 363.232948 | 363.232954 | 4608738 | 25 | 32 | 0 | 2 | 0 | 0 |
| 364.103762 | 364.10379 | 1355720 | 17 | 19 | 1 | 8 | 0 | 0 |
| 364.14029 | 364.140176 | 1578189 | 18 | 23 | 1 | 7 | 0 | 0 |
| 364.17655 | 364.176561 | 1654865 | 19 | 27 | 1 | 6 | 0 | 0 |
| 365.030281 | 365.030291 | 3098294 | 19 | 10 | 0 | 8 | 0 | 0 |
| 365.051437 | 365.05142 | 1416376 | 16 | 14 | 0 | 10 | 0 | 0 |
| 365.066708 | 365.066676 | 5535674 | 20 | 14 | 0 | 7 | 0 | 0 |
| 365.087762 | 365.087806 | 5664189 | 17 | 18 | 0 | 9 | 0 | 0 |
| 365.103024 | 365.103062 | 6860222 | 21 | 18 | 0 | 6 | 0 | 0 |
| 365.124188 | 365.124191 | 29791168 | 18 | 22 | 0 | 8 | 0 | 0 |
| 365.139468 | 365.139447 | 12287938 | 22 | 22 | 0 | 5 | 0 | 0 |
| 365.175782 | 365.175833 | 15911879 | 23 | 26 | 0 | 4 | 0 | 0 |
| 365.212228 | 365.212218 | 12101579 | 24 | 30 | 0 | 3 | 0 | 0 |
| 365.233228 | 365.233348 | 2424270 | 21 | 34 | 0 | 5 | 0 | 0 |
| 365.248537 | 365.248604 | 4489168 | 25 | 34 | 0 | 2 | 0 | 0 |
| 367.009458 | 367.009555 | 1455010 | 18 | 8 | 0 | 9 | 0 | 0 |
| 367.045922 | 367.045941 | 4550310 | 19 | 12 | 0 | 8 | 0 | 0 |
| 367.066849 | 367.06707 | 1380648 | 16 | 16 | 0 | 10 | 0 | 0 |
| 367.082325 | 367.082326 | 7847595 | 20 | 16 | 0 | 7 | 0 | 0 |
| 367.095476 | 367.095213 | 1324204 | 17 | 21 | 0 | 7 | 0 | 1 |
| 367.103437 | 367.103456 | 9041581 | 17 | 20 | 0 | 9 | 0 | 0 |
| 367.118666 | 367.118712 | 11046063 | 21 | 20 | 0 | 6 | 0 | 0 |
| 367.139821 | 367.139841 | 28703922 | 18 | 24 | 0 | 8 | 0 | 0 |
| 367.155119 | 367.155097 | 19932340 | 22 | 24 | 0 | 5 | 0 | 0 |
| 367.176204 | 367.176227 | 22963382 | 19 | 28 | 0 | 7 | 0 | 0 |
| 367.19142 | 367.191483 | 21595320 | 23 | 28 | 0 | 4 | 0 | 0 |
| 367.227903 | 367.227868 | 11164860 | 24 | 32 | 0 | 3 | 0 | 0 |
| 367.264187 | 367.264254 | 2326720 | 25 | 36 | 0 | 2 | 0 | 0 |
| 368.114123 | 368.113961 | 1503015 | 20 | 19 | 1 | 6 | 0 | 0 |
| 368.150205 | 368.150346 | 1650731 | 21 | 23 | 1 | 5 | 0 | 0 |
| 368.186696 | 368.186732 | 1393455 | 22 | 27 | 1 | 4 | 0 | 0 |
| 369.025213 | 369.025205 | 2568469 | 18 | 10 | 0 | 9 | 0 | 0 |
| 369.061619 | 369.061591 | 5882266 | 19 | 14 | 0 | 8 | 0 | 0 |
| 369.076915 | 369.076847 | 1346715 | 23 | 14 | 0 | 5 | 0 | 0 |
| 369.082706 | 369.08272 | 1626844 | 16 | 18 | 0 | 10 | 0 | 0 |
| 369.097925 | 369.097976 | 8660382 | 20 | 18 | 0 | 7 | 0 | 0 |
| 369.101256 | 369.101347 | 1479582 | 17 | 22 | 0 | 7 | 1 | 0 |
| 369.119105 | 369.119106 | 7950241 | 17 | 22 | 0 | 9 | 0 | 0 |
| 369.134388 | 369.134362 | 18921890 | 21 | 22 | 0 | 6 | 0 | 0 |
| 369.155467 | 369.155491 | 13899173 | 18 | 26 | 0 | 8 | 0 | 0 |
| 369.170702 | 369.170747 | 29078950 | 22 | 26 | 0 | 5 | 0 | 0 |
| 369.191853 | 369.191877 | 7138730 | 19 | 30 | 0 | 7 | 0 | 0 |
| 369.207149 | 369.207133 | 21822892 | 23 | 30 | 0 | 4 | 0 | 0 |
| 369.243455 | 369.243519 | 7645616 | 24 | 34 | 0 | 3 | 0 | 0 |
| 369.279912 | 369.279904 | 1315764 | 25 | 38 | 0 | 2 | 0 | 0 |
| 370.093211 | 370.093225 | 1671959 | 19 | 17 | 1 | 7 | 0 | 0 |
| 370.129549 | 370.129611 | 1965596 | 20 | 21 | 1 | 6 | 0 | 0 |
| 370.16595 | 370.165996 | 1779488 | 21 | 25 | 1 | 5 | 0 | 0 |
| 371.040807 | 371.040856 | 3922828 | 18 | 12 | 0 | 9 | 0 | 0 |
| 371.077223 | 371.077241 | 6775952 | 19 | 16 | 0 | 8 | 0 | 0 |
| 371.092629 | 371.092497 | 1649042 | 23 | 16 | 0 | 5 | 0 | 0 |
| 371.098232 | 371.09837 | 1491475 | 16 | 20 | 0 | 10 | 0 | 0 |
| 371.113577 | 371.113627 | 14447253 | 20 | 20 | 0 | 7 | 0 | 0 |
| 371.116923 | 371.116997 | 2069909 | 17 | 24 | 0 | 7 | 1 | 0 |
| 371.128768 | 371.128883 | 1670807 | 24 | 20 | 0 | 4 | 0 | 0 |
| 371.134753 | 371.134756 | 3872663 | 17 | 24 | 0 | 9 | 0 | 0 |
| 371.150028 | 371.150012 | 30965402 | 21 | 24 | 0 | 6 | 0 | 0 |
| 371.165245 | 371.165268 | 1849499 | 25 | 24 | 0 | 3 | 0 | 0 |
| 371.171076 | 371.171141 | 3953308 | 18 | 28 | 0 | 8 | 0 | 0 |
| 371.186348 | 371.186398 | 31166110 | 22 | 28 | 0 | 5 | 0 | 0 |
| 371.201573 | 371.201654 | 2169760 | 26 | 28 | 0 | 2 | 0 | 0 |
| 371.207571 | 371.207527 | 1476064 | 19 | 32 | 0 | 7 | 0 | 0 |
| 371.222792 | 371.222783 | 17142434 | 23 | 32 | 0 | 4 | 0 | 0 |
| 371.259143 | 371.259169 | 3821223 | 24 | 36 | 0 | 3 | 0 | 0 |
| 372.109011 | 372.108876 | 1434576 | 19 | 19 | 1 | 7 | 0 | 0 |
| 372.145201 | 372.145261 | 1861780 | 20 | 23 | 1 | 6 | 0 | 0 |
| 372.181548 | 372.181647 | 1352857 | 21 | 27 | 1 | 5 | 0 | 0 |
| 373.056465 | 373.056506 | 4419461 | 18 | 14 | 0 | 9 | 0 | 0 |
| 373.071808 | 373.071762 | 2259079 | 22 | 14 | 0 | 6 | 0 | 0 |
| 373.092827 | 373.092891 | 9280394 | 19 | 18 | 0 | 8 | 0 | 0 |
| 373.096166 | 373.096262 | 1531786 | 16 | 22 | 0 | 8 | 1 | 0 |
| 373.108206 | 373.108147 | 2295692 | 23 | 18 | 0 | 5 | 0 | 0 |
| 373.129302 | 373.129277 | 25909134 | 20 | 22 | 0 | 7 | 0 | 0 |
| 373.144565 | 373.144533 | 3434896 | 24 | 22 | 0 | 4 | 0 | 0 |
| 373.165629 | 373.165662 | 36939668 | 21 | 26 | 0 | 6 | 0 | 0 |
| 373.180874 | 373.180918 | 3369109 | 25 | 26 | 0 | 3 | 0 | 0 |
| 373.202068 | 373.202048 | 26576792 | 22 | 30 | 0 | 5 | 0 | 0 |
| 373.21729 | 373.217304 | 2455705 | 26 | 30 | 0 | 2 | 0 | 0 |
| 373.238392 | 373.238433 | 8337820 | 23 | 34 | 0 | 4 | 0 | 0 |
| 374.088103 | 374.08814 | 1425926 | 18 | 17 | 1 | 8 | 0 | 0 |
| 374.124457 | 374.124526 | 1724682 | 19 | 21 | 1 | 7 | 0 | 0 |
| 374.160843 | 374.160911 | 2281359 | 20 | 25 | 1 | 6 | 0 | 0 |
| 374.197286 | 374.197297 | 1367571 | 21 | 29 | 1 | 5 | 0 | 0 |
| 375.035701 | 375.03577 | 1741949 | 17 | 12 | 0 | 10 | 0 | 0 |
| 375.051051 | 375.051026 | 2675583 | 21 | 12 | 0 | 7 | 0 | 0 |
| 375.072117 | 375.072156 | 4741761 | 18 | 16 | 0 | 9 | 0 | 0 |
| 375.087416 | 375.087412 | 3666307 | 22 | 16 | 0 | 6 | 0 | 0 |
| 375.108505 | 375.108541 | 16040070 | 19 | 20 | 0 | 8 | 0 | 0 |
| 375.111948 | 375.111912 | 2096390 | 16 | 24 | 0 | 8 | 1 | 0 |
| 375.123776 | 375.123797 | 4635784 | 23 | 20 | 0 | 5 | 0 | 0 |
| 375.144921 | 375.144927 | 39391372 | 20 | 24 | 0 | 7 | 0 | 0 |
| 375.160127 | 375.160183 | 5531788 | 24 | 24 | 0 | 4 | 0 | 0 |
| 375.181275 | 375.181312 | 38224016 | 21 | 28 | 0 | 6 | 0 | 0 |
| 375.196609 | 375.196568 | 5593233 | 25 | 28 | 0 | 3 | 0 | 0 |
| 375.217706 | 375.217698 | 16025747 | 22 | 32 | 0 | 5 | 0 | 0 |
| 375.232886 | 375.232954 | 2730133 | 26 | 32 | 0 | 2 | 0 | 0 |
| 375.253966 | 375.254083 | 3554968 | 23 | 36 | 0 | 4 | 0 | 0 |
| 376.103861 | 376.10379 | 1316355 | 18 | 19 | 1 | 8 | 0 | 0 |
| 376.140315 | 376.140176 | 1662056 | 19 | 23 | 1 | 7 | 0 | 0 |
| 377.030223 | 377.030291 | 2171512 | 20 | 10 | 0 | 8 | 0 | 0 |
| 377.051516 | 377.05142 | 1912571 | 17 | 14 | 0 | 10 | 0 | 0 |
| 377.066633 | 377.066676 | 3755901 | 21 | 14 | 0 | 7 | 0 | 0 |
| 377.087834 | 377.087806 | 6460288 | 18 | 18 | 0 | 9 | 0 | 0 |
| 377.103062 | 377.103062 | 4900226 | 22 | 18 | 0 | 6 | 0 | 0 |
| 377.124134 | 377.124191 | 27902340 | 19 | 22 | 0 | 8 | 0 | 0 |
| 377.139376 | 377.139447 | 7082886 | 23 | 22 | 0 | 5 | 0 | 0 |
| 377.160593 | 377.160577 | 47837576 | 20 | 26 | 0 | 7 | 0 | 0 |
| 377.175847 | 377.175833 | 9799051 | 24 | 26 | 0 | 4 | 0 | 0 |
| 377.196931 | 377.196962 | 25000334 | 21 | 30 | 0 | 6 | 0 | 0 |
| 377.212178 | 377.212218 | 7391119 | 25 | 30 | 0 | 3 | 0 | 0 |
| 377.233355 | 377.233348 | 6618002 | 22 | 34 | 0 | 5 | 0 | 0 |
| 377.24851 | 377.248604 | 3440276 | 26 | 34 | 0 | 2 | 0 | 0 |
| 378.119343 | 378.11944 | 1316099 | 18 | 21 | 1 | 8 | 0 | 0 |
| 378.155938 | 378.155826 | 1737095 | 19 | 25 | 1 | 7 | 0 | 0 |
| 379.045899 | 379.045941 | 3995257 | 20 | 12 | 0 | 8 | 0 | 0 |
| 379.067062 | 379.06707 | 2502267 | 17 | 16 | 0 | 10 | 0 | 0 |
| 379.08235 | 379.082326 | 5661822 | 21 | 16 | 0 | 7 | 0 | 0 |
| 379.103465 | 379.103456 | 11171456 | 18 | 20 | 0 | 9 | 0 | 0 |
| 379.11866 | 379.118712 | 7573634 | 22 | 20 | 0 | 6 | 0 | 0 |
| 379.139809 | 379.139841 | 38449796 | 19 | 24 | 0 | 8 | 0 | 0 |
| 379.155058 | 379.155097 | 12400263 | 23 | 24 | 0 | 5 | 0 | 0 |
| 379.191452 | 379.191483 | 14258827 | 24 | 28 | 0 | 4 | 0 | 0 |
| 379.212601 | 379.212612 | 11026062 | 21 | 32 | 0 | 6 | 0 | 0 |
| 379.227865 | 379.227868 | 9390736 | 25 | 32 | 0 | 3 | 0 | 0 |
| 379.249113 | 379.248998 | 1778707 | 22 | 36 | 0 | 5 | 0 | 0 |
| 379.264259 | 379.264254 | 3166101 | 26 | 36 | 0 | 2 | 0 | 0 |
| 380.150446 | 380.150346 | 1504518 | 22 | 23 | 1 | 5 | 0 | 0 |
| 381.025127 | 381.025205 | 2533750 | 19 | 10 | 0 | 9 | 0 | 0 |
| 381.061587 | 381.061591 | 4736891 | 20 | 14 | 0 | 8 | 0 | 0 |
| 381.082735 | 381.08272 | 3011198 | 17 | 18 | 0 | 10 | 0 | 0 |
| 381.09796 | 381.097976 | 7223168 | 21 | 18 | 0 | 7 | 0 | 0 |
| 381.101318 | 381.101347 | 1647744 | 18 | 22 | 0 | 7 | 1 | 0 |
| 381.119094 | 381.119106 | 16727938 | 18 | 22 | 0 | 9 | 0 | 0 |
| 381.134311 | 381.134362 | 12819332 | 22 | 22 | 0 | 6 | 0 | 0 |
| 381.137802 | 381.137733 | 1366789 | 19 | 26 | 0 | 6 | 1 | 0 |
| 381.155464 | 381.155491 | 32279432 | 19 | 26 | 0 | 8 | 0 | 0 |
| 381.170748 | 381.170747 | 20253578 | 23 | 26 | 0 | 5 | 0 | 0 |
| 381.191863 | 381.191877 | 20706188 | 20 | 30 | 0 | 7 | 0 | 0 |
| 381.20711 | 381.207133 | 17420174 | 24 | 30 | 0 | 4 | 0 | 0 |
| 381.228204 | 381.228262 | 3215249 | 21 | 34 | 0 | 6 | 0 | 0 |
| 381.243538 | 381.243519 | 7974291 | 25 | 34 | 0 | 3 | 0 | 0 |
| 381.279929 | 381.279904 | 1863191 | 26 | 38 | 0 | 2 | 0 | 0 |
| 382.129618 | 382.129611 | 1789957 | 21 | 21 | 1 | 6 | 0 | 0 |
| 382.166034 | 382.165996 | 1743753 | 22 | 25 | 1 | 5 | 0 | 0 |
| 383.004381 | 383.00447 | 1369590 | 18 | 8 | 0 | 10 | 0 | 0 |
| 383.040767 | 383.040856 | 3777147 | 19 | 12 | 0 | 9 | 0 | 0 |
| 383.077271 | 383.077241 | 6299263 | 20 | 16 | 0 | 8 | 0 | 0 |
| 383.080776 | 383.080612 | 1485440 | 17 | 20 | 0 | 8 | 1 | 0 |
| 383.098377 | 383.09837 | 3566722 | 17 | 20 | 0 | 10 | 0 | 0 |
| 383.113565 | 383.113627 | 10461316 | 21 | 20 | 0 | 7 | 0 | 0 |
| 383.117004 | 383.116997 | 2197380 | 18 | 24 | 0 | 7 | 1 | 0 |
| 383.13477 | 383.134756 | 12541063 | 18 | 24 | 0 | 9 | 0 | 0 |
| 383.150029 | 383.150012 | 21742728 | 22 | 24 | 0 | 6 | 0 | 0 |
| 383.153547 | 383.153383 | 1381769 | 19 | 28 | 0 | 6 | 1 | 0 |
| 383.171142 | 383.171141 | 13849739 | 19 | 28 | 0 | 8 | 0 | 0 |
| 383.186337 | 383.186398 | 26813582 | 23 | 28 | 0 | 5 | 0 | 0 |
| 383.222785 | 383.222783 | 18052242 | 24 | 32 | 0 | 4 | 0 | 0 |
| 383.259098 | 383.259169 | 5151639 | 25 | 36 | 0 | 3 | 0 | 0 |
| 384.10885 | 384.108876 | 1854341 | 20 | 19 | 1 | 7 | 0 | 0 |
| 384.145331 | 384.145261 | 2041482 | 21 | 23 | 1 | 6 | 0 | 0 |
| 385.020133 | 385.02012 | 2022267 | 18 | 10 | 0 | 10 | 0 | 0 |
| 385.05654 | 385.056506 | 4535680 | 19 | 14 | 0 | 9 | 0 | 0 |
| 385.071681 | 385.071762 | 1664642 | 23 | 14 | 0 | 6 | 0 | 0 |
| 385.092878 | 385.092891 | 8193413 | 20 | 18 | 0 | 8 | 0 | 0 |
| 385.09623 | 385.096262 | 2525061 | 17 | 22 | 0 | 8 | 1 | 0 |
| 385.108062 | 385.108147 | 1693703 | 24 | 18 | 0 | 5 | 0 | 0 |
| 385.113953 | 385.11402 | 2461448 | 17 | 22 | 0 | 10 | 0 | 0 |
| 385.129288 | 385.129277 | 17833354 | 21 | 22 | 0 | 7 | 0 | 0 |
| 385.132735 | 385.132647 | 2385290 | 18 | 26 | 0 | 7 | 1 | 0 |
| 385.144549 | 385.144533 | 2182284 | 25 | 22 | 0 | 4 | 0 | 0 |
| 385.150416 | 385.150406 | 5634957 | 18 | 26 | 0 | 9 | 0 | 0 |
| 385.165677 | 385.165662 | 31092110 | 22 | 26 | 0 | 6 | 0 | 0 |
| 385.18088 | 385.180918 | 1822352 | 26 | 26 | 0 | 3 | 0 | 0 |
| 385.186721 | 385.186791 | 4024209 | 19 | 30 | 0 | 8 | 0 | 0 |
| 385.202039 | 385.202048 | 27471250 | 23 | 30 | 0 | 5 | 0 | 0 |
| 385.238477 | 385.238433 | 12544408 | 24 | 34 | 0 | 4 | 0 | 0 |
| 385.274785 | 385.274819 | 2052253 | 25 | 38 | 0 | 3 | 0 | 0 |
| 386.103356 | 386.103396 | 1353992 | 23 | 17 | 1 | 5 | 0 | 0 |
| 386.124453 | 386.124526 | 2064139 | 20 | 21 | 1 | 7 | 0 | 0 |
| 386.16087 | 386.160911 | 1912208 | 21 | 25 | 1 | 6 | 0 | 0 |
| 386.197123 | 386.197297 | 1459093 | 22 | 29 | 1 | 5 | 0 | 0 |
| 387.035806 | 387.03577 | 1908226 | 18 | 12 | 0 | 10 | 0 | 0 |
| 387.05086 | 387.051026 | 1874564 | 22 | 12 | 0 | 7 | 0 | 0 |
| 387.072066 | 387.072156 | 5065351 | 19 | 16 | 0 | 9 | 0 | 0 |
| 387.075678 | 387.075526 | 1319303 | 16 | 20 | 0 | 9 | 1 | 0 |
| 387.087467 | 387.087412 | 2479241 | 23 | 16 | 0 | 6 | 0 | 0 |
| 387.108582 | 387.108541 | 12867212 | 20 | 20 | 0 | 8 | 0 | 0 |
| 387.111941 | 387.111912 | 2740876 | 17 | 24 | 0 | 8 | 1 | 0 |
| 387.123889 | 387.123797 | 2448270 | 24 | 20 | 0 | 5 | 0 | 0 |
| 387.144883 | 387.144927 | 28200592 | 21 | 24 | 0 | 7 | 0 | 0 |
| 387.148298 | 387.148297 | 1797137 | 18 | 28 | 0 | 7 | 1 | 0 |
| 387.160236 | 387.160183 | 3084179 | 25 | 24 | 0 | 4 | 0 | 0 |
| 387.166204 | 387.166056 | 1458195 | 18 | 28 | 0 | 9 | 0 | 0 |
| 387.181331 | 387.181312 | 36308628 | 22 | 28 | 0 | 6 | 0 | 0 |
| 387.196571 | 387.196568 | 3377559 | 26 | 28 | 0 | 3 | 0 | 0 |
| 387.217657 | 387.217698 | 21325466 | 23 | 32 | 0 | 5 | 0 | 0 |
| 387.233005 | 387.232954 | 1835676 | 27 | 32 | 0 | 2 | 0 | 0 |
| 387.254091 | 387.254083 | 6464159 | 24 | 36 | 0 | 4 | 0 | 0 |
| 388.140288 | 388.140176 | 2056979 | 20 | 23 | 1 | 7 | 0 | 0 |
| 388.176543 | 388.176561 | 1778583 | 21 | 27 | 1 | 6 | 0 | 0 |
| 389.051376 | 389.05142 | 2861194 | 18 | 14 | 0 | 10 | 0 | 0 |
| 389.06666 | 389.066676 | 3120524 | 22 | 14 | 0 | 7 | 0 | 0 |
| 389.087859 | 389.087806 | 7157647 | 19 | 18 | 0 | 9 | 0 | 0 |
| 389.091152 | 389.091177 | 2081167 | 16 | 22 | 0 | 9 | 1 | 0 |
| 389.103061 | 389.103062 | 3741073 | 23 | 18 | 0 | 6 | 0 | 0 |
| 389.124153 | 389.124191 | 20348820 | 20 | 22 | 0 | 8 | 0 | 0 |
| 389.127499 | 389.127562 | 2280084 | 17 | 26 | 0 | 8 | 1 | 0 |
| 389.139453 | 389.139447 | 4025750 | 24 | 22 | 0 | 5 | 0 | 0 |
| 389.157813 | 389.158074 | 1327256 | 25 | 26 | 0 | 2 | 1 | 0 |
| 389.160608 | 389.160577 | 40761240 | 21 | 26 | 0 | 7 | 0 | 0 |
| 389.175909 | 389.175833 | 5395611 | 25 | 26 | 0 | 4 | 0 | 0 |
| 389.196947 | 389.196962 | 31528862 | 22 | 30 | 0 | 6 | 0 | 0 |
| 389.212224 | 389.212218 | 4786591 | 26 | 30 | 0 | 3 | 0 | 0 |
| 389.233346 | 389.233348 | 12665762 | 23 | 34 | 0 | 5 | 0 | 0 |
| 389.248593 | 389.248604 | 2270628 | 27 | 34 | 0 | 2 | 0 | 0 |
| 389.269844 | 389.269733 | 1991079 | 24 | 38 | 0 | 4 | 0 | 0 |
| 390.155635 | 390.155826 | 1677851 | 20 | 25 | 1 | 7 | 0 | 0 |
| 390.19242 | 390.192211 | 1592352 | 21 | 29 | 1 | 6 | 0 | 0 |
| 391.045935 | 391.045941 | 3110032 | 21 | 12 | 0 | 8 | 0 | 0 |
| 391.067139 | 391.06707 | 2789011 | 18 | 16 | 0 | 10 | 0 | 0 |
| 391.0824 | 391.082326 | 3878293 | 22 | 16 | 0 | 7 | 0 | 0 |
| 391.103448 | 391.103456 | 10298520 | 19 | 20 | 0 | 9 | 0 | 0 |
| 391.10665 | 391.106827 | 1494936 | 16 | 24 | 0 | 9 | 1 | 0 |
| 391.118685 | 391.118712 | 4585370 | 23 | 20 | 0 | 6 | 0 | 0 |
| 391.139832 | 391.139841 | 35641500 | 20 | 24 | 0 | 8 | 0 | 0 |
| 391.15509 | 391.155097 | 7878303 | 24 | 24 | 0 | 5 | 0 | 0 |
| 391.17624 | 391.176227 | 42502304 | 21 | 28 | 0 | 7 | 0 | 0 |
| 391.191515 | 391.191483 | 9598115 | 25 | 28 | 0 | 4 | 0 | 0 |
| 391.212602 | 391.212612 | 19609766 | 22 | 32 | 0 | 6 | 0 | 0 |
| 391.227882 | 391.227868 | 6452904 | 26 | 32 | 0 | 3 | 0 | 0 |
| 391.249022 | 391.248998 | 4324011 | 23 | 36 | 0 | 5 | 0 | 0 |
| 391.264284 | 391.264254 | 2611117 | 27 | 36 | 0 | 2 | 0 | 0 |
| 392.113846 | 392.113961 | 1369629 | 22 | 19 | 1 | 6 | 0 | 0 |
| 392.135164 | 392.13509 | 1791135 | 19 | 23 | 1 | 8 | 0 | 0 |
| 392.171475 | 392.171476 | 1426212 | 20 | 27 | 1 | 7 | 0 | 0 |
| 393.025192 | 393.025205 | 2451605 | 20 | 10 | 0 | 9 | 0 | 0 |
| 393.046289 | 393.046335 | 1398167 | 17 | 14 | 0 | 11 | 0 | 0 |
| 393.061584 | 393.061591 | 4177817 | 21 | 14 | 0 | 8 | 0 | 0 |
| 393.082751 | 393.08272 | 3465884 | 18 | 18 | 0 | 10 | 0 | 0 |
| 393.097883 | 393.097976 | 4850078 | 22 | 18 | 0 | 7 | 0 | 0 |
| 393.119133 | 393.119106 | 19929504 | 19 | 22 | 0 | 9 | 0 | 0 |
| 393.134391 | 393.134362 | 8444323 | 23 | 22 | 0 | 6 | 0 | 0 |
| 393.155482 | 393.155491 | 42994084 | 20 | 26 | 0 | 8 | 0 | 0 |
| 393.170706 | 393.170747 | 12418472 | 24 | 26 | 0 | 5 | 0 | 0 |
| 393.191842 | 393.191877 | 25651626 | 21 | 30 | 0 | 7 | 0 | 0 |
| 393.207119 | 393.207133 | 13556140 | 25 | 30 | 0 | 4 | 0 | 0 |
| 393.228363 | 393.228262 | 7834032 | 22 | 34 | 0 | 6 | 0 | 0 |
| 393.243579 | 393.243519 | 6947250 | 26 | 34 | 0 | 3 | 0 | 0 |
| 393.279921 | 393.279904 | 1984694 | 27 | 38 | 0 | 2 | 0 | 0 |
| 395.040788 | 395.040856 | 3727262 | 20 | 12 | 0 | 9 | 0 | 0 |
| 395.077208 | 395.077241 | 4555683 | 21 | 16 | 0 | 8 | 0 | 0 |
| 395.090138 | 395.090128 | 1341349 | 18 | 21 | 0 | 8 | 0 | 1 |
| 395.098418 | 395.09837 | 6298790 | 18 | 20 | 0 | 10 | 0 | 0 |
| 395.113683 | 395.113627 | 8003752 | 22 | 20 | 0 | 7 | 0 | 0 |
| 395.134767 | 395.134756 | 24083114 | 19 | 24 | 0 | 9 | 0 | 0 |
| 395.149954 | 395.150012 | 12464813 | 23 | 24 | 0 | 6 | 0 | 0 |
| 395.171097 | 395.171141 | 28119728 | 20 | 28 | 0 | 8 | 0 | 0 |
| 395.186444 | 395.186398 | 19333810 | 24 | 28 | 0 | 5 | 0 | 0 |
| 395.207555 | 395.207527 | 11707060 | 21 | 32 | 0 | 7 | 0 | 0 |
| 395.222802 | 395.222783 | 14684854 | 25 | 32 | 0 | 4 | 0 | 0 |
| 395.243791 | 395.243912 | 2126521 | 22 | 36 | 0 | 6 | 0 | 0 |
| 395.25907 | 395.259169 | 6071995 | 26 | 36 | 0 | 3 | 0 | 0 |
| 395.295645 | 395.295554 | 1346240 | 27 | 40 | 0 | 2 | 0 | 0 |
| 396.108972 | 396.108876 | 1384491 | 21 | 19 | 1 | 7 | 0 | 0 |
| 397.020077 | 397.02012 | 1659683 | 19 | 10 | 0 | 10 | 0 | 0 |
| 397.056537 | 397.056506 | 4260264 | 20 | 14 | 0 | 9 | 0 | 0 |
| 397.077458 | 397.077635 | 1781163 | 17 | 18 | 0 | 11 | 0 | 0 |
| 397.092897 | 397.092891 | 6414765 | 21 | 18 | 0 | 8 | 0 | 0 |
| 397.096095 | 397.096262 | 2411181 | 18 | 22 | 0 | 8 | 1 | 0 |
| 397.114101 | 397.11402 | 6561200 | 18 | 22 | 0 | 10 | 0 | 0 |
| 397.129286 | 397.129277 | 12623794 | 22 | 22 | 0 | 7 | 0 | 0 |
| 397.132607 | 397.132647 | 2375346 | 19 | 26 | 0 | 7 | 1 | 0 |
| 397.150385 | 397.150406 | 15273908 | 19 | 26 | 0 | 9 | 0 | 0 |
| 397.165607 | 397.165662 | 21142454 | 23 | 26 | 0 | 6 | 0 | 0 |
| 397.186806 | 397.186791 | 11729849 | 20 | 30 | 0 | 8 | 0 | 0 |
| 397.202067 | 397.202048 | 23538620 | 24 | 30 | 0 | 5 | 0 | 0 |
| 397.223191 | 397.223177 | 3077054 | 21 | 34 | 0 | 7 | 0 | 0 |
| 397.238465 | 397.238433 | 11917248 | 25 | 34 | 0 | 4 | 0 | 0 |
| 397.274773 | 397.274819 | 3792069 | 26 | 38 | 0 | 3 | 0 | 0 |
| 398.124492 | 398.124526 | 1773109 | 21 | 21 | 1 | 7 | 0 | 0 |
| 398.160871 | 398.160911 | 1758266 | 22 | 25 | 1 | 6 | 0 | 0 |
| 399.035842 | 399.03577 | 2450093 | 19 | 12 | 0 | 10 | 0 | 0 |
| 399.051077 | 399.051026 | 1357999 | 23 | 12 | 0 | 7 | 0 | 0 |
| 399.072176 | 399.072156 | 4668082 | 20 | 16 | 0 | 9 | 0 | 0 |
| 399.07549 | 399.075526 | 1390258 | 17 | 20 | 0 | 9 | 1 | 0 |
| 399.087357 | 399.087412 | 1939380 | 24 | 16 | 0 | 6 | 0 | 0 |
| 399.108526 | 399.108541 | 8853687 | 21 | 20 | 0 | 8 | 0 | 0 |
| 399.111977 | 399.111912 | 3056311 | 18 | 24 | 0 | 8 | 1 | 0 |
| 399.123713 | 399.123797 | 1855673 | 25 | 20 | 0 | 5 | 0 | 0 |
| 399.12967 | 399.129671 | 3686330 | 18 | 24 | 0 | 10 | 0 | 0 |
| 399.144916 | 399.144927 | 20022460 | 22 | 24 | 0 | 7 | 0 | 0 |
| 399.148418 | 399.148297 | 2851004 | 19 | 28 | 0 | 7 | 1 | 0 |
| 399.160345 | 399.160183 | 1867198 | 26 | 24 | 0 | 4 | 0 | 0 |
| 399.166076 | 399.166056 | 6141631 | 19 | 28 | 0 | 9 | 0 | 0 |
| 399.18132 | 399.181312 | 28527808 | 23 | 28 | 0 | 6 | 0 | 0 |
| 399.196749 | 399.196568 | 1439682 | 27 | 28 | 0 | 3 | 0 | 0 |
| 399.202415 | 399.202442 | 3245251 | 20 | 32 | 0 | 8 | 0 | 0 |
| 399.217651 | 399.217698 | 21843142 | 24 | 32 | 0 | 5 | 0 | 0 |
| 399.25413 | 399.254083 | 8900810 | 25 | 36 | 0 | 4 | 0 | 0 |
| 400.140188 | 400.140176 | 1708095 | 21 | 23 | 1 | 7 | 0 | 0 |
| 400.176494 | 400.176561 | 1621316 | 22 | 27 | 1 | 6 | 0 | 0 |
| 401.051498 | 401.05142 | 3280311 | 19 | 14 | 0 | 10 | 0 | 0 |
| 401.066807 | 401.066676 | 2219321 | 23 | 14 | 0 | 7 | 0 | 0 |
| 401.087823 | 401.087806 | 5745852 | 20 | 18 | 0 | 9 | 0 | 0 |
| 401.091076 | 401.091177 | 2604220 | 17 | 22 | 0 | 9 | 1 | 0 |
| 401.103035 | 401.103062 | 3128766 | 24 | 18 | 0 | 6 | 0 | 0 |
| 401.124161 | 401.124191 | 15284672 | 21 | 22 | 0 | 8 | 0 | 0 |
| 401.127548 | 401.127562 | 3951041 | 18 | 26 | 0 | 8 | 1 | 0 |
| 401.139445 | 401.139447 | 2796994 | 25 | 22 | 0 | 5 | 0 | 0 |
| 401.160575 | 401.160577 | 29228486 | 22 | 26 | 0 | 7 | 0 | 0 |
| 401.164051 | 401.163948 | 2051910 | 19 | 30 | 0 | 7 | 1 | 0 |
| 401.175728 | 401.175833 | 3090631 | 26 | 26 | 0 | 4 | 0 | 0 |
| 401.182 | 401.181706 | 1723592 | 19 | 30 | 0 | 9 | 0 | 0 |
| 401.196926 | 401.196962 | 29896138 | 23 | 30 | 0 | 6 | 0 | 0 |
| 401.21221 | 401.212218 | 2660812 | 27 | 30 | 0 | 3 | 0 | 0 |
| 401.23334 | 401.233348 | 17073614 | 24 | 34 | 0 | 5 | 0 | 0 |
| 401.269795 | 401.269733 | 3569364 | 25 | 38 | 0 | 4 | 0 | 0 |
| 402.155675 | 402.155826 | 2051912 | 21 | 25 | 1 | 7 | 0 | 0 |
| 402.192337 | 402.192211 | 1459405 | 22 | 29 | 1 | 6 | 0 | 0 |
| 403.046042 | 403.045941 | 2282685 | 22 | 12 | 0 | 8 | 0 | 0 |
| 403.067114 | 403.06707 | 2681024 | 19 | 16 | 0 | 10 | 0 | 0 |
| 403.08239 | 403.082326 | 3018946 | 23 | 16 | 0 | 7 | 0 | 0 |
| 403.103478 | 403.103456 | 8821957 | 20 | 20 | 0 | 9 | 0 | 0 |
| 403.106791 | 403.106827 | 2583877 | 17 | 24 | 0 | 9 | 1 | 0 |
| 403.11877 | 403.118712 | 3501255 | 24 | 20 | 0 | 6 | 0 | 0 |
| 403.13985 | 403.139841 | 25978570 | 21 | 24 | 0 | 8 | 0 | 0 |
| 403.155115 | 403.155097 | 4972748 | 25 | 24 | 0 | 5 | 0 | 0 |
| 403.176212 | 403.176227 | 38780624 | 22 | 28 | 0 | 7 | 0 | 0 |
| 403.191386 | 403.191483 | 4993744 | 26 | 28 | 0 | 4 | 0 | 0 |
| 403.212611 | 403.212612 | 26361554 | 23 | 32 | 0 | 6 | 0 | 0 |
| 403.227839 | 403.227868 | 4052181 | 27 | 32 | 0 | 3 | 0 | 0 |
| 403.248957 | 403.248998 | 9649880 | 24 | 36 | 0 | 5 | 0 | 0 |
| 403.264112 | 403.264254 | 2107098 | 28 | 36 | 0 | 2 | 0 | 0 |
| 404.135088 | 404.13509 | 1746124 | 20 | 23 | 1 | 8 | 0 | 0 |
| 404.171241 | 404.171476 | 1614161 | 21 | 27 | 1 | 7 | 0 | 0 |
| 405.025016 | 405.025205 | 1643841 | 21 | 10 | 0 | 9 | 0 | 0 |
| 405.046281 | 405.046335 | 1570243 | 18 | 14 | 0 | 11 | 0 | 0 |
| 405.061646 | 405.061591 | 3905989 | 22 | 14 | 0 | 8 | 0 | 0 |
| 405.082608 | 405.08272 | 3949768 | 19 | 18 | 0 | 10 | 0 | 0 |
| 405.098087 | 405.097976 | 4563914 | 23 | 18 | 0 | 7 | 0 | 0 |
| 405.119112 | 405.119106 | 16226253 | 20 | 22 | 0 | 9 | 0 | 0 |
| 405.12261 | 405.122477 | 1901645 | 17 | 26 | 0 | 9 | 1 | 0 |
| 405.134404 | 405.134362 | 5222351 | 24 | 22 | 0 | 6 | 0 | 0 |
| 405.155471 | 405.155491 | 37654480 | 21 | 26 | 0 | 8 | 0 | 0 |
| 405.170697 | 405.170747 | 7866836 | 25 | 26 | 0 | 5 | 0 | 0 |
| 405.19187 | 405.191877 | 36716504 | 22 | 30 | 0 | 7 | 0 | 0 |
| 405.207115 | 405.207133 | 7744985 | 26 | 30 | 0 | 4 | 0 | 0 |
| 405.228256 | 405.228262 | 15050715 | 23 | 34 | 0 | 6 | 0 | 0 |
| 405.243493 | 405.243519 | 5512669 | 27 | 34 | 0 | 3 | 0 | 0 |
| 405.280006 | 405.279904 | 2043618 | 28 | 38 | 0 | 2 | 0 | 0 |
| 406.15088 | 406.15074 | 1461076 | 20 | 25 | 1 | 8 | 0 | 0 |
| 406.187078 | 406.187126 | 1528153 | 21 | 29 | 1 | 7 | 0 | 0 |
| 407.040825 | 407.040856 | 2851016 | 21 | 12 | 0 | 9 | 0 | 0 |
| 407.077229 | 407.077241 | 5261005 | 22 | 16 | 0 | 8 | 0 | 0 |
| 407.080459 | 407.080612 | 1417933 | 19 | 20 | 0 | 8 | 1 | 0 |
| 407.098338 | 407.09837 | 5641680 | 19 | 20 | 0 | 10 | 0 | 0 |
| 407.113568 | 407.113627 | 5931218 | 23 | 20 | 0 | 7 | 0 | 0 |
| 407.117002 | 407.116997 | 1552338 | 20 | 24 | 0 | 7 | 1 | 0 |
| 407.13472 | 407.134756 | 27451604 | 20 | 24 | 0 | 9 | 0 | 0 |
| 407.149944 | 407.150012 | 8743126 | 24 | 24 | 0 | 6 | 0 | 0 |
| 407.171134 | 407.171141 | 38764760 | 21 | 28 | 0 | 8 | 0 | 0 |
| 407.186398 | 407.186398 | 12632283 | 25 | 28 | 0 | 5 | 0 | 0 |
| 407.207508 | 407.207527 | 21147870 | 22 | 32 | 0 | 7 | 0 | 0 |
| 407.222762 | 407.222783 | 11759840 | 26 | 32 | 0 | 4 | 0 | 0 |
| 407.243903 | 407.243912 | 5040355 | 23 | 36 | 0 | 6 | 0 | 0 |
| 407.259167 | 407.259169 | 5852389 | 27 | 36 | 0 | 3 | 0 | 0 |
| 408.145192 | 408.145261 | 1401688 | 23 | 23 | 1 | 6 | 0 | 0 |
| 409.020126 | 409.02012 | 2176714 | 20 | 10 | 0 | 10 | 0 | 0 |
| 409.056543 | 409.056506 | 4498895 | 21 | 14 | 0 | 9 | 0 | 0 |
| 409.077649 | 409.077635 | 1922513 | 18 | 18 | 0 | 11 | 0 | 0 |
| 409.092922 | 409.092891 | 6346707 | 22 | 18 | 0 | 8 | 0 | 0 |
| 409.09628 | 409.096262 | 1885204 | 19 | 22 | 0 | 8 | 1 | 0 |
| 409.105862 | 409.105778 | 1686869 | 19 | 23 | 0 | 8 | 0 | 1 |
| 409.114044 | 409.11402 | 10267606 | 19 | 22 | 0 | 10 | 0 | 0 |
| 409.129224 | 409.129277 | 8398296 | 23 | 22 | 0 | 7 | 0 | 0 |
| 409.132521 | 409.132647 | 2174296 | 20 | 26 | 0 | 7 | 1 | 0 |
| 409.150388 | 409.150406 | 25848282 | 20 | 26 | 0 | 9 | 0 | 0 |
| 409.165584 | 409.165662 | 13692380 | 24 | 26 | 0 | 6 | 0 | 0 |
| 409.186781 | 409.186791 | 21686752 | 21 | 30 | 0 | 8 | 0 | 0 |
| 409.202053 | 409.202048 | 16759265 | 25 | 30 | 0 | 5 | 0 | 0 |
| 409.223204 | 409.223177 | 7802340 | 22 | 34 | 0 | 7 | 0 | 0 |
| 409.238354 | 409.238433 | 12370406 | 26 | 34 | 0 | 4 | 0 | 0 |
| 409.274748 | 409.274819 | 4796139 | 27 | 38 | 0 | 3 | 0 | 0 |
| 410.124653 | 410.124526 | 1357273 | 22 | 21 | 1 | 7 | 0 | 0 |
| 410.16108 | 410.160911 | 1416926 | 23 | 25 | 1 | 6 | 0 | 0 |
| 410.197429 | 410.197297 | 1419362 | 24 | 29 | 1 | 5 | 0 | 0 |
| 411.035793 | 411.03577 | 2814927 | 20 | 12 | 0 | 10 | 0 | 0 |
| 411.07216 | 411.072156 | 5073107 | 21 | 16 | 0 | 9 | 0 | 0 |
| 411.07549 | 411.075526 | 1851604 | 18 | 20 | 0 | 9 | 1 | 0 |
| 411.093288 | 411.093285 | 2358870 | 18 | 20 | 0 | 11 | 0 | 0 |
| 411.108464 | 411.108541 | 7305432 | 22 | 20 | 0 | 8 | 0 | 0 |
| 411.111872 | 411.111912 | 3695320 | 19 | 24 | 0 | 8 | 1 | 0 |
| 411.129669 | 411.129671 | 10467035 | 19 | 24 | 0 | 10 | 0 | 0 |
| 411.144957 | 411.144927 | 13749981 | 23 | 24 | 0 | 7 | 0 | 0 |
| 411.148386 | 411.148297 | 2499805 | 20 | 28 | 0 | 7 | 1 | 0 |
| 411.166044 | 411.166056 | 14158559 | 20 | 28 | 0 | 9 | 0 | 0 |
| 411.181314 | 411.181312 | 21527266 | 24 | 28 | 0 | 6 | 0 | 0 |
| 411.202428 | 411.202442 | 7619300 | 21 | 32 | 0 | 8 | 0 | 0 |
| 411.217667 | 411.217698 | 19333862 | 25 | 32 | 0 | 5 | 0 | 0 |
| 411.238957 | 411.238827 | 1874153 | 22 | 36 | 0 | 7 | 0 | 0 |
| 411.254093 | 411.254083 | 10112747 | 26 | 36 | 0 | 4 | 0 | 0 |
| 411.290505 | 411.290469 | 2738415 | 27 | 40 | 0 | 3 | 0 | 0 |
| 412.140161 | 412.140176 | 1510749 | 22 | 23 | 1 | 7 | 0 | 0 |
| 412.176602 | 412.176561 | 1509602 | 23 | 27 | 1 | 6 | 0 | 0 |
| 413.051485 | 413.05142 | 3223506 | 20 | 14 | 0 | 10 | 0 | 0 |
| 413.066745 | 413.066676 | 1898708 | 24 | 14 | 0 | 7 | 0 | 0 |
| 413.087722 | 413.087806 | 5918167 | 21 | 18 | 0 | 9 | 0 | 0 |
| 413.091251 | 413.091177 | 2086103 | 18 | 22 | 0 | 9 | 1 | 0 |
| 413.103046 | 413.103062 | 1836505 | 25 | 18 | 0 | 6 | 0 | 0 |
| 413.108849 | 413.108935 | 1984985 | 18 | 22 | 0 | 11 | 0 | 0 |
| 413.124206 | 413.124191 | 10806235 | 22 | 22 | 0 | 8 | 0 | 0 |
| 413.127564 | 413.127562 | 4465116 | 19 | 26 | 0 | 8 | 1 | 0 |
| 413.139621 | 413.139447 | 2048093 | 26 | 22 | 0 | 5 | 0 | 0 |
| 413.145287 | 413.145321 | 4356318 | 19 | 26 | 0 | 10 | 0 | 0 |
| 413.160552 | 413.160577 | 22023136 | 23 | 26 | 0 | 7 | 0 | 0 |
| 413.163681 | 413.163948 | 2190560 | 20 | 30 | 0 | 7 | 1 | 0 |
| 413.17577 | 413.175833 | 2067426 | 27 | 26 | 0 | 4 | 0 | 0 |
| 413.181731 | 413.181706 | 5242339 | 20 | 30 | 0 | 9 | 0 | 0 |
| 413.196936 | 413.196962 | 26670052 | 24 | 30 | 0 | 6 | 0 | 0 |
| 413.218187 | 413.218092 | 2206055 | 21 | 34 | 0 | 8 | 0 | 0 |
| 413.233346 | 413.233348 | 17605610 | 25 | 34 | 0 | 5 | 0 | 0 |
| 413.269696 | 413.269733 | 6168558 | 26 | 38 | 0 | 4 | 0 | 0 |
| 413.306193 | 413.306119 | 1467123 | 27 | 42 | 0 | 3 | 0 | 0 |
| 414.119651 | 414.11944 | 1402971 | 21 | 21 | 1 | 8 | 0 | 0 |
| 414.155773 | 414.155826 | 1588191 | 22 | 25 | 1 | 7 | 0 | 0 |
| 414.192073 | 414.192211 | 1515236 | 23 | 29 | 1 | 6 | 0 | 0 |
| 415.030612 | 415.030685 | 1682639 | 19 | 12 | 0 | 11 | 0 | 0 |
| 415.04605 | 415.045941 | 1762001 | 23 | 12 | 0 | 8 | 0 | 0 |
| 415.067146 | 415.06707 | 3950036 | 20 | 16 | 0 | 10 | 0 | 0 |
| 415.082403 | 415.082326 | 2173973 | 24 | 16 | 0 | 7 | 0 | 0 |
| 415.103425 | 415.103456 | 7306456 | 21 | 20 | 0 | 9 | 0 | 0 |
| 415.10688 | 415.106827 | 3433177 | 18 | 24 | 0 | 9 | 1 | 0 |
| 415.118601 | 415.118712 | 2790874 | 25 | 20 | 0 | 6 | 0 | 0 |
| 415.13986 | 415.139841 | 18330844 | 22 | 24 | 0 | 8 | 0 | 0 |
| 415.14323 | 415.143212 | 4515037 | 19 | 28 | 0 | 8 | 1 | 0 |
| 415.155098 | 415.155097 | 2884575 | 26 | 24 | 0 | 5 | 0 | 0 |
| 415.161036 | 415.160971 | 1982431 | 19 | 28 | 0 | 10 | 0 | 0 |
| 415.176234 | 415.176227 | 31315170 | 23 | 28 | 0 | 7 | 0 | 0 |
| 415.191542 | 415.191483 | 2738019 | 27 | 28 | 0 | 4 | 0 | 0 |
| 415.197485 | 415.197356 | 1578404 | 20 | 32 | 0 | 9 | 0 | 0 |
| 415.212602 | 415.212612 | 28335334 | 24 | 32 | 0 | 6 | 0 | 0 |
| 415.227919 | 415.227868 | 2009320 | 28 | 32 | 0 | 3 | 0 | 0 |
| 415.249024 | 415.248998 | 12191979 | 25 | 36 | 0 | 5 | 0 | 0 |
| 415.285374 | 415.285383 | 2717679 | 26 | 40 | 0 | 4 | 0 | 0 |
| 416.134851 | 416.13509 | 1413851 | 21 | 23 | 1 | 8 | 0 | 0 |
| 416.17173 | 416.171476 | 1485664 | 22 | 27 | 1 | 7 | 0 | 0 |
| 417.025362 | 417.025205 | 1401292 | 22 | 10 | 0 | 9 | 0 | 0 |
| 417.046131 | 417.046335 | 2191054 | 19 | 14 | 0 | 11 | 0 | 0 |
| 417.061619 | 417.061591 | 3230672 | 23 | 14 | 0 | 8 | 0 | 0 |
| 417.082661 | 417.08272 | 4209107 | 20 | 18 | 0 | 10 | 0 | 0 |
| 417.086074 | 417.086091 | 1543123 | 17 | 22 | 0 | 10 | 1 | 0 |
| 417.098009 | 417.097976 | 3402453 | 24 | 18 | 0 | 7 | 0 | 0 |
| 417.119166 | 417.119106 | 12046807 | 21 | 22 | 0 | 9 | 0 | 0 |
| 417.122479 | 417.122477 | 3527128 | 18 | 26 | 0 | 9 | 1 | 0 |
| 417.134279 | 417.134362 | 3691737 | 25 | 22 | 0 | 6 | 0 | 0 |
| 417.15547 | 417.155491 | 28839388 | 22 | 26 | 0 | 8 | 0 | 0 |
| 417.17079 | 417.170747 | 4184798 | 26 | 26 | 0 | 5 | 0 | 0 |
| 417.191896 | 417.191877 | 34956768 | 23 | 30 | 0 | 7 | 0 | 0 |
| 417.207242 | 417.207133 | 5010915 | 27 | 30 | 0 | 4 | 0 | 0 |
| 417.228266 | 417.228262 | 22609382 | 24 | 34 | 0 | 6 | 0 | 0 |
| 417.243661 | 417.243519 | 2728679 | 28 | 34 | 0 | 3 | 0 | 0 |
| 417.264633 | 417.264648 | 6291178 | 25 | 38 | 0 | 5 | 0 | 0 |
| 418.150592 | 418.15074 | 1732441 | 21 | 25 | 1 | 8 | 0 | 0 |
| 418.187337 | 418.187126 | 1755486 | 22 | 29 | 1 | 7 | 0 | 0 |
| 419.040775 | 419.040856 | 2611657 | 22 | 12 | 0 | 9 | 0 | 0 |
| 419.062118 | 419.061985 | 2360268 | 19 | 16 | 0 | 11 | 0 | 0 |
| 419.077299 | 419.077241 | 4480206 | 23 | 16 | 0 | 8 | 0 | 0 |
| 419.098358 | 419.09837 | 5619920 | 20 | 20 | 0 | 10 | 0 | 0 |
| 419.10178 | 419.101741 | 1735889 | 17 | 24 | 0 | 10 | 1 | 0 |
| 419.113608 | 419.113627 | 4294866 | 24 | 20 | 0 | 7 | 0 | 0 |
| 419.134763 | 419.134756 | 20389588 | 21 | 24 | 0 | 9 | 0 | 0 |
| 419.138243 | 419.138127 | 2131925 | 18 | 28 | 0 | 9 | 1 | 0 |
| 419.149958 | 419.150012 | 6464215 | 25 | 24 | 0 | 6 | 0 | 0 |
| 419.171151 | 419.171141 | 39704280 | 22 | 28 | 0 | 8 | 0 | 0 |
| 419.186382 | 419.186398 | 8215259 | 26 | 28 | 0 | 5 | 0 | 0 |
| 419.207547 | 419.207527 | 30344926 | 23 | 32 | 0 | 7 | 0 | 0 |
| 419.222808 | 419.222783 | 7364320 | 27 | 32 | 0 | 4 | 0 | 0 |
| 419.243883 | 419.243912 | 12924642 | 24 | 36 | 0 | 6 | 0 | 0 |
| 419.259217 | 419.259169 | 3339748 | 28 | 36 | 0 | 3 | 0 | 0 |
| 419.280598 | 419.280298 | 2476519 | 25 | 40 | 0 | 5 | 0 | 0 |
| 420.15652 | 420.156494 | 1364564 | 23 | 23 | 3 | 5 | 0 | 0 |
| 421.020402 | 421.02012 | 1512383 | 21 | 10 | 0 | 10 | 0 | 0 |
| 421.056584 | 421.056506 | 3851204 | 22 | 14 | 0 | 9 | 0 | 0 |
| 421.077891 | 421.077635 | 1938886 | 19 | 18 | 0 | 11 | 0 | 0 |
| 421.092891 | 421.092891 | 4416456 | 23 | 18 | 0 | 8 | 0 | 0 |
| 421.096121 | 421.096262 | 1593801 | 20 | 22 | 0 | 8 | 1 | 0 |
| 421.114052 | 421.11402 | 10275787 | 20 | 22 | 0 | 10 | 0 | 0 |
| 421.129271 | 421.129277 | 5524429 | 24 | 22 | 0 | 7 | 0 | 0 |
| 421.1504 | 421.150406 | 29829072 | 21 | 26 | 0 | 9 | 0 | 0 |
| 421.165629 | 421.165662 | 8862673 | 25 | 26 | 0 | 6 | 0 | 0 |
| 421.186813 | 421.186791 | 33548244 | 22 | 30 | 0 | 8 | 0 | 0 |
| 421.202058 | 421.202048 | 11907030 | 26 | 30 | 0 | 5 | 0 | 0 |
| 421.223152 | 421.223177 | 15516632 | 23 | 34 | 0 | 7 | 0 | 0 |
| 421.238426 | 421.238433 | 9015770 | 27 | 34 | 0 | 4 | 0 | 0 |
| 421.259549 | 421.259562 | 4236253 | 24 | 38 | 0 | 6 | 0 | 0 |
| 421.274826 | 421.274819 | 4077279 | 28 | 38 | 0 | 3 | 0 | 0 |
| 423.035778 | 423.03577 | 2554807 | 21 | 12 | 0 | 10 | 0 | 0 |
| 423.072179 | 423.072156 | 4138428 | 22 | 16 | 0 | 9 | 0 | 0 |
| 423.093216 | 423.093285 | 2890174 | 19 | 20 | 0 | 11 | 0 | 0 |
| 423.108587 | 423.108541 | 5693120 | 23 | 20 | 0 | 8 | 0 | 0 |
| 423.112187 | 423.111912 | 2104001 | 20 | 24 | 0 | 8 | 1 | 0 |
| 423.121649 | 423.121428 | 1453250 | 20 | 25 | 0 | 8 | 0 | 1 |
| 423.12969 | 423.129671 | 15021251 | 20 | 24 | 0 | 10 | 0 | 0 |
| 423.144964 | 423.144927 | 8988869 | 24 | 24 | 0 | 7 | 0 | 0 |
| 423.16605 | 423.166056 | 25725128 | 21 | 28 | 0 | 9 | 0 | 0 |
| 423.181374 | 423.181312 | 13965513 | 25 | 28 | 0 | 6 | 0 | 0 |
| 423.202425 | 423.202442 | 16185548 | 22 | 32 | 0 | 8 | 0 | 0 |
| 423.217696 | 423.217698 | 14679246 | 26 | 32 | 0 | 5 | 0 | 0 |
| 423.238846 | 423.238827 | 5273808 | 23 | 36 | 0 | 7 | 0 | 0 |
| 423.254109 | 423.254083 | 9600210 | 27 | 36 | 0 | 4 | 0 | 0 |
| 423.290532 | 423.290469 | 3415255 | 28 | 40 | 0 | 3 | 0 | 0 |
| 425.051566 | 425.05142 | 3573677 | 21 | 14 | 0 | 10 | 0 | 0 |
| 425.087865 | 425.087806 | 5354417 | 22 | 18 | 0 | 9 | 0 | 0 |
| 425.091342 | 425.091177 | 2188721 | 19 | 22 | 0 | 9 | 1 | 0 |
| 425.103153 | 425.103062 | 1353523 | 26 | 18 | 0 | 6 | 0 | 0 |
| 425.10892 | 425.108935 | 3721140 | 19 | 22 | 0 | 11 | 0 | 0 |
| 425.124191 | 425.124191 | 8020406 | 23 | 22 | 0 | 8 | 0 | 0 |
| 425.127479 | 425.127562 | 3442358 | 20 | 26 | 0 | 8 | 1 | 0 |
| 425.145298 | 425.145321 | 11570616 | 20 | 26 | 0 | 10 | 0 | 0 |
| 425.16054 | 425.160577 | 14239162 | 24 | 26 | 0 | 7 | 0 | 0 |
| 425.164084 | 425.163948 | 1982394 | 21 | 30 | 0 | 7 | 1 | 0 |
| 425.181682 | 425.181706 | 11296188 | 21 | 30 | 0 | 9 | 0 | 0 |
| 425.196982 | 425.196962 | 20801982 | 25 | 30 | 0 | 6 | 0 | 0 |
| 425.21815 | 425.218092 | 5386177 | 22 | 34 | 0 | 8 | 0 | 0 |
| 425.23336 | 425.233348 | 16677314 | 26 | 34 | 0 | 5 | 0 | 0 |
| 425.269706 | 425.269733 | 8310215 | 27 | 38 | 0 | 4 | 0 | 0 |
| 425.306304 | 425.306119 | 1833035 | 28 | 42 | 0 | 3 | 0 | 0 |
| 426.119234 | 426.11944 | 1451565 | 22 | 21 | 1 | 8 | 0 | 0 |
| 427.030544 | 427.030685 | 1850522 | 20 | 12 | 0 | 11 | 0 | 0 |
| 427.067159 | 427.06707 | 3921823 | 21 | 16 | 0 | 10 | 0 | 0 |
| 427.082225 | 427.082326 | 1855520 | 25 | 16 | 0 | 7 | 0 | 0 |
| 427.103516 | 427.103456 | 6376611 | 22 | 20 | 0 | 9 | 0 | 0 |
| 427.106962 | 427.106827 | 4014755 | 19 | 24 | 0 | 9 | 1 | 0 |
| 427.118655 | 427.118712 | 1565605 | 26 | 20 | 0 | 6 | 0 | 0 |
| 427.124607 | 427.124585 | 3144613 | 19 | 24 | 0 | 11 | 0 | 0 |
| 427.136835 | 427.136687 | 1395623 | 19 | 28 | 2 | 5 | 2 | 0 |
| 427.139808 | 427.139841 | 12660391 | 23 | 24 | 0 | 8 | 0 | 0 |
| 427.1432 | 427.143212 | 3931048 | 20 | 28 | 0 | 8 | 1 | 0 |
| 427.155148 | 427.155097 | 2015913 | 27 | 24 | 0 | 5 | 0 | 0 |
| 427.161009 | 427.160971 | 4740522 | 20 | 28 | 0 | 10 | 0 | 0 |
| 427.176258 | 427.176227 | 22065836 | 24 | 28 | 0 | 7 | 0 | 0 |
| 427.179336 | 427.179598 | 1618604 | 21 | 32 | 0 | 7 | 1 | 0 |
| 427.191682 | 427.191483 | 1553837 | 28 | 28 | 0 | 4 | 0 | 0 |
| 427.197446 | 427.197356 | 4063662 | 21 | 32 | 0 | 9 | 0 | 0 |
| 427.212617 | 427.212612 | 23900848 | 25 | 32 | 0 | 6 | 0 | 0 |
| 427.233859 | 427.233742 | 1952690 | 22 | 36 | 0 | 8 | 0 | 0 |
| 427.249015 | 427.248998 | 13976244 | 26 | 36 | 0 | 5 | 0 | 0 |
| 427.285387 | 427.285383 | 4818617 | 27 | 40 | 0 | 4 | 0 | 0 |
| 428.171178 | 428.171476 | 1447969 | 23 | 27 | 1 | 7 | 0 | 0 |
| 429.046298 | 429.046335 | 2244489 | 20 | 14 | 0 | 11 | 0 | 0 |
| 429.061634 | 429.061591 | 1839818 | 24 | 14 | 0 | 8 | 0 | 0 |
| 429.082691 | 429.08272 | 4231565 | 21 | 18 | 0 | 10 | 0 | 0 |
| 429.086029 | 429.086091 | 1521933 | 18 | 22 | 0 | 10 | 1 | 0 |
| 429.098066 | 429.097976 | 2605583 | 25 | 18 | 0 | 7 | 0 | 0 |
| 429.119106 | 429.119106 | 8635793 | 22 | 22 | 0 | 9 | 0 | 0 |
| 429.122447 | 429.122477 | 4927378 | 19 | 26 | 0 | 9 | 1 | 0 |
| 429.13428 | 429.134362 | 2471827 | 26 | 22 | 0 | 6 | 0 | 0 |
| 429.155488 | 429.155491 | 20581270 | 23 | 26 | 0 | 8 | 0 | 0 |
| 429.158932 | 429.158862 | 3499414 | 20 | 30 | 0 | 8 | 1 | 0 |
| 429.170667 | 429.170747 | 2816151 | 27 | 26 | 0 | 5 | 0 | 0 |
| 429.176803 | 429.176621 | 1638424 | 20 | 30 | 0 | 10 | 0 | 0 |
| 429.191853 | 429.191877 | 29207450 | 24 | 30 | 0 | 7 | 0 | 0 |
| 429.207051 | 429.207133 | 2659483 | 28 | 30 | 0 | 4 | 0 | 0 |
| 429.213018 | 429.213006 | 1425180 | 21 | 34 | 0 | 9 | 0 | 0 |
| 429.228274 | 429.228262 | 22354846 | 25 | 34 | 0 | 6 | 0 | 0 |
| 429.264702 | 429.264648 | 9151906 | 26 | 38 | 0 | 5 | 0 | 0 |
| 429.301113 | 429.301033 | 2384806 | 27 | 42 | 0 | 4 | 0 | 0 |
| 430.150759 | 430.15074 | 1636617 | 22 | 25 | 1 | 8 | 0 | 0 |
| 430.187063 | 430.187126 | 1687182 | 23 | 29 | 1 | 7 | 0 | 0 |
| 431.040704 | 431.040856 | 2048625 | 23 | 12 | 0 | 9 | 0 | 0 |
| 431.061948 | 431.061985 | 2043891 | 20 | 16 | 0 | 11 | 0 | 0 |
| 431.077319 | 431.077241 | 2711925 | 24 | 16 | 0 | 8 | 0 | 0 |
| 431.098448 | 431.09837 | 5169271 | 21 | 20 | 0 | 10 | 0 | 0 |
| 431.101725 | 431.101741 | 2490488 | 18 | 24 | 0 | 10 | 1 | 0 |
| 431.1136 | 431.113627 | 3329913 | 25 | 20 | 0 | 7 | 0 | 0 |
| 431.134726 | 431.134756 | 14986363 | 22 | 24 | 0 | 9 | 0 | 0 |
| 431.138237 | 431.138127 | 4323964 | 19 | 28 | 0 | 9 | 1 | 0 |
| 431.150078 | 431.150012 | 3842173 | 26 | 24 | 0 | 6 | 0 | 0 |
| 431.17116 | 431.171141 | 29547648 | 23 | 28 | 0 | 8 | 0 | 0 |
| 431.174571 | 431.174512 | 1737344 | 20 | 32 | 0 | 8 | 1 | 0 |
| 431.18643 | 431.186398 | 5119618 | 27 | 28 | 0 | 5 | 0 | 0 |
| 431.207547 | 431.207527 | 32228484 | 24 | 32 | 0 | 7 | 0 | 0 |
| 431.222812 | 431.222783 | 3438982 | 28 | 32 | 0 | 4 | 0 | 0 |
| 431.243941 | 431.243912 | 17632392 | 25 | 36 | 0 | 6 | 0 | 0 |
| 431.280246 | 431.280298 | 4284556 | 26 | 40 | 0 | 5 | 0 | 0 |
| 432.13004 | 432.130005 | 1626094 | 21 | 23 | 1 | 9 | 0 | 0 |
| 432.166438 | 432.16639 | 1606130 | 22 | 27 | 1 | 8 | 0 | 0 |
| 433.020047 | 433.02012 | 1376595 | 22 | 10 | 0 | 10 | 0 | 0 |
| 433.056573 | 433.056506 | 3190871 | 23 | 14 | 0 | 9 | 0 | 0 |
| 433.077606 | 433.077635 | 2463833 | 20 | 18 | 0 | 11 | 0 | 0 |
| 433.093038 | 433.092891 | 3664219 | 24 | 18 | 0 | 8 | 0 | 0 |
| 433.11398 | 433.11402 | 7787870 | 21 | 22 | 0 | 10 | 0 | 0 |
| 433.117434 | 433.117391 | 2214238 | 18 | 26 | 0 | 10 | 1 | 0 |
| 433.129253 | 433.129277 | 4288863 | 25 | 22 | 0 | 7 | 0 | 0 |
| 433.150445 | 433.150406 | 25621858 | 22 | 26 | 0 | 9 | 0 | 0 |
| 433.153558 | 433.153777 | 2072162 | 19 | 30 | 0 | 9 | 1 | 0 |
| 433.165762 | 433.165662 | 5750883 | 26 | 26 | 0 | 6 | 0 | 0 |
| 433.186821 | 433.186791 | 35745124 | 23 | 30 | 0 | 8 | 0 | 0 |
| 433.202041 | 433.202048 | 7252328 | 27 | 30 | 0 | 5 | 0 | 0 |
| 433.223158 | 433.223177 | 25204074 | 24 | 34 | 0 | 7 | 0 | 0 |
| 433.238455 | 433.238433 | 5427052 | 28 | 34 | 0 | 4 | 0 | 0 |
| 433.259617 | 433.259562 | 8946030 | 25 | 38 | 0 | 6 | 0 | 0 |
| 433.274947 | 433.274819 | 2309488 | 29 | 38 | 0 | 3 | 0 | 0 |
| 434.18221 | 434.18204 | 1421654 | 22 | 29 | 1 | 8 | 0 | 0 |
| 435.035848 | 435.03577 | 2541877 | 22 | 12 | 0 | 10 | 0 | 0 |
| 435.07217 | 435.072156 | 3860025 | 23 | 16 | 0 | 9 | 0 | 0 |
| 435.093269 | 435.093285 | 3053371 | 20 | 20 | 0 | 11 | 0 | 0 |
| 435.108549 | 435.108541 | 4421181 | 24 | 20 | 0 | 8 | 0 | 0 |
| 435.111976 | 435.111912 | 1484861 | 21 | 24 | 0 | 8 | 1 | 0 |
| 435.129664 | 435.129671 | 13802047 | 21 | 24 | 0 | 10 | 0 | 0 |
| 435.144977 | 435.144927 | 5959489 | 25 | 24 | 0 | 7 | 0 | 0 |
| 435.16607 | 435.166056 | 31956548 | 22 | 28 | 0 | 9 | 0 | 0 |
| 435.181314 | 435.181312 | 9483845 | 26 | 28 | 0 | 6 | 0 | 0 |
| 435.202471 | 435.202442 | 26197576 | 23 | 32 | 0 | 8 | 0 | 0 |
| 435.217759 | 435.217698 | 9530953 | 27 | 32 | 0 | 5 | 0 | 0 |
| 435.238835 | 435.238827 | 13016651 | 24 | 36 | 0 | 7 | 0 | 0 |
| 435.253991 | 435.254083 | 6686285 | 28 | 36 | 0 | 4 | 0 | 0 |
| 435.27523 | 435.275213 | 3304271 | 25 | 40 | 0 | 6 | 0 | 0 |
| 435.290573 | 435.290469 | 1873489 | 29 | 40 | 0 | 3 | 0 | 0 |
| 437.051469 | 437.05142 | 3094034 | 22 | 14 | 0 | 10 | 0 | 0 |
| 437.087892 | 437.087806 | 4588822 | 23 | 18 | 0 | 9 | 0 | 0 |
| 437.091228 | 437.091177 | 1638166 | 20 | 22 | 0 | 9 | 1 | 0 |
| 437.10891 | 437.108935 | 5166873 | 20 | 22 | 0 | 11 | 0 | 0 |
| 437.124157 | 437.124191 | 5645594 | 24 | 22 | 0 | 8 | 0 | 0 |
| 437.127691 | 437.127562 | 2294682 | 21 | 26 | 0 | 8 | 1 | 0 |
| 437.14535 | 437.145321 | 16761628 | 21 | 26 | 0 | 10 | 0 | 0 |
| 437.160559 | 437.160577 | 10423070 | 25 | 26 | 0 | 7 | 0 | 0 |
| 437.18174 | 437.181706 | 21679904 | 22 | 30 | 0 | 9 | 0 | 0 |
| 437.197034 | 437.196962 | 13719330 | 26 | 30 | 0 | 6 | 0 | 0 |
| 437.218178 | 437.218092 | 12796708 | 23 | 34 | 0 | 8 | 0 | 0 |
| 437.233414 | 437.233348 | 12786470 | 27 | 34 | 0 | 5 | 0 | 0 |
| 437.254648 | 437.254477 | 3866664 | 24 | 38 | 0 | 7 | 0 | 0 |
| 437.269733 | 437.269733 | 7543594 | 28 | 38 | 0 | 4 | 0 | 0 |
| 437.30615 | 437.306119 | 1776174 | 29 | 42 | 0 | 3 | 0 | 0 |
| 438.155688 | 438.155826 | 1448969 | 24 | 25 | 1 | 7 | 0 | 0 |
| 439.030884 | 439.030685 | 1525991 | 21 | 12 | 0 | 11 | 0 | 0 |
| 439.067063 | 439.06707 | 4002026 | 22 | 16 | 0 | 10 | 0 | 0 |
| 439.070492 | 439.070441 | 1379051 | 19 | 20 | 0 | 10 | 1 | 0 |
| 439.082306 | 439.082326 | 1502188 | 26 | 16 | 0 | 7 | 0 | 0 |
| 439.103419 | 439.103456 | 5393390 | 23 | 20 | 0 | 9 | 0 | 0 |
| 439.106803 | 439.106827 | 2699247 | 20 | 24 | 0 | 9 | 1 | 0 |
| 439.124625 | 439.124585 | 5569521 | 20 | 24 | 0 | 11 | 0 | 0 |
| 439.139842 | 439.139841 | 9334770 | 24 | 24 | 0 | 8 | 0 | 0 |
| 439.143057 | 439.143212 | 2543090 | 21 | 28 | 0 | 8 | 1 | 0 |
| 439.161011 | 439.160971 | 10741748 | 21 | 28 | 0 | 10 | 0 | 0 |
| 439.176253 | 439.176227 | 16066550 | 25 | 28 | 0 | 7 | 0 | 0 |
| 439.197341 | 439.197356 | 10218488 | 22 | 32 | 0 | 9 | 0 | 0 |
| 439.212619 | 439.212612 | 18728954 | 26 | 32 | 0 | 6 | 0 | 0 |
| 439.233775 | 439.233742 | 4054268 | 23 | 36 | 0 | 8 | 0 | 0 |
| 439.249019 | 439.248998 | 14127102 | 27 | 36 | 0 | 5 | 0 | 0 |
| 439.269996 | 439.270127 | 1383168 | 24 | 40 | 0 | 7 | 0 | 0 |
| 439.285384 | 439.285383 | 6217730 | 28 | 40 | 0 | 4 | 0 | 0 |
| 440.171202 | 440.171476 | 1420767 | 24 | 27 | 1 | 7 | 0 | 0 |
| 441.046266 | 441.046335 | 2371258 | 21 | 14 | 0 | 11 | 0 | 0 |
| 441.061919 | 441.061591 | 1464379 | 25 | 14 | 0 | 8 | 0 | 0 |
| 441.0828 | 441.08272 | 3860669 | 22 | 18 | 0 | 10 | 0 | 0 |
| 441.086193 | 441.086091 | 1939646 | 19 | 22 | 0 | 10 | 1 | 0 |
| 441.098188 | 441.097976 | 1667391 | 26 | 18 | 0 | 7 | 0 | 0 |
| 441.11915 | 441.119106 | 6956737 | 23 | 22 | 0 | 9 | 0 | 0 |
| 441.122357 | 441.122477 | 4231874 | 20 | 26 | 0 | 9 | 1 | 0 |
| 441.134269 | 441.134362 | 1610179 | 27 | 22 | 0 | 6 | 0 | 0 |
| 441.14029 | 441.140235 | 3828931 | 20 | 26 | 0 | 11 | 0 | 0 |
| 441.155536 | 441.155491 | 14918853 | 24 | 26 | 0 | 8 | 0 | 0 |
| 441.158906 | 441.158862 | 3835589 | 21 | 30 | 0 | 8 | 1 | 0 |
| 441.170664 | 441.170747 | 1991622 | 28 | 26 | 0 | 5 | 0 | 0 |
| 441.176556 | 441.176621 | 4394695 | 21 | 30 | 0 | 10 | 0 | 0 |
| 441.191906 | 441.191877 | 22196424 | 25 | 30 | 0 | 7 | 0 | 0 |
| 441.207177 | 441.207133 | 1544778 | 29 | 30 | 0 | 4 | 0 | 0 |
| 441.213011 | 441.213006 | 2966475 | 22 | 34 | 0 | 9 | 0 | 0 |
| 441.228231 | 441.228262 | 20039884 | 26 | 34 | 0 | 6 | 0 | 0 |
| 441.249687 | 441.249392 | 1558734 | 23 | 38 | 0 | 8 | 0 | 0 |
| 441.264651 | 441.264648 | 11715792 | 27 | 38 | 0 | 5 | 0 | 0 |
| 441.301002 | 441.301033 | 3863764 | 28 | 42 | 0 | 4 | 0 | 0 |
| 442.150774 | 442.15074 | 1570091 | 23 | 25 | 1 | 8 | 0 | 0 |
| 442.187353 | 442.187126 | 1509678 | 24 | 29 | 1 | 7 | 0 | 0 |
| 443.062107 | 443.061985 | 2416263 | 21 | 16 | 0 | 11 | 0 | 0 |
| 443.077202 | 443.077241 | 2407304 | 25 | 16 | 0 | 8 | 0 | 0 |
| 443.098393 | 443.09837 | 4878731 | 22 | 20 | 0 | 10 | 0 | 0 |
| 443.101777 | 443.101741 | 3517067 | 19 | 24 | 0 | 10 | 1 | 0 |
| 443.11358 | 443.113627 | 2516364 | 26 | 20 | 0 | 7 | 0 | 0 |
| 443.134737 | 443.134756 | 10609038 | 23 | 24 | 0 | 9 | 0 | 0 |
| 443.138234 | 443.138127 | 5482895 | 20 | 28 | 0 | 9 | 1 | 0 |
| 443.149848 | 443.150012 | 2679696 | 27 | 24 | 0 | 6 | 0 | 0 |
| 443.155919 | 443.155885 | 1471248 | 20 | 28 | 0 | 11 | 0 | 0 |
| 443.171162 | 443.171141 | 21991826 | 24 | 28 | 0 | 8 | 0 | 0 |
| 443.174505 | 443.174512 | 2199954 | 21 | 32 | 0 | 8 | 1 | 0 |
| 443.186563 | 443.186398 | 2259539 | 28 | 28 | 0 | 5 | 0 | 0 |
| 443.19251 | 443.192271 | 1651860 | 21 | 32 | 0 | 10 | 0 | 0 |
| 443.207553 | 443.207527 | 27435414 | 25 | 32 | 0 | 7 | 0 | 0 |
| 443.222781 | 443.222783 | 2100887 | 29 | 32 | 0 | 4 | 0 | 0 |
| 443.2439 | 443.243912 | 18666906 | 26 | 36 | 0 | 6 | 0 | 0 |
| 443.280359 | 443.280298 | 7802269 | 27 | 40 | 0 | 5 | 0 | 0 |
| 443.316736 | 443.316683 | 1698208 | 28 | 44 | 0 | 4 | 0 | 0 |
| 444.166525 | 444.16639 | 1443061 | 23 | 27 | 1 | 8 | 0 | 0 |
| 444.202654 | 444.202776 | 1539320 | 24 | 31 | 1 | 7 | 0 | 0 |
| 445.041381 | 445.041249 | 1835595 | 20 | 14 | 0 | 12 | 0 | 0 |
| 445.056511 | 445.056506 | 2601804 | 24 | 14 | 0 | 9 | 0 | 0 |
| 445.077802 | 445.077635 | 3040334 | 21 | 18 | 0 | 11 | 0 | 0 |
| 445.093022 | 445.092891 | 3011664 | 25 | 18 | 0 | 8 | 0 | 0 |
| 445.114029 | 445.11402 | 6569042 | 22 | 22 | 0 | 10 | 0 | 0 |
| 445.117333 | 445.117391 | 4268114 | 19 | 26 | 0 | 10 | 1 | 0 |
| 445.129378 | 445.129277 | 3471699 | 26 | 22 | 0 | 7 | 0 | 0 |
| 445.15039 | 445.150406 | 17328726 | 23 | 26 | 0 | 9 | 0 | 0 |
| 445.153732 | 445.153777 | 4158038 | 20 | 30 | 0 | 9 | 1 | 0 |
| 445.165769 | 445.165662 | 3757143 | 27 | 26 | 0 | 6 | 0 | 0 |
| 445.186837 | 445.186791 | 29733464 | 24 | 30 | 0 | 8 | 0 | 0 |
| 445.202091 | 445.202048 | 4044378 | 28 | 30 | 0 | 5 | 0 | 0 |
| 445.223216 | 445.223177 | 28897884 | 25 | 34 | 0 | 7 | 0 | 0 |
| 445.238443 | 445.238433 | 2963038 | 29 | 34 | 0 | 4 | 0 | 0 |
| 445.259525 | 445.259562 | 12350048 | 26 | 38 | 0 | 6 | 0 | 0 |
| 446.145569 | 446.145655 | 1515445 | 22 | 25 | 1 | 9 | 0 | 0 |
| 447.035643 | 447.03577 | 1820682 | 23 | 12 | 0 | 10 | 0 | 0 |
| 447.072192 | 447.072156 | 3332238 | 24 | 16 | 0 | 9 | 0 | 0 |
| 447.093167 | 447.093285 | 3938064 | 21 | 20 | 0 | 11 | 0 | 0 |
| 447.108535 | 447.108541 | 3968273 | 25 | 20 | 0 | 8 | 0 | 0 |
| 447.12965 | 447.129671 | 11154195 | 22 | 24 | 0 | 10 | 0 | 0 |
| 447.132942 | 447.133041 | 2542227 | 19 | 28 | 0 | 10 | 1 | 0 |
| 447.144887 | 447.144927 | 4675349 | 26 | 24 | 0 | 7 | 0 | 0 |
| 447.166127 | 447.166056 | 26573590 | 23 | 28 | 0 | 9 | 0 | 0 |
| 447.181357 | 447.181312 | 6073112 | 27 | 28 | 0 | 6 | 0 | 0 |
| 447.202436 | 447.202442 | 32123674 | 24 | 32 | 0 | 8 | 0 | 0 |
| 447.217636 | 447.217698 | 5407515 | 28 | 32 | 0 | 5 | 0 | 0 |
| 447.238809 | 447.238827 | 21066526 | 25 | 36 | 0 | 7 | 0 | 0 |
| 447.254019 | 447.254083 | 3578911 | 29 | 36 | 0 | 4 | 0 | 0 |
| 447.27521 | 447.275213 | 6208289 | 26 | 40 | 0 | 6 | 0 | 0 |
| 448.151358 | 448.151409 | 1474162 | 24 | 23 | 3 | 6 | 0 | 0 |
| 449.051418 | 449.05142 | 3112389 | 23 | 14 | 0 | 10 | 0 | 0 |
| 449.087965 | 449.087806 | 3984840 | 24 | 18 | 0 | 9 | 0 | 0 |
| 449.108955 | 449.108935 | 5049290 | 21 | 22 | 0 | 11 | 0 | 0 |
| 449.124145 | 449.124191 | 5251020 | 25 | 22 | 0 | 8 | 0 | 0 |
| 449.145367 | 449.145321 | 17593294 | 22 | 26 | 0 | 10 | 0 | 0 |
| 449.160634 | 449.160577 | 6697935 | 26 | 26 | 0 | 7 | 0 | 0 |
| 449.181683 | 449.181706 | 27085776 | 23 | 30 | 0 | 9 | 0 | 0 |
| 449.196983 | 449.196962 | 8349138 | 27 | 30 | 0 | 6 | 0 | 0 |
| 449.218098 | 449.218092 | 22518740 | 24 | 34 | 0 | 8 | 0 | 0 |
| 449.233334 | 449.233348 | 8191958 | 28 | 34 | 0 | 5 | 0 | 0 |
| 449.254521 | 449.254477 | 11240407 | 25 | 38 | 0 | 7 | 0 | 0 |
| 449.269833 | 449.269733 | 3995097 | 29 | 38 | 0 | 4 | 0 | 0 |
| 449.290825 | 449.290863 | 2123483 | 26 | 42 | 0 | 6 | 0 | 0 |
| 451.030988 | 451.030685 | 1566838 | 22 | 12 | 0 | 11 | 0 | 0 |
| 451.067099 | 451.06707 | 3590777 | 23 | 16 | 0 | 10 | 0 | 0 |
| 451.088344 | 451.0882 | 2066555 | 20 | 20 | 0 | 12 | 0 | 0 |
| 451.103394 | 451.103456 | 4557436 | 24 | 20 | 0 | 9 | 0 | 0 |
| 451.107029 | 451.106827 | 1751165 | 21 | 24 | 0 | 9 | 1 | 0 |
| 451.124592 | 451.124585 | 7336574 | 21 | 24 | 0 | 11 | 0 | 0 |
| 451.139917 | 451.139841 | 6537344 | 25 | 24 | 0 | 8 | 0 | 0 |
| 451.143378 | 451.143212 | 2083712 | 22 | 28 | 0 | 8 | 1 | 0 |
| 451.160996 | 451.160971 | 17711234 | 22 | 28 | 0 | 10 | 0 | 0 |
| 451.176291 | 451.176227 | 10460291 | 26 | 28 | 0 | 7 | 0 | 0 |
| 451.19738 | 451.197356 | 18086020 | 23 | 32 | 0 | 9 | 0 | 0 |
| 451.212597 | 451.212612 | 12722310 | 27 | 32 | 0 | 6 | 0 | 0 |
| 451.233736 | 451.233742 | 10422408 | 24 | 36 | 0 | 8 | 0 | 0 |
| 451.248983 | 451.248998 | 10700937 | 28 | 36 | 0 | 5 | 0 | 0 |
| 451.270149 | 451.270127 | 3284875 | 25 | 40 | 0 | 7 | 0 | 0 |
| 451.285444 | 451.285383 | 4214156 | 29 | 40 | 0 | 4 | 0 | 0 |
| 452.182632 | 452.182709 | 1588186 | 24 | 27 | 3 | 6 | 0 | 0 |
| 453.046162 | 453.046335 | 2574883 | 22 | 14 | 0 | 11 | 0 | 0 |
| 453.082548 | 453.08272 | 3916070 | 23 | 18 | 0 | 10 | 0 | 0 |
| 453.086038 | 453.086091 | 1534758 | 20 | 22 | 0 | 10 | 1 | 0 |
| 453.098154 | 453.097976 | 1716647 | 27 | 18 | 0 | 7 | 0 | 0 |
| 453.119135 | 453.119106 | 5764393 | 24 | 22 | 0 | 9 | 0 | 0 |
| 453.12239 | 453.122477 | 3166505 | 21 | 26 | 0 | 9 | 1 | 0 |
| 453.140198 | 453.140235 | 7563051 | 21 | 26 | 0 | 11 | 0 | 0 |
| 453.155548 | 453.155491 | 9850668 | 25 | 26 | 0 | 8 | 0 | 0 |
| 453.158686 | 453.158862 | 3378732 | 22 | 30 | 0 | 8 | 1 | 0 |
| 453.176607 | 453.176621 | 10928430 | 22 | 30 | 0 | 10 | 0 | 0 |
| 453.19186 | 453.191877 | 15844655 | 26 | 30 | 0 | 7 | 0 | 0 |
| 453.212959 | 453.213006 | 8378673 | 23 | 34 | 0 | 9 | 0 | 0 |
| 453.228273 | 453.228262 | 16735538 | 27 | 34 | 0 | 6 | 0 | 0 |
| 453.249413 | 453.249392 | 3759412 | 24 | 38 | 0 | 8 | 0 | 0 |
| 453.26468 | 453.264648 | 10438965 | 28 | 38 | 0 | 5 | 0 | 0 |
| 453.301052 | 453.301033 | 3738680 | 29 | 42 | 0 | 4 | 0 | 0 |
| 455.062156 | 455.061985 | 2733513 | 22 | 16 | 0 | 11 | 0 | 0 |
| 455.077201 | 455.077241 | 1775434 | 26 | 16 | 0 | 8 | 0 | 0 |
| 455.098398 | 455.09837 | 4248012 | 23 | 20 | 0 | 10 | 0 | 0 |
| 455.101923 | 455.101741 | 2583500 | 20 | 24 | 0 | 10 | 1 | 0 |
| 455.113675 | 455.113627 | 1937869 | 27 | 20 | 0 | 7 | 0 | 0 |
| 455.119324 | 455.1195 | 1552717 | 20 | 24 | 0 | 12 | 0 | 0 |
| 455.134757 | 455.134756 | 8065999 | 24 | 24 | 0 | 9 | 0 | 0 |
| 455.138201 | 455.138127 | 4557775 | 21 | 28 | 0 | 9 | 1 | 0 |
| 455.150094 | 455.150012 | 1434960 | 28 | 24 | 0 | 6 | 0 | 0 |
| 455.155795 | 455.155885 | 4061136 | 21 | 28 | 0 | 11 | 0 | 0 |
| 455.171193 | 455.171141 | 15208913 | 25 | 28 | 0 | 8 | 0 | 0 |
| 455.174545 | 455.174512 | 3088594 | 22 | 32 | 0 | 8 | 1 | 0 |
| 455.18644 | 455.186398 | 1467987 | 29 | 28 | 0 | 5 | 0 | 0 |
| 455.192245 | 455.192271 | 4256211 | 22 | 32 | 0 | 10 | 0 | 0 |
| 455.207551 | 455.207527 | 21206484 | 26 | 32 | 0 | 7 | 0 | 0 |
| 455.228784 | 455.228656 | 2906838 | 23 | 36 | 0 | 9 | 0 | 0 |
| 455.243921 | 455.243912 | 18288088 | 27 | 36 | 0 | 6 | 0 | 0 |
| 455.280297 | 455.280298 | 10024410 | 28 | 40 | 0 | 5 | 0 | 0 |
| 455.316497 | 455.316683 | 2099933 | 29 | 44 | 0 | 4 | 0 | 0 |
| 456.166555 | 456.16639 | 1565088 | 24 | 27 | 1 | 8 | 0 | 0 |
| 457.041463 | 457.041249 | 1496676 | 21 | 14 | 0 | 12 | 0 | 0 |
| 457.056719 | 457.056506 | 2025061 | 25 | 14 | 0 | 9 | 0 | 0 |
| 457.077715 | 457.077635 | 2996839 | 22 | 18 | 0 | 11 | 0 | 0 |
| 457.081078 | 457.081006 | 1603175 | 19 | 22 | 0 | 11 | 1 | 0 |
| 457.092674 | 457.092891 | 2280168 | 26 | 18 | 0 | 8 | 0 | 0 |
| 457.113956 | 457.11402 | 5766250 | 23 | 22 | 0 | 10 | 0 | 0 |
| 457.117279 | 457.117391 | 4048490 | 20 | 26 | 0 | 10 | 1 | 0 |
| 457.129256 | 457.129277 | 2507115 | 27 | 22 | 0 | 7 | 0 | 0 |
| 457.153912 | 457.153777 | 4102253 | 21 | 30 | 0 | 9 | 1 | 0 |
| 457.165755 | 457.165662 | 2716782 | 28 | 26 | 0 | 6 | 0 | 0 |
| 457.171407 | 457.171535 | 1563758 | 21 | 30 | 0 | 11 | 0 | 0 |
| 457.186798 | 457.186791 | 23643760 | 25 | 30 | 0 | 8 | 0 | 0 |
| 457.202248 | 457.202048 | 2607728 | 29 | 30 | 0 | 5 | 0 | 0 |
| 457.207744 | 457.207921 | 1702001 | 22 | 34 | 0 | 10 | 0 | 0 |
| 457.223188 | 457.223177 | 24764018 | 26 | 34 | 0 | 7 | 0 | 0 |
| 457.259557 | 457.259562 | 16250485 | 27 | 38 | 0 | 6 | 0 | 0 |
| 457.295963 | 457.295948 | 6153336 | 28 | 42 | 0 | 5 | 0 | 0 |
| 458.182271 | 458.18204 | 1509306 | 24 | 29 | 1 | 8 | 0 | 0 |
| 459.035895 | 459.03577 | 1515513 | 24 | 12 | 0 | 10 | 0 | 0 |
| 459.056971 | 459.0569 | 1734906 | 21 | 16 | 0 | 12 | 0 | 0 |
| 459.072068 | 459.072156 | 3029243 | 25 | 16 | 0 | 9 | 0 | 0 |
| 459.093166 | 459.093285 | 3553533 | 22 | 20 | 0 | 11 | 0 | 0 |
| 459.096808 | 459.096656 | 1758717 | 19 | 24 | 0 | 11 | 1 | 0 |
| 459.108554 | 459.108541 | 2828542 | 26 | 20 | 0 | 8 | 0 | 0 |
| 459.129689 | 459.129671 | 8683263 | 23 | 24 | 0 | 10 | 0 | 0 |
| 459.133146 | 459.133041 | 3958912 | 20 | 28 | 0 | 10 | 1 | 0 |
| 459.144798 | 459.144927 | 2969857 | 27 | 24 | 0 | 7 | 0 | 0 |
| 459.166085 | 459.166056 | 19797762 | 24 | 28 | 0 | 9 | 0 | 0 |
| 459.169486 | 459.169427 | 2386690 | 21 | 32 | 0 | 9 | 1 | 0 |
| 459.181453 | 459.181312 | 3327235 | 28 | 28 | 0 | 6 | 0 | 0 |
| 459.202433 | 459.202442 | 29826820 | 25 | 32 | 0 | 8 | 0 | 0 |
| 459.217887 | 459.217698 | 3121414 | 29 | 32 | 0 | 5 | 0 | 0 |
| 459.238846 | 459.238827 | 21845768 | 26 | 36 | 0 | 7 | 0 | 0 |
| 459.253996 | 459.254083 | 1860616 | 30 | 36 | 0 | 4 | 0 | 0 |
| 459.275195 | 459.275213 | 11008778 | 27 | 40 | 0 | 6 | 0 | 0 |
| 459.311774 | 459.311598 | 2720397 | 28 | 44 | 0 | 5 | 0 | 0 |
| 460.16116 | 460.161305 | 1421385 | 23 | 27 | 1 | 9 | 0 | 0 |
| 460.19782 | 460.197691 | 1416268 | 24 | 31 | 1 | 8 | 0 | 0 |
| 461.051351 | 461.05142 | 2475143 | 24 | 14 | 0 | 10 | 0 | 0 |
| 461.072527 | 461.07255 | 1776264 | 21 | 18 | 0 | 12 | 0 | 0 |
| 461.087881 | 461.087806 | 3402633 | 25 | 18 | 0 | 9 | 0 | 0 |
| 461.10896 | 461.108935 | 4047499 | 22 | 22 | 0 | 11 | 0 | 0 |
| 461.112466 | 461.112306 | 1726859 | 19 | 26 | 0 | 11 | 1 | 0 |
| 461.123992 | 461.124191 | 3484684 | 26 | 22 | 0 | 8 | 0 | 0 |
| 461.145362 | 461.145321 | 13426573 | 23 | 26 | 0 | 10 | 0 | 0 |
| 461.148603 | 461.148691 | 2430989 | 20 | 30 | 0 | 10 | 1 | 0 |
| 461.160659 | 461.160577 | 4393870 | 27 | 26 | 0 | 7 | 0 | 0 |
| 461.181711 | 461.181706 | 27864976 | 24 | 30 | 0 | 9 | 0 | 0 |
| 461.196983 | 461.196962 | 4941969 | 28 | 30 | 0 | 6 | 0 | 0 |
| 461.218097 | 461.218092 | 30312338 | 25 | 34 | 0 | 8 | 0 | 0 |
| 461.233379 | 461.233348 | 4266131 | 29 | 34 | 0 | 5 | 0 | 0 |
| 461.254436 | 461.254477 | 13970324 | 26 | 38 | 0 | 7 | 0 | 0 |
| 461.269857 | 461.269733 | 2088342 | 30 | 38 | 0 | 4 | 0 | 0 |
| 461.290866 | 461.290863 | 5254551 | 27 | 42 | 0 | 6 | 0 | 0 |
| 463.030894 | 463.030685 | 1737994 | 23 | 12 | 0 | 11 | 0 | 0 |
| 463.066966 | 463.06707 | 3471884 | 24 | 16 | 0 | 10 | 0 | 0 |
| 463.087964 | 463.0882 | 1687565 | 21 | 20 | 0 | 12 | 0 | 0 |
| 463.10351 | 463.103456 | 4227599 | 25 | 20 | 0 | 9 | 0 | 0 |
| 463.124587 | 463.124585 | 6393360 | 22 | 24 | 0 | 11 | 0 | 0 |
| 463.139892 | 463.139841 | 4626449 | 26 | 24 | 0 | 8 | 0 | 0 |
| 463.161003 | 463.160971 | 18087954 | 23 | 28 | 0 | 10 | 0 | 0 |
| 463.176224 | 463.176227 | 7240211 | 27 | 28 | 0 | 7 | 0 | 0 |
| 463.19741 | 463.197356 | 26523668 | 24 | 32 | 0 | 9 | 0 | 0 |
| 463.212709 | 463.212612 | 7622678 | 28 | 32 | 0 | 6 | 0 | 0 |
| 463.233786 | 463.233742 | 19906582 | 25 | 36 | 0 | 8 | 0 | 0 |
| 463.248858 | 463.248998 | 5368856 | 29 | 36 | 0 | 5 | 0 | 0 |
| 463.270008 | 463.270127 | 6961177 | 26 | 40 | 0 | 7 | 0 | 0 |
| 463.28526 | 463.285383 | 1705626 | 30 | 40 | 0 | 4 | 0 | 0 |
| 465.046236 | 465.046335 | 2567815 | 23 | 14 | 0 | 11 | 0 | 0 |
| 465.082713 | 465.08272 | 3401353 | 24 | 18 | 0 | 10 | 0 | 0 |
| 465.103839 | 465.10385 | 2626186 | 21 | 22 | 0 | 12 | 0 | 0 |
| 465.119273 | 465.119106 | 4588171 | 25 | 22 | 0 | 9 | 0 | 0 |
| 465.122392 | 465.122477 | 1971595 | 22 | 26 | 0 | 9 | 1 | 0 |
| 465.140345 | 465.140235 | 9829516 | 22 | 26 | 0 | 11 | 0 | 0 |
| 465.155518 | 465.155491 | 7154829 | 26 | 26 | 0 | 8 | 0 | 0 |
| 465.158364 | 465.158482 | 1713037 | 25 | 27 | 2 | 5 | 0 | 1 |
| 465.176645 | 465.176621 | 17610894 | 23 | 30 | 0 | 10 | 0 | 0 |
| 465.191828 | 465.191877 | 9831567 | 27 | 30 | 0 | 7 | 0 | 0 |
| 465.212982 | 465.213006 | 15997072 | 24 | 34 | 0 | 9 | 0 | 0 |
| 465.228287 | 465.228262 | 10751121 | 28 | 34 | 0 | 6 | 0 | 0 |
| 465.249422 | 465.249392 | 8954515 | 25 | 38 | 0 | 8 | 0 | 0 |
| 465.26469 | 465.264648 | 6379668 | 29 | 38 | 0 | 5 | 0 | 0 |
| 465.285984 | 465.285777 | 2043541 | 26 | 42 | 0 | 7 | 0 | 0 |
| 465.301227 | 465.301033 | 1472918 | 30 | 42 | 0 | 4 | 0 | 0 |
| 466.162231 | 466.161974 | 1408456 | 24 | 25 | 3 | 7 | 0 | 0 |
| 467.061916 | 467.061985 | 3062522 | 23 | 16 | 0 | 11 | 0 | 0 |
| 467.077419 | 467.077241 | 1527163 | 27 | 16 | 0 | 8 | 0 | 0 |
| 467.098302 | 467.09837 | 4316924 | 24 | 20 | 0 | 10 | 0 | 0 |
| 467.101783 | 467.101741 | 2151420 | 21 | 24 | 0 | 10 | 1 | 0 |
| 467.113842 | 467.113627 | 1633789 | 28 | 20 | 0 | 7 | 0 | 0 |
| 467.119583 | 467.1195 | 2932733 | 21 | 24 | 0 | 12 | 0 | 0 |
| 467.134738 | 467.134756 | 6313726 | 25 | 24 | 0 | 9 | 0 | 0 |
| 467.138114 | 467.138127 | 3786494 | 22 | 28 | 0 | 9 | 1 | 0 |
| 467.15002 | 467.150012 | 1741823 | 29 | 24 | 0 | 6 | 0 | 0 |
| 467.155815 | 467.155885 | 6979839 | 22 | 28 | 0 | 11 | 0 | 0 |
| 467.171213 | 467.171141 | 10172160 | 26 | 28 | 0 | 8 | 0 | 0 |
| 467.174479 | 467.174512 | 2554880 | 23 | 32 | 0 | 8 | 1 | 0 |
| 467.192255 | 467.192271 | 9664769 | 23 | 32 | 0 | 10 | 0 | 0 |
| 467.207549 | 467.207527 | 14603522 | 27 | 32 | 0 | 7 | 0 | 0 |
| 467.22864 | 467.228656 | 6938883 | 24 | 36 | 0 | 9 | 0 | 0 |
| 467.243942 | 467.243912 | 14240004 | 28 | 36 | 0 | 6 | 0 | 0 |
| 467.26497 | 467.265042 | 2877701 | 25 | 40 | 0 | 8 | 0 | 0 |
| 467.280429 | 467.280298 | 7283462 | 29 | 40 | 0 | 5 | 0 | 0 |
| 468.202761 | 468.202776 | 1466935 | 26 | 31 | 1 | 7 | 0 | 0 |
| 469.041339 | 469.041249 | 1976162 | 22 | 14 | 0 | 12 | 0 | 0 |
| 469.056583 | 469.056506 | 1430371 | 26 | 14 | 0 | 9 | 0 | 0 |
| 469.077582 | 469.077635 | 3513956 | 23 | 18 | 0 | 11 | 0 | 0 |
| 469.080845 | 469.081006 | 1379940 | 20 | 22 | 0 | 11 | 1 | 0 |
| 469.092836 | 469.092891 | 1612389 | 27 | 18 | 0 | 8 | 0 | 0 |
| 469.114161 | 469.11402 | 4581734 | 24 | 22 | 0 | 10 | 0 | 0 |
| 469.117327 | 469.117391 | 3023974 | 21 | 26 | 0 | 10 | 1 | 0 |
| 469.129521 | 469.129277 | 2008679 | 28 | 22 | 0 | 7 | 0 | 0 |
| 469.135242 | 469.13515 | 2332775 | 21 | 26 | 0 | 12 | 0 | 0 |
| 469.150368 | 469.150406 | 9457000 | 25 | 26 | 0 | 9 | 0 | 0 |
| 469.15371 | 469.153777 | 4531560 | 22 | 30 | 0 | 9 | 1 | 0 |
| 469.165644 | 469.165662 | 1678441 | 29 | 26 | 0 | 6 | 0 | 0 |
| 469.171474 | 469.171535 | 4342633 | 22 | 30 | 0 | 11 | 0 | 0 |
| 469.186787 | 469.186791 | 15837546 | 26 | 30 | 0 | 8 | 0 | 0 |
| 469.201842 | 469.202048 | 1408363 | 30 | 30 | 0 | 5 | 0 | 0 |
| 469.207989 | 469.207921 | 3729003 | 23 | 34 | 0 | 10 | 0 | 0 |
| 469.223179 | 469.223177 | 19323244 | 27 | 34 | 0 | 7 | 0 | 0 |
| 469.244454 | 469.244306 | 2439021 | 24 | 38 | 0 | 9 | 0 | 0 |
| 469.259606 | 469.259562 | 15769965 | 28 | 38 | 0 | 6 | 0 | 0 |
| 469.295894 | 469.295948 | 6410095 | 29 | 42 | 0 | 5 | 0 | 0 |
| 470.182162 | 470.18204 | 1553306 | 25 | 29 | 1 | 8 | 0 | 0 |
| 471.056711 | 471.0569 | 2123203 | 22 | 16 | 0 | 12 | 0 | 0 |
| 471.071729 | 471.072156 | 1651140 | 26 | 16 | 0 | 9 | 0 | 0 |
| 471.093117 | 471.093285 | 3617733 | 23 | 20 | 0 | 11 | 0 | 0 |
| 471.096566 | 471.096656 | 2021573 | 20 | 24 | 0 | 11 | 1 | 0 |
| 471.108654 | 471.108541 | 2348741 | 27 | 20 | 0 | 8 | 0 | 0 |
| 471.129615 | 471.129671 | 6360007 | 24 | 24 | 0 | 10 | 0 | 0 |
| 471.132982 | 471.133041 | 3750471 | 21 | 28 | 0 | 10 | 1 | 0 |
| 471.144848 | 471.144927 | 1985735 | 28 | 24 | 0 | 7 | 0 | 0 |
| 471.166063 | 471.166056 | 13807048 | 25 | 28 | 0 | 9 | 0 | 0 |
| 471.169511 | 471.169427 | 3908040 | 22 | 32 | 0 | 9 | 1 | 0 |
| 471.181312 | 471.181312 | 2091209 | 29 | 28 | 0 | 6 | 0 | 0 |
| 471.202462 | 471.202442 | 23202250 | 26 | 32 | 0 | 8 | 0 | 0 |
| 471.217503 | 471.217698 | 1547018 | 30 | 32 | 0 | 5 | 0 | 0 |
| 471.223727 | 471.223571 | 1413067 | 23 | 36 | 0 | 10 | 0 | 0 |
| 471.238836 | 471.238827 | 22987212 | 27 | 36 | 0 | 7 | 0 | 0 |
| 471.275215 | 471.275213 | 13690317 | 28 | 40 | 0 | 6 | 0 | 0 |
| 471.311627 | 471.311598 | 4583375 | 29 | 44 | 0 | 5 | 0 | 0 |
| 472.19769 | 472.197691 | 1389685 | 25 | 31 | 1 | 8 | 0 | 0 |
| 473.051381 | 473.05142 | 1727513 | 25 | 14 | 0 | 10 | 0 | 0 |
| 473.072704 | 473.07255 | 2534938 | 22 | 18 | 0 | 12 | 0 | 0 |
| 473.087887 | 473.087806 | 3361818 | 26 | 18 | 0 | 9 | 0 | 0 |
| 473.10891 | 473.108935 | 3891995 | 23 | 22 | 0 | 11 | 0 | 0 |
| 473.112374 | 473.112306 | 2770971 | 20 | 26 | 0 | 11 | 1 | 0 |
| 473.124296 | 473.124191 | 2573852 | 27 | 22 | 0 | 8 | 0 | 0 |
| 473.145379 | 473.145321 | 9619996 | 24 | 26 | 0 | 10 | 0 | 0 |
| 473.148754 | 473.148691 | 3228701 | 21 | 30 | 0 | 10 | 1 | 0 |
| 473.160419 | 473.160577 | 3067421 | 28 | 26 | 0 | 7 | 0 | 0 |
| 473.181717 | 473.181706 | 22149662 | 25 | 30 | 0 | 9 | 0 | 0 |
| 473.196991 | 473.196962 | 2886431 | 29 | 30 | 0 | 6 | 0 | 0 |
| 473.218107 | 473.218092 | 26315296 | 26 | 34 | 0 | 8 | 0 | 0 |
| 473.233476 | 473.233348 | 2210720 | 30 | 34 | 0 | 5 | 0 | 0 |
| 473.254498 | 473.254477 | 20068896 | 27 | 38 | 0 | 7 | 0 | 0 |
| 473.290921 | 473.290863 | 9618978 | 28 | 42 | 0 | 6 | 0 | 0 |
| 473.327266 | 473.327248 | 2351140 | 29 | 46 | 0 | 5 | 0 | 0 |
| 475.067004 | 475.06707 | 2763877 | 25 | 16 | 0 | 10 | 0 | 0 |
| 475.088205 | 475.0882 | 1945190 | 22 | 20 | 0 | 12 | 0 | 0 |
| 475.103501 | 475.103456 | 3415142 | 26 | 20 | 0 | 9 | 0 | 0 |
| 475.124685 | 475.124585 | 5149287 | 23 | 24 | 0 | 11 | 0 | 0 |
| 475.127926 | 475.127956 | 2303463 | 20 | 28 | 0 | 11 | 1 | 0 |
| 475.139873 | 475.139841 | 3628136 | 27 | 24 | 0 | 8 | 0 | 0 |
| 475.160949 | 475.160971 | 15889000 | 24 | 28 | 0 | 10 | 0 | 0 |
| 475.164383 | 475.164341 | 1916872 | 21 | 32 | 0 | 10 | 1 | 0 |
| 475.176199 | 475.176227 | 3887977 | 28 | 28 | 0 | 7 | 0 | 0 |
| 475.197406 | 475.197356 | 27994730 | 25 | 32 | 0 | 9 | 0 | 0 |
| 475.212578 | 475.212612 | 3898986 | 29 | 32 | 0 | 6 | 0 | 0 |
| 475.233735 | 475.233742 | 24070762 | 26 | 36 | 0 | 8 | 0 | 0 |
| 475.249028 | 475.248998 | 2876011 | 30 | 36 | 0 | 5 | 0 | 0 |
| 475.270145 | 475.270127 | 13647468 | 27 | 40 | 0 | 7 | 0 | 0 |
| 475.306586 | 475.306513 | 4065901 | 28 | 44 | 0 | 6 | 0 | 0 |
| 477.0462 | 477.046335 | 2064678 | 24 | 14 | 0 | 11 | 0 | 0 |
| 477.082698 | 477.08272 | 3104679 | 25 | 18 | 0 | 10 | 0 | 0 |
| 477.103722 | 477.10385 | 2411687 | 22 | 22 | 0 | 12 | 0 | 0 |
| 477.118986 | 477.119106 | 4143272 | 26 | 22 | 0 | 9 | 0 | 0 |
| 477.140308 | 477.140235 | 8367785 | 23 | 26 | 0 | 11 | 0 | 0 |
| 477.155601 | 477.155491 | 4608681 | 27 | 26 | 0 | 8 | 0 | 0 |
| 477.176579 | 477.176621 | 19284650 | 24 | 30 | 0 | 10 | 0 | 0 |
| 477.191807 | 477.191877 | 6017706 | 28 | 30 | 0 | 7 | 0 | 0 |
| 477.213004 | 477.213006 | 24552106 | 25 | 34 | 0 | 9 | 0 | 0 |
| 477.22828 | 477.228262 | 5533355 | 29 | 34 | 0 | 6 | 0 | 0 |
| 477.249407 | 477.249392 | 15415980 | 26 | 38 | 0 | 8 | 0 | 0 |
| 477.264784 | 477.264648 | 3070636 | 30 | 38 | 0 | 5 | 0 | 0 |
| 479.062053 | 479.061985 | 2557405 | 24 | 16 | 0 | 11 | 0 | 0 |
| 479.098359 | 479.09837 | 3597022 | 25 | 20 | 0 | 10 | 0 | 0 |
| 479.1196 | 479.1195 | 3375966 | 22 | 24 | 0 | 12 | 0 | 0 |
| 479.134815 | 479.134756 | 4761311 | 26 | 24 | 0 | 9 | 0 | 0 |
| 479.137922 | 479.138127 | 1569247 | 23 | 28 | 0 | 9 | 1 | 0 |
| 479.155959 | 479.155885 | 10993375 | 23 | 28 | 0 | 11 | 0 | 0 |
| 479.171199 | 479.171141 | 7191264 | 27 | 28 | 0 | 8 | 0 | 0 |
| 479.192342 | 479.192271 | 16372448 | 24 | 32 | 0 | 10 | 0 | 0 |
| 479.207588 | 479.207527 | 9467616 | 28 | 32 | 0 | 7 | 0 | 0 |
| 479.228733 | 479.228656 | 15074017 | 25 | 36 | 0 | 9 | 0 | 0 |
| 479.243918 | 479.243912 | 8004321 | 29 | 36 | 0 | 6 | 0 | 0 |
| 479.26513 | 479.265042 | 6287074 | 26 | 40 | 0 | 8 | 0 | 0 |
| 479.280368 | 479.280298 | 2745570 | 30 | 40 | 0 | 5 | 0 | 0 |
| 479.301253 | 479.301427 | 1716962 | 27 | 44 | 0 | 7 | 0 | 0 |
| 481.041288 | 481.041249 | 1589512 | 23 | 14 | 0 | 12 | 0 | 0 |
| 481.077644 | 481.077635 | 2943496 | 24 | 18 | 0 | 11 | 0 | 0 |
| 481.092919 | 481.092891 | 1514121 | 28 | 18 | 0 | 8 | 0 | 0 |
| 481.114027 | 481.11402 | 4441353 | 25 | 22 | 0 | 10 | 0 | 0 |
| 481.117403 | 481.117391 | 2112521 | 22 | 26 | 0 | 10 | 1 | 0 |
| 481.135223 | 481.13515 | 3744522 | 22 | 26 | 0 | 12 | 0 | 0 |
| 481.15036 | 481.150406 | 6690058 | 26 | 26 | 0 | 9 | 0 | 0 |
| 481.153802 | 481.153777 | 2983946 | 23 | 30 | 0 | 9 | 1 | 0 |
| 481.171523 | 481.171535 | 8093450 | 23 | 30 | 0 | 11 | 0 | 0 |
| 481.186836 | 481.186791 | 11022090 | 27 | 30 | 0 | 8 | 0 | 0 |
| 481.190351 | 481.190162 | 2277643 | 24 | 34 | 0 | 8 | 1 | 0 |
| 481.208016 | 481.207921 | 9572107 | 24 | 34 | 0 | 10 | 0 | 0 |
| 481.223218 | 481.223177 | 13344523 | 28 | 34 | 0 | 7 | 0 | 0 |
| 481.244402 | 481.244306 | 6126860 | 25 | 38 | 0 | 9 | 0 | 0 |
| 481.259535 | 481.259562 | 10052364 | 29 | 38 | 0 | 6 | 0 | 0 |
| 481.280779 | 481.280692 | 1979660 | 26 | 42 | 0 | 8 | 0 | 0 |
| 481.296084 | 481.295948 | 3063565 | 30 | 42 | 0 | 5 | 0 | 0 |
| 483.05709 | 483.0569 | 1802408 | 23 | 16 | 0 | 12 | 0 | 0 |
| 483.072448 | 483.072156 | 1548584 | 27 | 16 | 0 | 9 | 0 | 0 |
| 483.09358 | 483.093285 | 3218217 | 24 | 20 | 0 | 11 | 0 | 0 |
| 483.096759 | 483.096656 | 1625129 | 21 | 24 | 0 | 11 | 1 | 0 |
| 483.108652 | 483.108541 | 1955369 | 28 | 20 | 0 | 8 | 0 | 0 |
| 483.129748 | 483.129671 | 5656361 | 25 | 24 | 0 | 10 | 0 | 0 |
| 483.133228 | 483.133041 | 3545897 | 22 | 28 | 0 | 10 | 1 | 0 |
| 483.150928 | 483.1508 | 2734889 | 22 | 28 | 0 | 12 | 0 | 0 |
| 483.16613 | 483.166056 | 10574634 | 26 | 28 | 0 | 9 | 0 | 0 |
| 483.169532 | 483.169427 | 3919658 | 23 | 32 | 0 | 9 | 1 | 0 |
| 483.181053 | 483.181312 | 1481770 | 30 | 28 | 0 | 6 | 0 | 0 |
| 483.187301 | 483.187185 | 4204842 | 23 | 32 | 0 | 11 | 0 | 0 |
| 483.202452 | 483.202442 | 16253738 | 27 | 32 | 0 | 8 | 0 | 0 |
| 483.206065 | 483.205812 | 1932586 | 24 | 36 | 0 | 8 | 1 | 0 |
| 483.223632 | 483.223571 | 3726634 | 24 | 36 | 0 | 10 | 0 | 0 |
| 483.238878 | 483.238827 | 17818410 | 28 | 36 | 0 | 7 | 0 | 0 |
| 483.259804 | 483.259956 | 2101803 | 25 | 40 | 0 | 9 | 0 | 0 |
| 483.27522 | 483.275213 | 11700011 | 29 | 40 | 0 | 6 | 0 | 0 |
| 483.311324 | 483.311598 | 2898988 | 30 | 44 | 0 | 5 | 0 | 0 |
| 484.197772 | 484.197691 | 1587509 | 26 | 31 | 1 | 8 | 0 | 0 |
| 485.072725 | 485.07255 | 2318909 | 23 | 18 | 0 | 12 | 0 | 0 |
| 485.087891 | 485.087806 | 2084669 | 27 | 18 | 0 | 9 | 0 | 0 |
| 485.109089 | 485.108935 | 3668797 | 24 | 22 | 0 | 11 | 0 | 0 |
| 485.112558 | 485.112306 | 2144829 | 21 | 26 | 0 | 11 | 1 | 0 |
| 485.124172 | 485.124191 | 2295357 | 28 | 22 | 0 | 8 | 0 | 0 |
| 485.145445 | 485.145321 | 7255358 | 25 | 26 | 0 | 10 | 0 | 0 |
| 485.148747 | 485.148691 | 4675902 | 22 | 30 | 0 | 10 | 1 | 0 |
| 485.160664 | 485.160577 | 2227006 | 29 | 26 | 0 | 7 | 0 | 0 |
| 485.166714 | 485.16645 | 1517374 | 22 | 30 | 0 | 12 | 0 | 0 |
| 485.181751 | 485.181706 | 14787390 | 26 | 30 | 0 | 9 | 0 | 0 |
| 485.185074 | 485.185077 | 2807614 | 23 | 34 | 0 | 9 | 1 | 0 |
| 485.197033 | 485.196962 | 1926206 | 30 | 30 | 0 | 6 | 0 | 0 |
| 485.218126 | 485.218092 | 20620094 | 27 | 34 | 0 | 8 | 0 | 0 |
| 485.233558 | 485.233348 | 1775678 | 31 | 34 | 0 | 5 | 0 | 0 |
| 485.254484 | 485.254477 | 19127102 | 28 | 38 | 0 | 7 | 0 | 0 |
| 485.290915 | 485.290863 | 11196222 | 29 | 42 | 0 | 6 | 0 | 0 |
| 485.32733 | 485.327248 | 2968895 | 30 | 46 | 0 | 5 | 0 | 0 |
| 487.066935 | 487.06707 | 1914309 | 26 | 16 | 0 | 10 | 0 | 0 |
| 487.088015 | 487.0882 | 2646342 | 23 | 20 | 0 | 12 | 0 | 0 |
| 487.103455 | 487.103456 | 2415558 | 27 | 20 | 0 | 9 | 0 | 0 |
| 487.124677 | 487.124585 | 5010758 | 24 | 24 | 0 | 11 | 0 | 0 |
| 487.127946 | 487.127956 | 2762566 | 21 | 28 | 0 | 11 | 1 | 0 |
| 487.139895 | 487.139841 | 2548038 | 28 | 24 | 0 | 8 | 0 | 0 |
| 487.161026 | 487.160971 | 12671813 | 25 | 28 | 0 | 10 | 0 | 0 |
| 487.16459 | 487.164341 | 3132742 | 22 | 32 | 0 | 10 | 1 | 0 |
| 487.1763 | 487.176227 | 2553926 | 29 | 28 | 0 | 7 | 0 | 0 |
| 487.197419 | 487.197356 | 22139718 | 26 | 32 | 0 | 9 | 0 | 0 |
| 487.212522 | 487.212612 | 2704454 | 30 | 32 | 0 | 6 | 0 | 0 |
| 487.233764 | 487.233742 | 24337222 | 27 | 36 | 0 | 8 | 0 | 0 |
| 487.248917 | 487.248998 | 1869894 | 31 | 36 | 0 | 5 | 0 | 0 |
| 487.270132 | 487.270127 | 17517382 | 28 | 40 | 0 | 7 | 0 | 0 |
| 487.306564 | 487.306513 | 8287046 | 29 | 44 | 0 | 6 | 0 | 0 |
| 487.342832 | 487.342898 | 1495878 | 30 | 48 | 0 | 5 | 0 | 0 |
| 489.046319 | 489.046335 | 1740098 | 25 | 14 | 0 | 11 | 0 | 0 |
| 489.082774 | 489.08272 | 3119426 | 26 | 18 | 0 | 10 | 0 | 0 |
| 489.103917 | 489.10385 | 2942786 | 23 | 22 | 0 | 12 | 0 | 0 |
| 489.119467 | 489.119106 | 2937666 | 27 | 22 | 0 | 9 | 0 | 0 |
| 489.140298 | 489.140235 | 7437122 | 24 | 26 | 0 | 11 | 0 | 0 |
| 489.143707 | 489.143606 | 2188097 | 21 | 30 | 0 | 11 | 1 | 0 |
| 489.155651 | 489.155491 | 2643009 | 28 | 26 | 0 | 8 | 0 | 0 |
| 489.176646 | 489.176621 | 18629442 | 25 | 30 | 0 | 10 | 0 | 0 |
| 489.191969 | 489.191877 | 3347009 | 29 | 30 | 0 | 7 | 0 | 0 |
| 489.213039 | 489.213006 | 24738626 | 26 | 34 | 0 | 9 | 0 | 0 |
| 489.228474 | 489.228262 | 3099457 | 30 | 34 | 0 | 6 | 0 | 0 |
| 489.249382 | 489.249392 | 21930816 | 27 | 38 | 0 | 8 | 0 | 0 |
| 489.264659 | 489.264648 | 2264641 | 31 | 38 | 0 | 5 | 0 | 0 |
| 489.285848 | 489.285777 | 11752257 | 28 | 42 | 0 | 7 | 0 | 0 |
| 489.322282 | 489.322163 | 3584321 | 29 | 46 | 0 | 6 | 0 | 0 |
| 491.061887 | 491.061985 | 2414258 | 25 | 16 | 0 | 11 | 0 | 0 |
| 491.083027 | 491.083114 | 1470130 | 22 | 20 | 0 | 13 | 0 | 0 |
| 491.098395 | 491.09837 | 3078962 | 26 | 20 | 0 | 10 | 0 | 0 |
| 491.119496 | 491.1195 | 3076145 | 23 | 24 | 0 | 12 | 0 | 0 |
| 491.134725 | 491.134756 | 3350833 | 27 | 24 | 0 | 9 | 0 | 0 |
| 491.155817 | 491.155885 | 10047281 | 24 | 28 | 0 | 11 | 0 | 0 |
| 491.171243 | 491.171141 | 4292401 | 28 | 28 | 0 | 8 | 0 | 0 |
| 491.192296 | 491.192271 | 19956528 | 25 | 32 | 0 | 10 | 0 | 0 |
| 491.207565 | 491.207527 | 5226800 | 29 | 32 | 0 | 7 | 0 | 0 |
| 491.228716 | 491.228656 | 21748528 | 26 | 36 | 0 | 9 | 0 | 0 |
| 491.243936 | 491.243912 | 3907888 | 30 | 36 | 0 | 6 | 0 | 0 |
| 491.26502 | 491.265042 | 13028144 | 27 | 40 | 0 | 8 | 0 | 0 |
| 491.280298 | 491.280298 | 1913391 | 31 | 40 | 0 | 5 | 0 | 0 |
| 491.301333 | 491.301427 | 4450095 | 28 | 44 | 0 | 7 | 0 | 0 |
| 493.041395 | 493.041249 | 1409046 | 24 | 14 | 0 | 12 | 0 | 0 |
| 493.077532 | 493.077635 | 3100950 | 25 | 18 | 0 | 11 | 0 | 0 |
| 493.113843 | 493.11402 | 3758357 | 26 | 22 | 0 | 10 | 0 | 0 |
| 493.135127 | 493.13515 | 4442389 | 23 | 26 | 0 | 12 | 0 | 0 |
| 493.150359 | 493.150406 | 4939541 | 27 | 26 | 0 | 9 | 0 | 0 |
| 493.15344 | 493.153777 | 1838613 | 24 | 30 | 0 | 9 | 1 | 0 |
| 493.171512 | 493.171535 | 10985236 | 24 | 30 | 0 | 11 | 0 | 0 |
| 493.186878 | 493.186791 | 6616852 | 28 | 30 | 0 | 8 | 0 | 0 |
| 493.207969 | 493.207921 | 16214804 | 25 | 34 | 0 | 10 | 0 | 0 |
| 493.223235 | 493.223177 | 8033044 | 29 | 34 | 0 | 7 | 0 | 0 |
| 493.244389 | 493.244306 | 11286291 | 26 | 38 | 0 | 9 | 0 | 0 |
| 493.259557 | 493.259562 | 4630291 | 30 | 38 | 0 | 6 | 0 | 0 |
| 493.296059 | 493.295948 | 1505554 | 31 | 42 | 0 | 5 | 0 | 0 |
| 495.056854 | 495.0569 | 2315247 | 24 | 16 | 0 | 12 | 0 | 0 |
| 495.072137 | 495.072156 | 1429231 | 28 | 16 | 0 | 9 | 0 | 0 |
| 495.0933 | 495.093285 | 3021038 | 25 | 20 | 0 | 11 | 0 | 0 |
| 495.114453 | 495.114414 | 1919214 | 22 | 24 | 0 | 13 | 0 | 0 |
| 495.129766 | 495.129671 | 3784686 | 26 | 24 | 0 | 10 | 0 | 0 |
| 495.132955 | 495.133041 | 2227949 | 23 | 28 | 0 | 10 | 1 | 0 |
| 495.150745 | 495.1508 | 4889325 | 23 | 28 | 0 | 12 | 0 | 0 |
| 495.16605 | 495.166056 | 6721261 | 27 | 28 | 0 | 9 | 0 | 0 |
| 495.169146 | 495.169427 | 2550765 | 24 | 32 | 0 | 9 | 1 | 0 |
| 495.187219 | 495.187185 | 8140524 | 24 | 32 | 0 | 11 | 0 | 0 |
| 495.202462 | 495.202442 | 10151660 | 28 | 32 | 0 | 8 | 0 | 0 |
| 495.223585 | 495.223571 | 8716011 | 25 | 36 | 0 | 10 | 0 | 0 |
| 495.238841 | 495.238827 | 9989867 | 29 | 36 | 0 | 7 | 0 | 0 |
| 495.259951 | 495.259956 | 4740843 | 26 | 40 | 0 | 9 | 0 | 0 |
| 495.275174 | 495.275213 | 5033706 | 30 | 40 | 0 | 6 | 0 | 0 |
| 497.051304 | 497.05142 | 1547453 | 27 | 14 | 0 | 10 | 0 | 0 |
| 497.072287 | 497.07255 | 2138557 | 24 | 18 | 0 | 12 | 0 | 0 |
| 497.088136 | 497.087806 | 2133180 | 28 | 18 | 0 | 9 | 0 | 0 |
| 497.109056 | 497.108935 | 3444924 | 25 | 22 | 0 | 11 | 0 | 0 |
| 497.11237 | 497.112306 | 1784508 | 22 | 26 | 0 | 11 | 1 | 0 |
| 497.124308 | 497.124191 | 1527483 | 29 | 22 | 0 | 8 | 0 | 0 |
| 497.130002 | 497.130064 | 1586619 | 22 | 26 | 0 | 13 | 0 | 0 |
| 497.145416 | 497.145321 | 5862587 | 26 | 26 | 0 | 10 | 0 | 0 |
| 497.148817 | 497.148691 | 3423931 | 23 | 30 | 0 | 10 | 1 | 0 |
| 497.166438 | 497.16645 | 3024570 | 23 | 30 | 0 | 12 | 0 | 0 |
| 497.1817 | 497.181706 | 10188474 | 27 | 30 | 0 | 9 | 0 | 0 |
| 497.18517 | 497.185077 | 3068090 | 24 | 34 | 0 | 9 | 1 | 0 |
| 497.202837 | 497.202835 | 3998905 | 24 | 34 | 0 | 11 | 0 | 0 |
| 497.218064 | 497.218092 | 13780665 | 28 | 34 | 0 | 8 | 0 | 0 |
| 497.239278 | 497.239221 | 3427000 | 25 | 38 | 0 | 10 | 0 | 0 |
| 497.254467 | 497.254477 | 13330104 | 29 | 38 | 0 | 7 | 0 | 0 |
| 497.275593 | 497.275606 | 1555383 | 26 | 42 | 0 | 9 | 0 | 0 |
| 497.290969 | 497.290863 | 5392311 | 30 | 42 | 0 | 6 | 0 | 0 |
| 499.067054 | 499.06707 | 1674625 | 27 | 16 | 0 | 10 | 0 | 0 |
| 499.088372 | 499.0882 | 2211968 | 24 | 20 | 0 | 12 | 0 | 0 |
| 499.103588 | 499.103456 | 2481280 | 28 | 20 | 0 | 9 | 0 | 0 |
| 499.124721 | 499.124585 | 3845759 | 25 | 24 | 0 | 11 | 0 | 0 |
| 499.127871 | 499.127956 | 3063423 | 22 | 28 | 0 | 11 | 1 | 0 |
| 499.139642 | 499.139841 | 1450366 | 29 | 24 | 0 | 8 | 0 | 0 |
| 499.160993 | 499.160971 | 8426110 | 26 | 28 | 0 | 10 | 0 | 0 |
| 499.164494 | 499.164341 | 3632766 | 23 | 32 | 0 | 10 | 1 | 0 |
| 499.176377 | 499.176227 | 1521533 | 30 | 28 | 0 | 7 | 0 | 0 |
| 499.181917 | 499.1821 | 1464445 | 23 | 32 | 0 | 12 | 0 | 0 |
| 499.197403 | 499.197356 | 15661692 | 27 | 32 | 0 | 9 | 0 | 0 |
| 499.200825 | 499.200727 | 2444669 | 24 | 36 | 0 | 9 | 1 | 0 |
| 499.212765 | 499.212612 | 1670396 | 31 | 32 | 0 | 6 | 0 | 0 |
| 499.218734 | 499.218486 | 1902204 | 24 | 36 | 0 | 11 | 0 | 0 |
| 499.233758 | 499.233742 | 18723452 | 28 | 36 | 0 | 8 | 0 | 0 |
| 499.255048 | 499.254871 | 1408635 | 25 | 40 | 0 | 10 | 0 | 0 |
| 499.270156 | 499.270127 | 14805626 | 29 | 40 | 0 | 7 | 0 | 0 |
| 499.306486 | 499.306513 | 6307449 | 30 | 44 | 0 | 6 | 0 | 0 |
| 501.046612 | 501.046335 | 1724732 | 26 | 14 | 0 | 11 | 0 | 0 |
| 501.067542 | 501.067464 | 1688123 | 23 | 18 | 0 | 13 | 0 | 0 |
| 501.082742 | 501.08272 | 2398779 | 27 | 18 | 0 | 10 | 0 | 0 |
| 501.10388 | 501.10385 | 2680378 | 24 | 22 | 0 | 12 | 0 | 0 |
| 501.119227 | 501.119106 | 2501177 | 28 | 22 | 0 | 9 | 0 | 0 |
| 501.140095 | 501.140235 | 5188153 | 25 | 26 | 0 | 11 | 0 | 0 |
| 501.143669 | 501.143606 | 2812728 | 22 | 30 | 0 | 11 | 1 | 0 |
| 501.155461 | 501.155491 | 1966904 | 29 | 26 | 0 | 8 | 0 | 0 |
| 501.176632 | 501.176621 | 12580407 | 26 | 30 | 0 | 10 | 0 | 0 |
| 501.180133 | 501.179992 | 2309431 | 23 | 34 | 0 | 10 | 1 | 0 |
| 501.191734 | 501.191877 | 1816758 | 30 | 30 | 0 | 7 | 0 | 0 |
| 501.213021 | 501.213006 | 21682742 | 27 | 34 | 0 | 9 | 0 | 0 |
| 501.228351 | 501.228262 | 1956405 | 31 | 34 | 0 | 6 | 0 | 0 |
| 501.249368 | 501.249392 | 22581812 | 28 | 38 | 0 | 8 | 0 | 0 |
| 501.264581 | 501.264648 | 1550644 | 32 | 38 | 0 | 5 | 0 | 0 |
| 501.285762 | 501.285777 | 15104563 | 29 | 42 | 0 | 7 | 0 | 0 |
| 501.32212 | 501.322163 | 4600882 | 30 | 46 | 0 | 6 | 0 | 0 |
| 503.062257 | 503.061985 | 1627884 | 26 | 16 | 0 | 11 | 0 | 0 |
| 503.098099 | 503.09837 | 2750955 | 27 | 20 | 0 | 10 | 0 | 0 |
| 503.119451 | 503.1195 | 2999274 | 24 | 24 | 0 | 12 | 0 | 0 |
| 503.122743 | 503.122871 | 1949674 | 21 | 28 | 0 | 12 | 1 | 0 |
| 503.134861 | 503.134756 | 2742249 | 28 | 24 | 0 | 9 | 0 | 0 |
| 503.155819 | 503.155885 | 9155560 | 25 | 28 | 0 | 11 | 0 | 0 |
| 503.159502 | 503.159256 | 2037736 | 22 | 32 | 0 | 11 | 1 | 0 |
| 503.171107 | 503.171141 | 3595240 | 29 | 28 | 0 | 8 | 0 | 0 |
| 503.192285 | 503.192271 | 18696678 | 26 | 32 | 0 | 10 | 0 | 0 |
| 503.207574 | 503.207527 | 2844006 | 30 | 32 | 0 | 7 | 0 | 0 |
| 503.228636 | 503.228656 | 23704038 | 27 | 36 | 0 | 9 | 0 | 0 |
| 503.244028 | 503.243912 | 2426341 | 31 | 36 | 0 | 6 | 0 | 0 |
| 503.265014 | 503.265042 | 17480164 | 28 | 40 | 0 | 8 | 0 | 0 |
| 503.301345 | 503.301427 | 9060834 | 29 | 44 | 0 | 7 | 0 | 0 |
| 503.337782 | 503.337813 | 2102881 | 30 | 48 | 0 | 6 | 0 | 0 |
| 505.077754 | 505.077635 | 2670996 | 26 | 18 | 0 | 11 | 0 | 0 |
| 505.09873 | 505.098764 | 1786003 | 23 | 22 | 0 | 13 | 0 | 0 |
| 505.114025 | 505.11402 | 3124627 | 27 | 22 | 0 | 10 | 0 | 0 |
| 505.135148 | 505.13515 | 4253586 | 24 | 26 | 0 | 12 | 0 | 0 |
| 505.150488 | 505.150406 | 3194257 | 28 | 26 | 0 | 9 | 0 | 0 |
| 505.171527 | 505.171535 | 11883920 | 25 | 30 | 0 | 11 | 0 | 0 |
| 505.186763 | 505.186791 | 4522896 | 29 | 30 | 0 | 8 | 0 | 0 |
| 505.207883 | 505.207921 | 19194254 | 26 | 34 | 0 | 10 | 0 | 0 |
| 505.223217 | 505.223177 | 4031630 | 30 | 34 | 0 | 7 | 0 | 0 |
| 505.244253 | 505.244306 | 18043276 | 27 | 38 | 0 | 9 | 0 | 0 |
| 505.259546 | 505.259562 | 2525324 | 31 | 38 | 0 | 6 | 0 | 0 |
| 505.28073 | 505.280692 | 10355083 | 28 | 42 | 0 | 8 | 0 | 0 |
| 505.296131 | 505.295948 | 1421194 | 32 | 42 | 0 | 5 | 0 | 0 |
| 505.317086 | 505.317077 | 4145033 | 29 | 46 | 0 | 7 | 0 | 0 |
| 507.057121 | 507.0569 | 2052406 | 25 | 16 | 0 | 12 | 0 | 0 |
| 507.093365 | 507.093285 | 2882356 | 26 | 20 | 0 | 11 | 0 | 0 |
| 507.114454 | 507.114414 | 1881651 | 23 | 24 | 0 | 13 | 0 | 0 |
| 507.12982 | 507.129671 | 3312434 | 27 | 24 | 0 | 10 | 0 | 0 |
| 507.133258 | 507.133041 | 1421746 | 24 | 28 | 0 | 10 | 1 | 0 |
| 507.150844 | 507.1508 | 5543730 | 24 | 28 | 0 | 12 | 0 | 0 |
| 507.165973 | 507.166056 | 5075249 | 28 | 28 | 0 | 9 | 0 | 0 |
| 507.169187 | 507.169427 | 1602352 | 25 | 32 | 0 | 9 | 1 | 0 |
| 507.187188 | 507.187185 | 11470127 | 25 | 32 | 0 | 11 | 0 | 0 |
| 507.202556 | 507.202442 | 5205807 | 29 | 32 | 0 | 8 | 0 | 0 |
| 507.223598 | 507.223571 | 13330734 | 26 | 36 | 0 | 10 | 0 | 0 |
| 507.238847 | 507.238827 | 4566829 | 30 | 36 | 0 | 7 | 0 | 0 |
| 507.259925 | 507.259956 | 9965868 | 27 | 40 | 0 | 9 | 0 | 0 |
| 507.275289 | 507.275213 | 3003691 | 31 | 40 | 0 | 6 | 0 | 0 |
| 507.296322 | 507.296342 | 4202538 | 28 | 44 | 0 | 8 | 0 | 0 |
| 509.072807 | 509.07255 | 2208463 | 25 | 18 | 0 | 12 | 0 | 0 |
| 509.087847 | 509.087806 | 1405902 | 29 | 18 | 0 | 9 | 0 | 0 |
| 509.109039 | 509.108935 | 3154637 | 26 | 22 | 0 | 11 | 0 | 0 |
| 509.112095 | 509.112306 | 1760972 | 23 | 26 | 0 | 11 | 1 | 0 |
| 509.130162 | 509.130064 | 1963211 | 23 | 26 | 0 | 13 | 0 | 0 |
| 509.145219 | 509.145321 | 4324043 | 27 | 26 | 0 | 10 | 0 | 0 |
| 509.148947 | 509.148691 | 2486987 | 24 | 30 | 0 | 10 | 1 | 0 |
| 509.166565 | 509.16645 | 4832970 | 24 | 30 | 0 | 12 | 0 | 0 |
| 509.181631 | 509.181706 | 6304457 | 28 | 30 | 0 | 9 | 0 | 0 |
| 509.184942 | 509.185077 | 2143177 | 25 | 34 | 0 | 9 | 1 | 0 |
| 509.202841 | 509.202835 | 7544008 | 25 | 34 | 0 | 11 | 0 | 0 |
| 509.217963 | 509.218092 | 7741127 | 29 | 34 | 0 | 8 | 0 | 0 |
| 509.239195 | 509.239221 | 6726854 | 26 | 38 | 0 | 10 | 0 | 0 |
| 509.254368 | 509.254477 | 5409989 | 30 | 38 | 0 | 7 | 0 | 0 |
| 509.275699 | 509.275606 | 3914948 | 27 | 42 | 0 | 9 | 0 | 0 |
| 509.290756 | 509.290863 | 2475971 | 31 | 42 | 0 | 6 | 0 | 0 |
| 511.067154 | 511.06707 | 1375841 | 28 | 16 | 0 | 10 | 0 | 0 |
| 511.088283 | 511.0882 | 2047072 | 25 | 20 | 0 | 12 | 0 | 0 |
| 511.124623 | 511.124585 | 3257950 | 26 | 24 | 0 | 11 | 0 | 0 |
| 511.127786 | 511.127956 | 1968990 | 23 | 28 | 0 | 11 | 1 | 0 |
| 511.139879 | 511.139841 | 1684573 | 30 | 24 | 0 | 8 | 0 | 0 |
| 511.145653 | 511.145715 | 1597789 | 23 | 28 | 0 | 13 | 0 | 0 |
| 511.161004 | 511.160971 | 5864028 | 27 | 28 | 0 | 10 | 0 | 0 |
| 511.164313 | 511.164341 | 2958428 | 24 | 32 | 0 | 10 | 1 | 0 |
| 511.182188 | 511.1821 | 3366491 | 24 | 32 | 0 | 12 | 0 | 0 |
| 511.197313 | 511.197356 | 9683034 | 28 | 32 | 0 | 9 | 0 | 0 |
| 511.218649 | 511.218486 | 3712601 | 25 | 36 | 0 | 11 | 0 | 0 |
| 511.233656 | 511.233742 | 11284568 | 29 | 36 | 0 | 8 | 0 | 0 |
| 511.254935 | 511.254871 | 2437463 | 26 | 40 | 0 | 10 | 0 | 0 |
| 511.270136 | 511.270127 | 7597142 | 30 | 40 | 0 | 7 | 0 | 0 |
| 511.290797 | 511.291257 | 1537109 | 27 | 44 | 0 | 9 | 0 | 0 |
| 511.306369 | 511.306513 | 1724244 | 31 | 44 | 0 | 6 | 0 | 0 |
| 513.067531 | 513.067464 | 1869805 | 24 | 18 | 0 | 13 | 0 | 0 |
| 513.103759 | 513.10385 | 2498283 | 25 | 22 | 0 | 12 | 0 | 0 |
| 513.119187 | 513.119106 | 1422570 | 29 | 22 | 0 | 9 | 0 | 0 |
| 513.14019 | 513.140235 | 4638953 | 26 | 26 | 0 | 11 | 0 | 0 |
| 513.143611 | 513.143606 | 2609129 | 23 | 30 | 0 | 11 | 1 | 0 |
| 513.155511 | 513.155491 | 1523944 | 30 | 26 | 0 | 8 | 0 | 0 |
| 513.176594 | 513.176621 | 8399847 | 27 | 30 | 0 | 10 | 0 | 0 |
| 513.180097 | 513.179992 | 2967015 | 24 | 34 | 0 | 10 | 1 | 0 |
| 513.191782 | 513.191877 | 1775078 | 31 | 30 | 0 | 7 | 0 | 0 |
| 513.197971 | 513.19775 | 1432550 | 24 | 34 | 0 | 12 | 0 | 0 |
| 513.21304 | 513.213006 | 14307301 | 28 | 34 | 0 | 9 | 0 | 0 |
| 513.234261 | 513.234136 | 2020324 | 25 | 38 | 0 | 11 | 0 | 0 |
| 513.249371 | 513.249392 | 14523363 | 29 | 38 | 0 | 8 | 0 | 0 |
| 513.285734 | 513.285777 | 8621536 | 30 | 42 | 0 | 7 | 0 | 0 |
| 513.322125 | 513.322163 | 1386718 | 31 | 46 | 0 | 6 | 0 | 0 |
| 515.061318 | 515.061333 | 1655412 | 19 | 20 | 2 | 13 | 1 | 0 |
| 515.082842 | 515.083114 | 1669555 | 24 | 20 | 0 | 13 | 0 | 0 |
| 515.098509 | 515.09837 | 1777266 | 28 | 20 | 0 | 10 | 0 | 0 |
| 515.119293 | 515.1195 | 2927473 | 25 | 24 | 0 | 12 | 0 | 0 |
| 515.134718 | 515.134756 | 1856112 | 29 | 24 | 0 | 9 | 0 | 0 |
| 515.155942 | 515.155885 | 6253423 | 26 | 28 | 0 | 11 | 0 | 0 |
| 515.159261 | 515.159256 | 2091758 | 23 | 32 | 0 | 11 | 1 | 0 |
| 515.171305 | 515.171141 | 2100078 | 30 | 28 | 0 | 8 | 0 | 0 |
| 515.192212 | 515.192271 | 13395820 | 27 | 32 | 0 | 10 | 0 | 0 |
| 515.207484 | 515.207527 | 1762027 | 31 | 32 | 0 | 7 | 0 | 0 |
| 515.228617 | 515.228656 | 18603882 | 28 | 36 | 0 | 9 | 0 | 0 |
| 515.244171 | 515.243912 | 1763689 | 32 | 36 | 0 | 6 | 0 | 0 |
| 515.265105 | 515.265042 | 18007912 | 29 | 40 | 0 | 8 | 0 | 0 |
| 515.301395 | 515.301427 | 9329509 | 30 | 44 | 0 | 7 | 0 | 0 |
| 517.077548 | 517.077635 | 2334453 | 27 | 18 | 0 | 11 | 0 | 0 |
| 517.09889 | 517.098764 | 1673715 | 24 | 22 | 0 | 13 | 0 | 0 |
| 517.113988 | 517.11402 | 1884914 | 28 | 22 | 0 | 10 | 0 | 0 |
| 517.135206 | 517.13515 | 3674353 | 25 | 26 | 0 | 12 | 0 | 0 |
| 517.150419 | 517.150406 | 2652400 | 29 | 26 | 0 | 9 | 0 | 0 |
| 517.171544 | 517.171535 | 10182383 | 26 | 30 | 0 | 11 | 0 | 0 |
| 517.18674 | 517.186791 | 2473454 | 30 | 30 | 0 | 8 | 0 | 0 |
| 517.207873 | 517.207921 | 18558700 | 27 | 34 | 0 | 10 | 0 | 0 |
| 517.223114 | 517.223177 | 2712811 | 31 | 34 | 0 | 7 | 0 | 0 |
| 517.24427 | 517.244306 | 19850986 | 28 | 38 | 0 | 9 | 0 | 0 |
| 517.259226 | 517.259562 | 1512937 | 32 | 38 | 0 | 6 | 0 | 0 |
| 517.280657 | 517.280692 | 14235368 | 29 | 42 | 0 | 8 | 0 | 0 |
| 517.317136 | 517.317077 | 6442725 | 30 | 46 | 0 | 7 | 0 | 0 |
| 519.056999 | 519.0569 | 1784947 | 26 | 16 | 0 | 12 | 0 | 0 |
| 519.093302 | 519.093285 | 1880817 | 27 | 20 | 0 | 11 | 0 | 0 |
| 519.114502 | 519.114414 | 1915759 | 24 | 24 | 0 | 13 | 0 | 0 |
| 519.129752 | 519.129671 | 2640494 | 28 | 24 | 0 | 10 | 0 | 0 |
| 519.150828 | 519.1508 | 5190253 | 25 | 28 | 0 | 12 | 0 | 0 |
| 519.166074 | 519.166056 | 2768236 | 29 | 28 | 0 | 9 | 0 | 0 |
| 519.187115 | 519.187185 | 11699818 | 26 | 32 | 0 | 11 | 0 | 0 |
| 519.202526 | 519.202442 | 3211881 | 30 | 32 | 0 | 8 | 0 | 0 |
| 519.223513 | 519.223571 | 16934504 | 27 | 36 | 0 | 10 | 0 | 0 |
| 519.238832 | 519.238827 | 2895463 | 31 | 36 | 0 | 7 | 0 | 0 |
| 519.25997 | 519.259956 | 15640165 | 28 | 40 | 0 | 9 | 0 | 0 |
| 519.275311 | 519.275213 | 1733476 | 32 | 40 | 0 | 6 | 0 | 0 |
| 519.296337 | 519.296342 | 8881763 | 29 | 44 | 0 | 8 | 0 | 0 |
| 519.332671 | 519.332727 | 2964065 | 30 | 48 | 0 | 7 | 0 | 0 |
| 521.072315 | 521.07255 | 2042859 | 26 | 18 | 0 | 12 | 0 | 0 |
| 521.108921 | 521.108935 | 2466536 | 27 | 22 | 0 | 11 | 0 | 0 |
| 521.129883 | 521.130064 | 2271719 | 24 | 26 | 0 | 13 | 0 | 0 |
| 521.145236 | 521.145321 | 3453670 | 28 | 26 | 0 | 10 | 0 | 0 |
| 521.166487 | 521.16645 | 5779045 | 25 | 30 | 0 | 12 | 0 | 0 |
| 521.181669 | 521.181706 | 3734499 | 29 | 30 | 0 | 9 | 0 | 0 |
| 521.202801 | 521.202835 | 10969570 | 26 | 34 | 0 | 11 | 0 | 0 |
| 521.217988 | 521.218092 | 4185057 | 30 | 34 | 0 | 8 | 0 | 0 |
| 521.239232 | 521.239221 | 12068319 | 27 | 38 | 0 | 10 | 0 | 0 |
| 521.254459 | 521.254477 | 3415774 | 31 | 38 | 0 | 7 | 0 | 0 |
| 521.275538 | 521.275606 | 7002077 | 28 | 42 | 0 | 9 | 0 | 0 |
| 521.29082 | 521.290863 | 1542364 | 32 | 42 | 0 | 6 | 0 | 0 |
| 521.311908 | 521.311992 | 3177690 | 29 | 46 | 0 | 8 | 0 | 0 |
| 523.088175 | 523.0882 | 2048863 | 26 | 20 | 0 | 12 | 0 | 0 |
| 523.124395 | 523.124585 | 2685917 | 27 | 24 | 0 | 11 | 0 | 0 |
| 523.145521 | 523.145715 | 2254427 | 24 | 28 | 0 | 13 | 0 | 0 |
| 523.161028 | 523.160971 | 3898714 | 28 | 28 | 0 | 10 | 0 | 0 |
| 523.182109 | 523.1821 | 4978521 | 25 | 32 | 0 | 12 | 0 | 0 |
| 523.197426 | 523.197356 | 5187928 | 29 | 32 | 0 | 9 | 0 | 0 |
| 523.200607 | 523.200727 | 1877591 | 26 | 36 | 0 | 9 | 1 | 0 |
| 523.218472 | 523.218486 | 7518550 | 26 | 36 | 0 | 11 | 0 | 0 |
| 523.233747 | 523.233742 | 5477717 | 30 | 36 | 0 | 8 | 0 | 0 |
| 523.254957 | 523.254871 | 5316948 | 27 | 40 | 0 | 10 | 0 | 0 |
| 523.269991 | 523.270127 | 3266130 | 31 | 40 | 0 | 7 | 0 | 0 |
| 523.291028 | 523.291257 | 3119441 | 28 | 44 | 0 | 9 | 0 | 0 |
| 523.306679 | 523.306513 | 1763664 | 32 | 44 | 0 | 6 | 0 | 0 |
| 525.067417 | 525.067464 | 1619027 | 25 | 18 | 0 | 13 | 0 | 0 |
| 525.103825 | 525.10385 | 2557648 | 26 | 22 | 0 | 12 | 0 | 0 |
| 525.140146 | 525.140235 | 3397838 | 27 | 26 | 0 | 11 | 0 | 0 |
| 525.143871 | 525.143606 | 2295502 | 24 | 30 | 0 | 11 | 1 | 0 |
| 525.176619 | 525.176621 | 5161931 | 28 | 30 | 0 | 10 | 0 | 0 |
| 525.180001 | 525.179992 | 2125259 | 25 | 34 | 0 | 10 | 1 | 0 |
| 525.197887 | 525.19775 | 2760906 | 25 | 34 | 0 | 12 | 0 | 0 |
| 525.212984 | 525.213006 | 8530121 | 29 | 34 | 0 | 9 | 0 | 0 |
| 525.233994 | 525.234136 | 3901127 | 26 | 38 | 0 | 11 | 0 | 0 |
| 525.249474 | 525.249392 | 7129286 | 30 | 38 | 0 | 8 | 0 | 0 |
| 525.270308 | 525.270521 | 2653893 | 27 | 42 | 0 | 10 | 0 | 0 |
| 525.285717 | 525.285777 | 3143619 | 31 | 42 | 0 | 7 | 0 | 0 |
| 527.083117 | 527.083114 | 1460034 | 25 | 20 | 0 | 13 | 0 | 0 |
| 527.098409 | 527.09837 | 1703745 | 29 | 20 | 0 | 10 | 0 | 0 |
| 527.119554 | 527.1195 | 2781248 | 26 | 24 | 0 | 12 | 0 | 0 |
| 527.155834 | 527.155885 | 4295485 | 27 | 28 | 0 | 11 | 0 | 0 |
| 527.158989 | 527.159256 | 2496957 | 24 | 32 | 0 | 11 | 1 | 0 |
| 527.192228 | 527.192271 | 7787578 | 28 | 32 | 0 | 10 | 0 | 0 |
| 527.195519 | 527.195642 | 2112442 | 25 | 36 | 0 | 10 | 1 | 0 |
| 527.213762 | 527.2134 | 1859129 | 25 | 36 | 0 | 12 | 0 | 0 |
| 527.228585 | 527.228656 | 11495480 | 29 | 36 | 0 | 9 | 0 | 0 |
| 527.249876 | 527.249786 | 1480502 | 26 | 40 | 0 | 11 | 0 | 0 |
| 527.265025 | 527.265042 | 9993269 | 30 | 40 | 0 | 8 | 0 | 0 |
| 527.301456 | 527.301427 | 2460722 | 31 | 44 | 0 | 7 | 0 | 0 |
| 529.077752 | 529.077635 | 1485745 | 28 | 18 | 0 | 11 | 0 | 0 |
| 529.098884 | 529.098764 | 1954479 | 25 | 22 | 0 | 13 | 0 | 0 |
| 529.114296 | 529.11402 | 1788078 | 29 | 22 | 0 | 10 | 0 | 0 |
| 529.135106 | 529.13515 | 2938797 | 26 | 26 | 0 | 12 | 0 | 0 |
| 529.138546 | 529.138521 | 1614765 | 23 | 30 | 0 | 12 | 1 | 0 |
| 529.150521 | 529.150406 | 1827244 | 30 | 26 | 0 | 9 | 0 | 0 |
| 529.171551 | 529.171535 | 6767530 | 27 | 30 | 0 | 11 | 0 | 0 |
| 529.175103 | 529.174906 | 2040746 | 24 | 34 | 0 | 11 | 1 | 0 |
| 529.186603 | 529.186791 | 1479593 | 31 | 30 | 0 | 8 | 0 | 0 |
| 529.207883 | 529.207921 | 11807655 | 28 | 34 | 0 | 10 | 0 | 0 |
| 529.223294 | 529.223177 | 2015398 | 32 | 34 | 0 | 7 | 0 | 0 |
| 529.244299 | 529.244306 | 15057829 | 29 | 38 | 0 | 9 | 0 | 0 |
| 529.280704 | 529.280692 | 11330466 | 30 | 42 | 0 | 8 | 0 | 0 |
| 529.316719 | 529.317077 | 1808799 | 31 | 46 | 0 | 7 | 0 | 0 |
| 531.09301 | 531.093285 | 1978397 | 28 | 20 | 0 | 11 | 0 | 0 |
| 531.114479 | 531.114414 | 1613340 | 25 | 24 | 0 | 13 | 0 | 0 |
| 531.12974 | 531.129671 | 2084122 | 29 | 24 | 0 | 10 | 0 | 0 |
| 531.150718 | 531.1508 | 4010777 | 26 | 28 | 0 | 12 | 0 | 0 |
| 531.154485 | 531.154171 | 1620761 | 23 | 32 | 0 | 12 | 1 | 0 |
| 531.165866 | 531.166056 | 2361112 | 30 | 28 | 0 | 9 | 0 | 0 |
| 531.187226 | 531.187185 | 9822998 | 27 | 32 | 0 | 11 | 0 | 0 |
| 531.202305 | 531.202442 | 2282773 | 31 | 32 | 0 | 8 | 0 | 0 |
| 531.223565 | 531.223571 | 15690515 | 28 | 36 | 0 | 10 | 0 | 0 |
| 531.239045 | 531.238827 | 2231570 | 32 | 36 | 0 | 7 | 0 | 0 |
| 531.259865 | 531.259956 | 15524625 | 29 | 40 | 0 | 9 | 0 | 0 |
| 531.296269 | 531.296342 | 10483470 | 30 | 44 | 0 | 8 | 0 | 0 |
| 533.108591 | 533.108935 | 1634185 | 28 | 22 | 0 | 11 | 0 | 0 |
| 533.129938 | 533.130064 | 1672775 | 25 | 26 | 0 | 13 | 0 | 0 |
| 533.145107 | 533.145321 | 2354310 | 29 | 26 | 0 | 10 | 0 | 0 |
| 533.166397 | 533.16645 | 5798021 | 26 | 30 | 0 | 12 | 0 | 0 |
| 533.181435 | 533.181706 | 2174723 | 30 | 30 | 0 | 9 | 0 | 0 |
| 533.202726 | 533.202835 | 11970178 | 27 | 34 | 0 | 11 | 0 | 0 |
| 533.217992 | 533.218092 | 2505345 | 31 | 34 | 0 | 8 | 0 | 0 |
| 533.239175 | 533.239221 | 14895743 | 28 | 38 | 0 | 10 | 0 | 0 |
| 533.254418 | 533.254477 | 2360446 | 32 | 38 | 0 | 7 | 0 | 0 |
| 533.275511 | 533.275606 | 11514492 | 29 | 42 | 0 | 9 | 0 | 0 |
| 533.31207 | 533.311992 | 6682234 | 30 | 46 | 0 | 8 | 0 | 0 |
| 535.088273 | 535.0882 | 2161655 | 27 | 20 | 0 | 12 | 0 | 0 |
| 535.124701 | 535.124585 | 2387444 | 28 | 24 | 0 | 11 | 0 | 0 |
| 535.145667 | 535.145715 | 3032563 | 25 | 28 | 0 | 13 | 0 | 0 |
| 535.161245 | 535.160971 | 2599921 | 29 | 28 | 0 | 10 | 0 | 0 |
| 535.1645 | 535.164341 | 1472753 | 26 | 32 | 0 | 10 | 1 | 0 |
| 535.182118 | 535.1821 | 6995440 | 26 | 32 | 0 | 12 | 0 | 0 |
| 535.197399 | 535.197356 | 3952623 | 30 | 32 | 0 | 9 | 0 | 0 |
| 535.200871 | 535.200727 | 1695214 | 27 | 36 | 0 | 9 | 1 | 0 |
| 535.218464 | 535.218486 | 10885613 | 27 | 36 | 0 | 11 | 0 | 0 |
| 535.233947 | 535.233742 | 2785260 | 31 | 36 | 0 | 8 | 0 | 0 |
| 535.254925 | 535.254871 | 8978410 | 28 | 40 | 0 | 10 | 0 | 0 |
| 535.270216 | 535.270127 | 2708457 | 32 | 40 | 0 | 7 | 0 | 0 |
| 535.29119 | 535.291257 | 6701544 | 29 | 44 | 0 | 9 | 0 | 0 |
| 535.327794 | 535.327642 | 2208741 | 30 | 48 | 0 | 8 | 0 | 0 |
| 537.067499 | 537.067464 | 1760613 | 26 | 18 | 0 | 13 | 0 | 0 |
| 537.103949 | 537.10385 | 2278755 | 27 | 22 | 0 | 12 | 0 | 0 |
| 537.140335 | 537.140235 | 2204000 | 28 | 26 | 0 | 11 | 0 | 0 |
| 537.161391 | 537.161365 | 2461023 | 25 | 30 | 0 | 13 | 0 | 0 |
| 537.176663 | 537.176621 | 4224350 | 29 | 30 | 0 | 10 | 0 | 0 |
| 537.180328 | 537.179992 | 1543005 | 26 | 34 | 0 | 10 | 1 | 0 |
| 537.197798 | 537.19775 | 5050204 | 26 | 34 | 0 | 12 | 0 | 0 |
| 537.213096 | 537.213006 | 3888475 | 30 | 34 | 0 | 9 | 0 | 0 |
| 537.234027 | 537.234136 | 6108505 | 27 | 38 | 0 | 11 | 0 | 0 |
| 537.249098 | 537.249392 | 3349336 | 31 | 38 | 0 | 8 | 0 | 0 |
| 537.270489 | 537.270521 | 4801879 | 28 | 42 | 0 | 10 | 0 | 0 |
| 537.285731 | 537.285777 | 2537302 | 32 | 42 | 0 | 7 | 0 | 0 |
| 537.306854 | 537.306907 | 2974036 | 29 | 46 | 0 | 9 | 0 | 0 |
| 539.08302 | 539.083114 | 1484498 | 26 | 20 | 0 | 13 | 0 | 0 |
| 539.119446 | 539.1195 | 2656464 | 27 | 24 | 0 | 12 | 0 | 0 |
| 539.155906 | 539.155885 | 3349197 | 28 | 28 | 0 | 11 | 0 | 0 |
| 539.15919 | 539.159256 | 2025677 | 25 | 32 | 0 | 11 | 1 | 0 |
| 539.177122 | 539.177015 | 2213836 | 25 | 32 | 0 | 13 | 0 | 0 |
| 539.192126 | 539.192271 | 4466379 | 29 | 32 | 0 | 10 | 0 | 0 |
| 539.195262 | 539.195642 | 2300618 | 26 | 36 | 0 | 10 | 1 | 0 |
| 539.213332 | 539.2134 | 3032265 | 26 | 36 | 0 | 12 | 0 | 0 |
| 539.228347 | 539.228656 | 5719240 | 30 | 36 | 0 | 9 | 0 | 0 |
| 539.249743 | 539.249786 | 2773574 | 27 | 40 | 0 | 11 | 0 | 0 |
| 539.265141 | 539.265042 | 4112581 | 31 | 40 | 0 | 8 | 0 | 0 |
| 539.286046 | 539.286171 | 1941188 | 28 | 44 | 0 | 10 | 0 | 0 |
| 539.301737 | 539.301427 | 1821891 | 32 | 44 | 0 | 7 | 0 | 0 |
| 541.09857 | 541.098764 | 1737281 | 26 | 22 | 0 | 13 | 0 | 0 |
| 541.114027 | 541.11402 | 1716288 | 30 | 22 | 0 | 10 | 0 | 0 |
| 541.135101 | 541.13515 | 2508863 | 27 | 26 | 0 | 12 | 0 | 0 |
| 541.150313 | 541.150406 | 1625918 | 31 | 26 | 0 | 9 | 0 | 0 |
| 541.171495 | 541.171535 | 4214332 | 28 | 30 | 0 | 11 | 0 | 0 |
| 541.174667 | 541.174906 | 2062492 | 25 | 34 | 0 | 11 | 1 | 0 |
| 541.207733 | 541.207921 | 6637626 | 29 | 34 | 0 | 10 | 0 | 0 |
| 541.228934 | 541.22905 | 1521464 | 26 | 38 | 0 | 12 | 0 | 0 |
| 541.244349 | 541.244306 | 7541815 | 30 | 38 | 0 | 9 | 0 | 0 |
| 541.28066 | 541.280692 | 4301876 | 31 | 42 | 0 | 8 | 0 | 0 |
| 541.317023 | 541.317077 | 1746482 | 32 | 46 | 0 | 7 | 0 | 0 |
| 543.041145 | 543.041643 | 1350327 | 24 | 16 | 0 | 15 | 0 | 0 |
| 543.114329 | 543.114414 | 1540530 | 26 | 24 | 0 | 13 | 0 | 0 |
| 543.150479 | 543.1508 | 3243952 | 27 | 28 | 0 | 12 | 0 | 0 |
| 543.165512 | 543.165404 | 2109359 | 23 | 32 | 2 | 11 | 1 | 0 |
| 543.187029 | 543.187185 | 6465453 | 28 | 32 | 0 | 11 | 0 | 0 |
| 543.190511 | 543.190556 | 2041517 | 25 | 36 | 0 | 11 | 1 | 0 |
| 543.223528 | 543.223571 | 9975722 | 29 | 36 | 0 | 10 | 0 | 0 |
| 543.238778 | 543.238827 | 1939625 | 33 | 36 | 0 | 7 | 0 | 0 |
| 543.259934 | 543.259956 | 10189736 | 30 | 40 | 0 | 9 | 0 | 0 |
| 543.296364 | 543.296342 | 3249573 | 31 | 44 | 0 | 8 | 0 | 0 |
| 545.072174 | 545.07255 | 1598250 | 28 | 18 | 0 | 12 | 0 | 0 |
| 545.109066 | 545.108935 | 1665831 | 29 | 22 | 0 | 11 | 0 | 0 |
| 545.130188 | 545.130064 | 1980710 | 26 | 26 | 0 | 13 | 0 | 0 |
| 545.145298 | 545.145321 | 1749797 | 30 | 26 | 0 | 10 | 0 | 0 |
| 545.166377 | 545.16645 | 4367140 | 27 | 30 | 0 | 12 | 0 | 0 |
| 545.181739 | 545.181706 | 1888546 | 31 | 30 | 0 | 9 | 0 | 0 |
| 545.202855 | 545.202835 | 8914721 | 28 | 34 | 0 | 11 | 0 | 0 |
| 545.218224 | 545.218092 | 2115616 | 32 | 34 | 0 | 8 | 0 | 0 |
| 545.239226 | 545.239221 | 12209950 | 29 | 38 | 0 | 10 | 0 | 0 |
| 545.254443 | 545.254477 | 1951773 | 33 | 38 | 0 | 7 | 0 | 0 |
| 545.275563 | 545.275606 | 10713884 | 30 | 42 | 0 | 9 | 0 | 0 |
| 545.31216 | 545.311992 | 2292506 | 31 | 46 | 0 | 8 | 0 | 0 |
| 547.088114 | 547.0882 | 1518113 | 28 | 20 | 0 | 12 | 0 | 0 |
| 547.124495 | 547.124585 | 2060959 | 29 | 24 | 0 | 11 | 0 | 0 |
| 547.145655 | 547.145715 | 2589341 | 26 | 28 | 0 | 13 | 0 | 0 |
| 547.160957 | 547.160971 | 1840028 | 30 | 28 | 0 | 10 | 0 | 0 |
| 547.182057 | 547.1821 | 5967515 | 27 | 32 | 0 | 12 | 0 | 0 |
| 547.197447 | 547.197356 | 2197146 | 31 | 32 | 0 | 9 | 0 | 0 |
| 547.218402 | 547.218486 | 10337944 | 28 | 36 | 0 | 11 | 0 | 0 |
| 547.233793 | 547.233742 | 2634903 | 32 | 36 | 0 | 8 | 0 | 0 |
| 547.254773 | 547.254871 | 11143830 | 29 | 40 | 0 | 10 | 0 | 0 |
| 547.269853 | 547.270127 | 1390869 | 33 | 40 | 0 | 7 | 0 | 0 |
| 547.291292 | 547.291257 | 8256148 | 30 | 44 | 0 | 9 | 0 | 0 |
| 549.104028 | 549.10385 | 1684380 | 28 | 22 | 0 | 12 | 0 | 0 |
| 549.1404 | 549.140235 | 2273818 | 29 | 26 | 0 | 11 | 0 | 0 |
| 549.161438 | 549.161365 | 3018264 | 26 | 30 | 0 | 13 | 0 | 0 |
| 549.176571 | 549.176621 | 2550807 | 30 | 30 | 0 | 10 | 0 | 0 |
| 549.197766 | 549.19775 | 6608406 | 27 | 34 | 0 | 12 | 0 | 0 |
| 549.212748 | 549.213006 | 2963989 | 31 | 34 | 0 | 9 | 0 | 0 |
| 549.234027 | 549.234136 | 8922644 | 28 | 38 | 0 | 11 | 0 | 0 |
| 549.249204 | 549.249392 | 2390035 | 32 | 38 | 0 | 8 | 0 | 0 |
| 549.270503 | 549.270521 | 7033874 | 29 | 42 | 0 | 10 | 0 | 0 |
| 549.286028 | 549.285777 | 1560592 | 33 | 42 | 0 | 7 | 0 | 0 |
| 549.306964 | 549.306907 | 4513295 | 30 | 46 | 0 | 9 | 0 | 0 |
| 551.083011 | 551.083114 | 1901470 | 27 | 20 | 0 | 13 | 0 | 0 |
| 551.119532 | 551.1195 | 1861276 | 28 | 24 | 0 | 12 | 0 | 0 |
| 551.155896 | 551.155885 | 2667674 | 29 | 28 | 0 | 11 | 0 | 0 |
| 551.176974 | 551.177015 | 2479640 | 26 | 32 | 0 | 13 | 0 | 0 |
| 551.192266 | 551.192271 | 3102615 | 30 | 32 | 0 | 10 | 0 | 0 |
| 551.21338 | 551.2134 | 4988310 | 27 | 36 | 0 | 12 | 0 | 0 |
| 551.228324 | 551.228656 | 3451285 | 31 | 36 | 0 | 9 | 0 | 0 |
| 551.232166 | 551.232027 | 1607573 | 28 | 40 | 0 | 9 | 1 | 0 |
| 551.24972 | 551.249786 | 5329812 | 28 | 40 | 0 | 11 | 0 | 0 |
| 551.265188 | 551.265042 | 3070355 | 32 | 40 | 0 | 8 | 0 | 0 |
| 551.286021 | 551.286171 | 3702161 | 29 | 44 | 0 | 10 | 0 | 0 |
| 551.322385 | 551.322557 | 2123151 | 30 | 48 | 0 | 9 | 0 | 0 |
| 553.09855 | 553.098764 | 1677219 | 27 | 22 | 0 | 13 | 0 | 0 |
| 553.135042 | 553.13515 | 2235680 | 28 | 26 | 0 | 12 | 0 | 0 |
| 553.171525 | 553.171535 | 2875166 | 29 | 30 | 0 | 11 | 0 | 0 |
| 553.17511 | 553.174906 | 2034462 | 26 | 34 | 0 | 11 | 1 | 0 |
| 553.192712 | 553.192665 | 1714461 | 26 | 34 | 0 | 13 | 0 | 0 |
| 553.20777 | 553.207921 | 3772700 | 30 | 34 | 0 | 10 | 0 | 0 |
| 553.211373 | 553.211292 | 2225436 | 27 | 38 | 0 | 10 | 1 | 0 |
| 553.229158 | 553.22905 | 3050267 | 27 | 38 | 0 | 12 | 0 | 0 |
| 553.243969 | 553.244306 | 3507866 | 31 | 38 | 0 | 9 | 0 | 0 |
| 553.265302 | 553.265436 | 2178329 | 28 | 42 | 0 | 11 | 0 | 0 |
| 553.280541 | 553.280692 | 2506520 | 32 | 42 | 0 | 8 | 0 | 0 |
| 553.301628 | 553.301821 | 1404950 | 29 | 46 | 0 | 10 | 0 | 0 |
| 554.268872 | 554.269416 | 1371102 | 30 | 41 | 3 | 5 | 1 | 0 |
| 555.114123 | 555.114414 | 1768621 | 27 | 24 | 0 | 13 | 0 | 0 |
| 555.150711 | 555.1508 | 2205611 | 28 | 28 | 0 | 12 | 0 | 0 |
| 555.18713 | 555.187185 | 4254889 | 29 | 32 | 0 | 11 | 0 | 0 |
| 555.190881 | 555.190556 | 1724329 | 26 | 36 | 0 | 11 | 1 | 0 |
| 555.223595 | 555.223571 | 6535335 | 30 | 36 | 0 | 10 | 0 | 0 |
| 555.244665 | 555.2447 | 1511845 | 27 | 40 | 0 | 12 | 0 | 0 |
| 555.259874 | 555.259956 | 4526245 | 31 | 40 | 0 | 9 | 0 | 0 |
| 555.296282 | 555.296342 | 2764963 | 32 | 44 | 0 | 8 | 0 | 0 |
| 557.109052 | 557.108935 | 1328447 | 30 | 22 | 0 | 11 | 0 | 0 |
| 557.130076 | 557.130064 | 1703741 | 27 | 26 | 0 | 13 | 0 | 0 |
| 557.166445 | 557.16645 | 2879548 | 28 | 30 | 0 | 12 | 0 | 0 |
| 557.202787 | 557.202835 | 5478458 | 29 | 34 | 0 | 11 | 0 | 0 |
| 557.218189 | 557.218092 | 1347129 | 33 | 34 | 0 | 8 | 0 | 0 |
| 557.239099 | 557.239221 | 7493688 | 30 | 38 | 0 | 10 | 0 | 0 |
| 557.27552 | 557.275606 | 3954998 | 31 | 42 | 0 | 9 | 0 | 0 |
| 557.311608 | 557.311992 | 2234420 | 32 | 46 | 0 | 8 | 0 | 0 |
| 558.279127 | 558.279586 | 2042369 | 33 | 41 | 3 | 3 | 1 | 0 |
| 559.088168 | 559.0882 | 1818072 | 29 | 20 | 0 | 12 | 0 | 0 |
| 559.124686 | 559.124585 | 2081238 | 30 | 24 | 0 | 11 | 0 | 0 |
| 559.145852 | 559.145715 | 1651157 | 27 | 28 | 0 | 13 | 0 | 0 |
| 559.16109 | 559.160971 | 1458644 | 31 | 28 | 0 | 10 | 0 | 0 |
| 559.182056 | 559.1821 | 4306387 | 28 | 32 | 0 | 12 | 0 | 0 |
| 559.197561 | 559.197356 | 1334354 | 32 | 32 | 0 | 9 | 0 | 0 |
| 559.218305 | 559.218486 | 7245777 | 29 | 36 | 0 | 11 | 0 | 0 |
| 559.233717 | 559.233742 | 1569744 | 33 | 36 | 0 | 8 | 0 | 0 |
| 559.25484 | 559.254871 | 8721359 | 30 | 40 | 0 | 10 | 0 | 0 |
| 559.291162 | 559.291257 | 3557325 | 31 | 44 | 0 | 9 | 0 | 0 |
| 559.327848 | 559.327642 | 1345995 | 32 | 48 | 0 | 8 | 0 | 0 |
| 561.103374 | 561.10385 | 1475061 | 29 | 22 | 0 | 12 | 0 | 0 |
| 561.140273 | 561.140235 | 1521651 | 30 | 26 | 0 | 11 | 0 | 0 |
| 561.160787 | 561.160852 | 2558834 | 40 | 22 | 2 | 2 | 0 | 0 |
| 561.176209 | 561.176621 | 1800050 | 31 | 30 | 0 | 10 | 0 | 0 |
| 561.197781 | 561.19775 | 5681009 | 28 | 34 | 0 | 12 | 0 | 0 |
| 561.213084 | 561.213006 | 2074480 | 32 | 34 | 0 | 9 | 0 | 0 |
| 561.233933 | 561.234136 | 8651631 | 29 | 38 | 0 | 11 | 0 | 0 |
| 561.249498 | 561.249392 | 2058606 | 33 | 38 | 0 | 8 | 0 | 0 |
| 561.270364 | 561.270521 | 7317869 | 30 | 42 | 0 | 10 | 0 | 0 |
| 561.285466 | 561.285777 | 1425004 | 34 | 42 | 0 | 7 | 0 | 0 |
| 561.30688 | 561.306907 | 2452331 | 31 | 46 | 0 | 9 | 0 | 0 |
| 563.118973 | 563.1195 | 1663001 | 29 | 24 | 0 | 12 | 0 | 0 |
| 563.155726 | 563.155885 | 1908055 | 30 | 28 | 0 | 11 | 0 | 0 |
| 563.176824 | 563.177015 | 3510550 | 27 | 32 | 0 | 13 | 0 | 0 |
| 563.192411 | 563.192271 | 2024726 | 31 | 32 | 0 | 10 | 0 | 0 |
| 563.195572 | 563.195642 | 1605909 | 28 | 36 | 0 | 10 | 1 | 0 |
| 563.213357 | 563.2134 | 6030101 | 28 | 36 | 0 | 12 | 0 | 0 |
| 563.228511 | 563.228656 | 3058452 | 32 | 36 | 0 | 9 | 0 | 0 |
| 563.249787 | 563.249786 | 7381779 | 29 | 40 | 0 | 11 | 0 | 0 |
| 563.265099 | 563.265042 | 1839378 | 33 | 40 | 0 | 8 | 0 | 0 |
| 563.285947 | 563.286171 | 5670162 | 30 | 44 | 0 | 10 | 0 | 0 |
| 563.301278 | 563.301427 | 1359377 | 34 | 44 | 0 | 7 | 0 | 0 |
| 565.134658 | 565.13515 | 1622211 | 29 | 26 | 0 | 12 | 0 | 0 |
| 565.155863 | 565.156279 | 1328834 | 26 | 30 | 0 | 14 | 0 | 0 |
| 565.171275 | 565.171535 | 2180801 | 30 | 30 | 0 | 11 | 0 | 0 |
| 565.17515 | 565.174906 | 1490113 | 27 | 34 | 0 | 11 | 1 | 0 |
| 565.192267 | 565.192665 | 2856128 | 27 | 34 | 0 | 13 | 0 | 0 |
| 565.207947 | 565.207921 | 2489792 | 31 | 34 | 0 | 10 | 0 | 0 |
| 565.211641 | 565.211292 | 1687231 | 28 | 38 | 0 | 10 | 1 | 0 |
| 565.229016 | 565.22905 | 4433343 | 28 | 38 | 0 | 12 | 0 | 0 |
| 565.244172 | 565.244306 | 2872766 | 32 | 38 | 0 | 9 | 0 | 0 |
| 565.265339 | 565.265436 | 4257981 | 29 | 42 | 0 | 11 | 0 | 0 |
| 565.280538 | 565.280692 | 2196669 | 33 | 42 | 0 | 8 | 0 | 0 |
| 565.301725 | 565.301821 | 2910396 | 30 | 46 | 0 | 10 | 0 | 0 |
| 567.113612 | 567.113762 | 1447540 | 20 | 28 | 2 | 15 | 1 | 0 |
| 567.150697 | 567.1508 | 2208626 | 29 | 28 | 0 | 12 | 0 | 0 |
| 567.18739 | 567.187185 | 2815089 | 30 | 32 | 0 | 11 | 0 | 0 |
| 567.208546 | 567.208315 | 2537072 | 27 | 36 | 0 | 13 | 0 | 0 |
| 567.223535 | 567.223571 | 2890096 | 31 | 36 | 0 | 10 | 0 | 0 |
| 567.22709 | 567.226942 | 2282607 | 28 | 40 | 0 | 10 | 1 | 0 |
| 567.24487 | 567.2447 | 2385519 | 28 | 40 | 0 | 12 | 0 | 0 |
| 567.259884 | 567.259956 | 3299438 | 32 | 40 | 0 | 9 | 0 | 0 |
| 567.263203 | 567.263327 | 1784942 | 29 | 44 | 0 | 9 | 1 | 0 |
| 567.280883 | 567.281086 | 1601773 | 29 | 44 | 0 | 11 | 0 | 0 |
| 567.296149 | 567.296342 | 1696877 | 33 | 44 | 0 | 8 | 0 | 0 |
| 569.129992 | 569.130064 | 1688489 | 28 | 26 | 0 | 13 | 0 | 0 |
| 569.166268 | 569.16645 | 2866728 | 29 | 30 | 0 | 12 | 0 | 0 |
| 569.202574 | 569.202835 | 3505446 | 30 | 34 | 0 | 11 | 0 | 0 |
| 569.206276 | 569.206206 | 1852454 | 27 | 38 | 0 | 11 | 1 | 0 |
| 569.239133 | 569.239221 | 3839013 | 31 | 38 | 0 | 10 | 0 | 0 |
| 569.275442 | 569.275606 | 2935332 | 32 | 42 | 0 | 9 | 0 | 0 |
| 569.311949 | 569.311992 | 1469987 | 33 | 46 | 0 | 8 | 0 | 0 |
| 571.145634 | 571.145715 | 1768420 | 28 | 28 | 0 | 13 | 0 | 0 |
| 571.181883 | 571.1821 | 3483619 | 29 | 32 | 0 | 12 | 0 | 0 |
| 571.218302 | 571.218486 | 4189409 | 30 | 36 | 0 | 11 | 0 | 0 |
| 571.254576 | 571.254871 | 4185568 | 31 | 40 | 0 | 10 | 0 | 0 |
| 571.290954 | 571.291257 | 2813663 | 32 | 44 | 0 | 9 | 0 | 0 |
| 571.327394 | 571.327642 | 1519582 | 33 | 48 | 0 | 8 | 0 | 0 |
| 573.139995 | 573.140235 | 1653413 | 31 | 26 | 0 | 11 | 0 | 0 |
| 573.161215 | 573.161365 | 2271140 | 28 | 30 | 0 | 13 | 0 | 0 |
| 573.175986 | 573.175969 | 1501859 | 24 | 34 | 2 | 12 | 1 | 0 |
| 573.197771 | 573.19775 | 4560291 | 29 | 34 | 0 | 12 | 0 | 0 |
| 573.213001 | 573.213006 | 1458850 | 33 | 34 | 0 | 9 | 0 | 0 |
| 573.234177 | 573.234136 | 5679522 | 30 | 38 | 0 | 11 | 0 | 0 |
| 573.249003 | 573.249392 | 1572257 | 34 | 38 | 0 | 8 | 0 | 0 |
| 573.270339 | 573.270521 | 3942817 | 31 | 42 | 0 | 10 | 0 | 0 |
| 573.306951 | 573.306907 | 2608544 | 32 | 46 | 0 | 9 | 0 | 0 |
| 575.141156 | 575.140629 | 1377642 | 27 | 28 | 0 | 14 | 0 | 0 |
| 575.155744 | 575.155885 | 1787242 | 31 | 28 | 0 | 11 | 0 | 0 |
| 575.176827 | 575.177015 | 2550121 | 28 | 32 | 0 | 13 | 0 | 0 |
| 575.192127 | 575.192271 | 1379177 | 32 | 32 | 0 | 10 | 0 | 0 |
| 575.213398 | 575.2134 | 5276008 | 29 | 36 | 0 | 12 | 0 | 0 |
| 575.228659 | 575.228656 | 1776488 | 33 | 36 | 0 | 9 | 0 | 0 |
| 575.249807 | 575.249786 | 6477671 | 30 | 40 | 0 | 11 | 0 | 0 |
| 575.265123 | 575.265042 | 1643623 | 34 | 40 | 0 | 8 | 0 | 0 |
| 575.286051 | 575.286171 | 3124582 | 31 | 44 | 0 | 10 | 0 | 0 |
| 575.322631 | 575.322557 | 1681509 | 32 | 48 | 0 | 9 | 0 | 0 |
| 577.134295 | 577.134498 | 1384245 | 22 | 30 | 2 | 14 | 1 | 0 |
| 577.156163 | 577.156279 | 1552180 | 27 | 30 | 0 | 14 | 0 | 0 |
| 577.17156 | 577.171535 | 1721140 | 31 | 30 | 0 | 11 | 0 | 0 |
| 577.192513 | 577.192665 | 3212339 | 28 | 34 | 0 | 13 | 0 | 0 |
| 577.207766 | 577.207921 | 1948979 | 32 | 34 | 0 | 10 | 0 | 0 |
| 577.228907 | 577.22905 | 5087538 | 29 | 38 | 0 | 12 | 0 | 0 |
| 577.243815 | 577.244306 | 1667122 | 33 | 38 | 0 | 9 | 0 | 0 |
| 577.265212 | 577.265436 | 5117233 | 30 | 42 | 0 | 11 | 0 | 0 |
| 577.280763 | 577.280692 | 1556529 | 34 | 42 | 0 | 8 | 0 | 0 |
| 577.301751 | 577.301821 | 1836336 | 31 | 46 | 0 | 10 | 0 | 0 |
| 579.150839 | 579.1508 | 1379843 | 30 | 28 | 0 | 12 | 0 | 0 |
| 579.171299 | 579.171416 | 1351939 | 40 | 24 | 2 | 3 | 0 | 0 |
| 579.187174 | 579.187185 | 2158338 | 31 | 32 | 0 | 11 | 0 | 0 |
| 579.208121 | 579.208315 | 3318530 | 28 | 36 | 0 | 13 | 0 | 0 |
| 579.223341 | 579.223571 | 2934530 | 32 | 36 | 0 | 10 | 0 | 0 |
| 579.244676 | 579.2447 | 3766529 | 29 | 40 | 0 | 12 | 0 | 0 |
| 579.259683 | 579.259956 | 2287361 | 33 | 40 | 0 | 9 | 0 | 0 |
| 579.280875 | 579.281086 | 3628288 | 30 | 44 | 0 | 11 | 0 | 0 |
| 579.296203 | 579.296342 | 1443584 | 34 | 44 | 0 | 8 | 0 | 0 |
| 581.166355 | 581.16645 | 1636566 | 30 | 30 | 0 | 12 | 0 | 0 |
| 581.20272 | 581.202835 | 2356438 | 31 | 34 | 0 | 11 | 0 | 0 |
| 581.206148 | 581.206206 | 1785302 | 28 | 38 | 0 | 11 | 1 | 0 |
| 581.223266 | 581.223313 | 1828565 | 20 | 42 | 2 | 15 | 1 | 0 |
| 581.238865 | 581.239221 | 2696917 | 32 | 38 | 0 | 10 | 0 | 0 |
| 581.259599 | 581.259698 | 1962964 | 21 | 46 | 2 | 14 | 1 | 0 |
| 581.275109 | 581.275606 | 2361556 | 33 | 42 | 0 | 9 | 0 | 0 |
| 581.296357 | 581.296736 | 1538004 | 30 | 46 | 0 | 11 | 0 | 0 |
| 583.108577 | 583.108677 | 1463471 | 20 | 28 | 2 | 16 | 1 | 0 |
| 583.145692 | 583.145715 | 1602734 | 29 | 28 | 0 | 13 | 0 | 0 |
| 583.182177 | 583.1821 | 2309806 | 30 | 32 | 0 | 12 | 0 | 0 |
| 583.18539 | 583.185471 | 1582382 | 27 | 36 | 0 | 12 | 1 | 0 |
| 583.218342 | 583.218486 | 2851757 | 31 | 36 | 0 | 11 | 0 | 0 |
| 583.221717 | 583.221856 | 1929389 | 28 | 40 | 0 | 11 | 1 | 0 |
| 583.254721 | 583.254871 | 2911916 | 32 | 40 | 0 | 10 | 0 | 0 |
| 583.291011 | 583.291257 | 2279084 | 33 | 44 | 0 | 9 | 0 | 0 |
| 583.327391 | 583.327642 | 1461163 | 34 | 48 | 0 | 8 | 0 | 0 |
| 585.161241 | 585.161365 | 1595146 | 29 | 30 | 0 | 13 | 0 | 0 |
| 585.197341 | 585.19775 | 2827657 | 30 | 34 | 0 | 12 | 0 | 0 |
| 585.200872 | 585.201121 | 1398793 | 27 | 38 | 0 | 12 | 1 | 0 |
| 585.234068 | 585.234136 | 3408521 | 31 | 38 | 0 | 11 | 0 | 0 |
| 585.270673 | 585.270521 | 2666632 | 32 | 42 | 0 | 10 | 0 | 0 |
| 585.307013 | 585.306907 | 1882759 | 33 | 46 | 0 | 9 | 0 | 0 |
| 587.177097 | 587.177015 | 2660457 | 29 | 32 | 0 | 13 | 0 | 0 |
| 587.213238 | 587.2134 | 3185513 | 30 | 36 | 0 | 12 | 0 | 0 |
| 587.249804 | 587.249786 | 3658344 | 31 | 40 | 0 | 11 | 0 | 0 |
| 587.285751 | 587.286171 | 3114728 | 32 | 44 | 0 | 10 | 0 | 0 |
| 587.32204 | 587.322557 | 1328487 | 33 | 48 | 0 | 9 | 0 | 0 |
| 589.098717 | 589.098764 | 1377614 | 30 | 22 | 0 | 13 | 0 | 0 |
| 589.156084 | 589.156279 | 1386637 | 28 | 30 | 0 | 14 | 0 | 0 |
| 589.192459 | 589.192665 | 2992204 | 29 | 34 | 0 | 13 | 0 | 0 |
| 589.207509 | 589.207921 | 1824844 | 33 | 34 | 0 | 10 | 0 | 0 |
| 589.228863 | 589.22905 | 4061772 | 30 | 38 | 0 | 12 | 0 | 0 |
| 589.265165 | 589.265436 | 3547211 | 31 | 42 | 0 | 11 | 0 | 0 |
| 589.301877 | 589.301821 | 2081099 | 32 | 46 | 0 | 10 | 0 | 0 |
| 591.171964 | 591.171929 | 1419827 | 28 | 32 | 0 | 14 | 0 | 0 |
| 591.186911 | 591.187185 | 1671987 | 32 | 32 | 0 | 11 | 0 | 0 |
| 591.20862 | 591.208315 | 2382387 | 29 | 36 | 0 | 13 | 0 | 0 |
| 591.223232 | 591.223571 | 1536819 | 33 | 36 | 0 | 10 | 0 | 0 |
| 591.244242 | 591.2447 | 3945522 | 30 | 40 | 0 | 12 | 0 | 0 |
| 591.259721 | 591.259956 | 1540914 | 34 | 40 | 0 | 9 | 0 | 0 |
| 591.281079 | 591.281086 | 2479666 | 31 | 44 | 0 | 11 | 0 | 0 |
| 593.18782 | 593.187579 | 1550109 | 28 | 34 | 0 | 14 | 0 | 0 |
| 593.22377 | 593.223965 | 2683933 | 29 | 38 | 0 | 13 | 0 | 0 |
| 593.239095 | 593.239221 | 2047261 | 33 | 38 | 0 | 10 | 0 | 0 |
| 593.260385 | 593.26035 | 3031068 | 30 | 42 | 0 | 12 | 0 | 0 |
| 593.275278 | 593.275606 | 1849116 | 34 | 42 | 0 | 9 | 0 | 0 |
| 593.296704 | 593.296736 | 1905692 | 31 | 46 | 0 | 11 | 0 | 0 |
| 595.145654 | 595.145715 | 1493770 | 30 | 28 | 0 | 13 | 0 | 0 |
| 595.181881 | 595.1821 | 1403658 | 31 | 32 | 0 | 12 | 0 | 0 |
| 595.203197 | 595.203229 | 1339146 | 28 | 36 | 0 | 14 | 0 | 0 |
| 595.218197 | 595.218486 | 2094602 | 32 | 36 | 0 | 11 | 0 | 0 |
| 595.221936 | 595.221856 | 1485322 | 29 | 40 | 0 | 11 | 1 | 0 |
| 595.239867 | 595.239615 | 1936394 | 29 | 40 | 0 | 13 | 0 | 0 |
| 595.254899 | 595.254871 | 1953034 | 33 | 40 | 0 | 10 | 0 | 0 |
| 595.258356 | 595.258242 | 1431049 | 30 | 44 | 0 | 10 | 1 | 0 |
| 595.276106 | 595.276 | 1688073 | 30 | 44 | 0 | 12 | 0 | 0 |
| 595.291087 | 595.291257 | 1710345 | 34 | 44 | 0 | 9 | 0 | 0 |
| 597.124842 | 597.124979 | 1416954 | 29 | 26 | 0 | 14 | 0 | 0 |
| 597.161581 | 597.161365 | 1375354 | 30 | 30 | 0 | 13 | 0 | 0 |
| 597.197627 | 597.19775 | 2661882 | 31 | 34 | 0 | 12 | 0 | 0 |
| 597.201102 | 597.201121 | 1545978 | 28 | 38 | 0 | 12 | 1 | 0 |
| 597.233824 | 597.234136 | 2607354 | 32 | 38 | 0 | 11 | 0 | 0 |
| 597.270678 | 597.270521 | 2212345 | 33 | 42 | 0 | 10 | 0 | 0 |
| 597.306932 | 597.306907 | 1780985 | 34 | 46 | 0 | 9 | 0 | 0 |
| 599.176717 | 599.177015 | 1918189 | 30 | 32 | 0 | 13 | 0 | 0 |
| 599.213308 | 599.2134 | 2285548 | 31 | 36 | 0 | 12 | 0 | 0 |
| 599.249792 | 599.249786 | 2705388 | 32 | 40 | 0 | 11 | 0 | 0 |
| 599.286237 | 599.286171 | 2123244 | 33 | 44 | 0 | 10 | 0 | 0 |
| 599.322472 | 599.322557 | 1601260 | 34 | 48 | 0 | 9 | 0 | 0 |
| 601.155996 | 601.156279 | 1353186 | 29 | 30 | 0 | 14 | 0 | 0 |
| 601.192561 | 601.192665 | 1997026 | 30 | 34 | 0 | 13 | 0 | 0 |
| 601.228505 | 601.22905 | 2832353 | 31 | 38 | 0 | 12 | 0 | 0 |
| 601.301365 | 601.301821 | 1810401 | 33 | 46 | 0 | 10 | 0 | 0 |
| 603.171941 | 603.171929 | 1848793 | 29 | 32 | 0 | 14 | 0 | 0 |
| 603.208185 | 603.208315 | 2513369 | 30 | 36 | 0 | 13 | 0 | 0 |
| 603.244425 | 603.2447 | 3366361 | 31 | 40 | 0 | 12 | 0 | 0 |
| 603.281108 | 603.281086 | 2087001 | 32 | 44 | 0 | 11 | 0 | 0 |
| 605.202687 | 605.202835 | 1543378 | 33 | 34 | 0 | 11 | 0 | 0 |
| 605.223854 | 605.223965 | 2770386 | 30 | 38 | 0 | 13 | 0 | 0 |
| 605.239733 | 605.239221 | 1494226 | 34 | 38 | 0 | 10 | 0 | 0 |
| 605.260246 | 605.26035 | 2578898 | 31 | 42 | 0 | 12 | 0 | 0 |
| 605.275757 | 605.275606 | 1864146 | 35 | 42 | 0 | 9 | 0 | 0 |
| 605.296723 | 605.296736 | 1912274 | 32 | 46 | 0 | 11 | 0 | 0 |
| 607.145502 | 607.145715 | 1352398 | 31 | 28 | 0 | 13 | 0 | 0 |
| 607.181368 | 607.181448 | 1604558 | 24 | 36 | 2 | 14 | 1 | 0 |
| 607.203173 | 607.203229 | 1842638 | 29 | 36 | 0 | 14 | 0 | 0 |
| 607.218492 | 607.218486 | 1676237 | 33 | 36 | 0 | 11 | 0 | 0 |
| 607.239269 | 607.239615 | 2420942 | 30 | 40 | 0 | 13 | 0 | 0 |
| 607.254887 | 607.254871 | 1625037 | 34 | 40 | 0 | 10 | 0 | 0 |
| 607.275836 | 607.276 | 1744717 | 31 | 44 | 0 | 12 | 0 | 0 |
| 607.290746 | 607.291257 | 1721293 | 35 | 44 | 0 | 9 | 0 | 0 |
| 609.197677 | 609.19775 | 1428426 | 32 | 34 | 0 | 12 | 0 | 0 |
| 609.237778 | 609.237506 | 1789898 | 30 | 42 | 0 | 11 | 1 | 0 |
| 609.255276 | 609.255265 | 1628618 | 30 | 42 | 0 | 13 | 0 | 0 |
| 609.270295 | 609.270521 | 1957578 | 34 | 42 | 0 | 10 | 0 | 0 |
| 611.177082 | 611.177015 | 1595337 | 31 | 32 | 0 | 13 | 0 | 0 |
| 611.212298 | 611.212748 | 1942473 | 24 | 40 | 2 | 14 | 1 | 0 |
| 611.216962 | 611.216771 | 1857481 | 29 | 40 | 0 | 12 | 1 | 0 |
| 611.249666 | 611.249786 | 2363337 | 33 | 40 | 0 | 11 | 0 | 0 |
| 611.286394 | 611.286171 | 1953737 | 34 | 44 | 0 | 10 | 0 | 0 |
| 613.192467 | 613.192665 | 1567177 | 31 | 34 | 0 | 13 | 0 | 0 |
| 613.22891 | 613.22905 | 2226377 | 32 | 38 | 0 | 12 | 0 | 0 |
| 613.265401 | 613.265436 | 1968585 | 33 | 42 | 0 | 11 | 0 | 0 |
| 613.301474 | 613.301821 | 1611209 | 34 | 46 | 0 | 10 | 0 | 0 |
| 615.208192 | 615.208315 | 1550154 | 31 | 36 | 0 | 13 | 0 | 0 |
| 615.244472 | 615.2447 | 2373066 | 32 | 40 | 0 | 12 | 0 | 0 |
| 615.280935 | 615.281086 | 2161098 | 33 | 44 | 0 | 11 | 0 | 0 |
| 615.317678 | 615.317471 | 1645002 | 34 | 48 | 0 | 10 | 0 | 0 |
| 617.187262 | 617.187579 | 1855435 | 30 | 34 | 0 | 14 | 0 | 0 |
| 617.223201 | 617.223313 | 2162635 | 23 | 42 | 2 | 15 | 1 | 0 |
| 617.26014 | 617.26035 | 2424780 | 32 | 42 | 0 | 12 | 0 | 0 |
| 617.296481 | 617.296736 | 2018507 | 33 | 46 | 0 | 11 | 0 | 0 |
| 619.203208 | 619.203229 | 1435086 | 30 | 36 | 0 | 14 | 0 | 0 |
| 619.238587 | 619.238963 | 2020302 | 23 | 44 | 2 | 15 | 1 | 0 |
| 619.276114 | 619.276 | 1770958 | 32 | 44 | 0 | 12 | 0 | 0 |
| 619.312156 | 619.312386 | 1501646 | 33 | 48 | 0 | 11 | 0 | 0 |
| 621.182404 | 621.182494 | 1304529 | 29 | 34 | 0 | 15 | 0 | 0 |
| 621.218946 | 621.218879 | 1782225 | 30 | 38 | 0 | 14 | 0 | 0 |
| 621.25456 | 621.254613 | 2163665 | 23 | 46 | 2 | 15 | 1 | 0 |
| 621.269399 | 621.269869 | 1329105 | 27 | 46 | 2 | 12 | 1 | 0 |
| 621.291783 | 621.29165 | 1691601 | 32 | 46 | 0 | 12 | 0 | 0 |
| 621.306773 | 621.306907 | 1468113 | 36 | 46 | 0 | 9 | 0 | 0 |
| 623.213242 | 623.2134 | 1644244 | 33 | 36 | 0 | 12 | 0 | 0 |
| 623.249851 | 623.249786 | 1425108 | 34 | 40 | 0 | 11 | 0 | 0 |
| 623.285687 | 623.286171 | 1741268 | 35 | 44 | 0 | 10 | 0 | 0 |
| 625.192695 | 625.192665 | 1594328 | 32 | 34 | 0 | 13 | 0 | 0 |
| 625.228779 | 625.22905 | 1813080 | 33 | 38 | 0 | 12 | 0 | 0 |
| 625.249298 | 625.249148 | 1297368 | 24 | 43 | 4 | 13 | 0 | 1 |
| 625.265752 | 625.265436 | 1712088 | 34 | 42 | 0 | 11 | 0 | 0 |
| 625.301898 | 625.301821 | 1464024 | 35 | 46 | 0 | 10 | 0 | 0 |
| 627.208246 | 627.208315 | 1969627 | 32 | 36 | 0 | 13 | 0 | 0 |
| 627.211752 | 627.211824 | 1726427 | 50 | 28 | 0 | 0 | 0 | 0 |
| 627.244036 | 627.244048 | 1566171 | 25 | 44 | 2 | 14 | 1 | 0 |
| 627.281118 | 627.281086 | 2275803 | 34 | 44 | 0 | 11 | 0 | 0 |
| 627.317482 | 627.317471 | 1691099 | 35 | 48 | 0 | 10 | 0 | 0 |
| 629.187035 | 629.187579 | 1399967 | 31 | 34 | 0 | 14 | 0 | 0 |
| 629.223829 | 629.223965 | 1842399 | 32 | 38 | 0 | 13 | 0 | 0 |
| 629.260172 | 629.26035 | 1841631 | 33 | 42 | 0 | 12 | 0 | 0 |
| 629.295804 | 629.296084 | 1676511 | 26 | 50 | 2 | 13 | 1 | 0 |
| 629.331753 | 629.332089 | 1308639 | 29 | 51 | 4 | 9 | 0 | 1 |
| 631.203302 | 631.203229 | 1698530 | 31 | 36 | 0 | 14 | 0 | 0 |
| 631.218129 | 631.218486 | 1292770 | 35 | 36 | 0 | 11 | 0 | 0 |
| 631.239147 | 631.239615 | 2041570 | 32 | 40 | 0 | 13 | 0 | 0 |
| 631.276038 | 631.276 | 1892322 | 33 | 44 | 0 | 12 | 0 | 0 |
| 631.311461 | 631.311734 | 1524194 | 26 | 52 | 2 | 13 | 1 | 0 |
| 633.197635 | 633.19775 | 1501925 | 34 | 34 | 0 | 12 | 0 | 0 |
| 633.218534 | 633.218879 | 1628389 | 31 | 38 | 0 | 14 | 0 | 0 |
| 633.254679 | 633.255265 | 1874917 | 32 | 42 | 0 | 13 | 0 | 0 |
| 633.291628 | 633.29165 | 1668581 | 33 | 46 | 0 | 12 | 0 | 0 |
| 635.212999 | 635.2134 | 1461224 | 34 | 36 | 0 | 12 | 0 | 0 |
| 635.234562 | 635.23453 | 1328872 | 31 | 40 | 0 | 14 | 0 | 0 |
| 635.248814 | 635.249134 | 1574888 | 27 | 44 | 2 | 13 | 1 | 0 |
| 635.270858 | 635.270915 | 1440744 | 32 | 44 | 0 | 13 | 0 | 0 |
| 637.192645 | 637.192665 | 1519083 | 33 | 34 | 0 | 13 | 0 | 0 |
| 637.228863 | 637.22905 | 1647595 | 34 | 38 | 0 | 12 | 0 | 0 |
| 637.265423 | 637.265436 | 1706987 | 35 | 42 | 0 | 11 | 0 | 0 |
| 637.301827 | 637.301821 | 1390827 | 36 | 46 | 0 | 10 | 0 | 0 |
| 639.207151 | 639.207663 | 1508333 | 25 | 40 | 2 | 15 | 1 | 0 |
| 639.244437 | 639.2447 | 1971693 | 34 | 40 | 0 | 12 | 0 | 0 |
| 639.280773 | 639.281086 | 1511405 | 35 | 44 | 0 | 11 | 0 | 0 |
| 641.223616 | 641.223965 | 1979375 | 33 | 38 | 0 | 13 | 0 | 0 |
| 641.26009 | 641.26035 | 2027759 | 34 | 42 | 0 | 12 | 0 | 0 |
| 641.296865 | 641.296736 | 1855471 | 35 | 46 | 0 | 11 | 0 | 0 |
| 643.202489 | 643.202577 | 1385713 | 24 | 40 | 2 | 16 | 1 | 0 |
| 643.239731 | 643.239615 | 1953777 | 33 | 40 | 0 | 13 | 0 | 0 |
| 643.276006 | 643.276 | 1872369 | 34 | 44 | 0 | 12 | 0 | 0 |
| 643.312446 | 643.312386 | 1440753 | 35 | 48 | 0 | 11 | 0 | 0 |
| 645.254948 | 645.255265 | 2247154 | 33 | 42 | 0 | 13 | 0 | 0 |
| 645.291566 | 645.29165 | 1813234 | 34 | 46 | 0 | 12 | 0 | 0 |
| 647.234451 | 647.23453 | 1332979 | 32 | 40 | 0 | 14 | 0 | 0 |
| 647.248887 | 647.249134 | 1287155 | 28 | 44 | 2 | 13 | 1 | 0 |
| 647.271126 | 647.270915 | 2070003 | 33 | 44 | 0 | 13 | 0 | 0 |
| 649.229109 | 649.22905 | 1335283 | 35 | 38 | 0 | 12 | 0 | 0 |
| 649.250225 | 649.25018 | 1458675 | 32 | 42 | 0 | 14 | 0 | 0 |
| 649.286119 | 649.286565 | 1365235 | 33 | 46 | 0 | 13 | 0 | 0 |
| 651.244547 | 651.2447 | 1626355 | 35 | 40 | 0 | 12 | 0 | 0 |
| 651.317293 | 651.317471 | 1313779 | 37 | 48 | 0 | 10 | 0 | 0 |
| 653.223723 | 653.223965 | 1634034 | 34 | 38 | 0 | 13 | 0 | 0 |
| 653.26019 | 653.26035 | 1767410 | 35 | 42 | 0 | 12 | 0 | 0 |
| 653.296823 | 653.296736 | 1400050 | 36 | 46 | 0 | 11 | 0 | 0 |
| 655.239596 | 655.239615 | 1878000 | 34 | 40 | 0 | 13 | 0 | 0 |
| 655.276186 | 655.276 | 1446128 | 35 | 44 | 0 | 12 | 0 | 0 |
| 655.311465 | 655.311734 | 1464304 | 28 | 52 | 2 | 13 | 1 | 0 |
| 657.254941 | 657.255265 | 1994478 | 34 | 42 | 0 | 13 | 0 | 0 |
| 657.291832 | 657.29165 | 1593838 | 35 | 46 | 0 | 12 | 0 | 0 |
| 659.233739 | 659.233878 | 1518827 | 25 | 44 | 2 | 16 | 1 | 0 |
| 659.270122 | 659.270263 | 1733099 | 26 | 48 | 2 | 15 | 1 | 0 |
| 659.306345 | 659.306649 | 1409003 | 27 | 52 | 2 | 14 | 1 | 0 |
| 661.250354 | 661.25018 | 1698023 | 33 | 42 | 0 | 14 | 0 | 0 |
| 663.265803 | 663.26583 | 1456866 | 33 | 44 | 0 | 14 | 0 | 0 |
| 663.281036 | 663.281086 | 1407458 | 37 | 44 | 0 | 11 | 0 | 0 |
| 665.222987 | 665.223313 | 1860060 | 27 | 42 | 2 | 15 | 1 | 0 |
| 665.260249 | 665.26035 | 1380316 | 36 | 42 | 0 | 12 | 0 | 0 |
| 665.295574 | 665.295704 | 1285340 | 31 | 47 | 4 | 10 | 0 | 1 |
| 667.275678 | 667.276 | 1289237 | 36 | 44 | 0 | 12 | 0 | 0 |
| 669.255169 | 669.255265 | 1576398 | 35 | 42 | 0 | 13 | 0 | 0 |
| 669.291469 | 669.29165 | 1780686 | 36 | 46 | 0 | 12 | 0 | 0 |
| 671.234574 | 671.23453 | 1325253 | 34 | 40 | 0 | 14 | 0 | 0 |
| 671.270857 | 671.270915 | 1512389 | 35 | 44 | 0 | 13 | 0 | 0 |
| 671.306759 | 671.307301 | 1312709 | 36 | 48 | 0 | 12 | 0 | 0 |
| 673.286771 | 673.286565 | 1300411 | 35 | 46 | 0 | 13 | 0 | 0 |
| 673.32248 | 673.322951 | 1333691 | 36 | 50 | 0 | 12 | 0 | 0 |
| 675.265661 | 675.26583 | 1400240 | 34 | 44 | 0 | 14 | 0 | 0 |
| 675.301683 | 675.302215 | 1288624 | 35 | 48 | 0 | 13 | 0 | 0 |
| 679.276195 | 679.276 | 1520791 | 37 | 44 | 0 | 12 | 0 | 0 |
| 681.254818 | 681.255265 | 1575048 | 36 | 42 | 0 | 13 | 0 | 0 |
| 681.291371 | 681.29165 | 1484424 | 37 | 46 | 0 | 12 | 0 | 0 |
| 683.234252 | 683.23453 | 1707385 | 35 | 40 | 0 | 14 | 0 | 0 |
| 683.270568 | 683.270915 | 1571576 | 36 | 44 | 0 | 13 | 0 | 0 |
| 685.28521 | 685.285533 | 1292647 | 30 | 47 | 4 | 12 | 0 | 1 |
| 687.302135 | 687.302215 | 1279572 | 36 | 48 | 0 | 13 | 0 | 0 |
| 689.244872 | 689.245094 | 1362752 | 34 | 42 | 0 | 15 | 0 | 0 |
| 689.281252 | 689.28148 | 1316928 | 35 | 46 | 0 | 14 | 0 | 0 |
| 689.317198 | 689.317865 | 1324096 | 36 | 50 | 0 | 13 | 0 | 0 |
| 695.343423 | 695.343686 | 1426169 | 39 | 52 | 0 | 11 | 0 | 0 |
| 697.250201 | 697.25018 | 1471200 | 36 | 42 | 0 | 14 | 0 | 0 |
| 697.2864 | 697.286565 | 1722336 | 37 | 46 | 0 | 13 | 0 | 0 |
| 697.322904 | 697.322951 | 1294047 | 38 | 50 | 0 | 12 | 0 | 0 |

7. Sample: P1_T_15_ (bacteria-only)

| Experimental mass | Exact mass | Peak height | C | H | N | O | S | P |
| --- | --- | --- | --- | --- | --- | --- | --- | --- |
| 207.102604 | 207.102668 | 1288404 | 12 | 16 | 0 | 3 | 0 | 0 |
| 207.139017 | 207.139053 | 1249160 | 13 | 20 | 0 | 2 | 0 | 0 |
| 209.118294 | 209.118318 | 1473354 | 12 | 18 | 0 | 3 | 0 | 0 |
| 211.097556 | 211.097583 | 1220344 | 11 | 16 | 0 | 4 | 0 | 0 |
| 213.11321 | 213.113233 | 1678387 | 11 | 18 | 0 | 4 | 0 | 0 |
| 217.123397 | 217.123403 | 1280120 | 14 | 18 | 0 | 2 | 0 | 0 |
| 219.102636 | 219.102668 | 2040931 | 13 | 16 | 0 | 3 | 0 | 0 |
| 219.139032 | 219.139053 | 1636644 | 14 | 20 | 0 | 2 | 0 | 0 |
| 221.081899 | 221.081932 | 1390341 | 12 | 14 | 0 | 4 | 0 | 0 |
| 221.118276 | 221.118318 | 1978213 | 13 | 18 | 0 | 3 | 0 | 0 |
| 223.09757 | 223.097583 | 2566509 | 12 | 16 | 0 | 4 | 0 | 0 |
| 223.133969 | 223.133968 | 1891118 | 13 | 20 | 0 | 3 | 0 | 0 |
| 225.113212 | 225.113233 | 3301724 | 12 | 18 | 0 | 4 | 0 | 0 |
| 227.092448 | 227.092497 | 1281295 | 11 | 16 | 0 | 5 | 0 | 0 |
| 227.128872 | 227.128883 | 2213936 | 12 | 20 | 0 | 4 | 0 | 0 |
| 229.086974 | 229.087018 | 1183864 | 14 | 14 | 0 | 3 | 0 | 0 |
| 229.108081 | 229.108147 | 1523864 | 11 | 18 | 0 | 5 | 0 | 0 |
| 231.102648 | 231.102668 | 1850534 | 14 | 16 | 0 | 3 | 0 | 0 |
| 233.081896 | 233.081932 | 1654729 | 13 | 14 | 0 | 4 | 0 | 0 |
| 233.118295 | 233.118318 | 2312906 | 14 | 18 | 0 | 3 | 0 | 0 |
| 235.061189 | 235.061197 | 1163280 | 12 | 12 | 0 | 5 | 0 | 0 |
| 235.097561 | 235.097583 | 2706129 | 13 | 16 | 0 | 4 | 0 | 0 |
| 235.13395 | 235.133968 | 3136019 | 14 | 20 | 0 | 3 | 0 | 0 |
| 237.113208 | 237.113233 | 3877662 | 13 | 18 | 0 | 4 | 0 | 0 |
| 237.149592 | 237.149618 | 2234335 | 14 | 22 | 0 | 3 | 0 | 0 |
| 239.092492 | 239.092497 | 2762477 | 12 | 16 | 0 | 5 | 0 | 0 |
| 241.108129 | 241.108147 | 4117506 | 12 | 18 | 0 | 5 | 0 | 0 |
| 241.144529 | 241.144533 | 2801220 | 13 | 22 | 0 | 4 | 0 | 0 |
| 243.123809 | 243.123797 | 1900762 | 12 | 20 | 0 | 5 | 0 | 0 |
| 245.118298 | 245.118318 | 1667637 | 15 | 18 | 0 | 3 | 0 | 0 |
| 247.061141 | 247.061197 | 1294480 | 13 | 12 | 0 | 5 | 0 | 0 |
| 247.09756 | 247.097583 | 2610130 | 14 | 16 | 0 | 4 | 0 | 0 |
| 247.133959 | 247.133968 | 3281556 | 15 | 20 | 0 | 3 | 0 | 0 |
| 249.076843 | 249.076847 | 1651442 | 13 | 14 | 0 | 5 | 0 | 0 |
| 249.113217 | 249.113233 | 4148852 | 14 | 18 | 0 | 4 | 0 | 0 |
| 249.149597 | 249.149618 | 3160694 | 15 | 22 | 0 | 3 | 0 | 0 |
| 251.092483 | 251.092497 | 2891926 | 13 | 16 | 0 | 5 | 0 | 0 |
| 251.128864 | 251.128883 | 6075480 | 14 | 20 | 0 | 4 | 0 | 0 |
| 253.108128 | 253.108147 | 4936253 | 13 | 18 | 0 | 5 | 0 | 0 |
| 253.144499 | 253.144533 | 4799679 | 14 | 22 | 0 | 4 | 0 | 0 |
| 255.087396 | 255.087412 | 1645315 | 12 | 16 | 0 | 6 | 0 | 0 |
| 255.12378 | 255.123797 | 6449061 | 13 | 20 | 0 | 5 | 0 | 0 |
| 255.160182 | 255.160183 | 2826663 | 14 | 24 | 0 | 4 | 0 | 0 |
| 257.103038 | 257.103062 | 2284557 | 12 | 18 | 0 | 6 | 0 | 0 |
| 257.139413 | 257.139447 | 2935311 | 13 | 22 | 0 | 5 | 0 | 0 |
| 259.097554 | 259.097583 | 1760056 | 15 | 16 | 0 | 4 | 0 | 0 |
| 259.118722 | 259.118712 | 1282617 | 12 | 20 | 0 | 6 | 0 | 0 |
| 259.133919 | 259.133968 | 1560826 | 16 | 20 | 0 | 3 | 0 | 0 |
| 261.076792 | 261.076847 | 1999075 | 14 | 14 | 0 | 5 | 0 | 0 |
| 261.113237 | 261.113233 | 2497957 | 15 | 18 | 0 | 4 | 0 | 0 |
| 261.149612 | 261.149618 | 1603815 | 16 | 22 | 0 | 3 | 0 | 0 |
| 263.092475 | 263.092497 | 3486033 | 14 | 16 | 0 | 5 | 0 | 0 |
| 263.128866 | 263.128883 | 5441619 | 15 | 20 | 0 | 4 | 0 | 0 |
| 263.165212 | 263.165268 | 1489301 | 16 | 24 | 0 | 3 | 0 | 0 |
| 265.108132 | 265.108147 | 5407424 | 14 | 18 | 0 | 5 | 0 | 0 |
| 265.144515 | 265.144533 | 7759298 | 15 | 22 | 0 | 4 | 0 | 0 |
| 267.087374 | 267.087412 | 2854317 | 13 | 16 | 0 | 6 | 0 | 0 |
| 267.12378 | 267.123797 | 10399535 | 14 | 20 | 0 | 5 | 0 | 0 |
| 269.103033 | 269.103062 | 4615069 | 13 | 18 | 0 | 6 | 0 | 0 |
| 269.13942 | 269.139447 | 7232671 | 14 | 22 | 0 | 5 | 0 | 0 |
| 269.175797 | 269.175833 | 3273505 | 15 | 26 | 0 | 4 | 0 | 0 |
| 271.097648 | 271.097583 | 1475339 | 16 | 16 | 0 | 4 | 0 | 0 |
| 271.118693 | 271.118712 | 3437452 | 13 | 20 | 0 | 6 | 0 | 0 |
| 271.134055 | 271.133968 | 1454541 | 17 | 20 | 0 | 3 | 0 | 0 |
| 271.155049 | 271.155097 | 3440782 | 14 | 24 | 0 | 5 | 0 | 0 |
| 273.113183 | 273.113233 | 1992570 | 16 | 18 | 0 | 4 | 0 | 0 |
| 273.13435 | 273.134362 | 1610747 | 13 | 22 | 0 | 6 | 0 | 0 |
| 273.149609 | 273.149618 | 1588732 | 17 | 22 | 0 | 3 | 0 | 0 |
| 275.092476 | 275.092497 | 2335079 | 15 | 16 | 0 | 5 | 0 | 0 |
| 275.128915 | 275.128883 | 2633449 | 16 | 20 | 0 | 4 | 0 | 0 |
| 275.165365 | 275.165268 | 1303682 | 17 | 24 | 0 | 3 | 0 | 0 |
| 277.071751 | 277.071762 | 1822802 | 14 | 14 | 0 | 6 | 0 | 0 |
| 277.108136 | 277.108147 | 5605716 | 15 | 18 | 0 | 5 | 0 | 0 |
| 277.180878 | 277.180918 | 1625688 | 17 | 26 | 0 | 3 | 0 | 0 |
| 279.087386 | 279.087412 | 2762303 | 14 | 16 | 0 | 6 | 0 | 0 |
| 279.123784 | 279.123797 | 10052545 | 15 | 20 | 0 | 5 | 0 | 0 |
| 279.160154 | 279.160183 | 3804867 | 16 | 24 | 0 | 4 | 0 | 0 |
| 281.103039 | 281.103062 | 5176362 | 14 | 18 | 0 | 6 | 0 | 0 |
| 281.17579 | 281.175833 | 2351150 | 16 | 26 | 0 | 4 | 0 | 0 |
| 283.082317 | 283.082326 | 1744274 | 13 | 16 | 0 | 7 | 0 | 0 |
| 283.1187 | 283.118712 | 9850516 | 14 | 20 | 0 | 6 | 0 | 0 |
| 283.155076 | 283.155097 | 11167382 | 15 | 24 | 0 | 5 | 0 | 0 |
| 285.097948 | 285.097976 | 2209146 | 13 | 18 | 0 | 7 | 0 | 0 |
| 285.13433 | 285.134362 | 5539836 | 14 | 22 | 0 | 6 | 0 | 0 |
| 285.149893 | 285.149618 | 1389692 | 18 | 22 | 0 | 3 | 0 | 0 |
| 285.170718 | 285.170747 | 3859965 | 15 | 26 | 0 | 5 | 0 | 0 |
| 287.092473 | 287.092497 | 1811294 | 16 | 16 | 0 | 5 | 0 | 0 |
| 287.113684 | 287.113627 | 1232031 | 13 | 20 | 0 | 7 | 0 | 0 |
| 287.128887 | 287.128883 | 2332128 | 17 | 20 | 0 | 4 | 0 | 0 |
| 287.15 | 287.150012 | 2502753 | 14 | 24 | 0 | 6 | 0 | 0 |
| 287.165369 | 287.165268 | 2097378 | 18 | 24 | 0 | 3 | 0 | 0 |
| 289.0717 | 289.071762 | 1527488 | 15 | 14 | 0 | 6 | 0 | 0 |
| 289.108146 | 289.108147 | 3591874 | 16 | 18 | 0 | 5 | 0 | 0 |
| 289.144524 | 289.144533 | 4047555 | 17 | 22 | 0 | 4 | 0 | 0 |
| 289.18097 | 289.180918 | 1179941 | 18 | 26 | 0 | 3 | 0 | 0 |
| 291.087373 | 291.087412 | 2993570 | 15 | 16 | 0 | 6 | 0 | 0 |
| 291.123792 | 291.123797 | 5528611 | 16 | 20 | 0 | 5 | 0 | 0 |
| 291.160206 | 291.160183 | 3502373 | 17 | 24 | 0 | 4 | 0 | 0 |
| 293.066673 | 293.066676 | 1386368 | 14 | 14 | 0 | 7 | 0 | 0 |
| 293.103059 | 293.103062 | 6098562 | 15 | 18 | 0 | 6 | 0 | 0 |
| 293.139432 | 293.139447 | 9153667 | 16 | 22 | 0 | 5 | 0 | 0 |
| 295.08231 | 295.082326 | 2766559 | 14 | 16 | 0 | 7 | 0 | 0 |
| 295.118696 | 295.118712 | 13624544 | 15 | 20 | 0 | 6 | 0 | 0 |
| 295.155077 | 295.155097 | 9162466 | 16 | 24 | 0 | 5 | 0 | 0 |
| 295.191448 | 295.191483 | 2503396 | 17 | 28 | 0 | 4 | 0 | 0 |
| 297.097984 | 297.097976 | 3819068 | 14 | 18 | 0 | 7 | 0 | 0 |
| 297.113191 | 297.113233 | 1664445 | 18 | 18 | 0 | 4 | 0 | 0 |
| 297.13435 | 297.134362 | 13088061 | 15 | 22 | 0 | 6 | 0 | 0 |
| 297.170745 | 297.170747 | 4435775 | 16 | 26 | 0 | 5 | 0 | 0 |
| 299.113606 | 299.113627 | 5115801 | 14 | 20 | 0 | 7 | 0 | 0 |
| 299.128886 | 299.128883 | 1664281 | 18 | 20 | 0 | 4 | 0 | 0 |
| 299.150008 | 299.150012 | 7361434 | 15 | 24 | 0 | 6 | 0 | 0 |
| 299.165339 | 299.165268 | 1549339 | 19 | 24 | 0 | 3 | 0 | 0 |
| 301.108161 | 301.108147 | 2283123 | 17 | 18 | 0 | 5 | 0 | 0 |
| 301.12925 | 301.129277 | 3212020 | 14 | 22 | 0 | 7 | 0 | 0 |
| 301.144507 | 301.144533 | 2536309 | 18 | 22 | 0 | 4 | 0 | 0 |
| 301.181092 | 301.180918 | 1562870 | 19 | 26 | 0 | 3 | 0 | 0 |
| 303.087394 | 303.087412 | 2990157 | 16 | 16 | 0 | 6 | 0 | 0 |
| 303.123781 | 303.123797 | 5304911 | 17 | 20 | 0 | 5 | 0 | 0 |
| 303.160159 | 303.160183 | 4076624 | 18 | 24 | 0 | 4 | 0 | 0 |
| 303.196665 | 303.196568 | 1466034 | 19 | 28 | 0 | 3 | 0 | 0 |
| 305.06671 | 305.066676 | 1444006 | 15 | 14 | 0 | 7 | 0 | 0 |
| 305.103049 | 305.103062 | 5311144 | 16 | 18 | 0 | 6 | 0 | 0 |
| 305.139443 | 305.139447 | 7630505 | 17 | 22 | 0 | 5 | 0 | 0 |
| 305.17585 | 305.175833 | 2861483 | 18 | 26 | 0 | 4 | 0 | 0 |
| 307.082318 | 307.082326 | 3039233 | 15 | 16 | 0 | 7 | 0 | 0 |
| 307.118699 | 307.118712 | 9125123 | 16 | 20 | 0 | 6 | 0 | 0 |
| 307.155101 | 307.155097 | 8486148 | 17 | 24 | 0 | 5 | 0 | 0 |
| 307.191488 | 307.191483 | 2756614 | 18 | 28 | 0 | 4 | 0 | 0 |
| 309.097969 | 309.097976 | 5835100 | 15 | 18 | 0 | 7 | 0 | 0 |
| 309.134348 | 309.134362 | 15141726 | 16 | 22 | 0 | 6 | 0 | 0 |
| 309.170738 | 309.170747 | 8795487 | 17 | 26 | 0 | 5 | 0 | 0 |
| 311.077269 | 311.077241 | 1662774 | 14 | 16 | 0 | 8 | 0 | 0 |
| 311.113611 | 311.113627 | 12242872 | 15 | 20 | 0 | 7 | 0 | 0 |
| 311.149987 | 311.150012 | 12792761 | 16 | 24 | 0 | 6 | 0 | 0 |
| 311.186372 | 311.186398 | 4267963 | 17 | 28 | 0 | 5 | 0 | 0 |
| 313.092875 | 313.092891 | 2573074 | 14 | 18 | 0 | 8 | 0 | 0 |
| 313.108161 | 313.108147 | 1446227 | 18 | 18 | 0 | 5 | 0 | 0 |
| 313.129278 | 313.129277 | 8979476 | 15 | 22 | 0 | 7 | 0 | 0 |
| 313.144535 | 313.144533 | 2106261 | 19 | 22 | 0 | 4 | 0 | 0 |
| 313.165652 | 313.165662 | 5796374 | 16 | 26 | 0 | 6 | 0 | 0 |
| 313.18106 | 313.180918 | 1281814 | 20 | 26 | 0 | 3 | 0 | 0 |
| 313.202066 | 313.202048 | 1345815 | 17 | 30 | 0 | 5 | 0 | 0 |
| 315.087441 | 315.087412 | 1599342 | 17 | 16 | 0 | 6 | 0 | 0 |
| 315.100507 | 315.100298 | 1390575 | 14 | 21 | 0 | 6 | 0 | 1 |
| 315.108501 | 315.108541 | 1905519 | 14 | 20 | 0 | 8 | 0 | 0 |
| 315.123828 | 315.123797 | 2905712 | 18 | 20 | 0 | 5 | 0 | 0 |
| 315.144893 | 315.144927 | 4284273 | 15 | 24 | 0 | 7 | 0 | 0 |
| 315.160163 | 315.160183 | 3533426 | 19 | 24 | 0 | 4 | 0 | 0 |
| 317.103064 | 317.103062 | 3912908 | 17 | 18 | 0 | 6 | 0 | 0 |
| 317.116109 | 317.115949 | 2107085 | 14 | 23 | 0 | 6 | 0 | 1 |
| 317.124182 | 317.124191 | 1193165 | 14 | 22 | 0 | 8 | 0 | 0 |
| 317.139435 | 317.139447 | 6435534 | 18 | 22 | 0 | 5 | 0 | 0 |
| 317.175876 | 317.175833 | 4058576 | 19 | 26 | 0 | 4 | 0 | 0 |
| 319.082304 | 319.082326 | 3296554 | 16 | 16 | 0 | 7 | 0 | 0 |
| 319.118697 | 319.118712 | 9082156 | 17 | 20 | 0 | 6 | 0 | 0 |
| 319.131729 | 319.131599 | 2090029 | 14 | 25 | 0 | 6 | 0 | 1 |
| 319.155067 | 319.155097 | 9670958 | 18 | 24 | 0 | 5 | 0 | 0 |
| 319.191491 | 319.191483 | 4445232 | 19 | 28 | 0 | 4 | 0 | 0 |
| 321.097959 | 321.097976 | 5643147 | 16 | 18 | 0 | 7 | 0 | 0 |
| 321.134347 | 321.134362 | 14413197 | 17 | 22 | 0 | 6 | 0 | 0 |
| 321.170738 | 321.170747 | 9713039 | 18 | 26 | 0 | 5 | 0 | 0 |
| 322.129612 | 322.129611 | 1266878 | 16 | 21 | 1 | 6 | 0 | 0 |
| 323.077247 | 323.077241 | 2053228 | 15 | 16 | 0 | 8 | 0 | 0 |
| 323.113621 | 323.113627 | 11654638 | 16 | 20 | 0 | 7 | 0 | 0 |
| 323.186392 | 323.186398 | 6700530 | 18 | 28 | 0 | 5 | 0 | 0 |
| 325.092897 | 325.092891 | 4598865 | 15 | 18 | 0 | 8 | 0 | 0 |
| 325.129268 | 325.129277 | 16062035 | 16 | 22 | 0 | 7 | 0 | 0 |
| 325.165658 | 325.165662 | 12199509 | 17 | 26 | 0 | 6 | 0 | 0 |
| 327.087381 | 327.087412 | 1352247 | 18 | 16 | 0 | 6 | 0 | 0 |
| 327.108543 | 327.108541 | 7335097 | 15 | 20 | 0 | 8 | 0 | 0 |
| 327.123773 | 327.123797 | 2387897 | 19 | 20 | 0 | 5 | 0 | 0 |
| 327.14491 | 327.144927 | 12511930 | 16 | 24 | 0 | 7 | 0 | 0 |
| 327.160108 | 327.160183 | 1848251 | 20 | 24 | 0 | 4 | 0 | 0 |
| 327.181286 | 327.181312 | 6303420 | 17 | 28 | 0 | 6 | 0 | 0 |
| 327.196686 | 327.196568 | 1267197 | 21 | 28 | 0 | 3 | 0 | 0 |
| 329.087778 | 329.087806 | 1459296 | 14 | 18 | 0 | 9 | 0 | 0 |
| 329.103025 | 329.103062 | 3272993 | 18 | 18 | 0 | 6 | 0 | 0 |
| 329.124204 | 329.124191 | 4733730 | 15 | 22 | 0 | 8 | 0 | 0 |
| 329.139435 | 329.139447 | 5292323 | 19 | 22 | 0 | 5 | 0 | 0 |
| 329.160558 | 329.160577 | 5921060 | 16 | 26 | 0 | 7 | 0 | 0 |
| 329.175832 | 329.175833 | 3265573 | 20 | 26 | 0 | 4 | 0 | 0 |
| 331.08232 | 331.082326 | 2752651 | 17 | 16 | 0 | 7 | 0 | 0 |
| 331.118692 | 331.118712 | 6297997 | 18 | 20 | 0 | 6 | 0 | 0 |
| 331.131758 | 331.131599 | 1438862 | 15 | 25 | 0 | 6 | 0 | 1 |
| 331.139857 | 331.139841 | 2182542 | 15 | 24 | 0 | 8 | 0 | 0 |
| 331.155107 | 331.155097 | 6943119 | 19 | 24 | 0 | 5 | 0 | 0 |
| 331.176257 | 331.176227 | 1465872 | 16 | 28 | 0 | 7 | 0 | 0 |
| 331.191496 | 331.191483 | 4459921 | 20 | 28 | 0 | 4 | 0 | 0 |
| 333.061607 | 333.061591 | 1403001 | 16 | 14 | 0 | 8 | 0 | 0 |
| 333.097969 | 333.097976 | 4990971 | 17 | 18 | 0 | 7 | 0 | 0 |
| 333.111117 | 333.110863 | 1649531 | 14 | 23 | 0 | 7 | 0 | 1 |
| 333.134385 | 333.134362 | 10397693 | 18 | 22 | 0 | 6 | 0 | 0 |
| 333.170752 | 333.170747 | 11193343 | 19 | 26 | 0 | 5 | 0 | 0 |
| 333.207137 | 333.207133 | 4723713 | 20 | 30 | 0 | 4 | 0 | 0 |
| 335.077233 | 335.077241 | 2194283 | 16 | 16 | 0 | 8 | 0 | 0 |
| 335.113616 | 335.113627 | 12531821 | 17 | 20 | 0 | 7 | 0 | 0 |
| 335.150005 | 335.150012 | 19468400 | 18 | 24 | 0 | 6 | 0 | 0 |
| 335.186404 | 335.186398 | 11250801 | 19 | 28 | 0 | 5 | 0 | 0 |
| 336.145172 | 336.145261 | 1271337 | 17 | 23 | 1 | 6 | 0 | 0 |
| 337.092885 | 337.092891 | 6714593 | 16 | 18 | 0 | 8 | 0 | 0 |
| 337.129283 | 337.129277 | 22517986 | 17 | 22 | 0 | 7 | 0 | 0 |
| 337.165655 | 337.165662 | 19482854 | 18 | 26 | 0 | 6 | 0 | 0 |
| 338.124499 | 338.124526 | 1572382 | 16 | 21 | 1 | 7 | 0 | 0 |
| 339.07213 | 339.072156 | 1456728 | 15 | 16 | 0 | 9 | 0 | 0 |
| 339.087459 | 339.087412 | 1237721 | 19 | 16 | 0 | 6 | 0 | 0 |
| 339.108528 | 339.108541 | 14569818 | 16 | 20 | 0 | 8 | 0 | 0 |
| 339.1813 | 339.181312 | 14223711 | 18 | 28 | 0 | 6 | 0 | 0 |
| 339.217676 | 339.217698 | 3365473 | 19 | 32 | 0 | 5 | 0 | 0 |
| 341.087796 | 341.087806 | 2864341 | 15 | 18 | 0 | 9 | 0 | 0 |
| 341.103081 | 341.103062 | 2096086 | 19 | 18 | 0 | 6 | 0 | 0 |
| 341.116147 | 341.115949 | 1491287 | 16 | 23 | 0 | 6 | 0 | 1 |
| 341.124187 | 341.124191 | 14125528 | 16 | 22 | 0 | 8 | 0 | 0 |
| 341.139461 | 341.139447 | 2500569 | 20 | 22 | 0 | 5 | 0 | 0 |
| 341.160566 | 341.160577 | 13551066 | 17 | 26 | 0 | 7 | 0 | 0 |
| 341.175927 | 341.175833 | 1979611 | 21 | 26 | 0 | 4 | 0 | 0 |
| 343.082411 | 343.082326 | 1538901 | 18 | 16 | 0 | 7 | 0 | 0 |
| 343.103487 | 343.103456 | 2699607 | 15 | 20 | 0 | 9 | 0 | 0 |
| 343.118691 | 343.118712 | 4517464 | 19 | 20 | 0 | 6 | 0 | 0 |
| 343.131766 | 343.131599 | 1492825 | 16 | 25 | 0 | 6 | 0 | 1 |
| 343.139832 | 343.139841 | 6677081 | 16 | 24 | 0 | 8 | 0 | 0 |
| 343.155096 | 343.155097 | 4706906 | 20 | 24 | 0 | 5 | 0 | 0 |
| 343.176226 | 343.176227 | 4944476 | 17 | 28 | 0 | 7 | 0 | 0 |
| 343.191502 | 343.191483 | 2533213 | 21 | 28 | 0 | 4 | 0 | 0 |
| 345.097968 | 345.097976 | 3951835 | 18 | 18 | 0 | 7 | 0 | 0 |
| 345.111044 | 345.110863 | 1777884 | 15 | 23 | 0 | 7 | 0 | 1 |
| 345.119079 | 345.119106 | 1441501 | 15 | 22 | 0 | 9 | 0 | 0 |
| 345.134344 | 345.134362 | 8254174 | 19 | 22 | 0 | 6 | 0 | 0 |
| 345.147439 | 345.147249 | 1445342 | 16 | 27 | 0 | 6 | 0 | 1 |
| 345.155474 | 345.155491 | 2284255 | 16 | 26 | 0 | 8 | 0 | 0 |
| 345.170734 | 345.170747 | 8716000 | 20 | 26 | 0 | 5 | 0 | 0 |
| 345.19187 | 345.191877 | 1233121 | 17 | 30 | 0 | 7 | 0 | 0 |
| 345.207185 | 345.207133 | 2011106 | 21 | 30 | 0 | 4 | 0 | 0 |
| 347.07725 | 347.077241 | 2367331 | 17 | 16 | 0 | 8 | 0 | 0 |
| 347.113621 | 347.113627 | 10419045 | 18 | 20 | 0 | 7 | 0 | 0 |
| 347.126668 | 347.126513 | 1689830 | 15 | 25 | 0 | 7 | 0 | 1 |
| 347.186398 | 347.186398 | 14531434 | 20 | 28 | 0 | 5 | 0 | 0 |
| 347.222795 | 347.222783 | 1475181 | 21 | 32 | 0 | 4 | 0 | 0 |
| 348.108817 | 348.108876 | 1242923 | 17 | 19 | 1 | 7 | 0 | 0 |
| 348.145232 | 348.145261 | 1457838 | 18 | 23 | 1 | 6 | 0 | 0 |
| 349.092885 | 349.092891 | 5857266 | 17 | 18 | 0 | 8 | 0 | 0 |
| 349.129275 | 349.129277 | 19270644 | 18 | 22 | 0 | 7 | 0 | 0 |
| 349.142236 | 349.142163 | 1254389 | 15 | 27 | 0 | 7 | 0 | 1 |
| 349.16566 | 349.165662 | 23155704 | 19 | 26 | 0 | 6 | 0 | 0 |
| 349.238393 | 349.238433 | 1244412 | 21 | 34 | 0 | 4 | 0 | 0 |
| 350.124527 | 350.124526 | 1361085 | 17 | 21 | 1 | 7 | 0 | 0 |
| 351.072118 | 351.072156 | 1910403 | 16 | 16 | 0 | 9 | 0 | 0 |
| 351.108522 | 351.108541 | 17446022 | 17 | 20 | 0 | 8 | 0 | 0 |
| 351.144906 | 351.144927 | 34458760 | 18 | 24 | 0 | 7 | 0 | 0 |
| 352.140155 | 352.140176 | 1484244 | 17 | 23 | 1 | 7 | 0 | 0 |
| 353.087794 | 353.087806 | 5754141 | 16 | 18 | 0 | 9 | 0 | 0 |
| 353.124177 | 353.124191 | 26971424 | 17 | 22 | 0 | 8 | 0 | 0 |
| 353.139479 | 353.139447 | 1510048 | 21 | 22 | 0 | 5 | 0 | 0 |
| 353.160569 | 353.160577 | 27041058 | 18 | 26 | 0 | 7 | 0 | 0 |
| 353.175863 | 353.175833 | 1570211 | 22 | 26 | 0 | 4 | 0 | 0 |
| 353.196941 | 353.196962 | 12975397 | 19 | 30 | 0 | 6 | 0 | 0 |
| 355.103458 | 355.103456 | 8504763 | 16 | 20 | 0 | 9 | 0 | 0 |
| 355.118792 | 355.118712 | 2262972 | 20 | 20 | 0 | 6 | 0 | 0 |
| 355.131754 | 355.131599 | 1521597 | 17 | 25 | 0 | 6 | 0 | 1 |
| 355.139836 | 355.139841 | 18701758 | 17 | 24 | 0 | 8 | 0 | 0 |
| 355.155099 | 355.155097 | 2176575 | 21 | 24 | 0 | 5 | 0 | 0 |
| 355.168094 | 355.167984 | 1247296 | 18 | 29 | 0 | 5 | 0 | 1 |
| 355.176217 | 355.176227 | 11787713 | 18 | 28 | 0 | 7 | 0 | 0 |
| 355.191694 | 355.191483 | 1803330 | 22 | 28 | 0 | 4 | 0 | 0 |
| 355.212622 | 355.212612 | 4803012 | 19 | 32 | 0 | 6 | 0 | 0 |
| 357.097984 | 357.097976 | 2843486 | 19 | 18 | 0 | 7 | 0 | 0 |
| 357.111004 | 357.110863 | 1870687 | 16 | 23 | 0 | 7 | 0 | 1 |
| 357.119104 | 357.119106 | 6195807 | 16 | 22 | 0 | 9 | 0 | 0 |
| 357.134388 | 357.134362 | 4932705 | 20 | 22 | 0 | 6 | 0 | 0 |
| 357.1475 | 357.147249 | 1870178 | 17 | 27 | 0 | 6 | 0 | 1 |
| 357.155502 | 357.155491 | 8114786 | 17 | 26 | 0 | 8 | 0 | 0 |
| 357.17079 | 357.170747 | 3462244 | 21 | 26 | 0 | 5 | 0 | 0 |
| 357.191855 | 357.191877 | 4655205 | 18 | 30 | 0 | 7 | 0 | 0 |
| 357.207142 | 357.207133 | 2463591 | 22 | 30 | 0 | 4 | 0 | 0 |
| 359.07728 | 359.077241 | 1811971 | 18 | 16 | 0 | 8 | 0 | 0 |
| 359.113603 | 359.113627 | 7199495 | 19 | 20 | 0 | 7 | 0 | 0 |
| 359.126663 | 359.126513 | 2268680 | 16 | 25 | 0 | 7 | 0 | 1 |
| 359.134753 | 359.134756 | 2439944 | 16 | 24 | 0 | 9 | 0 | 0 |
| 359.150002 | 359.150012 | 10483466 | 20 | 24 | 0 | 6 | 0 | 0 |
| 359.163013 | 359.162899 | 1656843 | 17 | 29 | 0 | 6 | 0 | 1 |
| 359.17113 | 359.171141 | 2722828 | 17 | 28 | 0 | 8 | 0 | 0 |
| 359.186443 | 359.186398 | 5427469 | 21 | 28 | 0 | 5 | 0 | 0 |
| 359.207477 | 359.207527 | 1579279 | 18 | 32 | 0 | 7 | 0 | 0 |
| 359.222831 | 359.222783 | 1707792 | 22 | 32 | 0 | 4 | 0 | 0 |
| 361.092855 | 361.092891 | 5364658 | 18 | 18 | 0 | 8 | 0 | 0 |
| 361.105944 | 361.105778 | 1512371 | 15 | 23 | 0 | 8 | 0 | 1 |
| 361.129265 | 361.129277 | 14954421 | 19 | 22 | 0 | 7 | 0 | 0 |
| 361.142327 | 361.142163 | 2571702 | 16 | 27 | 0 | 7 | 0 | 1 |
| 361.165659 | 361.165662 | 19906488 | 20 | 26 | 0 | 6 | 0 | 0 |
| 361.178786 | 361.178549 | 1240633 | 17 | 31 | 0 | 6 | 0 | 1 |
| 361.202037 | 361.202048 | 6048699 | 21 | 30 | 0 | 5 | 0 | 0 |
| 361.238464 | 361.238433 | 1595070 | 22 | 34 | 0 | 4 | 0 | 0 |
| 362.124464 | 362.124526 | 1603340 | 18 | 21 | 1 | 7 | 0 | 0 |
| 362.160938 | 362.160911 | 1344015 | 19 | 25 | 1 | 6 | 0 | 0 |
| 363.0722 | 363.072156 | 2181985 | 17 | 16 | 0 | 9 | 0 | 0 |
| 363.108543 | 363.108541 | 12830820 | 18 | 20 | 0 | 8 | 0 | 0 |
| 363.121601 | 363.121428 | 1249125 | 15 | 25 | 0 | 8 | 0 | 1 |
| 363.144926 | 363.144927 | 27725928 | 19 | 24 | 0 | 7 | 0 | 0 |
| 363.181316 | 363.181312 | 27066474 | 20 | 28 | 0 | 6 | 0 | 0 |
| 363.217687 | 363.217698 | 4514926 | 21 | 32 | 0 | 5 | 0 | 0 |
| 364.140168 | 364.140176 | 1745729 | 18 | 23 | 1 | 7 | 0 | 0 |
| 365.087786 | 365.087806 | 6084888 | 17 | 18 | 0 | 9 | 0 | 0 |
| 365.160562 | 365.160577 | 38646048 | 19 | 26 | 0 | 7 | 0 | 0 |
| 365.23335 | 365.233348 | 3836709 | 21 | 34 | 0 | 5 | 0 | 0 |
| 366.155839 | 366.155826 | 1669754 | 18 | 25 | 1 | 7 | 0 | 0 |
| 367.103442 | 367.103456 | 14885330 | 17 | 20 | 0 | 9 | 0 | 0 |
| 367.118662 | 367.118712 | 1635796 | 21 | 20 | 0 | 6 | 0 | 0 |
| 367.13182 | 367.131599 | 1467221 | 18 | 25 | 0 | 6 | 0 | 1 |
| 367.139832 | 367.139841 | 40518100 | 18 | 24 | 0 | 8 | 0 | 0 |
| 367.155032 | 367.155097 | 1717207 | 22 | 24 | 0 | 5 | 0 | 0 |
| 367.1915 | 367.191483 | 1523419 | 23 | 28 | 0 | 4 | 0 | 0 |
| 368.133768 | 368.134058 | 1643315 | 11 | 24 | 5 | 7 | 0 | 1 |
| 369.082701 | 369.08272 | 3434638 | 16 | 18 | 0 | 10 | 0 | 0 |
| 369.097946 | 369.097976 | 1263119 | 20 | 18 | 0 | 7 | 0 | 0 |
| 369.111073 | 369.110863 | 1486736 | 17 | 23 | 0 | 7 | 0 | 1 |
| 369.119098 | 369.119106 | 17951376 | 17 | 22 | 0 | 9 | 0 | 0 |
| 369.134316 | 369.134362 | 2797202 | 21 | 22 | 0 | 6 | 0 | 0 |
| 369.147443 | 369.147249 | 2856596 | 18 | 27 | 0 | 6 | 0 | 1 |
| 369.155489 | 369.155491 | 23468692 | 18 | 26 | 0 | 8 | 0 | 0 |
| 369.170762 | 369.170747 | 2932886 | 22 | 26 | 0 | 5 | 0 | 0 |
| 369.183817 | 369.183634 | 1841303 | 19 | 31 | 0 | 5 | 0 | 1 |
| 369.207451 | 369.207133 | 1497369 | 23 | 30 | 0 | 4 | 0 | 0 |
| 369.228278 | 369.228262 | 5076124 | 20 | 34 | 0 | 6 | 0 | 0 |
| 371.077149 | 371.077241 | 1300686 | 19 | 16 | 0 | 8 | 0 | 0 |
| 371.090319 | 371.090128 | 1264847 | 16 | 21 | 0 | 8 | 0 | 1 |
| 371.098358 | 371.09837 | 3950928 | 16 | 20 | 0 | 10 | 0 | 0 |
| 371.113613 | 371.113627 | 4172113 | 20 | 20 | 0 | 7 | 0 | 0 |
| 371.126682 | 371.126513 | 3011411 | 17 | 25 | 0 | 7 | 0 | 1 |
| 371.134749 | 371.134756 | 10535763 | 17 | 24 | 0 | 9 | 0 | 0 |
| 371.149994 | 371.150012 | 4648277 | 21 | 24 | 0 | 6 | 0 | 0 |
| 371.163092 | 371.162899 | 3630934 | 18 | 29 | 0 | 6 | 0 | 1 |
| 371.171124 | 371.171141 | 9121623 | 18 | 28 | 0 | 8 | 0 | 0 |
| 371.18643 | 371.186398 | 3952984 | 22 | 28 | 0 | 5 | 0 | 0 |
| 371.207491 | 371.207527 | 4752731 | 19 | 32 | 0 | 7 | 0 | 0 |
| 371.223095 | 371.222783 | 1821468 | 23 | 32 | 0 | 4 | 0 | 0 |
| 373.09289 | 373.092891 | 3814931 | 19 | 18 | 0 | 8 | 0 | 0 |
| 373.105987 | 373.105778 | 1674773 | 16 | 23 | 0 | 8 | 0 | 1 |
| 373.114021 | 373.11402 | 2191893 | 16 | 22 | 0 | 10 | 0 | 0 |
| 373.129294 | 373.129277 | 8425495 | 20 | 22 | 0 | 7 | 0 | 0 |
| 373.142362 | 373.142163 | 2802712 | 17 | 27 | 0 | 7 | 0 | 1 |
| 373.150388 | 373.150406 | 3700761 | 17 | 26 | 0 | 9 | 0 | 0 |
| 373.165673 | 373.165662 | 9237530 | 21 | 26 | 0 | 6 | 0 | 0 |
| 373.178693 | 373.178549 | 2184476 | 18 | 31 | 0 | 6 | 0 | 1 |
| 373.202097 | 373.202048 | 4483102 | 22 | 30 | 0 | 5 | 0 | 0 |
| 373.238392 | 373.238433 | 1718050 | 23 | 34 | 0 | 4 | 0 | 0 |
| 374.160874 | 374.160911 | 1246333 | 20 | 25 | 1 | 6 | 0 | 0 |
| 375.072159 | 375.072156 | 1583832 | 18 | 16 | 0 | 9 | 0 | 0 |
| 375.108526 | 375.108541 | 8849628 | 19 | 20 | 0 | 8 | 0 | 0 |
| 375.121616 | 375.121428 | 2234077 | 16 | 25 | 0 | 8 | 0 | 1 |
| 375.144924 | 375.144927 | 17921248 | 20 | 24 | 0 | 7 | 0 | 0 |
| 375.157996 | 375.157813 | 2587105 | 17 | 29 | 0 | 7 | 0 | 1 |
| 375.181312 | 375.181312 | 13614307 | 21 | 28 | 0 | 6 | 0 | 0 |
| 375.217718 | 375.217698 | 5145831 | 22 | 32 | 0 | 5 | 0 | 0 |
| 375.254055 | 375.254083 | 1556714 | 23 | 36 | 0 | 4 | 0 | 0 |
| 376.140246 | 376.140176 | 1872964 | 19 | 23 | 1 | 7 | 0 | 0 |
| 376.176565 | 376.176561 | 1750855 | 20 | 27 | 1 | 6 | 0 | 0 |
| 377.08781 | 377.087806 | 4841380 | 18 | 18 | 0 | 9 | 0 | 0 |
| 377.124191 | 377.124191 | 22644136 | 19 | 22 | 0 | 8 | 0 | 0 |
| 377.137214 | 377.137078 | 2120873 | 16 | 27 | 0 | 8 | 0 | 1 |
| 377.160573 | 377.160577 | 31827370 | 20 | 26 | 0 | 7 | 0 | 0 |
| 377.173655 | 377.173463 | 1857196 | 17 | 31 | 0 | 7 | 0 | 1 |
| 377.196949 | 377.196962 | 14183855 | 21 | 30 | 0 | 6 | 0 | 0 |
| 377.233358 | 377.233348 | 3453874 | 22 | 34 | 0 | 5 | 0 | 0 |
| 378.119571 | 378.11944 | 1351181 | 18 | 21 | 1 | 8 | 0 | 0 |
| 378.155808 | 378.155826 | 2033168 | 19 | 25 | 1 | 7 | 0 | 0 |
| 379.103461 | 379.103456 | 12596850 | 18 | 20 | 0 | 9 | 0 | 0 |
| 379.13984 | 379.139841 | 39237236 | 19 | 24 | 0 | 8 | 0 | 0 |
| 379.212612 | 379.212612 | 10120829 | 21 | 32 | 0 | 6 | 0 | 0 |
| 379.248884 | 379.248998 | 1755009 | 22 | 36 | 0 | 5 | 0 | 0 |
| 380.135079 | 380.13509 | 1755868 | 18 | 23 | 1 | 8 | 0 | 0 |
| 380.171438 | 380.171476 | 1668832 | 19 | 27 | 1 | 7 | 0 | 0 |
| 381.08273 | 381.08272 | 3737406 | 17 | 18 | 0 | 10 | 0 | 0 |
| 381.119109 | 381.119106 | 26478402 | 18 | 22 | 0 | 9 | 0 | 0 |
| 381.134426 | 381.134362 | 1498307 | 22 | 22 | 0 | 6 | 0 | 0 |
| 381.147437 | 381.147249 | 1696197 | 19 | 27 | 0 | 6 | 0 | 1 |
| 381.155496 | 381.155491 | 39941956 | 19 | 26 | 0 | 8 | 0 | 0 |
| 381.19188 | 381.191877 | 27080522 | 20 | 30 | 0 | 7 | 0 | 0 |
| 381.228257 | 381.228262 | 5027661 | 21 | 34 | 0 | 6 | 0 | 0 |
| 382.114375 | 382.114355 | 1372586 | 17 | 21 | 1 | 9 | 0 | 0 |
| 383.098355 | 383.09837 | 8970256 | 17 | 20 | 0 | 10 | 0 | 0 |
| 383.11364 | 383.113627 | 2189074 | 21 | 20 | 0 | 7 | 0 | 0 |
| 383.126677 | 383.126513 | 3218451 | 18 | 25 | 0 | 7 | 0 | 1 |
| 383.134746 | 383.134756 | 28096532 | 18 | 24 | 0 | 9 | 0 | 0 |
| 383.149993 | 383.150012 | 3305494 | 22 | 24 | 0 | 6 | 0 | 0 |
| 383.163073 | 383.162899 | 4531223 | 19 | 29 | 0 | 6 | 0 | 1 |
| 383.171123 | 383.171141 | 24791064 | 19 | 28 | 0 | 8 | 0 | 0 |
| 383.186365 | 383.186398 | 2971162 | 23 | 28 | 0 | 5 | 0 | 0 |
| 383.207509 | 383.207527 | 15319068 | 20 | 32 | 0 | 7 | 0 | 0 |
| 383.24387 | 383.243912 | 2032416 | 21 | 36 | 0 | 6 | 0 | 0 |
| 384.145309 | 384.145261 | 1428094 | 21 | 23 | 1 | 6 | 0 | 0 |
| 385.077551 | 385.077635 | 1373665 | 16 | 18 | 0 | 11 | 0 | 0 |
| 385.092912 | 385.092891 | 2715106 | 20 | 18 | 0 | 8 | 0 | 0 |
| 385.105948 | 385.105778 | 2719460 | 17 | 23 | 0 | 8 | 0 | 1 |
| 385.114008 | 385.11402 | 9333988 | 17 | 22 | 0 | 10 | 0 | 0 |
| 385.129285 | 385.129277 | 5438694 | 21 | 22 | 0 | 7 | 0 | 0 |
| 385.142354 | 385.142163 | 6263016 | 18 | 27 | 0 | 7 | 0 | 1 |
| 385.150404 | 385.150406 | 16072936 | 18 | 26 | 0 | 9 | 0 | 0 |
| 385.160098 | 385.159789 | 1564137 | 29 | 22 | 0 | 1 | 0 | 0 |
| 385.165667 | 385.165662 | 6158570 | 22 | 26 | 0 | 6 | 0 | 0 |
| 385.178718 | 385.178549 | 7382252 | 19 | 31 | 0 | 6 | 0 | 1 |
| 385.186779 | 385.186791 | 11192556 | 19 | 30 | 0 | 8 | 0 | 0 |
| 385.202016 | 385.202048 | 4344046 | 23 | 30 | 0 | 5 | 0 | 0 |
| 385.23856 | 385.238433 | 2163442 | 24 | 34 | 0 | 4 | 0 | 0 |
| 387.072113 | 387.072156 | 1520820 | 19 | 16 | 0 | 9 | 0 | 0 |
| 387.093174 | 387.093285 | 1304502 | 16 | 20 | 0 | 11 | 0 | 0 |
| 387.108554 | 387.108541 | 6479288 | 20 | 20 | 0 | 8 | 0 | 0 |
| 387.121618 | 387.121428 | 3962809 | 17 | 25 | 0 | 8 | 0 | 1 |
| 387.129666 | 387.129671 | 4279738 | 17 | 24 | 0 | 10 | 0 | 0 |
| 387.139423 | 387.139053 | 1854395 | 28 | 20 | 0 | 2 | 0 | 0 |
| 387.144902 | 387.144927 | 11256252 | 21 | 24 | 0 | 7 | 0 | 0 |
| 387.15798 | 387.157813 | 6684093 | 18 | 29 | 0 | 7 | 0 | 1 |
| 387.16605 | 387.166056 | 5408190 | 18 | 28 | 0 | 9 | 0 | 0 |
| 387.175757 | 387.175439 | 2396351 | 29 | 24 | 0 | 1 | 0 | 0 |
| 387.181304 | 387.181312 | 9420224 | 22 | 28 | 0 | 6 | 0 | 0 |
| 387.19437 | 387.194199 | 5046721 | 19 | 33 | 0 | 6 | 0 | 1 |
| 387.217702 | 387.217698 | 5475780 | 23 | 32 | 0 | 5 | 0 | 0 |
| 387.254032 | 387.254083 | 1691207 | 24 | 36 | 0 | 4 | 0 | 0 |
| 388.140211 | 388.140176 | 1670438 | 20 | 23 | 1 | 7 | 0 | 0 |
| 389.087826 | 389.087806 | 4029067 | 19 | 18 | 0 | 9 | 0 | 0 |
| 389.100841 | 389.100692 | 1383436 | 16 | 23 | 0 | 9 | 0 | 1 |
| 389.118603 | 389.118318 | 1538190 | 27 | 18 | 0 | 3 | 0 | 0 |
| 389.124186 | 389.124191 | 13982351 | 20 | 22 | 0 | 8 | 0 | 0 |
| 389.137269 | 389.137078 | 3924624 | 17 | 27 | 0 | 8 | 0 | 1 |
| 389.145268 | 389.145321 | 1259153 | 17 | 26 | 0 | 10 | 0 | 0 |
| 389.155015 | 389.154703 | 1589906 | 28 | 22 | 0 | 2 | 0 | 0 |
| 389.160578 | 389.160577 | 19155602 | 21 | 26 | 0 | 7 | 0 | 0 |
| 389.173665 | 389.173463 | 4053652 | 18 | 31 | 0 | 7 | 0 | 1 |
| 389.181717 | 389.181706 | 1785749 | 18 | 30 | 0 | 9 | 0 | 0 |
| 389.196966 | 389.196962 | 13091479 | 22 | 30 | 0 | 6 | 0 | 0 |
| 389.233353 | 389.233348 | 4295835 | 23 | 34 | 0 | 5 | 0 | 0 |
| 391.067025 | 391.06707 | 1321567 | 18 | 16 | 0 | 10 | 0 | 0 |
| 391.103442 | 391.103456 | 8520547 | 19 | 20 | 0 | 9 | 0 | 0 |
| 391.116662 | 391.116342 | 1472484 | 16 | 25 | 0 | 9 | 0 | 1 |
| 391.139848 | 391.139841 | 26360678 | 20 | 24 | 0 | 8 | 0 | 0 |
| 391.152898 | 391.152728 | 1997672 | 17 | 29 | 0 | 8 | 0 | 1 |
| 391.176241 | 391.176227 | 23587690 | 21 | 28 | 0 | 7 | 0 | 0 |
| 391.189337 | 391.189113 | 1449836 | 18 | 33 | 0 | 7 | 0 | 1 |
| 391.212605 | 391.212612 | 12196719 | 22 | 32 | 0 | 6 | 0 | 0 |
| 391.248968 | 391.248998 | 2664307 | 23 | 36 | 0 | 5 | 0 | 0 |
| 392.135071 | 392.13509 | 1481426 | 19 | 23 | 1 | 8 | 0 | 0 |
| 392.171401 | 392.171476 | 1653718 | 20 | 27 | 1 | 7 | 0 | 0 |
| 393.0827 | 393.08272 | 2995768 | 18 | 18 | 0 | 10 | 0 | 0 |
| 393.119109 | 393.119106 | 20171836 | 19 | 22 | 0 | 9 | 0 | 0 |
| 393.155503 | 393.155491 | 36797504 | 20 | 26 | 0 | 8 | 0 | 0 |
| 393.191881 | 393.191877 | 19774532 | 21 | 30 | 0 | 7 | 0 | 0 |
| 393.228272 | 393.228262 | 6155336 | 22 | 34 | 0 | 6 | 0 | 0 |
| 394.150759 | 394.15074 | 1656491 | 19 | 25 | 1 | 8 | 0 | 0 |
| 394.187073 | 394.187126 | 1425583 | 20 | 29 | 1 | 7 | 0 | 0 |
| 395.098386 | 395.09837 | 8302865 | 18 | 20 | 0 | 10 | 0 | 0 |
| 395.126787 | 395.126513 | 1664276 | 19 | 25 | 0 | 7 | 0 | 1 |
| 395.134761 | 395.134756 | 32967958 | 19 | 24 | 0 | 9 | 0 | 0 |
| 395.149906 | 395.150012 | 1306391 | 23 | 24 | 0 | 6 | 0 | 0 |
| 395.171137 | 395.171141 | 35028248 | 20 | 28 | 0 | 8 | 0 | 0 |
| 395.186759 | 395.186398 | 1333275 | 24 | 28 | 0 | 5 | 0 | 0 |
| 395.207535 | 395.207527 | 11994397 | 21 | 32 | 0 | 7 | 0 | 0 |
| 395.222848 | 395.222783 | 1519775 | 25 | 32 | 0 | 4 | 0 | 0 |
| 395.243945 | 395.243912 | 3060001 | 22 | 36 | 0 | 6 | 0 | 0 |
| 396.166419 | 396.16639 | 1267973 | 19 | 27 | 1 | 8 | 0 | 0 |
| 397.077561 | 397.077635 | 2554343 | 17 | 18 | 0 | 11 | 0 | 0 |
| 397.09289 | 397.092891 | 1355753 | 21 | 18 | 0 | 8 | 0 | 0 |
| 397.105989 | 397.105778 | 1688042 | 18 | 23 | 0 | 8 | 0 | 1 |
| 397.11401 | 397.11402 | 15001067 | 18 | 22 | 0 | 10 | 0 | 0 |
| 397.129337 | 397.129277 | 2926061 | 22 | 22 | 0 | 7 | 0 | 0 |
| 397.142396 | 397.142163 | 3975662 | 19 | 27 | 0 | 7 | 0 | 1 |
| 397.1504 | 397.150406 | 29302254 | 19 | 26 | 0 | 9 | 0 | 0 |
| 397.165636 | 397.165662 | 3052017 | 23 | 26 | 0 | 6 | 0 | 0 |
| 397.178738 | 397.178549 | 2224626 | 20 | 31 | 0 | 6 | 0 | 1 |
| 397.186769 | 397.186791 | 20958706 | 20 | 30 | 0 | 8 | 0 | 0 |
| 397.202123 | 397.202048 | 1910388 | 24 | 30 | 0 | 5 | 0 | 0 |
| 397.223155 | 397.223177 | 5658615 | 21 | 34 | 0 | 7 | 0 | 0 |
| 399.093269 | 399.093285 | 3908801 | 17 | 20 | 0 | 11 | 0 | 0 |
| 399.108524 | 399.108541 | 2952898 | 21 | 20 | 0 | 8 | 0 | 0 |
| 399.121597 | 399.121428 | 3908292 | 18 | 25 | 0 | 8 | 0 | 1 |
| 399.129659 | 399.129671 | 12355268 | 18 | 24 | 0 | 10 | 0 | 0 |
| 399.144917 | 399.144927 | 5506758 | 22 | 24 | 0 | 7 | 0 | 0 |
| 399.158009 | 399.157813 | 7812808 | 19 | 29 | 0 | 7 | 0 | 1 |
| 399.166056 | 399.166056 | 13958856 | 19 | 28 | 0 | 9 | 0 | 0 |
| 399.181321 | 399.181312 | 5376714 | 23 | 28 | 0 | 6 | 0 | 0 |
| 399.194386 | 399.194199 | 1995979 | 20 | 33 | 0 | 6 | 0 | 1 |
| 399.217678 | 399.217698 | 3761358 | 24 | 32 | 0 | 5 | 0 | 0 |
| 399.238884 | 399.238827 | 2147792 | 21 | 36 | 0 | 7 | 0 | 0 |
| 399.254128 | 399.254083 | 1528786 | 25 | 36 | 0 | 4 | 0 | 0 |
| 401.08781 | 401.087806 | 2523544 | 20 | 18 | 0 | 9 | 0 | 0 |
| 401.10084 | 401.100692 | 2520473 | 17 | 23 | 0 | 9 | 0 | 1 |
| 401.10886 | 401.108935 | 2214810 | 17 | 22 | 0 | 11 | 0 | 0 |
| 401.12419 | 401.124191 | 7938972 | 21 | 22 | 0 | 8 | 0 | 0 |
| 401.137251 | 401.137078 | 5547933 | 18 | 27 | 0 | 8 | 0 | 1 |
| 401.145324 | 401.145321 | 5099934 | 18 | 26 | 0 | 10 | 0 | 0 |
| 401.155059 | 401.154703 | 2016671 | 29 | 22 | 0 | 2 | 0 | 0 |
| 401.160568 | 401.160577 | 10772383 | 22 | 26 | 0 | 7 | 0 | 0 |
| 401.173639 | 401.173463 | 12175265 | 19 | 31 | 0 | 7 | 0 | 1 |
| 401.181709 | 401.181706 | 5652386 | 19 | 30 | 0 | 9 | 0 | 0 |
| 401.196965 | 401.196962 | 9369507 | 23 | 30 | 0 | 6 | 0 | 0 |
| 401.210011 | 401.209849 | 1529253 | 20 | 35 | 0 | 6 | 0 | 1 |
| 401.233369 | 401.233348 | 5521320 | 24 | 34 | 0 | 5 | 0 | 0 |
| 402.155938 | 402.155826 | 1281291 | 21 | 25 | 1 | 7 | 0 | 0 |
| 403.10345 | 403.103456 | 6524017 | 20 | 20 | 0 | 9 | 0 | 0 |
| 403.116546 | 403.116342 | 2442354 | 17 | 25 | 0 | 9 | 0 | 1 |
| 403.134304 | 403.133968 | 1814900 | 28 | 20 | 0 | 3 | 0 | 0 |
| 403.139833 | 403.139841 | 16787572 | 21 | 24 | 0 | 8 | 0 | 0 |
| 403.152913 | 403.152728 | 5384310 | 18 | 29 | 0 | 8 | 0 | 1 |
| 403.160994 | 403.160971 | 1837559 | 18 | 28 | 0 | 10 | 0 | 0 |
| 403.170697 | 403.170354 | 4263032 | 29 | 24 | 0 | 2 | 0 | 0 |
| 403.176239 | 403.176227 | 19906680 | 22 | 28 | 0 | 7 | 0 | 0 |
| 403.18929 | 403.189113 | 6667386 | 19 | 33 | 0 | 7 | 0 | 1 |
| 403.212609 | 403.212612 | 12189820 | 23 | 32 | 0 | 6 | 0 | 0 |
| 403.24903 | 403.248998 | 4466816 | 24 | 36 | 0 | 5 | 0 | 0 |
| 404.135102 | 404.13509 | 1930207 | 20 | 23 | 1 | 8 | 0 | 0 |
| 404.171398 | 404.171476 | 1575651 | 21 | 27 | 1 | 7 | 0 | 0 |
| 405.082706 | 405.08272 | 3225925 | 19 | 18 | 0 | 10 | 0 | 0 |
| 405.113512 | 405.113233 | 1485128 | 27 | 18 | 0 | 4 | 0 | 0 |
| 405.119092 | 405.119106 | 16296265 | 20 | 22 | 0 | 9 | 0 | 0 |
| 405.132116 | 405.131993 | 2099530 | 17 | 27 | 0 | 9 | 0 | 1 |
| 405.150025 | 405.150304 | 1422668 | 21 | 22 | 6 | 1 | 1 | 0 |
| 405.155481 | 405.155491 | 31560012 | 21 | 26 | 0 | 8 | 0 | 0 |
| 405.168523 | 405.168378 | 3319118 | 18 | 31 | 0 | 8 | 0 | 1 |
| 405.186346 | 405.186004 | 2502992 | 29 | 26 | 0 | 2 | 0 | 0 |
| 405.204965 | 405.204764 | 2264658 | 19 | 35 | 0 | 7 | 0 | 1 |
| 405.228241 | 405.228262 | 10160468 | 23 | 34 | 0 | 6 | 0 | 0 |
| 405.264735 | 405.264648 | 2662233 | 24 | 38 | 0 | 5 | 0 | 0 |
| 406.150691 | 406.15074 | 2042551 | 20 | 25 | 1 | 8 | 0 | 0 |
| 406.187211 | 406.187126 | 1343163 | 21 | 29 | 1 | 7 | 0 | 0 |
| 407.098357 | 407.09837 | 9088540 | 19 | 20 | 0 | 10 | 0 | 0 |
| 407.134732 | 407.134756 | 36114976 | 20 | 24 | 0 | 9 | 0 | 0 |
| 407.147806 | 407.147643 | 1349410 | 17 | 29 | 0 | 9 | 0 | 1 |
| 407.17113 | 407.171141 | 39440932 | 21 | 28 | 0 | 8 | 0 | 0 |
| 407.20752 | 407.207527 | 20353576 | 22 | 32 | 0 | 7 | 0 | 0 |
| 407.243887 | 407.243912 | 6100524 | 23 | 36 | 0 | 6 | 0 | 0 |
| 408.129999 | 408.130005 | 1694858 | 19 | 23 | 1 | 9 | 0 | 0 |
| 408.166379 | 408.16639 | 1942158 | 20 | 27 | 1 | 8 | 0 | 0 |
| 409.077638 | 409.077635 | 2125550 | 18 | 18 | 0 | 11 | 0 | 0 |
| 409.106005 | 409.105778 | 1294321 | 19 | 23 | 0 | 8 | 0 | 1 |
| 409.114015 | 409.11402 | 22582002 | 19 | 22 | 0 | 10 | 0 | 0 |
| 409.129276 | 409.129277 | 1822452 | 23 | 22 | 0 | 7 | 0 | 0 |
| 409.142398 | 409.142163 | 2282485 | 20 | 27 | 0 | 7 | 0 | 1 |
| 409.150392 | 409.150406 | 47076088 | 20 | 26 | 0 | 9 | 0 | 0 |
| 409.165597 | 409.165662 | 1490680 | 24 | 26 | 0 | 6 | 0 | 0 |
| 409.178851 | 409.178549 | 1732345 | 21 | 31 | 0 | 6 | 0 | 1 |
| 409.186788 | 409.186791 | 29922042 | 21 | 30 | 0 | 8 | 0 | 0 |
| 409.201965 | 409.202048 | 1596668 | 25 | 30 | 0 | 5 | 0 | 0 |
| 409.22316 | 409.223177 | 11395838 | 22 | 34 | 0 | 7 | 0 | 0 |
| 409.238388 | 409.238433 | 1534463 | 26 | 34 | 0 | 4 | 0 | 0 |
| 409.259526 | 409.259562 | 2811138 | 23 | 38 | 0 | 6 | 0 | 0 |
| 411.093269 | 411.093285 | 6012867 | 18 | 20 | 0 | 11 | 0 | 0 |
| 411.108547 | 411.108541 | 1638853 | 22 | 20 | 0 | 8 | 0 | 0 |
| 411.121658 | 411.121428 | 2872262 | 19 | 25 | 0 | 8 | 0 | 1 |
| 411.127568 | 411.127854 | 1412551 | 16 | 24 | 6 | 3 | 2 | 0 |
| 411.12967 | 411.129671 | 26878918 | 19 | 24 | 0 | 10 | 0 | 0 |
| 411.144963 | 411.144927 | 2910664 | 23 | 24 | 0 | 7 | 0 | 0 |
| 411.157993 | 411.157813 | 3647946 | 20 | 29 | 0 | 7 | 0 | 1 |
| 411.166048 | 411.166056 | 29860810 | 20 | 28 | 0 | 9 | 0 | 0 |
| 411.181316 | 411.181312 | 3160012 | 24 | 28 | 0 | 6 | 0 | 0 |
| 411.194372 | 411.194199 | 2369486 | 21 | 33 | 0 | 6 | 0 | 1 |
| 411.202423 | 411.202442 | 14136270 | 21 | 32 | 0 | 8 | 0 | 0 |
| 411.217791 | 411.217698 | 2449872 | 25 | 32 | 0 | 5 | 0 | 0 |
| 411.238836 | 411.238827 | 5103570 | 22 | 36 | 0 | 7 | 0 | 0 |
| 411.254266 | 411.254083 | 1333652 | 26 | 36 | 0 | 4 | 0 | 0 |
| 413.087826 | 413.087806 | 1349652 | 21 | 18 | 0 | 9 | 0 | 0 |
| 413.100858 | 413.100692 | 2292885 | 18 | 23 | 0 | 9 | 0 | 1 |
| 413.108947 | 413.108935 | 7217302 | 18 | 22 | 0 | 11 | 0 | 0 |
| 413.124198 | 413.124191 | 4205720 | 22 | 22 | 0 | 8 | 0 | 0 |
| 413.137261 | 413.137078 | 5701785 | 19 | 27 | 0 | 8 | 0 | 1 |
| 413.145318 | 413.145321 | 14804121 | 19 | 26 | 0 | 10 | 0 | 0 |
| 413.160569 | 413.160577 | 6217883 | 23 | 26 | 0 | 7 | 0 | 0 |
| 413.17363 | 413.173463 | 4140701 | 20 | 31 | 0 | 7 | 0 | 1 |
| 413.181704 | 413.181706 | 14298269 | 20 | 30 | 0 | 9 | 0 | 0 |
| 413.196961 | 413.196962 | 5258399 | 24 | 30 | 0 | 6 | 0 | 0 |
| 413.209966 | 413.209849 | 1895072 | 21 | 35 | 0 | 6 | 0 | 1 |
| 413.218114 | 413.218092 | 6272161 | 21 | 34 | 0 | 8 | 0 | 0 |
| 413.233397 | 413.233348 | 2596771 | 25 | 34 | 0 | 5 | 0 | 0 |
| 415.103416 | 415.103456 | 3916132 | 21 | 20 | 0 | 9 | 0 | 0 |
| 415.116505 | 415.116342 | 3687270 | 18 | 25 | 0 | 9 | 0 | 1 |
| 415.124599 | 415.124585 | 4248423 | 18 | 24 | 0 | 11 | 0 | 0 |
| 415.13431 | 415.133968 | 2046312 | 29 | 20 | 0 | 3 | 0 | 0 |
| 415.139817 | 415.139841 | 7875944 | 22 | 24 | 0 | 8 | 0 | 0 |
| 415.152917 | 415.152728 | 7581034 | 19 | 29 | 0 | 8 | 0 | 1 |
| 415.160979 | 415.160971 | 6483306 | 19 | 28 | 0 | 10 | 0 | 0 |
| 415.176228 | 415.176227 | 10165612 | 23 | 28 | 0 | 7 | 0 | 0 |
| 415.189301 | 415.189113 | 3122541 | 20 | 33 | 0 | 7 | 0 | 1 |
| 415.197327 | 415.197356 | 5765486 | 20 | 32 | 0 | 9 | 0 | 0 |
| 415.212609 | 415.212612 | 8564080 | 24 | 32 | 0 | 6 | 0 | 0 |
| 415.249036 | 415.248998 | 3522419 | 25 | 36 | 0 | 5 | 0 | 0 |
| 416.17143 | 416.171476 | 1605330 | 22 | 27 | 1 | 7 | 0 | 0 |
[truncated: 376,824 more chars]
